# Integrative analysis of genome‐wide lncRNA and mRNA expression in newly synthesized *Brassica* hexaploids

**DOI:** 10.1002/ece3.4152

**Published:** 2018-05-15

**Authors:** Ruihua Wang, Jun Zou, Jinling Meng, Jianbo Wang

**Affiliations:** ^1^ State Key Laboratory of Hybrid Rice Department of Plant Science College of Life Sciences Wuhan University Wuhan China; ^2^ National Key Laboratory of Crop Genetic Improvement College of Plant Science and Technology Huazhong Agricultural University Wuhan China

**Keywords:** *Brassica* hexaploid, expression pattern, gene expression, high‐throughput sequencing, lncRNA, miRNA

## Abstract

Polyploidization, as a significant evolution force, has been considered to facilitate plant diversity. The expression levels of lncRNAs and how they control the expression of protein‐coding genes in allopolyploids remain largely unknown. In this study, lncRNA expression profiles were compared between *Brassica* hexaploid and its parents using a high‐throughput sequencing approach. A total of 2,725, 1,672, and 2,810 lncRNAs were discovered in *Brassica rapa*,* Brassica carinata*, and *Brassica* hexaploid, respectively. It was also discovered that 725 lncRNAs were differentially expressed between *Brassica* hexaploid and its parents, and 379 lncRNAs were nonadditively expressed in this hexaploid. LncRNAs have multiple expression patterns between *Brassica* hexaploid and its parents and show paternal parent‐biased expression. These lncRNAs were found to implement regulatory functions directly in the long‐chain form, and acted as precursors or targets of miRNAs. According to the prediction of the targets of differentially expressed lncRNAs, 109 lncRNAs were annotated, and their target genes were involved in the metabolic process, pigmentation, reproduction, exposure to stimulus, biological regulation, and so on. Compared with the paternal parent, differentially expressed lncRNAs between *Brassica* hexaploid and its maternal parent participated in more regulation pathways. Additionally, 61 lncRNAs were identified as putative targets of known miRNAs, and 15 other lncRNAs worked as precursors of miRNAs. Some conservative motifs of lncRNAs from different groups were detected, which indicated that these motifs could be responsible for their regulatory roles. Our findings may provide a reference for the further study of the function and action mechanisms of lncRNAs during plant evolution.

## 1 INTRODUCTION

It is believed that all flowering plants have experienced one or several rounds of genome duplication in the process of evolution (Jiao et al., 2011). Allopolyploids that are widely distributed in the world originate from crossing different species followed by chromosome doubling or fusing unreduced gametes between different species (Chen, 2010), and contain some commercial crops, such as cotton, oilseed rape, wheat, and coffee. Because of intergenomic interactions and heterozygosity, they possess various phenotypes and growth vigor (Ni et al., 2009) and have a number of advantages over their parents, such as improved ability to survive in harsh environmental conditions, increased resistance to pathogens, and other superior traits (Aversano et al., 2012). They are born with a succession of non‐Mendelian interactions and processes, including recombination between homeologous chromosomes (Gaeta, Pires, Iniguez‐Luy, Leon, & Osborn, 2007), chromosomal rearrangement (Xiong, Gaeta, & Pires, 2011), DNA sequence elimination (Kashkush, Feldman, & Levy, 2002), and gene epigenetic modification (Madlung et al., 2002). In terms of gene expression, the increase of genetic information in allopolyploids gives rise to transcriptome pattern differences compared with its parental species (Fujimoto, Taylor, Sasaki, Kawanabe, & Dennis, 2011). Transcriptome changes could promote the construction of gene expression programs and the production of stable species (Coate & Doyle, 2010). Recent studies have detected the variations of gene expression in allopolyploids (Han et al., 2016; Qi et al., 2012; Tanaka et al., 2015), and the gene expression differences probably vary from species to species.

Long noncoding RNAs (lncRNAs) are RNA molecules that are at least 200 nucleotides in length, lack a protein‐coding capacity and play vital regulatory roles in a wide range of biological processes in plants and animals (Wang, Yuan et al., 2015). They are subject to strict regulation at the transcriptional and post‐transcriptional level, implying that they could possess important regulatory functions in organisms (Shafiq, Li, & Sun, 2016). Based on genomic locations relative to neighboring genes, they can be classified as sense, natural antisense, intronic, or intergenic lncRNAs (Ariel, Romero‐Barrios, Jegu, Benhamed, & Crespi, 2015). The majority of identified lncRNAs are transcribed by RNA polymerase II in mammals; in addition to RNA polymerase II, plant‐specific RNA polymerase IV and V also play important role in their production (Wierzbicki, Haag, & Pikaard, 2008). A recent report also showed that some lncRNAs might be the transcriptional products of polymerase III in Arabidopsis (Wu, Ma, Chen, Wang, & Wang, 2012; Wu, Liu et al., 2012; Wu, Okada et al., 2012). RNA‐seq provides information on genome‐wide lncRNA expression and has very low background signal, more accurate quantification, and high levels of reproducibility. A mass of lncRNAs have been identified by RNA‐seq in rice (Zhang, Liao et al., 2014), *Populus trichocarpa* (Chen, Wang, Bao, Chen, & Wang, 2016), *Medicago* (Wang, Liu, Zhao, Chen, & Zhang, 2015), and so on. They were found to be involved in controlling flowering time (Zhang, Mujahid, Hou, Nallamilli, & Peng, 2013), regulation of photoperiodic‐sensitive male sterility (Ding et al., 2012), response to pathogen invasion (Xin et al., 2011), nodule organogenesis (Sousa et al., 2001), and take part in the diverse biological pathways of plants.

LncRNAs regulate gene expression on multiple levels via abundant complex mechanisms. They could increase the expression of target genes by reinforcing the accessibility of these genes to RNA polymerase (Hirota et al., 2008) or inhibiting gene expression by preventing the formation of the transcription initiation complex (Martianov, Ramadass, Barros, Chow, & Akoulitchev, 2007). Some of them may control transcription elongation by blocking the RNA polymerase activities to regulate target gene expression (Chekanova, 2015). In addition, lncRNAs can also adjust target gene expression via trans‐action. They bring about mRNA degradation (Golden, Gerbasi, & Sontheimer, 2008) or protect mRNAs from miRNA‐mediated degradation (Faghihi et al., 2010) by base complementary, and they facilitate mRNA translation (Carrieri et al., 2012) or interdict mRNA translation (Kawano, Aravind, & Storz, 2007) by integrating with the 5′ part of the target mRNA. A few studies suggest that a small number of them could act as miRNA precursors (Jalali, Jayaraj, & Scaria, 2012; Chen, Fu et al., 2015; Chen, Quan, & Zhang, 2015; Chen et al., 2016).

In plants, the action mechanisms of some lncRNAs have clearly been studied, proving the various ways in which lncRNAs regulate biological processes. For example, *COLDAIR* (an intronic lncRNA) recruits PRC2, a chromatin remodeling complex, to the *Flowering Locus C* (*FLC*) gene, which represses the transcription of *FLC* (Heo & Sung, 2011). *APOLO* (auxin regulated promoter loop), an intergenic lncRNA, is involved in the formation of chromatin loops, repressing the transcription of the *PINOID* gene (Ariel et al., 2014). In *Medicago truncatula*,* ENOD40* (an antisense lncRNA), as molecular cargos for protein re‐location, interacts with RNA binding protein 1 (MtRBP1) during nodulation, making MtRBP1 re‐locate from the nuclear speckle into the cytoplasmic granule (Campalans, Kondorosi, & Crespi, 2004).

The genus *Brassica* is usually regarded as a model system to study genomic changes during the early stages of polyploidization. Steady and directed genetic modifications have been found in *Brassica* polyploids (Song, Lu, Tang, & Osborn, 1995). The U‐triangle intuitively shows the relationships among three ancestral *Brassica* diploid species (*B. rapa*,* B. nigra*, and *B. oleracea*) and three *Brassica* allotetraploid species (*B. juncea*,* B. carinata*, and *B. napus*) (Nagaharu, 1935). The allotetraploids are generated by hybridization among three diploid species following genome doubling. The trigenomic allohexaploid is artificially synthesized by the crossing between diploid *B. rapa* and allotetraploid *B. carinata* followed by chromosome doubling (Tian et al., 2010). It possesses a higher level of redundancy and heterozygosity compared with allotetraploid, while expansive transcriptome alternations (Zhao, Zou, Meng, Mei, & Wang, 2013) and dynamic miRNA expression patterns, compared with its parents, had been identified in this *Brassica* hexaploid (Shen et al., 2014). In addition, the effects of allopolyploidization on proteomic divergence also have been explored. *Brassica* hexaploid showed a protein expression level dominance bias toward maternal parents (*B. carinata*) and nonadditive expression patterns (Shen, Zhang, Zou, Meng, & Wang, 2015). For *Brassica* allopolyploidy, increased heterozygosity and flexibility is conducive to their survival in a broader range of living conditions. However, little information on lncRNAs in this hexaploid is available. RNA‐seq has provided a powerful approach for exploring transcriptome changes and gene function annotation.

In this study, lncRNAs were identified and characterized on a genomic scale by RNA‐seq in *Brassica* hexaploid and its parents. LncRNA expression patterns and the action of lncRNAs in the regulation of gene expression were explored. After allopolyploidization, lncRNAs showed paternal parent‐biased expression and nonadditive expression in *Brassica* hexaploid; moreover, inhibition could be a pattern of nonadditive lncRNA regulation. In addition, the interaction networks between lncRNAs and mRNAs were constructed and the function of lncRNAs was then investigated based on the lncRNA‐mRNA interaction networks. To explore the effects of changes in gene expression mediated by lncRNAs on protein expression, we compared transcriptomic data presented here with proteomic data (Shen et al., 2015), and poor correlations were detected between lncRNA regulation and changes in protein expression level. Finally, some lncRNAs were identified as putative targets or precursors of miRNAs. These results provide a resource for investigating the functions of lncRNAs during the growth and development of *Brassica* plants and offer new insights into the roles of lncRNAs in allopolyploidization.

## 2 MATERIALS AND METHODS

### 2.1 Plant materials

**Figure 1:**
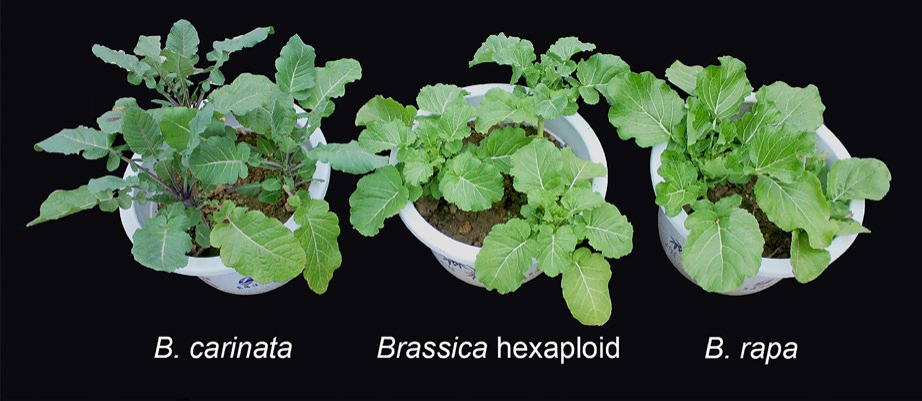
The organism photographs of three species

*Brassica rapa* cv. BaiguotianYC (AA, 2*n* = 20), *B. carinata* cv. CGN03955 (BBCC, 2*n* = 34) and the eighth generation synthesized *Brassica* hexaploid (BBCCAA, 2*n* = 54) were used in this study. Figure 1 shows the morphology of three species growing in flowpots. *B. carinata* (maternal parent) was crossed with *B. rapa* (paternal parent) followed with chromosome doubling, and the trigenomic *Brassica* allohexaploid was generated (Tian et al., 2010). The plant materials were grown and self‐pollinated in the field of Hubei Academy of Agricultural Science, China, under natural conditions. Three‐month‐old leaves from five individuals of both *Brassica* allohexaploid and its parents were collected, and then, leaves were frozen in liquid nitrogen for later use.

### 2.2 cDNA library construction, high‐throughput sequencing, and assembly of transcripts

Total RNA was extracted from mixed leaves of five individuals of *Brassica* hexaploid and its parents using Trizol reagent (Invitrogen) following the manufacturer's procedure, respectively. The experiments were repeated three times, which resulted in three independent pools for each species. After three independent pools’ quantity and purity standard for each species, three independent pools were mixed to a total pool to prepare for RNA‐seq. Ribosomal RNA of three species was depleted according to the introductions of the Epicentre Ribo‐Zero Gold Kit (Illumina, San Diego, USA). Following purification, RNA (rRNA depleted) was fragmented into small pieces using fragmentation buffer. The cleaved RNA fragments were reverse‐transcribed to create the three cDNA libraries for three species in accordance with the protocol for the mRNA‐Seq sample preparation kit (Illumina). The paired‐end sequencing (2 × 125 bp) was carried out by an Illumina Hiseq2000 sequencer at the LC Biotech, Hangzhou, China.

Preprocessing contained deletion of the adapter sequences, the reads in which the proportion of N (unidentified base) is greater than five percents, and low quality reads whose proportion of base (*Q* ≤ 10) is greater than twenty percents. After deleting the adapter reads and the low‐quality reads, sequencing reads were quality checked. The quality of raw data was assessed by FastQC (0.10.1), which assigned a quality score (*Q*) to each base in the read using a phred‐like algorithm (Ewing & Green, 1998). In addition, the analysis of the distribution of GC content using FastQC was applied to measure whether sequencing caused GC separation phenomenon so as not to affect the subsequent quantitative analysis. The clean reads from three cDNA libraries were mapped to the *B. rapa* genome v1.5 (http://brassicadb.org/brad/datasets/pub/BrassicaceaeGenome/Brassica_rapa/Bra_Chromosome_V1.5/) using the Tophat2 package (2.0.9) (mate_inner_dist = 50, mate_std_dev = 20, Tophat_threads = 16, min_intron_length = 45, max_intron_length = 5,000, min_segment_length = 45, max_segment_length = 5,000, mis = 2, library_type = fr‐firststrand), allowing a maximum of two base mismatches. (Feng, Li, Yu, Zhao, & Kong, 2015). Cufflinks software (2.1.1) was used to assemble the mapped reads to establish transcriptome (Trapnell et al., 2010). Cuffcompare program (2.1.1) was used to annotate the assembled transcripts on the basis of the annotation of *B. rapa* genome v1.5 sequence (Trapnell et al., 2010). The abundance of transcripts was estimated using Cuffdiff (2.1.1), and the unit of measurement is fragments per kilobase of exon per million mapped reads (FPKM). (Cufflinks: tophat_gtf_guide = G, cufflink_gtf_guide = g, cufflinks_Threads = 16, mask_file = N, label = CUFF, max_bundle_frags = 1,000,000, cuffmerge_Threads = 16, cuffdiff_Threads = 20, cuffdiff_gtf = g, frag_bias_correct = yes, multi_read_correct = yes, novel_transcript_class_code = i,j,u,o,x, novel_trans_length = 200).

### 2.3 Identification of mRNAs and lncRNAs

Known protein‐coding transcripts were confirmed using Cuffcompare program (2.1.1) according to the annotation of *B. rapa* genome v1.5 sequence, and the rest of unknown transcripts were used to screen out lncRNAs. To screen out lncRNAs from the rest of unknown transcripts, three steps needed to be carried out. Firstly, the remaining unknown transcripts that were longer than 200 nt were reserved. Secondly, transcripts with read coverage less than 3 were excluded. Finally, the coding capacity of transcripts was predicted by CPC (coding potential calculator, 0.9‐r2, http://cpc.cbi.pku.edu.cn/) (Kong et al., 2007) and CNCI (coding noncoding index, 2.0, https://github.com/www-bioinfo-org/CNCI) (Sun et al., 2013). If the value of CPC was less than −1 and the value of CNCI was less than 0, transcripts were considered to be noncoding. Meeting the three criteria, transcripts were deemed to be lncRNAs.

### 2.4 Analysis of differentially expressed mRNAs and lncRNAs

Differentially expressed mRNAs/lncRNAs were confirmed using Cuffdiff software (2.1.1) with |log_2_FC| ≥1 and *p*‐value <.05. The FPKM of *Brassica* hexaploid divided by the FPKM of *B. rapa*/*B. carinata* is equivalent to the fold change (FC), and *p* value is on behalf of statistical significance.

### 2.5 Confirmation of non‐additive lncRNAs

To study how hybridization and allopolyploidization alter lncRNAs in nonadditive expression profiles, the expression values of lncRNAs in *Brassica* hexaploid were compared with the average of lncRNA expression values in parents. If the value of an lncRNA in *Brassica* hexaploid was at least a twofold change and *p *<* *.05 relative to the mid‐parent value (MPV), the lncRNA was regarded as nonadditive expression, and the rest of lncRNAs were considered as additive expression.

### 2.6 Prediction of the target genes of differentially expressed lncRNAs and construction of lncRNAs‐mRNAs co‐expression network

To determine cis–regulation relationship pairs, we regarded differentially expressed lncRNA and differentially expressed mRNA as a pair if they were co‐expressed and less than 100 kb apart, in accordance with the reported method (Liao et al., 2011). The target genes of differentially expressed lncRNAs via trans‐acting were identified by sequence complementarity (Chen, Fu et al., 2015; Chen, Quan, & Zhang, 2015). A target gene sequence complementary to the lncRNA was selected by BLAST with *E*‐value = 1e‐5 and identity = 95%; then, the targets with *E*‐value = −30 was singled out using RNAplex program (Tafer & Hofacker, 2008). To give a visual representation of the interactions between lncRNAs and target protein‐coding RNAs, the interactive networks were built using Cytoscape software (3.1.1) (Saito et al., 2012).

### 2.7 Function classification of the target genes of differentially expressed lncRNAs

WEGO (http://wego.genomics.org.cn/cgi-bin/wego/index.pl) was used to functionally classify the target genes and graphically represent the target gene functions (Mazumdar & Chattopadhyay, 2016). It is a useful tool for plotting GO annotation results and shows the categorization into three main ontologies: “cellular component,” “molecular function’,” and “biological process.”

### 2.8 Prediction of miRNA targets and precursors

We used psRobot web to examine whether lncRNAs were targets of known miRNAs. The psRobot is an online free miRNA target prediction tool (http://omicslab.genetics.ac.cn/psRobot/target_prediction_1.php) (Wu, Ma, Chen, Wang, & Wang, 2012; Wu, Liu et al., 2012; Wu, Okada et al., 2012), with appropriate parameters (maximal number of permitted gaps = 1, five prime boundary of essential sequence = 2, penalty score threshold = 2.5, position after which with gaps permitted = 17, three prime boundary of essential sequence = 17). We also determined if lncRNAs served as miRNA precursors. The secondary structure of lncRNA transcripts was predicted by the Vienna RNA package RNAfold program, which checked out the stability of hairpin structures (Chen, Fu et al., 2015; Chen, Quan, & Zhang, 2015).

### 2.9 Validation of lncRNA expression by qRT‐PCR

Total RNA was isolated from young leaves of *B. rapa*,* B. carinata,* and *Brassica* hexaploid, respectively. RNase‐free DNase I (Fermentas, Canada) was used to eliminate DNA contamination. About 2 μg RNA was reverse‐transcribed using random hexamer primers (Bioligo Biotech, Shanghai, China) into cDNA. Ten lncRNAs were randomly chosen to be validated. The gene‐specific primers were designed using the Primer5 software, and the sequences are listed in Table S1. QRT‐PCR was performed using the ABI Step One Plus Real‐Time PCR System with the SYBR kit (Applied Biosystems, USA). *Actin2/7* gene was chosen as the internal control to standardize the results, and the comparative Ct method (2^−ΔΔCT^) was used to calculate the relative expression level (Zhang, Peng et al., 2014). All reactions were performed in two technical replicates and two biological replicates. The reactions were carried out with the following conditions: 95°C for 5 min and 42 cycles of 95°C for 30 s, 60°C for 30 s and 72°C for 20 s, 72°C for 10 min. After each run, a melting curve was produced to ensure the product specificity and to check whether primer dimers appear. The results were averages of four independent tests (two technical replicates and two biological replicates), and all the values of qRT‐PCR experiments were expressed as the mean ± *SD* of four replicates (Zhang, Peng et al., 2014).

## 3 RESULTS

### 3.1 The analysis of high‐throughput sequencing data

Initially, high‐through sequencing generated 103,779,510, 96,915,650, and 88,869,438 raw reads in *B. rapa*,* B. carinata*, and *Brassica* hexaploid, respectively. The high‐throughput sequencing error rate rose with the increase of the read length, and a quality assessment of the raw data is necessary. The results showed that the quality of the raw data met the requirements (Figure S1), and the GC separation phenomenon did not exist in the raw data (Figure S2). The analysis reflected that our data were credible. After trimming, 103,087,524 (*B. rapa*), 96,083,300 (*B. carinata*), and 88,088,334 (*Brassica* hexaploid) clean reads were gained, indicating that more than 99% of the raw data were clean reads (Table 1).

**Table 1:** Summary of the RNA‐seq reads for three cDNA libraries

Sample	Raw data (reads)		Valid data (reads)		Valid ratios	*Q*20%	*Q*30%
A	103779510	12.97G	103087524	12.89G	99.33	99.49	88.82
ABC	88869438	11.11G	88088334	11.01G	99.12	99.68	88.64
BC	96915650	12.11G	96083300	12.01G	99.14	99.65	88.68

A, *Brassica rapa*; BC, *Brassica carinata*; ABC, *Brassica* hexaploid. Q20% represents the proportion of the data that quality values are greater than Q20 in raw data. Q30% represents the proportion of the data that quality values are greater than Q30 in raw data.

### 3.2 Differentially expressed mRNAs between *Brassica* hexaploid and its parents

**Figure 2:**
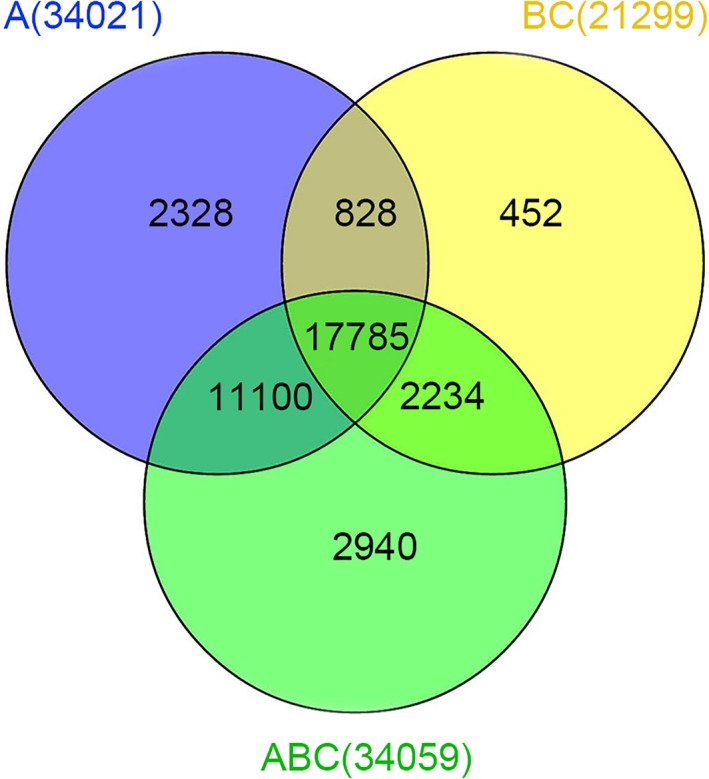
Venn diagram showing known mRNAs expressed in *Brassica* hexaploid and its parents. A, *Brassica rapa*; BC,* Brassica carinata*; ABC,* Brassica* hexaploid

**Figure 3:**
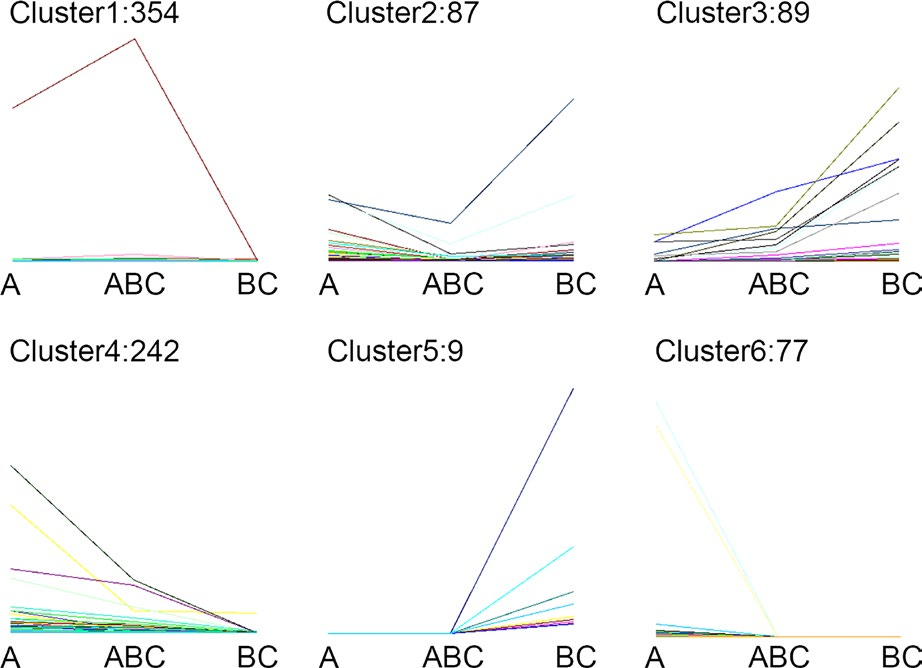
Six clusters of differentially expressed mRNAs expression pattern. A, *Brassica rapa*; BC,* Brassica carinata*; ABC,* Brassica* hexaploid. The six clusters of differentially expressed mRNAs expression pattern were grouped by up‐ or down‐regulation in *Brassica* hexaploid compared to its parents

We confirmed 32,041, 21,299, and 34,059 mRNAs in *B. rapa*,* B. carinata*, and *Brassica* hexaploid, respectively (Figure 2). Among all mRNAs, only 17,785 were expressed across the three *Brassica* species. In addition, 28,885 were expressed in *Brassica* hexaploid and *B. rapa*, and 20,019 were expressed in *Brassica* hexaploid and *B. carinata*, while 18,613 were expressed in *B. carinata* and *B. rapa*. The number of specifically expressed mRNAs exhibited the highest number in *Brassica* hexaploid, which was lower in *B. rapa* and the lowest in *B. carinata*. Comparing *Brassica* hexaploid with *B. rapa*, 461 mRNAs were differentially expressed with 250 mRNAs up‐regulated and 211 mRNAs down‐regulated. Relative to *B. carinata*, 424 were differentially expressed in the *Brassica* hexaploid, containing 375 up‐regulated mRNAs and 49 down‐regulated mRNAs. The expression patterns of 858 differentially expressed mRNAs could be divided into six clusters (Figure 3). Cluster 1 was made of mRNAs with the highest expression in the *Brassica* hexaploid. Conversely, Cluster 2 was composed of mRNAs with the lowest expression in *Brassica* hexaploid. The mRNAs in Cluster 3 were the highest in *B. carinata*, lower in *Brassica* hexaploid, and the lowest in *B. rapa*, while mRNAs in Cluster 4 had the highest expression in *B. rapa*, the lower expression in *Brassica* hexaploid, and the lowest expression in *B. carinata*. Cluster 5 contained mRNAs that were only expressed in *B. carinata*, and Cluster 6 consisted of mRNAs only expressed in *B. rapa*. Cluster 1 contained more differentially expressed mRNAs, which showed that these mRNAs were inclined to have higher expression in *Brassica* hexaploid. Some of the differentially expressed mRNAs could become the targets of differentially expressed lncRNAs. Next, the lncRNA‐mRNA relationship pairs were found in order to construct interactive networks.

### 3.3 Genome‐wide identification and characterization of lncRNAs

**Figure 4:**
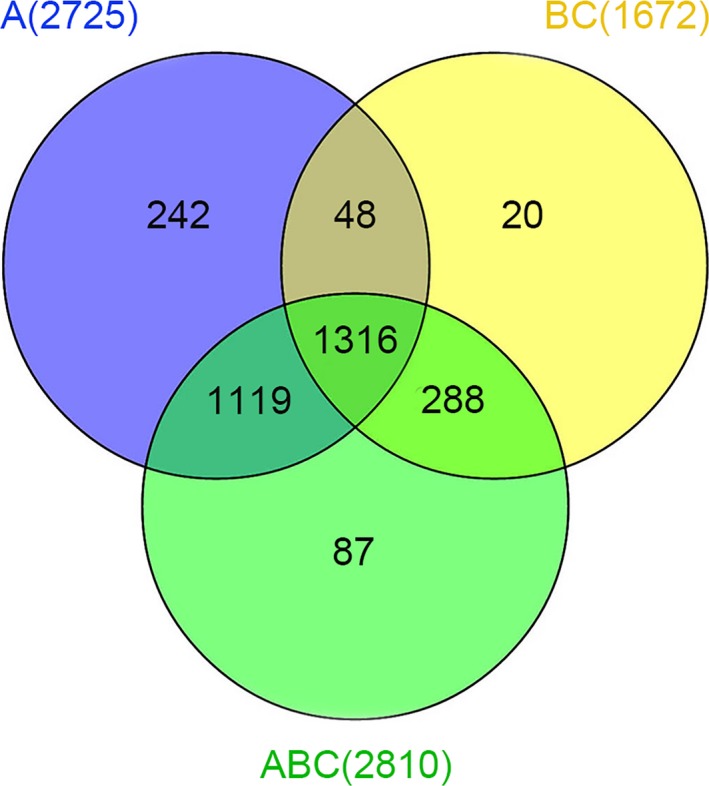
Venn diagram showing lncRNAs expressed in *Brassica* hexaploid and its parents. A, *Brassica rapa*; BC,* Brassica carinata*; ABC,* Brassica* hexaploid

**Figure 5:**
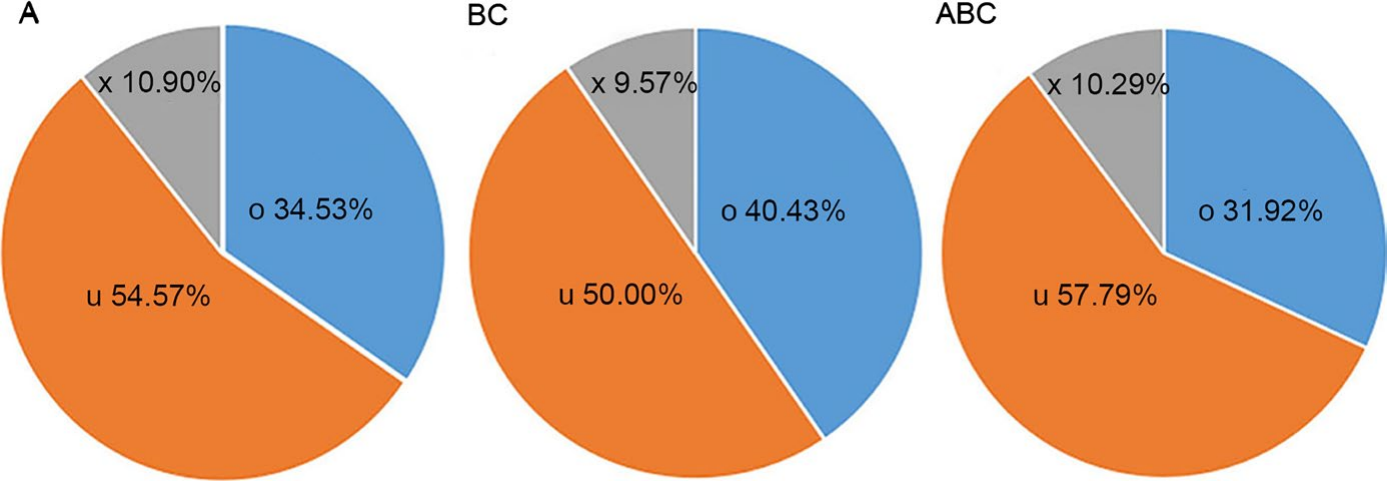
Different types of lncRNAs in *Brassica* hexaploid and its parents. A, *Brassica rapa*; BC,* Brassica carinata*; ABC,* Brassica* hexaploid. The “x” contained antisense lncRNAs, the “o” contained sense lncRNAs, the “u” contained intergenic lncRNAs

**Figure 6:**
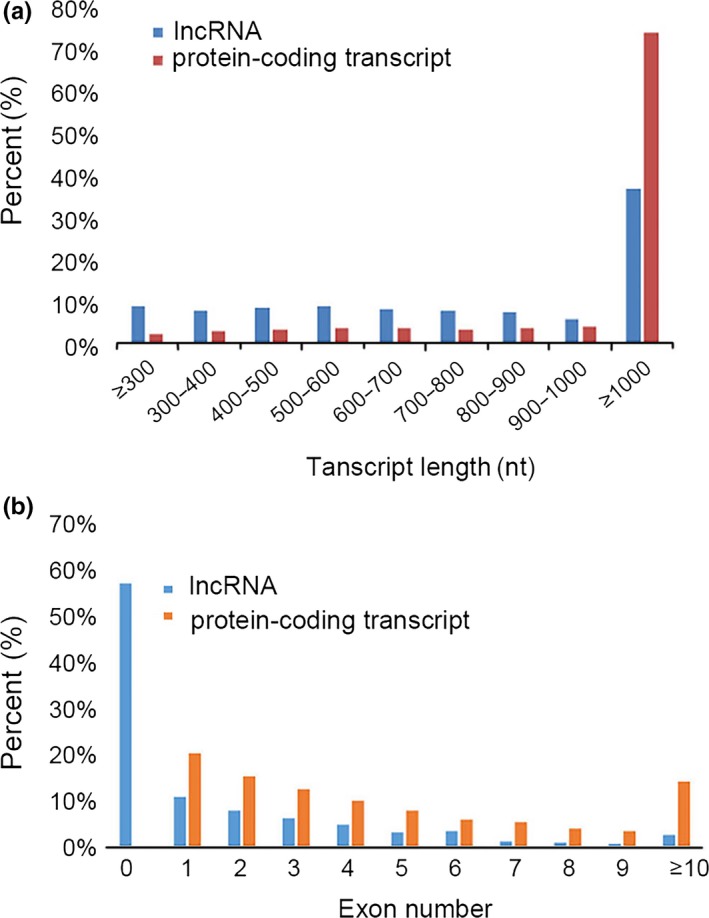
The distribution of length (a) and numbers of exons (b) of lncRNAs in comparison with protein‐coding transcripts

In accordance with the transcript length, read coverage and coding capacity, 3,120 lncRNAs were identified in total, and 1,316 of which were expressed in all three species. LncRNA coordinates were compared with transposon element coordinates to identify genomic relationship, and 1,217 of 3,120 lncRNAs were overlapped with transposon elements based on *B. rapa*_TEs_v1.5 annotation (Table S2). All the lncRNA sequences were shown in Data S1. Moreover, 1,364 were expressed in *B. rapa* and *B. carinata*, and 1,604 were expressed in *B. carinata* and *Brassica* hexaploid, while 2,435 were expressed in *B. rapa* and *Brassica* hexaploid. The number of expressed lncRNAs in *B. rapa* and *Brassica* hexaploid is more than those in *B. carinata* and *Brassica* hexaploid. The number of specifically expressed lncRNAs was the highest in *B. rapa*, lower in *Brassica* hexaploid, and the lowest in *B. carinata*. Compared with its parents, *Brassica* hexaploid possessed more lncRNAs (Figure 4). According to the location relative to the nearby protein‐coding genes, they were classified into three types: intergenic, sense, and antisense (denoted as u, o and x). The “u” contained the intergenic lncRNAs. The “o” contained the lncRNAs that have generic exonic overlap with a known transcript. The “x” contained the lncRNAs that have exonic overlap with a known transcript, but on the opposite strand. Most lncRNAs were located in intergenic regions (Figure 5). It is reported that lncRNAs are shorter and possess fewer exons than protein‐coding transcripts. We analyzed the exon number and the distribution of the length between lncRNAs and protein‐coding transcripts. Figure 6a shows that 63% of lncRNAs ranged in length from 200 to 1,000 nucleotides, and only 37% were longer than 1,000 nucleotides. In contrast, 74% of the protein‐coding transcripts were longer than 1,000 nucleotides. In addition, 68% of the lncRNAs consisted of zero or one exon, while >80% of the protein‐coding transcripts had more than one exon (Figure 6b). Hence, most of the lncRNAs were shorter and had fewer exons relative to protein‐coding transcripts.

### 3.4 Differentially expressed lncRNAs and their expression patterns

**Figure 7:**
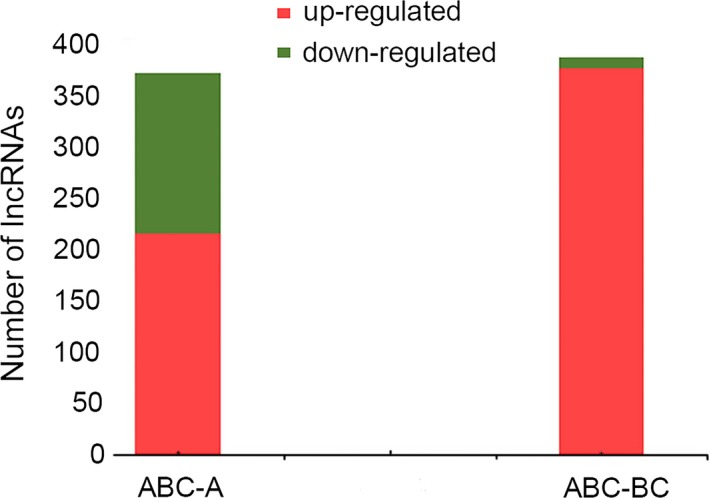
Differentially expressed lncRNAs between *Brassica* hexaploid and its parents. A, *Brassica rapa*; BC,* Brassica carinata*; ABC,* Brassica* hexaploid

**Figure 8:**
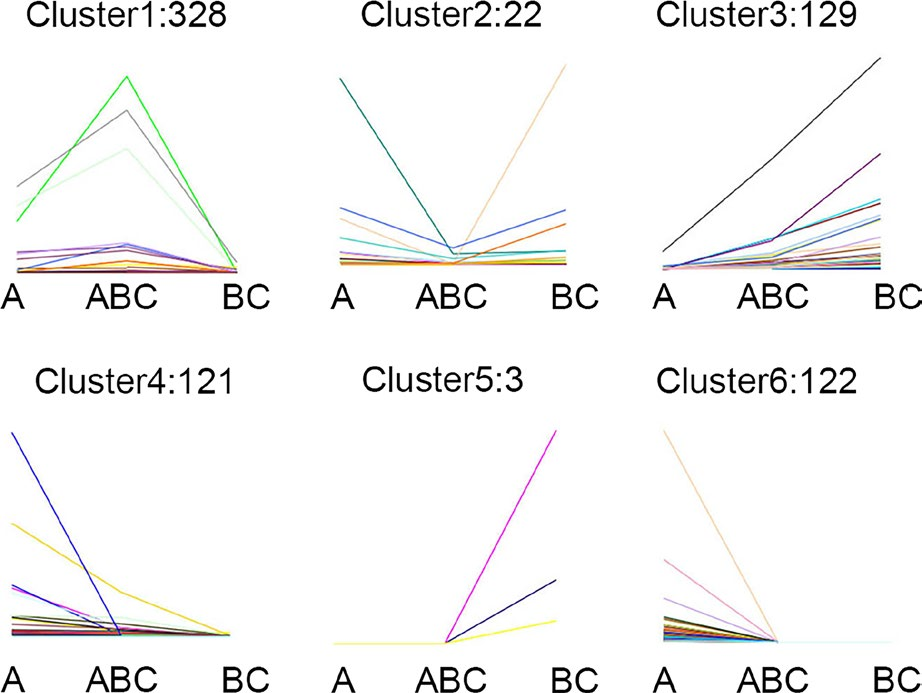
Six clusters of lncRNAs expression pattern. A, *Brassica rapa*; BC,* Brassica carinata*; ABC,* Brassica* hexaploid. The six clusters of lncRNAs expression pattern were grouped by up‐ or down‐regulation in *Brassica* hexaploid compared to its parents

We detected 727 differentially expressed lncRNAs between *Brassica* hexaploid and its parents. It is observed that 367 lncRNAs were differentially expressed in *Brassica* hexaploid compared with *B. rapa* (Table S3), with 214 up‐regulated and 153 down‐regulated. Compared with *B. carinata*, 382 lncRNAs were differentially expressed in *Brassica* hexaploid (Table S4), including 372 up‐regulated and 10 down‐regulated (Figure 7). The two comparisons indicated that the number of differentially expressed lncRNAs was similar, but up‐regulated lncRNAs accounted for ninety‐seven percents between *Brassica* hexaploid and *B. carinata*, indicating that the vast majority of lncRNAs in *Brassica* hexaploid have higher expression levels than its maternal parent. In addition, 571 lncRNAs were differentially expressed between the progenitors, of which 163 were up‐regulated and 408 were down‐regulated in *B. carinata*. The total value of |log_2_FC| was 6507.95 in differentially expressed lncRNAs between *Brassica* hexaploid and *B. rapa*, while the total value was 7101.09 between *Brassica* hexaploid and *B. carinata*. These results demonstrated that there were more differentially expressed lncRNAs with larger expression differences between *Brassica* hexaploid and *B. carinata*, which indicated paternal parent‐biased expression. The expression patterns of the differentially expressed lncRNAs were categorized into six types (Figure 8). Cluster 1 was composed of lncRNAs with the highest expression in *Brassica* hexaploid. Conversely, Cluster 2 was made up of lncRNAs with the lowest expression in the *Brassica* hexaploid. LncRNAs in Cluster 3 were the highest in *B. carinata*, lower in *Brassica* hexaploid, and the lowest in *B. rapa*. LncRNAs in Cluster 4 were the highest in *B. rapa*, lower in *Brassica* hexaploid, and the lowest in *B. carinata*. Cluster 5 consisted of lncRNAs only expressed in *B. carinata*, and Cluster 6 was comprised of lncRNAs only expressed in *B. rapa*. These results embodied the diversity of the lncRNA expression level between *Brassica* hexaploid and its parent.

**Figure 9:**
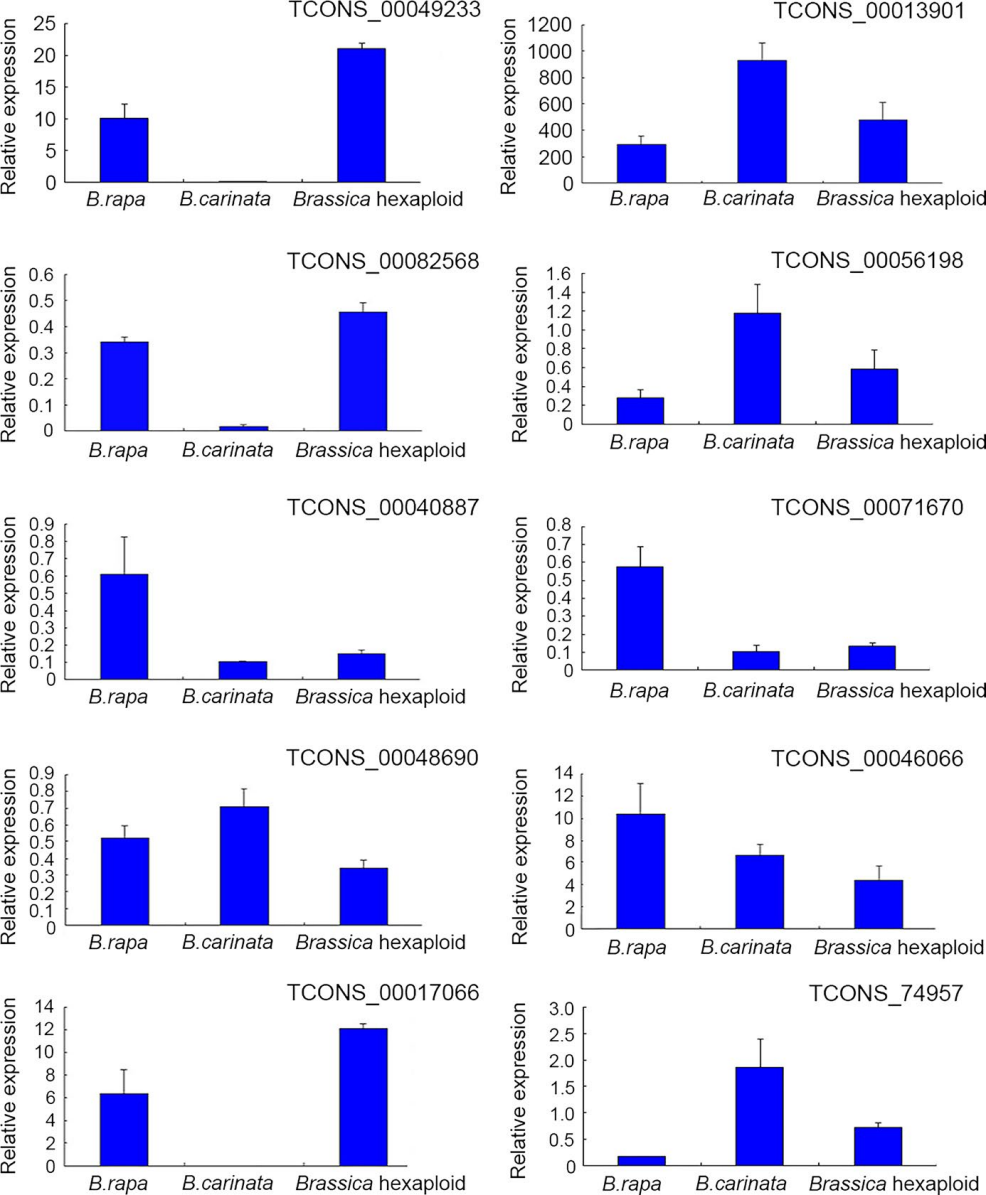
The relative expression levels of selected ten lncRNAs are validated by quantitative real‐time RT‐PCR. Error bars display the standard deviation of four replicates

To verify the expression patterns identified by RNA‐seq, 10 lncRNAs were selected to validate their expression by qRT‐PCR, and the expression levels of 10 lncRNAs identified by RNA‐seq were shown in Table S5. Figure 9 shows the relative express levels of 10 lncRNAs in three samples normalized to the expression level of *Actin2/7* gene. These results were consistent with those identified patterns from the high‐throughput sequencing, indicating that the sequencing data were reliable and represent real transcripts.

### 3.5 Nonadditive lncRNAs expression pattern and progenitor‐biased repression

After screening, 379 lncRNAs were nonadditively expressed in *Brassica* hexaploid, and 198 showed down‐regulation, indicating that inhibition could be a pattern of non‐additive lncRNA regulation in allohexaploids (Table S6). Repressed nonadditive lncRNAs were divided into three categories based on their expression patterns in two parents. First, 26.47% of the lncRNAs that showed higher expression levels in the paternal parent than that in maternal parent were repressed in *Brassica* hexaploid. Second, 44.17% of the lncRNAs that showed higher expression levels in the maternal parent than that in the paternal parent were repressed in *Brassica* hexaploid. Third, 0.73% of the lncRNAs that were equally expressed in the paternal and maternal parent were down‐regulated in *Brassica* hexaploid. In the second category, biased repression existed in the maternal parent lncRNAs, while there was no bias in the other category. The result suggested that the lncRNAs that were more highly expressed in *B. carinata* than in *B. rapa* were likely to be repressed in the *Brassica* hexaploid. The reason for paternal parent‐biased expression maybe that a subset of maternal parent genomic loci suffered from homeologous genome‐specific RNA‐mediated DNA methylation, and numerous maternal parent genes were subject to transcriptional repression (Chen, Ha, Lackey, Wang, & Chen, 2008).

### 3.6 LncRNA‐mRNA interaction network analysis

**Figure 10:**
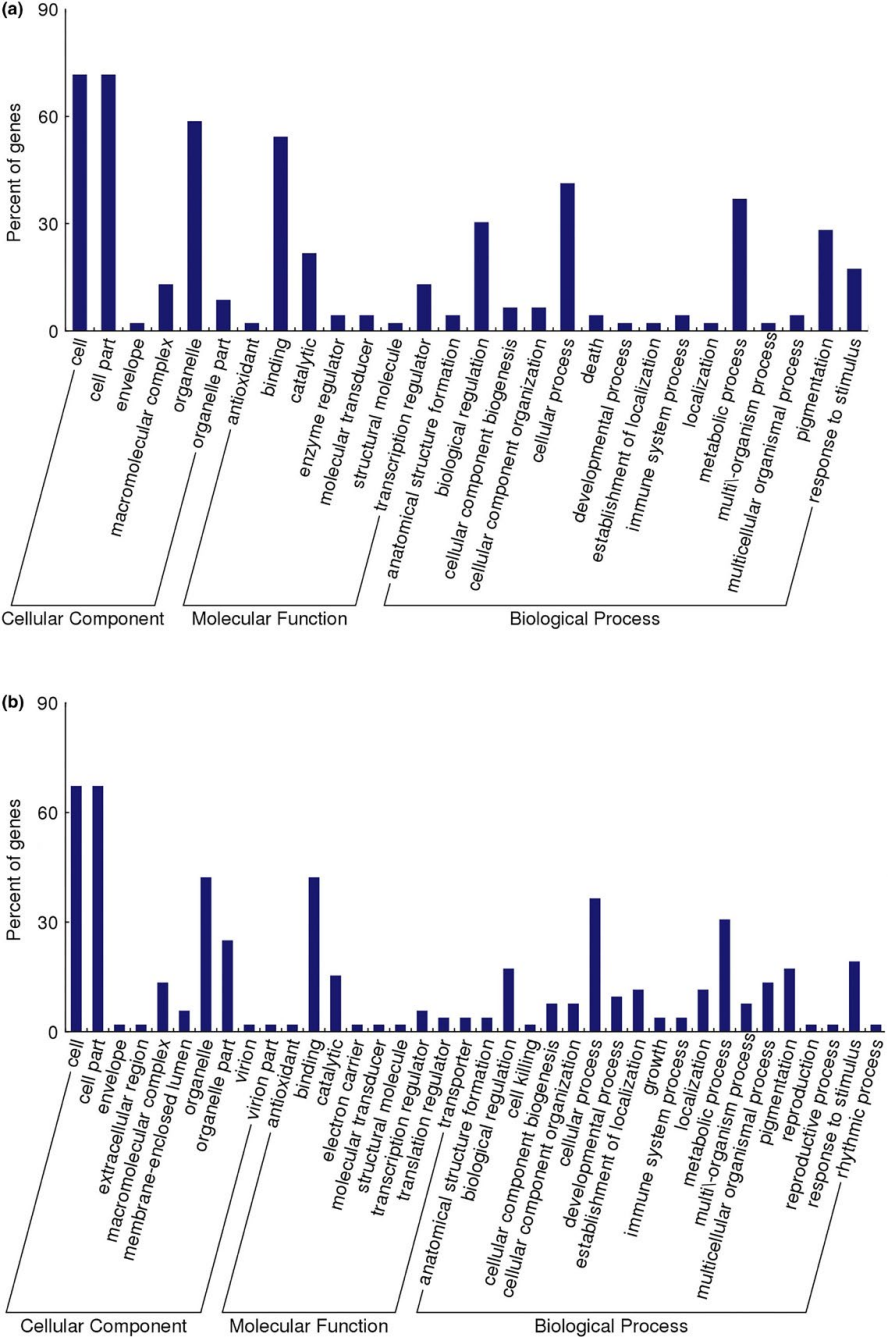
(a) Gene ontology (GO) enrichment analysis on target genes of differentially expressed lncRNAs between *Brassica* hexaploid and *Brassica rapa*. (b) GO enrichment analysis on target genes of differentially expressed lncRNAs between *Brassica* hexaploid and *Brassica carinata*. The *y*‐axis represents the percent of target genes, and the *x*‐axis represents the GO functional groups

**Figure 11:**
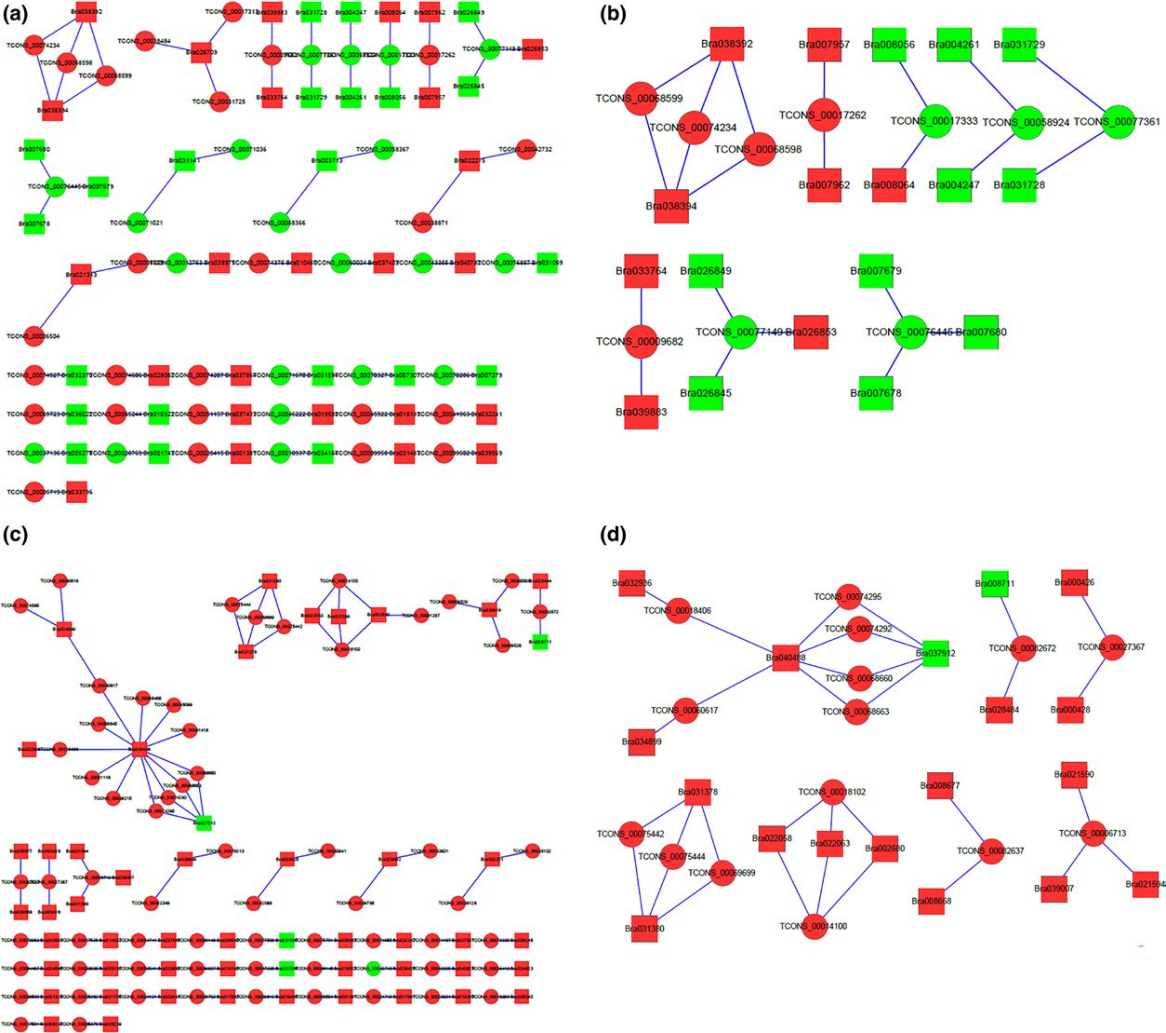
The interaction network among lncRNAs and target protein‐coding genes. The circle and rectangle nodes represent lncRNAs and protein‐coding genes, respectively. The up‐regulated and down‐regulated nodes are separately colored in red and green. Edges show regulatory interactions among nodes

After allopolyploidization, lncRNAs showed changes in expression levels, along with significant alterations in the expression of relevant protein‐coding genes. There were 367 differentially expressed lncRNAs and 461 differentially expressed mRNAs between *Brassica* hexaploid and *B. rapa*. Through cis‐acting and trans‐acting regulation prediction of the differentially expressed lncRNAs, 47 protein‐coding genes were regarded as potential targets for the 45 lncRNAs, and a total of 57 matched lncRNA‐mRNA pairs were found (Table S7). Cis–regulation relationships contained 49 lncRNA‐mRNA pairs, including 38 lncRNAs and 41 mRNAs. Between the lncRNAs and their target genes, four kinds of positional relationships existed, in which lncRNAs were located in the up‐stream or down‐stream of the target genes, and in the loci where the target genes were located. The last one was that lncRNA was overlapped with the promoter of the target gene and had only one lncRNA. Its expression tendency was consistent with the target gene, and the transcription of lncRNA may facilitate the expression of the target gene. Trans‐regulation relationships contained eight matched lncRNA‐mRNA pairs, including eight lncRNAs and six mRNAs. Gene Ontology (GO) terms of genes targeted by differentially expressed lncRNAs were analyzed to understand the potential biological roles of lncRNAs. These target genes were categorized in accordance with the secondary classification of the GO terms, and they were divided into 15 GO terms in the biological process category, 7 GO terms in the molecular function category, and 6 GO terms in the cellular component category (Figure 10a). The cell part and organelle were predominant in the cellular component category. For the molecular function category, binding was dominant. Cellular process, metabolic process, biological regulation, and pigmentation were prevailing in the biological process category. The enrichment number of the target genes in each functional category could be found in Table S8. RNAs regulated and bound by the same RNA‐binding proteins (RBP) generally contained conserved primary sequence motifs. LncRNAs were divided into different groups in accordance with the functional annotations of target genes, picking out some groups with more lncRNAs. LncRNAs in the same group could be regulated and bound by the same RBP, so conservative motifs might exist among them. Each group of lncRNAs was inspected to find the conserved sequence motifs using MEME web (sequence motif width constrained to 4–12 nucleotides, which is the common RBP binding size, the significance threshold set to an *E*‐value of 0.05, allowing 0 or 1 motif per sequence), but no motifs were found. The reason that no motifs were found may be that the lncRNAs in each group have no sequence similarity and interact with different RBP. The modification levels of paternal genome are different from maternal genome, and its DNA fragments tend to be lost during allopolyploid formation (Gaeta et al., 2007). The 45 lncRNAs with target genes were composed of 25 up‐regulated lncRNAs and 20 down‐regulated lncRNAs. The up‐regulated lncRNAs increased the expression quantity of 19 target genes and decreased the expression quantity of three genes. The down‐regulated lncRNAs made six target genes up‐regulate and 19 genes down‐regulate. From this, we can see that most of the target genes were subjected to positive control. To visually display the relationship between lncRNAs and protein‐coding genes, interactive networks were constructed using Cytoscape software (3.1.1). The interactive network was composed of 92 nodes and 57 edges (Figure 11a). One lncRNA could regulate multiple protein‐coding genes, while one protein‐coding gene could be controlled by multiple lncRNAs as shown in Figure 11a. AS seen from the figure, the lncRNA‐mRNA pairs with the same expression trend were more than those with the opposite expression trend. Nine matched pairs reflected the opposite expression trend, implying that relevant lncRNAs inhibited the expression of target genes. LncRNAs with two or more nodes were regarded as the key objects, constructing the local network (Figure 11b). There were eight target genes (Bra021313, Bra022275, Bra018193, Bra028062, Bra037403, Bra010460, Bra008064, and Bra019580) that were only expressed in *Brassica* hexaploid, and they could be activated by relevant lncRNAs (TCONS_00006504, TCONS_00006505, TCONS_00038871, TCONS_00042732, TCONS_00045922, TCONS_00074606, TCONS_00060024, TCONS_00074375, TCONS_00017333, TCONS_00046222). Two target genes (Bra031594 and Bra007679) in *Brassica* hexaploid had no expression, and they may be repressed by lncRNAs (TCONS_00071670 and TCONS_00076445).

**Figure 12:**
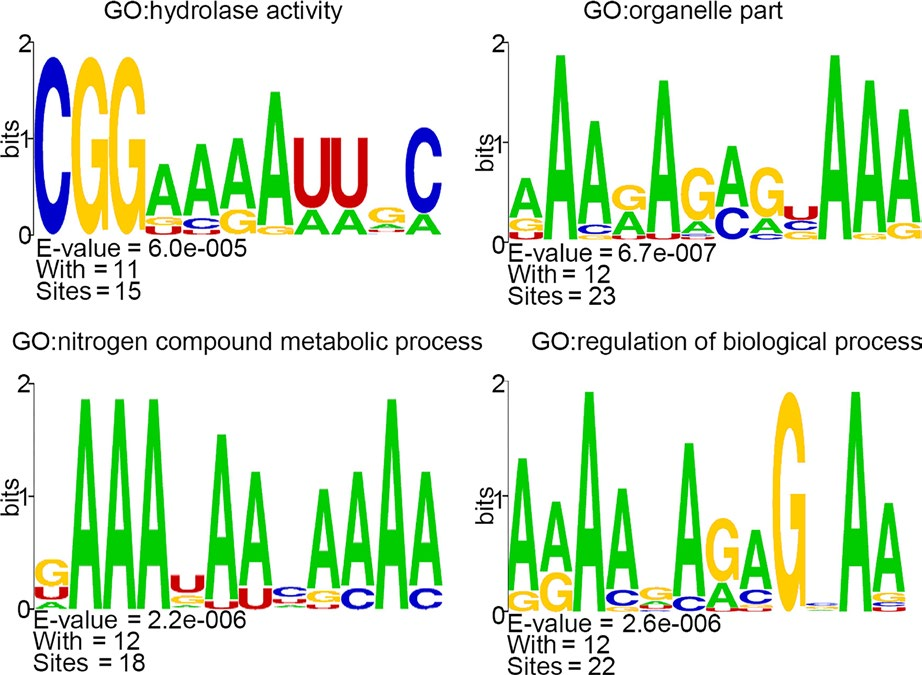
Four conserved motifs of lncRNA associated with four gene ontology terms. The *y*‐axis of the sequence logo represents information contents in bits, the *x*‐axis of the sequence logo represents the width of motif, and *E*‐values for sequence motifs are calculated by comparison with shuffled sequences

**Figure 13:**
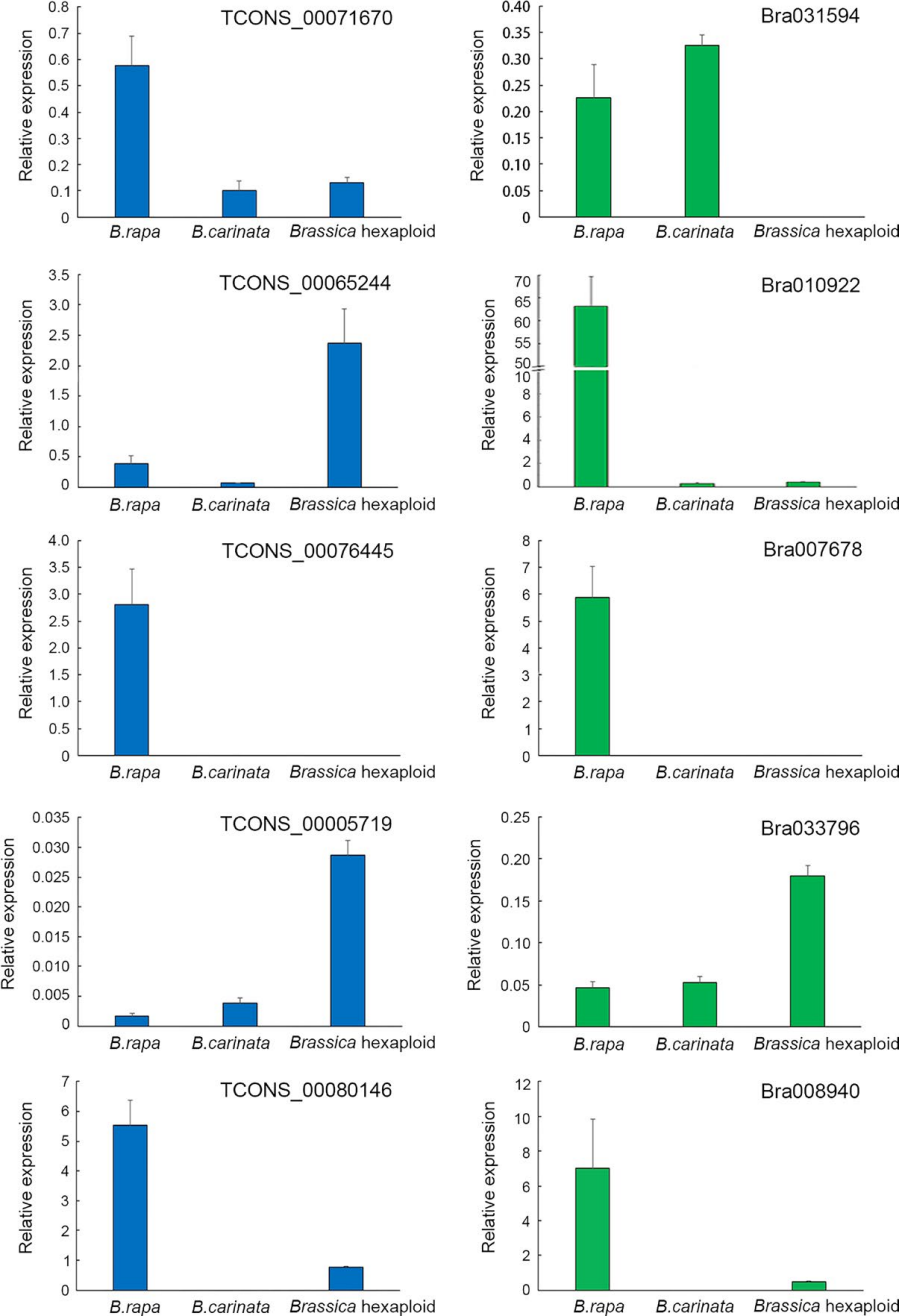
The relative expression levels of five lncRNA‐mRNA pairs are validated by qRT‐PCR. Error bars display the standard deviation of four replicates

Compared with *B. carinata*, 382 differentially expressed lncRNAs and 424 differentially expressed mRNAs were detected in *Brassica* hexaploid. We found 52 potential target genes for 64 differentially expressed lncRNAs, and a total of 82 matched lncRNA‐mRNA pairs were detected (Table S7). Cis–regulation relationships embraced 58 lncRNA‐mRNA pairs, including 50 lncRNAs and 44 mRNAs. There were three kinds of positional relationships between these lncRNAs and target genes. The lncRNAs were located in the up‐stream or down‐stream of the target genes, and in the loci where the target genes were located. Trans‐regulation relationships contained 24 lncRNA‐mRNA pairs, including 24 lncRNAs and 10 mRNAs. Target genes were divided into 19 GO terms in the biological process category, 9 GO terms in the molecular function category, and 10 GO terms in the cellular component category. For the cellular component category, the cell part and organelle were significant terms. Binding was the dominant term in the molecular function category. Regarding the biological process category, cellular process and metabolic process were ascendant (Figure 10b). The enrichment number of the target genes in each functional category could be found in Table S9. We divided lncRNAs into eighteen different groups according to the functional annotations of the target genes. The conservative motifs from eighteen different groups were found (Figure S3). For example, a CGG motif was found to respond to the hydrolase activity (Figure 12, upper left). These lncRNAs with target genes were made up of 63 up‐regulated lncRNAs and 1 down‐regulated lncRNA, and 63 lncRNAs contributed to the expression of 47 target genes and limited the expression of four genes, while the down‐regulated one increased the expression of the target gene. The expression level of the majority of the target genes took on an increasing trend. The interactive network between the lncRNAs and target genes consisted of 116 nodes and 82 edges (Figure 11c). The overwhelming majority of the objects were up‐regulated, and only nine objects’ expression levels were down‐regulated. The relationship pairs with the same expression trend accounted for 93.1%, showing that most of the lncRNAs promoted target gene expression. LncRNAs with two or more nodes were regarded as the key objects constructing the local network (Figure 11d). The expression levels of five lncRNA‐mRNA pairs were verified to show relevance by qRT‐PCR (Figure 13).

To investigate the correlation between lncRNA regulation and changes in protein expression level, the transcriptomic data in this study were compared with the proteomic data from the previous study (Shen et al., 2015). The conjoint analysis of 425 differentially expressed proteins and 858 differentially expressed mRNAs between *Brassica* hexaploid and its parents indicated that only two mRNAs (Bra018943 and Bra011794) showed significant protein level differences. Poor correlations were found between mRNA and protein expression. The result was in alignment with the research studied by Shen et al. (2015). Ninety‐nine target genes regulated by lncRNAs had no significant protein level differences between *Brassica* hexaploid and its parents, which maybe because the changes in gene expression mediated by lncRNAs merely brought about fine‐tuning of protein expression. The low correlation between mRNA and protein expression could contribute to the poor correlation between lncRNA regulation and changes in protein expression level.

### 3.7 LncRNA as targets of miRNAs and as miRNAs precursors

**Figure 14:**
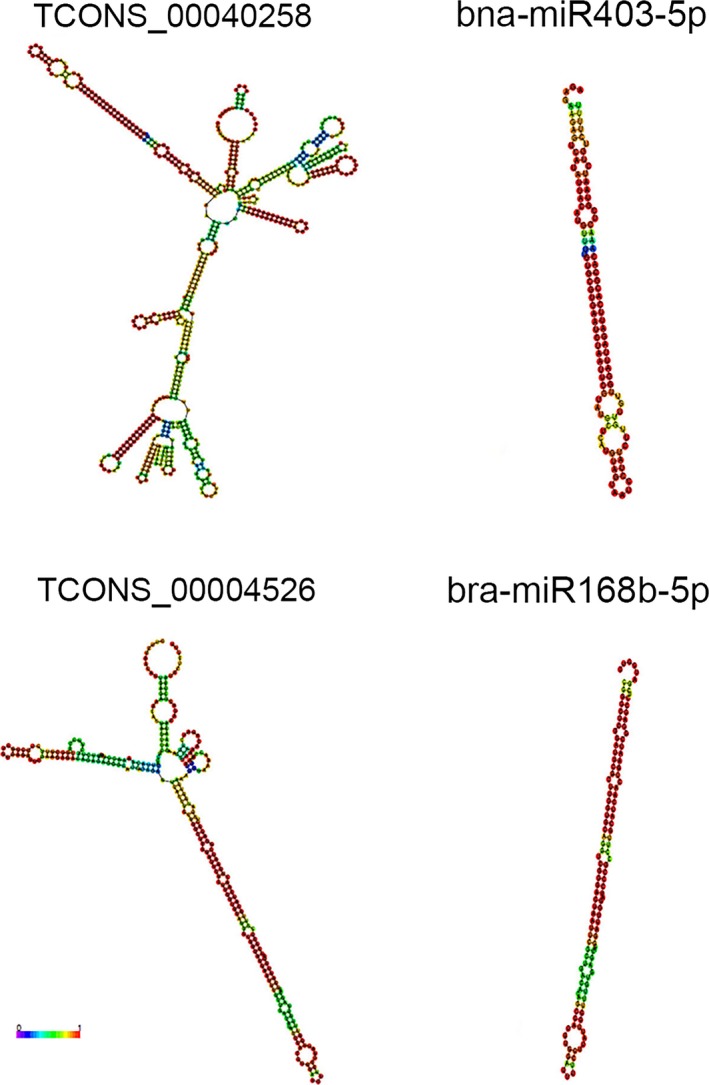
The predicted secondary structure of two lncRNA transcripts and two miRNA sequences. The color bars indicate base‐pair probabilities. The left was lncRNA, and the right was corresponding miRNA

LncRNAs could act as targets or precursors of miRNAs to regulate gene expression (Chen, Fu et al., 2015; Chen, Quan, & Zhang, 2015). Target mimicry is a vital function for lncRNAs to regulate growth and metabolic progress in plants (Zhu & Wang, 2012). By binding miRNAs, this type of lncRNAs could sequester the miRNAs’ effects on their target genes. To investigate the indirect regulatory functions of lncRNAs, we examined their sequences to determine if they could be targets or precursors of known miRNAs, and 61 lncRNAs were found as potential targets of 100 miRNAs, showing that one lncRNA may be targeted by more miRNAs, such as TCONS_00017006 (Table S10). Six lncRNAs were targeted by bra‐miR5721, indicating that one miRNA could target more than one lncRNA. Seven of the sixty‐one lncRNAs were differentially expressed lncRNAs between *Brassica* allohexaploid and its parents. We checked the sequences of lncRNAs to screen out miRNA precursors and found that only 15 of 3,120 lncRNAs (0.48%) harbored complete precursors for 15 miRNAs (Table S11), which was consistent with the earlier study that only 0.2% of lncRNAs serve as miRNA precursors to regulate biological processes (Chen, Fu et al., 2015; Chen, Quan, & Zhang, 2015). Prediction of the secondary structure for the 15 transcripts using the Vienna RNA package RNAfold program showed that these miRNA precursors had stable hairpin structures (Figure S4), two of which are shown in Figure 14. These lncRNAs could be involved in the miRNA‐mRNA network action.

## 4 DISCUSSION

In recent years, increasing studies have revealed that lncRNAs exert various crucial roles in multiple biological processes in plants, but information with respect to the characteristics, expression patterns, and potential function of lncRNAs in allopolyploids still remains largely unknown. In this study, RNA‐seq was used for lncRNAs analysis, addressing the following three aspects: (1) the characteristics of lncRNAs (number, length, exon); (2) the changes in the expression patterns of lncRNAs between *Brassica* hexaploid and its parents; and (3) the effects of lncRNA on genes expression in *Brassica* allopolyploid. Our results provide new insight into the regulation function of lncRNAs in *Brassica*, providing abundant information for further studying the molecular mechanisms for *Brassica* allopolyploid adaption and evolution.

### 4.1 Long‐coding RNAs play an important role in gene expression regulation during the plant evolution process

In *Brassica* allohexaploid, nonadditive miRNA regulation may enhance the potential for adaption (Shen et al., 2014). Similar to small RNAs, our data indicated that lncRNAs might also play an important role during the plant evolution process. The regulation roles of lncRNAs were primarily described from three aspects: control on the expression of target genes by cis/trans‐action, the interaction with miRNAs and precursors of miRNAs. We paid close attention to the regulation of differentially expressed lncRNAs on target genes by cis/trans‐action. Compared with its parents, the expression levels of target genes in *Brassica* hexaploid are not only up‐regulated but also down‐regulated. In trans‐action, lncRNAs control genes by sequence complementarity, and expression levels of all the target genes are up‐regulated. The variation in the expression of target genes has been recounted, but detailed regulatory mechanisms are unknown.

LncRNAs could also be complementary to miRNAs, as noncleavable targets through forming mismatch loops, and sequestrating the regulation roles of miRNAs on their target genes. In our data, 61 lncRNAs targeted by 100 miRNAs were screened out, along with 15 lncRNAs corresponding to 15 miRNA precursors with stable hairpin structures. To sum up, these lncRNAs work not just through a single method but via various pathways. The prediction of target genes of lncRNAs offers a helpful way to know which processes lncRNAs take part in, thereby further speculating their potential roles. Compared with *B. rapa*, target genes of differentially expressed lncRNAs between *Brassica* hexaploid and *B. carinata* were classified into more functional groups, such as the extracellular region, translation regulator, and rhythmic process. Transcription factors exist in many different signal transduction pathways, and specially bind to the cis‐acting elements in promoter regions, controlling the expression of downstream genes. Eleven transcription factor genes (Table S12) were found to be target genes, which were classified into eight families that were involved in growth and development, response to environmental stress, cell differentiation, and disease resistance. Some target genes were also involved in multiple biological processes, such as metabolic process (Bra004261, Bra008711, Bra007679, etc.), response to stimulus (Bra037912, Bra031728, Bra006963, etc.), immune system process (Bra021594, Bra008056, Bra037864, etc.), and so on. The response to stimulus process is significant to regulate plant adaptability to the environment, and lncRNAs targeted these genes. This result suggested that the regulation of lncRNAs might have crucial roles in plant environmental adaption. Due to suffering genomic shock induced by heterozygosity and polyploidy, new genomes go through a series of reactions, resulting in genome structure and gene expression pattern changes during allopolyploid formation. The variation in lncRNA expression patterns of *Brassica* allohexaploid may have an effect on their regulation function in a certain extent.

Finally, a limited correlation between protein expression and transcript expression existed in our results. This phenomenon also existed in *Arabidopsis thaliana* (Ng, Zhang et al., 2012), *B. napus* (Marmagne, Brabant, Thiellement, & Alix, 2010), *Agave Americana* (Shakeel, Aman, Ul Haq, Heckathorn, & Luthe, 2013), and so on, showing a wide spread low correlation between mRNA expression and protein expression. The lack of concordance between mRNA and protein abundance could lead to the condition that alterations on gene expression mediated by lncRNAs had no obvious influence on the protein abundance. This result indicated a complex and multiple regulation of post‐transcription, translation, and post‐translation processes. RNA interference, as a pathway for gene expression regulation, may explain the discordance between transcriptome and proteome. For example, sequence alignment between miRNAs and mRNA targets promoted mRNA cleavage or translational repression, which induced the differential protein regulation. The intricate regulatory networks could exist among lncRNAs, mRNAs, and proteins. The research of the regulatory mechanisms among three factors can develop a new field for studying allopolyploid adaptability mechanisms.

### 4.2 Regulation of lncRNAs might increase the possibility for adaption in *Brassica* hexaploid

Gene expression changes in allopolyploids could be an adaptive mechanism that contributes to evolution in the direction of stability (Pikaard, 2001), which finally leads to some phenotypic changes that are superior to parents. Small RNAs give rise to changes in gene expression in allopolyploid, which could enhance the possibility for adaptive evolution (Ng, Lu, & Chen, 2012). It is unclear whether lncRNA regulation could increase the potential for fitness during the evolution process of *Brassica* polyploids. Our data indicated that gene expression changes caused by lncRNA regulation could improve the possibility for fitness in *Brassica* hexaploid.

Compared with the paternal parent, down‐regulated Bra007678 gene (negative regulation of Calvin cycle) in *Brassica* hexaploid was controlled by down‐regulated TCONS_00076445. Photosynthesis is the basic guarantee of survival and reproduction in plants, and the Calvin cycle as the dark reactions of photosynthesis play an important role in CO_2_ assimilation. It could be proposed that down‐regulated TCONS_00076445 is conducive to enhancing the photosynthesis ability for *Brassica* hexaploid. Ultraviolet exposure cuts down leaf length and plant height, and anthocyanin pigmentation could protect plants from the harmful effects of ultraviolet light (Klaper, Frankel, & Berenbaum, 1996). Up‐regulated Bra007957 is an anthocyanin biosynthetic gene identified in *B. rapa* (Guo et al., 2014), and up‐regulated Bra007962 is involved in the lipid catabolic process, while both of them were targeted by TCONS_00017262, indicating that TCONS_00017262 is likely to facilitate anthocyanin biosynthesis to induce anthocyanin pigmentation accumulation to protect plants, and lipid metabolism. Up‐regulated Bra031483 takes part in a metabolic process resulting in cell growth. AT1G05530, its orthologous genes, involved in IAA metabolic pathway, plays a vital role in IAA homeostasis (Tanaka et al., 2014), and TCONS_00009958, targeting Bra031483, could be involved in the IAA metabolic pathway and play an important regulatory role in growth and development. Up‐regulated Bra008064 (an ABA receptor) was targeted by TCONS_00017333 and can reduce the free ABA content, and the decrease of the free ABA contributes to floral initiation and flower development (Su, Huang, She, & Chen, 2002). Compared with *B. rapa*, down‐regulated TCONS_00017333 could activate the expression of Bra008064 in *Brassica* hexaploid, making for floral initiation.

Compared with the maternal parent, up‐regulated Bra019369 in the *Brassica* hexaploid encodes a SAUR (small auxin‐up RNA) protein (Chu et al., 2014), and this protein is tightly tied to auxin biosynthesis and the signaling pathway (Wu, Ma, Chen, Wang, & Wang, 2012; Wu, Liu et al., 2012; Wu, Okada et al., 2012). Up‐regulated TCONS_00018406, as a Bra019369 regulator, possibly take part in auxin‐dependent metabolism, thereby regulating plant growth and development. TCONS_00082637, the regulator of up‐regulated Bra008668 (homologous to the flowering‐time gene), could positively regulate the plant flowering process, contributing to the reproductive growth process for *Brassica* hexaploid. Succinate dehydrogenase is the most important dehydrogenase in the tricarboxylic acid cycle, and its activity is generally used as the evaluation index of the tricarboxylic acid cycle operation level. TCONS_00027367, targeting up‐regulated Bra000428 (encoding succinate dehydrogenase, a component of mitochondrial respiratory complex II), possibly takes part in regulating the tricarboxylic acid cycle in a positive way, making *Brassica* hexaploid use more energy for various life activities. Compared with *B. carinata*, the expression level of Bra008711 (encode an FtsH protease and degrade light‐harvesting complex B3 during high‐light acclimation) targeted by TCONS_00082672 was very low, with almost no expression in *Brassica* hexaploid. Light‐harvesting complex B3 is part of photosystem II, and its main function is to absorb the light energy and quickly transmit the light energy to the photosynthetic reaction center, evoking a series of chemical reactions in photosynthesis. It is suggested that TCONS_00082672 probably makes *Brassica* hexaploid continue the photosynthesis process under certain high light conditions.

LncRNAs can function as a member of the miRNA regulation network to realize the regulation of gene expression (Wu, Wang, Wang, & Wang, 2013). For example, the interaction between miRNAs and lncRNAs is an important mechanism to maintain phosphate homeostasis. MiR399 is induced by phosphate starvation, and the *PHO2* gene, a target of miR399, is repressed so that plants increase phosphate uptake (Pant, Buhtz, Kehr, & Scheible, 2008). *IPS1*, a long intergenic noncoding RNA, can carry out its functions as a target mimic of miR399. It seems that the increased expression of *IPS1* could counterbalance the influence of miR399 accumulation (Franco‐Zorrilla et al., 2007). Bra‐miR5721, bra‐miR5711, and bra‐miR5716 are responsive to heat stress in *B*. *rapa* (Yu et al., 2012). Their targets (TCONS_00035262, TCONS_00080146 and TCONS_00060804, respectively) were differentially expressed between *Brassica* hexaploid and *B. carinata*. Similar to phosphate uptake homeostasis, each relationship pair may play an important role in response to heat stress. In addition to the above three miRNAs, eight other miRNAs (bra‐miR167a, bol‐miR157a, bra‐miR5712, bra‐miR5713, bra‐miR5717, bra‐miR5718, bra‐miR1140, and bra‐miR156g) are also responsive to heat stress (Chen, Fu et al., 2015; Chen, Quan, & Zhang, 2015; Yu et al., 2012), and a total of 11 lncRNAs were targeted by them. Bna‐miR156a and bna‐miR167a are responsive to salt stress (Ding et al., 2009), with the former targeting TCONS_00017006 and TCONS_00065829, and the latter targeting TCONS_00042206 and TCONS_00023069. LncRNAs could be involved in the miRNA regulation networks that are responsive to heat and salt stress and the interaction between them might have important effects in response to stresses in *Brassica* hexaploid.

## 5 CONCLUSIONS

In this study, we analyzed the differences in the lncRNA expression levels and regulation on genes between *Brassica* hexaploid and its parents, and these differences may contribute to *Brassica* hexaploid survival. LncRNAs could directly modulate gene expression by cis or trans‐action, and interact with miRNAs or act as precursors of miRNAs to indirectly implement control functions. The diverse ways of regulating gene expression could play an important role in adapting a wider range of surrounding conditions for polyploid plants. Our results provide a new perspective for studying the mechanisms of polyploid evolution.

## CONFLICT OF INTEREST

None declared.

## AUTHOR CONTRIBUTIONS

JW and RW designed the experiment. RW carried out the field sampling and gene expression analysis. RW wrote the manuscript draft and JW revised the manuscript. JZ and JM provided the experimental materials. All authors read and approved the final manuscript.

## DATA DEPOSITION

The raw data of RNA‐seq reads were deposited in the NCBI database under accession number (SRR5428550, SRR5430783, and SRR5456947).
