# Supplementary material for: Integrative analysis of genome‐wide lncRNA and mRNA expression in newly synthesized Brassica hexaploids
Source: Ecol Evol. 2018 May 15;8(12):6034–52. doi: 10.1002/ece3.4152 (PMC6024132; doi:10.1002/ece3.4152)
Supplement: Supplementary file 1 [file ECE3-8-6034-s001.docx]

>TCONS_00056834

CGATGAAGATGCAATCCACTGTTTTAGGTCCAATAGTGACATTCTTTGGTGGCGGTACCGCAACTTTTGCCAGGCACCCCCACACTTTGAGGTATTTATACGAAGGTAAATTACCTTTCCATAATTCATATGGAGTTTTGCCAGTTATTTTGTGTGGAATTTTGTTGAGGATATAATTAGTGGTAAGCAATGCTTCCCCCCACATGTTCTGGGGTAACCCAGATTCCTGCAACATAGCATTCATCATCTCTTTTAGAGTTCGATTTTTTCGTTCAGCAACTCCATTAGATTCTGGTGAGTAAGGAGCTGTAGTTTGATGGATTATTTCATGTTCTTTACAGAATGCATTGAACGGACCATCATACTCGCCTCCTCTGTCGCTTCTAACTACTTTAATAGTTGTTTTAAGCTGATTCTCGACCTCGAGTTTAAATTCTTTAAATTTTTCCAAGGTTTCATCTTTGCTATGTAATAAATAAACATAACAATATTTTGTGCAGTCATCTATGAAGGTCACAAAGTACTTTTTACCACCTCTAGTTTGTAAGTATTTTAAATCACATAAATCAGTGTGAATTAAATCTAGAGGTTTATTAGTTCTTTCAACACGAGGTGATGGCGTTTTTGTGAGCTTAGCCTGTACGCATACTTCACATTTTTGTTTACTTGTTTTGCATTTAGGAATTAGATTTAAATTCATTAATCTTTGTATAGACTTGTAGTTTACATGGCCTAATCTTTCATGCCATATATTAAAAGACTCAACCAAATAAGCAACTGGCTCATTCTTATTCATTGAAACTTTTGGAGCTACAACTTTTGGAGTGACTGTCATTACATTCAACTTGACAAGTCCACCCTTAACATAACCCCTACCCAAATACATCCCATTCTTCCTAATCACGAGCTTATCCGCCTCAAAGCTTGTGGCGAATCCATTCTTGCTGAGCAAGGTTCCCGAGACCAGGTTCTTCCTCATGTCTGGCACATGCTTCACATTTGTCAGAGTGACCTCACGTCCAGATGTCATCTTCAAGATCACATTGCCGCGTCCTTCAATCTTGGAGACTGCAGTGTTTCCCATCTTGAGCTTCTCCTCAGTCTTGCTTTTCTGATAGGTGCTGAACATCGCCCTATCGGTGCAAATGTGGGTGGTTGCACCAG

>TCONS_00006000

CTTCATCTCTCTTCTTCGTGCGGCTACGACTCGGTGACGTCTTAGCTTCTCCTCGCCATCTGAAACGGTCCGAGTTATGGCTGTATCGAAGCGCCTCTTCAATTCCCTCATTAACAGTATCTATTCACTTCTCTCACAATCTCTCATTCAGTGGATATCCATTACATTGATTGAGTGAGTCTTCGACTATTTCGACTATAAATATATGGATTTAACTTCTTATAATCAGAACCTGATACCTTCAGAACCTGATACCTTCAGAACCTGATACTTAAGGAAAGAGAAGAAAAGAAACTGAAAGAACAAGCTCTGCTCTTCCACCGAACCCAAGGAACTGAAAGTAGCACTTGAATTGAAACACATACGTGTTCTCCACATGAAATAGCTGAATATATTGTGGTTCCTTTTGTATTTAATTCAGCTTGAGTTATATGTGTGTTTAAGACTTTTAGTGCAAGTTGTTTTCTCTATTATGTTATTGTTGTAAACCTGTGATGTGATCATCTTTGATCAATAAGTTAG

>TCONS_00065375

GGAGGAAGAGATGCAGGATCTGAACAGGGCTACAAACTCGAAGATGGCGTGGTTGGGTTTGCTCTCTCTTTTTGTATGCTTAGGAGTTGCTGGGATGCAGTTTATGCACTTGAAGACGTTTTTTGAGAAGAAGAAAGTCATCTAAGATGATTTTAGATGGAATAATTCCACTCTTTCTTTAATCAGCTCAAGGGAATCTTTAACTCTCTAGTGGGTTTTGTATATTCAGATTTTTGTGGCTTGAGCATATGGCCTGCCTCTTGAACTAAAATGAATGTGAAAGTGTTATGCCATTTTTGCACTCTCTGTCAACAGAAAGAATAACTTGGAAAGTCATGGAGTTTGAAGTAAAGGCTTTGATTGAAACCGTTAACTCGATTCATGATGAGATGTTTTATCTTAGAGACAGGTGACGTCGAGTCAGGGGAACACGTACCATCACGCGGAGGAGGTGGACTCGGGGCAGTTCTCGTTCACGGCGGTGGAAGGTGGAGAATACTCGGCGTGTTTCGCTGCTCACGATCATAAGCCTGACGTGACGTTGAGTATCGATTTGGAATGGGGAAGTGGTGTTCATTACAAGAGTTTGGGTAGTTTGGCGAAGAAGAGCAAAGTCGAATTCTCATCAATTAGAAACACGCACGTGTCAAACGCTCACCTTCCTCCTCCGTCGTCATCGTTCATCTCTGCAACAACGTACGACCGAGGAGCAAAAAAAAAAGAAAGACCTCTCCTTTAAGCATTTCATCATGGTTCTCCGACCAGAATATCTATGCACATTCCTCTTAACCATAGCCATCCTATCGCACGTTTCCCAACCCCTCCACTTCGAGTTAAAATCAGGCAAATCAAAATGCATCTCGGAGGACATCAAAAGCAATGCAATGACCGTCGGAAAGTACACCGTCGTCAACCCCAACGAGCCTCACCCTACTCCACAATCTCACAAGATCAACATAAGG

>TCONS_00049719

TAAACACTTCTGAATCTCTTGCTTCGCAATACAACCTTTATGACCAAGCAGAGAAGTTGAGATCAAAAGTACCAAGATTTAAAGCACGTTCCAACCAACAGGTTTATCTTTTAGTAGATATTCTTTCATACCAATTGGTTATTGTAGCTTTCTAGATTAGAGTTTATATTATTTCTCTGATCTCTCACTTATGCTAGCCTTTCCACAGTAAATTGGAAAGTAAGTAATATTGGTATTCAAGCTACCCTTGCACCATTCTGCAACACTTTGTCATGATGCGTTGGATCAGATTCTGGGAAAATAGGAACTTCAAGCGATGTGGCTCTTTTCAGCTAGCGTTACTCGCCTCAACAACTTAAACAAGAGAACCCGTGATGTGATGGCTTCAAGTTTTTGCGTTTCTGTTGGTTCGCGTCGTTTCGTTATCTATTCTTCTCCTCACATGAGTTACAAGTTGCATTGATTCAACTTTATTAACGAGATCTTAACTCTTACCACCATCTGCAACCAAGATCATTCATTCAATGATCCGTATCCCTCTTCCAATCGGTGGATCTCCACCTATAAAAAG

>TCONS_00005559

GTGAATGATAAAATTGTTGCGTTGTCTAGTGTTTTTCTGAAGTTTTTGTTTCTTGTGTGGAAGGGTATATGGTGATGATACAATAATGTTGTGGACTAGAGTTTCAGATCTGGGCACTTCACCCAGAAGTTGAAGAGTAACCCTTGGCTGTCTGAGTATTCCCTTTCACCTTTTTTATAAATTGTTTTGATTAATTTGGTTTTCTTTTTTATTGATTTTGGCTTAGATGGAATATATGATGAGCTTATGATGATAAAAACAATTATCTTAGCGGATTTCAGTGAGGTTTTTATTGATCAATGATCTGAATTTGTTACACACACTGAAGCTCTTTTGTCTACCTAGTTTATGTTTTTTTATTATAATATACTTTTGATGCTATGATGAGATGAAAGCACGAAGAGTGTAAGAAGAGCAGTGGACAATTACAAATTCTGTAAAGGGTTTGTTTTGCCGTGAGCTTTACTAATTAAACGACTTCTCCTTCCACTGAGCGTATTGAAATTGTTTTTAGTGTTTTTTTGGGCGTCCATTACCAATTCTGAGGGATAAGACAATTCCTTCAGGTATAATGGCTTGTGACGCAATATCAATTTGTGGTATATCCTGATCATCTTGCTCTTTTCACTGCTTTTTGATCCTGGAAATGCCTCGTATATTATTTAACGATTGCTTCTTTGATTTACTAGTATCTCTCTCTTCCATAATTCATTTGTAGTTCTCTTTGCATATCTACTGGTTTTCATCGTTGCCATGATTGTGTAAAGGGTTGAAGAAGCTCATTGATGTGTCGCCACCCCTTTTGTCAAGAGTTTGAATGGCGCGAAGCTTTTACGAGGCTGAGATTGGTGGGAGGCCAAAAAGCTATAATACGATGTGATTTCAAGTTCGTTTTTGTTATTTAAGCTCATACTGTGTCCCTTTAATCTCTGGCCTTCCTATTTTGGTAGCTGTTTTGTTTTTTTTCGGAGAAGAATGTAAATAACAAAATGG

>TCONS_00066000

TAAAAAAGCTGCAAAGTGTTAAATCATGGCCGATCTTGACCACTTTGGATTGGATACATACACAAAGATAAAAGAAGATTATAATATAAGATGGTACCATCACATATTATGTTAAGGACTTTATGGGTTGTGGTTAAGGTCACGTCGTTTTCTAACATGGCTGTCTTCTCCTTACAAGCTTCATCAACCATTCTTCATCTCTTTACTATCTTTTATCATGTTGCCTTGAGGAGTGTGT

>TCONS_00057801

GTGACTGCAATGTTATCTCTTGCAGCTGCGTGTTCATCAGCAGGAGTTGTAGTCTTATTTGCCAGAGATCTTAAGTATTGCAATATCCGTGAGCAGTTTTCATGTCTTAGGTATCAAGTATCTGTAGCGTTGGCTTTCGTCTCTTGGTTTCAGATTGCTGTTTCTTCTCATGTCACATTTTGGATCTTAGCCTCTGTTTAGCTACTTGATTGCTTCGATGGGTCTTCAGTTGCTGTGGAGCTTTGGCCTCGCATGTCTTGATGTTTATGCTCTGAGGGGCAAGAAAGATCTCCAAAACCCAATCTTGGTTAGCTTATTCGTCGTTGGTGATTGGCTCCTCTTTAATCAGAGTTTCTGGGTTTAGGGTGTTTACATTTGGAGATGAAGAAGATAATCGGAGGTCCAGGAACTGTGTGTGGTCTGTTACTTAGAATCGGACAATGTGCTTCTGCTGCTGCTTCAATCGGCGTTATGCTCTCTTCCACCGAGTTCTATAACCGCACTGCCTTCTG

>TCONS_00006588

GTGAAATAAGACCTCCCCGAAATGAAAACTAATGGGCTTTCTTCAAGGCAGGCCTGTAACGAATTAAAAGGCCAATTAATCTGACCCATCGTTTCTGAAGTTTACAGCCTAGAAAACCCTAATTTCTAACATCCCCATGTATATATACTAAACACTGCCGGCGAGAGATGTAAACATTCATACGATCTCTTGTTTGTGTATATTTGCTGTTAGTTTTCATCTTAAAAAATGTTTTGGTTACGTGCTTAGATTCGCCTCTCTACTGGATTTTCCTGTAGAATTTTAAATTTCTACCGGATGAATCGAGAGGCGATAAATTTAAGCAGGGGCGTTGAGTCGCTTCGCCTATGTAAAAAACAGGCATAGCGAATAAGCTCGTGCTATATCTAATTGTTTTTTTTTCCTTGTTTCTTTGAGGATCCTATGATGGCATGAAATCTTTGAGACCTGATTTATATTGATGAAACGCTATAAGCATTTATACTCTGAGGATCTTATTTATTTTTGTATTGGTATTGTGTTTTTTGAATTAAATTGGCGATTTGAATCTAAAGATAGAAAAAGATGATGAATATGCACTGGGCTCTGAGTTGTTGATTTGACAGTCGACCTCTTTTTGATGGATAATGCAGCCTAGAAAGCTTAATCTGATTCTTCTATCTTTGTTTTCGTAGTTCTCTTAAAATCTTTATCCTGTTGTTGTATCTTGTCTGAAATCTTTACTTGTGTTGTTGTAGATACTTATCAGCTGTACCTTTACTTGTAATTGCTGTGACTCTGTTACGCATTGTAACTCTCTCATGCCGTATGCGTAAACTTTCAGTTCAGACTTCAGAACCGACTTGCTTTGTTGACCATTGCCATTATGAGTCCTTGTATGAAATGC

>TCONS_00024904

TAAAAAGATGTTCGCCACCTATGAGAAAAGGTCGGAAGAACAGGATAAGCTCGTGAATACCTTGACCAAACAGGTTGAAACCTTAACGGCAAGGACTCAAGCAATCCATCCCCGCGGAACCACTAAAGTTCGCGGGAAAAGACTCGACTTCGCAACACCACTCGATAGGCCTGGAACCGCGCGGTAACGACCAAACTGTGGAAGTACGGATAAAACTGGGGGTAGATCGGCCTTGGTCCGACCATTCGCTACGTATTGAGTGACCAAGCCAATCGGTCGGTCGCTACATATTGAGCGACGAAGGCACTCGGTCACTCACTAAGTATTGAGCGACCAAGCCACTCGATCGGTCGCTACGTATTGAGCGACCAAGGCACTCCGTCGGTCAC

>TCONS_00045743

GTTCTATTCTTCTCATTCTTTTCATCCAATTCTTCTTCTACTCCTTCTGTACATTGGCGTGGCCTTGTTGATGTGTTGGGATTGGTTATAACCGTGGTTCTGGTTGAGTAATGAAGTCGCGGTCCATCCGTCGAGTTAGGCGCGCGCATGGTCTCAACCAGCCTCAGGTGATCCGATTGGATATGGGCGGTTCTGTTCTGTTCTGAACGGTTGCAACCCTTCCCTGAACAGTCACAATGGTTCATGACTTGTGTAGGTTAAGGCGTGGTCCTTTGGCCATAGGCCGGACACACGTACAAACCAGACAGTCCGTACGGACAGTTAGGTCGAACGGTTGGAACATCTGAATGGGTGCGAGTTGCCAAGGGTCATGAGTTACCAAGAGGCACGTGTGTCCAAAGGGTACTAGTTCCCAAAGGGTGTGAGTTCCAAACGGTGCCATTGGTTCAAGGCTTAGGCCGAACCAAGTGGACAGTCCGCGGCTGCATAGTCGAACGGACGGACGGTTAGTTTAACCGGAGTGTTGTGTCGCAAGGTCTGATTGGTTGCAAGGCAATCCGTTTTGGTCTTGGGCCTTACTCTGTGGTATGGATCCAGGCTTTGGGTCGGATCAGGTAAGAAGGTCCGTGAGCCTTTGGGCCAGACTTATAACTGGTCGATCGCACCTAGATCTTAACCAGACGGTTAGACGGGACAATGGACGATCCGGCTATGGTTGGATGGTTCTAGCCGCAAAGGCTAAGTGGGATATCTTACAATCAACTTTGGGTTGGTGAGTAGGATAGGCGCCATATGCCTTATGTAGGGCGTAGACCCACATGATGGCAATGGAAGGCCGGTTATATCAGTACATGCTAAGTGGACGATGGCTTATCAAGTCTAGTGGTCGGATCATGTTTCATGACGATGGTTCGATGGCCGAGCACTAGTATGGATAGTCACGTTGCTGGAGTAAGAGTATTGATTTGATGCTTCTAGATATCAACCTTGGTGTTGATAGCTTCGAGATTGGCTAAGTGTCATGACGAAGTGTGTGGCTAGTACTATGTGTCGTAGACCATAGATACTGAGCCTAAGTGTGATTGTTCAAGGAAGGCCGTGTAAGATCTGATCATGGTTGAGTATGGACGACCTGACCTCTGAATGATGGTTCAAGGTGTCGGTACTAACCTGATTAATGAATGCATCGGTTGGTATGAACAAGTCATATATGTTGTCTGGGTTAAGTCCCAAGGCCGGTCAGGCCAGATGATGACTCATCAGTTCCAAGTCATGTGAGGATGGTTGGTTGATTGACTTAGGATCTCATGAGCTTTGTCAGTCCAGAAGATGGACTTGGTACTATTGCCTATAAGGCAAAAGGATTTCGGATTGTGCTTGAGCCGTTGGACTCAGCACGTTGGCCCTTATCCTTTCGAAGACTTCTCAAAGGTTACTTATGTCTGTGGGGATGGTTGGTTGAATGACTAAGTACCTAGGGGAGTTGTTTGATGCAGGAATCAAGATAAGGACCTTAAGTAAGATCATTGAGTGATCGTGGTCCAGCTGTCAAGCAAGAGCAATTCGAGTCAATGGAGGACCGATGAGCTAATAAGCTCCGTAGATATGGCAAAGGTATGATCTAGCATTTACTTATGTAGATCTAGATAGAATGGTTTAGGAGAATGAAACCTCAGAATCGTATGGCTTGGTTTGGGTTTAGGATTTACCTTTAAGCTAATTGGTAGTTGAGTAAACTGACCAAGGCTAAGGTTATTCAACCAGACAAGTGTTAGATTAGTTTTAGACCAATGGTTAATTAGAGAATAATCCGCTGC

>TCONS_00030747

AGATTACCTCGTACCTCTCTAAGGGGAGAACTAACAAATCAGCCGGAAAAACCATATCTTGGATAATGATTGGAACTGTTAGAATACAACCTTCAGTTTCAAGAACATGATCTCCGGGAGTTAGAACGGGAATGGATAAATTCACCTTAGTGAATTCTCCATTGAGTCGGGATGCTACTTCGGGAGCTACAAAACTATGTGTTGCTCCTGATGGTCCTGGTAACTCTAAAGCGTAAGCCCTACCAGCAGTGGCTTGACGCTTAGCAGGTCTAAGACTGAATAACTGCACAACCAGGCTGGTGAAGGACATAACTTAGTTCTAAAATTATTTCAGAAGTTGGTCAGTAATTTTAAATCATTTTTTAACCAAATATTCCTTGTCTTTGGCTCTCGTCTTGTTCTCGGAAAATCTCTTCCAGTTTAATAAAAATTCGACCAAAATATTTAAGACTCGACCAAACTATCAAGTGAAGGTCTTTCAACCACAAGACAGTCCCTTCGAGAAATTAATCTACCGGGTCGATTTTTATTTTTATTAATCCTTGTTGAACCAACTAAAAACTGGTCGAGCGGGATTTTTCTCGACCAAAAATTTATTGGCTCATGAGGGTTATTACACTCTTCCCCCCTTAAAAGAATTCGTCCCCGAATTCTCAAAATACAAGTGTACACCCTTTACTGGACGACTGGGGTATCTGAGAAGAGTTCTGGATAATCAACTCGGAACTTATCTTCATCCTCCCATGTTATAACAACTCTCTGTCGCTTTCCCCAAAATACTTGAACTTGCAAAATTT

>TCONS_00074010

CTTTGCACCTTCTAGCCTACTTGCTTCCTCAGTAATTAGGTCACATGACCTGCACCTACTTGCTTGTCTGCTTGCTCATTAATTCAGTCACATGATTGTCACCAGCTGCTTGCAGCTTTCTTCTTGGGAGGGAAAGGACAGCAGCTTGCTGTTGGAAGTGCTGAAGCTGCTCTCCACATGTCCTTTCTCAGCCTTGCTTTGTGCTGAGTGAAACCCTCACCTGAAGCAACTTCTTATGTTATAAAACACCACCTTCTCTCTTCTGGTCGTCCATAACCACAAGAAAATCAGAGAGAAACTTTCAGAAAAATCAGAGAGAGAGAAGAGAGAAAATATGAAAGAAAACCATAAGAAAAATTCAGAGAAAATTCTGAAGAGAGATCTGGGAATGGACGACCCTTCCTAACCCTCTTGTGCCTTGAATCAGTGACTGGGGTGTGTGGAAAAATTAATAGATTGTCTAGAAGATTCATGAGATCACTTCACAGCTTAGTAATCAAGTATTAAACCAGTAAGATTTTAAGTCAGTTTGATCAGATCAGTTTTAAGGAAGCTTCATGTTTGATTAAGGCCTAAGCCATGTTCTGTTTCTTATCTTAAACCTACTGTGGAACCTTTATGCTGCTCAGGTCAGTGTGAGTGGAGATTGAAGGAGAGGATAAGTTGTGTTCATTGATCAGTTTAGTATAGCCGGTAAGCCTTACCTTCTTGCTCTTAGATAAGTTCTGAAAAGAACCGGTTATTGGTTGATGAATAACCTATCTGGATCAGTTTGATTAAGGCCTATGTGGATTGTTATTAGTCCAGTTCATGGTCAGCTAGCCATGTTGAGTCAACACGCAAACGATTTGTCTCTGAACCAGACTGAACTGATAGATGGATCAGATTATTGTTCTGAAACTTCTTAGCTATTGTTATAGTATGAACCAAACTTAGTCTGAACTAAACATGTTCTGAACTTCCCTTGGTAGCTGTCCTGAATCATGAACTGCATGGTTGAATCAGTTTTATACAACCGGTCGATTGGAGTGTTTTGTCTTGCTCGGATCAGATGTAATGGAGAGTGGTTGGATCAGCCAGACATGTCCGGTTGATCTTTCCGAATCTGAAGTGTACCAGTAGTGGAATGTGATTAGCTTGAGCTCGTGTCTAGTCTACCATAGCCAATCTCAAGGACTAAAAGGTGAGTCTAGATAGACGGTTGATCAATACTGAATGTAGATGATTGATAGGCTTTGTCTCTTGATCAAATATTGAATTGGTGAGGTGTTTACTTAATGTAAACACTTATTAAATATTTATGAATGATCATAGAGGTTTATTCCAAACCTTAGTACTTGTTCTCAAGTACTAATGCATTTGTATAGTATTAAATATATAAGTATAAATCATGTGAAGTCTTATTGTATACTCACTCTCCCGAGAGTTCCCGCATTACCGTGAATTGTTCCTGCGATACGGAACAAGATCACGAAGACACGATGATGGGGTCGCACCCTGGAGACCGTGTAACTGCATGCAGCGTTAGATGTTCGATCTTGGAATATCTAATGGAGATGATGGTTATATTTATTTC

>TCONS_00079143

GGGCTGATCACGTTGTTTCAATATGATACATACTTGACGAAGGGACTTCTCATTTAGCCTTCCATCTGGTGAGAGGTAGCGAAACCTATGCCTTCCACCTTCATCCACAATTTGTTCTATGGTCCATCCCATATACTTAAGATGCTTCCTAACATGGAAAGCCACTCTCTGTAACGGCGCCCTAATATAGCTCGTCACGGCTTGTGGGCATGATTTAGCAACACAATCAAAAGGTTGCCAGGTTCCAAGTTTATCGGGTAGTATCAACTTGATATGGTCGGAAGTAGATTTTTGGCTGGAAAGTTCCGTTACCCCATTGATGTAACTTTCCTCCGACCTTATGCCAGACAGAGAAGCTAAGCCAGGCATGGACATGCATCTGTGATCTTTGTCATTGGAGTAAAACTGTTGTTCTTGTGGAGCAATCACTTCCACTTCCACACCACTTGGACAAGCATAAGTCTCATTAACCGCTTGAGAAGCTTTTTTGACTAGCTCGAACTCTTGGCGTGGATATCTTTCAGCATCATAATCACGAAGAAACTGACTTATGGTGATTTTCAGATTATCCTCAATCACCTCCACCATCAAGACTTCCCATAAATGCCTAATAGAGCAAGTCCATTCCCTGATCCTCGCAAAACCAACTCTGACCCGTATGTCATACCAAAGCTGCTTAACCGAAACCGGGAGATAGTTATCCTCCTCATACTTCTCAACCAACTCAAGAAACATCCATCTCCCACGACACTCCCAAGCCTCTGTAACCTCATCCCAATCCTGAGTAACACGCAAAGATTGAAGATCAGCATCAGTTTCATCACCCAAATCAGGGAAAAAGACTCTCCTTTTCTCAGAACCGTTTTCATGGTCAAAAACCACACCTTCCCACCAAGCTTCACTGATCAATACATCAACACAGAGTCCATACACAAGGCTGAATCGATCAACTTCCAGCTTCGGCGGCACAGGTCTTAAACGTCCACGTAGACTACCAGCAGAAGAAGAAGAGCTCAGCCCTTCCACTACCTCGGAAACATCAACAGTCTCCACAAGATAATCAGTAGCATCATCAGAGAGGATGTGATCGTATCTGATGCCACGGAGGCGTCGCTTTTTGGAGGAAAGAACAGTTGCTGAATACCAAGAACCGAGAAACCCTTCTTCAACACTTCTTAC

>TCONS_00018326

GAGATTGCTCAGATCAGTGGTGAGATGATGGGTGGCGCCACTGTCGAGGATCCAGCTATTTGGATTGTATGACTGTGCCATAGCCATATTAGCACGTGGTTGCCATGCGGTTGGACCTGGTGAGAACGATCGAGCACCGGGCGCGGAGGAGTAAGCAGCGCCCTGCAGTTGTGGACATCGTCGAGCGCTGTGTCCTTGGACAGCACAAATTTGGCAGCGACCCTGATAGCCACGAGAAGACTGATCACCACGGGCAGAGAAGTTTTGTTGTTGCGACCAAGGCTGCTGTTGGTTACGATTGTTGTTGTTGTTGCGGTTCTGCCCATGATAGTTGTTGCGACCATTGTTGTTAGAACCAGTTGAGTTCCCACGGTGAGTAGCTGCATTCGCGGTGATGGGAGGTGATGACAGCACGCCTTTGCTCTGAAGCTTAACTTCATGATTCAAGAGCTTTTCATGAACCTCCGTGAGGGAAGGAGGTGAGTCTCTGCCTTCGATGGCATCAATGACCTGCTTGTAATCTTCTGGTAGTCCTTCAAGAACATACTCAAGCTGATCTTCAATGTCAATTGGCTTGCCGAGTAACGCTAACTGATCAAAGCGCGTAATGAAGCCTTGCACATACACATCGATCGACGTCGTTCCTTTGGTCCAAGTTTTGAGTTGTTGACGGATCTGTTTGACGTGCGCACAGCTGGGCTTCGCGTACGTCTCTGAGAGTGTTTCCCAGATGTGGTTTGAGGTCGTTGCGGTAGAGAGGATTGGTTGTATCGTGACGGTGATAGCCCCAAGCAGCGCACTATAGATCAGCTTAGCTTGTCGTTTCCACAGCGTATACTCCGGATTTGGCGTAGTTACTCCATCGACAGTGTTCGTTGGTGGCGGAATTATGACCGATCCGTCAAGGTGGCCAGCGAGATCATATCCATCAAGTAGGGCGTGGACTTGACGGCTCCACATCAGAAAGTTTGAAGCCGTTAGCTTGGTAACATTTGTCATGTTGATGTTGAGAAGCTTGACAGTGTCAGACACTGTGATTGTTTCAGCGTTAGAAGCCGGTGAAGAAGAAGACATGATGAAGCGTGAAGAAGAAGAAACATAAGAAGGGTAGAAAGAAGGGTTGGCGGCTTAAAAATTTTCAGATCCCTAGATATCAAGGCTCTGATACCATATAGAGTATGTAGGCTGAATGAGTTTATTATGTAACGTGATTACAATATTTATAGTGATTAGAGGAGCTAAGCTAGGTATTACAGAATGACTAATCTACCCTTCTATACATATACTCTACAGTTCTTACATTGAAATCTT

>TCONS_00018349

AAGTCTGGCAGCTTAATTCTCAAGTCTGGCAGCTTAATTCTCAAGTCTAGCAAAGATAAATAATTAAAATAAAGTCTTGAACTGAGATCATCAAATGAGTGATAGGAAAGCAACATATGGCCTCATTAAAAACCTATCTTTGGAAAACCCAGTGGGACAAAACCAAAGATAGGGAAAAAGAGCACACATATGTTACTTGCTCATTTGATGTCTATCTTGGAGCTGAGATGGCTTGAATTGTGGTAAGTGTGTCTTGGTTGATCAAGCTTCTTCCAAGACTCTCTTCTTGCTTCAGTGATGTCTTAAGTAATCCTCCAATGGCTTCTTTGAGTTGCTTGCTTCTGGCTCGAGTCATAGGACCTTCAGATATGGCTAGAGCTCCTCCATCAGCTGGATCTTCCATGCTCCCATCATCTTCTTTGTTTGACTGGTCCATGATCATATCATCCCCTCCCTCTTGAAAAGGATTTGTCCTCAAATCTAGCTCATCTGCAACAAAAGGAGCCAAGTCAGTAACATTAAAGCTATTACTTATATCATACTTACCTTGAAGATCAAGCTTGTAGGCATTGTCATTGATTTTCTGGATGACTTCAAAAGGGCCATCAATCCGCGGCATGAGCTTTGATTTTCTTTCATTTGGAAACCTCTCCTTGCGCAAGTGAACCCACACTTTATCACCTACATCAAAGATTATCTCACGCCTGCCTTTGTTTGCTCTTTTAGTGTACTGCTTGGTTTTCTCCACAATGTTTCTTCTTGCCTGCTCATGAATCTGCTGCACCATTTCTGCTTTCTTTTTCCCATCCATACTCACCCTTTCACACTCAGGTAAAGGTATTAGATCCAAAGGTGAGATGGGATTGAACCCATAAACAATTTCAAAAGGAGAATACTTAGAAGCAGAATGCTGAGCATGATTATAAGCAAATTCACACTGAGGC

>TCONS_00017591

ATATTTTTCTGTGAGAGCAGTTCAAAACATTAGAGCAGGTGTTAACACCTCGAGCCGGTTGTGTTATTGTTTCGCTTGAGAGAAACTTATTCTGTTATATATAAGTTTAACTAATCAAATATTTGAAAGACATACACGCATTTAAATGAATCAAACGCTTACAAAAATAGGGAAAAGGTTTCAAATTCCTTTAATAATGTTCGAGTTTCGTTTCGACTTGATTGCTAGCTAGATTGAGATACAACAATACGGAAAAGGTTTTTTTGTTTGCTTGAACAAACGCTTTGTTATTTTCCAAATAGAGAATCAACAGAAAAGGGAAAAAAAAATGACAGACTTGAAAAAACAATACGCCCACCGTGTACCTCGAACCCACAACCAAAAGGTTATGAGCCTTGCGCTCTACCAACTGAGCTAGACGGGGTTGGTGATGTTAACATGTACAAGTTCAAAAAGTAACAAATACATTTCAGACATGACGTTCAAAAATGAAAATGTTTTAAACTTCGATCACAAATTTCTGAATGGGATCTAAAAGATATTCAAATTCAATGACATCTACAAAATTTTCAGCTGTCATATGAACTAGCTCGAGCAACAATGGAGATCAAATCAAGCCAGCCGAATCGTCCTCGAGAACCTTGGTATCAATCCAGCCGAATCGTACTCGAGGTCCAAACTCTTTTTTTTTTCAGAATTTTTTTCTCTGTAGCGTGTTGAGGCAAGGCTTTTGCGTTTCTGGGAATCCCGAAATGTTAAGAAAGATGGTGAGCTCATGGGCTTTGATATTCTTTTGGTTGATGGCAAGGTGTAGTTCCTCGAAAAAAAAATGAAAGGTGTTCTCACCAACCCTAATATGAAACAAAGATCCTCTTTCCCCTTCCTGCAGCAGTGAACTGATGACGTTAAGGCTTCCTCATAATTTTCATTTATTAGCTCCATCTCGAATTGTATTCAAAGCTTTCTTACTATCTTAGAGGCGGTGCCATGATCTGTATAAATAGAG

>TCONS_00050480

GGGGTGAGCAATTTTCCTCTCTCTTAGAAAAGTTTTCTCTCATCCCCTTTTCTGAGACCAAAGCTTCGAAACCCTAATTTTGCCGGTGGTGGCGGCAGTCGCCTCTACCGCCGGCCTCTGTGTTTTTTCTTTATGTTCGCCTCTCACCTTCCTTCTCCCTCTCTGTTGATATGCTCGCATCTCTGCTTGGTGGTGATTCGGCGAGGCAGGGTTAGGGGTCGGTGGTGGTTGGCTCGGCGACGGGGGATGTCCTCTTCCCGACTCAGATCTGGAGGCTTTTCTCGGATATGGCTGTTCGCAGGCGGTGGCATGGTGCTTGAGGACTGTACCTGTAAGTTGTCTCTGTTTCTAGCCAGTCGGGTTTAGATCTCCGATGGTAGCTTGAGCGGGTTAGGGATATCTCGGGGTGTGTTAGTGCGGGGACTATCGTTGGTCAGACGGTTGTTGGAGATGGGTTAGGGAAGTGACGTTTGTTCCCTACGCTCACCCTCCATTCCAGATCTGTACCATGAAACATGAGTTTGAGAGTTGTTACCTGGGTGTTTTTCTACTCTCAATCTTCTATGTTGGCTGTGCACGAGGAAGAATTCTGCGGGTTTGCAGTGGTGTGTGGGGCTATCTACGCGCGTGTGGATAGCTGATGGCGTCACACTTGCTTCTCTGGCCGTGAAGAAGTGATATGCGCAGAGGATTTCATGGGCTTGAAGCTTCTTCTTTGTTGGGCAGTTCGATGGTGTGGCAAACTTTACCTTTCACGCGTCTTTCCGAATAAGAGCAGTGGTTTGTATGAATGCTTGGCTTGGTAGGATGGAGAAGAAGCGCAACAGTCAATCAATGTCTGGTGTGACCAGCATTTATGTGTTTTGCAGTGGTACTCGGCGGATGAGTTGACGTTTCATGCGGTAGCACAGATTAGCTAACTGTGCGGCAAAGGTTTTGGCTAGATCGGTGGAACAGAGGCATACAGTTTCACCTTTATCCGGATCTCCGGTTTTTACCGGGCCGAACCGTTCACTCCGAATGCATTCGCTTGAGCCATTGTGAAACTTGGAAGATGTGTGTGCTAATTTTGCAGGTTATCAAAGGCGTGTATGTGATGAAGCTGTAGGTCCGGAGTGGTTTTGAGGGCTGGCCAATTCTCCGTAAACTCGCGGCAACTTAGCAGCCACAACAATGAGACTCCAGCTCTTGGCTCACTGCGATCCTAGGCAAAGAGAGTTGTTCTGTTATTCAAGATCAGTATCAGTTATTGCATTTCTACTATTTACTGTGTTTTCCTTTTGTATTTTGGTGTTGTTTAGTTGTCTTTACTTGTTCCTGCTACTAGTTTTCTCTGTATCTTCTGTCAAAAACTCAAA

>TCONS_00063305

AGTAATGTTTTCTATCTCAGTTTCGAGAAACAAAGGTTGTTTTCAAGGTGGAGATGTCAAACTGAGACGCTCTCCTAGATGGTTAACTGAAACATAGCGATGCTCTGATGGCATAGCGAACAGCCACAAAAGCAGTGGTTCGGATTTAGGAAGCATTTAGAGATAATGCAATGAAGTCGGCACACTCAAAGGTCATCCATTTTCTTACCCACACTCATTTCAATATTATATTTCTTCCTTGCATATGCTATTAAACAGGTTAATATGAATCTTAGTTTTGGTCTCAGGCTTAAAATCAGAGT

>TCONS_00033051

ATTTTGCAAACAATCAAAATCCCCTAAAACTAAGAAAAATGGCTGGTCTAATGAAATTGGCATGCTTGGTCTTGGCCTGCATGATTGTGGCCGGTCCAATCACAACGAACGCGGCTCTAAGCTGTGGAACCGTTAGCGGTAACTTGGCAGCATGCATTGGCTACTTAACCCAAAATGGGCCCCTTCCCAGAGGGTGCTGCACCGGCGTTACTAATCTAAACAACATGGCCCGTACAACCCCGGACCGTCAGCAAGCTTGCCGTTGTCTTGTAGGAGCCGCTAACTCCTTCCCTACTCTCAACGCTGCCCGAGCTGCTGGACTTCCTAAGGCATGTGGAGTCAATATTCCTTACAAAATCAGCAAAAGCACCAACTGCAACAGCGTTAGATGAGCGGCGGTCGGATGAAGAAACTCAAGCGGACGTACGAATCTAATATAATGTATGAGAGAGTACTAAATAAGATGTTGTTTTTCTAGTGTTTTAAAATTTCATGTTTCTCTTCTGTTGTGTCGTTCCTTTACTTTGGTTGTATGTACTATGTTCGTAATCAACGTCCGCTATATGAAGTTCATATCCAAG

>TCONS_00036383

CCGGAGGAGACAGACGCACAACGAAACTTACGAAGAAGAAGAAGGATGATATACCGAAAGTGGAGTTTGCTTTCCGGTCCACCGGTTATCCTCGGCGGAGCTGTAGTTGCCGCGATCGCCGTCGGCATCGTCTTACAGAACGTTACGAAAGGGGAGCAAAAGAAGAACGTTTCACCAAACAAATGAGATCATGGATAATGAGAAGAAGACGCTACACTACACAAGTGTGAGTTGAAACGTTTTTTAGTTCATGGAGTGTTTGTTGTAAGCTCAATAATGCTTTTTGTAAGCTTTTGAACACACAGATATCATTTTGTGTCCGTTTTAAGTTTCTTGTTGCTAATAATAATAATAATTCATTTTGCACATTGATTGATA

>TCONS_00077149

ACTGGTTTTTCTGTTAGTTTACAAGCTTGGAGAATCGAGATGGTTGGGAAAGAGGCGATTCCTGGTTTATCCTATGTGTAAGGGAAGAGGGTGAGGGGCGATCAAGAAGTTAGAGCTTCCAGTGTTTCCTAGCGATCATTTCGCTCTGCTTTTAACTCTCTGCCCATATGAGATTAAACAATCGGAGCTCTAAGTTCCTTTTTCCCCTCTGGAAACTCGTGGTTGGAATACTGATAAGAGGTTTCATGTTTTTTTTCTTCATTGTTTTGAACTGAGCGTCTTATGGCCAACGCCAACACTTGTAATGAAGATTATGAAACTAATCAGAGTATCACTAAACACATGAGATATGTTTTAATATAGCAGGGTCTGGTTTCAGACAGTTACTAAGTTATTCACTGAATCTTAATTTTGAATTTTTAACGTACAAAGCATGTTCTAACAATAAGCTCGATTAATTGTTTGTGTCAACAATGAAGAGTTTGAAGCTTCGAAGATGAGAAAATGATGGTTGAAGCCACTTCTTGCCGTATTACCAACGTCAAGAACCTGGTTATTCTATATGTTTATTTTCAAAGTATGGAAACACGAGAACGATAAGATCCAAGCTCCAATAACTGTGATTCACAGACTACAAGAAGATTAGCTACCTCTCACGAGATATCGTCCTGTCATTCTCTCAGATGCTTTATAATTGCATAAAGGAAGAAGATTATACGGCCCTTCTATTTATATGTATCTATAACCGAAAATCCTCTTAGCAGGTCAGAGTCTTTGCATCACTTTGACCTATCATTACCCCTTCCACAAATTCAGCTTGCCCTCAAGCTGGAAAGTAGGAACTGATGTTGCAATAGCTATGACAACATTCTTTTTTACAAGAAATGTGAGTCATGCTATTTGTCAGTTAGAAATTTCTAGATAAAAAACAGAGCTTTTCTTTGAGAAAAGGAAAAACAGAGCTCGTCAGTTAGAAAGTTTTGATAAAAAACAGATCTTTGAATTTGATGCCTAGGTGATCTGGGACGAGCAAGGCTCGAGCTAAGCATTGTCCATGACCTTATCAGTAAAGAACCTACAACATTGAAAGGCAGTTGAGAGCTGCAGTCCTCAGATGTGAGTAAAAGTCGAGACCAGATATGCATATGAACATTTGTGTATGTTGTTTCGTAAGAGTTAGCCGGTTACGAGCAGTAATGAACCATAGTCCTCAAATGAGAGTAAAACTTTTGAGGGTATTACTTAAGCTTTGGTTCTTCATTTGATTGTCTCTGACTTTCCTATTGCGTTTGGTATAGTCTTACAGTGTTTTGGTATTAAAATGTGCAGGCGTAAGTAGGAATGTCAAAGACTTAGTGGAGAATGGAGAAGATAAGCTTTGTAAGATCTTTTTTTTTTCAGTTTCTCTCAAAAAATGAAAAGAAACTGATCACTGAACTGAAAGTGGATTGTCTTCTGACTTTTAAGCAGTGCGGACATCAATGTGTCAAGCAGTGCCGGGCCTCTGGGGATAGGTGAGTTCCATCTGCGAGATTTGGAAGCGCGATTTTTGTGTGTTCTTCCCTGAGATAGATGAGATTTTCTTGGAAATCGAACTGGTGGAAAGAGTATACTTGTTTTCTCTCAGTAGATGCGACTCAATCACCGTGGCTAATTCTGCATGCTGGTCATCGTCTTCCTCCTCCTCTAGGTCACGCACCAAATGTTCATCCCTCAATTATCCGTCTAAGAAGCTCAATTACATGATCTGCTTGTCTCCATCCCTTGGTCCCTCTCAATCTCCTCGAACCATGAATATAAATGGGCGTGCGAAGGCTGCACCTTTCTGAACACCTACAAGAACTCAGTGTGAGGACGCGATCGCCGTCGTCTTCTCCTCCCCTGATTTGACCGATTCGGGTCTCGAAAGCAAAAACATGAACAATCGGATTGCTCTCCCGGGTGGGTTTTCCTTCCTCCGCGGCGGTTAGCAAGCGCAATGCCATGTTTGACGACGCCATCGAGGTTGATGGTGGTTCGGAGAAGAAGATCAACGAAAG

>TCONS_00057378

GGGTAGAAGAAGCTTCTTCAATTCTTCTTCGCCAGCTTTCGTTCTCCCGGGAAGCGTTTATCTTCGCTTTCTCCGGTTTATTCCGGGTTCTTTCCCGGTTATGTCCGATCAAGGCCTATTTGTGTTTATCTGCTCAATTGACTCTCGAATCTCGCAGATCGTGTTCGATTGATGTCGATTGACTATCCTTGTAGCGGTTTTGATCTAATCTTTTGGTCTGAAAGGTTTCTCCTTTGGATCGGTTACGGTTTGATGAAGCTCGCCGTCGAAGAGTCTCGAGCGGTGAGGGAAGAAGAGTTGGGTTCGATGGCTCTGGAAGAGATCTCGGGATAGTCTCGATGATGCTCTCCGTCGTAAGAGTCCAGGCAAAAATATGGGTGAGATGGTTTCAGTCTCCGGCGTGTTCCGGCGAAACTACTGGTGGTTTCGTCTTCTCCGGCGACGTCTTCTCGGTATCGGACGCAGTGTAGTGACCAGATGACGCGTGTCTCTGGAAGGGGTGGGTCTCAACACGTGGCGTCCATGCGCGTTTCTGGACGCGTGGTGAAGGGTGGCGCCGTTCGTTTAATGGGTTGGGCTAGTCTCGT

>TCONS_00042870

CATGACCGGACCCGGTGGTTGTGGTTTGGTTCTCTGGTGTTGAAGTGAGGTTAGAGAGGAGGGGGTTGAGTCAGGGTCGGCGTTCTAGGGTTTCACGAGTGGGGTTTTCCAGATCTATCCTATCTTATAGGTGACGGCGCGTGGTGGTAGGAGGCCAACTCCTCTCTATGTTCCGGTGGTAGATCTTTAGATCTGGCCTGTACCGTTCTTCGTCGGAGGTGTGGCGTTAGAAGTTTGTCATGTGGTATTGCCTTGGTGTCTTGGTACTGACTCTCTTCTGACGCTGTGAGTGCTTCAGAGTGTTGTTTCCTCATTGGCGTGATGTATGCTGGTGGATTTCCTAGCATGTGTTAAGGTTATTAGTGTTGGCTTGTGGAAATTCACGGATCTGATGTAACGGTGTGGTCGTCTATGGTTATGCTTGGCAGCTCCGTTGTCATCCGGCTCAACTCTCTCTTCTACCCGGTTCTTCTTCCCGTTTCTTCTGTGATGCAGGTGGCTCGCTAATCAGAAGACTGCTCCTTATTTCAGTTTGTGTAGGTTGGTATGTGTCTCCGTTTCAACCTTTGGCATGTGAAAAGATTGGTTCCAGTTCTCATAATCAACTATAGGCGAGGTGCTTAGACCATTTGGGTTGTCTCCAAACTTAAGCTTACCTCAGAGATTCCTTGTGGTGACTGGCACTTCCCAGTGTCAACGGTGGTCCCTAAGACTGTCTCGGCAGTGTTTGGTCACACTCTTCTTGTAGGCCTTGGAGTGTGGTCGGGATATTGTAACAAGCAGTTCATCGAGCCCGTCTTTTTGCTTTTGTTGAAGAGCCTCCAAAGATGCTTTTGATTTCAAGATAGGTACGTCCGTTGTGGGTCTGCGTTGTGGGTGCGTGAGAGGAGGATAGTCGTCGGATTAGTCAACCATGACTCTTCTTCTTGGTGGCGTTGCATACAAAGAAAGTTCGTGTAGGAACATGATATAGGGCTATCTCTTGTCCACTATTGTTATGGTATGGTGGTGCTGTTTTTTATGTCTGCTTGGTGTAGAGTTTTGACGTGTGACAATCTTTGGAGGTTTTTCTTTCTTTCCGAATAAATGCTCTGCTTTGTCGAACGGGTGTGTTTGGCCTTGTGTGTTGCTGTCTGGCCATGTTAGTTTCTGTTTTTGAGGTCTACAGCGAGTTGTTCTCGGTAAACCAAATTTTAAAGAACAATTTATTGGGTACGTCAATTTCTACCGGACTTTGTCTTTCGTAATCATTGTAATTTCTTTTTTTGTGACAAGCGGTTAGTGCTATTGGAGTAGTGTTTTATGGTAAGCAGTTATACTTTTGTGTCAG

>TCONS_00010935

GAAGCATGTTACTCATCACCAATTTCCCTTTTCTTTTTGCTCTTTCAGTTTTTGATATTCAAGAAAGTTAAATAAGCTTAAAGAAGAATGAAAGGCTTTTGGAACTTAACGAGTACCATTTCTCTTACTCTTTCTATACTTTTTTTATTCGGCTACAGTTATGAGGACGTGTCTGCGGTTCCAACTACGCACTTGTGTCGTCCAGATCAAAGGGATGCACTTCTCCAATTAAAGAACGAGTTTGAGGTTCTAAACTCTTCCTTTACTTATTATAATTGTATTGATCGTGGTATTACGCCTCATCGGAAGACAGAGTCATGGGTGAATAACAGCGACTGCTGTAATTGGGAGGGTATCACGTGCAACGTCAAGTCTGGTGAAGTGATCGAGCTAAACCTTAGTTGCAGCAACCTAGGTGGCAAGTTTCATTCTAAAAGCAGTCTTCGAAACCTTCATTCTCTAACTACTCTACTCCTTTCAAATAATTATTTTAGTGGTCCAATCATGTCTT

>TCONS_00021920

GATTCATTCTCTGTAGTTTAGCTCTTTTCTGTGTCTGGAGTTTAGGGTTAGAAGTTCCGCAGGAGCCTCCCGGGCTAGTCTGAAAATAATAATTTGAGGAATTAAGCCCCAAAGCTCCTGCTAGATTGATGACATTCGTGTTGCAGTTCTGTCTAAATTTCTCCTGGCTTAACTGTGTCAAAGCAATGTCTAGATTTCGTATTAGTGATTGTTTTTATTTAGTTTGATAATCAAAGGTGTTTTTGGCTATGATTACAAAATATCCGCGCTTCTGAGATTTATTCTGTGAAGTAATCGGAAAACAATGGTTGAATTTCTTAATATGAGCCTTCTCTTTGATTCATTATTATTTTAGTTTTATTTTCCGGCAGTGATGATTAAAACTAACAGCTTTACGCCAGTTCTGCTTCAGAAGATTCTTGATAGAAGAAGCTGTATAAAATCTGAGCCTTTTTTTTAATTTTATGAATGTTTTGTTTGTGTTGCCGATGAGGAAATCCAATTATGTGAGGTGCAGTGGAGCTGAGACCCTGTTTTCAGGGAGAACCCCTTGGCAGAAGCTGCAATTTCTATGAAGCTAATGGACTAACTGAGGCGTTTTGTCTTTTTAATCTGTTTTAAAATCTATTGATCGATTCATTGTCTGAGTTTAACTATACACTAGCATAAGCCAATGTGCTCGTAGTCTGAATAACTCAGAAATTGATAGTGTTTTACATCAGCTTTATCTCTGCTTCGGCCTTGGGATATTAGTTAAACTAGAACACTATATTATTTATCGAATCAACTATCTTTATGGGTATATTGTGACTATTCGTATTGTTTAGCGCTGGATGGCAATGAGAGTAACATAATTTCGTTACCCAATTTTGTTCTGAGGTTTTTATAAGCGAGATGGAATGCACTATATGGTGTGGAATGGAAAGTATTGATGGTGACCAATATTATAAAGGCCTAAACATGATGTGAATAAACATACAAAGTGTCTTTTGTTGTCCAGTTAAACACATTTTATAGCATAATGAAACCCTTATTCCTGAAGAAACAAAGGAAGCACTAATCTTCTAACCAAAGAAATCAGTACATATTAGTTTTCTAAAATTTAATCTGTTGAG

>TCONS_00025498

AGTTCCTTCTCTTTAGTTTTTTTTTAATCTTCAGTCTCTTGACTATCTGCTGTTTTAAATGTTTCTGTTTAAGGTTTGCATGTCGGATGGATTACTGCTAGTGTTTTGTTCTGTCTTTTCTCTCCACTTTTTGGAATCTAAAATTTTAATTTCGATTAAATGAATCGGAGCAGATGATGATTTTTTTATGCACTACAACGGTGCTTCCAATTAACCATGCGGATTATATAAATCGAGCTTTTTAAAACTGATGCTCCCATTCACCAATCTATAGATTTTTCTTCTTTTGCAATTTCATAAACTTCCGCTTAAAATGTTTGCTAATTGCTTAAATTGATTTATAATGGATTTAATCTCTTGGTTCGGTAGATTGAATTGTTTTCAACCGATGCCAATTAGGAATGTAACATCTTGTGATCCATCTGTTCTTGTACAGAAGAAACGCATAAGAATTAGTAATGGTATGTCTCAAACTAGTGTGAAAGGGAAACCACTGTTGATGTATCATGACTGCTTAGTTCAGATCCGTATTTTGACACTTAATTTATATTAAATCTGGCGCTAGTTAATTTGGAAATCTTGATTGATTTTGATGCTTTTCGATCAAAATGTTTTTGAATTTATATTAATTCCTATAAGTAGATTTGATCTGAACGTATT

>TCONS_00068223

AGCCCCTCTTTCAGTAGCCTCTCTATCCGATAGACTGCCATAGAAATCAGTGTCATCATCCCTTTTGTTGAACTCCACATGTAGAACACTTCTACAATTCTCTTGATTTACTTGCATTAGATAACAAAAAACGTCTTCATCTTCCCAGATATATGATGGCTTGTTCGAATACATTACCATTGGAAAGTAACTAATCTTCGCTTCCATCTTATAGTCATCTACCTTTATCTTTCTGCAAATACGCTCAACCAAAACAGAGCGTGTGATCTCATCCACTGATTTCTTCAACACAATAGTGTGAATTTCATCTTCCCCTTCACAATCTCCCGAAATCCATTTCATTTCAGC

>TCONS_00029510

GCCATGGTTGTTTATGCTCGTTTATGCATGCTTAAGAACACATATTTACATTAACATATAGAATTCCATTATTAGTAGTAAACACGTAACTATTTTACATTATATAAGCTTCATCAACTCAGTTAAAGTACTAGTAGATACGTAACAGACACAACACTTAATTGGTAGTGACCTAAAGCTTATTCTAAGTGAAAGCCTTGACAACAAGCTTTAGCATATGTGTTGGTCTGCATTATTTTCAGGAAGCAGAATTAAAATATCTTTTATATATATATATATCACCATCTTACAAACTCATCCTGTGTCGCTTACTTACGAAACTCCTTTATAAGCACCCCTCCATAGAGTTGGACCGCCTCATCGCAGCTAATAGCCCGCCCTTTTGGCCTCTCAAACCGGTTGGCATAGCCGATAAAAGGACGGGCAG

>TCONS_00008112

GTAGAGTTCTTGATATGCTTGGTGTTGACTATCTTAGGCTACATCCCAGGGATAATCTATGCGCTTTACGTGATCGTGTTCCAGAACCGTGAAGGGGAAACTCAGGATTACAGTGCTCCACTCAACTCAGCTTGAGATTATCATAATCTTTAACAGTTTCTTGTCATCTCTGAGAAAAAATGGCTAGCAATATGGAAGTTTTCTGCGAGATCTTAATAGCGATCCTTCTTCCACCTCTTGGAGTCTGCCTCAAGCGTGGCTGTTGCACT

>TCONS_00056517

GAACATAGGAGCACTTGGTTGCACACAATCTTTCTTGCCGCTGTACAGTGTTGTGGCTGCTTCTCAACTCACCTCCCACCTTAATGTTAACTTGCGGGCTTTCTGCGAGCTGTCTAATGGTACCTTCCCACGCACTTGTCAAGATCGCTAATAGTTGAGTGTTACTTTTCTTAGGATTTCGTCAGATAATATGAGAATTAAGCGGGAATTTTTCTTTATTTGAACATGCTAAAACTACGGATCTTCTTGCGCATTTGATGTCCTTTCTTTTGGTGGTTCTGTCTGTTTGTGTGAATTTTCAATTAAAAGAATTGATTGGGATTGATAAAGAATTGAAATGGTGGGTGATGCAAGTGGATGGGCTACCTATTATCATTGCGAATTCGAGTTTCCTGTAAACCCTAGCAAAACCTTGTGAGCTCTCTCTATTCACATACGGTGAGCAAAAATGGCTTGGCGCAATGCAGGATCTACCGCTCGTTCTTTTGTATCCGCCGCCGCAAGAGCACCGTCTCTCCGGTCTCCTACGGCGGCGCTTCCTCGCCTCCGTCCTTCCCCATCCTCCTTACCTGGCCGTCGCTCCTCCTTTTCATTGCCCTCTAG

>TCONS_00024672

CTGTTGGGGTCAAAATCGGTCACGACGGAATCAATGCCTGAAAGTCCGTAAAAATCAGCATGAACGTTTTTACGAAAAAGTAATCTTCGTAAAGATATCTTTACAAAGAGCCTTGCGGTAAAATCTTGTTCAAATCTCAATCGAACCACTAAATACCGATTGTCCGAAGGAAACAGACATGAATCCAATTCGGCCGCGGACAAGCTCGAGTACGGCAATCGGAC

>TCONS_00037572

CGTCTTTTGGCTCAGTTCCTCTCCTTTTCTCTCTTGTTTCAGATTTCTTTAGCTGAGATTGAAAGATTCTTTATATCCGAGTGATGCAGAGATCAATTATTGACAAGAAGAACCAATGTCTTCTGACATAAGCTAAACTCTCTTCTTACAACCTCTTTCTCTCTCTCTCTCTCTCTCTCTGCTTCCTTTTCCTCTCTCTTCTCCTTGTAAAGTTTTGAACTTTAGTTCGTGAACTAGAAATGGCGGCAGCTCAAGGCAACAGATCTATCTCTCTGGTTCTGTTTCCAACTGTTTGAAAGTCTGGTTCTTTGGACAGAGGCTAGGGTTGTTAAATTAGATTCCTTTTGGGCTTTTTGGGGGAAAAGGAATGGGTAAGAGGCAAATCTCACAGGATGAAGTAGGACCGCCGATAAATCCAAGAGCTGGATTACGAAGAGAACAAGCTGGAAGAGGTTCATACAGGGGTAGTTAGTCACTTTGTAGCTTACCCGAAGGTCAAAAAGGT

>TCONS_00038221

CATCCATCAATTGGTTGTACCGTCATCGTATCCGCTATCTCCTATCGAACAGCAAGCAAGTATTCAACACCTCGACTATGTTGAGTTGGGAGACTCAAAGCATCTTCTCTCCTTTTCCAAAACCACTTTCCTAGCTTCTCAATTTTGAACTCCAACAGGAACTGAACCGGAAATCCTCTAGCTCGCTTCAGAGCGGAATTAATAGATTCAGCGATGTTGCTTGTTTTTATGTTGTACTTGTCCCCTTGACAATAAACCCTTGACCACAGGCTGACGTCGGCATTCTCCAAATAAGTTGCAAGGTCCGGGTTTGCACTCTGTATCTCAGCCATGTACCGGTCAAAATTTGAAACCGTATGAGCATAAGCAGCACCTTTCACCAAGTACAAGAGATGTTTCCCTTTATACTTTTTGACAATGTTATCTTGAAGGTGATAATAACATATTCCTCGGGTTGCCCAAGGAAACACCTTATCACACGCACTTTTAATGGCAGCGTGCCGGTCGGAGACTATCACCAAAGGCTGCTCATTAGATACACAACTAGCCAATTTTGTGAAAAACCATTCCCAAGAAGGTTCATCTTCAGCATCTACGATCCCAAAAGCCAATGGAAATATCTGAAAATTCCCATCTTGTGCGGCTGCAACTAACATAACACCTCCATACTTCCCACTTAGGTGAGTCCCATCCACCACAATGACCCTTCTACGATACTTAAAATCTTTGATAGAAGCTCCAAAAGAGAAAAATAGATACTTAAATCTATTGATAGAATCAAGTTCTATAGCCGTGATAGAATCGGGATTTGCTAAGGAGATTTGCTCGAGATATGATGGCAGACGCGAATACCCATCTTCTGTTGATCCTCTTACCAATGTTTGTGCATATAAAAGTGCTCTGTATGAAGTGGTGTAATCAAGCGTCATGCCAAACATGTTCTTCATTGCATCAGTGATATGCTGCGGATTCAACCCATCAATGATTCCAACACGATCAATGAAAAGCCTACCAACATACTTCGGAGTACAGTGTCTCCGCTGAGCGAGTCGGTCTCCCACTGAGCATGTATGTTTTTCCACATACTTTGTTACCCAAAACGTGTTTGTCCCATGTTTCACACTCGCTCTGACCTTCCATTGACAACCACTAATCGGACATGTTGTCACAAGGAGAGTTTTCGTTGACTTGTATATTCTGAAGGAGAACTTAAACCTCACTGTTGTCAACCGCA

>TCONS_00060642

CTCACATTAATACATATTATAAGTTGTATCAAAGCTTGTAAATAACCCCCTCTTCCGTTGTACAGTCAGTTCAATTCGTCAGTCAATTATAATTTTATTTTGCGATATACCGGCAAATTTCCCTAAAAATATCAGAGAATTGTACTTGGTTTATCATCACTAGCATGCGAACATATCATATTGGGCTTGTTAAAAGGATGTTGTTAAAAAGCTCACATGTTATTCACAGTATGTATAGAAGCCCAAATAGTCAAATACCAATACATGGAATCTTCTTTCATGTTACCGAGATTCCCAGAAAAGACAAGCACTCTACAAATCCAAAAGTAGAACTGTAAAACAATAAACTAAAGTCCTAAAATCGCATCACATTCTACTTGCTTCCTCTAAAATTAGATATTCTAATTCTGGGTTGGTCCGGTTGATTATTACGACTTTGATTGAGTCAGAAAACCAGAGAACGAGCTAGTCGGAAATAGAGTGGTCATGCCAGCAAAGGAGCATGGAAACACAGCGTCGGAGGATGTAGAGGCGAGTTCGTTGTTGCTTAGCCATCAGCAGGCACCGATCTCCGAAGCTGGTTTTCCGGTTCGGTTTACTCGAAGAAGGATTGGACATATACAGACAAGTTTCAAGGGAGCAATATCTATAAAATTTTGTTTTAGTTGATGTGAAAAAGCAAG

>TCONS_00023033

CTGCACAAGGCGCAGATCTTAACCTAAGTATGTATATCTTTAATAATTTTAAATCTTAAACATTTTCTAAATCTCAGGCAAAAACAAAATAATGAAATGAGAATAAACTCAAAGATCAATATTTATATCGAGCAATAACCAAAATGAAAAAACAACACAAAAATGAGAAAAAATATTGAAGATAGATAGAATTGGCACGTGAACGTAAACTTCTCATAACAATAATTAACTTTAAGTTGCTAAATCATTTATATACTAAAGCACAAGTCACTTCTCCACTAAAATAATGACACATGACTATTGTTTTACAAAAAAAAACTAAAAAAATCATCACGTAATTTCCAAAATTTGCAACTTCAAATATAACTAAACTCCTATATTTTCTCAAAAGACCTCATCCCACGTTCAAAATTAGTTTTCACCACTTCAAACAATTTTTTCCTACATAATTTTTGATCTACGAATCTGTTTCACGTCATATATAACTTCTCAAATCATGATATGACTTCAACCTGTTTCATCTTCTACTATATTATTTTCTATAACTGGTTTTTTTCTCTCTTCTTCTCCATCGTTTCATATTCAGTTTTCCCAGCTTTTCTGTATATAAACAAAAATCAGTACAGGTCTGTGACTATTCTATTCATATTATTTTTATATTCTGCTCAAACTAATATCATTTGATTTGTTTAACATGATTCAGATAGAAGATGTAACTTGAGTATTATGTTTTTTCAGAGCTTAATTCTTCAAAAGGACAGGGAAACCAGAGCAATATTGCAAATGAGGTAAAAATAAGTACATTCTCTCTATATAAAGAATTGGCTATTTAAAATTAGTTTTTATGGCTTATCTTTAGAAAGGTATATTCTTCAATCAAAAAGTTCTAATTTATTAGAAGTTTTGCATGTTTTGGTGATGCTTCTATATAGAAAAGGAGTGAACACTTGATTAGTGGACCATTAACACAAGTATTATGAAAAATATGAAGAGCAAATAAAAACAAAAACAAAAAGATAAAACGATGTGCGTACAAGTGAGTGAATTCTTCTAACACAACACTAGACTAGAGAATCCACATTATTTTACTGGATCAGATTATAACAACATTATATCAGGTAGGAACATTTTTGAGGATCTCATCAGTTACCTCATTGGATCTTCGACCAATATTATTGTATATATTAGAGTTTCATAAGATATATATTAATTTTTGTGCTCGTACGTAGTACGAGACAGAACACTAGTCATGATATAAAACCGAACTGGCATAGTTTCATTGTAACCAAATAATAGGTCTGAGATTCTTCGGCCTAAACGTTAACTTCTTATGCGATAAGTGATTTTTTTGACCTAAAATATTTTAAAAAATGAGATCAGCTCATATGTAAACAAATTATGGAATTTACAAAAAAAAAATTAAAAAAATTGTTTTATGGCCACAAATATTAATTCGATCAAGAATATAAATTTTATTTTTACATACGATATTTCTTAAATAACTTTCATATAAATTCACCCTAGTTGTACTAATAAAAATATAACATATCATTTAGAAACGTACGAAACAATTTTATTTTACTTTGGTTGTTCAGTTGGAAAAAGGGAAAAAAAGAGCTGAAAAGTGAAAACTATAAAGTGGGATTTATTCTGAAAAAAAAAAAAGATTGTAAGTTGGTGAAACTAAACAATTTTTTAGAGAAAAAAAGGGTAAGTGTTATTGGGGTTATCCTGTGAGTCATATGTTGATATATCAAGCGACGAAGATACATAAATCTGGGATGCATAAAAATATTTTAATAGCAATTGTTAGGAAGATGACACGTTGTGTAAAATCAATTAAAGAAAATTTGTTATAGATTGTTCAAGTAGTTTTAATAAAGCAAAATATGTTTTCTTATACTCAATACTGTATTATTTTATTTATTTTCTATTTTACTACTTTATTGTCTTGCGTCTTACCCTCGCTGAAAATACACAAGATGGCTGGTATTTCCTCAAATTATATTTATTGTAATACAGAACCAATATCAGCTCAAAAGGAAGACAAAGGAGAACTCCATTCTGAAAGAATATTCCTTGATATTATTATTCTAGTTTATAAAGTCAATTACGCTAGAATAGATCTCCTGTAATAAAGGTAAAGATCTCTCAACTACTCTGTCCGTATAAGCTTTTGTATATCGTTTGTTATGATTAAGCAAAAACACAGACTTTCACATTCCTCCAGTCGAATACCTAAAAGTGTCTGACTCGGAGAAGCTTATAAAAAAGGCAGCTATAAAACGGACGTGCAAGTAAGAAAACTAGATCCGCTGCAAAGACAATTTCATCACCAACCTTAAACTGCCAAGACAAAGCCACCTAGAAACCACGTCTAGTTGCCCTGTTCCACTAGAGGAAGAGTTGCCCTGTTGGTCTAGGCCAGAGATCGATTCCCCTCTAGTGCAAAATTATTAATTCCACATATGGATATACGGATATAGACTCATGCTTACGGCCCATTTGAATATCCGAGAGAAGATCCATCCGTGGATTGTACCTTTCACCCGGAAATTAGATTTGTGTCTTTAATAGACTCGGGTTCAACCCTTTAGTTCTCAAAAAAAAAAAAGACAAAGCCACCTAGAGCCCGATTGGTAAGATTTTCGCTTTAAATTTTACCTTTAGATTTTTTGTCGTTAGATATCTTAGCATTAACTGTGAATAATTTTTTTAGACTGTGAAGGTCAAAACATATTTATAAAACTAACCTAAAATTATTAAAAATAAAAATCCTTAATATAAAATAAATTTTCAATAACTTATGATTAATATAAAAGTTAAATATTTATTTAATTGTCTTAATTTAAAATAAATGAATGATTCATTTTATTACAG

>TCONS_00013639

TTTGTTAATCCATCTCACGAGGCTCTGCAGAAGGTGATGTTGACTTTGATTTTTTGTCGTATGATTGAAACAAAGTTTCCAGCTTCAGACGCAAGTTCTTCATTTTCGCATCACGTGTACTCATGTCAAGTCTCCCTAAACAATACTCTACAACTGGAAGCTTCAAGCGTGGATCAAAGACAGCTGTCATTGCAAAAAGATCACTGACTTCTTCCCAGTACTTATCGAACTTCTCTTTCATCGGTATCACCATCTCTCGGATAATCTCATCATCGCTTTCCTCATTTATCTGCAGCCAATTATGGATCTTCCATACCTGATAGAAATACAAGTTCGATGTTGGGTATTTCGAACCTGACATCAAACTAGTAATCTTAGCAAAAGGCTCCAAGAAAACACATATTTTCGCAGCTCTGCTCCATTCTGTAGCTGTAGGCAAAAACTTATAGTTTCTCCTATCATATATCTCCAACTTAACAAACGCTTCACGGTACGGAAGGGCTCTTTCAAGCATATAGAATGTCGAGTTCCATCTTGTCTGCACATCCATTATCAAACCAGCATTCTCCTTTATACCAGCAGCATCCACGCATTTTGCAAACGTCATCTCACGCGTCTCTGACGCTGTTACATACTTAACACTCTCTCTAACTTTGTGCAAAGAGTCTTTAATAACCTTCAAACCATCTTGCACAATGAGGTTGAGTATGTGAGCTGCACATCTGACGTGAAAAAACTCACCTCCACACAACAAATCATTGCTCAAAAGCACCTGAGTTTTCACTATATCTTGCATCGAATCATTACTTGTAGCATTGTCTAATGTGAGAGAGAACATTTTTTTCTCTAGTCCCCACTCTTCCACAGATTCAAGAAGCTTTAAAGCAACATTCATACCTGTGTGAGGCGGTGGGAGAGCACAAAAAGTCAGTATTTTACTGTTCAG

>TCONS_00061663

GTAACAACGTGGTGAATGGTAACTTAACAAAATCACACTTTCTGCTGAAAAGTAATAAACATGGCGTTCCAATAAAGGTACAAAAGGAATGGATACAGATGAGGAGAAGAATAGTACGTGTAACTTCTCGACCAATGGGATATACAGTTTTACCCAAATGCAGTAGGGTGGATGGGTACAGAGTCACGGCTTTGTTTTCTCTGATGATTGGGGACTCCGTGATTTGCATCTTCATCTTTGCTCTCCATCTCTG

>TCONS_00082236

ATCAGACCTAATGGCTTTAATCTTTGCATTGTATTGAGTTGAAACATGTGTAAGAAACAAAGGAAATACAATGGAAACTTCACTTTTATTTCTTAACAAATATATCCAAGTGACTCTTGTACAATCATCAACCAAAGTAAGAAAGTACTTATATCCTTCTACAGACTCAACATGAAAAGGACCCCAAACATCCAAATGAACTAAATCAAAAGGACTAGACGCAAGGTTATTGTGTGAGACATACGCCAAACGCCGTTGTTTTGCCAAAGGACACACCATACAAGGAGTAGATGCAGAAGATACATCCTTAAAAGCTGGTAAAGACGAAGAAATCTTTTTAAAAACGCTTGAAGATGGATGTCCCAATCGTTGATGCCAAATTGAACTATGAGTAGAAACCGAACCACAGAAAAAAGTGTTGCTTGAAGAACAAGTACGCGGTGTATCAAGAATGTAAAGATTGTTGTACATACTACCCTTCCCAATCATCAAGCCCCGAGAAAGTTCCTGAATCAAGCATCCATTAGGAAAGAAATGAGCAGCACAAAACAAAGACTTTATCAAGCTACTCACACTGATCAAGTTAAAGCGAAAATCAGGTACAAGCAAAACATCATATAAGATCAAAGAATCAGTAAGCTTGATAATACCAATATGTGTAATAGGAACTCGACTACCATTAGGGAGAGTAACTGTAACAGAGTTAACCGGTACAAAGCTATCAAACATTGTTAAGTCAGAACAGACATGACTAGAGGCTCCACTATCAATGATCCAAGCATCATTAGGCAAAAAAGATTGAAGTGTAGAAAGACAGTGATCTCTATAATGAAGAGATTGGTTTTGAAAAGTAAGGTTTATGGAAGGAAAATGAAATGTACCAGACGAGGAAGAAGGGTCCATGACTCCATGTTCAGTAATCGTAGCATGGGAAGTTGATGCATATGATGGAGGAACTCGTGGCTCTGAAACCCGGACATGAGCATTGAACTGAGTGATTAAACTTTGAATTTGTTGTGGCGTGAAGCTCTGTAGATTCAAGTCCATGCCTCCGCTA

>TCONS_00037469

TATCAAAGTCCGTTGGGTTTGTAGTACTAAAAAGACCATCAAAATAATCTACCGCCACCTTCTCAATCCCATTTTCCTCTGTAATCCAATTTCCGTGTTCATCATGGAGTCCCACTATTTTATTACGGACCCTACGCTGCTTAGTCAGAGCATGGTAAAATTTGGTATTAAGATCTCCAGATGAATGCCACATATTCCGGCTTTTCTGATGCCAATATTCCTCTTCATCTTTATACGCCTCTTGTAACTTTCTGGATACCTCAAGGATGTCCTCTTGTGATCTACTATTATCTGACTGGACCTCCTCGAGTGCATGTTGAAGATCTTTAATTTTATCCTTTCCGTATGGTTGATTATCCTTTCGCCATGATGAAATTTCATGACGACAATTACTAATTTTTGTAACAACATCCTCCAGCTGTCCTTCATGATTTTCTGTCCATCCTGCCACAATTGATTCCATAAGCCCCTCTTGGCCAATCCATCTCTTATCAAACCTGAACTGACCTCTTTTTTTCCTAAATACCTTATCCTCCAAAAAAGCTATCACAGGGCGATGATCAGAGCCCACCATCCTCAAATATTCTGTGTAAGAACATGGGAACAGTGTATGCCACTCCTCATTTGCCAAGGCCCTATCTAATCGACACCTGATCGTCATTGCTCCTTTTCCTTTACCTCTCTTTGCTTGCCATGACCATTTATTACCGCGAGCCGGAAATTCTAGTAGACCACTGTTCCTTATCATATTGTTAAAGGGTATGAATGAGGTGGCACTTCTCAGGGATCCCCCATCCTTTTCATGATTTCCAGTTATCTCGTTTAGATCTCCAATAATAAACCGTGGTTCAGAGCGTGATAATCCATACCGAGTTAGTCTCTCCCAGACCTGTTCTCGTAACTTTTGCACCGGGTCTCCATAAACAAAAGTAAGAAAAACTTGTTTTCCTTTGACCACCGCCTCAATATCTATCATTCTGTTGCTAGAATATAAGATTTGAACTTGATATTCTTTATTGTAATAAAGAGCTAAACCACCACTTCTCC

>TCONS_00013980

GAGAAAGCAAAGAATGAGAGATCGAGTTTCGATAGTTTTCGAGCGATTTAGGCAGTTTTCGAGAACAGTTACTCTGCCGATTTTGAGTCAGCACTTGGAGAAGATTCTGTTCAAGTGAAGAGAGATCAGTTCTAGTGGAGACCAGTTCAAGTGAAGATCAGTCCAGACGTGGGTCTACTTGTGAAGGTCGGGAAAGGGTTCGGATCACAGAAGTCGGGTTTGGGGTCAAGGCCACGGTCAACCAAAAACTGTGAGTTATAATCAATTGATTGCTGAGTTGTTTTTATGCAGGGTCCCGTTACTTGGAAGTTGGATCATGGCAGGAGGCCAAGTCTAACTTAGTAACGGTTTGCTTAGCCTTAGCTTTTATTGCATGCTAGGGCTAGATTGATTGTTATTTTGGGTTGTCTGGGTAGAGTCTAGGTTGCGGGTAGAGGCCGCCAGCTCACTGAGTAATACTAGATTACTCATCCAACTCCGTTGTCCTTTTTGCAGGTAGCTT

>TCONS_00056043

CATGTCCACTAAATCTGAGCAGTCCGTCTCGAACCGTATCGAGGTTATTCCTCTGTCTCGCATACATGAAGCTGCCCAAAGTAAACCTTCCATCTCAGCATGCAAAGCTGAGAGGCTCCGGTTACACGCTCGTAATCCGCAATACTCCGAGCCCATTTCATCCCTAAGACTCCACCCTAAGCCACTAACACTGCCATTATTGATCCACGATGCATCAATTTGGCAAGTAGGAATTTGGGGTATCCAAGGGGGCACTGTCTCAACCTCTGTAGTAGGGGGATCGTCATGATCCTCGTCTGCTTCCTCCTTTTCGTTAGCCTTCCTCCAACATTCTGCCTCAAGAGACGCATGTTGGAGGGTGTCAATTGGAGATGCGTCTTTCCCATTGAAGAGTTTGTCGTTTCTCGCCTTCCAAATGTACCAACAGATCCATGGAAAGGTATCGAATTGTGGTCTCAGAGGAGCCACCTCTTTTCTCTTCCAAAACAGGAAATTCATGTTTTGATATATGGATGTACTCGGGAAGTAACCCGGAATGGACGGGTAGTCCGATAAAGCCCAAATCTGAAGAGCTGGTGGGCATTCAAAAAGAAGATGATTAATCGACTCCACTGGGCCAGCACATCTAGGACAACTCCTATCGGTGCCCAGGTGTCTGTACATGAGTCTCTCTGCCGTTGCTACACAGCCCGAGATAGCCTGCCACAAAAAATGTTTCATCTTACTCGGGGCCTTTATCTTCCAAGCATGGCTTTGAAGGCTCGTGTAACTTGGTTCTACAGCCCCTTCCTGTGAAGGACTCAACTTGCTCGATCGAAGTAGATCATAGCCGGTTTTAACTGAATAAATTCCAGATTTTGTATGGTTCCAAACATATCGATCCGGAGCACGAGAGAGAGAAGGTTTCAGCCCAAGTATCAAAGGGATATCGTCTGGGTGAAAGAAATCCCGTAGAAGTTGTATATCCCACTCTTTGGTATCATTCCTAATGAAGGACTGAACAAGGAGTTGGGGTAGCATGTATACAATGTGATCAGCGGGCCTAGGTGGTCGAGCCACTACATCTGGAACCCAAGACTCACTCCAAACCCTTGTATCCTGGCCCGTGCCAATTGTTTTCCGTAAACCCGATATGAGTAGAGGTTTTGCTGCCATGATACTACGCCATCCATATGATGGTGAGTATGTACGCCGATCTTCTAATGGAGAAGTGTGATTATAGTACCGTCCTTTCAAAACACGTGCTAATAAGGAGTCCGGAAAATGAATTAGTCTCCACAATTGTTTTGCAAGAAGGGCGATATTGAAGTCATGGAGATCTCGAAATCCTAGTCCCCCCAAATCTTTGGGGGTACAGATCTCGTCCCATGCAATCCAATGTA

>TCONS_00050508

ACTTATGCCACCTATTCCCTCCTGCACCAGGTTAGTTTGTAACCAAACATTACTCCAACTGTTTATATTCAAAGTTTTGAGCTTTTACAGCTCTATATTTTTAGGTTTAGTTACCTAATTAATTGATAACAAAGCAGCTGTGAGAGTCAGCAACAATTTAGTGGCCTCTAATAATCTGAAGCTTACCCATTGCGAGGTTTCTTCGAAGTGTGCACACCATCGGCCTCTGGAAACTCTTCAGGAGACAGGGATATTGTTTGGTTCGATATTTCCTCCTGGGTGGCCTTCCACCATGACATGATTACTGGGGCCGCCGCTGTTGACAACTAACAGCTTTCAAATCCTTGGGATGAATCTTTTTTCACAGCTCCAACTCATGGCATTTCATCGTCGGGTTATCATCACCTCCATGTGGAGAAGTAATGGGAAACATAAGGCATCAATGCAAGGAGTGAGGAAACGTAAGTATGAATGGAGTGAGCTTACTTGTGCTGTGAATTTCGGGAGAGGTGCATGTTCGTTGCGATCAAAAATGCGGATACGGTGAATGACTGATGCTTTGCTGGGAAATTAAATTCTGAAAGCTTCAGCTGGGGTGTTAAGTTCCTCCCTACTATATCTATGAGGCACAGTGAACATAAAAAAACCACGCTGAAGTTTCCGGGAGCGTTTCGAACATGAGATGTAGCACCACGCGTTTGATGTTTCAATGTTGTCAATTTCCAGAGTGCATAGGAACTCTGCAGCCTGCAAGTGTTTGGGTTTGTAACAGGGCAGTTGCAGAGTAAGCTGGAGGTTTTAAGTTGGATGCTAACCTCTGGTGGTGAAGTGATTACATAAGCATTCAATTCCGACAGTGTTAAAGATTTGATCTTCTTAACCCTTCCATATTGTATAGCAGTAGAGGCCGACCCATCTCACTCCATTGGATACTGGAAAATGCCGCGTATAAGTACAATAGATACTTTAAAACTGAAAAAACATACCGCAAGGCAAACAACTCAAAC

>TCONS_00052688

ACCGTTCCAGCAAGAGCGTACGCAACAACCAAAGGAGGAGATGCAAGGTAGTTGGCTCTTGTTAGTGGATGAACACGACCCTCAAAGTTCCTGTTTTCGGAAAGCACAGCAGCCGCCACAATGTCATTTTCTGTGATAGCAACTCCCACTGATTCATTAGTTTCTCCAGAGTTACCAATGCATGTAGTGCAGCCATAGCCGACAATATTGAAACCCTACTGATTCAGATATTCTTGCAGTCCAC

>TCONS_00054061

CTAAATCTACTTTAGGCAACCGGCGGTTTTGCTTTGCCGTTGGCTCCGCCTCCGTTTCTCTGCTCTCTTCTTCTCTTCGCCTCAAGCTTCTCTGTCTATCTCTCTGCTGATATGCTCGCCGTTCCGAGGCTTGACCATTACTGCAGATCTGTTGGTTCTGTTCTTGGAAGTGCGGCGAGGCTTGGATACTCGCGGGAATAAGCTTTTCTCGGTCGGGTGTAATGGCATGGGGTCCAAATCTTTGCGTCGATTGTGAGGCTCTCTCTCTTCTACGCCATCCATCCTCTTTCTCGCTTGGGGTGTGAGCAGATCGTGGGCCTGTTCCGGTGTGACGCTTCGAAGTTCTGGTCTGAGGGAGCGTTTCTTCGACCGTGCCTCTTGTTTCTCGCTTGGTTCTTTTAGGTCCGTCGGTGGCTCCACGGTGGTCTGTCTGGTGAAAACATCATCTCCAACAGAGCAGTACGTCCGCTTCTCCTGCGTCAAGGCACATGGGACTAAGGATCTGTGGCTTTGTCATCCTGGGTCTTCCGTTCTGTGGCGGCGAGTGCGGTTCTGGCCTGCTTCAGGAGCCTGATCCAAGGCATGCTTCTGACTGCTCTTTCGTGGAGTGATGCAGACTATGTCCGTTTGTTTAGTTTCCGGTGCGGTGGTGTGGCACTTCTCAACGGTTCCTCTTTCATGAATGATGGCTCTGCCTTGTCTTGTGGGTGTGCTTGACCTCGGGTTTTACCATATGTCTGGGTTGCTGCAGATTAGGAGGTCGTCTACAAGTTGTTCTGGTTCAACCGTATCCTTAAGAGCGGATCATCGGGCATGCCAAATTCTACTGGGTTTTGTCTCTCGTATTCACTGGGTTCCTCCTTTTGCGGCAAGTAGGTTGTTCCAATTAGTCGAGGCGTCCGGTTAGCGGTATTCTTGTTGCGACAGTACCTATTTTGCCTCGGCTGAGTTTTTGTTTAGATTGCATTTAAAATCCATATGCCGCTTGTTTTCTATGTACTTCTGT

>TCONS_00019973

GGGTGACCATTCGGGTTCGGGTCGGTTCTAATCGGGTTTCGGTTTTTCGGGTTTACAAAAATCAGCCCCAATCGGGTATTATAAAAGTTCGGTTCGGTTTCGGTTCGGGTTTTGTCGGGTTCGGGTCGGATTCTGTAGCACATCCTAAACCCGGTTAAACCCATTTACTTTCCGGGTTCGGTTCTAAACCGGGTTTCGGTTATCCAAACTACTAATACTACCCGAAATACCTATCCCTACCCGAAAGAATGAACCTGTTATCTTGTTTCATGAAACACTTATAGACATAAACTAAAACACACTTCAAAAAATCAATCAAAGCATAGCATGCATTACACAAACACAAGTAATGGTGACGAAGAGTCTATAAGTGTTTCATCTTTAAGCAACACAAGTTATTTACACAACTATATCCAAATGGGGACGAAGACTAGAATAAATAATCAACAGAACAGTTACAATGGTGAAAATAAAACATATAAGAACCTTTATTAAACTTCAAAAGAAAGCAAACTTGTTCATAATGAACCTTAAAAGCAACACTCGAAACAGAGTACATCTCTAATGAGATCAAAGTTGAAATATAAAGCAAAGCCAACACCAAAACAGAAACAAAAAGACTTCCAAGTTCCAAAGCAATCTTCAGTCTTCAATCCTCACAGTCCCAAACTCTGAAGCGACAAACAAAAGCTTGGTTAAGTACACCAACAATCAAATGAAATGGTAAGAGAAACCAAGATAAATAATGCATACCTTTCTCAAGCTTGTCTAGCTACTCAATTTCAGCTAAAATCTGCTCATTTGTTTGGACTTTCTCAGAGAACTTAATATCAGCTTTCAGCCACTGCTCACTGCACATCAGAACCTCGATCAAGTAATGGGTTAAACAGCTCCTATATGGTTCCAAGATTCTGCCACTTGTGCTAAAAGCACTT

>TCONS_00004448

AACCAAGGCAAAGTGGTAGTGACTCAGCACGAAAACGCGTCGCACCAGGGTTCGAACAACATATTGGTACCCGATTGAGAAGGATAGATTAAATATCTCCCTCTCGGCCTCATGAGAATGTTTCGTGAGAGTTAGTTTGCCTAACTAATACAGCAGCAAATAGTCTGCTATGCCAGATCCTTGTCTAAGAGATCTGATGATCGATGTCGGGATGGTCCATCCGGAGACGTGCTTAATCTGAGATGGCGATTTGAGGTA

>TCONS_00052340

TTTTATTCCATCAATGACTGCCTCAATGTCAATCATTCTGTTATTCGAAAATAAAACATTAACTTGAAAATCATCCATAAAAAATAAAGCTAAACCTCCGCTGAGTCCAAGTGGATCAACGGTAAACAACTGATCAAATCCTAAATCGGCTTGAATGTTTTGCAACATCTGCCTCCTATTCTTCGTTTCAGTAAGGAACACAAGTCCTGGGCGATGCTTCTGACACATCTCCTTTAGGCGTCGAACTGTGAGGTCGTTCCCAATCCCTCGACAGCTCCAACTGAGTGTCTTCATTGATGGGTGGGGGATGGGTTCTTGAACCCCATCGAACCCTCATTTTTGGATAACCCAGATTTTTGTTCCTTCGACCTGAGATTATGATGTCTTGATCTCCCCTTTTTTCCAACAGTGCGGGTATCATGAGACGAAGATGAACGCTTGTGTGGAGATCCCCGACGGAGAATCCCAAATTTCTTATTCTGAATATCCAAAGGAGCACTCATCTTGGAGCCATGCTTGCTGCCTCTCAGAGCTCTACCAGCCGACTTGGTATGTGCTGTTTTCTCCTCCATCTCCGCCAGCTCTAACCCCATCAGATCGTCATTCTGGACGTCGCAATCCATCAGTCCACCATCTTGTTTGTCCGCAATATCCATATCAGTTAGCGCGTCGATGATCCGGTCCTCTCCATCACCCATCTCCGGCTCCTTATCACTCAGTGATTCAAAAGATAAAACCCGGGGTGATACTTTGGGGCGTTTTGTGACGTTCTCTTCTCTCTCGCGGTCAATCCGGGACGGTGTAACAATAGTACTAGCTAATCGCCGGTTACCATGAGTTTCTTCACTGATCTTGCTCTCGGAGCTCTTGCAATCAGGAAGTGTTAAAGCCTTACTGTGTGTAGATGTATGTCCATAAGAGACACTATCTTTCCCAACAGGTACAGCATCCAAGCCCTGACGCACAACAACCTCTGTTCCTTGGCGAGGCTGCACCCTCTTCTCCCTCCATGCTACACCACTGTTTCCATCACGTCTGTAGCGGTCATAAGGCCCGGTACCAACCCGCGAACCCCCATAGCGACTGCTCCGTGTGTGATCATCCCGGCGCCTAATGATCCTATCAACATGACCGCTGTTGCCTTCCCGTGAATAAGCAACAAGACTGCTATCACCCCGGCTGCTCCTGTCACTGTTGCCATATCTTGAATCATTGAACTGGCGCACCTTGTGCTCACTGTATGGGTGAGCAGTTTGTGTTCTCAACACAGGCTCCTTCTTGACAGGCTTGTACCTCGACCGTGCGTCTGGCACCTGTACCCTTGCAAAGACACCAGGGCGGTTACCTGGCAGATTAATCCTAGCCTGAGGGGTCAGAGAGGGACAGTACTCCTTCTCGTGGGAAAGCAATCCACAAGTAGAACAATGCTTAAATAACATCTCGTATTTGATCTCAATTGTAACCTCATCCCCTTCCGGAGATTCAGCCTTCCTTGCGAACTTCAGCGGCCGACGAGTATCAATATCAATGAGCATCCTCCCTTCCATTAACTCAATTTGGTCCTGATGAACATGTCCCAACCGAGCTCCTATCCCCTTCAGATTATTAACCGTCCACAGGTGTAGTGGAACACCAATCAATCGGGTCCAGAAAGGGATTATCCAAGGGTAATCATCATGGACAATAGGTTC

>TCONS_00070427

GCAGGGATAGTAATAAGATGGTGGAAAGGATTGAAAGGTTAGAAGCGGCGAAGAAGTATGTGGATAAGAGACAAATGAACCTGAGGGGATCATGATGTTCTTCGTTATATTATAGTTTTCAATCTGAATTTCATATCCGCCGCTCCCTTTATCTTTGCTTCTTACCACCGCTTTTTTTGTTTTCGTTCTTCTCCGCCACCGACCTTGTGATGATCATATCTGAAGGTCCCAGAAAAAT

>TCONS_00030623

ATAAAAGATGGGATTCCATGCTTGGTTCCAAAGGATGGCAAAATACTTGACGAGGATGCTGATGCATCCAAAGCTTCTTGATTGTTGCACCGTATCTGCAGCCAGAGGCTTCTTAATGGTTCCTTCTTTCCTATGTACAAGCTCTGAGTAGCAGTTTTAAAGTTTAAAGTTTTAGTGAGAAACTATTGACTCATGATTTAAAGTTAATCATCTAATAAGACATGATTTAAAAGTATTGGTTCTGCGAGAAGACGAAGTCTCTGGTCAGCGACACGATCGGTGTCTCATTTCCTGCGTTTTACACCGGTCGTGAGAGAGAGAGAGAGAGCTAAAGATTGAAGCTGCTCGAACATGGTGAGAATAAGCAGAGTGATGCTTAAAGAGGCTGGAAATGCAATCGACAAGACTCTGTCGGAGATTCTCGTGTGCCCTCTCTCCAAGCAACCCTTGAG

>TCONS_00014010

CGACTGGTTTCAGAACACTCTGTTTGTCCTTTTGCTCTAAGCCCATCTCACTACCTTGTGTCTTCTGTTGGGCTATGATACTGAGCAAAGCCTCTGTCTTTGGCTCGTTATCGCTTACACACCCCCTCAAACTGGTGGCAGGAGGTTCTGAGACACCAAGTTTGGACCGGAGATTGAGAAAAGGACGTTGTGGTAGTGACTTCGTAAAGATATCTGCGAGCTGATGTGATGCCGGTATCTGTTGTACCACGAGAGCTCCAAGCGCAACACGTTCACGCACATAGTGAAAGTCAGTATCAAAATGCTTTGAGCGGTTGTGAAGGGCCGGGTTGGCTGTGAGGTAGACCGCAGACAGATTGTCACAGTAGAGTCTTGGAGTGACAGATATAAACACTCCCATTTCGCCAAGAAGAGCTGCTAGCCATTTCATTTCACATGCAGTAGTGGAGAGTGTGCGGTACTCGGCTTCGGTTGAGGAACGTGACACGGTTGGGTGTCTTTTGGCAGACCATGATATCACATTTGATCCAAGCAAAGTACAGAAACCTCCAGTGGATCTTCTTGTTTCTTTGCACCCAGCCCAGTCGCTATCACTGAAGGCTTGGAGAGTGATATCCGTGTTGGCACTGAGATTGATCCCAAAAGTATGTGTTCCTTTTAGATACCTCAGTACACGTTTCAGGTTTGTGAAGTCCGCAACCGTCGGAGCATGCATCTTTCTACACACATAATTCACAGCATATTGTATATCAGGTCTTGTCAGTGTAAGGTATTGGAGTTTACCGGCAAGACTTCTGAAATAGGTAGGCTGAGGAAAGAGCTCTGTTTGTCCTGGTACTCTATCCAATTGCAGTGGCAATGGTGTAAGAACAGGAGCACACTCGTCCATCCCAGCGTTGATTAACAGGTCGATTGTGTACTTCTCTTGACTGAGAAACAAACCATCTTCGTGAGCATGAACCTGAAGGCCAAGGAAGTAATGAACTGGACCCATATCCTTCATTCTGAACGTCGTGTTGAGAGAGTAAAGCAACTTCTCAATCAGCTTATCATTACTGCCAGTCAAGAGCATGTCGTCGACATAAAGAAGAAGATAGATGACATCAGTACCGTGAAGATACACGAACAAGGAAGGATCTCTGAGGGTGCATTTGAACCCAAACTCGATTAAGAAAGTACTGAATTTGTCAAACCACGCTTGTGGAGACTGCTTGAGACCGTAAATAGCTTTTCGTAGCTTGCACATATGGTGAGGTTTGTCTGGATCAGCAAAACCGGGAGGTTGTTTCATGTAAACAGTTTCCTTTAAATCACCGTTGAGGAATGCATTCTCTACATCAAGTTGTTTTATCTTCCATCTCTTGACGGTGGCAACATGTAGGACTGTCCGGATAGTAGCAGTTCGAACAACGGGGCTGTATGTCTCCATAAAATCTATACCTTCTTTTTGACTGTTTCCATTTGCTACCAAGCGAGATTTTAACTTATCCTCTGTTCCATCTGCCTTCAACTTCACTTTATGTACCCAGTGACAAGATAGAGGAGTCACATTAGGATCAGGAGGTACTAGATCCCATGTGTGAGTAACCTCAAAGTTGTTCATCTCATGCTTCATCGACTTGTTCCAGCGTTCATCTTTCAATGCAGCAGCGGTGGACTTTGGAGTAGGATAATTTGTCTTGACGGTGAGCAAGACGTATCTAGGATTTGGTTTTCGAATCCCTTGTTTTCCTCTTGTCATCATAGAATGTGTTGGAACAGGCGCCATAGAAGAAGAATCAGTAGAAGATGTCTGAGACTGAACAGGAGATGGTAAAGGAGGAAAATCTGCGTCGTTTAATAGGTTTTGTTGAGGTACTACTACTGCAGTCACAGGAACAGCTGGAGAAACCAGAGGAGGCATGTCTTCTATGACAGGTTGATCAGGTTCTTCAACTGACTGAGGCTTCATAAAGGTTGACTGCCACGCAGATAACAAAGGAGTGTCTAACGATGTATGAAACTCTTGATAATCAGTAGCATAG

>TCONS_00063702

GAAGTTGTGGAAGAGAAGAGTGAGTGGAAGAAAAAATGGCAAACTCAGCTCTCAGGGCTAAAGAAAAGAAGCCATCAGAAAGAAAACAACTCAATGTCTTGGGCTGCTAGAATGCGCCGTCAACAGCACATGTCTGAAGATTCTTCTTCATAACCCAAAACACCAAATCTATTCGCCTCGTTAATGTATAGGTTATAAATTGTTGATGTAATAATAGAAAGCATAGTATAATTATCCACGAATTTGTATATATGTATATACACTTTGATGTACCTATGTCAATCGGCAGGTATATCAAAAACCGTAATCACTAAAATAAGTTTCTTTTGTTTGTGCTTTTGAATTGTTAGCTTTGCAATATTTTTTTTTATCTACGACACGAGTTCTGAACAACACTAAAACCAACTTACAAAACCTGAGATCGAAACAATGAAATGTTACAAAGGGTCATCCATTCTCGCTACCGATTACTACCCTTTCCTCTACAAACGACCAGTCTTTTCTCCGTCCGCTTCGTTCCCATCTACCACTATCTCCTACCCGGCCCGAACCCGATTCTTATCCACCCGGATCCAAGCCAGACTCACCCAAGACGATCCAGTTAAACAATCTGAGGATTTAAGCTTCTATGATCTCCTCGGCGTCACCGAATCCGTTACTCTCCCGGAAATCAAACAAGCGTATAAACAGCTTGCTCGTAAGTACCATCCCGATGTTTCGCCTCCGGATCAGGTCGAGGAATACACAGATCGGTTTATTAGGGTTCAAGAAGCTTACGAGACTCTCTCCGATCCTCGCCGGAGAGTTCTATACGACCAAGATTTATCAATGGGATTCTCATTTTCCTTCTCAGGTCGACGCAGGAATCGATACGATGAG

>TCONS_00042552

TAGCTAAAGGACCTCTACCACCAGTTCCCAATATGGAGATCCCTGAGGAGTTTGTAGTTAAGGTTGCTGATGATATTCGTGTTTTGATCAATCGTGTGTTGTCAATTGCAAGTGATATAACCGTTGCTAGAAATCCCATTCGTCTTCTTCAGGTTAATACTTTAGTCTTTTGAGTTTTCATAACTTGGCTGAACTCTCTCTTTTAAATCCTTTTTGTTTTTGCTTGTGTAATAGTCTAAATCATTCTCGTGTTGGCTTTTGCTGTTAAATAGATGTCACTGGTCTTGTGGGCGATATCCTATGTGGGGACGTTAATCAACTCCCTCACTCTTGTCTACATTGGTGAGTGTCTAACCTTTTTTTTTCCTTGAGTATAGTGTTATAATCTTTCAGTTTATATGAGTTACTGGTTTATCTATAACAGGGATTCTTCTAAGTCTTTCTGTTCCTATTGTGTACGAGAAGTACCAAGACCACATCGACGATAAACTGAACTCGACTTCTAAAGTCATTCGCAGTATCTCAATGAAGATTCCGATGATGCCCGTAAGCAAAGAAAAGAAGTACCAGTAGTTAGTGTAAATGGGCGATTCCGCTTCTTCTTCTTCTTCCTCATCACGCGTTTTTGTTCACAACTCTCTGGGCGGTGGTTCAGGCGAGTCTTTTTCCCTTTACCATTCCAATCGTCTTTCTGATTATCATCAGAGTCTAATCGAGATTCTTCGAGTTTGCCTCTCGATTCAATTGTTAATCTTTGAGATTTTGCGATTAGAACGTTCTTGCTTATTACTCTGGATTGATCGGTTATCTCTAGGGTTTAGTTGGGTTGTTTTGCGCATCTGAGGATCATTAGTTATGAATACGTTTTGACAATTTGACGAATTTAGAGACATTAGTAATCGTTTAGATAGTGTGTTGCCTCCTTAGAACACAACAGCTTGAATTTGAAGTTTTGAGTTTTCCCTTTTTGGTGTGCAGTTGCTGATTTACTGCTGTGGAGAAACAGGACTGGTGCAGTCATTGTCCTAGTCTCCTCTACTGTGTTCTGGTTTCTGTTTGAGAGAGCTGGTTACAATCTCTTGTCTTTCGTCTCCAACGTTCTGCTTCTTCTTGTTGCCATCCTCTTCCTATGGGCCAAGTCTGCTTCCCTACTCAACCG

>TCONS_00033192

GGAATAGGTAATGTCAATGATCTTCCAAGGATTGAAACTGGTTAAGGAGCTTGAGTCAAGCTTACCGGAAAAGTCACTAGAAGCTCTATCGACCTCCCTCGACGAGATCACGAGGACATTTGGTGATGCAAACGAGCGGCTGAAGATAGTTCTTGAATTCAGAAACTCGGTGGTTGTGTCTTGTTCAGACCAGTTGCTAATGCAGAATGAACCGGGTCTGATGCAAGAGTATTGGTTAAGGTGTGGCGGGTCCACGTCATCTCAGGGAACCGGAGCCGTGACTCAAAGACAGATCATGGCCGTTGAAGATGGCGGAAGAAACTTGACGGCTGTAGAAAGATCAGGCGGCTCCGGTAGCAATCCTTCCACGCCAAGGCAACGCAGAAGGTAAAACCTAAAAAGTAGAATTGTATAATTTTTTATTTGGTTTATGGTATAGAAACGATAAAAGTCAGAGAATGATACCGCAAAATACTATTGAATCAACAAAGTATTGTTTAAAAATACCACAACTTTGAAAATTGTTTCAAAAATACTACGAAGTTTAGGTTTAGTAACGATTGCTAAAATTTTAGCATACTCAAATGATTCATTGCGGTATTTTTCGAAAGTTGAAGTTATGTCCAAGTGGGCAAGTATAAGTATAACAAGTTTCTTTTGTTTTTCATTAAGAAGATAAAGCAAGAACATATCTAGCTAAATCTTTCAAGAACTCGATGGTTGATTATTTTATGTTTATTTGCAAACTACTAAAAGAAAATAAAATCTATGCAGTACGTACCGTATGTACTAACGTGGATTAAGTGAGTTCATATTTAGGAAAAACGAAGGAGGAGAACACGTGGTGTTGGTGGCGGCGTTAAGGACAGGAAACATAAACCTACCACCTGACGATAACCATACTTGGCGTAAATACGGTCAAAAGGAAATTCTCGGTTCTAAGTTTCCTAGGGCGTACTATAGATGCACCCACCAAAAGTTATACAATTGTCCAGCTAAGAAGCAAGTACAACGCCTCAATGACGATCCCTTCACCTTCCGAGTCACTTACCGTGGTTCACACACTTGCCAAATCTACTCAACCGCTCCCACCGCTTCCGCCGCTGCCCCCACCACTCCAGTTACAACTAGTTTTTCCCCTGACTACGGTCCCCCTCTCTTCGACATGGCTGATGCTATGATGTTTGGTAGCAGTGGGATTGGGGTCAACATGGACATATATCCTTTCAATGATCCAAGTCATCATGATGATCACTGTTACCGACGGTCAGAAAACAGCGACGACGAAGACAAGTAG

>TCONS_00076445

AAAAATAAAAGCACCTGTTTATTTAAAGAAAAGTCAAGAAAGCTGTCTGAAATTGAACAATGGAGCACAGTAGCATACATGTATCTTCCGGAAAAACAATTTAACAAATCCCCAAAAATCCGGTCTCAGATTTGTTGTTTATTTCGTAAGATGTCAAGAAACCATATAATAACATAAAAATAAAGACCGACAACATTTGAGAGGTGGTAGTTGTTGAATTCATTACTCTTCAGGGTGGACAACGCTGATCTCGCCCCTCTCAAGCAAGTCATAGAAAGCTCTGGTCTTCCTCTGCTCGTTCCAGTGATGCATCTTCCAGAGACCACCAGCAGCTAAACCGAGTGCCAAACCGATAACTAGCTCCTTCACCACACTCGGGCCTTTCAGTGTGGCATGCGCAACTCTGTGTCCCGCCATTTCAACCTACCAACAAATCCTCTGCACGAATTTAAAGTCATTATCATAATTTGAAAACTTCATAATCCTTACGAAGCTAATAAAGCTACAAACGTTATTTAACAAACACTAAACATCT

>TCONS_00026461

GTTTTGGATTGAAAATTCTGGACAATGGATACGTCTTAATTAGTATCATCGAGGCAATCAAGCAGCTGTTAATGTGAAAATTAGGTTATGTCTCCGGTCAGAACCAATGTTGAGTTCATTTAAATATTTTTTTTTCGTTCTGGATTCTGTACAGAGCAAAGCAAGATGGTCCTATCGTCATGGTACGACTCTCTTTTTTTTTTGCCTGTAGTCTGATCGAATCATTAAAGTTCTTATGCGGAGTTCGGTTATCAATAAAGATTGAAAAAAACTTATCAAATGCTCGTGATTGTGGTGAACAAAAATAAAAATAAAATGCTCGTGATTGTTTTTACGCGGAGTTCGGTTATCAACCAAATATATAGAATTACTATAGATTACAGTTAAATAAACAAATAAGTTGGAACGATTTCTTACTATTCAATCGTTCCAACTTCGTCTTAAACCGAGTGCATGTAATGCGGATCTCTCTCTTCTACAATCGTATCTCATTTTGTCATTGAGTTTTCTTTGTATAGCTTTAATTCATTGTCCAAAAAACTGCTTTGATGTTTTTTTTTTTTGATGGTTCATGAATACAGCTGAATCTTCAATTTTTGATTATAGATCTGAAGTTATCCATCTTTTTTTTCTTTTCCTATTCTAAGCTTCGTTTGCATCACTGCTTCAGTTTGCATAATACACAAAGTGAGTAGTGTTTAATGCTTCTATTTACACGAATCAGTTAACTTCTCAGTCGGGTCTTTCCCCGAGAGATTCCACGTGTTGTCTTTGTTTTCCTGATCAGAAACGCAAGCCTAAGCTCTACCTGATGCACGCCGCTTCCAAGGAGCTGAAAATTCGTGCTACTTCCAGCGTTTCTGAACTTCAAAAGTGATAACTTTATTTGTCTTAACTAGAAAAAAAAAACTTTTAATGAGTGTTTTCCTCTGTTCTGTCCAGGTCTACTTCAAGTAGCGATGTTAACATGACGGCAGAGTTTGAGTTGGAGTTGCAAGAGTTGTTCAATGATGTCAAATCCATGGTTAAGATTGGGGTGAGAGTGACGCAATGGACCTTCTCCGAGCTAATTACGTTGCTGTCAAAGAAGAGATCGATTCGGGCTTGAAAGGTGTCGAACAAGGTGCTCTTCTGGACATAATTGCCTTAGGGTTTATGGCTGAGACTTAAACCTCTTCCTGCATTGCTTGATATGGTGAGTCTAGAGTCAAGACATAACATTCTTCTTTTAACTGGATTGTGTTGTCTGAAACATAAGTTGACAAATGCAGATAAATAAGATTGTTGATAAGTTAAGGGACAACAAAGACGAGGTAGTTCTTGTTATCTTCTAGTTTTCTTCTATCAGTTTTGGAGGGACTAAGAAGTTTTTTTTTTTGCTCACAATTTGCAGATTGACATGTCATTTCTTGTTGCTACTCGCTGGATAGTACCCGCTGATGTTGCAGCTAGTGGTAATAAACCCAACTTTTCTGAAGATCTATAATTTGATAAAACAGGATTCTTCTTTCCCAAGTTAGGTATTAGACTTTTCTTGTTTTCGATTTTATTTTATACCCGTTGATGGTTTTTGTTAGTTATAGTGCTACTAAGCATATGGAGAATCCGGCAGAGGAATGCGTAGGAGGAGAAGAGCATATGGGAGGAGAAGAGCATATGGGTCGAATCGTTTAGGAGAGTCTGAAAATTCCAGTGCGAATCGGGAATGTCTAGGGTTTCGAGAAAATGGATGGCGGAGGTATAACAATAACATACAAGGGAGTCACTGTACACACTCCCAAGACGTGGCACGATCTTTATGTG

>TCONS_00047164

GAAGCTTTTTGGTTGGAGGCGGTTAACCAAGCCGACCTGCTTACTCGAATCCGGTTGGCTCAAGGTCAGGACGAGAACCTGAATAAGGTTGCTCAGAATGATAGGACGGAGTACCAGACTGCAAAGGATGGTACCATCCTAGTAAACGGTCGGATCAGTGTTCCTAATGATTGAAGTCTAAAGGAAGAGATTATGAGGGAGGCTCATAAGTCCAGATTCTCGGTTCATCCTGGTGCAACCAAGATGTACCAGAACCTTAAGAAATTCTATCATTGTTGGGACGGCC

>TCONS_00014366

AAATGAGTTGGTTTGTTAAGGAAAGAAGAGGAGGTGTATGGAAGCAAGGATGACGTCATCAGCACCTCTACTTACTCTCTTTGCCATCATATCTCTACTCCTCTTTCTATCTTCCTACCCACGTTACAGGCTTCTATGCCGCTTACTACAAATCCGATTGTCTCTTCCGAATCCACTTCTTCATCTTCTTCTTGTGGATGTTCGTGGTCGTGGCTAAAGCCGTTTTCGTTTACCGTCTCAATAACGAGACCGATCCTAGGCTGTACCCTGGGACGAAGATACATGAGTTTAGGTTGGAGGATTACTCGGGATGGGTACGTAGATTGGTCATCAAAGACGATGAATGGTATCGTACTAGGAGATGTCTTGTTAAGGGCAATGTTTGTAACAAACTATTCTCTAACCAAAACATGTCGGCTTCTGAGTTTCGTCAGATGAATCTAACTCCTATACAGTCGGGTTGCTGCAAACCACCGCTTTCATGTGGATTAACTTACGTGAAACCAAACATTTGGACAATGTCAAGATATCATAACAACGTTGAAGATGATTGCAAGACATGGAACAATACGGCAAACACATTATGCTTCGATTGCGATTCATGTAAAGCCGTGACTATTGCAGATTTACATAATACTTCATTTTCCTTAACATTTAATATCCTTCATATCGTATTTAGTCTTTCCATTGGCGTTGTTGGTTGGTTTGCTTGGTTAAGGATCCTTCGAGAAACTGAGAACTAGCTAGACTTTAATTCTAACATATGTATAAATATATCTTTCTCTTGTTTTATTTTTCTTGTACAGTCTCGATTCTTTACCGATATGCTGCATGTGAAGTCAATCGACTCTTTTAGTTTTCATATTCAGAATGGTATAACTAAACCAAACCAAACCGAACCAAAGTAAAATGATATAAACTGATGAAAAAATATTTTGTCAAACATATATATTTGAAAAATCCTTTATTTTGCTAAGAAACCGAATCTATTTTTAAAACTTGATGGTGAAATCATTTTTTTTTTCTTCTGATAATTAATAATAGAGATACCCAACCCGGTTAAGCTAACGTCATA

>TCONS_00056235

CCCCAATATAAGTTTATTTATCCTTCCAAACAATCAAGATGCATATACTTTAGTTACATCTCCACACCAACCCAAAACCACAAAAAGCTAAACTTAAGAATAATAAAGGGGGCAATAATACTAGTTTTTATAATTAATTTTAATTCAATACATATTGATATCATTAGCCATGATGGGTTGGTTAGTGTAAACCGAACCACCCCAATGATTAACGCCACTCATCATCATATCATCGTAATATCCGTCGCAATTAATCCCAG

>TCONS_00009801

GTTATTCCCGGGATGATGGAGAAAGGGAAAGGAACTATACTCTTCACCGGTTGCTCGGCGTCATTGAATGGCGTTGCTGGTTTCTCCGAGCTATGTAATTCTCTTTTGAATATTTTAATATCTGTAAAACGAAAATATGCTTTTAATATTGGATATTAATAACTAAAGAAATATTTGAAAATGGAATAGGCTGTGGAAAATTTGCGCTGAGGGCACTTTCTCAATGCTTAGCAAGAGAATATCAAGCTTTTGGAATTCATGTGGCTCATGTTATCATCGACGGTGTGGTTGGTCCTCCAAGGTACTTAAGTTATATGGTCATTAATTACAATATATATATATATATATATATATTATACTACAAATTATCTACAAAAGTAGGGAAAATAATAGAAGATGAAATTAGAACCACAAAATCTGTATAGAAAGGTGTTTTAAACTTTTCCTACCTTCCACAAACGGCATTGTTCTGATCCGAACCCAAAAGATTCTAAGAATCGCTAATATATGAAGAACCTTTTCACTATCGGACTTTACCAGACAAAAGTTTTAATTGGCACATTAAGCATTTGAAGATCTAAGAAACAAATAGGATCGGATGAGATAAAATTCCGGAAAATGTATTTTCAGAGAAACAAACATCCCACCGAGAGGAGTGGTCGCAGAGCAATCATACAACTGTAGAAGCGAAGATGGAGAAGGAGAAGGCGAAAGCTCAGGAGTGATGGGAATGGATCCTGATGTATTGGCTCAAACATATTGGAGCCTTCACGTTCAAGATCGGAGAGCTTGGACTCAAGAACTCGATATCCGACCATCAAACACAAGGTTCTAGTAGTTAAATATGATTTGATTCGATTAAAAAAATATATATGATTTGATTTTTTGGGTTTCTGATTTTTTTCGATGTTATTTTGGTCGTATGGTATGTGGTAAGTATGTGAGAATGATGAGAAACGTAGGAAGTTCGAGCTCCGGCAGCAAAGGCATAGCGGCGGGGGGCGGCGGAGGTCCAAAGCTAGGCCGTTCAGTTGCTCGAAAGTTCGCACATGAAGGCTACACCGTTGGTCGGCGTAGGTCCAAAGCTAGGCCGTTCAGTCGCTCGAAAGTTCGCACATGAAGGCTACACCGTCGCCATTCTCGCCCGCGATCTAGGTTTGAGTCCTCTTACTCTTTTGTATCGATTTCTCTCTACTCATACATACAGATACGCACACACATAAACTAGCAGAGATTCAATGTACATGTATATGTATGTGTGCCTTTCTAATGCGCCCTCGGAATTTTCCTTCTCTGTTTATTATATTCCAAAATTGCGGCAGTTTTTCTTTTTAAAAGGAGTTATTTTAGATTTTTTTTTTCTTATTAGATATAATTAAGTCAAAGGTGTTTTCTAATATATTTTTTGAATGTAAATTTTGTTAGAAAGATTTCAGATTTGCTAACAAGACTTCGAGTTTTACATCATTACATTTCTGTAGGCTTAATTGGTTGGCAACCAGTCTATATTTTTAACAAATGTTTAAAAAAAAATAAACAAAATTGACTGTGTAAGTTTTACTCTGAGTTACATTGATTGGTAACTACTACATATAACAGACTAAAGTTTTCTTCTTATACATTAAATAACACATAGCAAAGAAGCTTTATCAGTTTCTGCAACATGCTAGCTGAACTAAGCTTTTTAGAACAAGTTGACTGAAAACTTATCTAATTAAATTGAAATAATTTCAGAATATTCGACAAAAATACTTGAAACAGTTGGATTTTTATTCTTCATCCTGTTCAGTAATGAAAAAATATTTACTTACTTGATTTCTATTTAATGTTAAGATTTTTTGTCCATTTAAGTCTTTTTTGGTGTAAATGTTAAATGTCCATTTAAGTCTTACCTCTTTCATTCCACTTTAATACCTTCTCTCATATTCTTACTCCAGCTTTTTACCAGTCACACCCATGTACTTATATTAATCACTAAGCGGTGCTAAATAGAAATATAATACTTCAACCAGTTCTCGTGATATATATGGTCGGCTGAATTGTGCAGGCAGGTTATCTCGAGTGGCTGAAGAGATAGCGAGAGAAGAGAAAGCGCAAGTGTTTGCTATAAGAATTGATTGTGCTGATCCAAGAAGCGTAAGAGAAGCTTTCGAAGGAGTGCTGTCGTTAGGGTTTGTTGAGGTTTTGGTGTACAATGCTTATCACTCGTCTTCTTCTTACACAAGTCACCATCCCACTTCATTCACCCACATTCCTTTCCAATCTTTTCATACTTCTCTTTCTGTCTCTGTCTTCGCTGCATTCCTGTGCGCCCAACAG

>TCONS_00044141

CTCAATTCATTTAAATAAAAACAAAAAAAAAACGTAAAAGTTTTCACATGCACGTAAAGTTGGGTGAAGGTTCTAACTACTTTGTCGGTCCAAATAAATGATGCATATCTTTATTAAATGATCATATCAAAGTAGGTAACAGATTTGAAGTTTATATATACTTCATACACTACAATGAATCCAACCCCTTAACTTATCGTCAGGACACCGAAAAGAAAAAAATGAAGGCACCTTCACAACAGGACATGGCTTACTTCGACCACATCAAGAAACGGCAAGATGATAAAGGCTGTTTATACGCAACGCTTTACGGATTGTTCTGTTGCTGTTGCTGCTACGAGACATGTGATTGTTGTTGCTGCGTCTGATTCTTATGATCATCTTATAATAGTATTATCTTTATGTGTCCCTGTTGTTTTATCCAAATCTTTTCTATGTAATAAAGCGCCACAAATGGGAGAAGTTGGGTTAAGGTTTAAATTTTGGGAATGGTTGGTTGGGTTCGGTTCTAATTTAATCCGAGTGTGGTTTGGCTCTAGATTAAAATATGTAATAAAAATTAAATAAAGA

>TCONS_00052240

ATTTATAGAAGACAGCAACCAATCAGCTTCAGGTTGGTGGCAGCCCGTGTGTCGTTCTGCATGGCTCCGGACGCATGCGCGGCGGTGCCTCGTGCTCCACATGGTTGGCTGAATGTCTCAATCACATTCAGGACAACACACCCACGTCCAGATGGCATTCCGCAGGACTGGAACTCATGCAAGGCGACACAACATTTCACACATGTCGATCAGCAGGCTTCGGTTGCATGCGCAGCAACACCTCGTGCTTGGTCGATCCACCTCGTGCTTCTACATGTCAGGCTGCATGTACTGTTTCCATGCAAGGCGACACCTCAAGCTTCTGTAGAAACTCAGCTTGTTCGATGGTTGACACAATGTTCTTAACCCATGCAACGAGACACCTCGAACTTCTCGGTTCATTTGTCTGATTTTTGTCTTTCCGGCAAATTTCTGATCTGCGATCAATCCTGAATATTTTTCTGCTCCCATTCTGATGGCTGATGTTTTTAATAAATGCTGACCTTGACGAAAAATATTTCCCGAGCCTCCGCCTTCGCCGAGAAATTCCAAAATACTAAAATTAAGGTTTTCATCATATTTCGGATTTTCCCATCATGCTTCGATCCCGTCTTGCTTGCTTCTCGTCCTTATTAATTCCCGTCCTGCTTCCGACATATTATGTTTCCAACATAATATTTTACTGATACGAAGTTTTGCAGAAAACTTCGTGCTGAAGAAACGTCGCTTTTCTAAAAAGTCGAGCTTCTAAAATGTCGTCTTTCAAAAACATGATGCTTCCAAAACGTTCTTTTGATCAATCTATTATATTGTCTAACCATGATCGAGCAGTTTATTCGACTCATGGTTCACTCATGATCAATTCTTTGACCATCTTGTACTTTAAAACTTCTCGCTCGATCTTTATCGCCCGCGATTCGACCACCAAGTTGATGCTCAACCAATCTTCTCTTTTCTCTGAATCATGTCAAGTTTTATGTGATCAAAACTCAACATTCCTCCAGATACTTTAGATTCCCAAAAGCTCGGCTCTAAATTCTAATTTTGTCGATCTTGACCATTTTACCTCTTCGGGAGGGTTACTACACCTCAAGCTTGGATTGATTCATAAAAGAAAAATCACTGATGGTGGACATGACCTTAAAGCAGCTGCTGAAGAACTACTTGAAACGGTTTTGGTTGCCACAAGTCAGTAACTCTCGTACTTGCATAGTTCTGA

>TCONS_00056461

GGGAGTCGCAGTAAGCATGGAGATTACGTATTTTTAAGCCGTGAGCCAAACGTAGACCTGCGATTAATGCTTCGTATTCGGCCTCGTTGTTTGAGGCATGGAATTCCAGCCTGAACGATTGTTCCAAGATCTCGCTCGTTGGAGATGTGAGACGGATCCCGATACCCGATCCTTGCTTGGATGAGGATCCGTCGACGTGGAGGAGCCAGGTGGAATTTGGTTCCTCATTGGTTATTGTTCCCGTTGGAAGTTCGACCAAGAAGTCTGCGAGCACTTGTGATTTTGCGCTCGTCCTTGGTCGGT

>TCONS_00057321

GTTTTCCGGATTACTTGGTAAGGAATTGTGGAGCCATAGCTCAATTGCATGCAATCTTTGAGTAGTTAATTACCAACCGCGTCTTCTTTAGCTGCTCGGGTTGAAGATTGGTTCTATGTGGCTTCTTGTAGAGGAAGAAATTTTATGTGTGGTGGTTGAACGTGCTGCACTGGTGAAAACGTTATAGGAGATCATCTTCCAACTCAGATAATTAAGCTTGCATCGCTTTTTCTTTCATATATGCTTTCGGCCGTTTAGCTTTTCCATTTTTGAAATTCTTCGCTTATCTCCCTATGCTCCTTATCAACTTCTCTTCTTCTTTTAGTTTAGCGTATGTTTTAGTGGATGTATCAAACTTTGTCTCCGGATGGTGCATATCACCATGCTGGCTTATGGTTCCGGAAAGGTTCCAACACTTTTGTTGGCTTTGTGAACTACCTTGTTCAAGTTAATATTAATCCATCAAATGACAAAAAAAAAGGATAAAAAACACTATACACATCAACAGAAACCAAACCAGCTGAGAGCCATGCGACTACTAATAGCATATGTAAGCGACAAAAAAATAAAAACCACTAAAACTTGGTCACAGAAAAAAAAAACCTTTGAGAGAAAACCCTAGAGCTACCGCAAGAGAAAAAGAGTGTTCATTGGTCTTACCTTCTCCGGTATCCGGCGACCTTCGTCCGTGCTGTGAGAAGGTCCTTAGTCTAGTATAGTGTCGTTTGTAGGCTGAGAAGAGGCTTCCAAGAAACAGGGGAGTTGGAGATAAGGGACTCGATGTCGATCTCTTCCTGTCGATTGCTTAGGCTGATTGACTACATCCGAAGCTTTTTCTGCTGTGCAGTGCAGGTTATACCGGCTATGATGTGGGGTGGTACTTCTTCCATCGGTTCAG

>TCONS_00028769

TCGAGATCGGCGGTGATTCAAGGTTGAAGAACATGCAATCAGCCTCAAGTATGTCGGAGATGGAGATGATCGGCATTCCAGCGAGTTGGTTCCGGCTTCGCTCTTTCCTTCCTCGACTAAGGTGTGGAAGCCACATAGAGAGACCACGTGTCACTCATATGATTGTAATTATCTGTAAAATATGGGCTTCAAGTTGAGATTAATATGTAGTGATAATGTTTGTATCCTTGACCTTTGGGATGTTGTTTTAATAAAACAAACTGTTGACAAAAAAAAATCTTGTGTACCTTTACGATGTAAAAAAAAATTATTTCGAATAAAACCC

>TCONS_00048893

CAAATCTTAAAGATAAAACCTCAAAACAAATAATTCACAAAGATCTTCAACGGAATGAAAGACATTTCCTCAGCCTGAGAGGCGCACCCAGAAAAAATGCAGAACAAGCCATTCAAGGAGGCGACAGAAGAATCTCATTTTGAAACATTCCCCCCAACATTTTGTTTAGTTTGGACGTCCGAAGCAAGGAGACCAAAACAAAAACAAAAGTTGGGTTTTATGGTAATTGCAATACGGCAAGTCATAAATCGGCATCAGACTCGTGTGATCAAGTTCTATTGATTTGATATCGATTAAGTAAAATAGAGCATGAATTGTCTTAAGTTCTAAGTTCTACAATATGAGATCAACTTTAATAATTTGCAACAGTAAGACCAAAAATTGTAGTATAGACCATCTTGTGATGTGTCTTTTCTTTC

>TCONS_00023917

GAAACCGCGAGCGACTTCTCCCTGTCGCTCCCATAACGTCGCTCCCAGCTGGAGCGACCTCGCTGTGTCGCTCCGAGAGGTCGCTCCGGGCTCGTTCTCGCGTCTCCGAGTGATGAAACCGCGAGCGACTTCTCCCTGTCGCTCTGGTTAGGTCGCTCCAGTAGGGTGATCAGAGCGACTTGGTGGTGTCGCTCCGGACTGGTCGCTCCCATGCCTTGCTCGCCCAATGACCACTCTAAACACTCCTTTTT

>TCONS_00073976

GGAGCTGATTGATCTGAAGGATACCGCTGCGACGTTTGATGTCGATAAGACCATGGCCGTGAATGGTGCCAAGGTCGTTGTAGAAACCCGTTAAAAAACAAAAAAAAAATATTATTATTTAGTGGTCGTACCGCGGGCAATGGATGGTCGAAGAATTGATCATGGGTGAACCATAACCTGCTGGACCATGGTTAGAAAAGTGTTAGATTGATCAGAAGGATGTTTTGGAAGCATAACATTTTTGGAAGCACGACGGTTTAGAAGCTCGGCGTTTTTGAAGACTGACGTTTCTTCATCACGAAGTTTTCTCGGAATATCTTCGTATCAGTAAAATATTATTCTGAAGACATTATAAGTCGGAAGCAGGACGGGAATTAAGCAGGACGGGAAGCAAGCACGACGGGATTGAAGCACGACGGGAAAACCCGAAGTTGGGCGAAAACCCTAATTTCGGTATTACGAAATTCTTTGAGGAAGCCGGAGGCTCGGGAAATATTTTCCATCAAGGTCAGGATTCACTTGGAGTTTATTAAAAATATTCATCTCATCAGAACGGGCGCAGAAAAATATTCGGGATTGATCGCGGGACGAAAATTCACCGGAAGGGTCGAAATCAGTCGAATGGACCGAGAAGCTCGAGCTGGCCTTATCTATGAGATAAGGACGTGATGTAACCTGCCTGGCATGGTATGTGGCAAGGGAAGCAGGAGGTGTTGAAGCCCAGGAGAGCACAGCATGCTGATTGACACCCAGAAGCACGAGGTGCCGCGAAACCTGCGATCGGAGCATGCTGAGCGACATGTGTGCGCTGAGTGTCAATCTGGATGAGGTCAGGCCGTGGATCAGACATCTGGAGGGCATGGTGTCACTCTGCATGATGTCCTGGTCATGCCATCAGACATGTGGAGCACGAGGTGCCGCGACGCATGCGTCCGGAGCCATGCGAGGCGACACACGGGCTGCCACACGGCTTGTTCTGATTGGTTGCTGCATCCTATATAAACCCCACGACCCCAGCTCATTTCAAACCATCCAGCACACCTGAAACTCAGAGAAAAACGTGAGAGAGAAAGCTAGAGAAAGAAAGTTCGAGTTTCGATCGTTTTCGAGTCTTTTCGGCAGTTATCGAGAACAGTTATTCTACTCGATTCGAGTCTACATCTAAGAGAGGTTCTGCTCGACTCAAGATCAGTTCAGAAGTGGGTCTACTTGTGAAGGTCAAGAAAGGGTTCGGAACGCAGAAGACGGATTCGGGGTCAAGGCCACGGTCAACCAAAAACTGTGAGTTATAATCAATTGATTGCTGAGTTGTTTTTATGCAGGGTCCCGTTACTTGGAAGTTGGATCATGGCAGGAGGCCAGGTCTAACTGAGTAACGGTTTGAGTAGTTAATAATTGAGGTTATGTTCATTAAGTTGATATCAT

>TCONS_00008207

GTGGCGAGGAGGGAAACTCTTCACACCGGGCAAGCTACATGTGATGATGTTATGGCAGAACAACTTCGTCATCGTCATCAAGCATCACCATCATCAACATCATCACATCATGTGGCTCATCACCATCATCAGCATCACCATCAGCAACAACAACGGTATCATGCATTCAACATAAGCCAGCCTACACATCCAATATCAACCATCATTTCTCCATCTACATCGCTTCACCATGCCCCCGTTAATATCCTTGACGATAATCCTTATCGTGTTCATAGAATTTTGCTCCCTAATGAAAATTACCAGGTAAGATAACTATTATTTTCTCTGTTCTTATTTACACATTTTAGTAACATTTTCAGAAAAATATACATTATTTAGTTAGTTGCGTTCACATCCCCTCACCCCAGCTCATTTTAATTAATTAGGTGAAAGTTACAATTTCATGAATATGAGCAAGTTAGCTGGAATATCACTATAGTTCGATAATCAAACTGATTTAGGAGTCATGCACCTATCATTTGTATAAGGGTCTCAACGTACAATTTAAATATGTGTATGCGTACATTTTACTACGCTGTACAAATTTTGGTTCTTGCACATGTTAGGTTTTGAAGATATTTGCATGCATATGACTTTACAATGATGGGACTACATAAGCATGCAAACTGAGTCATACCAGAATACATTGTGAAAAATAAAGGGCATAGAAGATAATTGGTCAAGAAAAAAAACAATTAAAATTGAATATAAGTATGCCTGATTTAGTCTAACTATCTATTATCCAAAGGCCAACCAAAAGTTAAGCCACTTTTTAGTGTCAGCCAAACTAAAAACAAGTAGATAACCAATGTGGTCTTAAGAGACAAGATGATTAATTTTAATGTTTAAAGACAGTTTTCTTAAAAATTAAATAATAGTTTTCTGAAATGACTTTTGCCTTTTTGACTGAACAAGGCACACCAACAACAACAAGACCAAGAAAGAGACGAGCAACACAATGATGGGAAGATGGGAGGAAGGTCAGCTTCTGGATTGGAAGAACTCATAATGGGCTGCTCTTCTTCGAACACTCATCATGATGTAAAAGATGTAAGTTATTAATCCTTTTTGACACTTAATTTCACGGGAAGCCCCTCTTTTTACCATCATGAGTTTAATGCAAAAATACACACACACGTATATATATATATATAGATTACGTATTATATGTGATTTTTATCACCTTATTATAGAATGTCATGCATAGAGTAAAATGTTGTAGTCATCATACAAGTGAAGCACGTACTGGAATCAAATGTGGTTTTGTTTTAACTGAGAGTAGTCTCGGATCTCGATTTAAATGCACGCTCTGTAAAACTGAAAAAACAAATATTTTTAAAAATAAGAACTGTTTATGATCATTGATTGATTGGTCGAAGATAACTATGCATGTTACATTTGAATAAAAAAAGAGAATATTTTTTAAATTTCTTTTCCTTTATTTTTTTCTTTTTCAGTTTTGCATGGGAAGCACTTAAAACTACATATATTCTTTTTATTTTTTTAGTTTGATTCTTTACTTAATTGGAAAGAGTTAAAACATAATTAAGTAAACAAAACCCGAAAAGAAAAAGAAAGAGAGATGTTTGTGCTTTTGATATAGTTTGCTTTCTTTTATCTATAGATTACCCTTCTAAAGAAGAACAATATATTTTTTCAAATTCTTTGCTTATTCTTCCCTTTAAACATTATTAGACCATGACTCTTAGCGGATATAAGAGAGAGACGCCTCCTAAATAATAGATATAAGAAACGTTTATTAGTTTTTTATAATTCATGCCATTAGTATAGAACTAATAGAAGGCAATGATGGAGCATTTATACATTTGTGAATATATGTATACAGAACGTGTGTATATGTAAATATTATTATGTCTTTTTCATGCGTGGTACCTCGCGAGACAAGTTCGGTAATAGACATAAGTGGCTTTCATTGTGTTCTCGTGGGAGACAGCATGTCTGCATTCCTTTTTTTAATCACTCTCAAATCGAGTGGAATATCCTTATTTTTATATATGTAAACATATTTCAAAAATATAATATATATTGTAATTCAATGTAAAATACGTAGCCCTAATATATGCCTAGTTGTGGAATGCAAATAGGCTTATACTTTATGTGAGCCTGGATTAGAATTAACATTAAAGTGAGTACTGAGTTCAGGTTCTTTGTGGGCGGTCAAATTAGGCCAGTTTGTTTCTATTTATAGCGAATGAGATGTACATAGTTTATACATGTATGCTCGCCCTATTACAAACGTATACAGTTTCATATTAATGTATATGTGTATGTATATATTGAGAAATAATATTGATTGGTGCAGGGATCATCATCAATGGGGAACCAACAAGAAGCAGAATGGTTGAAGTACTCAACAACATTTTGGCCAGCCCCTGACTCTTCTGATAATCAAGATCATCACGGGTAATTGTGCCATTTTCCAGTCTGATGATCATGACCCTCATCACAAAGTGTTGTTGCCACTTATACCTATCATGGTGGTTATCTTCATCGTCGTCATCATCATAACATCTCCACAGAAAATGGTAAATCCATCGATCTTTCACCAAAGAAGTTATATATGCATGAATTTGACATGGTGAAGGGTATTTAAGCTGCGCCAAAAAAAATCAAGTGTTTTGGTAAAAAAGTTAAAGAAGGGAAGCGAAAAAAGAAGTATAATCTGTTTTTGGAAATAGAAGAAGAAGAAGAAAAAACAAGGAATTCTGCATCAAAACCATCAATAATGCTATATAGAACCATATATATGTTTATTTTAAAATTCATTTTCTTTGTTCATTAAATATCATCGTCTGTTTTTTCTTTCTTTAGAATGAACCGTAAATTGGGTTGGTTTACCCGCAGGAGTGAAATTTGACCCAACGGATCAAGAGTTGATAGAACATTTAGAGGCAAAAGTATTAGTTAAAGAAATGAAATCTCACCCTCTAATTGACGAATTCATCCCCACCATTGAAGGCGAAGACGGCATTTGTTACACGCATCCCGAGAAACTTCCCGGTACATAGACATATTTATACATTCCATACATTCCATACATCACATCGATCGTTATATATTATATGATTCACTATAGTATATGCTTTGTTTAATATGACTAGGGTACACTAAAGTTTAAATACTTTTTTTTCTTAAGTTGGGTCTCACCAAAGTTTTGAATCTTTTATTAGCTTCGATCACACATACATCTATATACATATTCATATTATTATTTTCATGCATTCCATATTTATAAATCACATACGAATATCTTTCATTATAATATATGATTCTTTGATATGACTAGATATAGAGAATTGGGTCATACCAAAGTTTGAATCTTTTTAATTTGGTTCACACAAGAAACATATATATAAAATAACAAAAATATTTTGTTTATTTTTTCAATTTGGTAGGAGTGACTAGAGATGGTTTAAGCAGACACTTTTTTCATAGACCCTCAAAGGCTTATACGACGGGAACAAGAAAGCGAAGAAAGATTCAAACGGAATGTGACAATAGTATGCATGGAAGTAGTAGTTCCGGGGAGACGAGGTGGCATAAGACCGGCAAGACAAGACCGGTTATGGTAAACGGTAAGCAAAAAGGTTGTAAAAAGATTTTGGTTTTGTATACCAATTTTGGTAAGAATCGAAAACCGGAGAAAACCAATTGGGTAATGCATCAATATCACTTGGGGACACATGAGGAAGAGAGAGAAGGAGAGCTTGTTGTGTCTAAGATCTCTTATCAGACTCAGCCTAGGCAATGCAATTGGTCGTCATCTACGTCAAGTCTGAACGCTATAGGTGGTGGAGGCGGTGAAGCGAATAGTGGTGGAGGCGGTGAAGCGAATAAGATGAGGAGAGGTAGTGGCACTACCAGTGGTGGGAGTTGTTCCTCGTCTAGAGAGATTATGAATGTTAATCCTACAAATCGATCTGATGAAGTAGGTGGTGTTGGTGGTGGCGCTATGGCTGTAGCGGCTGCTGCGGCGGCGGTTGTGGCTGGTCTCCCGAGTTATGCAATGGATCAACTAAGCTTTCTTCCTTTTATGAAGAGCTTCGATGAGTAATTCTCTCTCCTCTTTGCAATTTCTCTCTCTCTCTCTCTTGATTTGCAGATCTTATTCTGCTCGACCAAGTTTGTTCCTTTTAGCATAATTTGAGGGTTAATTCCCCATAAATTCTTACTTTGTCTTGTTGTGTCGGTTAGGTTTTCAAGAAAACCCCTCTTTTGTCTTGTCTCCTCAAAATCAGTGGTTTATTTGTCAATAATAAAAGAGGGTTTTGTTCAAGTCTTCTCTCTCTAGTCTCTCTCTATATATATAAATACTCAACTCTCCGAAATGATCAGAAGCTCTTTGTTATTATCTCTTGACCTACGCACAGACGGATAAGAGAAAGTGACCAAAGGAATTAGGGATCGAGATGAACAAGAGTAATCCTGCTGGCTCAGTTACCGGTTCAGATATAATTGACGCAAAGATCGAAGAGCATCAACTTTGTGGATCCAATAAGTGTCCCAGCTGCGGCCACAAGCTCGAGGGCAAACCA

>TCONS_00006701

GGTCTCTTTCGCTTCAGTTCCGCGGCGAAACATACCTGTGAAACTCTCGGATTTCCCCAAATTACCTCGACTCCGTTAGGGGTCGGGAACTTGAGGCAAAGATGGTAGGTTGATGGGATTGCGCGCATGGTGTTCAGCCATGGCGTCCCCATGATAACGTTGTAAGATGCGGGGCGGTCAACGACTAGAAACTCTGTGACGTTCGTCACGGTTCTGGCTTTGACAGCGAGATTAATCGATCCATAGACCATGGTTGTTTCCCCCGAAAGTCCCAGCAGTGGGCTTGGGCATTTCGTGACTTCGGATTGATTGATCCCCATCTTTTCGAGAGTGTCTTTGAAGATGATATCGGCCGAGCTTCCGGTATCGATTAGTACTCTAGCGACGTCGATATCTCGAATCGTCAACTCGATAACAAGGAGATCGTTTCGAGGTTTGGCTCGATCGATCGTTGCTCCCCCCTTGAATGAGATGAC

>TCONS_00056219

TGTCTAGACTGTATGAACTCAAATTTAAATAGATAAGATTGGGATGGAATAGAGAGATTATTACATAGTTGGAAACCGAACAGAAATAATTAAAAGCCAATAATATAGGAAATAACATCGAATTTTCTTTTACATATAGATGCTCTTAATGTCTTCTTCTGGATGTGAAGGCATGTAGTTATTTCTTAAGAGGAATCTCAATGGCTTCAATTAGTATTACGGTACCTAGGTTGTGGATAACGTCCTGATTCAATGTCATTCACATCTCTAATGAGCAACTCGTAGTGTCTTTTCACCTCTTCTGCTGATTTTCCACCAACTGCTCTTGCAACGTTTTGCCATCGGTCAGGAGTATCCTTGTCGTATGTAGCCAATGCCACTTCGAACTGCTTGTCTTGTTTAGCTGTCCATGACGAGTTTGAGCTCCGGGAAGTGCTAATTGAGTTGGAAGCCATTAGTGTGAAAATGTGGAAGTGTATATGAGTTTTGAGAAGTTGGGTTTGTATTAACGATGAAGATCAGGGTTGATTTGGGTTTAGCTGATGAGAGTGTGGAGCTCCTTATATGTAGGACGAGAAAAGAATATAAGCCAGTTGGTGGAATATATCTTTTGAAAAGAGAGATTTTTAGAGTGACCCAATATTCTTTTAGTAACCACATATATTTAGTGACGGGACAATTTTTTTTTGGAAAGAAGCTGCTTTGATGATTATGACGATGATCATTAGAATATAATCCTTTAGAAGGTCTAGCTAATAGTTGGCGGAATTGCGATATTCTTTGACCACTGCTATAGAAAATCAGGATGCTTC

>TCONS_00069869

ATTTTTTGAATTTTACATGGTTTTTGGCAAACCACTTATTACAAGAGAGAACAAGAAGAAAGACACAATTAACTATAAGGAGAGATGAACCAAAAATATAGCTGAAGCAGAGACAGAGAAGACACAGTCGCTGAGGGGAAAGACAAGAGCCTGTCACGCATAGCACAATAGATCACATGAAAGAAGGCTGGAGGAGACAAGGACGAGCCGCGGAAAATAGCCATTATTTGAATATGACTTCCTAACATCAAAGAGAACTAGTAAGGAATAACAGGCTAATCCTAAAAAAGAAGGAAAAAAACACGCTAAACCGATTACAAACAGACCTTTATTACAAGATTCCCTTCCCAAACACTTATAAGCGACATATTATTTTTTAAAGCTGACATAAACATATATTTCATGTCTGTTATGACTTATGAGCTGCCCGTAGACCCATGTTTGCATCGGTTGTAGATGGTACATTGGCTGTTCTGCTGACTATAGATTGGGGTGCAGAGATGATTCATTGAAGACT

>TCONS_00075184

TGCAACCTAAAATAATCATAATAAGCTAAAATATAAATCTAAAACTCATGAAAATCATGAGATATCAGTTACAAAACTACTCCTTAATAGGGCACATAATGTATGTTCTTTTTTTAACTTTGTTCCTGTTCTTAAAACCCCCCCCCAACCAAAAAAACTTAATACTAAACCAAGAAAAAACCAAAACCCAAAACAAACTTGATTTTCTATGATAAAAAGTCCTCGAATTAAGGATATTTTCTTTTAGTAAAAACATGATCAAAAACTCAAAAAAACCATACCAAAACAAATACAATATATGGTTCATATAAACTTAAACCGAACAACCGAATCGATTGAAAAAACCTTGAATCTCACGACTTCAATCTTGTCTTATGTTATCTAAAGGAGGAGGGAGCTAAGTGGAAGTGTTCAGATTCCAAAGATCATCCTCCCAGACAACATCTTCGAACAATACTCCATAATTTTTCTCAACAAACTCCACGTTCCAACATGAGGGCAACACGGTGCTCCATGTC

>TCONS_00076342

CACTACTGTGTTCAGTCATTCGCAGACCGTTGTTGTATGCGGAAACTGCCAGACAGTTCTGTGCCAGCCTACAGGAGGAAAGGCAAGGCTCACGGAAGGATGCTCTTTCAGGAAGAAGTGAGATGATTTGAAGCTTTAATCAATCATTAAATCAGTCATGGCCGGTTTCTTTTTGTTTTTGTTTTTTTGTTTGGATGTTGTGGATCTTGCTTATCTGTTTTGAAAAGCAACTCGTTGGAGTTTAGAGGAAGAAACCTTTGTTGATTTTGGATTTTAATGTTATTACCATTTTAAAGGTTCTTTGTTTCGAGATTGTCTCTGGAGATGTGAAATGCCAAGGCTGCTTCAACATGTTCTTCAAAACGATATCGATCTGCTTAACCCACCAGCTGAGCTCGAGAAGAGGAAGCACAAGCTCAAGCGTCTTGTTCAATCCCCCAACTCATTTTTCATGACTCTATCGCTTATTGTTGTCGCTGCCTGCTATCTCCGCCGTCTCTTGAGATTCTTGCGAAGATG

>TCONS_00030424

TGGAAATACTGATCTTACATCGAAAGATCAAAGTAATACAGAAACTGTAATCTGCATATTGCCAGAAAGATACATACATATCTCTGATACTTTGCCTACGGTGAGAAACAACAAGAGACACAACAGAAGAAAGACGAACCTAAAAGTAAACCGGCAACTAACACAGGAGATACCCAAAGACACAATTAAAAAAAGAAAGCCTATGAACAAGTACTCTATACAAACACTAAACTCGAAGAGAAACTAAGACCTGTTAAGAAGATATTTCGACCTTGCCCCTTGAAAAAATAAGGGAAGATTCGAAACGGTTTCATAACTGAAGATGCTGTTTCTTCACCTTCTCGATTTCATCGTCTGTAGATGATTT

>TCONS_00042305

ATATTACATAGTTGTTCACACATCCTTAGAGATTACATTATAAGTTCACACTAAAGCAACATTGTAATCCTTAGAGATACACAATCTTATCATTTGGCCAAGCCACGGTGCTTCCTACTGCATCACCTATGAATTCGATTTCAGAGTTTGGCCTCCACACTCGAGCCCCATCGAACTCGGAAACCTCTACCCAAACCTTCGTAGCATTGGGACCTAAAGGAACGAAATGCACTTTCTCTGCTGGATCAGTTGAGAACACACGACCTAAAGCAACTCTCTGTCCTAAGTTATGACAGTCCAAGAGAGTAACCTTCTGTTTGCCACCTTTGTTGCTACTACTAGTGCTGTTACCACCACTCTAAAACCAAAAAAGACAAACACATTCACCACAATATCAACACCGGCAACCTAACAATTCAATATCTAATCACCTTCTAAGAATTTACAAACTTACCTGATTGGATATCTTGTTTGATTCAGTAGGTGAATGTTGATCAGTAGGTGAATTTTGATCAGGTAGTCTTATCTTATCTAGAGGCCACGCTATTGTCCCATGTACTGCATCACCCATCTTTGTCATTTCAGGTGTAGGTCTCCAGATATAGGCATTAGGTTTAAAAACCATTTCGACTCTAAGAACTGCTGCATTCGGTCCCAGTGGAATTTGGTTGACG

>TCONS_00032304

TTTTCTTTCTCAGATTTAGGGTTTCTCTCAGTTTCGGCTCGGTCCCAGAACCAAGATCGCAGGGCTTCAAGATGCCGTCGCACAAGTCATTCATGATCAAGAAGAAGCTTGCCAAGAAAATGAGGCAGAACAGGCCAATCCCTCACTGGATTCGTCTTCGCACTGACAACACCATCAGGTACAACGCAAAGCGTAGACACTGGCGCAGAACCAAGCTTGGATTCTAAGCCGTCTTACTTTTGCC

>TCONS_00025887

GTTTAATATGTCTCTTTATTTAAGTCATATAAGGTGCAAGCAAATCAAACGGGTCTAGAAGAGCCTACAACGGACCAAAGTAAATGTGTTGAGAAACAAACTTGGATTACAACTTGAGAAAATCCAAAATAGTAAATGCTGGTTTAACTTGCTAATTCCCTTGCTTGAAAAGAATGGATGCAATACCTCCCCTCCATCCGAAAACCGCTGCTGGCTCAGGCCTTTTGTGTAAAAGCGATGCGAGACCGACTGATACGCCCTGACCAGGC

>TCONS_00060598

GTTATGGCTATACACACTTGAGTTGTCACATCCTTTATGCCAATCTTATTTTGACAAACTCTAGTGGTAGACCACTCCCAAAACCCTTCCCTCCTTTTAAGCTTTCATTGTTTGATGAGTGAGGCCTTTTTCGGAAAGTCTTACATGTGCATAATGTTGAGAGTATCGGGAACGACAATGCTTGATCTTCATTCTTGCTAGATTGGGCACTCTATTGTCTAGCTATAGGTGGGGGGTGAGTGTTGTGATTATGATTTGGGAGCAATGAAAAAGGAAAAGAAATGACTCTTTGTGTTTAAGTGAATTGTCTTATTGGGATAAGTAGAAAAACCTCTAGCTCAAGTTATGAAAAGTCTTGGACCCCAC

>TCONS_00083666

CCGCCATCGCGACCTCTCCCTCTCTTTTTAATAGGGAGCAACTCTTTTGTGTAGCTCTGTCTCTCCTATCTCCTTACAACTGCTCTTTCGTCCTCTGTGTGTTTTCTTTCGTTTCTTTTTTGCAATAATTCCTGATGAGGATAACATATCTAAAGCTATGGATCTGATATTTCAGATTCATATGATGCTATATTGTCATAGTTACTCTGATGGGAAATTATTTGTGTTCTTTTTTCTTGTGAAACATTGAATCTCTGACATAGTTAGGTTGACATTCAAAATATAGGGCAACCTTTTGCCGAGGAGATTATGAGATTGAATCTGTTGATCTACCTCTTTTTCCCTGTTGCCAATATAATTTCATATGCATTATTACATAAGCTTGCTTTATAGTTTAAGACCAATTCCTATAAGCTTTTATGTTTTTGCTACAGAACAAAATAATTTTTTTAGTATTGTTTTGAATACAAGAGGCAACTTTTTGCCGAGTGAATCGAATATCGATCTCTCTCTTTTTCCCAGTTGCCTAAATCATGTTATGGCCGCCTGAGAGGAATTCGCTGATTGGTTATGTAATTTCAAAGTATCCAATCTTTTCCTCTTTGCTGCTACATTTTGTTTTGCTCCAATTACTCAATCTACTATTCATTGTGAATTCATAAATTTTTCCTATGATACAAGATAAAACGTTTCCTGATAACTTTACGATTTTGAGATTGAGCACGTGACGAGACCACTACTTTGGGTTCATAGGCATAGTTCATCTAGTTAAGAACATGATCAAGATATGTTCTCATTTGGACTGAATAATTCCCAATGGCCAAAATAATTAGGAGAATTAATTAAAAGGTATTAATTAATTCTAAACGTATTGTAATCTGAACCAAAAATATGTTTTGGAACTTTTATACACAATAATAACTTAGAAATGTTTATCTTTGGTATGTAATAAAAGAAGATTAACTCAAATAACTGAACATGGCGATATTCAAATAACTGAAC

>TCONS_00038129

ATGAAAGGCTAGATGCTGATTCATGTTTGTGAGGAAGATACTTAAGGCGTGAGCAGGAGCCAAAGTAATCCTTGTCATGGCACAATCAAACTTTTCCAGATAGATGTCGATGGAGTCGTTTGCTTGTTTTAGACTGACTAATTCAGAGAGAGGATCATCGAAAAGTTCGCTGAAGCGTTCCGATACAGCAGAGACATAGTCGGGCCATAGTGGAAATATGTTGTAGCGGTTGGCTATGTAGGCGTGATGCCATTGAAGAGCCTTGCCGGTCATATGAAGGGAGGCGAGACGAACCTTCAATTCTGGTGGAGTACTATCGATCGAAAAGAATTGTTCGCAACGATAAATCCATTCACGAAGTGCTGTTCCATCAAACATTGGGAACCCTATTTTTGTTAACCGTGACGAGAGTGTATTGGGGAGCTGTGGAGTAGCGTTTTGGTCGGAGTTCATTGGTGTTGTTTTTGCTGTACTCTGTTCTCCTGTGAAGCGATCTGGTGGGTCTGGTTTCTGGGATTTGTCAGCAGCTGTGAGGCCGGAACTTCCCTCTTGTTGTGTTGACTGCGAATTGCTATTAAGGAATTTTTCCATCATAGCTTTAAGTTCGGCGATTTCTCCTCCAAGATCCTTGCGTATTTCCTCTGATTGTTGAGTTTGTGATAAACGCAAGTCGTCAAGGTTTTTGTTCATCTCGGCCAGCGCGGTGGTTTGCCTTGTGTCCAT

>TCONS_00009898

ATATCAAATCCCAAAGAAATAATTATATATTTTTCACTTACCTGGTAATACAAAAGGATCATATTTTGCATATTGTCTTCGTGGTGAGACATCAACAAGACCAAATGGATGTATTCTCATACCGCGATTTGTTGTGTTGTCAAACCACGAACATTTAAAAACCATGACTTTCAAGCCAACATCACCATGATACTCTACCATCATGATCTCTTGTACAAGACCATAGTAGTCTGTCTCGTTGGTTCCAGGTACACATACACCATAATGTTGAGTTCTCTTATTTTGACCATGGTTATGAGTGTGGAAAGTGTATCCACGTGTGTGGTATATTGGCCAAGACCTGTAACTACGCCTTGGACCTTGTACAAAATCCAACATCCATATAGGAAATGTGTAAAACTGAGTCGCATCATTAATCTGTAATACAATAACATTGTTATAAAATACAAATTTAATTAAAAAAATTGTGAAACTTTATAATATAAAACAATAACTCACGTAGTCCTTGCACCAATCAGCAAATTTGGTGTCTTTCGCTTTTTGCATTGCAACTGGAGTAATATCAGGAACATTTAACGTCATATATTCTTCAAACATCCTGCACATAGACAAATCATAGTTATGTAACTAGAAATATGTATTTATATTATATTCTTCATAATATTAAATTACCTTTCATATGGAGCAAATGTTTCACAGTTGAGCATCATAAATGTCTGAAGAATAGTGTTATCTTCATCATTCAACCAACCAGTTGAGCATTGACCACTAATTCTCCCTTCATGGTAAAACAAGGGGGGTACATCCGGATAATTGTATGTGAAACGAATATCATTCGGACCTTCTGGAATGCTCATGATTTCAGGATGCCCAAAAAAATTTGAGGAAGCATTAGAAATCTCCTCATTTATCCATTGTGCAACTATTGAACCTGCAATATGTGCTTTGTTTTTGACCATCTGCTTCAAATGATACATGTATCTCTCAAATACATACATCCACCTAAAATGGACAGGACCACCTAAGGCTACTTCATCTGGGAGGTGCACAAGAAGATGTTCCATAACATCGAAGAACGATGGAAGAAATATTTTTTCCAAGTTACAAAGCTTCACACCGACATTTGCCTTTAATAGGCC

>TCONS_00046554

AGGGCATACAACCTCGATTATACTTATGTTCATATATATATAACATAGTTTACGATATTTTCAATACTTAAAGTACTTCTTTTGAATATATCACATAAATCTCGGGATATCTTAGAACCTCTGGATATCTCACAAGAAATTGATAAGCTCGTCATCATATGTGTTTATTTGCGATTATGTATCGGTCTTTTCTTGTTCGCACTATTGTAATCCATGTCAACCAATCCATCACCATCATTTCCTCCTCCGTATTGCCATGAGTTCCGATTCTCTTGATCCTTC

>TCONS_00018241

GATGAAATGAAAACAAACCAGCCAAAGTGGCTCTGATACCAACTGATAGAACCCTAAGATAGGGAGGATCACTTTAGCTGGGTGTTGGATATGATCCGAGAAGGCAAACGGCACTTCTGGAACTGAGATGGATTGAAAGGATCGTCAAGAGATGCGGAATGGCTCTTGATATACCCACGATCTAAGGCTAACCACCTAAGAACAAATGTAGTGTGTTGGTTAACCACCAAACGACACACAAAGATGATTGGCTGACCACCAAATCAACAAGCCGGTTCAAACCGATGAACTAGCTAAAGAACTCTCTCTCTAACTCAGAGAAGAAACTAAATAAACTCAAAAACATAGACTGATTTTATTCATGGCAGAGTTTACTTATTTATAGTACAATGAGTCAAAGAAAGACTTAAGAGTTTTGTAGAAAACTAGAAACTTAGAAAACATAAACAAAGACTAAGACTTTTGACTAGAAATAAGTGTGTTGTTGAATGGCTCTTGGATGATCACGACTTTGACTCCTTTTGTTGATATTTCTTGATGCAAATGGGCAGGAGACCATCAGCAAAGGCCTGGTCGAATTTGGTGTAAAACCGACCCAAATTGAATTTGTTATGAATTTTTCTTTGGGCTGAAAAAGGTCCATTTCGCCCAGCAACTAAACCAGCTCCATCTTCAGTGTCACTTAGCTCATTCAGCTCCAAGCTAGTGTCACTTAGCTCACTCAGCTCATCCAGCTCAGATATGTAAGTGTCACTTCGATTCAGCTCGACCAGCTCGCCCAAATAAGTGTCACTTCGTCCAGCTCGTC

>TCONS_00049526

GATGGATATAAGGTTTTTAATCCTTAATAGCTGTGGGATGCTGTACTGTTACTGAAAAGCTATGGGAGAAGGGCTTCCATCAGGAGGACAGCGTGTTTAGGAAGGCCGTAGGGAAAACGAAAAAGAGGGTGGTGAAGTAACAGAGAATCTCCTGGACATTGACCCGCTAGGAAACATGGTTAGAAATGATATTCAAGTGTAGAATAATAAAGAAAGAAAAACTTCAAAGGATAAGGTAAAACCCCATCTTTACATTTTTCCATCTAATTTTGTTTGATGAAAATTTGATCCATAGGTTTCTTTGTTTGAATATATTTGAATTAATGGCAGGATCGGA

>TCONS_00019946

CCCACGAGCTGCCCAACCGCACCAGAAGCAGCCGATACATAGACGGTCTCTCCTTTCTTAGGTGAACATACTTCATGAAATCCAGCATAGGCAGTCATACCGGGCATACCGAGAAGTCCAGTGTAGTAGGATAATGGAACATCGGTATCATGGATCTTGAAATGCATTTCAGGAGTAACAACACTGTACTCCTCCCACCCAACGAT

>TCONS_00035536

CTTTTGCTAAACAATCAGCAGGCATGTTGGACTCACGAAAAACAAAAGATACATTATAATAAAAAAATAAGGAAGAAACAGACAGAGAAAACGTCCGACATCACTCGTGTAGTTCTGACGATCGATAAGCCGATGATAAAGCTCTGACGAGCACTTGGGAATCTAAGCAGAGACAAATATGAGTAAGCGGAGGTGGATAGCGTGGTGAAGGGCAGCTCTGATGGCGAGAGCCTCTGCTATGCAAGATGAGGAGACATGATTCTGAGATTGAGAGCCCGGGTTCAGTTCGTGTGAAGATTGATCTGTAAAGATCCAACCTAAGCATGTTGCTCTGAATGCCATGCCGCATCGGTGTTGCAGAATATTGTTTGCGAGTGGAGATTCTGTGATGGAGGAGGACGAGGACGCATCACCATGACAGTGGCTTTATTAGGCTGAACAAGCTCCCACTCCGTTAAGGAGATTAACGCTTTCAAGATTGTTTCCGCTGGAATGGAGGTCTTGTTTAGGAATAAGATCTTGTTTCTAGAGATCCATATAAACCAATAAATTCACAGAAATAGTTTGGAGGTGACGCTGTAAAGTGGGAGATTGGCCATGGAGTGAGACGATTGGGGATCCGTTTTGAAAGACAGACAAG

>TCONS_00054940

TATTCTAAATTGTCATTAACACTAGTGGCCCACTTGATTTGAAGCTTTGGAAAATATCACCGTCGGTTTATTGTAAAACCGGTCGGTATATTAGTTTCTTTGAAATGAACCAAGTATAGATCCTATGAATTAAAGTTTAAAACATACAAGTACAAATCTTCGAGAATCTAAGGTATGGGAGAAAAGGATGGTAAAGCATAATAATTCAATAGTAAAGCATAATAATTCAAGAGACGAAAGAGAAAGTTCTGTCGGCAACCTCTCATTGTTTCACAGATCACCACATAACTTGCTTTTGGTTTTGATTGGTCCACTCAATTCCTTTTATCCATTTAACAAAAATAAAAAAAACAC

>TCONS_00038840

AAAAAAAGTAAAACCCAACGACAACTCTCTTTCGCGTGACCCCAAGAACCCTAAATCCCCAAATTGTGAAATCTTCAAACTCTTGATTCTCTTTCTTTTTAACATGATTGCTCATCAATTTGGTCATGTACCCGCTCTACTCTACCATTAAAACATTCGATTCACATAGGATAAGTGAATCCGAAACCCAGTCGACGAACCCTAAATCCCCTTTCTGAAATTCACAGTTCCGTCGATCTCAAGCCTTTCATCACTTATTCTCGATGATTTCCACACTATTATAAGCTCATATCCGCTTCAATCATCCATTCATGCAATCTAAGAAGCAATCTTCTGGAAATATACAATCTATCTTTGGACCACAAAGGAATCGCTGGACAGTATCATCATAGTTTTTTTTTGGGAAAGAGCTGCAAGTTCCATTGAATACTGATGATGTCTCTGGTATGGTGTGTCTTGTAAAAATGACTGCGTAGAGAAATATAGGAGATACTGGTGAGATGGTTCCTGCAAGGCTGAGGAAAAAAAGTTCACATCAGCTAAAGATTTCAAAACTCAAAAGTGTGCCTGGAATGAGGATCCTGGTGACTGTGGTGTTTAAGCCATAAAGTATATTGAGAGTAAGGCGATTGGTTGTGGTTTTGAGGACTTTTTGACCGGTGCATCCCGACAACGCGTATGAAGTTAGCTGCTGAGATCTATGATGAGGTTGCGGATATGTAGTTGTATTTCATGTTTTAGAAACCAAACTTATTTACTACTTTGTGGAATTGTGAACATATCTTCATTTTCTTGTCCAATGGTTAAT

>TCONS_00005512

TGACGATTGATATTAGGTCTCGTTCGTCGTAATATCGGCCTTAAAAAGCCGATTTGCCGACCATTCTGTTATATCACCACCCATATTCTGAGTCCTTTTTTGTATGTTTCTTATCATTCTCTTTTATTTGGCCGATGATTAAATCAATTAAGACAAGCATATGTCTGAATTAATCCCTGTTGATAAGCCTAATCTTCTCAATGAGGCCGATTTATTAGTTCTTTTGTTGTTTTATTTAGTAGGAGGAAGGGTCAAGTGATGAATAATAATGCATAGTTCAGATGAAAATAGTCTCCATGATTACAGTTTTGTAGCATAGTCTTTCGCTCCTATCTGATGACCCTCTCCTATCTTGTTTTCTCTGCTTTTGCCTACGTAAATCTTTGTTAAATTCTAATCTTCTATATTGTCAAACTGATAATGATGTTGTTTTGGTTAACTCCTTCCAAGATTCCGAAAGAGTTAAGACTGCAAGCCCTAACAAAGATGACGATCCAATGCAATATATTCAAGTGAAGTTGCAACAAACTTGAAAGTGCTCAATTTGCTGTAATAGATAAACTTGTTAATGCTTGAAAATATTCTGAAGTGGCTTCGTTTCTTTGGCTTAAACCAAAATTATGTATGCAATTTGCGGTGAGCCTGTTCTTGTGACGATCACAAGTTGATTTATATATGTATCTTTAAGAGGAGTGTACAAAGCTCGTGAGGTTTTGACATATCTTCGTTCTTGTTCTCCAGTACTTGTCAATTCCATTGGAC

>TCONS_00029838

TGCATTTGGAGCTCAAAAAGGAGTGTTTAGAGTGGTCATTGGGCGAGCAAGGCATGGGAGCGACCAGTCCGGAGCGACACCACCAAGTCGCTCTGATCACCCTACTGGAGCGACCTAACCAGAGCGACAGGGAGAAGTCGCTCGCGGTTTCATCACTCGGAGACGCGAGAACGAGCCCGGAGCGACCTCTCGGAGCGACACAGCGAGGTCGCTCCAGCTGGGAGCGACGTTATGGGAGCGACAGGGAGAAGTCGCTCGCGGTTTCACAAAGCCCGCTTCACACTGAGCAACAGCAGCAAAGATCTTCCACCACCATGTGGTTTGTGTTCAGCCAACTGTAGATAAGACAAAAACGATCAAAATCCATCCGAAGTATTTATCAAATGATCTTGTGGAGCGGAAAGAGAAAGGCAAAAAGACAAATAAAATCTACAGTTTTATAAAAAATAATCAAAAGCTACTAAATATAAGTTTTATTAGGATTTGTGAGAATGATGTTCAAGGGAATGATGCTAACCTTTATATAGTGTAGAAACACCGCCTTGCAGAGGGTAAACTCGCATTCAGTGTCAGGTCATGTCTCACTTCGGTCTGTGGAGTTAAGAAGACGATAAAGGATGGATCTGTAATCCGGAGCGTTTAAGCTGATTAGGAGAGTAACCAGAACGACGCCATGGAAGATCAAGAAAGATATACCTCGAATCATTTAGGGGAAATATTTCGTCTACGCAAAGAGAAGACATAACGCCATATCGAAGATTCTTAGAGCCATGATTAACATGGATTGCAAACATTGAAACTCCATCGATGTATCAAAG

>TCONS_00064626

CTGCAATCTAGGCCTACTGCAATCTCTTTTTTCTTTTTAAGAATTTAACTTTTCAAAAATAATTGTTTCCAAAATTAAATTTTAGTTTAAGTGAATACATGTGTTAAGGAGAAGAAAAAGGAGAAACAAGCATGGAAGTGGTTCAGCGAGTCGAGTTGGAGAGAGTCGATCCGACACGAGTCAGGTGAAGCTCGCCACGATGGAGAGAGTCTTACCAGGACAGGTGAAGCTCGTCACAGTGGCTATAATTTAATCAGACGAGGAGCTTGAGGCAAACGATTTGGCCGGAAAGTCGTCCGATAAGGTAGTGGAGATGATGCGGCAGATTTGGCTGAAGAGTAAACCATCGACGTCATGGGGAACATAGTCAGACTCCGACTCTGCTTTCCGGCGTCGTGACCTTGCTTCTCGCCGTCGCGAGCAGCTTGAGAGCATCAATGGCCAATTTTTGTTTTTTTGCACGTTCAGTCCTTTACTTTTTATATATTTTCGTTTTTGACTCCTAACTATTTAAATATACAATATGTTCT

>TCONS_00032629

AGACGACTCATCACGACTGTAACAGAAAACTCGCTGGAGAAGCCACCATGTGCGCAGATGACTGCCGTCCTCTCGGTTTCCTCTTAGGTCTCCCTTTCGCCTTCTTATCTCTCCTTCTCTCCATCGTCGGTGTCATCATCTGGATCGTCGGATTGTTGCTGTCGTGCATATGTCCGTGTTGCTTATGCGTGACGATACTGGTGGAGATGGCGTTAGGGTTGATCAAAGCCCCAATTCATGTTATGGAGTGGTTCACTTCCACCATACCTTGTTGATCTTCTTCTTCTTCTTTCATGTACATGAATAATCAATCTTGTTATTGCATTTTCTTTATTGTATTTGTTTTTGTTTGAGAATAAGTAAC

>TCONS_00030018

ATGGCTCTGGATATATTTCGAGAGCTTCCTATGTGCCATGAATGGGAAAACCAAGACAATGATGAGAACCTGGCAACGTATGATTAGATATTGGATGTGTTATTGCGATTGTATTTGAACTTGATGCTTTTGTGTACTGTTGTTAGGTAGATTAGGGGATGTTAACAGGGTCAGGATAGGATGATGTTTTTTGTAACCTATCCTCACACCAAACTAGAATTCCGTAGGAAATTAGTTTGGTAGTGCAGTAACCTTTCGGAATATGTAATGTTTAACTTGTATTTTTTAAATTGCACTGTTGGAATTAGTTTCGGAATATGTAATGCTTAACATAGAAGGTCTACAAAGAAAAGTTCATAAAACATATAAATTCATAACATAGAGTCTACAGAGAAGTTCATAAAACATAGAAAATCATTGGACGACTTACTTAAAGGTCTTCTGGACGACTACAGAGAAGTTCATAAAACATAGAAAATCATTGGACGACTTACTTAAAG

>TCONS_00052339

GTCTGGGTAACCAGACCGGAATAGAACAACCAATAAAAAGTAACTCTCTCCGAAATGTCCTAGCAGTCTTAGCTAAAAAGTCTGAAAACTGATTGCGCACTCGTGGAACATGTATGATTTTGAAGTCCGGAAAGCAAATCTGCAGCGTCTCTATCTTCTCCAATTCTGTCGCAAATCTTGGCCATTTTTGGGGTTCATTTATCATAGCAATCAGCTCCTTGCAGTCTGTTCCAAAGCTCTGACATGGCGAGTGTTGAAGCATGTTCTCCATCGCGCAGTGCAGTGCTTCTATCTCAGAATGCAATGCTGATTCACATCGACTGAAGTTTCGTGTACCCAAAAGTTGTATGTTCTCCCCGCTATCCATCCAAGCCCATCCGCATCCACTGAATCTATCGGAAGCTGTCCATGATCCATCCAGGAGGCAAATATTACCCAAGCTTAAGACTTGGTTTTCCTCATTGTTGTTGTCTTGCACTACCGGTGGTATCAACTCGTTTGCACTAAACCAGGCTTGGCATTCGCTTTCTGCATATCGGACTAGCTCCAAAGGATCTCTGTCTATTCCCCTGAAGAGCTTATCATTACGAGCCTTCCATATATACCATATTATCCAGGGATAAGGATCTCTGTCTTGGTCTAATGCTATGATATTCTTTTTCCTCTAGAATAGATAGTCCATGTTTGTGTAGACACTTGACGCTGGAAATATGCTTGGGCTTGTAGGAGTTGTCGATAAGGACCATGCTTGCAGAGCAGGAGGGCATTCAAATATGGCATGAGTTACAGATTCCTCTGCCTCCCCACACCTCGGACAGTAGTTATCACAACGCATATTTCTTCTCACTAGATTTCTCGTTACTGCCATCTGACCCGTTATCACCTGCCATATTAAATGACATATCTTCCTAGGCGCTTTCAACTTCCAAGCAAAGGCTTGAAGTTTAGTGATACTTGGTTCCAGTATCAGCTTTTCCTCATCTAACTTCAGCAGATTTTGAGCAACCCAATATCCAGACTTAACTGTGTATTGTCCATTCTTCGTGTATTCCCAGCAGAAAGAATCTCGACGATGAGTAGAGCTTATGGCCATACTGCGAATGAGCGGTATATCACCCGGATGGACATAGTTCTCTAACGTCCCTTCCTCCCATTCCTTCGAGTCCTGGTTAATGAAATCACTAACTCTCATGTTCGGGTTCATCACAGGCGCTACAGGTATAGCTGGTCTAGCAGGTCTTGTTGGGATCCATGGATCCTCCCACACCCTAACTTCATAGCCAGAATGAATCTTCTGTCTGATCCCCAGGAGAAGTAGTTTCCGTGAGCCGGAGATGCTTGTCCACACATATGATGGACTGCTTGAAGAGACTGCTCGCAATGGCGAGGTCATCCTATAATATCTACCCTTCAACACTCTGGCAACGAGTGAATCAGGGTACTGTATCAATCTCCATAATTGTTTTGCCAGTAGTGCCAAATTGAACTCATGAATCAATCTGACCCCAATTCCTCCTTCCTCTTTTGGTAAACAAACTTTTTCCCATTTCGACCAGTGTATGTCTCTTTTAGGGGGATTCGAACTCCACCAGAATTGTGTAATGGCACTAGCGAGGTTCTCACAAATCTCTAATGGAAGCAAAAATGTCGACATGACGTATGTCGGAAGAACGAGTAGAATGGACTTGATCATTACCTCCTTCCCCCCTTTTGAGAGCCATCTACCAGTCCACCCGTTTACTCTATGCATCAAGTTGTCCTTAAGAAATGCAAAAAGTTTACATTTGGATCCACTGATATCCTCTGGAATTCCTAAGTACTTCCCCATCCCACCATCATTATGAATTCCAAGTACAACTTTGATCTCTTGCCTAGTAGCTGCATTAATCTTCTTACCAAAGAGTAACGAGGATTTATCGAAATTAATACATTGTCCAGATGCTTTGCCATATGTCCTGACTGCTTTCATTACTTCTTCACATTCACGGGGCTCCGCCTTACAGAAGAAAAGGCTATCATCAGCAAAGAGAAGGTGGGATACCGAAGGACATGCGCGTGTGACGCGCATCCCCGTTATCTTCCCTTGGTTCTCTGCATGATTGAGAAGGCTAACGAGCACTTCCGTGCATATAATAAAAATGAAAGGAGACAAAGGATCTCCTTGTCGTAGACCTCTACCTGGGACAATGTTCCCTCTTGGTTGACCATTTATAAGAACCTTGTATTTTACCGACGTAATGCATCTCATAATCCAAGTGACCCATGTTTCAGAAAATCCCATCTTCCGCATCACAGCTTCGATAAACGACCATTCCATCCTATCATATGCTTTACTCATGTCTGTTTTGATGGTCATCCTCTTATTACGTCCAC

>TCONS_00018337

GAATTACTTCGGATACAAAGCAATATGAGCAAGTCGAAATAAATGGGAAACTTGATCGGCTAGAAGAACAAAACTCAGTGAAACAGTCGTTAATATCGGCTTACAAAAAGAACTTGTATCCACCTTTGAAAGAAGAATTTTCTAGAGCATTTCCACAAGATCAAACGGTCAATATGTCAAGATCAAAGACAAGATTAACAAGTAAGGTCATTAACGTTTTATAGCATAGCACTTCAATACTTTTTTGTTGGTTACAGTTGTAGTAACGATGTGTCATTTTTGGGTTCACATATAGATCGATGGCCAACGAGTTGGTGGATGCAATTCTCAGTTTTACTGAAGCGGGGATTAAAGGAGAGAAGTCACGAATCATTTTCAGGACTTAGAATATTTATGGTCATGTCGGTTTCTTTACTTTCTGGCCTCTTATGGTGGCATTCTCGCGTTGCTCATATACAAGATCAGGTAAAATAGTAGACAGTGATATTAAAATTTCGATTCTCCTTTTTTCATGTATATATATATTGAAGTAGTTGACATAACTTGTTAACCCATAAATCTCTTTTCAAATTACAAATACAAACATGATATGAAAAATGATATGTTTTGGTATTTTGAGTTTAGGTGATCATGATTTTAAAATGATGTTACTACTTTTTCTGTTTTCATATAAAAAATGAGGAATAGCTATTTTTGCTTAACTATAAAATGATGGATAATATATTCAGTACAACAACAAACTTTCAGTTTATCATTGCTTATAAACTTAAAATAACCTAAAAATATTTCAACTTCTTAAAACAAAATGATTTCAAGAAAACATCATTTATATATTGATATGTTGTAAATAGTCTTTTAACGAAAACTGGACATCGTCGTCAAAACTTATGTATTATTCTTGTTCATTAAAATAGGTGGGTCTATTATTCTTCTTCTCGATATTCTGGGGATTCCTTCCTCTCTTCAACGCTATTTTTACATTTCCTCAAGAACGTCCAATGCTCATTAAAGAGCGGTCCTCAGGAATCTACAGACTCTCCTCTTACTACATAGCAAGAACAGTCGGCGATTTACCAATGGAACTCATTCTTCCAACGATCTTTGTCACGATCACATATTGGATGGGAGGTCTCAAGTCATCTTTAACCACATTCATCATGACCCTTATGATCGTTCTCTACAGTGTTCTAGTGTCTCAAGGCGTAGGGTTAGCTTTGGGAGCAATCTTGATGGATGCGAAAAAAGCAGCGACATTATCTTCTGTGTTAATGCTTGTGTTCTTACTAGCTGGAGGATACTATATCCAACATATACCGGGATTCATCGCGTGGTTGAAGTATATTTCTTTTAGCCATTATTGCTATAAGCTTCTTGTCGGAGTTCAATACACATGGGATGAGGTTTATGAATGTGGACCAGGGTTGCATTGTAGCGTTATGGATTATGAAGGGATTAAGAATTTGAGGTTAGGGAACATGATGTGGGATGTTTTGGCTCTTACCCTTATGTTGCTTCTTTATAGGTTTCTAGCTTACGTAGCTCTAAGGAACTTGTGACGCATTCTAATTTGATTTTCTTGGTACTATGTATAAGGTAAGTAAGTGTAAGATATCTATAACCTTCTTTTTTTGTTAAGTATGTGAATTTCATTTTCAAGATAATGAGACAAGGTCCCATAATAGTCCCCACTTATTTTCCTTTATAAACACATCACATTACATGTGAACTTCTCATCTATACACACCAAATATTTCTCTCTATTTTCTCTGTGTGATCTCCCTCTAGATGATGCCTCCTAATGAGCAAGAATCTATCTTTCCGATAATACCGGGGGAAAGCCGAAACGAAACCATCCCGGTTCAAGAAACCTGGTTTAGTTCTCCAAGTCATGTAATCCCATGCCTTGACGACGACGGTCCGAGTCACCAATCATGCCAATCTTCTGTTCTACGACAATCTTTACGTCCTATAATTCTCAAGGTCGTTAAATTTCTATACAATCTATTTATTTTTCTTTCATTTACGTATTAAAAAAATAATTATTTATATGATTTGTGTACGTAAATTCAGTTTGAGGAGTTAACGTACACAATTAAAATACAAAGCGGGAAAGGAAGCTATTGGTTCGGTTCACAAGAACCAAAACAGAACCGGTTAATTCTTAACGGAGTAAGCGGTATAGTCAAACCAGGTGAGCTACTAGCAATGCTAGGTCCATCAGGGAGCGGCAAAACAACGCTAGTAACGGCGTTAGCCGGACGTTTACAAGGGAAGCTTTCAGGAACCGTTAGTTATAACGGAGCTCCGTTTACAAGCTCCGTGAAACGGAGAACGGGATTCGTTACACAAGACGACGTTCTCTACCCACACTTAACGGTGATGGAGACGCTAACGTACACAGCTTTGCTCCGTTTACCTAAAGAACTGACCCGGAAAGAAAAAATCCAGCAGGCGGAGTCGGTTATTTCGGATCTTGGGTTGACCCGGTGTTGTAACAGCGTGATCGGAGGCGGGTTGATCCGAGGGATTTCGGGTGGGGAAAGAAAACGGGTTAGCATCGGTCAAGAACTGCTCGTGAATCCGAGTTTGTTACTTCTTGATGAGCCAACGTCGGGGCTTGATTCGACCACGGCGGCGCGTATAGTCGCAACGTTGAGATCGCTGGCGCGTGGAGGTAGGACAGTAGTGACCACCATTCATCAGCCGTCGAGTAGGCTTTATCGGATGTTCGATAAAGTGTTGGTTTTGTCGGATGGAAGTCCGATTTATAGTGGGGATTCGGGTCGGGTCATGGAGTATTTTGGTTCAATTGGGTTTCAACCCGGATCCAGCTTCGTTAACCCGGCTGATTTCGTGCTTGATCTTGCTAACG

>TCONS_00043829

GAGAGGGTTGTCAAAACATTACAAAGGTAAATCTCAGTCATTCACATCCTTGTCGGCAGCGTTAACAGTAGGGGCTCTCGCAAAACCCGAGAATCCTTTCAACGTTAAACTGAAGCAGCGACGGGGGAACACTCACTGTCGACGGCTCTCCGGATGTGGAGGTGCATCGGAGCAAAACTTAGGCGTGCACGACGCTTTCCACTCCGGAAACGGTAGGCCGCCGAGACTTTCCGGTAACAGAGCGCCTCCGAGAGCCCAGACGCTCTCGGCTGCTCATATATCGGCTTTGCTCACTCGAACCTAAACCGGAAAGTTTTTAGCATTTTTCTTTCTCTAAACCGGAACCGGGTTCCGTCTGGGACGATGTTTTCCTAGCTTCCGTCTTCTTAATCGTGTGAATGTAAAAGTTATGAAGTATGTTGACTCCTTTAATCGTTTTGTAAATTACAGTTAATTAAATTAACTCAAGCCTACACTTGCACATTTTTTTTTGTATAAAAGCTACTGTTTCTGCTCTCAAGTCTTGAAGCGGCTTTGAAGAAGAAAAAAACATGCACTATCAAGAACAGATGGAGTCTCTTATGTTGGGTGAAGAACGCAGACGTGGAAACTGCGTTAGGGACGCAGATGAAGGTTTTAACTCTCCGTCTTCTTTTCCTAACTCTCCTGACGATTCGGACCGTCGCAGTTCATCTTCTTTCAG

>TCONS_00032115

GACCAACAGTATGTCATCAATATAAATTAAGGCCGTATGAAGTAGAGGCTTGAATATACGAAGCATCGCTTGCTGGAATAATGATGGTGCGGTCTTGAGTCCGAAAGGTAAGACGTTCCACTGAAGATAACGATTAGGAAGACAGCATGCAGTTTTGTATCTTTTTTCAGGATGAACACCAAGTTGCCAGAATCCCGACTTAAGATCAAATTTTGAGAAAATAGTTTCCCCTTGAAGATGTTGAAGAAGCGTCATTTTGTTTGGTAGCGGAAATTTGATGTCTTGTAGATACTGATTCAGCGGTTTGTAGTCGATAACTAGTCTTAGCTTCCCTCTTGCTTGTTCTGCACGATTATTAACGTAAAAAGCTTTGCATGCCCATGGCGAGTTGGTTGCAGAGACTACTCCTTGTTGTACTAGCTGGTCGCATTCTTCATTCGCTTGTTGTAGTAGGGTTGGTGGCATTCCTGCATGACTTGCTTTAGTTGGGGTGGTTTGTATATTTTCTTTGAATGGCAAAGAAACAAAGAACTCCGCA

>TCONS_00083016

GCTTGCGAGACTTCCGGTCCAATGATGCTCCATGTAGCTTTGAAGAACTCTGAAGTATAGCCATCCGGACCGCTGGCCTTGTTGCTTGGGAGGGAGAAGAAAGCGTCTTTTATTTCCTGCGGTGTGAACTGCTTACAGAAGCCATCACTCACCTCGTTAGGGCAGGAGAAATCAAAGAGTAGGTCGAGGTCTGATTGTTCAAACTGCTGCGAGGATACAGGACCGCCCAGTAACTTAGAGAAATACTCGACGCAATGCTCCTCAATCTCTGATTGGTTTTCGATTCGTCCACCATTTTCGTCAGCTAGGAAATGAATATGGTTCGCCGCTCTCCTCGTGCTGGTTAATCGATGGTAGTAGGATGTACTTGCGTCCCCCAGACCTAACCAAGAAACTGAGGCTCTTTGCATGAGGAAACTCTCTTCCGCCTTGGACAGAACTTGCCATTTTCGG

>TCONS_00035764

ATACATGCTTGCGGCCGCCGTCATCGGGCTAGGTTACGCCGTTGTACAACTGGTCTTCACAATCTCCCAATTCGCCACCGGAACTATACATCCTTTTAACTATCTGTTCAGTTTTTATGGCGATAAGGTATTTAAAATTGATTTTTTAAATTTTGAGTAAATTGTGTTTCAAAAAAAGTGTTTCAAAAAAAATTGAGTAAATAATTATCTAGAAATAGAAAAATCATACAATAGAATGTTAGTAAATATGAAACAATTATATCTCTCGACCTCCTAGATTAATTAATTCTAAAATATAACATGTATTAAATATGATATATGTATGTTTTGGACATTTTTTATTAAAATAAATTTGTTTTTTCTTATATTAACATAATATCTCAATATGATAAGTTTCATGTTATATGTACAATATATTTAAAATCAATAAAATAATGTGTTGTAATATGTTTTTTTTTTGTTAAGATCTCAACTCATTAGTTCCATTATATCTAGTTTGTATATTTATGTTCTGTAAAACACAACGAGAGGACATACATATATTGCTTTTGAATCACATATTCTTGTTAATGATTGTTAAAATCTGGACAGATTATATCATATTTACTTGCAACGGGTTCGGCAGCTGGATTTGGAGTGAGCAAAGATTTGAAAGATACATACTTAGCATTAATAGATTTTGATTCAACAGATCCAGTGGATAACTTCTTCAGTAAAGGATACGCATCAGCCAGTCTTCTTCTCTTTGCTTTCGTCTCTCTTGCCGTTTTATCAGTTTTCTCATCTCTTGTTCTTTCCAAACGACCAATTCAAGGCTCATAAACACACGTATCATTTTAACCGTGTATGCTTTCTTCATTATGATTCGTGCATGCTTTCTCCCACCAATCGTGGAACTTGATCAGATTATATATATGCTTTTCAAGATGTATTGGTTACGTTACCAAAATAAGAGATTCTTAATTTTGACAAGAAGGTCTCATTGTTATTTCTGGTTTACTATTTCTGTAATGCCTTTTTGGGGGTTATCTAGTTATCTGTATTATTAATATGAAATATCACCCTGATGGCTCCACCACCTCCTTCACCGCCGTCCGTTATGTTGAGGACAGTGCTCCTCCTCCTCAGGGTTTTGACGGCGGCTTTCCTCGTTATAACGGTTGCCCTTGTCAGCACCAACACCGTCACCCTCGATATTCAAGGAACGTCTATAAAAATGCATTTTAACGATGTTTATGCTTATAG

>TCONS_00006210

ATGCTCCTAACCATCTCTATTAAAGTACGGTTCCGCCTCTCAGCTACACCATTCTGCTGAGGAGTACCAGGCATTGTGTATTGTGCACATATGCCCCTACTTTCAAGTAACTTTGCAAATGGACCAGGACATTGTCCACTTTCATCGAATTTTCCATAAAATTCACCACCTCTATCAGATCTCACTATTTTAACCTTTTTATCTAACTGCCTTTCCACCTCGTCAATGAATACCTCTAGGACATTCACAGACTTAGACTTTTCATGCAGTAGATAGGTATATCCATACCGTGAGTAATCATCAATAAATGTGATGAAATATTTTTCGCCACTCCATGAAGGAGCATCGAAAGGGCCACATATATCGGTGTGTATTAACTCAAGAAGCTGAGTGCTTCTTGTGGCTGGTTTCTTTACAGTATGTTTCGTCTGCTTTCCCTTAATGCAGTCTATGCATACATCTAAGTCACTGAAATCCAATTGAGGAAGAATTTCATTTTTTATTAACCTTTTAATTCTTTCTTTGGAAATATGACCTAGTCGTTGATGCCACAAGAAAGCAGAACTTTCATTAATTGCGCTACGCTTAATGCCTCGATTTTCAACATTAAATAGGGATTCAGAAAACTTTGCATCAAGGTTGAATTTATAAAGTGAATCAAATAAAATACCACTACCATAAAAGTAATCATTTCGGTACAATGTGAAAACACCATGTGCAACCTTAATACTAAATCCTAAATTGTCCAACCTACTAACAGAAACAAGATTTCTAGCACACTCAGGTACATAGAGACACCCTTCAAGATCCACATGACTTCCAGTGTCCAAGATCAATCTATACGTCCCAATTCCTTCTATTTGTGCCTTCATCCTGTTTCCCATGAACAAGTACTGTTCAGGTCCTCTTATGGGCTGGATCGAACTGAATCCCTGTTTAATATGAGACACATGAGTAGTAGCACCAGAATCTAACCACCAAGTATTATTAGGAACTTCAACAAGATTCATTTCATAGCAAACAAAGCTGTAATGTTTACCTTTCTTGTCGAACCAGTCCTTCCTTTTAGGACAATCCTTCTTGAAGTGTCCTACTTGCTTACAAAAGAAACACTTCTTTTCCTTCTGGATTTGACTTTGAGGTCCTTTCACGAAGTTTTTAACATTCCTTTTGTCCTTCTTACTTGATGTTCTTTTGCTGCTACTGGCACTTCCAAGACCTACGAGGTTAGCAACTTGATCTTTCATCTTCCTTAGTCTCCCCTCCTCTTGAATTAGCATAGCCTTTAATTCTTTAAAGTTCCATTTATCCTTAATGGTGTTGTAATTCACCTGGAACTGGCTAAATTCAAGAGGTAGAGAGTTCATGATGAATTGGACCAAGAATTGCTCATGTACCTCCATTCCCAAGGTCGTTAACTTTGCTGCCAGATTAGACATGTGCGTCAAATGATCATGGATTGGCTGAGACCAGTCAAACTTCTTTGTAGTTAGCTCATTCATGAGACTTCCTACAATCGACTTGTCAGCCAATTCCGATTGTGAACACTCCTTAATTTTCTCTATGAATTCCCTTGCTTTCTCTGTCTTAGGCATCGATGGCTTAATACTTTCTGCCATCGTCAACCTCATAAGGTTCAAGCTCAGCCTATTAGATCACTCCCATCTTTCATAACGAGACTTTCATATTCGGAGCTATCTACTGTAATAGCTGAGGGTTCCTCATCAGTTAGTATGGCGGAGTCCAAAGCCATTACACCCAGTGTAAACCGGATTTGCTCGAACCACTCACCATAGTTGAGGCCATTAAACTTTACCACAGAGTTAGCAGATGCAAACACATTTGATGAAGCTGCAAGCATTCATACATGTTTACCCGTTAGTGCTTTGAGACTTTAATATATATTCAGACTAATAGAAACTTTAACACTTATATTTTAAATAACC

>TCONS_00077837

GCTTCTTCCGTCTTCCTCAGAGCTTGTGACAATGCTTTTAAAACATCCGAATGGTTCGTCAACTGATCCGAGTCCGACCCACTTCTCCTACTATTGATCTGTTCCTTTAATCTACGGTCAAGATCTTTCTTCTCTCGATCCCACTCACATCTCCACCGTCCCTCACAATGATTAATATCTTCCTGACCGAGACACGAACCGTCATGATCCTGAGGTTTGGATTCGATGTTTGGATCGTCCCATAACAAACCTTTAAGCTCCCGATGAAGCGTGGGATAAAGATGAGAGAGTAAATAATCCGTCGCATCCGGACCGTTGAACCCGTCGTATATCCCGACAAAAAGCCAACCGTGCTCCTCCGAAACGACGACGTGTACTCGATCCTCGCCTGCTTTCCCTTGAGCCCACTGAAGATTCTGGCCCTCGAGCGAAACGTCGTCGTCCAACGTCCCCTCGCTGCTAAAGTTCAAGCTATTAACCGTGAGGTTATTCTCGTTGTGGTTCTTGCTGCTCTCGGATTCTTTAACGGACTTGATCGGAGCGACGATCGAGTTCTGCCCTCGAGAAACCGTCCTCGAGATCGCACGGCGGAGGATCCGAACCAAAGACCGTTTTCTCGACCCGACACGTAAAGCCAAACCGTGAGAGAAGCTACGCTGGAATTGATGATCGGAGATGGGCTTTTCAAGCGGGCCGGAGAATAGCCCGCCGCGGTCAAGCGGACCCGATGAGAACCCTCTCTCTATCGGACCCGAAAGGAACCCTCTTTCGATTGGACCGGAGCCCAAGGCGACCGGACCACCACCACCACCGCCGCCGCCGGAGCTTTTGGGGATGGGCTGGAGAGGGATGGAAGGAAATGACGTCGTGCTCTCGAACGCGGCGGCTCTGTCGATGTGGCCGTAGGGATCGTAGAGAGAGGTGGAGAGAGGAGTGGCGGCGTTGGCGCTGACGGAGGCGCCGGAGATGGTTCGGAACATAGTGGTCTCTTCCTCCGAATGGACTTTTGAGCAAGTGATGAGGGTCGGTTCG

>TCONS_00013627

TTTGAATTATTAATTAGATATAGTTTTTTTTTTTTTCTGTTTGATGGAAGGAAGATTGCCTCTTCTTTCAGCAGCAAGTTCTGTTCTTGTTAGCTTTGTTCTGGAGGCATATATCAATTCGCCATCACGAGAGGGATTTTATAATTTTGCAGAGTTATTCCTCTGTATAATATTATCCTTCTCTTTGGTACATTCATCTTCTTCTTCCAAATATTTATATCCATCTGACTTGCTTTGAAATGATTCATTTATTGGTAGATCGAAAACTTTCTTGCTTATTAGAAATTTTTATTCAGAACTATGCTTTGGTCATTCTTATAATATGTGGGGTTTACAGTTTAGATTAGTTGTGAAGACATTATCATCGTCTCAAAAAAAAAAATCGATTGATTGATACCATTCAATGGATTTAAAAGGATCCCATATTTGAAAATAATTTGCCTTACTACATACCAAGAAACATATTAAAAAACATTGACGAAAATGATAATGGAATGTGGGGATTCACATAATAAGTTTGAAATCATTTCATATGAATAACAACGATAATGATTTGACATGCTTAAGAGTGTTGATCTCAGTTATAACCGTGTTTCCCACATTGGACTAAGAATGAATGAATGATCATTAAGCAAATCTAACTAATCAGGCTTAGCATACTGTACATTGTGCCTCTGTCCTATCCGAATCTAACTGATCAGGCTTAGCATACTGTACATTGATCCATGAGCTATTACCTAGCCAGGAACAAGATCAGCAATGTAGATGGCTTACACAGATTGCTGGGGCAGCTAGTTTCCAACTACGCCTAATTCAGACAAACGTTGGTGAGGAGCAGCTGTGTAAGACCGTCTCTAGCATGCTCCCTAAGCTAGTTTACCTAAACAAGCAGGAAGCAACTCACTAAGCCACAACGAGCCAGGGAAGTGTTCAAAAACCGTCCCAAAAGCTGCTTTTGGTAGCGGAGATTCTCTGTCAAACCAACAGACACAGTAGAGTTGTTGGACTATCAGATTCCCTAGCGTCCATCAAGGAGGCCAAAGGCAGAAGCCAGCTTCAGCCGCAGAGACACCGCCACATTGACGTCTCATCTTTTTACACATGTTATAAATTGGCCAATGTAAAGAGAATGATCCTCTCAAAACAGTTGTCGTATTCTACTGAATAGAGTTTGTCAACTTATATTTCATAAAACATCATCTTCACAAATATGGATTATATTCTTTTTCACTGTATATAATAAAAATAGTAACCCCAAAGTAAATATAAATGTAACATTCTGTAAAATGGGAACTCATGAAAGAAACTTCACCGGCGGTCTTACGTCCCCCCGGCAGCATATATTTCATCTCCCTTCTCTTTTCATCATCTTCTTCTTCCCCTGTATCGCCACCACAGTTCTCTGACTGTGAGACTTGTTTACAAAAATAAAACAAACTCTTCTCCTTTACTTCCTCTTGTTTCAGATTGATTGTCTCTTGGTAAATGGTTTTTGGCAGATGAGAAGTCTCTGATCACTACTGCTTTCAACCTCTATAACTCTAGCCTCCC

>TCONS_00079213

TGTTATTCTAAGACCAACGATCGAGCCAGAATCCAAAACGGACGTACCAATTCTTCTCCAGTCCATCGCAACTCAATTCTGTACTGTACCGCACGTACTGCCCGTACGACCAGTTTGAGGCTTCATCAATACCCATGTCCAGACGATCGAATTCACCGAACTGGAGCTCGTATTTCCCGTACTGATTGGCATTTTAAGAGCAACGGTCAAGACAGATTTGGATTCGGACGAGTGGAACTCAAGATAGGCCGTGACACTTCAAAACTGGCTACTTTGGACTGTCCTGCTTGTGTACTGGCCCAGTCCGTAGGACATGCTTCAGGACACAATGAACCTGGACGGAATTTGAAGGGTTTTTCACCAGTCAAAGTGTGATTTCGTGCCTTGACCAGATCTGGTCAATTTTATCATTTTCTATGTTTAGATTTCCCACATTTTTCTTTAAGTCTTTTGACTAGAACTTCTATATAATGTAACCATCAGATCTGAATAAGATAAATTCGTTTTGCTTTCATTCTTTTGAATTTTTACTCTCTTTGTTCTTTGTTTAGAACACTTCTTAGTTTCTTGGTGAGGTTATCTCCAAGTTTCGATCCTTCTTTTGTTGGACCGGTGCGTCACATCCGGCAACGATCGAAGTCTGGTAGTATCTTTGGGCTATTCCGCAACCCTTAGTGTCA

>TCONS_00051131

AGACGCACCAGTCAGGTCACATGACCTAAAGACAAGCGTGTCATGGTCCAAAACGTGGTTAAGGGAATCAGGTAGCTGGAACTTAACCAAAATAAGGCAGAGGCAAGCTCAAAGGAGCTGGACAAGCGAGCTGGTAGCTGGACGAGATCCAGGTGAAGCTCGATGAAGTGGGTGATCAGAATGGATGATGGGAGAACTAAGTCTAGGTCTGGGAATAGACCAAGGGACTTAGAAGGATTGAAGAAGATAAAGAAAGCAAGCTGGACACAGTGTATAACAGCTGGGCGATGCAGTAAGCAAGCTCGACCAGCTAGGTGAAGTGTAGTGCAGCTCAATGTAGCTCACTGAAGTGTAGGTCAGCTCCCTGAGCTGGATGGTCTAGCTCACTCAGCTGGATCAGCTGGGGATCAGCTCAACTCATCTGGACTGAGTGTTCAAGTCATGGGCAGTTGGGCCGGGTCTGGACAGTGGCCGGGCCATGTGGGCGACCCGGGTGTACCGATGGGCTGGTTGGCTCTTGGGATTGAGCCAGGAGCTTGGGCAATCCGTGTGGGCTTGTTTGGACTCGTCCAGGAGTGGATTGGCAAACCCAGGGCGTCTGGAAACTGCCTGA

>TCONS_00032068

GTAGATCGAGTCGCATATAGAGTAGAATGATCACGCTATTGAATGGTAGAGTCCTTAGGGTTATTTTATTTATGGAACAGGATCGACCTTGGAGCTAGCTGGCAGTTGTGTTTACTGACCAGTAGCTGAGGTGATCTGACCAGACAAGTGTTAGATTGGATTTAGTCCAATGGATGATAGATGTTATTCCGCTGTGCATAAGTTGAAATGATCTAAGCTAGGGAATGACTAAGGTAGTCTTGAGCTAAGATCTGAGTTAGCCCTCGCCTATGGACGATGTGTTTTAAATAAAGGGCAAAATTTTTAGAGGTTCGGTCAGTGTCTAGACCGAGCGACGTGAGGCATCGACCGCGGCCTGGTCGGCCGGGATCGGGTCTTACACTCCCC

>TCONS_00027936

GCTAGACTATGAGGTAGCGGAGCTGACGTGGGAGAACGGGCAACTAGGGCTGCACGGCTTCTGTCCACCGCGGGTGCCTGCTCCGTCAAAATACTCCGTTGCTACAAATAGTTTACATAACAAAAAAAAAGAAACATTATAAGAAAGAGAACATCTCAACAACATAATATCCTCTAAATTTGATAATAGCAATATATACACAAGTCAGCTTCAGGATGAGCCAGTGTGTTCCAAACTACCACATCGATGATACTCCGGCGGCCACCGTCCGCTCCACTAAAGCTGCAGATATCCCCAC

>TCONS_00019116

CACAAAACACGGGAGAAATCGTGAGGAATAGAAAGCTATTGGTTGTGGAGAAGGAGCCGGAGACTCAGAACTCAAGGCAAGACGGAGGAAATGATGGTTATGGATTGGTGGATATGGATTATAATAGTGCGAACAAGAAAAGACCGATACACAACCGCTAATTAATAAACACATGTGATGAATTTATCAATTTCTTGTGAGATATCCCAAGATTCTATGATATCCCGAGATTTATATAATATATTCAACAGAAATACTATCAAGTAGCCAGAGAGAAATAAACTAAGTCACTTCTAAAAATGGCAGTTAGGGTTTATTACAAGCAACTCCTCCTGGCATTTCTTCTCATCATCTTAATGTATTCTCCTGCTCAAG

>TCONS_00034091

ATATTTTTGACGAGCTTTATTGATTTAAGTTCTTGAATTACAATCAAGATCCATGGCGGAAGAGCATCGATGTCAAACGCCGGAAGGCCACCGTCTCTGTGCTAACAACTGCGGCTTCCTCGGCAGCTCCGCAACCATGAATCTCTGCTCCAACTGCTACGGCGATCTCTGTCTTAAACAACAGCAACAAGGCTCATCCTCTCTCTCCGCCGTATCTCCTCCGTCGCCGGTGATCACTTCCATCTCTACTCCGATGATCCAGCCTCTCGTTCAAAACCCATCTGCTGAATTGGAGGTACCGGCGAAGAACGTGCCGGTGACGGTAACGGCGACGGAGCAGCCGCAGAAACGGCCGAACCGGTGTACCACGTGCAGGAAACGGGTCGGGTTGACCGGATTCAAGTGCCGGTGCGGGACGACTTTCTGTGGGGCCCACAGGTATCCGGAGGTCCATGGATGCACCTTCGATTTCAAATCGGCCGGTCGCGAAGAGATCGCGAAGGCGAACCCGTTGGTCAAAACGGCGAAGCTTCAGAAGATTTGACTTAGCTGTGTATGTATATATATATTTTTTTGAAAAAGCTGTGTATGTATATTTTCTCTTCCCAATATCATTGTTGTATGGTAAAGATAAAGTTTAGTGAGTCATCTTTCTTCTTCTCTCAAGACAG

>TCONS_00009953

ATTCTTGTCCCCGTTTTGTCTCTTCACAAAGAAGTGCTTCATCCTCTTGATCTAAAACTTCGACTTCCCAGCCATTCAACAAGGACAAAATGCGCAATTCTTTGTTAGGACAAACCTCAGCATGAGCTCTTGACCAGGGTGCTTTACATTTGAAGCAAATCTTAGCTCTTCTCATTGCGTCCAATTCTGCATCCGAGTGTTTCTGTCTTGGTCGTTGAGACGCTTGATTTTGCGTTGTACTCTCCTTCAGCTGCAATGCATCACTCTTCTGTTTGCCATCAGATTGCCATTTAGTAGCCACTGTCACTTGCTTCTCATTTGTGTTTTGACGTTTCTGGTGACTACCTGTTGAGTTTGATGCATTGCTGACTACCTTGCAAAACAAGCTCGTCTCCATTCTTAACACCGCTGAAATGTAACCTTGCAACCCTTGAGGATCCTTGATTCTTATCACCTCTTTCATCTCTTGACTCAATCCGTTGTAAAAAATCTGCTCCAACAAATGATCTGATAAACCAGGAACTTGAGCTGATAACGTTTCAAAAGCTGAAACGTATTCCGTAATTGATCCTATCTGTCTCAATGCAAACAACCTCGTCTCTGGCTCATCATCAATTGATTCGGAAAACCTTATCATCAACTTCTGCTTAAAATCCGTCCAACTTCGAAAACCTCCTCGACGTCTTACCCAGCCATACCACTTCTTCAAAGCTCCTTGTAGGCATAATGGAACCAGATCCATCTTAGCGTCATCACTATACCTCCCCAACGCGTAAAAATACTCAATATCTCCAATCCACTCTGAAACTCCCGTACCATCACAACAAGGCATTTCAACTTTCTTTAACATAGATTCACGATTCGCTAAATTCAATCCCTCAGCTCGATAACCTAGCGGAACTCCTCCATCTTGTTCCGTGAAAGAAGGATATACCTGAGGTGATCTTCGGATCGGCGCCGATGGTGAAGCACTCTCGTGAATCGGCATCTTCCCAATCGACTTCATCATCGCCTGGACTGAAGAATTCAACTCTTGAATTGTTGACTGAAGTGAATCCACTGTCGAATTAGTCGCAGCATTCTCCTTCGTCAGTCGATTCACGCTCTTCGCTAGATCGTAAACCTGCATCTTCCATTCCGC

>TCONS_00009692

GGATTATGCGATGTCGGTAGAATGACGGCTGACAACAAAGCAAGCAGGGCATACTTGATCCTCGTTTCTGTATCAGCAACCGTCTTCTTCTTTAGCATGCTAATGACATAACTGACAGGGACTTCCCTCATAGAGCCAAATAGCTCTCCCCAATAAGGCTTCTCTATTAGGTTCTTCGAGCTCTTTTTCTTTGTACGCGGCGGGAAGTTTCGACAATTGAGCCCTGTGACTAGAGCAAATTCTCTAATTGAGAATCTTACGGGTTTGCCAGCAAATATAAACCAGACTTCGTTCTTCTTGCCCACTTTCAACTGTCTTGATATAAGAAAACGGCCAAACCGACCGGAGTATGGGGGTTTATCTGCGATTTTGACAAGCTTTCCAAACGGGGTCTCCTTAATCCTCCGGACTTCATCAGGGCCGAGTGCGTTGAGGATTTTTCGAATGGATGTGGGTTTGTGGTAAGGTGTAACCCTAACTCCCTCGGGTTCTTCCCCTAACGCAAACATTCGGGGAGGAAGTGGTAGTGGAGGAAGAGATTCATCGCTGTGATCCATAGAAGGATTACTACGACCGAAGTGGAATCTAGATCTGTTTAGAAACTGATTGGAATGACAAGAACAGAGTATCGACGGTGAATAATATAGTAAAAAAAGATGGAGAGAATCACGGTTTTCGATACCAACTACCAAATAGGGAGGTTTCGAAAAATGCAAAACATCAACTTGCCTGACATATTAG

>TCONS_00046717

AAAACCCAGACCGAGGAAAAACACATAAAAACTGAAACTTTGATGGTAGGAAACTAATGACTCCGGTTCTCTGCGAAATCTTGCTCTCTGGGCTCACGGTTAAATCTGCTCTCCGCCGTAGAACCCATCTCGTTCAATCATTCTCCGTCGTCTTCCTTTACTGGTTTTACAACGTTTCATGAATTGCTGAAAACCCTCAAATCTAATCCT

>TCONS_00058622

AAAAAAGTTTCGAAAAACAGTGGTTGTATGGTGGTCGGTGGCCTTCGCTGCCGGTTACCATCTTCGTTTTTTATTTTGCTTTACGCCATTTTTATTAGTGGTCTCTCTCGGGTATCGTCGATTCTGTAGAGTTGCATGTCCGGTTTAAATTCAGTATCACACGTATCTATTGGTGGATGACTGACTTTTTACTCTGGGTTCAGACCGTCTTCTTCGGTATTGGTCGCGGGCTTTTGTATCCATTGTACATCAGGGGTGTTTGTTGTCGGTTGGTTTGTGGTTAAAATCTGAAGCGTTGTAGAAGGTTTCCTCGGCGGTCTAGATCGGTGAGATGATTCCGTCTTTGTGTTCGGTGGTTCAGTTTGTGGATGAGATCTCGATTTAGATCGGTTGAAGCTCTCCGAATAACGATTATAAGTGTATGTGTTGAAGACGATGGATTTCTTTTAACTTACAACATAGTCATTGGAGGTTTCTTTCGTCGTGGTGTTCCTGGTTGATCCAAGTGGTTCGATGAGAAGTTTAGTCCGGTGGAGACGTTCAGTCGGTGAAGACAGTGGGGCTCGTTTCAGCATGTGTCTCTCTCTCCACGTTTGGTTTCCAACACGTGTCGATGCTTCTCTTGTGGTCCCATGGGTTAGGTTTGGACCAGTGTTGAGTCTCTTTAGTTTGTGTTTTTTTGGGCTTGTAAGCTTATGCC

>TCONS_00051157

CTTGCATTGATGAGGAGGATAATTGATGCTAAGCTCCCACTGGAGGAGCAAGTGAAGAAAAGATAATTATAGTTTGGACAAGAGGACGAGGTGTGTGTTTTGGTGTAGTTGAAGATCTGTTTAGAAAACTCGGAAGATACTAACATCAAATGGGTCTCTAGTTCTCTCTTGTTAAAACCCACTAAACTGCTTTTTAGCGTCTTCAACATTTGAACAAAAATGCATCTGTGGCCATCATTGAAACTTAGAAACTCTTTCAAGAGCACTAGCAAGAAGAGATATCAAAGGACTAACAGCAGCGGGCAAAATAGTCAAAGCAATCAACAGAAACTCCTAGAATCTGGCCATGAGATTCCATCTGATAGCTGGTTTGGTGCTGTCTGCAGAGATCTTGCCATGGTTCTCTCTTGCTGCTTCTGTTGTTTCTGCTGCGGAG

>TCONS_00052608

GACCAAGGACGAGCGCAAAATCACAAGTGCTCGCAGACTTCTTGGTCGAACTTCCAACGGGAACAATAACCAATGAGGAACCAAATTCCACCTGGCTCCTCCACGTCGACGGATCCTCATCCAAGCAAGGATCGGGTATCGGGATCCGTCTCACATCTCCAACGAGCGAGATCTTGGAACAATCGTTCAGGCTGGAATTCCATGCCTCAAACAACGAGGCCGAATACGAAGCATTAATCGCAGGTCTACGTTTGGCTCACGGCTTAAAAATACGTAATCTCCATGCTTACTGCGACTCCCAGTTAGTGGCCAG

>TCONS_00030916

CTCTACCTCTTTCCATCTTTCACTGATTTGATCTTCTTGTTCTTCTTCTTATTTTCAGTTGAAGTTTTAATTAATCAAATCATTTCGTTTCAGGCTTCTTTGACTTCGATAATGAAAAGAACACAAAACTGATCCCCAAATCTCCAGAAAATTTCTGACCAACAAAACTGATAATTTTTTCAACAAATCCTTTCTTTTTGTGATATGGTGGATGTAGAGGTGCTTTGTTTCGTCTTGGTTTTGCTGGAGCAAGCAACAACTTCTTCTACCTCTGATCGGAGCTTCTAGATATGAGCTTCTCGTAGGCGCTCCGGCTTCTCTCCTCTCTGCAAATCGACGGGCTGTGGTGGCCAATACTCTCTCTTAAAAGTTGTTTGGTCTGGTGGCATGTGTTGTGAAGGGAAATAGTTCGCTTATGGTGAGGTCGATTTCAGATCTGGAGATCCTTGTGCATGGCTGGAGACTATGGTTTGCCTTTTTCTTCTGTGGTTTTCATGCTGTACGTATGGAGTGATCTCAACACTGCCTCATCCTCTAGCCTGTGTTTCGCTGTTGCTTGGGTGTGAGCTTTCCAACTCTGCTTCTCTAGTTAGTTGAAGTTTCGCTTAGGAACGGATGTTCTTCTTAGGTAAGCTCTCGGGGCTTCCTCCTCTTGCCGTCGATGTCCTCCCGGCTTTGTGTTCTCCATAGTCTTATTTAAGCTCTCAATCCGTGGTTGCCGGTTAGTGGTTGGGAGTTTGAGGTTTTGTTGGGGTGGTTCTTTGGTGCTCCGTGGGTTGAAGTTCTCTATTTTAATGATCCTTCCAGTTGTTGCTGACTTGCTGGTGTGCTCTTTTGACCTTGCTGCTTCACTAGATTAGTCTGTTTCTTGGCTTGAAGGTTAAGCAAGTGGTGGTTTTATCTTTGCTCGTTGATGCATGGATCATTGTCTTAGGTTCTTTGATTTATTGATTTCTTTTTGACTCTGTCTTTGTGTCTCTTAGAGTTGTTTGGTTATCTAGAATGAGCTTGCTTGTTATGCTCTGTTTTTCATGTCTTTCTAGTCTCCCTTTTGTGGGCTTTTAGACTCAGGGGATGAGTTTCTTCTATCCTCCTGCTGTATGTCTCTCGATCTTGTATTCCTCATTGATATGGTAACAAAAATTAATTTTCAGTGTGAAAAAAA

>TCONS_00073986

AGATAATCAAAAACATTTCTCAAGATTCTTTATCGATTTCCCGTTTATAACAGCCATTGTTACCAGAGGATCTTATTTGAGATTGACTCAGGCTCTTGGGCTTATGGAGGACAATGTGATGTCCTGAAAAACCTTGAGCCTCTTCTTGTCCAACACTTTCGTCTCTGTTCCCAAAACAGCCATGATCACCACTTCTTCGAACTCCTTGCTGTCTGGTAATCCAGATTCTATCACTTTCTTCTCTTTCGTGTTTGTCTCTCCCTCATCACCACCACCAAAACTCAACATTCCCATGGTTTGATTGGCGATCTTGTCCTCTTCAGCTTCTCTTTATTGTTGATCAACTTCGAAGAGAAGACGGTTTTGCATCAACCCTTAATGGGTTGTTTAGTACTACATTCGCATTCACATTTGTTTTCTCGTTCTCGTCACCGTACCCACCATTGCTCAAATCCGGCTTTGCCTTAGGCTTCACGCTCTGTTCTCTACAAGGCTGAGGCAGCCATATAGAGGAAGAAACAAACATGGTGTCGAAAAGCCTTCTCTGCTCAACCCTATCATCCCCTCCTCTTCTCATCATCTCGGAGCCAAGATCGTTATAAGCATCTGCTGCGCTCTCTTGTAAGATTTTAAATGCGAATCCACTCCTCTAGGACCGTCCATGACCGCTGGCTTCACAAGCCGTAACGTCTCTTTGGCTTCATTGATCCTGCCTTGCAGACAGATTCCGAGCACATCTTGTTGTTGTTAGGTGCGATAGATAGAGCTCTCCTGTAGGCGTGTTCAGCTTCCACGAAGTTGTCTCTTTGCATCAAAGCCCATCCTAAGTTCCCCAGTAGCCGAATTGCTTCTTGGTCAACAGAGACTTGAAACTTCTTCCCCTGAGATCCAGCGGTTTTGGTTTGTTTGCCTGTTTAAGGCGAGTCCTTTATATATCAAGAAGAGTTTGTGTTTCAACAGTGCAATCTCGTCAACTTTCTCCCACATCTCTGTAAACAAAAATAACACACAATGGTTCATTAAAAACTCAAGAATTGCTTAAGGAAACAAAGAAAAAACAGAGGATGTTGTGTTTTTTTTATAA

>TCONS_00037915

CAATATGAAAAGGTTGTCTTATTCAAAACCCCAATTATGAAATCCCTTCTATCCCACTTCACACCCCGGAACGCACCGTTCTTATAGAGAGAAAGGCACTTTCACATCTTTTTAACCCGAAATGGCTGGGGAGAGAAAAGGTTCCTTTTTTGTAGGGTACTCCTGGGAACAGATCCAGTGGAGACGGGGTGGGGCTTGTAGCTCAGAGGATTAGAGCACGTGGCTACGAACCACGGTGTCGGGGGTTCA

>TCONS_00078628

CTACCACTATACATCCTCCTTAACTTTTCCTTTTTCATGTTCAAATGAATCCATTCCGAAAAAGGTTTTCTCTTTCGCCTATATAATCCCAACCCATTATTTCACATAACGTCAAAAAACATTATTGGGTTTCATTACAACAATGGGTTATACCAAAGATCAAGATCGGTTCTGTTCCACGCCTAAGCTTCCTCTGTTTTCATATCCAATGAACATGCCATATGAAACTCCGGGACTAGCTACACCGCCCGTTAACATCGGCGGATCTGTTCCTTTTCTTTGGGAAGAAGCTCCGGGAAAGCCAAAGGCTCGTTCTTCAGTTAGAAAACCGCCGAGGTCGAAACATACCGGAGAAAAAAGAGGAGTCGTGAGAAGCTTGGACCTTCCTCCGAGACTGCTTTTGCCCGGGGAGTCTTGTAAATCGTTTGCGGCAAACGAGCCTTCTCCGACAACTGTTCTCGACGGTCCGTACGATTTGCGTCGTCGTTCGCTGTCGCTTCCGAGATCGGCGTATGTAGTCAGGAAGCTGAGAGGTGTTCCGGCACCGGCGCCGGCGAAGGAGAAGAGATTGTTTGGGTCGAGTAGATGGGGAAGTTTCGGGAAATGTAAAGATGTCTCTGAGGGTATATTTGACTTTCCACGTTTTAGACACGGCGGCTGTGATTGCCGGAGGGATTGGACCGGAGCAGGAAGTGACTTCTCCGGCGACGGCGGCACCAAAGTGAAACTTTTTAGACTAAAAAGGAAAGGAAACCTTTTTAACCTCTCTCATGCAACAAAGTCAGAGTTCTGGGGAAAGGGTTTACGAAGGGTTTAAGCAAGCGATTCCATGGAGGCGTAAGCAAGAGAATCTGTAAAGGATTAATTATTCTACTATTTAAATCGAATTTCTCTTAAATTTCTTCTAATTTTATAGCTGATCTACTAATTAATTAGTTATCTCTTGTTTATA

>TCONS_00064467

TACCGTTAAAGAAGATCCGTGGTTGTTTGAGCAGGAAGAGTCATTTGCGGTTTTAAGTTTTACTTTAATCTTGTAATCCGTGTGTGCGGAAACTCGCATGGGCTGATTAGGAAGTATGGTCTTAACTGCTGTAGACAGTGCTTCCGCAGCAACGCGAAAGAAATTGGATTCATCAAGTAGGGTTTTCCAAACTTTCTGAGTGTTATCACGCCGTCCGTCTTCGTCTTCGTCTTCTAGATTCGCATTAGAAGATCATGGGTTTCGCTGCGATTTGGAACTCTCATCCCAAGAAGTACGGGCCTGGTTCCCGCACCTG

>TCONS_00063690

GCCACCCCCACTTCGCCCCGCCGTATCCAATCCTCTCCCTGTCAAGATACGGTCACTTCACCTCCTCTTTGCTTCTCCTTCTCTGGTTTTCACCGGATTGCGGCCGGATCCGGTGACCAAATCGATTTTTGGGTGTGTCCCCAATGTCTCTGGAGAGAGGGCGAGGTCGTGACTGTCCGGAAGCTTACAGGAGGCACCAATTGTTTTGTAGATCTAGGGTTTGTTTTGTCGCGGTTCTGGGTCAGATCTGCGTCGGAGGTTTGCCAGACCGAGAGCGTCGCTGCCTCTCTTCCACCGGGTTATCGCCTCCTCTGCTCTCCCCTGTCTCTCTCTCTTCGGTTGCTGTTGCAGGTGCTTCCCCATCTTCGTACTCTGGTCCGAGATGTAAAGGCGAGTCTGAGGAACTCGTTGGGGACCTCCTCCCGTTCTGATGTCTGAGCATTTACCATGGTGTTTGGTTGGTGATCCTTTCGATGGGTTCAAGATGGTCTAGGGTTCGTGCCGTTCTCTCTTTCGTCAGATCTCCATAGTCGGGTCTAGCCTCTCTTCCACCTGCTGATTGGAGCTTTCGTCTCCTCCGCCCTTCAAGTTTCAGAAGGAAGCCGTCAGCCGTTACCTGCCTTTCCGATGCTCTGCAGCCAATCCTTCTGTGTGGTACTTGCGTGATGGGACCGGAGCTGAGCCGTATAACCTCCGCTTCCATCAGCCTGTCCCTCACCTGCTCTTCATCTCCTGGCTTATTTGTGTTGGGTGGGACTCACAGGGACACGCCTTGCCGTCTTCTGGAGTGGACAGCGTCTGGAGTAGATTTCACTGGGTC

>TCONS_00024894

ATGAGGAAGAGAAGCGCGAGAAAGAGGAGAGCCGAGGCAGGGCCACCACTGGTGCCCGATCCGAGAGAGGAAATTGGAGGCGAGGAGGTAGATGTGGGGATGCTTGGTCTTGGATTCGTCGGCGCCGGATTCGCCGGGATAAGAGAGATCGGCGTCGGAGTGAGGACGGCGGAAGGAGAGAGCGCGATCGAGCATCGTCATCGCGTTGGCCCGTAAACCCATTAACGAGACCTAATCAGAGAGCGCACCACCAAACCGTCACGAGGATCTCTATCTCCTTCTCTGTTGCGCAGGAGACGATCTCCATCTCTATTCGTCCTTTCCGAACCTGAGAAACGTGAACGGAGATGGTACCTTTGTCATTATCTCAACATAATCTCCGGCCGCCGGAATCTGTATCTACGGTGTTTCGGGGTTACGCAACCGCCACATCGAAACACGCCTGCGACCATCTTCGCCGTCACCTCCGACTTGGCTTCCATATCCGCTCTTCCCTTCAGATACTGCTGCAAGTCTATGATGCTGCACGAACGGAGTTCTCAAGAAGAGTCGTTGTATTCAACAGGGCTAAGTTTCTTCACTCCAGAGATGATATCAGATCTTTGTTATCTGTATGTTTCTTTAGTGGATGAGAGAGTAGGATAGGTACTAGAGGTGAGGAGGGGTCATCATCAAACCAGGAGACATCCAAGAGAAACACAAGCAGTGGACGGAGGTGGACCAATGTCCTCCTTGCCGTTAACGTTATGTAATGTGTTTAGTTCTTCTCTCTGTTACATGTAACAATAGATCATGTCTTTTGGTTTGAGATGTTGCATTAGATAAATGCTTTAGGCTTATGGGTTTCATTCTTGTGTCATGACAGAATGTATATTGCACAAGTTGCATCCAATGGTAGAGTGCTGACATGGGGAGCCAAGGTATCATATTCAGAGACTTGA

>TCONS_00039447

GTAGAAGAGAGAGAAGAGAGAGAAAGGAGATCTCGGGTCCGGCGAACGGCCGGCGCGTGAGCGCGCGTGCCGACGCCGGAGCCCCCCTGTTTTTCATGCAAGATATCGTATTTGGATCTCTTCACCTTTTCTTCCGGCCTCCGCCTTGTCCGAGCTCTAGATAGCCTCCATCTGTGGAGTCTCTTTTTCGTGGAGGGAAGACGCGGTCGCCGGAGTTGAGAGTATGGTGAAGTAGATCGCAGTGGTTTAGTCTCGATTCCGGGAGGTGGAGGCTACGACAGCTCCGCCGACGCCGGCCTTAGTCGCCGAGAGGTGGAAGGTTCTTCAACTTCACCGTCTTTGGCTCAAGCTTCCGGAAGGTGGAGGCTCCTTCAGTCTCACGTCGCCGGCTCTAGGTTTTTTTTGGTGTTCTATGTTACTTAGATGGTGGTCACGTTCTTGGCTCGGGAGAGTTTCTCGGCATAGATATTAAAGAGGTTGTTGCTTCGCCGCTGGAGGAGATCTCATTAGAGAGATGAAACTCTCCGACATCGGTTCAAGAAGTTGAAGAAGGAAGTTGGTTACGGCGGAGGCTCGAAG

>TCONS_00047655

AAAACACTTGCGTTACAAAAAAAAAACAAAACACACAAACAGAGACTTATTCGAAAACATATTACACACACATGTACGGTACGACATGGTGTTGTACGGAATATTAGATAGAAAGGAACAACACCAGACGATGGATATGGTAGATTTGAGTTATGAGAATCGATCGATAGATCAGCAATGTCTGGTGCAGTAGCAACGACGACGGAAACCACGGCATTTACCTCCGCCAAAACCTTCGTTGTGACACACGTTTCCGCAGTTTGTTGAGCTCACACATGTACCCTTGAACCTAT

>TCONS_00017262

ATGGTCATTGATAGCGGGAAGGTTACCTGGACGAACCGATACTGAAATAAGGATCCATTGGGAAACTTACTTAAAGAGGAAACTCATGAAAATGGGAATAGACCCAACCAACCATCGTCTCTACCATCACGCAAACTACATTTCAAGACAATACCTCAATTCCTCTCATAAGAAACTTGAAACAGATATCATTAGTGATCAATCTTCTTCGGTATCCGAATCATGTGATATGAGAATGTTACCCGTTTCAATTAGTACCAATTGCTATGATGATAGTGCTAGTAGTAGTGCCGGACATAGTCATTTGCCTGACCTCAACATCAGTCTCATCCCCGCGGAGACGGTGGTTTCTTGGCCAGTTTGTGGCCTTCAAGAATCTAACAGCAATGGTTCAACGCGTCAATAAACGCTTCTTCTTTTCCAGTGAAATCGGATATTTTATTGCTATATAAAAATGAAAAATGGTGTATTTATCTGTACTATTTTGCTAGATATGGTTGCTACATCGTGCCGAGAGATGTAGAGTGGGAGGGAGAAACTACGAAAAACCTGAACTCAAACAAAGCAACTTCTCAAAAGACGAAGACGATCTCATACTTAGGCTTCATGCACTTCTTGGCAATAGCTTATTAGAAGAAGCATGAACATAGTCTGTGTCCGCGCTCTCTATCCGCCCTCCA

>TCONS_00036348

CTTTGTAAGAATGTAACAAACATCTCTCATATATATAGTCTTGAGGAATTTACAACACTGACATAGATGGATGTATGATATATTTTGAGCTAAAACGACACCCGAGACAAAGAGGATGTATAATTATTTAGTTACGAGAAGGGAAGATTGAATGAAGGATGTGGGCTGTGTTAGATTCATTCAATTCATGAATGGCCCCTCTAAAAGGCTCTTCTTCTTC

>TCONS_00056494

GCGACTCAACTAGGTTATTGCCATCTTAGGGTTTTAGAACTAGGAATCTCGCCGACAGCTCTCGTAGCCCAGGCTCTTACCTTTTTGTAACGCTCAAACGCGAATTCGGAATAAGATCTACTTTGCTCTCTTTTCGATTTCTTATACTTTATCGTTGTTATTCTTGTGTTCTGATTGCTTGGCGTGTGGTATTAGCAGATATCTAGGACCTCTGGGAAATTAGGGTTTTCCTAGTTTCCTTATTTAAACGGAAATCGACAGTGCG

>TCONS_00081923

GCTAGATGGAACCCCGGAGATGTCGACGTTGTTGGGATCTTGTTTTGGAGGTCTATTTCAGATACCTGTCCGTAGACTCTACAGTGGGAAGGTCGTACACAGCATGATGACGAGGCAATTGGTTACTACAAAAAAAACGAGATGTGGCCCGTTTTTGGGTGTGGAAGCGTGCCTGCGTGGTTAGTGAACACATGTACAATGCATTATGACAATATAGTGTACGTTATATATTGTCGTATGGGCACAACCTGTTTTTGTTGTCATATATACATCATACCAGTATATGCATGGTTGAATGTACATTTTATAGTTGATACGAGTATAGACATAGCTTGGATGAACAAAAAAGAGATATCTTGAGTATTCGTATTGAAAATTTTATGTGTACTAATATGTGGTGTACATGAGTGAATTGTTTGTACGATAAACGTATACGAAATATCTAATGTACTCCCTTTAATTATGTACAACGTATTTTAATATTTACATTATGTGTACACATCAACATGTACACTAAATGTACACAAAGCTATCTCATATTTTATACACATGTACGCGTGCACGATATGTACACTGTCATCTCAGCATTCAGGCTGGTCGCCATAACCCAATCATGTACACCACAACATGTACACCCTTATATCATGTACAACGTATAAAATGTTTACATGATATAAGGGTGTACGTGTTGTGGTGTACATGACTGGGTTATGGCGACCAACAGTGTACATGATAAGAATACGCATAGCCTGAATGCTAACAAAGTCTGTTAAAGTGATCTGTGTTTTTATTGTACATATGTATAAGGTGGGTAACTAGATGGTGTACAAAATGGTCATCATTTCCCAGACACAACGTACGTTTACACATGTACGCGGAAGGTGAGCGGCAATGTAATTTCAAATACACAACGGTTGTTGTTTATGTGTACACACATTTCCATGTACACGAGTTCATGTAAGGGGTAATACATGTTTGCAGAACACGTCTAGTGGTGAGTGTACATGTGGATTTTTAAACATAGATGTATATTGTACATATTGTGGTTGTGGGTGTGGAAGTGTGCCTGAGTGGAA

>TCONS_00051965

ACCTTTTAATGTTTTGCATGATACTGGATCGTTCTGAGAACCAAGTACTTCCAGCTATCCACCTGCAAATGAAGACAACAACCTTGCGAACAATAGAAACATAACACGGTAGGTATCGACAAACTGCTCTTACTGGAGTAAGAGAGGAAGAGTAGCCAGAAGTGATCTATACAAAAAAGCAAGACGGAAGGGAGAAGTACAAAAATATGAGAATCAGTTCTACAAAAGTCAAAGTGAAGGGACAAAAAAAACATGGTAATTCAAATCGTCAAGGATAATCGATGATTGACTTTTTAGAAGTTCTGGCGAAGATGAATGACTTGATTGCAGAAGCTCTGTTTAAATGCTAGAACACCCAAGTATCGTGATCTGAGGAATCACAGAATCTACACTGCAAGATTCATCTTCCCCTCGGTGTCTCTTTTATTTTTTTGTGTCGGTGAGGCAGAGATGTATTTCTTCATCTCAATTCATCAACCTTAAACAATCTCCTAATCTCAATCGAAGACTCACCTAACGAAACGAAAAAGATTGTCCC

>TCONS_00042320

GAAAGTGGAGGAGGCACAGGAGATTGATGAATGAGGAACAGAAGATTGATGGAGGAGGTAGCAGTGGTGGGTCGTGAGCAGAGCCGGCTTGAATGTTGAAGACGAAGAAGCTCGGTCCTCATCTAATCTCATCTCTCTTATCCTCTTCCCATCTCTCTTTCTCCTCTGCTCATGAATCATTTACTTTTCTTCTTTCAAGCAAATGGATCTGAACGAAGAGGATTACCACCGTCATGCTTCTCACCACCATGCTCCTTTCTATTCGGTTGCGCTCTTCACCACCACCACCACCGCACGCTCGTCAACACCACCACCG

>TCONS_00011750

CACCATCACCAACAACCCAAGCATAACCCTTTACGATCACCTCTAAGATTCCGAGCCCCACACTCCGCATAGTCGAAGACCATGAGCCAGTAGAAGCAAGCCAAGATAAATCTTGTAAATCACCGACGTTATACTTACAACGCAGAACTTGGGCCCAAAGGCTAGTTTTATCATTCAACAGTCTCCAACTTACCTTTGCTAGTAACGCCATATTCATCTCCTTAGCATACCTGATCCCCAAACCACCATCCTGTTTTGGTTTACATATTCGATCCCAGGCGACCAAATGCTGCTTGCGTTGTTCACTCGTGCTTCCCCATATAAAATCCTTTGATAACTTATCCAAAGCATTAAGTGTAGATACAGGTAGCTGAATATTGCTCATACTGTGAACTGGGGTAGAGGACAACACAGCTTTAGTTAAAGTAATCCTACCAGCCCGGCTCAACATCTTCCCTTTCCACCCTGCTAACCGCGAGGAGACTCTCCCAATAACTTCACTAAACGTATCTTTATTAATCCGTTTATGAAGGATCGGCATTCCCAAGTACTTCCCAAGATCATGTGTGGATTGAATACCACTCTCAGATGTGATACGATTACTCAAATCCAGAGCCACATTCTTGGAGAAAAAGATCTTGGACTTCTCTAAGCTCACCTTCTGGCCTGAGGCATTGCAGAACCTCTCAAGGACTCTGCGAATCACTCTGATCTGCGCTACAGATGCCTCCGCAAACAATATCAAGTCATCAGCAAAGCAAATATGAGAGAGTTTAGGACCTCCACGCGAGAGCTGAATCGGTTTCCATATCTTCGCTCCCACCGCATCTTCAATAAGGTGGCATAGTCGCTCCATACAC

>TCONS_00079062

GTTAATATCTCAGTCTGATTGAGAGAGACCAAGATGTCCGATCTTCTTCGCCAAAGGTGACGTGGAAAATTAGGGTTCATCCTTTGCTTCTATCTCTGACGAATCTAGACCAACAATGGAGTTTTAGGATGTTGAGTGAGACGTATTATCGTGAGGACTCTCTGTGCCAAGCTTTGGTTGTGTCGTTTAGTCTTCTGTTTTTATCCTCTGAAACGTTACGAGAGGTGAGGTTGTATCTGTGAAATTCATCATTCTCTTTAGAATTTGAATCCGCCGAGCCTTTTGAATTCTCCGATGATGGTGGTGAAGGAGTGAGAGCAGGTTGCATTCGCATCAGGTGTCCGACGGACACGAAGAGCCTATCTCGGTTGTTTACCTCAGAGTGAAGAAGAACACCATGGCAAAAAGCCATGTGAGAGACTGTGATTTGATGATGATCAGAGATTTCGCAGAAGATGATCATCAGGGAGTTGGCCAATCTACAGACTTATTGCCAAGGACCATGATTCCGGAGACTTCAGAGAAGAGTGAGGATTTAGAAGGAACTAGGTTCAGACACGTTTTCATGACCATTGGAGTAACAAGAAGGAGTATGTCCTTATAATTGTTTTCAGACACGTTTTTATTATAAAAGAGCTGCAAACTTCATTAGAAGACGCTAATAAAAGTTTTCATGTCATAGAAGCTGAAGTTTCTTGGTGGAAAGACACTGTATGTGTATATGTAGGCTATGAGCATATACGTTCTAAACTCAACTCTAAGGAATCCAGTGTTTCAAGTTTTATTAGAAAATAAAAGTGTGTAAACC

>TCONS_00018580

GTTTCTGCAAATCACGAGCCACTGGTGCCTTCCCCGGGTCTCCGAACTTTGCCTTGTACACTGCTGCAATAACCCGAGATGTTGCTCTCGACTTGTAGCCCTGTCTGAATTCAATGGGGCAGATATGATCAAGCTGCGCCTTCCTTATCTCATAGAAACCACAACCCCTTATTTCATGTGCTGTCACACGCCAGTCACACCTTTTATCTGGACACTCCACCACAAAAGAACCAACTTTCGTCCTTGTCTGAGTAAACTTAAACTGCTTCTTAATTGCATATATCGCGAGAGCGATCTGACAATCTTGCTTACTACCAAACACTTTCCCCACAGCTATTTGGCCGTCATTTACATCAACATCCATGTCGGGGATTTCTGCAGCGTCGTATTCGGTATCATCAAACATTGGGGGGATGTCAAACTCCTCATCATCAATATCTGAAAGCTTACGTGTTGGTTTAGCGCTCATGTTTCTACCTACACCATCCTCACTCCCACCTCCATTCCAGGGATTACAATCCTCTGGCTTCATCATGTGTGCTTCCCTTTCACCTCCGCCTTCGCCACCAACCTCAACATAGTGGTCAAAAGCACTTCCGGTATCACAAAAGTATGATCTTGGCCCTGTCTTTTTCTTCAGCCCTTCAACAATCTCATCCTCGTTGAACACAACATTAACAGCATCGGAACCACCATAGTCGCCACCTAAAAGCGGTTCCCAAAACTCCCTGTCATACCCCCTTGGCCTCACTTCTATCTCCTCCACATCTTCTTCCAAAGTTCCTTCTGTCTCGGTATCCGCAGTGTAACTACTCTCACCCACATCTTTGCTGCTTTCTCCTTCATGCCTTTCACCCATTAATTTTTCTTCTACTTTCTCGACCTCCCGTACAAAATCAACATCATCAGCAAGGTTTATGACCTTCGTCTTTGAAGCAGCAGATGGCCAGAACCCCTTCTTCGAGGAACCGTTACGGAACTTCGCAGTATCTGGTGACAAAAACTTTGCCGCTTCGGGTGACACAAACCCCATACCAGAATCATCCACAACATCAGTGTCCACAACCTTCTTTAACGCTTCAAACCGAGCAAACAGATTCATCGCACCTTTCACTTTCAAGTGTTGGCAGAAATACTTCACACCTCCATCACTAGTAACCAGCACCGGTGGG

>TCONS_00078260

TAAATAAATATTGAGGAAGAAAGCTGTAGAGAGAAGAAGAAGAAGTCAATAGAGAGAGAGAAAGAGACGACAAATGGCGGAGGAGAATGAAGAGAGGGTGAAGCTGTTCGTAGGACAAGTTCCGAAGCACATGACGGAGGATCAGCTCCTCGCATTGTTCCAGGACTTCTCCATCGTCCACGAAGTCAACATCATTAAGGACAAGATAACGCGAGCTTCCCGAGGTTTTTCTCTTTTTAAAAAAAAATAATTTCTTCCTCTGTTTTCTCCGTCTTCATCGTTTAATTTTTCTTTGATTTCTCTTGAAACCTTAGGTTGTTGTTTTCTGATATGTTCTTCAAGAGAAGAAGCAGACAAGGTGGTCAATGGTTGTCATAACAAGAAGACTTTACCTGGGGCGAGTTTCATTTCACCATTCTTAATTTCCACTTTTTTTGCTTGATTTTCTCTTATTTTTTCTGATGATCAGTGTGATATTTCTGGTTACCTTAAAGTGGTTGATCTGTTAGTTGCACATTTATCAAGCCGGTTGGAATCTCGAGATTGATTTTCCTTCCTTTTCTATCTCTAGATCTGCATTGTACTTTTTCACCCTACTAATTCCGGATTTGTTTAGTTCAGAGTTTTGCATGTTGTTGTACTACATCAGTCTTGAATATGATTGATTTGGTTGACCAAAAGCTTCCTGACCGGAGCTCACTGGTTGTTGGAATCTGATTACTTGAGGTTTAAAAATGATTATATATTGTTTCTTCAAACCCACGTAAACTATACAGCAAGACTAACTTTAGCTTTAGTTTTTTTCTTACTTGTTGATTGATTGCTAATAATATGTTGAGCGCATCTTAGGTGAGAACTTGATTCATCTAATTAATGTTCACTTCCTATTGTTATTTTCAGGCGTCTAGTCCGTTGCAAGTTAAGTATGCAGATGGCGAGGTTGAAAGACTAGGTACGCAACATGCTTCTGGTTGATGGTTGCACAATGAACACATTTGCATAAATGATTTTTCTCTGAAAAGTTTCCGCATTTTAAGAAAATTTTCTTGCAATCCAGAGCACAAACTTTTCGTCGGTATGCTTCCAAAGAATGTCACTGAAGCTGAAGTCTTATCCTTATTCTCCAAATACGGAACCATAAAGGATGTGCAGATTCTAAGAGGGTCTCTACAAACTAGCAGAGGTGTGTGTACCCATCATTTGATTGTACATCTTAGTTAAGCTCTACTTATATGTACCAACGTTTTGACTTCATTTTGACTTTTCTTAGGCTGTGTTTTTCTGAAGTATGAGTCAAGAGAACAAGCGCTCGCTGCTATGGAGGCTATTAACGGAAAACATATAATGGAGGGATCGAATGTTCCTTTGGTTGTCAAATGGGCAGACACAGAAAGGGAAAGACAAGCCCGAAGACTTCAGAAAGCTCAATCTCATGCCTCCAGGCTCGCTAACTCTGATCCACAAAACCCTTCTCTGTTTGGAGCCTTGCCCATGAGTTATGTTCCTCCTTATAACGGATATGGCTATCATGTAAGTCCTTGTTCTCTCATTTACTTTTAGTACACGTTCTTAAATGCATCATAAACTGTAGCAACCACACTGATCTAATAAAATATTATCTCCATTCTCTAAGCTTTTATTATGTGATACTAGCCTTAAAATATCTATGCACTATAACAGGGACCTGGAACTTATGGGTACATGCTACAACCTGCATTCCACAATGTGATTTCACCAAACCATGGCGCCAATAATGCATTGCATGGAGCCGCTTTGACCGAGTCTGTTCCACCGCGTTTAGCCGCCCGTAGAAACTTCCCTATGCCTCTTGGAAACTACAGCTATCATGGTCTTCAGTATCCAATGGCGTTTCCGAGAGGGATGATCAGTCCTCGCCTTCCCCTAACCACTGTTTCACATGGCATTTCAAATAATGGCACATCGTCACCTTCTTCCCTTCAAACTGAAGGTTAGTTTAGTCCTAACCAAGAACATATATGATTTTGAAACAAATTATTCCTTATGCGTTTGAACAATTTTATGGTGTTCAGGACCGGCAGGTGCAAATCTATTTATCTACAACATACCTCGGGAATTTGGAGATCAAGAACTTGCGGTTGCATTTCAACCGTTTGGTAAGGTTGTAAGCGCCAAGGTTTTTGTAGACAAGGCCACTGGTGTAAGCAAATGTTTTGGTAAGAAAAATATCACCACCACCCTTCTTTTTCATAGACTCGAACTAGCGGTTGACATTATATTTTGTTACCACAGGATTCATTAGTTATGACTCACAAGAAGCTGCCCAAAACGCTATTAACACAATGAACGGTCACCAATTAAGCGGCAAGAAGTTAAAAGTTCAGGTTAAGAGAGAAAACGGCCAACAACAACAGAGTAGCAAACCCGTAAACAGCTGA

>TCONS_00009592

CTCTCGAACCACCAAGAGCATCCTGGAGACCACGACGAGGTTGATATCGAGTTTCTAGGGACAACGCCAGGGAAACCTTATAGCCTTCAGACTAATGTATTCGTTAGGGGAAGTGGTGACCGAAATGTCATCGGGAGAGAGATGAAATTTAACTTGTGGTTTGATCCTACTCAAGATTTTCACCATTACGCAATTTTGTGGAACCCTAATCAAGTAGTGTAAGTTAAAAACTATTTTTTCTATACGTACAGAAGAACCGTTTATACCAATTATATCCATCTTTTTGAGAAACAAAATACATCCATATGTTACGTTTGATATACTAATTAAATGGTCAATTTTTCATATTATATAGATTTTATGTAGACGATGTACCCATACGTACGTATGATAGAAAGAATGAAGCTATCTTCCCTACAAGACCTATGTGGGTGTACGGATCGATATGGGATGCATCAGACTGGGCCACGGAAAACGGGAGGATCAAAGCCGACTATCGATACCAACCATTTGTTGCGCAGTATAGAAATTTCAAACTAGCGGGATGCACGGCAGACAGGTCTAACTCATGCAGACCACCATCAGCTGCACCTACGGGGAACCGAGGACTGAGCCAGCAACAAATGGGGGCAATGGCATGGGCACAGAGGAACTTCTTGGTCTATAACTATTGCCATGATCCTAAAAGAGACCATACGCAGACACCAGAGTGTTAAATGGCTTTTCTTGTTATTCTTCTAGCTCTCTTAGTGTTCTCCCATTGTGGTTACAGCCAACGTTCTCCTTCGCCAGGATACTACCCCAGTTCTCGAGTACCGACTTCACCATATGATCGTGATTTTCGAACTCAATGGGGATTTCAACACCAGCGCAGAGAACACGATATAACCACTCTTTGGCTCGACAAAGCTTCTGGTCTTGTTCTTTCTTTTTTTTTTCTTTTTTTTAACTCAATCTTGTTCTTTCTTTTTCACCTTTTTCGTGTGCATCCAAATTTGTCGATTTATTGGTCCATATTTTGTGTCCATGTTATATACGTCTAAAGTATTATATGGTATTCATTTGAAAAAGGAACAAAGAAAAATGATATGAATGTTTTTAGCAAAAAAAAAATATGAATGTTAATTTGATATGAATTGATTTAGGTATGTGACGATTTGTTTCTTTGTCGAACCTCCCGCACTTATTTTCCTGATGTTATTTAATTCACCGCTCGTCTTGAAAATGTGTCGGCAGCTTATAGTATTTATGATATTTTATTTCCAATTAATTATTTTAATCAGTATAATTTATGCTTTTGTCGTATATAATAAAAACACTGTAATTAATTTAAATGTGATTATGAACAGGGAGTGGATTCAAGTCTATCCGTCCGTACATATCGGGTTACTTTGGTGCTTCCATTAAACTTCAATCAGGCTACACAGCTGGAGTTGATACGTCTTTCTTT

>TCONS_00008560

TTTCGTACTATTTCTGAAGTTCAGGATATCGTATTTTTCTGCGGACCTTTACTTTGTGTCTTACATGTGATTAACTGTAAACAGAAACATAGAAGATAGTAAACCGCAGCCACCAATCAGGAATTTTTGTAAGGTAATGAACAAATAGCTTTTCAAAGAACTCTAGCTCTAACATGTTCTTGTGCTTCGCTGGCCCTGGCCGTAATTATGGTCATGATCTTGCAGAAAACTATGATAGAAACTATCGAGTAGCTTGCTTACTGAGACACAAAGTAACCGGCATCAAATATTTTCCATCACTATACAGAAACCAAATAAAAGGGTTTTTTACAATTCTGTTCTAAAATTAACAACCTGCAACTAAAAGATTACATCAGCACACAAAAGTTACAGCTTTTTAATACAGGACTAACACCTTAAGTGCATCACCAGCGACTACTTTTTCTTAATCATCCAACCCCACAACGAATTGGAACTTTGTTTTGTTAAGAGATTTGTAATAATTGACAGACGACCAAATAATTTGTATTTACAGATCAGACCAAGCTGATTTCATCAATCAAACTACTCATCGCGGAAATTTCACTACACAGACTCGTTTCTTTTTTTTCACAAAGTTATTGATTCAAACGTCATAGTTAAGCTGAACGTAATTGAGGTGAAAACGGTACCTGGGAGATAAGTCACCAGCAGCAACTTCTCTCTTCTGCGCTTCTGCTTCAACAGCATCTCAAGGCCAATTAGGAGGCCTCCTGTCACAATGTTTAGGGTTTCTTCATATTCACGTCCAGTAAGAACGCCGTTGAGAAAAGAGAGAAGTCGGAGCCGTCGATGATGAACCGTCTTGGAATTGGCTTTTGTGTTTGCTAGGAGTAGCAGCCGCCGGTTTTCCCCCGTTTTTTAATGGGTAAAGGGAAACTGAGATAAGGTTAAGAGTTTCATGGTGGAGAATGAGAGAGACGAAGGGTCGAAAGAAAGATGTAAATATTAGCTGATCTGAATATTAGAATGAGCTGGCAAACACTGGTTGGCTTGAATTTTTAATCTGACGTGGATAGTTTAGAATTGGAGGATATCCAACTTTTAGTATAGTATAGATTAGCAAATGATGATTATGGGCCTAGTGGAAGATTAGAGTCCAAAGCTAAACTCCTCGTGGTTAAAACAACAACATAGGTCACGTCAAAATTGCGATTGATATTGAAATTAATATTCTAGACAGAACTGCCGTAAGAAATGAGGTTATAGACTGAACCACAGTATAGATTTATCTTATTTATATACCAATGCATCTACACATACATGAG

>TCONS_00073984

TTCATGTGGATTCGCCACATCTCCTATGGGTGCTTCCATATTCTTGTGGATTCGCCACATCTCCTATGGATACCTAGCGTGAACTACTGCCACACATACTTTCCTTAGACTCTATATGCTTTCCCCACCCATGCTTAACCGTAACATTATTCTTTCATACTTTCACTTATCCTTTCACATCCTTATCATTTTCACTTATCCCTTCCCATTCTCATTCACTTTCCTTTTCATTTAGACTTGTACTTGGACTCATTCACTAGACGCCACCCGTTATCGGTGTCACACACAATACACATCGCACTCAAGTTCTACTTCAATAACATTAACAATCATTACTCATCTATCAGCTTAGCATTCATTTATCTCTAGCATCCTAGCTTTAATCACAAACCACTCATGGGACTACACATCCAAACATACAAGCATAAATCACCAATC

>TCONS_00056544

GGTCAATGGAAGAGAGTACAACATGAACTTCAAAGACATTGGGAGGGTGATGGGGTTCCAAGACCTAGAAGACCACTCCCTTCCCAAGTGTGAGAACCTTCCCACAGAGCTTTGGAAGTTGATCACTGGAAACAGACACTCCACCGGGGCTGACAAGAACTCACACATCCGACACCCGTCTGTACGCTACCTCCACAGATTGCTAGTCCATGCTTTCTACCCACGGA

>TCONS_00060617

CCCACGCGAAAGTATTAAGGTTCTTTTTAAGACAGGTTATGAGTTCTGTCCTTAAAGGTTCGTGGAGATTGGCTCCAATCTCGACGCAGCGTTCTGGGAAGGCTTCGTCGAGACAGATCGTTACCACGGGTTCACAAGTTGGCTCGCGTTTTTCATCTAGAGCTGCGAAGATACGAGATTGCCAGAAGAATTCCGCTGAATCCTGACTTCGCGTATCTTTGCTGGAGGTCTTTTTCTCCGCTTTTCTAGGAGTGATTTCGAGGATCGGTCTCTTTCGTTTTAGTTCCGCGGCGAAACAAACCTGCGAAACTCTTGGATTTCCCCAGATTACCTCGACTCCGTTGGGGGTCGGGAACTTGAGGCAAAGATGGTAGGTTGATGGGATTGCGCGCATGGTGTTCAGCCATGGCGTCCCCATGATAACGTTGTAAGATGCGGGGCGGTCAACGACTAGAAACTCTGTGACGTTCGTCACGGTTCCGGCTTTGACAGCGAGATTAATCGATCCATAGGCCATGGTCGTTTCCCCCGAAAGCCCTAGCAGTGGGCTTGGGCATTTTGCGATCTCGGATTGACTGATCCCCATCTTTTCAAGAGTGTCTTTGAAGATGATATCGGCCGAGCTTCCGGTATCGATTAGTACTCTAGCGACGTCGATATCTCGAATCGTCAACTCGATAACAAGGAGGTCGTTTCGAGGTTTGGCTCGATCGATTGCTTCTCCTCCCTTGAACGAGATGACGTTCGCGCACGTCGAATCTCGCATATCGTTCCTGATCGTTGAGTGGCTTTTGCGGGTTAGATTGATCCGTATGCCCCCCTCCTTCTAGCGCCAAACTGTGGGAACCGAAATTCACACTGTCGATTTCCGTTTAAATAAGGAAAATAGGAAAACCCTAATTTCCCAGAGGACCCGGATATCTGCTAATTACCACACGTCAAGCAATCAGAACACGAGAATAACAACGATAAAAATAAGAAATCGAAAAGAGAGCAAAATAGATCTTATTCCGAATCTGCGTATGAGCGTTACAACAAGGTATAAGCCTGGGCTCGAGAGCTGTCGGCGAGATTCCTAGTTCTAGCAACCCTAAGACGGCTAAACCTAATTGAGTCGCAGCTCGAAATAACAAAAACGGAAAATTGCCTAAATTGCTCTAAGTGCTAAGTTTGCTCTGAAAAAGTTCTCCCTCTTGCTCCTCGCCTAGGACTCCTTATATACTAGCTCCAAGGTCGGTTTACGCTTTTACTCTTCTGCCCTTAAGCCGTC

>TCONS_00050707

ATTTACCAGAAGTTGTTCATGTCACACTTGATAGTTTATATGAACTGACAACCCCACTCAGATTTGGGCGTAGGCCTTTCTGCTGCCAATCCGAAAAATCTTAATATGGTGAAGAAGTTATCATTGGCAATGTTAACACAGGTACGTCTGTTTATTTTTACATACCTGTCATATCGCTTATGGTTCCTCTACCTTATGCTGTACAGTTATGGACATGGCTTCTCAAGGTTTTGCAGCGAAACTTACCAAGTCCCAAGCCAAACAAATAACAATCTCTTTGTTATGTAGCTAAGTGCTGGTCTTCTCATCATCAGATGTTGTGGTCACTTCTTGGAAGGGTTTATTGTTTTTCCACGACGAATCGAAGAGTCATTTTCTTCTTTGATCTCTTGAGTGTCTATATTTCTCTAAAATGGCTAATTCATCAATCCTTTTACTTTGATTAATTTTGTCGAAATTAGACATTCTGTAAACAACGTCTCTTTTTGGAGACTCCGCCATACAAACTCACCGATAGAAAATTCTGGAAACGTAACGTTTCCAATCGTCACCTTCGATGGGCTCATTAACAAGATCAGTGATAACAATGATTTCGATTTTGGTTTCTGGAAAATGATTTCAATTTTGGTTTTGTTTGTTAACTTAGCTTACATTTGTTCATGTATAGATTATCATTTTTTATTCCAGAGTTTTTGTGTATCGATGAAGATTTAGATTTGTTTGTTGATCTCTTTGATTGTTCCTTTTGATTTCATCAGTTTCGTCACCGGAAATCAAGTAAGCAAATCATGAACAGGGAGAGATGGGTTTGGGTTTGATCTTGGACTCGGGGAAGGATGATGAACCAGATCCATATACTCGCGGATATG

>TCONS_00050234

CCGCTAATCCGTTTTTTGTAATTCTTCAGAATTGGTTACTGTTCTCAACCCTTAACGATAATCACAAGAAGGTGAGGTTCTCCTCGTTGCCTCGGATGCCGGGACTTCTCGTGTCGTTTCCGGAGTAGCTAGCTCCCAAAGAACTCCACCATACTTGCAACCATCTCTAAACATGAAGCTCCAAACGACAAATAGAGGAGAGTTTCGGGTACATACCAGAGCTTTGCTAATGCCACCAAAGCCGACTTGTACACAGCACCACACAGCACTTGAAACTGCCTCCCGACCGAACAGAAACTCGCTACTGTCGCCGTAGAACATTAAAAAGGCCTGACCTCCAAGCCACACACCAATCCAAGAGAGCTCTAACGACCAGCACACAAAACAGCCAGCTACACTACCAGTGACGGGTCAAGTGACCCTTTGCCGGTACTGGGATGGTAGTGGAACACAAGAGAGACACCGCCACCGAAGACCAATCTGTGACGCCTCTTGCCGCCTTTGAGAACCGAAGAAACGGAGAATGTGACTCGCAACATAATCCACCTGTGTCAATAGTGAACCAACAGATAAAGCACGAGAGCTCGACTCCAATAGAGAAGCGGCCGCTAAACTAATGAAGTGCTCGAACTGATGACGAGTTGATGAAGCCGGAAAGACAAGTGGAACGCTGAGGAGAGCTTGAAACAGCCATAGACGACTGTGTAGGACTCAACTCGGCACTCCAACACGAGCCATAGACAGCCATAGACGACTGTGGAGACCGAGTACAACTAACACCAGCGTACCCAACCTCGACTCGCCTCCAGTTGCCTTTGTTTCACCGCAGATGGAGACGAACCCGACGACGCCGTAAGGGTTCCCGGTTCCTCTCGAAGAAAACACACACCACACCGGACAAAAGCAGCAACGAAGCCACTACCTCACCGTAACGGCGAGCCAACTCACCACCACGAACATGGAGGCCTCGAGAGAAGATCTAAAAAGGGAAATGCGACCTCTCTCTCAGCCGTAAAAAGCCGACTCCGCCAACCATAACCCCTGCAACACACCGCGCCTCGACTCCAGAGCAAGCCGGATCTCCACCTTCGCCGAATTCCTCGCGCGCGGCTCATTGAAAACAGAGACTCGCCTGACCAGAGACCGGGAACAAAGCGATAGGAGAGGGGTTTTGATCGTGAAGCAAAGACGAAGACATATAAAAAAAGAGCTGAGCCCCCGACGGCCTGACGCGTGCGGACGCGCCGGTAACGTCGGAGCAGAA

>TCONS_00060673

AAGCTTGGCACTCACTCTCTGCATATCTGACTAGCTCCAATGGATCTCTGTCTATGCCTCTGAACAACTTATCATTCCTAGCTTTCCAGATATACCAAATTATCCAAGGATAAGGATCTCTATCCTCTTCTGGTTCCAGAATATCATTCTTCCTCCAAAAAAGAAAGTCCATATTAGCGTAAATACTCGATACAGGAAAGATTTCAGAGCTCGATGGTGTTGATGATAACTCCCATGCTTGTAAGGCCGGTGGACACTCGAAGATAGCATGAGTAACGGTCTCTTCTGGCGCTCCACATCTTGGGCAATAATTATCACATCTCATGTTGCGGCGTACCAAATTTCTTGTTACGGCTATCTGTCCAGATATTAGTTGCCATATGAGATGGCGGATCTTTTGTGGCGCATTCACCTTCCAAGCAAAGGCTTGAAGTTTTGTGATGCTAGGCTGTAAAACTTGTAACTCCTCTTCGTCTCTCATCAAGTTTGTTGCAACCCAATATCCCGATTTGACAGTATATTGTCCAGTCTTTGTGTAACTCCAGCAAAATGTATCACGTCGATGAGTAGGACTAATGGTCAAACTCAGAATCATCGGGATGTCTTCTTGAGCTACATATTGTTCCAATAGTCGAGTATCCCACTCTTTGGTAACAGGATTAATAAGGCTGCTGTCAAGCATCTTAGGATTCACTGTTGGAGCTCGGGCACGCGCCGGCCTAGCTGGCATCATAGGGATCCAAGGATCTTCCCACACATTAATTTCGTACCCTGAGTGCACCTTACTTCTAATTCCCAGAAGTAATAACTTCCTCGCGGCAGTTATACTTGTCCATACATAAGATGGAGAATCCACAGTGCCTATTCGCAACGGCGAACTGAGACGGTAATATCTTCCTCGGAGAACTCTCGCTACTAGTGAATCCGGAAATTGCACAAGTCGCCACAGCTGCTTTGCTAGAAGAGTTAGGTTAAATTCATGGATCATACGGAAACCAATTCCTCCCTCTTCTCTAGGTGCACACATCTTTTCCCATTTTGCCCAGTGAAC

>TCONS_00035811

TTCGATCCTGTCATAAGCCTTGCTCATATCTGTTTTGACAGCCATCGAGCACCGGACTTTTGCTTTCGATGTGCGTAGATAGTGAAGGATCTCGTGTGTGATGAGGACATTGTCTCCAATCGCCCTTTTTGGGACAAAAGCCGACTGGTGAGATGAGATTATCCGCGGCAGGAGAGGCTGTAAGCGCTTCGTGAGTATCTTCGCGATAATTTTATAGTGGGTGGTGCAGAGCGCTATTGGTCGGAAGTCTGCAACCTTCCGCGGGGCTGAGGTTTTTGCAATCAAACGGACATGAGTTTCATTTTGTCGAGGATGCAAATAGCCTGTTTCGAAGAAGTCTCGGATGTCCCGGTAGATGTCTTCTCCGACGATGTTCCAGAACGAGTGATAGAACCCCGCAGAAAAACCGTCCGGTCCCGGTGCCTTGTCAGCATGGATAGCAAAGACTGCTTTGCGAACTTCCTCTCTGTCCGGGATTGCTGTAAGGCAATCATTGTCTTCCTGTGAGATGCAAGGGTTCAGCGCTTCATTCACTGTATCCATATATTCAGAGCCGGTTGCTGTGAAGATAGACCGAAAGTACTGAGAGATGGTTGCGACAATTCCCTCTTCAGTATCATAGATCTTTCCTTCCTCATCTTCAAGAATGGAGAAAGTGTTAACTTGGCGTCTGCCTCGGGTTATAGCATGAAAAAACCCAGAGTTTCGGTCCCCGCATTGAAGCCATTGTATGCGACTTCTCTGCTTCCAAAATTCCTCTTCATCAAGGTAAGCTTTCTCAAGCTTGGAAGAGATGGCATGGATTAGTTCTGTATCAGGTGTAGTGGCCGATAGAGCTTCTTCGAGAATCTGCTGTGCCTCCCTTATCACCTCACAGCTCTTAACATTCTGTTCTTTTGACCATTCAACGATCTTCCTCCTTACTCTGTCTGTCTTTGAGATGACAGCTTCCGGTCTTGGGAGAGGCCAATGTTCTTCCACAAGCTTGAGAATGTCCGGGTTTTTCCGCAGTCTCCTATCGAAGCGGAAGGATCCTTTCTTGTGGGCTTTAGTCTGATCAAAGAGAGTAAGAAGGGGGCGATGGTCTGATCCCTCAAAGCGGAGGTAGATGCTACGGCCAGAAGGAAAACATTCCGACCACGCACAGTTCGCCATTGCTCGATCAAGACGGGATTGAACAAAGTGATCGTGTCTCATTCCTCTCCAGGAGAGATGATTACCAGCATGGGGAAGATCCCATAA

>TCONS_00074913

ATAATGGTAACGTCGACAACCCTGATGTTGTTCGCGGGGAGATTCGGACTTGCACCATCAGCGAATAGAAAAGCAACCGCTGGACTTAAGCTGGAGGCACGTGACTCGGGTCTACAAACGGGTGACCCAGCCGGGTTCACGCTCGCTGACACTTTGGCTTGTGGCACCGTTGGTCATATCATCGGAGTCGGAGTTGTTCTTGGCCTCAAAAACATCGGTGCTATTTAAGCAACTGTTCAGCCCATGAGACGCAAGGCAAATGGAGCTTTGGGTGCAAAGTGTGGTGACTTCATTGGTTCATCAACAAATCTGGCAGAATCCATTCTGTGATCGCCAAAGCCACACGATGATTCTTGGCTAAATCATTTTAAAGACAAATAAGAAGAAAAAAACTGGTATAATCATAAATCTGCAACACAGATCGCCCAAGATAATACAAGTTTTTTTTTCACGAAAATAAGTCAGAAGAAAATGGCGAGCACTTTGATGACTACACTGCCTCAGTTCAGTGGTCTTAGAGCCAGCAAGATCTCTGCAGCTCCTGTTCAAGGCCTC

>TCONS_00068983

TGCATATTGAGATGAAGACCAAGGAGGCTGAGACAGTAACTTTAGAGTTTTACTGTGGTAACTTTATTTTAACCACAGTAACTACTTGGGGAAGTTATCTGATGGGATGAGAAGGTAACTTAGTGAAGAGAAGAGAGGAAACTCGACCAAAGAGGATAAGGGAGGATAAGGAAGGATAAGGGATCACTTTGACAGCCTGTATGATGCTGGAAAGGAGAAGTTACCTTTATACTAGTGCAAGCATGTGAGAAAGCACAGCCAAGTGATTCAAAAGGAGTTCCAGCCTGTCCTTTGACTACACATGCTTAACCATTTCGAATTTCCTTGTTGCTCTCTCTATTAATACTTGTAAACCTCATCAGTTATTAATTAATGGAGTTTTGAGTCTCTCTCTCTTTATTCTCTC

>TCONS_00018612

CTGGAAGCTTGTTCCTATTTTCGCAGCTCCATTGTGGTGGAGAAGTATAACCATCATTAGAAGTCTTCCATCCCTCTCATTCAGTGGAGCATCGATCTTGTAGTGAATAAAAAGAACTGAGGTTAGAATTCACAGCAACCAAAAATATTAAAAGCATGAAAGATTGTTTTTGTTTCCTTACTTGTACAATACGCGTTTGAGTGACCGTGAAAGCTTTTCTATGCCGTCCACGTATAGATCATCCAATGATGTTGCCAGCTAAATCCTTTTCGATTGAGTGTCTTTAGAGCTGAACCTTGCTTCCGCAAGTCAATTTAACTGTAGCATCCATAGAACATACAATATTCAACTGGAGGAAAAGAAAACATCTATATTGAACGCTTTTAGTCCAGCTAAAGAAACCATGTGAATAGGTAAGAGACTGAGTCACCAAACAAACTTATCAAAATCCTTGTGTTTTCCTTTACAATCCATCAGATTTGACATGCATCAAAGTTCTTATAGATCAATGTTGTATTGTTGTTGCTTGATTTCTCACACTGCACGGCTTTCTTGGATTTTTTATGTATATGTGCAAGTGTGAAAATTAGTTTGTGGTCACTGTAGGATGCTATAAAAACCCAGATACTCTTGCAACCCTTGTCGTATGCAGTCTCCATGTTGGCTAATCATCTTCACGTTAGAGGTAACACAGTTTGAATTTAAGTACAATATGCAAAGTAGAAAGAAATTCTTACTGAATTCATGACATCATGTGCATACAGACTCCAATAAAAATTTACTCCGGTGGGC

>TCONS_00068397

CAGGCTTATAAACTTTTCTGAAAATACTTTTGTATTATTGAGATTCGGTGATATGTTTACAACAGATGATTGATCTTTATATAGAGATCGAATCAATACAAGTATAAGAGACATAACTCTTTATACTAAAGGACACAACATGAAGAGTTACAACTCTTTGTGTAATAACATAAATAACTAACTTATGTAAATGTATCAAGGAGAGATTAGATTATATAGTTTAACAAGGGTTACCTGAGAATTTCCCCAGAACTTGAGAGAAGAGAAGAACGATCCAAAGACCATCGGAATAATGAGGCAGAGAGATCGAATCTTGCAAGCAGTGTGGATTGAAGTCAAAGAGTAAAACAGACGACGCCACGAAGAATTAAGCAATGTTCTTTATGATTATGAACCACGGAGAGAGAAACATAAACTGTGGATAATATACAGAAGAAGAAGAAGAAGAGGGAATGGGTGAAGAAGACGCGTTTGTCTTTCAATGTCGCGAACCTGTTTTGTTGGAAACTTCAAAAGAAGTGTTTGTCG

>TCONS_00063965

ACGACAATTTAGAGAGACCTTCTCTACAACCACTGTAAAGTGGTTGATTCGCCGCGTTTAACAATTCGTAAAACTTTTTTGCATCTATATTAGGTTCTTCATCTTCATCATGAGCTACGAATGCATCAGCTACCATATCATGAACCCTATCATAATCTACCATCTCCTCCTGATGGTAACTATGTTCATTATGCAAATGATGATCAACCGGTTCTTTTTCCTGAAAATTGCTATTACTATTACTAGCTTCATTCTGATAATAATTAAAACCTTCTCCATGTTGAAACCGGATATAGTAATTTAGCGTGAAACCTCTATTTATTAAATGCTTCCAAACATTTTCACGGTTTGCCAGTTTCGAATTGTTGCATTTCCGACAAGGACAGAACATCTTACCACTTTCTTGGGTAAGCGGTGTTGAATCTGCTTGATGCATAAATGTCTCCAGCCCCGCAAGGTATTCTTTCGTCACTCTCCCGTTAGCATCTCTATGCATATACATCCAATTCCGCAACTCGTAAATAGTCCCAGAGCCAGCCATTTTTTTTTCTTTCACGTTTTTTGTTGTTGGTGTGTTTAAAATGATGTTCAAACATCCATATTTATAAGAAATTTTGAATCTGGTAGTTGTAATTTTCCTATGAATTTACGACGAAAATTAATTAGGTGGCAAAAAAAAACGTGTAACACCTACAAAGTTGGTTGGATTCAAAATTTCCTCGCTAAATACACGTAAATTATTTCTTTGTAAATAACACGCGAAGTTTACGTCATATTTACGAGGAAATAGTATTTTCCTCGTAAAAGACACGTCAAGTTACATCGACTTTACGACGAAACGCTTTTGTCGTTACTTTACGAAGAAATAACGATGACATTATTTTTCCATGTAAGTTCCTCGTAAAATCAACGTAAATTTACGAGGATAGTTTTTCCTCGTTAAGATTCCTCGTTAAGCTTGTGTTTTCTTGTAGTGGGTCTTCTCATTTGAGCAGCCGGTTTACTCGGTTCACCTTCTTCTTCTCCAATTGCAACAGCAAACTCAAAATCGAAAGTGGTTCCTTTTCGTCGGCTG

>TCONS_00054592

CTGCAACTCATAAAGTCATAAGTCAACCTTTCTGTGTGTATTCATATATTTGGCGGGAAAATAACTATCAACCCGTTCTTCCTGATCGATATATAAAGTGAAACAGAGGTAGTGGGTGTGAGAAAAGTTTTAACGGAGAACGCACTCAAGGTGTTCGACGAAATTCCGTATAAAGACTATATAAGATGCCGCCACCAGAACCGATTCGATTCTTGAGAAACAGCGTGATCGTAGCTCTTGGCGGGTTTGTGACCATAAACGTGGTGTCCGCGGCAGCCTTGAGCGCATTTCGACTAGCTACAGAAGAAAAACGGAAGAAGAGCGGGTTGGCTTGTGAAGCGTGTCGTGGGAAAGGGTTTTACATATGCAAAGTATGCAAAGGAGACGCAACTATAAAATGGTCGCCGTTGTATGATCCTGTTTGTATCAATCCATGTCTTTGCCCTACTTGCGATGGTCACAGGGTACAAAGATGTCTCAACTGCTTAGGAAAAGGATACTGGTGACTCTTTTGAATCACTTAGGAAGCATGGGACCATTTCAACACTCTCGGACCGGTTTAGAGGAAGACAAAATCTTTAGTC

>TCONS_00052349

CCTGATAGAACCAAAATAAGGATCACTCTTTCGGTAAGGTTGATATGAACTCAGATCCGAGAGGATTGAGAACTGATGGTATGAACGGTGACACTAAGGGTTGCGGAATAGCCCAAAGATACTACCAGACTTCGATCGTTGCCGGATGTGACGCACCGGTCCAACAAAAGAAGGATCGAAACTTGGAGATAACCTCACCAAGAAACTAAGAAATGTTCTAACAAAGAACAAAGAGAGTAAAAACTCAAAAGAATGAAAGCAAAACGAAATTATCTTATTCAGATCTGATGGTTACATTATATAGAAGTTCTAGTCAAAAGACTTAAAGAAAAATGTGGGAAATCTAAACATAGAAAATGATAAAATTGACCAGATCTGGTCAAGGCACGAAATCACACTTTGACTAGTGAAAAACCCTTCAAATTCCGTCCAGGTTCATTGTATCCTGAAGCATGTCCTGCGGACTGGGCCAGTACACAAGCAGGACAGTCCAAAGTAGCCAGTTTTGAAGTGTCACGGCCTATCTTGAGTTCCACTCGTCCGAATCCAAATCTGTCTCGACCGTTGCTCTTAAAATGCCAATCAGTACGGGAAATATGAGCTCCAGTTCGGTGAATTCGATCGTCTGGACATGGGTATTGATGAAGCCTCAAACTGGACATCCGGG

>TCONS_00045436

GGCCTTTTCTTCCAAGCAATCTTTCACCTCATGGTCTAAACGGAGACAGGTGGAGCAATGCTTCTCCAGCTTTTCATACACTAATGTAGCAATGATTTCTTCACCATTTGAGTACTCAATAACGGTAGATTTAATCAATGGGAGAAGACCATTGACATGCGCTCGCATCTTCATAGCTAGGGGAGTGATATCAGCTTTTTCGAACTTTCCTATATCATCTCCGATGCTTCTCAGTATCTCCTCTGACCAGAGATGAATAGGTACTCCCTGTACCTTTATCCAAAAAGGGATTTCTGATGGGAAGCTTTTTGACAGAGTAGGTTCCCATCTCTGGACTATAACCATCCATCTGGCATAGTGGTAAGGTCGCTTGTCTAGGACGTTTTGCAGGTCTTGTTCACGCTCAAACTGAAACTGAAACATTCCATTCCCCAGATCTGCGCCTACTGGCCTTACATCTGACTTCCAAAGCTCTGTGAAGAAAGATAGCAGAGGCCACATTCTTTGAACCGAGGGATTTGTGATTCTTCCAATCAAGGTAAGCGAGCATTTGTTTATAAGTTCAATGTTCTCTGGCAGTTCAGCTCGAACTCGG

>TCONS_00009260

GTTGACAAGTGGAACGAGCAGAGGGAGACCGGAACTTTCCCTGGTCCTCTCTGATTCAAGCAAGAAGTCTCTCCTTTTATCATTTTATGCTTATGGAGGATGAGGTAATCATTCCTATTTTTTTATATATTTAACGTACATACGATTAGCTCTGGTTTCCCCCGGGAGATTGTAAATGAGGATTTGTTATTTTTTTTTTTTGAAGTTTGAACCTTGATTAAAGATCTGATTGATCACCACTTAATGCTTTACATCAGATTATTCGTGCGGAATTCGATTGTGATTGAAAGTTAGCTACTTTGTAATCTGTTAGATCCTTTTTCTCAAACACCTAGTTTTGTTGCTTGTGTGGGGTGTTTAGTATTGTAAGAGGTAGGCTAGCAATTTAAAGGCCAAGAGAGATGAAGCTTTGGGTTATGAAAAAATGTCTGTTTGTTTCACTCTAGTAGATCAGATTAAGAATTGAAAGGGAATATAAAGGATGAAGCTTTTGAATTCGAAAAGGCAATTGTTTATTTTCTTAGTTGATTTCATCTTTGTTTGTGTTTCTTTTGAATCAGATTGAGCTGAAAACTGCCCCTGCTGATTTTCGGTTCCCTACAACGAACCAGACAAGGCACTGTTTCACCCGTTACATCGAGTTCCACAGGTACTCTTTGTTCAAAATGTTACATTTGAGTTTCAACATGTACTCTTGTTTGATTGAGTTAGTGAGTCTTCAGTTCAAACTTAACTTGATACAACAAAACCATATCTTCAGGTGCACAACTGCAAAGGGTGAGAACTCCAGTGACTGCGAGAGGTTCGCCAAGTACTACCGCGCTCTCTGCCCTGGAGAATGG

>TCONS_00044928

AGCAAATCGTCTCCCACCACCACAAAGAATCCATCGTGCCAAGAAGAGTGATTTCTATTGAGAGTGAGGAGTCGTCCGATCATTGAAGATAGTAATCTGAGCGAGCCTGAAGACGAGAGGAGAGTTCAGTAATTCAAGTTTGTTTTTAGAGATCCGTATTAGAAGAGCTCTCTGCCTCGCTTTGTGCTTGAGTTCAGCTGCTTAACTGATTCTGAGCATAGGCTTGTTTAGTTCCTGTAAAACGAAATACATGTGGGATGAGTTTTTTGCTTCTATCCTTTTGTTCGTTATGGAGGTCGTAGTGCGGACCTCCCGACCAGTCTTTTGGTTCGCAATCTACGCCATGACTGCAGGTATATGTGGATTAGTTTTTTGAATTTAGCACCTGTTTAGACTTGTACTTTTTAGTCGCTTAAGGAGAAGGATCAAGGATGCAATGTTGCTTGATATGTTTCATGTGTGTGTTAATTGAAGTTGTTGAGGTTTAGCAGTGAATCTAAAGAATTGAAGACATGAATTAGAGTTTTTATGAAGATGTTGTGTATGGTAGTGAAAGAGTTTTGAAGTAAAG

>TCONS_00066026

AGGGAGATACACGAAGCACTTCCAAAATGAAGAAGCTTAACGGAGCCGGAAGCAGAGATGAGTGAGAAAGCGATGCAAGCCTAATTATTTGAGTTCGAAGAGGATCTCCTAGAACTGTTCCACCAGAGAAACACGTTCAATAACCATACATAAGACTGCTCCAACACAAGAAGCCACAAGAGAATCGATCTTCAACCCGAGCAGCCAAAGAAGACGACGGTTGGCAATTAACTACTCAAAGACTGCACGCAATTGAGCTAAGGCTCCACAGCTCCTTACCAAGTAATCCGGAAGAACCCGTTGGTTTCAAACCAAGGAACTGCACAGCTTCCCCGCCCCGAAACTTCACCAACAGAAGAGAGCAATATAACGACCAGAGGACTAACAGCAAGGCCTTCCAAAGATTACACCAATCAAGAACCAGACGCCTTGTCGAAAACAGAGCACAACCAAATTCCAATGCAGAGAAACTCTACCTGAAAACATAGCAAGTGATATCTCCCACCAAAGCCATTAGACCAAACAAACTCCACAAGTCCATCAACAACTAAATCTTCTTCCAAGAAATCTAAAAGCCGAACCCCTGACTCTGAATCCGCTACACTCTCCCAAGTTGCCTCCACGAAGAAACTTCATTAAGGACTCCCATAAGAACGCCCTTAAACGACGACCTTCATGCAGAGCCGAACTTATATCGAAAGGCTTTAGACCCAGAGCTAGGTTAAGACAAACACAACTAACAACTAAACGAGATCGAGAACATCCGAACCAGCAAAACGAAGTCAAAAGGACAGAGAAAGAACGAGAGACCACCATTGCAATACATGAGTTCTGCTTCACCCGATCCGATGGAAGAAGTATCACCCCACATCAGAGCCGGTATAACCTGCACCGCGCACCAGAAAAAGCTACGGATCTAGTGAAGGAGCCCAAGCAAACCAGAGGATGAAATCAAATCGATTCCCAGATCCAAACTTCTCTGACTCTCGAAAGCCTCATCTCAGCCTAAACAGACAAAAGGAACTAGACTAAGGACCTTCTCACGGCACCGACGAAGCTCGCCGGCCACCGGAGAAGGTAGGATCAAGAATCGCTCTCTTTTTTTTTACGGCGGCG

>TCONS_00005404

ACAATCTCTCGTCTCTCTATCTCCGACATAAATTCGATTGCTTTATGACAATCACCGCAGATCCTCAGGTTCTTGAACACTTGTATCCGTGACCCCTCAGGTAGCTTTAAGCACCCATAAGCAACAGCTAACTTCTCGCTATGCCATAACAAAAGCTTCTCCTTCTGCTCTTCTTCTACGTCGTGTAAAGCAAACTCTAGCTCCGGTTTGTATCCAGCTAGTCTCATCGTCTTCTCCATCTCCTTCAGCTTCGTGTGTATTGAGTCTAGCTCCGGGTGAATCCTATCGCTTGATCTGAAATGGTGAACCTTGTTTCGAATCTCTATCCAGCTATAACCAGGTGCCTTCACAACGTTGCTCTGCTTCATTCTCTTGCGAACCCTTGCAACGTCTTCCCAACGGTTTCTCGAAGCGTATATATTCGCAAGCTGTACATACCCGGCTGCGTTACTCGGGTCAAGCTCGAGCAGTTTCTCAGCTGCGAGTTCACCCAAGTCCACGTTCTTGTGCACCCTACAAGCCCCCAAGAGCGTCCCAAAGACAGCAGCGTGCGGCTTAAACGGCATTGATCTTATCAGCTCCAACGCCTCCTCCAGCTTCCCAGCTCGGCCCAAAAGATCAACCATACAAGTGTAATGATCAGGCCTTGGTTCCACCCTGTAATCTCTCACCATGGAATCGAAATACTCTACACCAGTATCCACCATCCCAGCATGGTTACAAGCCAGCAAAACAGCAACAAACGTTATCCAATCCGGTCTGGTCTTGTTAACCCTCATCTCACGGAACAAAGACAAAGCCTTCTCAGCTTTCCCGTGCTGTGCGTACCCGGAGATCATCGCGTTCCACGCCACAACGTCTCTCATCTTCATCCCCTGAAAGAGCTTCCAAGCATCACCAAGCTCTCCACATTTGCAGTACATGCTTACCAGAGACGTCAACGCGGTAATGTCATTACACAGTGTAGATGATTTGCACACTATCTGATGAACTTGCCTCCCAAACCGTAAGGCCGATAACTCGCT

>TCONS_00069552

CCACTCTCATTCTCTTTCTCTCTCTGACACGTAACTAACTATCCTTCCTGGATTCCTATCTTCTCCGCACACACACATACAAAACACAAAGACACTTAATATACATTGAGAAAGAAGATGTCGAGCAGAAGATCATCACGTTCAAGACAGTCATCAGGGAGCTCAAGAATCTCTGACGATCAGATTTCTGATCTTGTAACAAGGCTCCAACACCTTATCCCTGAACTCCGCCGCCGCCGTTCTGACAAGGTGTCAGCATCTACGGTACTACAAGAGACGTGCAACTACATCAGGAACTTGCACAGAGAGGTTGATGATCTAAGTGACCGTTTGTCGGAACTCTTGGCTACGACGGACGACAACAGCGCGGAAGCAGCCATCATTAGGAGCTTGCTTAATTACTAATATTCTCATAAATTATCTGAGAACTATTATAAATCATCTATTGTCGCCAG

>TCONS_00077911

GCCATAGGTTCCAAAGAGCAAGCAGAACATGAAACAACAGCAATTACAAATAGCAAGATGATTCGGTTCCTCGGTGAGAAGTAGAGCTTTAACTCCGATGAATACTTAAGTCTCCAAATCCAGAAATCGTGACTTTACTTCGGCTGGGTGTCCAAACCAGCCTCTGCTTCGTCAATACTCACCGCAAAGGGACCACTGTCGGAGGGGACCCGCGGCCACTGACGCCGGATCTATAAAGAAATCGAAACCCAAAGCCCTAACCACCGCAATTACTCTGTTACGCCACCAAACTCGCAGGTCCGACGCATCGCGTACAACCCGCCATCGCCTCTAAGCCGAGAAGAAATCCGCACCGGAGGAGACAGATCCCAAGGAACCGCCATCCTCAAAGACTGCTCCGTCGTTGGCAAATTCTCTCGAAGATCCGAAAGTATAAACCCTAGGCCCCGATGTCTATGTTTCCGTTTCCAGCCGCAACTCCACCATCCTGAAACGGAGCACAGAAGCCCGAGAAACCGATGAGCCACCAGGACCACCGCAACAGAGCCTTTACCGAATCAAACCGAGAAGAGAAGAAGAGAGTACCTCCGGAGGCGGGATCTAGACTGCTCCGACCATTAAGCTTTGAAGATCTTGAAAGGGAGGGGTGGGAGAGCTACACACGGGTCTGGCGAGGTCGCCGTAATGTAAACCGGAGAGCCTCCACACACGCAGATGGTGATCTCTCTTTGGAGAGAGAAAACAAAAGGCGCGTGTAGGCCACGC

>TCONS_00046566

CTGTAATTTTGCTCTATTAAATATTGCATACCTTCCTCAGTAGCTTTCTTTATATATGTTCTCTCCAGTACAATCTCCATTGCTCTAATATCATATTTTCAAGAAAACAAGATAGGACTCTCTCTTCTCACTACAGCTTTGGTCGTATGGATGATCTAAAGGAGAACAAGAACTCGCCATGGCTATCAGTACCACAGTTTGGAGACTGGGATCAGAAAGGAGGAACCATTCCTGACTACTCAATGGATTTCACAAAGATCCTTCTCGAGCCAGTCTTGGCAACGAAGAAGACCTCATTAACCCTTTTCATAACCAACCTACTTCAGTTGATAATACTAAGCCTAAACTCACGACTGTTCACAGCGACAACAACAGAACCAACACTGAGTTCTCTCACCACCAGCCACTTTCTCCATCTGTAAGAGATTACTTTTCAAATTTTTTGTTGATATCTGAATGATTGATTGTGTTCTCGATCAGTTCTGTGCCATTAATATAGGCTATCGAATATAACTTTGTAGTAATTTGGTTACACAATATTTGTTCTATATAGTTGATCATGTGTTTATATTCATTAAGTTACATGCATACACTTGAATTTCCCATCTTAACATTCATTTTTTCTTGATGTGGGTGTATATGATCTAGATGATTGATAGTGTCTTCAAACCGTTAGGTAGGTGTTTTTAATTTAGTTAGGTTGTGGAATCTAGCTTTGTAACTTTATATGGTTAGATGTTGGGTCACACATTGTTTGTTCTACTTTACCTTTTTCAAAATTGCAGACTTTTTTTTTGAGATTTTGACTGAGTCGTGTCTATGTGAATTGGATGATTGTGTATTTTGGATTATAGCTGTGTGTGTTCATTTGATGTGGGCCTTACCTTCTTGGGTTGGTTGCTTATGGAATCTAACTTTGTAAGCATTATATGGCTTCTTCTAGCAATTGGGTGAATGTGGTCCCTCAGTGTTTGGTCTATGTTAAGTTGCTAGCTGCTATTTATATATACACATGATATTAACACCCCCACCAAACTTCATAAATTAGATTTTGAGTTTATATTAGATCTGGATCATGATTGGGTATGGTTGAGGGGGTTTATATGGTTATAGAATCACATCAAAGAGATAACTATATAGTTCTTGTTCAAAGTAAAGCATAATCCTTTACAAGAAAAATGTGTGGCTTATAACCTTTTGTGATCTACCACGTGAACTGCACAGACGAGGAGAGGAATCTTCAGCTGCTTCAACTGCTGCGTTAAAGCTTGAAGGTTTTTTTTTATAATAGAGACGATGAGGAGATGAATCTTCAGCTGTTTCGATTATTTATAATAATGAAGACGGAAATTAAAATGAGTAATGGTGACACCATTACACTTGTATTTTTCTGCGGAAGCAGTAAAGTATTAATCAAGTTTGTCTGCTGTTTACTGTTATCTTCCAATGTCTAACTATGTTTTTGGTATTAATTTGAAGTGTAGTTTAACTTTCTCTCCTTCTTTTTTTGTTGTAATTTTCTTTTTTTCCC

>TCONS_00026857

TGTGAAGATAATGAATGGACCAGCGCCACACTCACAACTCGATGCAGATTTAGCCGCTGGAATGGGAATGCCGGACAATGGTCCAAAGCTCATGGCTGCTTGAGTTTACTTCAGTTTTGTTTTTTTTTTGTTTCGTTTCTTTTCTCATTAGTCTACAGTTTATGGAAACCTAGATTGTTGTTGGTAGAGATGGTCCTTAGTAGTTAAACTATATAAATCCTAAATTAATCATTTGCGTTTTTTGTTCTGAGTACCCGTATCAAAAATGTCAATGGAACATCCTTCTTCTGAACCAGCTCGGTATTTCTCCAAGGCGTCTGTGCGAGATGATCATTTCAAGACGAAGAAACATAAGAAGCGGAATATAAAATGGGTAGATGTCCGACGAGGAAGGTGAAGAAGAGGCGTTTGTCTCACAAAACAGCTCGTCGCGACAAATTCGAAGTCAAAGGCGACGATTTAGTGTACACTGAGCTGCGTAAACCAGAGACGGAGACAAAGCCTTTGGAGCTTAACGAGGATTTGCCTGGGATGGGTCAATTCTACTGCTTGCATTGCGAGCAAGACTCTGCTAGGGCTTCTCTTCCGCATCGTCGTCTCCATCTCCTCCTCGTGTAGCTTCTTCTCTAAACC

>TCONS_00060636

TTTTTTTTTGCTTCGGATCTTAATGAGGAACAATTGCAAATCTGTGGTTTTTGGAAGCACCTCTTTTCTTTTCCGTGTCACGTGCTCTTGCGCGGTGGCTCTCTTCGAACTCCATGTACTCAGAAACTCTTCGACAGGCTTCAGTGGTGGTTCTATCTGCTGGAGGACAGCGCAGAGGCTGCTTAGGGTTTCATAAAAAAGTCAAAGAAGCTTAGAGAAGGTTATTGCGGCATCAGTCGGTTAGTTTGTTCTGGATGATGTGGGAAACTTCCAGGATGGCAATTGTATCTTGTGAGATGGTGGTTCCTCTTGGTTATCAACTTGTCGGAGGCAGAATTTGATCTTATACGTATCAATTTTGAGGGTTGTGTGCAGTGTTTGACCTCTATGTGTCGGCACCGCTTCAACAGCTTTTGTGGTGGCAGTGACAGGGTCGGTTACTCTCTTGAGGTAACCGGTGTTAGTGTCCGCGAGACTCCTCCTAGAGTTGTTTCATTATTGAAGCTTCATCGTCCACGGATCTCTAGGATGGTTATGGAAAAACCGGTCATCTGAAAAGTCTTCGAATGCTCGTGTCTATCAGAATTGGAAGCTATCCTTCAATAGTCTTCTTCCCACTCTTTTTTTTAATATGTATATCTGCTTATTTGATTGTTCTTTTGTCTAATTTATTTTTCACTTCGAACTTTGATCCGGAATCTCGATGTACCAACTGTTTGATCCTCGTTTGGGTCTTTAATAATAATTATTAT

>TCONS_00016010

GTCGACTTTGCTAGATCTGTTCTCTGCTCTTTAGTTTTCAAATTTAAAATTCTTGTGTTCTAAAAGGTTTTCTTTATGAGAAAAACCAGCTTGGATATTTAGTTTAGGGCAAATCTTGTTTGTATCTTTTCTTTAAGACAACCTAGGAGCTGTTATTATCTTTTGTCTGAAAGTAGTTTTCATCGGTTTTTGATTAAAAAAAAAGAGTAAAACGGAGAAGTGGAGGTGATGACAATTTCCCCAGATGATCGAAGTTGATTATACTGAGCGAATAGAATTTTCCTCTCATCAGAGGAAAGTCAAGCCAATTAGTCTGATCCTTCTCTCTTTTATTTATTTGAATTAGATTTTATTGTTGTGTTTTTTATTTTTAATTAAGTTTAAAGATATAGTAAATGGAGAGTTGGAGGTGATGACAATTTCCCCAGATGATCGAATCTGATTATACTGAGCGATTTTATATTTCCTCTTCATCAAGAGGAAGTCAAGCCTATTTTCTGATCCTTCTCTCTAATTATTTATTTTTTTCCAAAGCGTGGATTTTTTTTTCTTGAGAATGTATTTTATAAAATCTATTAGTGATTGCAATGGAGATATGGAGGTGATGACAATTTCCCCAAATGATCGAATCTGATTATACTAAGCGATTATTATTTCCTCTCATCAGAGGAAATCAAGCCAATATTCTGA

>TCONS_00052278

CCTAACGCCCTTCTTTATACATGCACCACCAACCCATATTCTCGGTCCATTAGGGGATGGCATAAGTTTCTTTTAGAGGCAGCAAGGGACAATGTTTTCAGTTATAGTCCTTGTCTTAGGACTTTCTCAAACTGTACCATTCAAATAACTGAAAACAGAAAAGTCTTTGGTCTTTAAGACTTGTAAGCCGTGGGTAATAAATCTTTGTCTTTGTCAAGACATGTGTAAGCCGTGGGTAATAAATATTTGTAAGCCGTGGGTAAAGAATCTGTTTTCAGTTATAGTCTTTTGTCT

>TCONS_00018700

TGGCATAAATGGTGATAATGTAGCCGGGTTGATATTGGAACGTATGGAGAAAGACGATGTGGTTCATCTATATGATCGCTTTTTTGTCAAAATGCCTTTTATTGTTGAAATATGCATTGGAAGGACATTTGATTAGCTCCAATGGTGTGTGATGAAAAGTCCGAAGTCTACAATGCTGAAAACGTGACGGTATTTTTACATCTTCTAAAGGTTATGGTTACTGAGAAAGTATTTTTCGATCTGAGGTAAATTTACTGATTTGTGTCTATTTTTTTGATTTTCGGGTTTGCTAAGCTTCTCTTAAAGGCTATGGTTACTATGTTTTCTACAGGCTAACACAGGGGCTTGGTTATCGAGTAGGTTGTGATATATATAACCTAGGATTCTATATCTACGTTGTGTCTCCTGACCATAAGCCTAAAGATCTCCTGATAAAGCTATATGACTTTGTTGCAACCATTCCTGTCTATTTGGTAATTTTTATTTGAAATTTAAGTATTTTTTTATTGTTGAACCTTACTAATTTCTATCTTGACGTTGTTTGCAGGCTGGTGTTGATGATCAAACTTTTAAAAGTTTCAGGAGTGTAGTTAATTTCCCAGATGAGTCTGGTTTGTCTTATGACTTGGTTGATGATGACAG

>TCONS_00063761

GGGGTCCAAGACTTTTCATAACTTGAGCTAGAGGTTTTTCTACTTATCCCAATAAGACAATTCACTTAAACACAAAGAGTCATTTCTTTTCCTTTTTCATTGCTCCCAAATCATAATCACAACACTCACCCCCCACCTATAGCTAGACAATAGAGTGCCCAATCTAGCAAGAATGAAGATCAAGCATTGTCGTTCCCGATACTCTCAACATTATGCACATGTAAGACTTTCCGAAAAAGGCCTCACTCATCAAACAATGAAAGCTTAAAAGGAGGGAAGGGTTTTGGGAGTGGTCTACCACTAGAGTTTGTCAAAATAAGATTGGCATAAAGGATGTGACAACTCAAGTGTGTATAGCCATAACTCAGTACATAAGGGACCATGAGCAAGAAGCATTAAGATCTTTCAGTTCAAATAAAGTTGTAGTTGGCTTCAAAGATTGAGTTTCCGCAACTAACGAGATTCAGGAAGAGTTTTCAAGGCTCGAAACATACAAGGCTTTTTGAG

>TCONS_00056380

GTGTTCCACACTTACCCCCATGGACATACAGTAATCATTAAAAGCTTGGGAACTGAATTCACTTGCATTGTCAAGACGTATAGTTTTAGTGGAAAATCTGGAAAATGGGCTCGCAGTCTTATGATCTGTGCCAATAGTCTTGCCAATGCTAAGTTTCTAGATGATAATAGACAAACATGCGACCATCTGGTCGATGCGTCAATGAGGACCATAAAATATCGAAATGTCCCACAAGGTGGGTGTATTGGTCCACATATATCACCATGTATTCTTTCCAGAAAGTTTAATGTTTCCTTACTCACCTTTCCAGGTGATGGCCTTATTATTAATTTCCCTTGTGAGCATGGAATACATGTAATGCTCATAGGGATAGTTATCCTATCTTTCAAGGAATGGTCAGTTAAGCTCAAGATTATTTTGCGCATCATGGATCTGCCAGGATAGCCAAGCCTTTCATGCCATTGTTTAAGGGCTTCTCTGAACTCATTAGGAATTGAAGTGTTAGCCTCACTTAATTTTATATTGGCACAATAGAGACCCATTGATATAGCAGGGATAGTCTCTAGTACTTTCTTATGGCCTTGGGCATTACATACTTTCTTAAGTGTGGAACACTCCGTGGCACATGTACATACACAGAACGGATTGGCCGAATCCTTCATTAAAAGAATCCAGTTGATTGCTCGACCATTACTCATGAGGTCGAAGCTACCAGTATCAGCTTGGGGACATGCGGTATTACATGCTGCTGAATTAATACGCATAAGGCCATCCAGTGAACATAGATATTCTCCATCCCAATTACTCTCGGGTCATGAACCAGACGTATCCCATATCAAAACATTTGGATGTTCTGTGTACGTCCCTATTGCTCCACCACAGAGAACAAAAATGGGACCTCAAAGGAGGATGGGAATATATGTTGGTTTTGATTCTCCAACCATTATTAAATATCTTGAGCCTAC

>TCONS_00007214

CTTCTGTCTATCGGATAGTATAGTGAGCCTCGGCATGTTCTCGGTGTTGGTTTCAAGAAGATTATGAAGCTCACACAAGAACCACATCCAGTTCTCGTCGTTTTCCTCGTCCACAATCCCAAACGCTAGCGGAAACAACGCGCCGTCTCCGTCGAACCCGGTCGCTAAAAGCAATGTACCCAGGTACTTGCTTTTGAGATAGGTTCTGTCTAGACCAATAAGCGGGCGGCACGCGTTTAAGAACCCGTAGATGGAAGCTTGGAAGGATATGAAGAGACGCTGGAAGCAGTCGTCGGACGGATAACCGTAGACCGAAGCGATGCTTCCAGGATTAGTCCTCTTGACCTGGTGGCAGTACTGAGGAAGCAAGCGATAGCCTTCTTCAAATGAGCCGCGCATAGTGGCCATGATCCTCTCTTTACCGCGCCACGCTTGCTTGTAGGATAACGTGATGCCGTGGACGCGGTGTATCTCCTCGAGAATCTCTTTAGGTTTGCAGTTGGGATTCTCGCGGAGACGCTGCTCCACGGAGGTGGCCACCCACTGGACCGAGGCCTGCTGGTGGCCCAAGTGGTTGATCCCTCCGCAGGTATGGCTTTCGTGGATGGTACGGATAGTGAACGTCGGGACGCCCGGGAGTTTCGCCGCGTGGACTCTCCACGGGCATCCTTCGGTAGAGCATTTGGCTGTAAATCGTGTTTTGTCTGATTTTATCGTCTGCATTTCGAAGTGCTGGGCGATGGCCATGTCTCTTAGCGCTCGGCGGCAGCTCTTGACGTCTGGGAACTCTTGTCCGACGGTTAGCTGGTAGGATGACTCGGCGGAGAGGGCGCGGGCTTGGACAGGGGTGGTGACCATTACCATCTCGCCGTGGTGGTCGATCTCGATCTCGTGGGTTGAGTCGATGGCTAGCTCGAGGTTCTCGTTGGTGCCGGATAAGGTGAACATTTGGTTCTCGTCGTCGTCTTCGTGTTCAGGCTTTTGATCAACGGAGAGTTCGTTCTCGTTCATGTAATCGTGATGATGATGTTCTTCTTCGTCTTGGTTTTCGTTGTGTTCTCCTAAGTCGAGCTCGTGGTCATCGTGTTGATGTGCTAAGTCAAGTTCGTGATGAGGTTGTTCTAAGCCCATGTCGTGGTTTTGACCGAGTTCTAGAGTGTGGTTGTGACCGAGGACCAGTGATTGATCCTGAGTTATACCGATGTTGTGG

>TCONS_00056466

TGTCGTTGGAGACATCGCTAGGATCCAGGTCAATGTGTTCGGCTTCGTTATCCTCCGAGTCCTTTGCAGGGAGCGGAAGACTTTCAGAGTTCCCCTTCTCGATGGGAGACTTCTCGCTGGGGTTTTGACCGGAAGGTCGTTCCCGCGCGACTCCTGCTCTATCGAGTGGGGTGGCGAAGTCGAGCCTCTTCCCGCGGATTTTGGTGGTTCCGCGGGGACGGATAGCTTGGGTCCTTGCCGTTAAGGTTTCAACCTGTTTGGTCAAGGTATTCACGAGCTTATCCTGTTCTTCCGACCTTTTTTCATAGGTGGCGAACATCTTTTTAAACTCCTCGAGCGTCGCAGCGTTGGCTTGCGCGTTGGCCGCGGATACGTCCGCTACTGGAGTGTGGAGATCGGTGCCGCTGCCTCCATTAAGAGGAGTTTGCACGTTATCCGCGTCGTTGGTTGACATGTCTGACTGTGTGTGGCTTTTGCGGGTTAGATTGATCCGTAAGTCCCCCTCCTTCTAGCGCCAAACTGTGGGAACCGAAATTCACACTGTCGATTTCCGTTTAAATAAGGAAACTAGGAAAACCCTAATTTCCCAGAGGACCCGGATATCTGTTAATTACCACACGTCAAGCAATCAGAACACGAGAATAACAACGATAAAAATAAGAAATCGAAAAGAGAGCAAAGTAGATCTTATTCCGAATCTGCGTATGAGCGTTACAACAAGGTATAAGCCTGGGCTCGAGAGCTGTCGGCGAGATTCCTAGTTCTAGCAACCCTAAGACGGCTAAACCTAATTGAGTCGCAGCTCGAAATAACAAAAACGGAAAATTGCCTAAATTGCTCTAAGTGCTAAGTTTGCTCTGAAAAAAGTTCTCCCC

>TCONS_00034122

TTTCCTTCGTGTTTCCTCAGCTGTCTAGACGCATACGCGATCACTTTGCCTTGTTGCATCAGAACACACCCAAGACCAACTTTGGATGCATCTGTGTAGACCACATATGGCTCGTTGTCCATTGGAAGAGCCAATACTGGAGTAGTGGTCAACATCTCCTTGAGTTTGCTGAAACTCACTTCACAGTTTTCTGTCCACACAAACTGAACATCTTTCCCAGTCAACTTTGTCAACGGCTGAGCCATGCTAGCAAATCCCTTAACAAAACGCCGGTAGTATCCAGCCAGTCCGAGAAAACTCCTGATCTCCGTTGGGTTCCTTGGTCTTGGCCAGTCCGCAATGGCTTTGATCTTTTCTGGGTCGACGGACACTCCCTTGTCCGACACCACATGACCCAAGAAACCAATTTCTTTTTGCCAGAAACTGCATTTGCTAAGCTTGGCGTACAGCTTATGTTCCCGCAGTTTATCCAAAACCATTCTCAAGTGTTGCTCGTGATCTTTCTGATTCTTTGAGTAGATCAAGATGTCGTCTATGAAGACAATAACGAATTCATCCAGGTAGTCTCTAAAAACGTTATTCATCATCTTCATAAACGCTGCAGGGGCGTTGGTCAGTCTTTATTCGGGTACATGGTTCCGCTCCATCCACTACTAATCAGACTTCCGAACCAAGAACTCCCATCCACCCACGGTAAGTCCTTACAACTTTGGCCAGCTCTTTCTAGCATCATCAAACCCATTCAGTACGTTCCAACAGTCCGGCCAGTCAGTCCTCAGATCATCCTATCATCGATCTTCGGAACACGGTCGCTACAAGTTCTCCCCCACTTGGCTCAGACTCGTCCTCGAGTCTTTCTTCCTGAATCTTCCTTGAGTTTGTCAAACCACTTTGGAAACTCAGCTTTCATTCTCGCCTCTGGTTCCCAAGTCGTTTCTTCTTCTCCATCGCAGTCCCAAACGACTTGGATCATTTTGATATTCTTTCTGCCTTCAGGTTTGGTTTTCCTACCAATGATCCGGACTGGTCGTCCCTCCACTGTAAGGTTTTCTCTCAAGTCAGATGGTGGTTCTGAAACCACATTGTCTTGATTCCTTATGCATTTTCGCAGCAAGGATACATGGAACACGTTATGGAACTGAGCCATCGATGAAGGCAGTTCCAGTTTGTAAGCGACCTTTCCAATCCTTTCCAGGATCTTATACGGCCCCATGTATCTTGGACTCAGCTTGGCATTCTTTGAGGATCGGTCCTTGCCTTTGTAGGTGACGGTTTTGAGATATACCATGTCCCCTACTTCAAACTCAAGCTCTTTTCTGCGTCTGTCTGCATAACTCTTCTGCCTGTCCTGAGACTCTTTCATCTTCAGTTTCAAGACTCGGATTCTTT

>TCONS_00069468

CAATCACAATCAGTTCAACCATCCAAATAAGAGTTCAACTCAGACACTGCCCCACTAAAAGATAAGATCTACACGTGTTTATCAAGAAAATAAATTAAAAATATGCTTGACACGTGTTTGTCGAAGGTGCAGGACGATTCTGGAAACAGAGGAAGCATCGACGGTGTCGCCGGAATCTTTGCCGGATCGAAAACCGTGAACTTTAATCTCTGATTAGTTGAACTGGTTGACCGCTCCGCCTTTCCAGATCGCCGCTGATCTTCTTCAGGTTCCTCATCGTTGCGCAAGTGTTAAGTCCAGTGAAAGCTTCTAGGAGATAAACACAAGAGTCTTGCCAGAATATTTCATGAGGAGAAACTCCGGCTTATCTGAATCTGCATCTTCTCTCCTTCGCAAAATCAAACATCTCAATCTTTGGATACTCCGCTATGTTTCATTTATGCGAAGATTGATTCCTTGCTGTATAATTGATCAAGCATAACTCACAAAAAACCCTCCAAAGACAAACGGAATAATCAATAGGACAGGAAAGGAAAAACGGATTTAAAACAAAAAAATTAACATAACAAAAGGTCCCCTAGGGAAATTCAGATCCGCTAACGGAAAACAAGAGTCGCCAAGCGAAGATAGATGTAATATGATACACAAACATATGTTACAGAGGCAAAAAAACAAAATGGGTGGTGACCGGCGGCGAGGTGCTCACCGGCCACCGGAGGACAGCTTTGTTTAATGTGTTTCTAGAGAGAAGTTGGAGAAAATAACAAGAGAGAGAAGTGTAGGAGGGTGGTTTTAAAATATTTTACTCATTATTCAAATTCTCATTAGGGAATTTGCGACTTGTATATTAGTATCCCTTATATATTAAAGAAGAAGCATTGTAATAAATGCATTCACACTATAATAGACACGTGGCAGCCTCACGATGATTTGATAATAAATATGTTAATGCGTTCATACTATATTTATAAATGTGTTCACACTATATATTTTATATTTTTAATATAAAAC

>TCONS_00081313

GGAGTACCCCAGGAACGGTCTCTAACAAGGAATGGTGGAAGGCGACAGATGAGAAGTTCCAAGCTTGGCCTAGAACCGCTGGCCCTCCCGTCGTCATGAATCCCATTAGCCGTCAGAATTTCATCGTCAAGTCTCGTCCAGAATAAGGGATAAAAGGATGTATCAATGCTTTTGGTTGTAAGCCTACAATGAATGAGAATAATGATGCTTCTAGAACTTGTACTAAATAATATTACAACACCGTTTGTGGAACTAACTCCACAGTCTAAAGAAAACTAGTTTACGTCTTCTTGTTCTATGGTGCTTCTGGCACCACAGATCTTCTTGAATGTTGATATGGTAGTGAGAATCGTTTTCAGGTTATAGAAGAAAATGGCAGCATCAACAGGAGGAGGTAAAGCCAAGTACATCATCGGTGCTCTCATCGGCTCTTTCGGAATCTCATACCTCTTTGACAAAGTTATCTCTGACAATAAGATCTTTGGAG

>TCONS_00038264

AAACGAAACTCCTACATAATCTGTGAATCTTCTTTCCATGTTTCTCTGTTATCATCCTTTTTGTTCCCTTCTAATGAAAAACCTTCTGCTAATCCATTTTTAAAATAAGTAATAACACTTCTAAACCTATACTAAGAAAATTTTCTTCTAAGTTTTCTTTAATATGACAAACGAAGCAGCTTTGAGATCATCAATGGTAGGTCTTGCATTGGTGATGGTGTTGGTATGGTTATGGACACAGTCTTTAAAGAAGACGGTTATTACTTATGCGATCGGTGTTTCTTTGATCGCTGGCATAGTCTTACCTGACTGGGACTTCTTTGATCGGAGTTTCTCCCGCTGGACCTACCCTGTTACGGCCGAGGAAAGAGCCGCCGCTCTCTCCCGCAAATCACAATCTTCAAGGTTTAGAGTGTACCCTATGCGGATGGTTGTATACGTAGCGGTGTACGGATATGCAGTGTATAGGTGGTGGATGTTCGTGACGAAATGAAGCCATTTTGTTCCATCTTTCTGTATGTTCTGTGTTGATTAGCATATTATGTTAGCGGTGTTCCTTTAAATAAAAATTATTTTGAATTCTGAATGATATGTCAGACCTACTAAAACCAAAAGAAAAAAAGAGATCCACCA

>TCONS_00059832

CTTCGGACACTACTTGATGGAGCTCGATCAGTTGCTGGAGAACGCTCCGTTACCGTTCATTGTACAACACGCGTCGAGGATCTCGAGCGTCAAACAAAGAGTCTCGTCGATGAATCTGGTGCTTAAGTCTATACAAAGGCGTATTGATAACATCGATCACATGCTCTCTGCCAACAACATTCAAGAATGGAGCAGCTATCTGAATCGAGCCACCGCGATTCAGCTGATGCGGCTAGCTCGTCGTTAACCACCGCCGCAGATGTCCACGTCGGAGGCGGTGGTGGTGGAGAAGCTATGGCTCGAGGGTTATCGGCGATGCTAGAGTCCGTAATTAAAGAATTCGACTCTAAATCACTCGATGCTCTCAGTAGCCAAGACAAACTCTCCGGCTCGCTCGATCGACTTGTCCAAG

>TCONS_00057541

GAGGACACCACGGTTGACGGATTTGAAGTACCTGTATCGCCGGTTTCAAGTTACAACAATGTATATGCCGCAACCGAAGACGAGACAAGAGACCCACCAGCTGTACCGCCACATCTTCAACACTCTCTGTTAGGCCATACAGGTAGCACGGAGTTGCCTTGTGCACCACAGAGTGTCGTCCTGAACCATCTCTATATCGAAAATCGAGACCCCCCAAGATCCGTTGTAGCGCTTGGCTTCTCGCATCGGTTCAGGTCTAAGTATGTCACTGTGGTGATATACAAGCCGGTCCAAAGAAGAGGAAACGCCAATGTTTGAATCTTTTTGAACATGAACAACCCTCATCCTGATGATGATCAC

>TCONS_00029674

CCCCGATCACGCCGGATCTAAATATCCGATCCGGCATCAGATCTTTTCTATTCAAGATCTGGCACATTGGACATTGTCCCCTGTGTGCATATCAGGCAGGCGTTGCCCCCCCACGAATCCATCCGTGAGTTAGTCACGAGTCTTCTCGTGAGTCGGGAGAGATTCAATCCCTCCCGGAAATCGGGTACCAATTTGTGTTTCGAACCCTGGTGCTACTCATTTTGCATGCTGAGTCACAACCACTCGCCC

>TCONS_00064911

GGAACAATGAAGCTGGACGCATGGTGTTGAATCAAACCTTCACCATAGAACCGATGCTTACGGTAGGAAGCATAAAACCGGTTATGTGGGATGATGATTGGACCGTGATAACAGAAGATGCAAGTCTCTCTGCGCAGTTCGAGCATACCATTCTTATAACCAAACATGGAGCTGAGATATTAACCAACTGTTAGTGATCATGCAGATAAACATAAGTATGGAGTTGTTAGACAGTTTGTAGGCCATGGTATTGGACGTGTCTTCCATTCTGATCCTGTTGTTCTTCACTTCCATGTGTCTGGCGATGAAACTTCGTCAATGGGCGTCACCGATAGGGTACCACATTTTCCTTTATGGTGCGCTGAGTCCGAGTGTAGATCGAGTTCCACCATGCAGGTCCACGATACCCGTAATTGTCACTCTGGTCGTTACTGATTGTCGAAGACATCCTTCAAGTAGACATCCGATCAGAACCATACTAGAAGTATAAATATAGTGGGTGGTTTTGAAATCGTTTTCAGAACTTTTAGGAGAATCAAAACTTGGCCAGGAACCGGGCCGTTACAATTCTCCCCCACTTGAATGGAATTCGTCCCCGAATTCCGTGTGTCGATACTCCAACTTTAACATCCCGAACCAAAATGGGTAAACAACTCGTCCTTTGAAATCTAATGGTTCATGCAACAGACCAGGTTCCATATTCCTCACGGAGATCCAACGTCCTCTTAATTCCCAAATCGATTCTCCATCAGCGTTCTCATCCCTAGATTGTTTTCATCTCATATGCATTTCCACATCACCACCACTCTTGTCGACGTAATTCTCTGATGGTCATGTACCTCTTCTATCCTTTCGTACAGCAGCTCATCATCAACAATTATATCTGCTAAGCGGTTTCTTGGATCATCTCAGGCTCGAGATTCTGATCTCCCCCACTTTGGTCCTACATAAGGGATTCAACATGGTTGTTCATACAATCCTCATAGGGGATATCCATTTGCTTGAGTCATAGCTAATGTCTTGCATGGATTCAACTAATATCTGATCCATTAACCAACTTCCTCTCTAAGCTCCACACACATGTACATCACATAGGTTGATTGCCATGTCCTTGGCGACCCAACAACAAAAATATATTGTTATCATATTCCATTTCCATTTTGACACATAAAACTCTTGCATCAATCATATACGGCCTCACATCCCACTATTCTCAACTAACAAGTCTTAGTTGCAAAGCTCCATTAAAACCCTCTTGGCAGTTCCTCTTCGAATCCGGATAAATCTGTTTGCTGCCCACTACAATGGCCATTGCGACCGATAAACTTGTCCCATGATCTCTTCCTTCACCGTTGTGTGGTACCCAACCACATCTCCGTTGATTAACTTAGCGAGCACTTCATCTCAGGAAACTTAACGAGCTACTCCACACCGAACATATCCTCGTCCTCTGAGTCCTTCGGTCTGGCCATGTTTTCCTTGATGAACGAACCTCCTATCATAGCCCTACTAGTTGTACTGGTTATACTGGTAGTACGCTCCCATCCATTATCACCTAGTGTGGTTCTTCTCGCACTTCCACTCTTGTCACAACGAAACCGCCTTGTGTTAATGACGATGTCGCAAGTTCTCCTTGGGCTCTGAGTATGTGATGCTTGGTTAGCCTATGATCTTCTTACACCAAACAATAGCTCTGCACCCTTTTAGATTAGTTCATGATCCCAGTAATCTTGCACGTTACAACTATTCTTCAGATCGGTTCTCATTTCCTTCAGGAAGCTGTGAATCAGTTCTCGCCCCCCTTAGAGAGTGCCTGACAATCTCCTCAGGGGTTATATCTTTATTGTATTCCCTTACAATCATGTCTCTCGGGCAGATATCCTAGAACTAACCCTCCATTAGATATATGTCATTCTTCGGGAAATACTTTCATTGACCTATGGCCTCAACTTGTTTCACTGCACCTCTAATGTTCCTCTAAACTCATGATATCCGCCACAACCTTCACGCTCTCGGTAGTTACATTAGTCGGGTTTCCATCTCTGGCTTTCCTCCAACCACTCCAACTCATGTGTCTGGCGATGGAACTTCATCAATGGACGTCACCGATGGGGTACCACCTTTCTCTTTATGATGCGCTGAGTCCGAGTGTAGTTCGGGTTCCACCATGCAGGTCCACGATACCCGTAATTGTCACTCTGGTCGATACTGGTTGTCGAAGACATCCTTCAAGTAGACATCCGATCAGAACCATAATAGAAGTATAGATATAGTGGGTGGTTTTGAAATCGTTCATAACTTTCAGAAAAATCACAACTTTCAGAAAAATCAAAACTTTTCACACACTCTTTTCTTTTAGTTAAAACAATTCTCTCTTTTCTTTTTTTTTTTTTTTTTTAAACGTCGGAAATGCCGATCCCTTAGGACAGAACTAAGGGATCTTAACCCGCTCTGATACCAATTGTAACAGCCCGATCCCCCGGCGTCCGACCGGGGTTATCGAAGGGGCGTTATGGACACGTGTCTACTCATTTAGACAACCCTACGTATCCCGTTACTGGTCCCGAGAGACGAGCGCATAACCTTCTTTGAAATACCGTCACACCCACATCCATATACTTCTACTTACAGTCCTGCGCACAGGAAAAAATGGAAATGGAGGGGTGAGCAACAAGTTACTCAGTGAAGTGGCTCTAGACCAGCCTGCCCGCATGACACTCTATACTACTCTATGCGGGAACTAGGACCGACTAGCCACTACAATCAGTTAATGCATTTTATCATTTCCTATCGTCATCTGTAATTTCCACATTAACTTCCATTCATTTCTAACAACCTAGTGATCAATCAATACAATGATCTCACTCATTGCCACACAGGTCAAACCCTAGACTTAACCGACTCATCTTACCAGTCCAACTCGATCCTATGACACCTTTAACTCCCCGTTACCCGACCATGATGCACGTTTTTATATCCCGCCTCTCGCGGTTGGCACACGTTCTTTTCATCAGTGGGTAGTCCCAACTAGGCTTCTACCCCATATTCATGTGGATTCGCCACATCTCCTATGGGTGCTTCCATATTCATGTGGATTCGCCACATCTCCTATGGGTGCTTCCATATTCATGTGGATTCGCCACATCTCCTATGGGTGCTTCCATATTCATGTGGATTCGCCACATCTCCTATGGGTGCTTCCATATTCATGTGGATTCGCCACATCTCCTATGGGTGCTTCCATATTCATGTGGATTCGCCACATCTCCTATGGGTGCTTCCATATTCATGTGGATTCGCCACATCTCCTATGGATACCTAGCGTGAACTGCTGCCACACATACTTTTCCTCAGTGGGTAGCCCCCCACTAGGCTTCTACCCCATATTCATGTGGATTGGCCACATCTCCTATGGGTGCTTCCATATTCATGTGGATTCGCCACATCTCCTATGGGTGCTTCCATATTCATGTGGATTCGCCACATCTCCTATGGGTGCTTCCATATTCATGTGGATTCACCACCACACGCCACCGCCGAGGAGGAGGGAGAGAGGAGAGAGAGAAGGAGAGAGAGAGAGAAGGAGAGAGAGAGAGACACGGCGCAGGGGAGAGAGAGAGAGGCTTGAGAGAAGAAGAGAGAAGAGGAAAAGTGAGAGAAGAAGAGAGAAAAGGAAAAGAGAAGCTTGACGGCTAGGGTTTCCTAGTCTCTCTAAATCTCTGCAGAGCTTCGCTTAGGTTTCAGGATGAGAGAAAAGTGAGAGGAGGCAGCTTCATTTATAGGAAAAGGAGGAAACCCTAGGTCATTTACCCTAATGGACTGCAGTCTTAACGGGCTCTCCTTAATAAATTTTTGGGCTAGGAACCGGGCCGTTACACTTCAGCTACTTTCTTCTGTGGAGATGTCGACGAGAAAGCTAAAAAGCTAGTCCAGGTACAATCAGTTCTATGAAAACTCTGGTTTATAATAATAGTTTGATGAGAGTTTTATGACTTTGAGTGTGGTGTAGGTGACAAAGGAGTCTCTAGACAAAGCGATATCAATATGTGGAGCTGGAGTAAAGTACAAGAAAATCGGCAAAACCATTCA

>TCONS_00059723

AGGAGGCGTGTTGGATCTGAGATACAGAGACTGGTGGAAGTAGACTGCTACAGAGGAAGTAGACATAGACATGGGATGCCTTGCAGAGGTCAAAGAACCAAGACCAACGCTCGCACTAAAAAGGGAAAGAGAGTTGCCATTGCAGGAAAGAAGAAAGCTCCTCGCAAGTAGAACCTGAGATGTTTGGTCTACGCAGATCCGCAGCGACGTTATTCGACCACAGCCAGTCTCTGCTTCGCAATCTATCGGTGAGTTCTTCGTCTGCTCATTTCGAATTCGAATAGTCGTCTCAAATCTCTACCGGAAAGATGTCGGCTTTCCGACGTTTCATCACTCTCTGCTTCGAGATTTTGAATCAGGGTTTCAATAGATCGAATCGATTGATCTGGGTCATGTTAAAGTATAAAGCTTTGCATATGTTAAGTTAGAATCATAAAGTTGCTAACTTTTTTTTAGCTTTGTTGATTTGATCACATCCTGTTTCTTCTTACATATCTGTTGAACTGAAGCCGTTAGGTTTCTTGCATTGATCGTGTCTGAACCAAAGCTTTTCAAACCCATTACCTTGTCCGAAGCATATATCAAACTCAACGGTCTCTTTCTATGCAGTTTCATGGATTGCGTGTGCAAGGAATCCGTGTGGGAAACGCAGAGGTTCCAAACCACAAGCCACTCAAGACGGGTCTTCAAGAAGTGTACGGAATAGGACGCCGTAAGTCTCACCAGGTTCTCTGTGGGCTTGGGATCACTAACAAACTTGCCAGAGACTTAACTGGGAAAGAACTCATTGACCTCCGTGAAGAAGTTGGCATGCACCAACATGGTGATGAGTTG

>TCONS_00081829

CCGGTATCTGGTTTGCCGAGTGGGTAGAGTTCGTCGAGAAACGCAGGCTGGAAGTCGAAAAGGTAACGCAGCTTGGAGAAATCGACATCAAATCCCTGACTCTTCACGATCTGTTCGTTCATAATACGATACTCCTCCGCTAGTGTGTGATTGTGAATCCGCTCGTCAAGTTTCCCGCGGATCTTCTCCAGCAGAAGCTCCGTATCTTCGGGCTCGTTTCCAGGATAATACATGGTGCACGCGCTCACGCATTTAGGATCCCACGGGTCAGTGATGATCTCGTAATCGATTGTGCTTGATGAAGCCATCACAAACCCTAGTTTCTCAAGTTTGGGGTTAAAACGAC

>TCONS_00069471

CTCGGTAGAACTGAATCAACATAAACCTTAGTAGAAGGCAGGTCTCTAGCACCTCGTGCTAGTTTGTCTGCCATTGTGTTTTGTGCCCTTGGAATATGTTGAATGAAGAAGTTGGAGAAGTGATCCTTACATCGTAGAAACTCTTCCATGTGAGTAGTGAATGCCGGCCATTCTGTTGGTGTAGACACCATCTTCACCAATTGTGAGCAGTCCGTTGCAAACACCACCTCTGAGATATGTAGGGTCTTCATGCACTCCATCGCCCATATCAAAACTTCACATTCTGCAGGTAAAGGTGATAGACTCCTGCGAATAGACATTGCACCCATCATAGTATCAGTGGATCTTTCGTTTCTGTAAAACCATCCTTGTCTTGTGAAGGAATCTTGTTCTCTTCATGCACCATCAACACAACATCGATCTATCCCATGTAAAATAGGGGTTCCAACATGGGAAGACTCCCTTAATGTAGCTTCTTTAGTCTGGGATTCGGCCCATAACACCTCTTCAATTTCCGCAGTGCGGAGAATATAAGGAGGATCCCTAACTTTGTTATTAAAAAATTTATCATTACGATTCTTCCAGATATACCATAAGATCCATGGGAAATAACTCAAATCTGGTTCTTTTGGAAGGCGCCAAAATAAATAATCCATATTGGTAAAAAGAGATTGGGTGGGAAAAACCCCTAGAAGGGAAGGAATGTTAGATAACGCCCATGTTTGTAGTGCCAGAGGACATTCAAAAAGCACATGGTTAACGGATTCTTCCTCTGCGCCACATATATGACACTGTAAATCACACTTAATCCCCCGAGAGCGAAGGTTCTTTGTAACTGGCAAAGTGCCAGAAAGTATTTGCCAGACAAAATGTCTTAATTTAGGTGAACACTTGAGTTTCCACGAATGAGCTAACAATGGTTTAGTGTTAGGCCCCCATTCACAGGCTTGAAAACCTGATTCCGGGAATGATATCTCAGTTCGATACCCTGATTTAACAGTATACCTGCCATGATCCGTAAACTGCCAGCTATATGAATCTGCCTTAGGGTACCGACTAATAGCTAAACCCTGAATAATCCTCACATCATCCTTGTGGATATATGCATTGAGCAAATTCAAGTTCCAAGAAAGATCAAATGGATTGATTAAATCCTCCACCTTAAGAAGTGGATTCAGGTATTGATTTTGAAGTTTTGGGGTTGCTGACCTCGGGCGATGAGCAGGAATCCAGGGATCATTCCATACAGAAATGCTTGAACTTGTACCCACTCTTTTGATTAGCCCTTTATTAACCAGAGATCTAGCTGAAGTAATACTTCTCCATCCATAAGAGGGAGAATATGAGCGTATCGGATCCATTGGATCTGATGTCCTATAATATCTTCCTTTAAAAACCTTCGCAAAAAGGGATTTAGGTTTATCAATAAGCCTCCATAGTTGTTTTGCTAATAAAGCAGTATTAAAATTCTGAATATCTCTGAAACCAATACCCCCTCATTTTTGTCTTTGCACATCTTATCCCATGAAAACCAATGCAAACCTTTTTTATTTCCACTCCCGCCCCACCAAAAATGAGCGACC

>TCONS_00052534

ATGGATTTATAACACTCCCCCTTGGATGCCATAACCATATCGGGCTTGTAATGTGCTAACGTTGCCTCGTTAAAACCTCTCCCGGAAAACCCAAAACCCAATGTGGTAAAAAGGGAAACCAAGGAAAGGAAAAAGAGTACAACACACATTACTCCCCCTGATTTGGACATCACTGAAGGTCCTTGAGTCTTCGCATGCCAATCTGCTGCGTGAGCTTCCTGAATGTGCTGGTGGGAAGTGACTTGGTGAAGAGGTCGGCTGAGTTCTCACTTGACCGGATTTGAGTGATGCTGACCTCCTTGGCTTTCTGCAACTCATGTGTGAAGAAGAACTTGGGTAGTACATGTTTGGTT

>TCONS_00078695

AATGGAATTAGACTAGTTAAGTTCAGTTAGCAATTCAATCAGCCTTAGCTAATAAAGCAAATATCCTTCCGCTTCAACCCAATCTTACTCGAAGAACTATTTATGTTTGCCGGAAGTACGGTCAGTGTGAGGGGTGGCTCTGTACATGTGCTTTTCAGTATTCAATAAGCCAGGAAGGAAGTGTGCATTCAGTTGTGCTTCTATGTCAGCTTGGCTTGTTGTTCTGGATTGAACTATGACTCCAAATAGACTCGGGATTGGAAAAGGCCGTTGACATTCGTAGATGAGAATTTAAAACGCCAATAATTTTTTTTTTAACGCTGATTTACTATATAAATGAAGTAATCTATTTTTCTTTTTTTTTATTACTTCATTATGGAATTAAGAATCCAGTGGAGTTGGAGCATTTTGATTCCATAATCCATTTTACTTAATTTTTGAAGAAAAAAATGAAATTTTACATTGGAGATACATTTAGTTGAATCACATGTAGTACCGG

>TCONS_00010615

TCAGAGCATTCAGCAGAGCAAGCTAAATACTTCAGTTCAGTTAAGCTTAGTTAAGCTTGAGTGTTTACCTTAAGGTTTTTATTCGAAGCAGACCTTCTCCAGATTTGCGACCTGCACAGTGAGAGAATAAATCTCAGCAGTGAGGTTATCAACCGCCAAACTGAGGCGGTTAACCTCTGCCTGCAGATGGAAACAAACACACCGTGTTACGGACTTAAAAATAACCAAACTTTACAAATCTTTACACTACCTATTCGATACTCTTAAAAAGTTCACTAACCTCAACTGTGTCGATATGTTTATTGAGATTGGGCACCAACTTGATCACCTGCTCAGCCTTCTCCACCCGCCTACGCAGACTTTCGATCTCCTCCTGCACACCTATAACCCAAGGCTGACGGTAATGAAACCCATCAGCCTTGTTGACAACATAAACACATTAGACTCGCGTCACTAAAACATAATCTATACTTCTTCAAACCCATCTGCATTGTATCTCTTACCTCGTAGTTGATGCACGTGAAGAAGCGTTTCCCAGGCAGAGTGTCGTACTTCTCCTTCACGCGAACCTCGTCTCGGATTCTCCCACCACAGGGACACCTCCTGGGAATCCCATATTCTGAATCGGCCACGTTTCCCATACTGTTGTAGTACTCCTCTTGCCTCTTTGTATTTCTTCTATCTTCCACGGGATCCATCTAAACAACAAAGATAAAACAGATTACGAAATAATCACGGAAAAT

>TCONS_00019608

CTCCCAAACAGCAGCTTAAACATTTCAATGGGAGAAGAAGAGTGAGATTTGGGATCACAGTAAAATCAGTCGACAGTAGCTGCTTCTCAAATCTCAACCGATCCGTTCTGAGATCGAAAGGAAGCTTTGATTATCGTCTTTGATGGCGAAGGAGCAAATCTTGAGGCCTTGGTTTCTGGATCTCGTACCGGCTTTGGTTATCTTGCTCGCCGCAGCTCATGTCATCGCCTTGGGTTACTGGATTTACAGATTGGCGACTGATCGTCGTGCTCAGAGTCAGAGAGGAAAGTTTCACTGAGGAAACAGTCGAGAGTAGGTTTCGTAAGCTGCATAGTTCCATAAACTGGCGGGGTTTGCTTCCGATCTGATGTTTGTGATCATTTTTATTTTTGTAGCTTATGATGTCGTTAGGTGGATGGATCATATATCTGTTTACTAGTTTGTTTCCACCTATGCTTGTAGCTTCTCTCTGAATTTTTATGCAATTTTTTGTTTGTTTGTTTTAAATAGATTTTGTAACGATTTTCATTGTTTTGTTT

>TCONS_00068558

GCAGATTTGGTGAGACGATCGACAATCACCCAAACTGCATCATTCCCACGGCCGGGTGCTCGAGGTAAACCAGAAATAAAGTCCATCGCTACTGCATCCCATTTCCATTCAGGGATAGGTAGGCTTTGAAGTAAGCCACATGGGACTTGGTGTTCAGCTTTTATCCGCTGGCAACTCTGACACTGAGAAACCCATATGGCAACATCTCTCTTCATCCCTGGCCAGTGGTAATATCTTCTGATATCTCGATACATCTTTGTGCTCCCGGGATGAATGCTTAAGAGAGAATGGTGTGCAGTCCTTAGAATCTCCTCTTGAAGTCCTTCTCCTTTCGGAACAGTTACTCGACCATTGAGTAACAAAGTTCCATCACTTGCAACTTGATATCCTCCGAAGTTTCCTTCATTCAACTGCTCTCGTATCTTCTTCAGGTTCACATCTCTCATTTGATATTCTCGGATACGAGAAAGTAAACCAGCTTGGCTTACTGCTTGTAGTCCTAGAGGTTCTCTTTCCTCTCCTTCTAAGGCAGCCAAACTAATCATCTTGAATTCCGCTTCCAGGTTCTGGAGTTCTTTTTCAATATCTACATCAACTTTCCTTCTGCTCAACGCATCTGCAACAACGTTGGCTTTTCCTGGATGGTATAGGATCTGCATGTCGTAATCAGCTACGAACTCCATCCATCGTCTTTGCCTTAGGTTCAAATCAGGTTGAGTGAAAAGATACTTAAGGCTTTTATGGTCAGTGAACACTTGGATCTTCTCACCATACATGTACGATCGCCAAATCCGAAGGGCAAATACTACTGCAGCCATCTCTAGGTCATGTGTCAGATAATTCGCTTCATGCTTTCTTAGTTGCCTAGATGCATAGGCGATTACCTTATCTTCCTGCATCAGCACACAACCTAAACCAACATGAGATGCATCCGTATACACAGTGTATGGTTGGTTAGGCTTAGGCAATGCTAAGATAGGTGCAGTCGTCAAAGCTTCTTTTAACTTTTCGAATGCTTTCTCGGTTTCTATACTCCATTCATAAGCAATCCCTTTACCGGTAAGACGAGTCAAAGGCTTTGACATGCTTGCAAATCCTTGGACAAACTTTCTGTAATAGCCGGCCAATCCGAGAAAACTTCGAATCTCGGTAACAGAAGTCGGATGCGGCCATTCCTTAATAGCAGCTATCTTTTCTGGATCGACGGCAACTCCTTCTTCTGACACTCGATGTCCTAAGAAGCCAACTTCTCTTTTCCAGAAACGACATTTACTAAGCTTAGCAAAAAGCTTTTGATCTCGGAGACGCTCCAAAACTAGTCGTAGATGCCTTTCGTGTTCTTCTGCACTTCGGGAATATATGAGGATGTCGTCAATGAATATGATAACAAACTCATCCAGGTAATCCCGAAATACTTCATTCATCAGTCGCATAAATGCAGCCGGGGCATTGGTAAGACCAAAAGGCATTACTACGAACTCAAAGTGTCCATAGCGGGTTCTAAAGGCGGTCTTCATGATATCGGTTTCGAAAATCGGAATCTGATGATAACCTGAAGCTAAATCAATCTTCGAAAACCAGCTTGCACCTTGTAACTGGTCTAATAGTTCATCAATTCTTGGGAGTGGGTACTTGTCTTTTATGGTTACGTTATTGATTCCACGATAATCAATACATAACCGCATGCTTCCGTCTTTCTTCTTTACAAATAGCACGGGTGCTCCCCATGGTGATGAACTAGGACGTATGAAACCTTTGTCTAACAAATCAGCTAACTGCTGCTTC

>TCONS_00022106

GTTTTTTTTCTTCATAATTTAGTTTATATAAGTTTTTGATTATTCTTATAGATTAGCTTTCATCCGAATCACATACGATTTCATCATTTGAAATCTAATCCTTCCTCATCGTATATATAAATAGGTTGTTTTGAGCACATCTACTCATCACATTCTTCTTCCCAAACCTTCATTACAAATTCGATCTTGCAAGTAAAACACTTATGGTGGGTTTCAACTCATTATATAATATGTAATTTTGTGACTATAAGTTTTGCATTTCTAATATCTATAGATTAAAAGTTATAATCTCTTTTAATCCATTCACTCCGTGCAGGGCGCGGATATCACCTAGTACAGTAGTAATTAAGGGCATATGAATGGACAAATTAGCCGGACAAAGTCGTAAAGAGATGTAAAATCACTTTTTTCGGGGCCAAACAAATAAGAGAGATCGAGCATAAGTGTGTGGAGTAAAATCATCGTATGAGGTCCATTTCTTTATATACATTCATTTTCCTTTGGTCCACCACCCTTCTTATCCCCTCTATCTATTTTCGTACCTACTTTATTTATAGTATTTTTTTATTATTATTATATATTATTACGCACGTATAATCATATAAATTTGATTTAGCAGTTGACCAAAAGAAGAGAAAATAATAATTTAACGACAGTTTATGATGCATGTAAAATGAGGTACCTTGCTGGAATTGGGAACCAGTTTCTGTAACGTCTTCATCCTTTGATTGATCTTATCTCTCCTCTTCTGCAATAAAACCAATCAAGAGACAATCACCAATGTTTAATTAATGAATATTAACTCTGAAATTTCATTCCTATACAAAGAAAACATTAACTATGAAACATAGTCGACGAATAACAATATAATTTTTCCTCTCGAAATAAATAAATAAGAGTAATAGGGTTGTTGTGACAAAAAAAAAGAGTAATAAAGGGTTGAAGGAGTGAATGAATGACACTTTCGGATTGGTTGTGAATAGCAGCGGCTCTGCTTCTCTTGGTTGAAGCTGATGATTTTCCTCCCGTTTGCTTCTGTTCTTCTTCTTCCTCCATCTATATATAGCGTTGTAATTCAAAGTTATTTTTTAAAAGTGATGTATAATAAATTAATTAGAAGGAATAATAAAATGAGAAGCTAATTGCCTGCGGGCGGCTGTGGCAGACCGAGTCATGATCGTCGATGTTGGTGGTTTTGGGGAAAGGCTTCCCGCCGGAGCTGGAGTTATCATCCAGCGATGTTGAGGTGAAGCCGTAGGTGTCAATGGTCAGGCGCTGGCTCCCGCTCGCGCTCCACTCGGGCGCCACTCTGGCTCGTTTCCCACAATCCATGGGATGACCATCGCTACACGAGCCCACGCCGGTGGCGGGCTGGCTCTGCTGCTCGGGGAG

>TCONS_00041717

GGAGAGTGCTGCACGTTTCTTTCAACCTAAGCCACTCTCTGGTCCATGTTACTTCCCTTGTCCAGGTCGTCCATACAAAGATCTTGCATTGCGTCCATCATCCAATCCGTCTATTATTCCCAAATAACTAGATAAACTAACCTTTATGTTTTCAGGGTCTTGCCGTGGAAGAGTGTATCTTGGCTAAGCCGGCTCATCTTGGAACTCCCCGGTTCACAAGCATGTAACCCATTTTTACCTCAACTCTCTCTTGGCTCACCACAGCTAAGGTACCAAGTCATCTCAGTTTTCAGAAATAATTTCGGTTTGGGAACTCAACATCTT

>TCONS_00049747

TGAAATAAGTGATGAAACGCAAAGGAGAAGTGGTGGCGTGATGAAGCAGACGCGGTGGTGGCGTGAGGATCGGAGGCAGTGTGACGAACTGGTCCTCTGAACAACGAAATGAATTTTCACAAGAGCCTGAGATGACTCGGTGGTTTCAGTTTAGATGACTCGGTGAGTTCGAGAAGAGGAAGATGGAGATTGGTGAATGTGTCCAGGCTAGGCGGCGGTGGAGGCGAGACGAATGAAAGCTTACCGGTAGTGGACGTGTGATACGGAGGTGAACCGGAGGCGGAGGTGACAAAAGCGGTGGCGGAGGCTCAACGTAGAAA

>TCONS_00016572

CCTTAATATTATTGAGAGCATATTTTATAATGATAGGGGCAAATCGGTCAATATAACATAGGATCATTAACCGAGTCCAGAAAATCAGGTGAATATCGAAGGTTAGGTTCATCTAGGGAAGTAGTTAAACCATTGATGATACTGATGATGCGATTTCCCGTGAGTATGAACAATCCAAAACGTACTAATTATTTCCAACAATTTGACTAATAACCATTCTAAGAAAACAGTTTTCAAATTAAGTAACAATAAAACGAATCATGTGAAAGTAAAAGAGTCCTACTTAATAATACTACTGCTCTATTTGGTAAGCACTTAACATAATTGATTAATTGACTAACTAAATTCTCGTGATTCTGTGATTTCAATTGACTAAACTTCCAAAATCTAGTAGGAAAAAATAAGAAATACGTGAAAGACGCCAATAAATCAGAAGTAATATGAATTTTTCATGGATTTTTATTGATCAAGATCGAATCGATGTATAAGTGGAGGAGCTCAAGCTTTAAAACATAATCAAACTCAAGCGACATATTGTTGACAAATTTACGTTTTGAAAAATTAACCAAAAGATTAAACAAACGCATGTAAAATATAAGAATTTAAATAGATAGTTGTTTAAATACAAGAAAGAGTTCATACCAAATCAACATTCTCGTTCTTGATATCCTCAAGAGAACCACCACCCACCATGCTTTCCTAGCTCTGTGCCTTCGTTAAACCCAAAACACAAAGGAAAAGTCGTTAAAATTATTCAGAAAAACTGCTGAAAAGTTAAGCAGAGGAAAGTAGCGATGATAAAGATATATTCGAGAATACCAAAAAAAAGGAGAGAAAATAAAATTGAAACGAACCAGATTCTTT

>TCONS_00015045

CAGCTCTAATACAAAATTATTCTCTGATCCTCTCTTCACTCAATAAAGGGTCTGAAGCCATCAAGACACAAATATTACATGAGAATCACACAAAGCAATGTCTCATAGTTGGGAAAAGAACTAAGAAACATGACCAATAACAATAACAAATAGCTTTGGAGTGTATTAAAAGTGACAAACATACATATCCACTACTGAAGCTTGAGTTCCATGATGTTCTTGGTCTTGGGAGGATACGCCAAGTAAACCTTGACCACCCTCTCCACAACCTCCAGCTGCTTCTTGCCTTCAGCATAAGCCTCCTCCACCTTAGAATGGTCGGTGAGATTCTTGTTCATGCGGAACCCATCCAAGGTCCTTCTCTTAGCGTACTCCCTGATGTTGTAGTCAGGGTACTGACGCCCCGCTCGAAGCAGGGAGCGACAGAGGCTGATGACTTGAGCTTTATCTACCATCGCAGTGAATGAAAAC

>TCONS_00056516

ACCTCAGCTAATGGTTAGTATACTCAACTACCAGCTAGCTCCAAGGTTGATTCTGAACACGAACCAAGTCATACAAATCTGAGGTTCCGTTTCCCTACCCATTCTATCTAGATCTATGCAACATTGCTAGATCATACCTTTGCCACATCCACGGAGCTTGTTAGCTCATCGGCCCAGCTACTCTAGCTGAATGAACTCATAGACCAGCTGATCGAGCTGTCACACTCGAACTGAGCTAGGACCGAGCTATGGCCAGCTGCCATATACTGCGTCCAGCTCCTTTACACACACAAAACAACTCCCTTAGGTACTTAGTCATTCAGCCAACCATCCCCACAGACATAAGTAACCCTTGAGAAGTCTTCAAACAGCTAAGGACATGCATCTGGCCCTTACCGGCTCATGCACTGTCCCACATCCTTTTGCCTTATCAACTTATGATACCAAGTCCAGCTCATGGAATAACAACGCCCATCAGATCCTGAGTCAATCAACCAACCACCCCACATGACCCAGAACTGATGAGTCTTCACACGTACCTAACCGGCCATGGAACCATGTCCCAACGAAGCCGATCTGTCCTCCTCTTACCACTGGTGCATCCTTTGATCAATCAGATCAGACACCTTGATCCATCACCTATGGATCAGGTCATCCATCCTTGCCAAAGATCAGATTACACACGGCCTTCTTTAGATAAACACACCGGCCCCATACTTTTGGTCCATGCCACTTAGTACTGGCCTTACTCGCCTCCACGGCTTGCTGCTGGTTCCAAATGAATTGGACCTTGCACCCCACATGGTTCCTCATGTACTAGGATCCCCATGTGTCTCCTCCATGATTCGGATCATTGATTCACACCATGATCAGAACTAACACACACACCATGATCCACCATGGATCACTATCCAATGAGTGGCCTCTAACTTCCATCCCACATGGATGAGTTAGATCAACTAAGATCCGCCCCTTATCTCAGGGTCCTGGATCCTTAGTTTACATCACAACATGTCACTTCGTAGCGACGTTCCATACTTGGCCTTAACCGCACCACAAGAGACAGAAACATAACCTTGAAAGTAATTATAAAATCAGTTACCTTATACATGATCTGATTAGATCAAGTGCCTTTCCTTATTATGTTCGGCCATGC

>TCONS_00024829

GCGGTCCGTTCTTCCTTGATATGGTTACTAGCAATCCACATTGTTCTTAACACTCCCCCTTGGATGCTATAACCATATGGGCTCGTATCATGCACGATGTTGCCTCGTTAAAACCTCTCTAGGAAAACCAAAAACCCAAGGTGGGAAAAATGGAAACCATAGACAGGAAAAAGAGTACAACGCATGATACTCCCCCTGATGAAGGCATCACTGAAGATCCTTCAGCCGGCGCATCCCTATCTGATGAACCAGCTTCCTGAACGTTGAGGTAGGCAGAGACTTGGTGAAAAGGTCGGCTGAATTGTCGCTTGATCGAACTTGAACTACTTGAACCTCCTTTGTCTTCTGCAAGTCGTGGGTGAAGAAGAACTTGGGCAGGATGTGCTTGGTCCTGTCTCCCTTGATGTATCCTTCCTTGAGCTGAGCAATGCACGCTGCATTGTCCTCGTAGATGATCATTGGCTCCTCCTTCTCTTGTCCCACGGCCAGACCACTCTCTTTTAAGACATGGCCGGTCATGTTCCTCGACCAGACAAGCTCACGGCTTGCCTCATACATGGCTATGATCTCAGCGTGATTAGATGATGTGGCCAC

>TCONS_00063755

TTCCCGCATTCGACGAACTAAGTTATCTATTCGGGGATCGATTACTTCTTTCTTCCACTTTGGATCCCCACCTTCATCTGGGATTGAATACGTAACAATAACCTCAAACAAAGAACACAAACATTCATAAGTACACAAAGTTTGAGCGTCTTAACTTCTGTTTTGCAAATAAGATAGACAACAGCATTGTACCTCTCTTTGGGTTTCCACCAGAAGTATGTCTTCAAAATTCAGAAGTGCATTTGTTGAGTCGCATCCTTCTGGTTCTTGCAAGAAAGAAGTGGTTTTATTAGGTTCGGGAATTTTCTCAAGCAATGAGGGGATAGCTTTAAAAGCAAAAAGTTGCAGCGCCAGAGGGAACCCATAACACGCTGTTGACTGCTGTTTCAAACGCAGGCGCATGACCGAAAGGGATTTATCCATTTTAGAAGGATCAGAAGGTTGAGGTGGTGTCAATCTAGACAGAGTGCTGACGAATGCCTCTCTCCCCCATGGATATTGAAGAAATGATCCGGTGTCTTCCAGCATCTCGACGTAAGCAGGCGTTACACGAAGAAGTTTGTGACCACAAACCAGGAGCCCATCTACAAGCGCAATGAGAGCTAGTGGCAACCGCTTCCACTCAGGAAGACTAGGCTGTTCAAGCATACGAAGAACATCTGGTACAGTCACGTCTTCATCTTCAGTTTCAAACAACTCTTTCCACATACGTCCAGGTGTGGCATCGATAGATTCTGACCCGTTTCCAACTTTTTTCCTTTCAGGCTCGCATTTCAGCCCTGTGATGTCTCCGAACTCACGTAGAGAAAAGCGTAGTGGCTTGTCTGCAAACAAGAACCAGAGCTCATATAGGCGCATCGTAACCAGCTGACGGCTGAGGAGAGAATGTACAAGTTTCGCAGAGTTTGAGCAGCGCGATACAGGTAAGTGGAACAGAGCTCCAAACTGACTTCATAGCAGACAATTCATTTCAGGGGAGCCGCTTAGCAACTTCACTAACGAGCCAATGATACTCGCCTTTGAATAGATGTTCAGTCGAGGTTTGCCAGGATAGCAATTACGTGCGAACAGTCTTTCCGGGAAAGTCGGCGTGGTGACAATAAATTCCGGCTCGGTGGATGCAGCTTCGTCTTGTTCAGTGGATGCAGCTTCTTCTTGGTCGGTGGATGCAGG

>TCONS_00070327

ACTGGGCTAAAAACCCACTTTGCCCATGGGCTAACTGGTCCAAACCAAATGGGTGTTGGGCTGGCCCAAAACACTTAATTTCTAGGTTTAGGCAATTTGGGGAAAATTGAAAATTCACGTTCTCTTCTTCTCTTCTCCCATTAGACGATTCTCTTCTTCTCTTCCACTATTTCTCTTCTCCATTCCCAGATTTTCGTAGCCGCGAGTCCTCCCTTACGTCTTCCTTATGAATCAGTAACCATTGCTCTGTTGTAATCTCCCTTACGTCTTTCACCATTGCTCTGAAACTTCTTTTAGCTGTCTTCTTCTGTGTTTCTCTTCCTTGTTATCTGCTCATAAGGAGGTTCAGCTTTGTCTCCATCTCTCCGCG

>TCONS_00042475

GCTTGCAAGGCTGACACTGGACCCAAACCATGTCGCTCCCGGAGTCAATCACCATGTACTGATCTCGCGGCGGGCTTCCGACGCCGATTCTGACGAAGTACTCGCCGCTCCCTTGGTCCATGCCGGAGACCACATCGGAGCCGAAGTCGTCGACTTCGTACTTGGAACCGTAAGGTACTGTTTTGCCGGAAATGCGGCGGAGGATGGCGGCGACTCGGTCTGTGTCTCTCCGCAAACGGGCGTGGAGACGGTGGTGGTGGTTGCGGTAAGAAACAGAAGGGAATCTATCGCGGTGGAGGAGACGAAGAGTGTACTTAGACTCGTCGGTGTTGTTGAAATCGGGAAGGGTTCCGGTTCCAGTGACGGTGAGCGGTTGTAAAACGTCGATGATCTGAAAATCAGGGAAGGAGATTGAGGAGGAGGAAGAGAGATGGAGATTGAGGAGGAGGAGGAGGAGGAAGAAGAAGAGAGGAAGCAGCATTGTTTACAACGCCGAACACAAAGACCCGTGTGAGAGCGTGAAGAGTGGTTCTTGGATTCTGGGTTTTAATGTCAGAAGATGAGTTTTATGTTTGGTGTCTTCTTCTTCCTCTCCAATTTCTTTATTGGAGGTGAGAGACCACTTGAGACTTGTG

>TCONS_00024621

GCCGCGTCAAACCCGTCTTAGAAGCACGGCCACTCTTCAGATGGAAAGCAGGATTGGAGCGGTTACCATCTTTGGAAGCTCTTTTGGGAAGGGGGTAGTATAAATAATACGGTGAAAGTATGAGATTGGGAGAGTTCTTCATCCCTTATTTATAGATGTGAGAATTAAGGTATTTGCAAAGTAGTGGTGGGAAAGAGTAATTAAAATCAACTTAGTGGGAGAGACGGTAGCAGAGAGTTAAAAGGAGATTCTTTATGACATGTTTGAATGACGATTAACGTTACAGATACATTCTCACATAGACGGCTGCAAAATTAGGGATTG

>TCONS_00037441

TTTAACCGAAAATAAAAACCCCAATTAAAAGATTGGGGTTAATCATGGTCTAATATCGGTAATTAGAGAAATAACTAACTAATATATAAAAAACTAGGATCGACCCGTCCTATGGGCGGGATGTATTACATTTGTATTTTTCATTACGAATAATATGATTGTTAATATGCAAATCTAATGGTAGGGGTTGGTTTGCAACTGATATCGTAGTTAGTTTGTTTCTGTCAAGTTTAAACCGATGGATGGATGATTGAAAATGAAAATCATGTTCTTTGAATTATTTTTGAGTTATTGTACTTTGGCTTTATTATTTAATCAAGTGTGGCTGTCTGCTGCATATAACAGAATGTGATTTCGGTTGAGTGAGTGTATTGGTGGAAATAACCTAAGCTTTAAAGGTTGGCTATCGCTATGTATCTCTGGAAATAACTTAAGTCTCGTAGTAGTATTTCTCGTAGTAGTCTAATTATAAATTGTTTTCTACACTGAAAGTAGAAGTGGACCGTAAGGTAATAAAGTATTTTGAGTTAATTTAGTTTGTGCTGGTTAAGGTAGTAAACCATAGAGTCATGACAACAATTGCATTTCATGGAGATTTTGTATTGTGTGTAAGGTGAGTGTTAGGATTGTAGTCCTACTTGTGCTTTAGAATTTAGAATAAATTAGATACCAATGACTTGTATTTTATTCACTTTCATAATAGTTAACTTAACACACAACCAAACTCTCTAGACAAAAGTTAAAATTAAAAGAAGAAAATATTTTCTTTTGTCTCAATTAGCTCTCGTGCTTTTCTGAATCGAATGCTCA

>TCONS_00054118

GCTGCGGTGGCGGCGATGCAGGTAGACGGGTTAGCGGCGGAGTGTAGAAGGGTTAAAAGGCTAGAAGCGGCGGAGAAGTATGTGGATAAAAAATAAATCAACCTGAGGGGAACATGATGTCCTTCCGTTATTTTATAGTTTTCAATCTGAATTTCAATTCCGCCGCTCGCTTTTTCTTGCTTCTACCACCGCTGTTCCCGTCGCT

>TCONS_00063308

TTGCTCTGAAGTTTGAGCATCGAACCGGCACGGGGTTGAAGAAGACGTGTTCACCCTGAGCGGGAAAATTCCATCTCGCCGACCACAAAAGAGGACACTGACCTTCCAGAAACATCTCGATGCAAGTCACCAGATGTTCGAGGAGTTGCCTTGCACACAATTTTACAAAGAAATATATCGTCTTAGTCTCCCACCTGGGATGTCTGCGGTGTAAGTTATCTTTCCTAACTTTCTAATCTCTTTCCGTTTTTGTTTGAAACATGTATATATATTTATAAAACGATTGTTTGTAAATAAGGTTGGAGATTTCAGTTGTAGATGGTGCTGCAGCAAACATCTAAGTCTAATTTGGAGAATTACTGATGTGTAAAGGTTGAGAAAGCTGAGAAAAGGTGGTTTTGGACAAGTGTTTGTGGGACGTGTATTAGTGGTGGTAATGAACATAATACATGAGCTAGCATCTTAGAGATGCCGGAGCTTCGCCATGGAGTCCGTCGTGGCTCTGATCGTCTTACAGACAAGAAACAGAGGACCACGACAAAAGGGTTGCTAGCGATTAGTAAAAATAGTGGTGGCTCCAACAAGGCGGCTGCACATGAAGAAGAAGGAAATACTCCTCCTTTCCCTAAAAGG

>TCONS_00082624

GCAGGACAGAAGATATCGGAGACCGCAGGGGGAGCTGTCAATGTTGTGAAGGAGAAGACCGGCATGAACAAGTAGCGGGAGAAGTCTAATGTGCTGATGGACAAGGTCAAGGATGCTGCTACCTCTGCTGGAGCGTCTGCTCAAACCCTAAAACATAAAACACATCAACGTTCATCTAAAAAAATATCTTCCTAAGAAAAATGGCAGACAACAAGCAAAGCTTCCAAGCCGGTCAAGCCGCTGGTCGTGCTGAG

>TCONS_00023295

CTGCCGGTCATTCCTCTCTCTCTCTCTCCCCCCGTTTAAGCAAACTAAAAGACAAGAAGAAAAGCTATGGCTGGTATGCTTCCCGGAGTTGCGTGCGCGAGGAGACGGCGTTTCCACGGCGGTGCACCACCGATTGACTTATCAAACACGGCTTCTGTCGCGGCAGCAACAGGACAGGTCTGGACTCGGCGACCATCGTTCTCTCTCTACACTACCAATAACGACCAAGCCTATGTCTCCTTCTCGGAGAGAAATGTTAGGAACAAATCGTACAGTGATGATAACGATGAGAAGCTCGTCGGAGCAGCCAAAGAAGCTAAGGAGAGGCTGGACGAGCGGCTGAGGAACCCGCGCAGAAGGGTCCGTTTGGTTAAACAAGCGTATGCTAGCAAATGAAGTACTAATTCACCTTAAAATGCGCCAAACCAGCTCAAGTCTTTTGCTTTGATTCCATGATAACGACGCATGATGATGTATTATTTATCTTTCAACATGTGGTGGTATCACATGAGCATATAATTGTGAGATTGCCTTTTGTCGGACAATTATTTTATGAGATCCCTTATTTTATCCCTTTATTTAGTATTAACGCACTTTCTCTGCCAAGCGCATTTTAGCAAGTCCTCTTTATTGAATGTTAAGTTGCGTCTTTTAGTTTATAATGGAAACTTCCCGGAAACCACTAGGTATCTTCCAAGATTGCCAAATCTGATATTAGCTCGTCTCGATTTTTGCTTTGAAT

>TCONS_00071289

ATGGAAGTTCAAGGCAAACCTCAAGCTGAGGGTGAATGGTCCACCGGTTTCTGTGATTCCTGCTCGGATTGCTCCATCTGTAAACAATTCTTTCCAATTTTCAATAATTACGAAATTTTGTAAATAATCGCATACTTAAAATTAAGCACCTATATATATATACACGATCCATATATAGTATCTCATATATATTTATAAGTTATTATACTGTATTGATATTTTATCATAATTTTATATTACAAATCTATCATTTTGAGCCCAAAATTGATTATCAAAAATAATAATGGAATTTAGGATTAATTGTTGCAGGTTGTTTGACATGTTGGTGTCCTTGCGTTACCGTTGGCCGAATCGTAGAGATTGTAGACAAAGGTTCCACTTCATGCTGTGCGGCTGGTGCGTTGTACATGTTACTAACGTGGTTCACGGGATGTGGATTCATCTACTCGTGCCATTACCGAACTAAAATGAGAAAACAATACAACCTTAAGGGTAGCGAATGTGGAGATTGTTTCAAACATTTCTGCTGTGAGCGTTGTGCCTTAACTCAAGCCTATCGCGAGCTCACCAACCGTGGTTTCGATGTAGCCCTTGGATGGCAAGGAAACGCTGGACGTCAAAATGCCGGCGTTGCAATGGGTGCCCCGGTTGTGCAGGGTGGCATGATGAGATGAATTATATTTGCGTAACTATTATATTTTGTTTTCCTCTCCTCTGAACTTTTTTTCTTTATATCGTTGCCTTGAAAATAAATGGGGTTATCCGGTTTGTGTTTTTTTTCTTCGTCTCTTGTGTTTGATATGGATTTTATGAAAGTTTATATAGTATGTGTGCGTAACTGTCATTTAATAAATAAATGTGTATATAATTGTGGACATGTAAAGTATTTTGTAAAAGTTTCGTTTAATGATTCCTCATGCATTTTTGTA

>TCONS_00050559

GAGCTCAGTGTCTCTAGCAGGAGACTGTGATCCAGGAAGGCCGAGTGTGAGCTCAGTGGCCTTGAGGCTGATAGTGGCTTTGTCATCATCTGCAAGGCCGGAGAGATTTGAGCTTCCAACAGAGGAACAATCAGAGAGACCCAAGTAGTTATGCTCCTTGAGTACTCCTACAATGTTCCTGGTAGAACTTGCCACAGAGCCATTGCTCTGCAGCTCCTCTTCCGGTGACATTTTGAAAACTTAT

>TCONS_00074152

CTTGTGTAGGAGATTTTGACCTCAAACTCATTATTATAGTACAGGGCTAAGCCCCCACTTCTGCCAATGGGATCCACCGTAACCAAGTTATCATAGCCAAAATGTGCTTGAAAGTCCTGCATAAACTCCTGGTCTTGCTTTGTTTCGGACAAAAATAAGAAATCCGGTTTATGTTTGTGCCAAATCTCCCTGAGATAGCTAATAGTCCATTTACTACCAGCTCCTCGACAATTCCAACTTAATATTCTCATATAATTAATTGGTAAATACTCCATAGAGCTAAATAAAAGTAGGATTGAAGAGTCCCAGCTCCGTAATATGCCATGGCATTCCCATTCCAAAAACCATATATCCCTCCACCGTAATGATTCCCCTTCCCCCCTCCAGGATGAATGTCAAGTAAAGATAATATAACCTGTCCAGCAGCATTGCAAGGTATTCCATAGCCATATCTATTGTAACGCATAAACCAGTGCCCAATCACACACCTACGTAAAATCAGGCCCATATCATAGTGACAATACCTTATCACCATAGGCAAATCCAACCACCACAATCCGGTAATCCAGTTCCACATTAGTATCCAGAAACACGACTGGTTTAAGGAGACCAGTTTAGTGATCAGATGATCCTGTGTCCCTTCCATGTGCGTGAACCAAATTATTCTCCATAGGCACACACATAATACTTCTCCCATCATAATAATAACCATTAACCAACATAGAGGAGAAACAAACACCCCCACCAGTATCCAAAGATACTCTTTTCCTTTCCACCCTTCTCTTTTCCACAATTGTTCAAAAGTGATAATGTTATATAGAAGACCGTTTACTCTCCATCTAAGAATCAAGAGATCAATACCGAACCTAAAAAAACTAAAATAGCAATGGTTTAAGCTTCTATTATCCGAAATCCTAGTTACCCCAAAGGTATCCAACAATTGAGATCGCACAAATTCACCTAGCAACCAGATATAAGAGATCACAGCACATAAACTTAAGAAACACCACTTCTCTATTATTATTCCATGCGTAGATAAACTCCCACCCCAAAGCAACAAAATCCAAACAAAATTATTAAAGAAAACCCCAGAATCCCAAATTATAGAAGACATTGCATGTATCATCATAAAATCAGTTCTTCATTAACTGTTTCGGGTGTAAGGAACCCTTTTCCTCTGGCTGCTTAATTCCAGCCCCCTTCCTATTACCATGTTTGAGCACTGCACGCTTGCGAGGAGAGAGGAGGACCTGACCAAACTTCTTGGTATTGATACCAATGTTGGGCAACCCTGCGGGTTTGCGCGTGCCGGTTTTCGTCTCCACCTGCACCTCCTCTGTTCCTCTCTCATTGCTCTGATGACCATTGTCTCCTATCTGAATAGTCCTGTCCTCCGTGATAGTATCCTTGGTCTCACTTTCTGCTATGGTTAACGGTTGCTCCATAGATACCATCAAAGCATCAAGACCATCTTCCTGGAATTCCTCCTCTCCTAATTTATCCTCCTCCCCTGCCCACTCTAAGCCCTCGTTGGAACCGAACTGGTCCAATGTATGAAGCCCATCTGCT

>TCONS_00033643

CAGCAAACATGGAAAACTTGCGGTAATTGTCAAGAAAAGCTTGCTTCTTCCTCAATTTATCATATTGTCTTAAACATTTGCTGAAAAGGTGTCTGATACTTGTGTCGCTTGCTAACATTAGTCCACTGACCTGAAATATCAGATACACTTAACAAAGTATAAGATATATACAGAACAAAGTAAGGTTACCCTATGGGCTGTTTGAACATATGGAGTGTATCATGTCATGAGGAAGTGACACCTTGGCGTTGGGATCAAATAAGCTAGCAAGCCAACCAAGTCATTGTTCATGTACCCTGGATAACGCAATGTCGTTGTGCTAGCAGACATCACAGTCGACGACACTAGCGAATTCGTTTGAGCAAAGGTGGGATTCGTGAGATGCAGACGCTCCACCTTTGTATGGCCTTTTTGAGAAACTTCTATTCCAACATCTATCATTAACAGGTACTCATTCCACCTTTGTATGGCCTTTTAATAGATTCTAATACAAATGGTAGAAATATACAATTAAAATATTTATTCATTTTTCTATATATTTCACTAGCTGATTAATAAAATAAATAAGTTTGGCTCATAATAATCCGTAACATAACAGAATGAAATGAACTAATAAGCTCTTTATCTCAGGTTAAATGTTGACCTTCTGATACTGTATTAGGTTCTTGAAATGCCTCATGTAAAAACTGGTTTACTTGATGAGTTTGACATTATTATGCAAACTAAAAGTTCATTAATAGATATGCAATATGGATAGTAGGCTAAAGTTAAAGTTGATGTATGTTCTTGGTTCTCGAATGACCTTTTGTGTTCTGAAGTAAGATTCCATAACAGAAGGCACAATAGAAGTTTCTCGAAATTGTGGACATTAGGCACCTGTTAATGATAGCTGTTGTTGGTTCTAGAGTGATGTGCCGCGGTCTTCTTGATAACATTCCTAGAGAATGAAGAGTTTTGAAAGAACATTACACCAATTGTCAGTTTTGATTGTTTTGTGAGATAAGATGACAAATGTTCTTAGTCTTGTGTCTTTGTTGATGTCCTAAGGTTCTAACTTGATCATAAGGGACTTGTTCGAAGGACATCCCACGTATTATGTATATGTTGGAATAGTAAACGTCCCATGGATATCACATAGATTATTGTGTAATATTTTGTCCAAGTACCACATTGGTCTTAAAAACATAGGAAGAATCATGTTCGTCCACTTGAAAACTAGGAGTCTAGGACTAAAACTAAACGTTCTACACGATAAGAATTACATCCACGACAAATCGGGAGTTGAACCGAGAAGGGTTTCCAAAGAAGAGAAACACGAATCATCCGTTGGCGAAACATGAA

>TCONS_00025540

TAGAAATAGAGATATTGAAATAGCTTATTAAAGCTTTATTGAACAAGAAGTAAAGAAAGGAAAAGGCTTTAGCTTTGTCACCAAGAACACAAGAACTTGAACTGATATTTCATTAAGGATAAGAGGGGCTGCCTTTCACGGCAGCAAAATTACCTTATTCATTACATAACAACTCTATTTATACTACTACTAACTCACTTGCAAGAAACCCTATCACACATGGGTTTACATCATAGTATTGGGCTTGTTAGTATTATCTTGGGCTTGGTCTTGAAATGCTGTCTTGTGTTAATACTCCCCCGCAAACTTGGCAGCGGGTGAACACAAAGCTGACAAGTTTGAATCCACATGAATTTGGCTTTGGAGGAGGCATCAGTCGAAGATGATCATACAGTAGACTTCTGAACCAGATGACAACTTTTGGTGTATACTTGGAGATCTATCATGACTCCCTCAGAGGTATCTTTGACTTGGAGAGTTTAGTATAGTCTCTCAGAGGTATTCTTTCACCATTGTTCATCACACAATTCTTGGTTCCTCTGTATCCATCACCAAGCAAATGGGAATGTCCCCACAAGCTATCCTTGCTAACTCCTATCCATTAGCCAACTATAAACTACCGCTGAACCATTCTAATTTTCTTTGTGGCATTTTATCAAGCACCTTGTGTGCCTTCTTGAAAACCAGGAACTTGCAGCCACAATTTAATTGTTATCAACCCAAAACAAGCATACTTAGTTCAGCGTTCCTTTTCCCAACCTATCTTAACCTCAGAATCTAAACTAGCCAGCAACTCAACCCAAC

>TCONS_00067204

ATTAAATGTGGCAAAAGTTTGTTACAGACAATACTTTAGTAGAAGCATAAACCCCATGCTAGGAATCAGTTTGAACGATTGTGAGAAGCCCGAGTTTCATGATCCAAAAGGCCCACTGAAGAGTAGTAAGGATTCCTATGAAGCCAAGCCCATTTAACTGAAATATCAACTCAGCCTGAGATATGGCAGTCGTAGAATTTTGAGAAGACGAAGGTTGATTTGTCTTTTTAAAAAGGAGAAGGTGGCACTGATCAGTTTGTAGACAGCCATCTCTGCATGGTTTGGCTAGAGAAAACTCCATTGTCATCTCGGAAGGATGATATCCTGTTTTTGACAGTAGCGTTTATTGAAGATACAAGAGAGGAAGGAGACCTGAAAATATTCCGATGAAGTCTGTTGTTTCTCTCTTTCCATATTTCATAAATTGAAGCTTGCCAAGCCAAAATAATGAGATAACGGAGGGTAGAGTCTCCTGTGAAGTCGATTAGAGCCTGAAGCGAATCATTCCAGCTATTGGATTGAAGGCTGAAGTTAAGCCTTGTTGCCAAGGAGCCCCAAATAGCGTAAGAGTACTCACAATCGAAGAATAAGTGGTCTCTGGATTCTTGGTATCTATTACAGAGGAGGCAACCTGCGTCAGTTTGGAGACCCCAAGCTAACAGTCTGTTCCTTGTTGGACAACGATCCAAGACAAATAACCAAGTAAGGGTTTTGAATTTCGGAATCCCTCTTTTGAGCCATACAACATTCTTCCAAGCTACAGATGGATTGGATTCCCTGATAAGGTTGTATACTTGATTTGCAGAGAAACACCTGCTTTGATTCGATCTTGTTGTCGAAGTATTCCAAATCATAGTATCTGGGCGATCTGATAAGGAGATTGATGTGAGATAGGTGAGCACTTCTTCCATCCTGTCAGATCTTGCTGGAGCAAGAGTCCACGAGTCATTGCTCCACAAGGACGCTAGAGAAGATGAAATTGGAATACCAGTAGAGCGAGGACCTGATTGTCCCACAAAGTTGATCAGTTGGCCAAATGGTGACCAAGGAGAAGTCCAAAATCTGCATTCCCTACCATTACCAGGTAAAATTTTCAACCAACG

>TCONS_00024414

ATTTTAGAGAGTTGAACAGGTATTGAGAGATTTAGAGAAAGATTAAAGTGAGATGAGATGATTTATGAAATGTAGAAGAGAAACATGATGAACTAGAGAGAGAATATGATGAACTCGTGTTAATCATTAATCAAGACAGAGTTTACAATATATAGAGAGAGACATTAGGGTATTCAGAAGCACTAAGCATGTGAGGTCAAGGATAAGGTTGCTTTAATTTGAATGTAAACCAATGGGGATGATCACACGCTTAAGGCATCTTTGGGTGAGACTCCTTTTGCCTTTACAGCCTGTGCCAGACTGTCAGGATAAGCCGCGGTCACTCTATCCATCCAAACGGTCATATCTTCTAAGGTAAAACCCCTTTGGCCGTTTACCTCTCTTACCCGGGTAACTTCTCTTCTTGGCCGAGTTATGGATCGACCCGGACAGTACGGACGGTACGGCCAGTACGGCCTCTTGTACGGCCTGGTCGATCCCTAACCTTCCTTCCCTTTGCCTCCACATTCTCACATCCTTAACTCCCTAAGCCATCTTCTCACATATTGATCAACTCATTTCAACACTCCCCCTCAAGCTTAACTTAATAACTTAGTTAAGCTTGGAACCACCATGAGATCTTCCTCATTAAAAACCTCACCAAGGAAAACCCATTGGGACAAAACCTTGATGAGGGAAAAAGAGTAAAGACCTCATGGCAAAG

>TCONS_00060804

ATTTACTTACTTTTAAATCAAAAAGTCAATTTTGCAATTCATAAAAAACTATAAAATATATTAAATTATCAGATTAAAGTATTGATTTATTAAAAATTAAATTAATCAAAATCGTTTAAATTTTCAGAATAATAAAACTAACTCAGTTATATTAAATTAATTAGTACAATAATGTATGTATTTAATTTTAAACGTTTACAAAATCAAATACCCGTGCAGACGCACGGATCAAGATCTAGTCATATGTTAACTGATAAACAGTACATACTTTGCCATGTATAATATACATTTTACATTCGATACAAGTTCGTCATCAACAAAACTAATAAGCCTGCAATTTAATATCGGTAGGCAAGTTCATCGTCGACAATGTTACCAAAAATCATCGTTTTCTTAACAGTCAAAGGTAAAAGACTCTTGACATACATGATACACCAGTTTATCTACTCTATTACCTCCTCTCTTTCTCAACTGATCACCTTCCCAAAATTGTTTGTTCCTGAGTCAGTTGCTCTCTCAGTGCCTTCTCATTTTATATATATTACTTCTAATTTCGAATCTATATTAGTCTGTGTCTATATGTTTAATTATCCAATCAACTATACAGAATATCTTATAACATTAAATCATCATACTACAAGGAATCATTATACCATGTCTGTCCCAGTTTCGTCTTCTGGCTTATGTGAAGGAGACAATTTTTTTTTACATCGGTTTATGGATTTGGTATACTCTAGATCAAACCGGTTCTCCTAATTAACAATAAACCACGGTTAAATACAAAACAAAATACAAAAAAACAGGATTTGAATGTAAATATCTTTGAGTAATAGTTTCCAGCAATATTTTCCAACTTAAATTGTAGATAAAACCCAAATTCCTAATTTGTAGCAAACTAAAACCAAGATAATATCAAATTTTAAATCAATCAAGATAAGAACATTAATTTTCTAATATATCTTCTAAATCTTAATGGAATAATATACGTCAAATTTATATGGTTAGATACAAAATTATACCAAATATGCTTCAACCGCTTCAACAAGATTATGTAATATCTTAAACCCTTCCAAATACAACCCTAGAACGTCTCAAGCCTACACGCTATATATACGCATAGTTACAGCACCATTGTATTATTCAAAACCTTTACTCTCCATTGTTTACGTGATGTCTCCAAAAGGTACAAACTTAGTTAGTGATATCAAACCCTGGAAAATAGAATGGCGAATTCATGCAAAAGCATTTCATTAGTGGAGGCAACAATCAAAGTTTGGAGGGGAATCCTTAGAAATGATACTTGCAGATGAAACTGTGAGGATCCATCCAATCATTTACGAATAGAAAGGATTGAAAATTCTTATTGTGGACAAAGATAGGAAGCCTCAGAGGAAGACTAAGAATGTAGGTTTCAAAGAAGTGTTTAACAACCTTGAAAACGGAGAGAAGAATGAATTCTGTGTTCTCTCTCTTTTTTACAGTTTTGAACAATGATGTGAATTTGTTTCTGAAGTTGTGAGCTCTTTTGAACCTTTTTAGTTCAGTATAATCGTCATGTTTCATTTAAAAGTTTTT

>TCONS_00038343

CCAGGATGAATCGCGATATAAGAAGCTTGATTTTGATACATAAAATAGTGGGAAATCACCAGGAAGTCGAATAAATCTCATAGGAGTTAGGATGAAGAAGCTATCCCACTTTCAAATCAGGTGATCCCAATTTTCCTGTTTGGGAATATGACAGCTTCTTTGTCATTCTAATCAAACAAGGATGAATCGCGATGTAAGAAGCTTCATTTTGAAACATAAAGTAGTGGAGAATCACCAGGAAGCTGAATAAATCTCATAGGAGTTAGGACGAAGAAGCTATCCCACTTTCAAATCACGTGATCCTAGTTTTCCTGTTTGGGAATATAACAGCTTCCTTGTCATTCTAATAGAACCAGGATGAATCGCGATATAAGAAGCTTGATTTTGATACATAAAATA

>TCONS_00009713

AATTGGTAGGCACCACATCCTTTTGTACGGTACCCTATTACGTCCTCGTCCTTGCGGCTTGAATTGTGGCTTCTTGTAGAATCGACATTCTTCTAACTTCTCATCATCTCCCCAGTAGATCATGCAATTGTCGGTGCAAACGTCTATCATCTCTGAAGGCAACCCAAGACTGTAAACCTGTTTTTGAATCTCATAATAAGAATCAGCAGACACATTGTCTTCCGGCAAATACTCTTTAAACAAGTCTGCCCATTCATTCATGCAACTTTCAGGTAGATTGTGATCAGTTTTAATATTCATCATTCTAGCAGCCAACGACAATTTAGAGAGATCTTCTCTACAACCACTGTAAAGTGGCTGATTCGCAGCATCTAACATTTCATAAAA

>TCONS_00004283

CTTTCTCAAGCAAACTGCTCGATTTGATTTCTCGAGATTTGATTTTCTTCTTGGATCGTTTACCACTGATCTGGATCCGGAGCTGTGTTAAATGGCGTACGTGGATCATGCGTTCTCGATATCGGACGAGGACATGATGATTGGGACTGCGTACACCGTGAATAGCCGGCCGCCGGTTAAGGAGATCGCACTCGCGGTGGCGCTACTCGTGTTCGGAGCCTTAGGAATCGTCTCAGGCTTCTTCATGGCGTACAATCTAGTTGGGGGTGATCGAGGCCACGGGATTTTCTTCATGGTCCTGGGGTGTCTGCTGTTCATCCCAGGGTTTTACTATACTCGGATCGCTTATTACGCTTACAAAGGATACCAAGGTTTCTCCTTCTCAAACATACCCCCCGTTTAGTCTAATGTATAGAGAATGATGTTTAAGCAATGGCCTGACTTGTTTTCGTTCTAGTGTAATAGTTTTAGAGGAGTTAAGTGATTCCATGTTTGATATACATATGTATCCCTTCCTGCTTTCCTCCTTACTCTTGTTAAATAATTGCGAGTCTG

>TCONS_00009249

GTTAAAATGATAGATGTCGAATAGAATGCCGAACAACAAAGACTTTTAATAAAAAAAGCCTTTGAGAGGCATTGAAACACACGCGCTTCTCTCCTCGTTCTCTCAAAAACCCAAACACCGATTGTAAAGGGTGGAGACACTACGGTGCTCCGGCGGCGACGTCGGTGTGCGTAGTGGCTATCCCTCTCCAGAAACTGCTCGACGAGACGGGATAGGCAAGATCCGGTGCTTCTAGGAGGTGTCCCTCTCTCTTCGCTGTGACTCGGGAGGGTTTTCTGAAGCGGTGGCCCCTAGAAATCCCGCTCTGGTGACTCGTCGGTGTGAGGTTGGTGTGCGCCGTTAGAGGACGACGGTGACCCAGCCGGAACCCGCTTGTCAGTCATTGGGGCTGGGGTTAAGCTCCGATTGAAAGGATTGTTCGTCGGTGTCGGTGGATCTCACATCCGGGATGGCGGTTCCCTTATGTCTTGAATCTCGCCGACGCGTCTTCTTCCTTTCTCCGCCGCAAAGAGTGGAGGTACACACAATCTCTCGGACATGTGAGGCGGTGAAGCTGTTGCGGCTAACGGCGGTTAGGGTTTTGGAGTTGGCTCATCTTGAAGTCGACGCTTTGTGGCCGCGGGACACTCCGTGGGTGGCTCCGTTGATGGTAACTGGTGATGGATCTGGCGGGCCTCCGACACCCCTGCCGTGACAAGTCTCTGGAAACCGGATCTGGTGGCTGCGGTGTCTAACGGAGGAGGGAGCTTGCTTCCTCAGTCTTCTTCCCCACAACCTTATATCTTCTCTGCTTAAACCTGTAATTTTTAGCCTAGCTTAAATCACCTGTAACCTAGGATTGAAGTTTCTCCGGTGTACCGGCTGTATTGTTCTGTCCCG

>TCONS_00010027

TTTCGACAAAAAAGCAAAATAATTTATCTCTCACCTGTCCGTTCCTTCTTCCCAAAGAGAAGAAGATGGCGATCTCCAACAGGATCCTGATCATCCGATCTTTAATGGTAAATCCTCAGTTACATTAGTTTCTCTTATTAATCTGTCATATATATCTTGTCCCCTTTGAAAGTGTTTTACGAAGAACAAACATAAATCAGATCTAACCTTTTTTAATCTCTTTTGCATGTTGGATCTGATGACTTTCTTGGTTATTCCGATGAGCTGGAAACATGTTACAATGCC

>TCONS_00081075

GAGCTTCGTTTTGATCGTGTCCAGCGGGAGGAGGCAGACGTAGGTGAAGGCGCCGGCGATTCCGCCGCCTCCCGCGCCGATTAAGGCGCGCTCGAAGACGGAGAGGTGTTTCATCAGCGTTTGGATCTTTGGGCTGCTTCGCGACGCAGGCTTCATCCATTTCAGGAATCCGGAGCCGGGATTGGAATCTGAGACTGAAGCCGATGCGAACGGAGGAGATGGTTTTCTCGATTTGGTTAGAGATTTTTTTCTGACGGCGGGGAATTGAGCGGAGGTGCGATCGGAGAAGTAAGTGAAGAGGGAGTTGAAATCGGTGGTGAGGTGGCAGTGGTTGAGATTCGGAGAGGGGAGGCCGAGGCTCTCCGAGAGTCTAGCTTCCATGGCTGAGTTGGTGGAAGCTGAGGACGAAGACGAAGCATCATAAGATGATACTCGAGAGCCCT

>TCONS_00045822

ATGGTGGTGAAGATTCTGCCGGCAGTCGAAAGCTCCTGGAAAGATGTCTCCGGCGGAGGCTGCGGTTTCGCAATGGCTACCTCAGGTTCGTCCTAACTCTTATCTAGGTTTCATAGAACGATTCATCGATTTACAATCGATAGTTCAATCGGAATCATAGTGAGATGGTTTTGTATTTGATTACTCGAGTGTTAATCAGAATCCGGGAAGCTGATCACATGCGGCTCGACGGATGATCTAGGTCAGATCTATGTCACATCAGGGAAGCACTGTGTAAGCTTTTCACTAACCTTTCTTCGTATGATTGTGTTCAGAACACAGTGTTCTAATTTGATATTGTTCTATGATTATGAGTTTTAGTCGAGTTACTCTTTCATAGGAGACTCCCGAGCCGTTTCCTCTGCCTCCGGAGGTTTGTGTACAGAAAGCTGAAGCTGGATGGGATCATTGTGTGGCGGTAACAGGTGACTTTCTTGATGTTTAGTTAGCTTTGACCACATTTGCGTAAAAGTTTGTTAATTTGATTTGTTTGGTTTGGATATAGAGAGCCATGAAGTTTATACATGGGGATGGAAGGAATGTATACCCACTGGAAGAGTGTTTGGACAAGTGGAAGGAGATTCGTGTGAAATGAACACCTCCTTCTGTGCAGAGCAAGGTTAATCTCTTTTGCATGTTAAAATATCTCATGTAATGACTGGTCTTATATCTCATGTTCCAAGTGCTTCCAGAAGTTTCAACGCAGGTTCTCATCTTCCACCAGTGCATCATGTAATGATGTAACACTGTTGGGATTGTAAGGTAGTCCATGTTGAACACATTTCCATTCACTCTCTCTAAGTAATTTAAAGCCAATCTCATTCCCATACGTTATAACAAGTTATTGGGTGTGTGGGTATTAATGGTGAGATGAACAAACTGACAAAGCCTTTCTACTGCTGCAGTTTCTCAAGCGATGGTTGCAGATACTAATTCCCTTTGTACTTAAACCTATTTGACATATAAGACGCAGGGATGGCAAACCAAATAACCTTAACCTTTCATGCCAAGCTCTCTAAGTTCAGACTTTTCATCAAACCATAAATCATTCACCCTAGCCCTGATATTAGATCAGAACCAACGCCAATTCCTACTTTTGATGGACAGGTAAGCGTACTCACTTACAACTGACGTGCTCTTCCTCTTAACATGTCAAACTGATCTTCACACAAACTCATTCGCAGGTAGGTAATGACAACTCGGGCAATGATATGCCAGGTTCTGGAGCTGTTAAAAAACTTACACCACTGATGGTGTAAAGACATAAAGTGTGAACAGTTGTGTCGCTCTGAACATCGTTGGCATCGGTACTATTGCTATTATTACATAGTATAAGTGAGTGAAAACCATGTAATGGTTAATCTTCAAGAGTATAGGAGTTTTAAACACTGTTGTGGACTTTAGTTGATGCTCTACTACCACTTTTTAAGAGAATCCTCTAGACAAAAGTATTTTACCTCTTTCGGTCATGCTAATTATTGCTTCTGATTCTTTTTCCTTCTCGATTTTTTGTACACAGCAAGGCAACTACGTTTGTACGGCAACTACGTTTGCTCTTTTTTAGAAATGGCCATGGGATCTCTGATTTTCTACACATATATATCTGATACTTCAGTGCTTATAAGTGCTTTTATTTTTTTTTTAAACTTTTGTACGTTGCCAGGCAAACTACGTGCTTTTCTTAAAAGTGTTAATGTGATTTCAGTTTTTCTACTCTCTTATATATATATCGGTTCCTATAAGTGCTACTGGATATTTTAATAATTTTCGTACACTGTAAGGCAACTACATTTGCTCTTATAAGTGCAAATTAAACTTCATCAGAAATACCATTAGTCATTGAGTGGTTACGTAAGAATAGACCCAAGCTTCATAACACTGTCAAAATTTTGTAACAATTAAATTAAAAACATTTTCCTGGATCAAGACCCATCCATCTCTTTCTCTAGCTATGGTGAAAAAAAACTTGCTGAAATAGGTGAATATGTAAGTGATTAATCAAAAAAGAAACACATCAACATAGAAGAAACACATCAAAATAGAAACAACAAATCTTTTGATACAAAACATAGAAGAAACACATCAAAATAGAAACAACAAATCTTTTGATACAAAACATAGAAGAAACACATCAAAATAGAAACAACAAATCTTTTGATACAAAACATAGAAGAAACACATCAAAATAGAAACAACAAATCTTTTGATACAAAACATAGAAGAAACACATCAAAATAGAAACAACAAATCTTTTGATACAAAACATAGAAGAAACACATCAAAATAGAAACAACAAATCTTTTGATACAAAACATAGAAGAAACACATCAAAATAGAAACAACAAATCTTTTGATACAAAACATAGAAGAAACACATCAAAATAGAAACAACAAATCTTTTGATACAAAACATAGAAGAAACACATCAAAATAGAAACAACAAATCTTTTGATACAAAACATAGAAGAAACACATCAAAATAGAAACAACAAATCTTTTGATACAAAACATAGAAGAAACACATCAAAATAGAAACAACAAATCTTTTGATACAAAACATAGAAGAAACACATCAAAATAGAAACAAATGTATTTTTTTGGAAAAGCTATAGAAATAGAAGCAGTTGTTAAACATTAAATTTAACCAAGATAAAATTTCCTTGCGTAGGTCAACCCCTAGGATAAACAAAAAAAATTGTGAAATATGACAACGACAATAATAGTAGAAATCAAACTAAATACTCAAAACAAAACATATCAAAATATTTACACCGACAAAAAAAAACATCCCATTAAATTTTAACATTATATTATGAAATTTAATTAACCGTAACGAAAAATCAAACATCAAACAATTTTAACCATAACAGTCGATAAACATAATAAGAAAAATAAAAGAAACAACATTTAAAATGATATACAGTAAAAAATTAATAATAAACATAAATTTAAAAAAAATATTAATGTTTCATAGTGTTTAGTAATTTAAAAATCTATGTGTCTTATTGATATCATTAACCTAAAATAAAAATTAAGAGAAATTAAATAATTCTGTTTTTAATAAATATTATAATTAGACAATCTTATAATTGCCAATATGAATTTTGAAATACATGGATAAGTTTTTTGAAAAAAAAAGATTATGAATCCTATTGGGATTTTTGGTTAAAAGTAAATATATATTATTATTTTATTATTCGTCTGCCCGTAGGGCGGGTTTACCCTAGTCAACACAATAATACCACAGAGTTCTCTCCAGAACATGACACATCAGATTTTAAAATGCCTGAAGAACTGACATGTCATTAACTGAAAAAACTTGAAGAACCAATCAACTTATTTCGTTTCTACACGTCATTTGTCTATGGTTATATTTATTTTTTTTGTTTTCTGAGCAAAATACTTTACTTTCTTATCCGTTAACCATCGTCGATGTTTTAGGTTAGTTCAAGAGAAAGAGAAGTGATAGAGAGTTAAAACCAAGTCCTGGTCTGAGATTCTTCTTCTATTTCATCTGTGTATCGCGAAGCTCTAGCCCATATTCTTCTTCGTCTGTCTCTCAAAAAGGTTTTCATTACGGTCTGCGACAAAGCCGAGTCTCAGATGTGATCTCGCGGCGTCTATGGATGGTGTTGATTTGATTAGGGTTCCATGGCGTTATGTCTTAATATGCTTTCAAACGAGTGCTATGTGGGCTTATATCAATCAATAGATAGATTTTAGTTGAAAGTATTTTGATAAACTGATTATGTTGTTTCTTCTTTTTCATTTGTCTACGGTTATGGTTATATTTTTAAGAAAGTTTGGTCCTTGATTTTGATTTTAGGATATAAGTGTATTGCTGGAAGTTGCTTTCATCATTGATAATCGGGATCGAGTATACCGTAGAGTGAAGATTGATACATTCATCAGTATGACTGTTTTTAGCTATCAGTACTTTACTTGGTGGAAGTATAGCGAAGGAGTTGCAGAATTATAGGTGTTGCGAACTGCGAAGTACTTGCTGGAAGTATATTGCTGGTGCCCTGTACAAGACAGTAATTACAATGGCTCATACATTGATCCTACACTACAACCTGAGAGCATGTGTGTGAGAGTTAGCTCTTTCATCTTTTGCCTGACATCTCTATGTAATTCATAAGCCTCTTTGGTCACTTTCCTATCCTCAGTAACCTGATAACTCATATTACAAATTGTATTAGTAATTAATATGTACCATTTGAATGTATGTATTACAAGTTAGCTAATTGCTGGATTTTCTGAGTTATTCATCTTCATACCGATTGTTAGATAGATTACTGCACTCTTGTTATTAATAGTTGATATCTGCAGTTTATTTTAGTGTTGTTGTGACCATAGGTTTGCAGGAAGCAGCGGATGACAACAGTGTTTACAGAAACGAGGTTCACCATTCGCTGCACCAAGGCCGTGCGCTGCACCAAGTGTCAACACAGCGAAGCTGTCTTCTTCCAGGTGAATTATATTATAAGCTTAATTTGTTATCAGTCAACAAGTAAACAACATTATTTAATTGTTTAGATCATTTAGCTTCTCCCAAAAAGACCATTGCATTTATAACATAAAAAACTGGGGAATACGACAGTAAGCTGCGCTTTGCAGAAAGAATACAAGTTTACTAATGATCTCTCGAAAAACCAGACTGAGAGACTAAGCAACAAACTGATGATTAAGAATTTTTTTATGGTGCTTAACAAGCTCGAAACCAAGTTATGAATATATCTTGGGCCCTTTTATATATCACTGAACTCCCAAGTTAAGAATCTTATGTGTGTGTTTTATTCCTGATTTGAAACATGTGTAGGTCCTATTACAATCGCTGATCAAATGGCTGAAGAGGCCATGGTATCCATTATCTTCCACAACTCCCTACTCATGGTATTTGAGTTTCCATCGAATAAAGACACATTGATTGCTTCAGAAACGCGGAGGTTCTTTATCTGCAAGCACCGGTAGGAACATTAGCTATCCATTGTCTCCCATGAACGGTGGATACATGCGATATGTAATGTAATAACAGGTCTGGATAATAAACTAACTCGAGATCATATACCTGATTATGTTTTGGTGATTGATTTTTGTAATATAGGAAACAAACGGATGGGAAGGTGATCTGCAAGTGTATTACACATTAACTATGGAGCTGGTGATGTCTCTTCTGACCCTGACGTAACAAAGCAGTCATTATATGCTTTAGCTCTTCTGGAAGAGCAGCCAGGTTCATAGCATTGTTTGCTTAATTATTAATAGTTTGTTCTATTTATAGCTGTCTAATGAATTGTTATGTTATTGATCTCCATACAGGTTGATTGCCATAAACAGTCAACAATGCTCTTTTGTCATACCCATGGTCAAGAAAATTCATTTGAAATGGAGCTTTAGTGGAGATCAATAAGGTGGGGAAAACTGGAGTAAGAGGTGATAGAATAATGAAGGAAACAAAGAAAGAAGAAAACACTTGGGAGCCATTTTGGGACGAGGAGTTTGAGTGTCAGCTTACAGTACCAGAGCTGGGGTTGCTCAGAGTTGAAGTTCACAACTACAACATGCTAGCGAAGGACAATTTCTCTGGCAGACATGTTTGTCTGTGGCGAGCTTAGACAGGGTCTACGTTCTGTTCCTTTGTATGATCGCAAAGGTGAGAGACTTGTGGCGGTTACACTTCTCATGCGGTCTCAGTTTGTTTATAAATCAAAGTTTACTGTCATACTTCAAAAGAGTTGTCTTAAAGAAGTTTTTACAGCACAAAACAAGACAATGTCCTTACATAAAAGATATAACGAATCAAATAACTATGGTTGGATATATATTCTGAAACAAAAGTAAGCTTACAACTCTTTTGAGATATATGAATGAGCGTATTTTTTTTTGCTCACAATTTGTGATGCTAAGTTTTGCATATTTTTA

>TCONS_00027060

ATCTCTGTATGCAAAGGGATGCAATAAGACAACTGTATCACTGTAAGGAGGCTTCACTTTGGGTGGCATTCTGGGATCACATTTATCATATGGTTTTGATGTTTATGTATTTAATTGCTTATGTTATGTGTACGCTTGGTCCTTTAAATATTTTTGTGCGTTCATAGGCTTAAGCTTCCACTTCAGTAGGAAAGGCAACACAAATGTGGACCACAAATCTGAAGTTCGATGTTGGAAATGTAGCTGGAGACCAGTGGAAATCCATGTCATATGTTGTGTTTCTAATACGTATGTTTGACAATAATGAAAAGATGTAAATCAAAGACTGAAGCCATGAGTTCCAATCCTTCTCACCGATTTGAAGGCCGGCCATTGTACCTCCGCCATTGAGGTCCGCTTGCTTTGGTTCTGGGAAGAAGCTAGGTATGGGAAGCGGCGAGCTAATGGAGGTTGATCTGTTGCTCCTCGACGCTAAGTGGGTTTTTATTGGTATCTCCGTTTTCTCCACGAAACATGTCACAGAAACCCTAAGGTTGGATTCTGTTCATCTTCCCGATTCAAAAGAAAAACTAGAAACGCTTTGTGGTTATGTCCAAGCGAAGAGACGTCTTCAACATAGTCATCAGCCATTGTCGATCAATCCTTCAATGATTCCCCATTTACTGCCTTCGTCAGTATGTACCACTATAAAAAG

>TCONS_00011441

ATTGGATTCATTTGAAAAGAGCATAAGATCTGAGAAAGAAAGAAAGAAAGAAAGAAAAAACACTCAAAAGTTCATAACTATTTATAAACAGTTAAAAATGAAGTCGATCGTCGTGATCCTCCTTACGTACCTTCTCCTTGTTGCGCCTTGCTTCGCTATCGGATCGGAGAACACGAATTCGGATGTGTACGAGATCGATTACAGGGGTCCGGAGACACATAACTCTAGACCTCCACCAGAGACTTTACATGGCAAGCGACCGTTCATCCACCATAAAACCTCCGCCGCTGGATCCGCCGGCGCTCATGTTGGAGGACAGAACTAGAGAGAGATTTTTCTAAAGAAGAGGAGAGGGACTCGGAAGCTGTAAGTGAAAGTGGAAGTATATGAGTGTGCGATAAGGTTAATGATGCGGGTACATAATTATTTATACAATACATATGATCATCAATGTTATCATGTCCGGTATTTTATTCATGTACCGTTCTATATATCTTTATTATTATTTTTTTTTTTTTGGGTCTGAATCTCTTGGAAGAAAGTTCAAAATTAATTCCGGGTAATGTGTCGCCATATGAATATCAGTGTCATTCTCAGATCTTTGAATCATTGTCTGTTCGGTTGGGGCTTATCTTGGGCTAAAAATTATGGATAAATATGAGAAAACGATATGTAATATATATAAATATTTTAGTTTTTATATATATTAAATGTCTTACATGCCAGATTACAAATAATAATCTTACAATAGACGTCATCAAAATATGTATTACAATACATTATATTTATGTATATATCTTGATCAAATGGCATGGTTCCTCCAAGCATTCATTCATTGGTGCAGGATGTACAGAAAACACTTTTAATTGACGACGTCTTTAATTTGTTGTCTCTCTATTATTTCATTACAGTTGTAAATAATATGAAAGTTAAAATCCACAGCATTGTTTGTAAAAGAGTAGTATATGATATTAACATTAGAATTCAATACATGTGATCATATCGAGTAATATAAGACATTGGATCCGTGGGATCCACTTTATCAGAAGTGAGCCCACTGCGCTTAATCACGACCTCAATCATTTCGTTTCAAAAATTGGATCATGTGTACTATCAGATGGTCATAACCAAACCGAGTCTCTCATATCAGAGAAAGTTTAGGTCTATGATTATTATTTAATGTGTGATTATGTCATTTGATTGGATTTATTAGGTTAATCATGTTGGAATTTTTTTTTTTTTCCCTGATAACTGATATATTAATAATAAATATAAACATACAAGACCCAAAAGAAAAGTAGTGCAAAGCTTTAACAAAAAAACACTGAGCCCAATATTGGCCCAAACAACATCAGATTCCACGATCTTAATACGTGTAACGGCAAGACACGGAGAAGACACGTGCAAGCCACATCTCCACGATCTTCACCGAACCATCCACGCGTCGCCATCTTCCACCGCATTCACCGCCATGCGAAATCTACGCTGGTGAGTTCACCGGCCATTCACCGTAGCCACCGTCTCCCACCGGAATCATCTCGGGAACATGAACTTCCCTCATCTATTCCGACTTCAGACTTCTCCATCAAACCAAACAGAGCATTTTTTCACCGGAAACCACCGGGATGTCAACCTCAACCACCGAAACAAACCCACCTCCAACGACTGTATTGGACGTATTGGGCTTCATCGGCTACGCCACCGAGTTTTAAGGAGCATCCATCGATTAAAATCGAGATCTCATCCAAACCCGTTAACGTTTCATCATAGCAATCAACTTCAATCTCGATCGATCCACCAATCAAACTCTTACTATCCTTCCGGAAAGGATAACAAACGACGAAAGAACAGAAGACAAACACGACACCAAAGTCGGATGATCCAACCAAGAAGGTCGCAAACCACCGAAGTCAATGCTGGCTACACCGCATCGAAGAGACTACCATGTACCAGAAGCTATGTTACAGGTCAGGAAGGAAGAAGATATGAGGGAACCACCACGAGTTCTTTTTCTCTCTCAACGTTTGTAAGAGAGAGAAGAGAGAGACTCTACCATTCCCTTTAATCATGTTGGAATAGTTTTGTAACACGATTTTTTAAAGGCACAAAATAAATGTTCTAGAAGACAAAGATGGACGAAAAAAAAAGGAAGACGTTCATCATCAAGAGTTTGATATGCAGGCTGATTAATTGATGTTTCTTTCTTTTTCTTTTCTATATATTTGGAGTAAAAGATCGATGTCGTGAATATTCTGCAGTTTCAGCTTTCTTAATAATTTCTTATATATAGATGTTTTTACTGTATCAGAGTCCATGAACCAAATGAATAGTACATAATTTTAGTGTTAATTTTTCTTATAAAATAGTCTTTAAAATTTTATATAATTTATATCTAAAAATTCAGTATATATTTTTTAGAAACTAAATATATATATATATATTTAATGAAGATTTATTTGCAATAAAATTATATTCAAGCATACGAAAAAGTAAAATATATTTGATAAAGAAAAAAAGTTTAAACTTACCTTATATTTTAAAACTAAAAAATGAAACTGTTTTTTTAATGAAAATTCACATATAGTAAAACGATGGAAGTGACAATGACAAATTATTTTCGAAAAGCCCGAAAAGTCCGAAAAGCCCTATAGCCCTACAAGGACCGGACCGGGCCAGCCCGAATTGACACCCCTAGTGTTGGCCTTGTGTAAGCTGGAATATATTGTTTGGTTTTCTGACAATGTAAATTTAGAAGTCGTTATGAGGTTATTTCCACGTCAAGATATATAGTTGAGATGAAAGGAATTGACATTCGATCTAACAATGATCAACAAAGTAGTTCCAACTATTCAGCCAAACAAAGAGAGGTATTTTAATTAGAATCGTGTTTTAAACACACTTATAGTTAGAATCTATCTTGTAGTCTGTTAGGTTGTGGATCGATTCCTACATGTACAATCTTTGAGTGAGTTAAGAACATCAATTAAGTTGATGCATATCTTTAATTTGAAATTTAGCTCTTTACCGGATGCAGGGGACAATGTCCTTGTACGGGAGGGTTATGCGGATTCTCACTTGGTCTTGTTGCTCTTGGTATGCCTTTTTAGTTTTTTGCCACTGAATTATGAATTGTTTAAGCTGACTCTACTCTTTTTGGCTCAAGGCCGTGGAGGTGATGCGCTAGGTTCTTTGGACTTCTTTGTCAATCGCTTACGAGGTTAATCTTTAGACTTCCACATTAACTGTATCCCAC

>TCONS_00037975

GTGCCATCCACAAAACCATTATCAGCCGCGTGGACACGTTTGTAGTCTTTTCTCCACCGCGGTAGAATATACCTCAAAGGGATCTCTTCAACGCCATTGAAGTTGAGAACACACAGAGCATGTCTGCACAAGTATCCATAGAAGTAGAAGCAGCTACAAATGCACCGTACTTCACCCACTGACCTATTGTACAGAACCTCGAAATCTCGGACTTCTCTTCTGTTGGATTCTCCTTGGACACGTTCTTTCACAAGGAAGATCACCAAAGGCCCGTCTACGTGGACCTGCGTCGTGCTGAAACACGAGTACATCTCCTCCACCTCTACTTGGAACTTCTTGAACATCTCTCTAGTGTAGACTCTCGAGAGCTGCGTCTCGAAAGAACACTTTGTTTTCAAGGTGAGAGACTCTAACTCAGCTAGAGTCTCTTCCCTGCGCTTCCTATGAAGAGCTAACTCGTACTTATCAAGAAACTCTTTCAACGGTGTCTGCTTATGAACGTACCTCTCGAAAAAGGGACTCAAACTCTCTCCAGGACGACAAGTAGCGAGTCCGGCGAAGAACGTGTCAATGAGATAAACAGGAGCCCATCTAGCTCGATCCTCGTACAAGGAACGTAGCCATTCGTTGTCGACAATGCCGAAACTGTGAACCATAAACCCCCAAGCTGCTTCAAAGTCCACAACTTTCAACGTTTCATACACTGCTTTCGTGAAAGCCTTCCTCACGCCATCGTAGCTAACCAACCCACCTAGCTTCTCAGGTACTTTCCTCATTATATGCGCCACACTGAACCTGTGATGACATCTCGGGAACACTTGGGAGACCGCAGCCTCCAAAGGCTTGCATCTGTCTGTAACAATAGTCTGAGGAGAACACTTCATTACACCGAACCATGCTTTGAGTAACCAATGGTAAGACTCTACCGTCTCTCCAGCGAGAAGGCCGCATCCAAGCAACGTAGTTTGCCCATGGTGATTCACTCCCACTAATGTCACAAGAGGAATCTCGAATTTTCCTGATATATAAGCGTTGTCGATGAAAACGACGTCGTTGAAGAAACTGCAAGACACTCTAGAGAAAGCATCCGCCCAGAACACGTTCCTTAGCTGCCCTTCATCGTTTATATCCATCAGGTAGAAGAAGTTTGGGTTTGTCAACTGCATACGGCAAAAGTAGTTGTAGATAGCTGATGAATCTCCTCTTCTAAGGTTGAGCACATCAGAAGAAGAGCTCTGAAACTGTTTCTTGGGAGCCGAGTTTGGGATCACACTGTTTCCAACATCCATCACACGAGCCTTGTACAGCTTAAGAGATGAGTCTGCGCACTTCCTCTTGGTCCCAGTCCTTTTAACAGATTTATAACCGAGCGAATGGTTATGGTCAAGCGTGACTTCAAGCACCCTCCACCTCTTGGAATCAACCTGCCTCATTCTCACCATCGCAGGACAGCCAGTTCGCGTCTCTTTCCTCACACGGTTAGCATCGTTAACTCTCTTGAAGCCTTGGCTGCTGCAGCAAAGCACAGCGCCGTACTTCTCTTTGCTACGCCGCTTGAACCATGAGTTCTTCACTCTTACTCTAAACCCAACTTCGTTAGCGTAGCAGTTGTAGTAGTTGTAACCATCGTCGTATGACTCGAACTCCATACCAACTGCTGGAGCATCAAACTCTTTGCCCTCAACAAACTCTTCTTCTTGGTGTTCACTCTTTCTTTGTTCGTTTTGAGTGAAC

>TCONS_00068938

TCAGGTGATCCCAGTTTTTCTGTTTGGGAATATGACAGCTTCTTTGTCATTCTAATCAAACCAGGATGAATCGCGATGCAAGAAGCTTGATTTTGATACATAAAGTAATGGAGAATCACTAGGAAGTTGAAGAAATCTCATAGGAGTTAGGATGAAGAAGCTATCCCACATTCAAATCAGGTGATCCCACTTTTCCTGTTTGGGAATATGACAGCTTCCTTGTCATTCTAATAGAACCAGGATGAATCGCGATATAAGAAGCTTGATTTTGATACATAAAGTAATGAAGAATCACTAGGAAGTTGAATAAATCTCATAGGAGTTAGGACGAAGAAGCTATCCCACTTTCAAGTCAGGTGATCCCAGTTTTCCTGTTTGAGAATATGACAGCTTCTTTGTCATTCTAATCAAACCAAGATGAATCGCGATGTAAGAAGCTTGATTTGATACATAAAGTAATGGAGAATCACTAGGAAGTTGAAGAAATCTCATAGGAGTTAGGATGAAGAAGCTATCCAACTTTCAAATCAGGTGATCCCAGTTTTCATGTTTGGGAATATAACAGCTTCCTTGTCATTCTAATAGAACCAGGATGAATCGCGATATAA

>TCONS_00008784

AAATGGTTTTGATGGTGGGGTTTTTCTGCAAAGTCTTGATTCGTTGACTCTAACTTATTCTTCTCCACCTCTACCTTCAACCTCCACAAGAATAACCACAAGAGAAAGATCATAAAAACGAGTTGTAACCATAAGTACAAATGAAGGCAAAGGAAAAGTGGCTAAGAAATCCGATGTATCTCCACCTCCTGCTCAATCTCCGTCTCAGCCACAGTTGAAGCTAGATGATGTAAATCCGGAGAGATTGGAACTAAGTTGACGGCAGCTTGGCGAGGTAAGATTATCCATGTGTACGCCTCCTATAAGTTGTCCATGTGTACGCCTTGTGTAAATTGTCCATGTGGACGTCTCAGTTGTCCATGTGAACGATATTTGGTGGGAATGCCTTGCTACTAAGTAATATTCTTATGGTTTTTTTTATTATGAAAGCCATGTATAAAAAATATCAAATGTTGTATTGCCCATACATATAATCTTGAACACTTGATTGGTATTTTGACTGAGGTGAGCTTAAGAAATCAGAATCAGTTGTAAGCACTCCACTCTATAAACGATTCCCATACATCACTCACATACTTTACAAATTTTTCTCATCTGTTAACTCACATACTTTGCAAACTCCAACCTTTTTTTCATCAGAACCTCTC

>TCONS_00006535

ATAATTTACAGCATTAAAGCTTTACATAACAAAAGAAAACACTTTAACCAAAGATAACCAAAACACAGAAAAACACAAAACACAGAAAAACACAGCTCTCGTTTCTCTCCTAAACACCCAACGGAACCCTAGCTCCGGCCCGGTTAATCCGGTGCCGGAGGCGCCTCTCTTTTCCTCTCCCTCTTTTTCCTTTTGCTTTCTCTTCCCCTTTGTCCTTAGTCGATATGCTCGTTACTCTGGTGGGATCTCCACTTGATGCTCCTCTTTGACGCAGACGGTGGCAAGATCTGACGATCTGGAGCTGCGGCGAGGCTAACGAGATGACGTGGGAGGACGAAAGTCGGCTTTTAGGGTTTTCAGAGTTACTGGATCTGGTTTTTCCCCTTTTCAGATTCACGGCAACTCCGACTCGGCGACGGCGCGTGGCTACGAAATACACTTCCTTCACCTCGGATCTACGTCCATACGTGACGGTGAGACAAACGAAGGAAAAGTAGACGGCGAACAGACAGAGTTTGGTCTTCCGGGATCTCCGGGAACACACGAACAATGGCTCCCGCAGACTAATGGGCAAAGAAGAAGATACACTCTAGCGGTGGTTCGTGGCGGTGGCTTATACCCTAGCTCTGAAGGTTTCTAACTTATGGTACGAGCTCCGCTTCTCTGAAATTCGGATATGGTGTATCGACCCGCGTTTTGTGCAGCTGGAACCCGACTTTCATTCGGATACTGTGTCCGTTTTTAGGCTGAGGGTCACAGCGGTTGAAGACCAACAAGATGAGGCTATCTCGACTACGCATCTACGGTGTTGAAGCTATTGGATCATGAACTTTGATGCCGATTATTAGGGTCCTTTAGCTAGTTTTAATTGGTCTCGGGTATAGTTCTTAGTCCTGTTGTGTTAGTAGGCGTTGTTGTACCATGAGGTCGAATTAGGAGGTTGAAGAGAAGGATCAGTCCGGGTCGTGGATTGATCTCTTTCTCATTGTGGTTCAAAGTACTTTGAGATTTAAATTAGGTAGCGTTGGATCCAATCAAGTGATTCAGGGTGTTACTAGATCATCTCAAAGTACTAGTGGTTTAAATTTCTAGTGGTCAAATCTTTGTATCCACAAAATTGGTGAAATGAATGTTGATGTTGAG

>TCONS_00026799

TAGTAGCAATGATCAAACGGTAAACATGGTGGTAGAGAGAGTGTTTGATAACTTAATGGGCAAGCCTTCAACGATTTTGCATTTGTATTATTTCTTTTTTCTTAAGATATTTTTGTTAGTGCCTTTCTTCTGAGGTTGTTCATACAGACAGAGATCAATGCCTCAACAGAAAGACACTAACAAAAATATCTCTCTGACGTATGCTTTTTGTTAGTGTCTTTCTTTTGAAGTTACATTTTTATTGTTCTACTTTTACATAATCTCACTATACAGAAATTTAAATGCCTAAGAAACTCAGTTCTTTTTAAAAGCATTCCATGTATTTAGTATAAAGACTTAACCATAGGCCCGTACTAGAGCAAATACTGAATCCAAAGACTGGAGTATATTATAGATATATTAGTTTAAAAAAAATACTATTATGAATAACAAAATTTTACAACTTTTAGTATTTTTTGTTGTATATATTCATAATTTAATGTAAATATAAAGTTGAAGTGGAAAATATTTAGTTATATATTTTCAGCGTGTCGTCGTTTAAGAACACTCAACACAGATTTGCCGGGAAGAGAGCGTGAGGTTGAGTTTCAAAGGATAGAATAGTCTATTCGCATTCAACTATATATACAAGTTTCTCCTCGTGAGACCCGAAAACCGCGTAAACTAAGAAAAAGACATGCGTCGATCGGCGAGCGCCTCCATAGTTTCCGACCAACTATCAGCGAAAGCTCCGTCGCCATCACCGTCTCCGCCGCGAATCCAAAGCGACACAGACTCGGAGGATGTACAGCTTCTTCTGCCTAGGTACGACCCAAACTCTTACCCAGGGAAGAAGGACAAGACGCGACTCAGATCCGCGGAAAACGCTATCCATTTCATCCCTCTCATTCTCATCATCTGCGCCCTAATACTTTGGTTGGTTTCGAATCCAG

>TCONS_00017512

GAGTTGTTATATTCGGTTAAGATATGGAACACATAAGAATGTAATTGTTAAGCTGATAAATATGGCGTTCGAAATATAAGGTGGAGGAATTGGTGACATTAGAGTTACTTATGATGGTCAGCTTGTACGCATGACTGGTCGGTCGGTACTTTCCAGCAGCAGGAGTTACAGACACATTCTCAATGAACCTCCATTGTCCCACCTGAAGTTTCTTAACACGAGCCAAGAAGAGTCTCTTGCAGGACAGTTTATGTGTCTAACTTGAAGCGACATATGGTCATAGAGTTAGCAAAAGCCAATTTCAGAAATAAAG

>TCONS_00022947

CACAAAAACCTGCAAGATTCACATTAGTCTCAAATGTATAATGAAGACCAAAGTCAAGAGTGCCTTTTACGTATTTGATAATACGCTTTACTGCATTCATGTGAGATTCTTTTGGTTTAGCTTGATAGCGAGCACAAATCCCCACACTGAGGCATAAATCAGGACGACTTGCCGTGAGGTAAAGGAGACTCCCAATCATGGCTCGGTAGAGTTTCTCATCAACTGGTTTCCCATCGCTATCTCTGGAAAGTTTGGTTGTTGTGCTCATAGGGGTTTTTGCAATCTTACTTGTCTGCATTCCAAATCGCTTGATGAGATTTTTGGCATAGGTACTCTGTGACACTGTGATTCCATCGGTTAGTTGTTTGATCTGCAGTCCAAGGAAATAGCTCAGTTCTCCAACCATACTCATTTCAAATTCCTTTGTCATAGTCTTCACGAAGTCGTCAACCATTTGCTTAGACGTTCCTCCGAAAATGATATCATCCACGTAAACTTGAATGATCAGCATGTCATTTCCATTTTCTCCGATGAATAGGGTCTTGTCCACACCACCTCTTTGGAAGCCAGCTTGTACCAGGAACTCCGTCAGACGGTCATACCAAGCTCGTGGAGCTTGTTTCAACCCATAGAGAGCTTTCTTGAGTTTGTAGACGTGATCCGGAAAATGCGGATCCTCAAACCCTTTTGGTTGTGTTACGTAGACTTCTTCTTGTAATACACCGTTTAGGAAAGCACTCTTGACATCCATTTGATAGAGTTTGATTCTCAGATTGCACGCCATTCCAAACAGTAGTCGTATTGATTCAAGTCGTGCAACTGGAGCAAAGGTTTCATCAAAGTCTACTCCTTCGATCTGAGAGTATCCCTGTCCAACTAACCGCGATTTGTTTCGAATGATATTTCCTTCCTCATCAGTCTTGTTCTTGTGAATCCACTTGGTTCCAATAATGTTTACATTCTTTGGTCTTGGTACCAGTTCCCACACTTGAAGTCGTTCAAAC

>TCONS_00075549

CTACCATTCAATCGACAGAAACCCTAATTTTAACCCTAAAAAAAATTAGGGTTACATACAATCAAATCGAAAAACGCGGACAGAGGAGTGAGAGATACCTCGTTGACAATCGAATAGGCAGAGAGATGGGTCGAATCAGCTGTTGGTTCAAATCGGGGGAGAGATCGATGTTGACAGAGGTTCACGGCTTTGGTCTCGATTCGATATGTGAGCGGTGGATTCGGTATGTCAGACCTTGATCCGGTATGTGAGCGGCTCGATTCGGTATCTGAGAGGAGATCGCCTCGAGAGAGAGAGATAACGCCGACACTGAGAGGAGAGAAGAGAGATCGTTCGTATCACCGTCGTAGGGATACAGAGGCCTCGATAAGTAGACTTTGAGGGGTCGATTCGCCGTTGTTACAACCGGAACACGGGTTCGGTCTCAAGAGATACAGATATCGCACGGGAGAGAGACAAGGGAGAAAAGAGAAGAAAGAAGAGAGAATGGAGTTGATTTGTTTTGCTCCCTCTCACGCGTCTTTCTTTCGGTCTCGCCTAGGTGACACGTGCCTCCACAAACCACATATAAACGCTTCTTAATTAACAAACAAAATCTGCGTTTATAACCATTTTTGATTTAAATTCATCCTTTTAATTGCTAAGAAATCTCGCTATATACAGCGATGCTGTTGCTCTAAGAAACGTCTCTTAGTTTTTTAGTTAAAAACTAAAAGACTGTATTTTATATTACGC

>TCONS_00046012

CTCTGCTTCCATCAGCCAGTACTCCACCTATCATTCGTCTGCTCGTTTCTTGTTGACGGGTTAGGATTCTCAGAGTGCGATTTGGCTTCTTCTTTATATTGCACGTCTGGAGTGGACTTCACCGGTAGTGATGGCTGGACCAGGACTTGCCGACCCTGCTTCACCTGTCTTCCTCTCAGATTCGCCTTAGGGACAAGTTCACGGTCCCTCTAACTGTTCTCCGGTTGAATTCTCAAATGGCTCGTCTGATTTTGATGCAGGTCCCGTCTTCAATATATACGCATTGGTTTCCTGCAATCTAAGTGTTGTCTATGAAATTGTAATGTTAGCTAAGCTTCAATCACTTCCTTATGGTT

>TCONS_00069022

CGAGAGAGATTGTTGGACCCAAGAGAAGCAAGCATGATTAAGCTGGAAGCAATAAAAACTTAACGGAGACGTGTATGTGGGATATCACAATGTTGAAGTTGATTCTTAGATGCATCAACGTGGTTAGCTCCAACACGATCGTAGCCAGTTGATTGGTAGCCTTGAGGTGTCAATCGATACAAAGCTTCAAACATCCACTGTGAAGATTGACAGTGTTTATTTTGGCCACTATATCTCTCTAACAATCATGGTTAAGTTATTTGTCACCAATCTATGTTTATGCTTGTGTTGGTTGTTGCAAAAGATCTGTTTCTCTAACCCCATAATAATCATTTCAGATTTTGAACTGGCATGAGAGGTTAATCCGAGTTCGGATTGATCGAGTATGCTGTTATACGCTGATAAAGATTTGATTCCCTTTTTCTATCGCAATGTCTCTTTTAGTATATCAATATATGCGTCTATATTTCTACTTCAGTGACCCATAATTTTATTAGTCGGCAGACCGTTGCTGCCTAACTGACTCTAAAAGCTTTGTGCTGACAGGTAAAGGGCATCTGAAGTACTTCTACAATCCTGTGTATACACATTAAAAGTTGGTTAGTATCAGTTTGAGTGGTACTCCATCAGTGGCTCTTCTCTGGGACCCATGAAACAGACCGACTGCTGCAGAGGCTCTGTACGTCCCGTTCTTTCAGGTAAACAAAAGAGTATTTGCTTCGACCCTATGGCTGTTACCGTTCTTGTTTAGATCTTGCTGATGGATATTCATAGTTTAAATTTATTAATTTTGAAGTCTCTAATGGGCTCCATTCATTGCATTTATACAGATTCAACCGCAAGTGTGGCCAGAGATAGTCCCCCT

>TCONS_00035809

TGGAAGAAGAAGCGTATGAGGAGGTTGAAGATGAAGAGACGAAAGATGAGACAGCGATCTAAGTAGACAGAGAGTGACTCCTCTCTTGAAGACTTCGCTTTCCAGTTATTTCATTTCGCTAAGTACTTTTGTTTTGATTTTGTTTCAGTTTTAAGTGTTCTTGTCTTCGTTTTTGTCCCGAGTTACGCTTCCTTCTCTCTTCTTGCTACCGTCACCGGGGAATATAGCCGAGTAAGGTTTTGAGAAAAAGAGTTCCTCCGCAATGAGAGCCAAG

>TCONS_00023749

CAAAAGCTCGTAACGCACTCGTCAACTCACCGCACTGGGCGTAAAAGTAAACGAGAGAGCTCTCAACAATGAGATTCTCCACTCTAACTTTCACCATACTACCGTGAACCTGCCTCCCTAGCTCAAACTCTGGTCTCCTACTACACAGATTCAACAGACACACAAACATCCTCCCGTTAGTATAACGAATCCCATGCTTCACGTAATCCTCAAACAGCGAAAACGCCTCATCTTCGAGACCGTACTTCAAGTACCCATCTATCATCGCGGTCCAAGTAACAGTGTTTCTCTCAGGCATACTGTCGAACACTTTACGTGCATAAACCAAATCGCCAAGCCTCACACAAGCACTAATCAAATTGTTACCGAAGTAGATCACTTGATCGTCGAAACACTTCAAGGCCATCGCGTGAATCCTTTTGATTAGCCGCATCCCGTTTGAAGCCTGTAGCCACTCCGCGAGCAGAGCGTAATCAACTCGGGAAGAATCATAGCTCGCATTCTCTCCGGGAAGCTCTAAACCGAAGTTATCGCTGAAACCCATATCGAAAGTTGATCTCTTTCTGGAAAAGAGACGAAACCCAGATGATCTTCTGTTCCTCTGTTCATCTCGCTTCGGTCTCGAGTCGGTAATGAGAGGAGGTAAAGTCGACGACTTGAGTGTTCGCGTCGCCGGAATCTCTGGAATCCGGAGGTGGGGTTGAATCAACGTTAACGAGAACATCGGAATCGGAGAAGATTCAGATAAAGCTCTCTCCTTTTTAAGAAATTAATCTGAAACTTTTAATTATGACGACAACAACAAATAATATCTCCGGTTGGGT

>TCONS_00074944

GGGATATCAAAGCACCGAGAATGCAAGTACTCACTTAATCTAAGTGCAACCAATGATTTAGATGAGTTTTAAGCTATGACTAAAACTAAAAAGCAATAACAGAATGATACTTTCTTGACTAAGGGAAAAGAGAACTCATGGGCATAGGGATTAGACCTTGGGTGATCAAGTATCGAACTAAGGATGGCAAACGATCAATCAAACTATCAACCTTAAGCCTAGACACAATTCTAAGCAAGCTCTATGTCTAGATGAATGCTCATTTGCTAACATATCTCAAACATCAAATGT

>TCONS_00004363

ATCGTCATGTTACCCATCGCCTCCGGTAACACGCCTGAGAACGAGTTGTTGAATATCTCGATTTGCTCGATGCTTTTCAGCTCCGTGATCCAACTCGGGACAGACCCGGTGAGTTGATTCATCGTCAAATCCAAGCTGACCAAGTGAGTCAACCGGGACAGAGCTTGAGGCACCGGACCAGCGAGGTTGCAGCCGGCGAGCCAGAGAACACGAAGCTCCGTTAGATTACCGAGTTGACTCGGGATTCTACCCGGTGAAAATAGATTGTAAGCGAGTTTGAGTTCTCTCAGGGTGGATACGTTTCCGAGAGAGGCGGGAATGGTACCGGAGAGGAGGTTTCCGGCGAGGTTTAACTTCTCGAGCTTGCGGAACTCTCCGAAGCTCGCTGGAATCGTATCTGATAAGTTGTTCCCGGAAACTTCGAAGTGTCGGAGGTTCGGCAGCGTGAAAGGAAGTGACTCCGGGATGGAACCAACCAAGAGATTCTCCGACAAATCGAGTTGGCTGAGATTCCGACACGATGTAAACTCATCGCCGGAGAGCGAACCGTTGATGGAGTTGTTGTAGAGAGAGAGGAAAGACAGCGAAGGAAGACGGCAGAGGACGGAGGGGAAAGGACCGACGAGCATGAAGCTGGAGAGATTAACGGAGAAGACAGCTGACGTGGCGTCGCAGGTGATGCCGCGCCAGTGGCACGGAGTGACGTCGTTTTCGGACCAGGAAGATAGTGACTGGGCCGGGTCAGATAAGCCCAGTTTGGCTTGCCGGAGGATGGTGGCATCGTGGTTTAG

>TCONS_00043611

GTGCAAAATAAATATATATATATATATACAAGGTAAGAGTATCTTACACTTGAACCATTGGCTAGCAGAAAACACACAAAACTGAAAACGGTAACTTCAAAAGTCTGTTTTGTTCAAACACTGAGTAGTTAAACTCAATATCTCTCCCGCTTCATTGTGTGTATGTTCAAGGCCCATCTGAATCAACAACCCGTTTTGTAAGCCCAGACGTGCTTCTCTAGATACGCAGCCACTGCCTCTTTGATAGCCAGATTTGGAACGAGCTTGGATGGGTC

>TCONS_00034468

CTGTAACAAACCTGCACAGAGACGGTAAGTTAGTTCCTATGTTGACCGAAGCTGGAGCTATCGCTGCAACAGCTGGAACTACTTCTTCTTAAAGGGCACAATCAATTTGTGATTTACTTCTCTCAGGAATAAAATAAGTTTGAGCAATAAATCTGAACTTTGTCTGAAATGTTTTAAAGTTGTGTTTATGTTGAAGTCGGTATTGAACTTCTGATACATTTATGTTATAATTATCAGCATTTGCTAGTAATGTCATTTCTCGTAATATTTGTAACGTTAATAGAAACTCGAATTTTAGTTTGGTAATATTTAATCAACTCTGGCTAAACTCTTGACGTGATGGTAGCCAAGTTCAATCTGCTCTGGCAGAATGGACTGGACAACGCACCGTTCCCAATGTGTTCATAGCAAGTCGTTTTGCCCATACTGTGTGAGAGTGAAGGAGCTTTTGCAGCAATTGGGAGCCAAATTCATCGCCGTTGAGCTCGACAAAGAGACAAAGAAAGCGAATCTAGGGTTTTCATAATTTCGAAGGAAGATGGCGATGCAGAAGGCTAAAGAGATCGTTTCCGGCAACGCCGTCGTTGTTTTCAG

>TCONS_00048757

AACAAGATTCATCAACATACAAACTCAAACTCAAGGCATTTCAATATCTTATCCATAACAAGCACAAGATAGTACCTTCTCTCCACTATCTCTAGTCAAGTCTGTGGACTCTCCACTCTCTTCCTCTTTTTCCTTCTCTTTCTCTTCTTCCTCTTTCTCCTCTTCTGTCTTCTCATCACCTGCCAAAAAAAATAGTCGCATGAAGCAATTGATTATATGAAGCAATTGTTTATATGAAGCAATTGTTTATACTAACCCATTGCTGGAAACCCTTTTAACCAGTTTCTAGTACAGACCAAGGCTTGGACATTAGTGGGTAGAAGAAAATTCCTATACTTGTTGAGCACTCTACTACCTATGCTAAATGAAGATTCTGAAGCCACAGTTGTTATGGGTATACTCAACACCTCTCGTGCCATTGTTGCTAACTCTCTGAACCGAGCCGAATTGTCTCTCCAGTAATGTAACACATTTAGTTTCTTGTGAGCGGCGTAGTCAAGCAATGGCTCATCCAGATATATGTCGAGAGCAGACTTGCCACTGCCAACAGCTTTCTGAGAAAAGAAGGCGTAGAATCCCTGAAACTGGTTAAACATCAGTGATTTAATATCAGAAATTCACAACACTTGTCACAAATAACTACAGAAATTGATAACTTACCCCATAACCGGCTGGAACATCGTGTGTTTCTCCTTGTGAAGTTGTTGCAGCACTACTGTTTCTCTTGTTTTTCTTGTAAGCTCCAAACAACTTATAGATCTTTGAACGAACATTAGCCAACCTTCTCTTACAAGTAGACTGATCCAAAACTGAGAAACAATATTCTAAAATCTCAAATTTCAGTCTTGGATCCAAGACTGCAGCAATTGCAAGGATGTCGCTGTACTCATCCCAGTATTTGTCGAACTTAAGCTTCATAGACTTCACCATTTCACAGATAACTTGATCAGAAGAATTTGCATGAGCTCTCAACCAACACTCAATCATCCAAATTTCATTGAAATACAAATTTGATGTTGGGTATGAGGAACCCGAAATCAAATCAGTAATCTCCGAAAATGGTTTCAAAAGCTCAGATAACATTTCCACTCTCCTCCACTCAGAATAAGAGGGAAAACTCACATAACTTTCTTCTACATCTGCCAAGTGACTTAGTGCATTTCTGAACTTTAAAGCTCTAGAAAGCATGAGGAAAGTAGAATTCCAGCGTGTGCTTACATCTAAGACCAGACCTCCTTCATCATCCTCAACTCCCACTGTTTCAACACAGCTTTTAAACATTATTTCCCTACTTTCTGATCCTCTAACGAACTTAACACTCTCCCTGATTTTTTGCAGAGCTCCTCC

>TCONS_00011616

ACTACTTACACCAAGCTCTCTCAACCATCATCATCAATGGCGAAATCAGCGGCGACGGCGACATCCTCCCTCGTGCAAAACCTCAGACGTTTCATCAAGAAGCCGTGGGAGATAACCGGACCCTGCGCTCATCCGGAGTACCTAGAGTCCGTTCCCAAGGCGACGGAGTATCGCATCAGATGCCCCGCCACGATCGACGAAGAGGCGATCGTACCTACCGCGGATCCAGAGAATGTATACAACATCTTGTACCACGCGAGGGATCGGCGCCGTAACCGTCCGCAAATCAGGCGATACGTATTGAAGAAGGAGGACGTGGCGCAGATGATGAACGAGAAGAAGACGGACTTCCCTAGGGTTTACTTGACGACCACCGTGGAGGAGGATGAGAACGCTCGCGGCGGAGGCTACGAGTAACGATCAGGTTTTCTCTAGTCTCTCTTTCCTGTAATCTGCTGGTGGGGGGAAGGATTGCGTGCGATATATCTCCTTGCGGTCAAAAGATTCTCCCTGGTTTGTTTGTTTATTTAATAATGATTCTTAGTAGCTTTATCTACGACAAAACAGTCTTTTCTTTCCTTCACAAGAACCAAATTGTTGTTCAGTCATATTCTCTGTGTTTACGTTCTATGATGAACTCATGTTGAGCTTTTGTCTCTCTCTTTTGTCTATGGCACACGCA

>TCONS_00009638

TTTTGAGTGATGAGGTCTGACACGGAGGCCAAGCCGTGGACGTTAATGGCAGCGTAGAAGCTGGAGACAGCTTCAGAGTTCAGACACAGATGTGGGAGCTGTTCGGGACGCAATGTCGATGGCTGTCTCCCACCACGCAGCTGCTTTTACAGACGGACTACAGGATGTGAACTATTGCCACCGAGTGATGGTTGTTGGTTTCAAACTATTGCCAGAAAGGTGAGTGAGAAGTTTCATCTCGTTGGTTACATATATTCTGCAAATATATCTTTTTATTCCTCCACTGCAAGGTATATCTTTTTATTCATCCAAGTATGCATCTTTATGGAGTTTTGTAGCTTGTGATTAAGTGTTTAGTGTAGCCAATCTTAAAGCTTCATTCGTGGAGGAGTTTAAGTCTGGAGTTTAAGTCTGAACGTTCTTTAAGATTTGAATAGTTTTCATTTTTGAAGGATTTGGTAAAGATCCCAACTTTTTAACGTTAACATGTGTGGATTCACAGGTTTATTCTTTGGACTGGACACCTGAGATGAACCAGATTGTCAGTGCATCTCAACAAGACACGCTCAGTTAGAAGAGAAAAGTGATGGAAAAGGGAAAGACACAACCAAAGGGCCAATTGATAAAGTGCTGAAAATGCAATCGTTTAGATCTTGAAAAGGTCAGACTTTAATAATACGATTAGTTAAATTCTATTTTTATATACTAATATATATCTAAATGGGTATATCACTCTGCCCACCTGCACCAAACCAAACTCACACGCTCACAGGTTTATTCCTTATCTCAGACACTTGTGGAGAAGATTATGGTCTCTGTGAGTAACCAACTTGAACCGTAGATCTACATAGATAGTTCGTTGTTTGTCTCATATAGTTTTGTTCATCAATTTCCAGGTCAAAGCTGCAGAGCCTTTTCCTTCAATCGTTGCAGCATCTAAAAGTATTGCAGTGGTTTCATCTGGATTGTGTGGAGATCACGGAGATGCCATACATTTGAATGCCGGATCTCTCTCTCTCTCTCCCGTGCCTCGCTCTGTCACGGTTACATGTCTCTCTCTCTCTCTCTCTCTGTGAGTTTTGATTTCGATTTGTTTTTGTCTTGGTAATGAAAGTTGGAGGAGGCAGAGAAGCTTGATGTAGGAGGACGGAGAAGAAGCTTCACGAGGTGGAGGAAGCGAGGTGGAGGAAGCTGGACAAACAGACAGCGGAAGAGGCGGGATGAAAAGGCGGAGGAGGAGGCATGACACTCAAGTTTGCCAGCGGTGGAGAGTTCAACAAGAAGATGGCGGCCTCTAGATTAG

>TCONS_00005687

AGGTTCTCGGCTTATACGACTTGTATGGTTGGAATCGAGCATCTCTCCGGAGACCGGAAGTGCTGGACCAAAATTTCGGATTTCTTTTATAGCGCTATTATCCTTGTGTCGGATGTAAGAGAGTCATCTTCTACGAGATGGCTAAGTCTGTTTAGGACGTTTAAGAGTTTGTTGTGATCAGCAACAAACTTAATGGTAAAAATTACTATTTTGAGTCTTCGCGAAGAATATGTTTTGAGAAGATGTTAGTGCGTATGACTGTCTGGTCTTCCATGAAGGTGTTTTTATCGAGGAAGGAAATTTCGTCGAAGAACTAATCTTCAGGCGTCCGCGACGTCTCGCGATGCTGAAGATTTGTTATTCTTTCGTATGCCACGTTTCGTGCTTGAAATGTTCGCGGGCTTTGAAGATATTTCGCGATGTTGCGAGGGTTTAGTATTAGCCCTGTGTTTGAGAAACATTCTGGTTTGACTAAGAATGTTCGCAGCCAAAAATTGTTGTTCCTGCTCGGATGCTAATAATTTTGTTTGCGATCGAGGAATTATGACTGAGGTGTCGTGTAAATTTGTCCATGGGAACAAGCGTTTTCCTTCATGGTGCTTTGTGAAGAGTTGGCCTTCCGAGATGTATTTCGTGCATTTTTTAGGATGTTTCGTATAGCTCCAGAAGCTTTGTTACGAATTTTTCCTTACTTGCCGCGTATTATGGTTAAAAC

>TCONS_00015892

GTATCAGCATCGAAAGTGTTACAAGAGACTTGCAACTACATAAGAAACTTGCACAAAGAAGCCGATGACCTTAGTGATCGGTTGTCTCAGCTTCTGGAGACCATTGATCCTAATAGCCCACAAGCAGCCATTATTAGGAGCTTGATTAATGAATAACTAAGATATATGTTGGCTAGTTGTGCTTTATATATCATTTACAATCTCCTGCGAGAGGTTGATCCATCAGGGTTTCTACTTAATTATCATTCATGTTTCTTATATATTGATTACAAGTTGAGCTAGATAGGGTTCATATTCGATAATCTTTTGCTATGCAACTAATAAGCGCACAAACAATTTTACTTCTTTTCTAACCCACAAGAGATAACAAAAAAAGAGAGAGTACTCTACTGGATATATATACAATCTCTATATAAAAAATTCAAAAAATGTCTAGCAGAAGGTCGAGACAAGCAAGCTCATCGTCAAGGATTAGCGATGATCAGATCACTGATCTTATCTCTAAGCTCCGACAGTCCATTCCAGAGATTCGTCAGAACCGTCGTTCCAGCACG

>TCONS_00046840

CTCTCGCTTTTGCCTAGCTTTTCGTTCCTTCGTTTTCCTCTTTCTCTTGTTCTTCCCTCTCTAAATCATCACCCATAACTGAGGGAGGGAAGAACGTACAAATCCTTTTAAAACTGAGTATATAATTGGTTATTATATATCAAATTAAGCAGTTAAAGAGAAGAGTGAAAGGGAGAAAATCTCTACTTTGAAAATGTCGGTGTCTAAACTTTTCTTCGGAGCTCTTACAATCGTATGGATAGTTTTCTCCATTTTTTTCCCAATGGCACAGGCTCAGTCCGCAGCTCCTGCACCGGCTCCGACAAGCGATGGAACAACAATAGACCAAGGCATAGCATACGTCCTTATGTTGGTGGCTTTGGTCCTCACTTATTACATCCATTAATCCTTTTGTCATTTTAATTTTGGTCTCTATTTAGTTTAATTATATTTTCTCATTTTGA

>TCONS_00005737

CTGAGATCGAGGTCTGGAAAGACTGAGCACCGGCACCGAGGACATTTCACGTTCAAACGCAGCCACTGGTCTATGCACTCCACATGGAAATTATGGGCGCAAGGTAAACCTCTAACCTCATGTCCAATATGGAACTCCTCTAAGCAGATTAAGCATTCACCGCAATCATCTGGGACGGCTTTTAACCTGAACTTTGGAAGTTCTTG

>TCONS_00056533

AGCTCTTGGTACTGAAGAACCGTTATTCAACATGAGCTCGGGGCGTTTAGAGGCATCACTGGATAGAGTAGAAGCCATTTTAACACATTCCGAATATCTATAACCAATCCGTTACTTGTGTGGTTTCTTTCAAACTAGTCTCTATCATCTGATAAACTGTTAGTATAACTCTCTTTTTTTCTTGGTTTTGATATTTAGATCGTCTTCGTGTTAGGTTGGTGTTGGGCATAACTTTCAAATGAACGCAATGGGTTGGAAATGGAAGCTTACCTCTTGTTTGGGTGGAGATGGTGTGTCTCGGATCCGAAACAAAACCACTCTTGGTTTGACTCCTGGTGTCGATCTGCGGTTTGGTTGGAGAGCTGATTTTGTACTCCCTGAAGTTACTGGTTGTATAACGAACCCACGAATGATTACCACGCAAAGCTTGTTCTGAAGCCATTATCACCAGAACGGAAGTGGAAGTTTATTTATGAGCCTTTACACCAAGAAGTTCGTGTTCTTTCAAAGAAGATTCCTCTCACCAGATTTCTCAATCTCCAGTGTCGCCGAAGTGTCGAGAGAGGGAAGGGCTTCCCGAAAATGAGAGGGAATCGGGTCTGGTTTGGGGATTTAAGATAGGGAATTGGTACCATTCGGTTTTTGGACGGTTATGTTTTAATTTCGGGTCGAAATTAGGGATTGGTTCGTGGTTCAACTCAATTCGGTTATTCCTTTGGTTAAGGTAGGTACAACCGGTAAATACTTCAAATGTTCTAGGGTTAAATGTAATTTCCGGCTTTTTATTTAACTTACCATTTTTGTTTTGTTTTCATGTCTTACAGAGATTAGAGATATAGCTGGGTGGGTATAGGAGATGATGTCCCTTTTGGAAGCATGGAGCCACCACCGAGTTCGCGAGCGGAGGAACCTCCGTCATGGGACGAGCTTTACAAAATCAATCTAATGCCATCGGAGCTGTTTCTCAAGTTCCGAAAAGAGCTCCAAGGCCTTCGCGTCGGCGTCAATCTCGAG

>TCONS_00074851

AGAAAATGATAAATGAGTCAAAAGTGCGGAATCTGATGAAGACCAGTCAGGATGACCGTTCGGCTACTATCCAGTTTCATCTGAACTGGCTGTGTCCAATGCGGTAAGGAGAAGGACGCACGCGGGACTGTCCGGATGGTCCGCCGGGTAAGAATGCATGTCCGTCTTTGGGTTCTTAACGACCCAACTTAAGTCTGGCTCGACTATTGTCCTTAGAATGTCGAGATGGTCGGGAAAGACGAGCTGTGGTACGGTCAGTTCGGTCGTCTGATCGTGGTTCCAGCCGAAACTCCATTCCGGACGAAGCATGGGTCGATCCGGTACACGGCTTGGTTTGTCTGATTGGTACGGTCGGATGGGCGAACCTCGGTTGAATTGTTCAGAACGTCCTGATCTCCATGCTGAGCTAGTTCCATGTACTGATCCATGGACTGGGGCTCATCACATGTCTTATCCCCATGTTAGGTATGACCGAACCCTGAACTGAATCGTTGATCAAATAAGAATAAGAAATTGTAGTCACACATTCTATGATCAACTTAACCCATTTCTCGTGAAAACCTAGTCTCTGAAGCACTTGTGAGATAAATCTCCATTCCACTATATCATATGTTTTAGACATATCAGTTTTGACTGCCATTGTGTCTTTCCCAGTATAAATGTTGACTGGTTCTCTGAAATAATCTTGCGTAGAGTTGGTTTCAGTATGAGTGCCAGTAATTTAGAGATAATCTTGAAATACACATTGCATAGAGAAATAGGACGATAGTCAGCCACCCTCTTTGCACCTATAATCTTGGGAATAAGTCTAACATTTGTTACATTTTGTGATGGTTTAAGGATTCCTGTCTCGAAGAACATTCTGATCTCCTTCACAACGTCTGGTCCTACTATATCTCAGTTAACTTGAAAAAAAACTGGCTAGAAAACTATCAGGTTAAGGGCTTTATCCGGATGTATTGCAAATATTGCTTCTTTAATTTTTAATGCGGTCAGTTACTTGATCAGTTGCAAAAGTTGCTGAGGTTGTTTTTAAAAAATAGAACCTAAAATCTAATAAAGAATCAAGAAAAATATACTTAAGTAAAAAAAACTAGAATTTACCTTTTGATTTATGTTCTCGTC

>TCONS_00083274

ATGGTTGAGAAGTGGAGTTACACTCCTCTTAACGACAATGCAGAAGCTGCAAAGCGTGTGATCCAAGATCTGGCTGAGCGTGGATGGAAAATTGAGGCGTGAACAAGTTTTGAAGAGTACTGTATTTTCTCTTTAGCGAATATAGAATGAGGTTGGTAATTAGCAGCGATCTGTTTGAAGATCGAATCATAAACTCAAGAGTGGCCCTTTGTTAGTGGTGGCATCTGGTCGTGATACTATCTCTGTCGCAAGCTCCATTAGACGACTAGCTATGGAATATGTCTTTGTTGTTCAGGTTAAGCAGATTTTTCGCATGTGATTCTTGTTTTAACTTAGTTTTTGTGATGTGGTTGGATTAGCAAATTGAGTTAGTTGTCCATTGTACATTCAGGTGCAACATCCAAGATCGCGTCTCGAGAGATTTGATCTAGTGATCACACCTCGACATGATTACTTTTCTCTAACACCTGAAGGGAAGAGGCAAACTTCTTTCTTTCTCAGGCCATGGGTGACTCCACGTGAACCTCCAGGTAGAAATGTGGTACGTATCATTTTTAGTGTTCACTAAAAGCGTTTTGATGTCAAGCAATTATTAACAAGCTCGAATTTATGGAATCCAGTTTTTGACTACCGGAGCTCTTCACAATGCTGACTCTTCTACTCTGAGAAACGCTGCTTTGGAATGGAAGGACGAGTTTGCCTCGCTGTCAAAGCCTTTGGTTGTTGTTAATATCGGAGGACCTACAAGTAAACACACGCACGGCTTAAACTTGAGTTGTCATTATAACTGCGATTAATTAAAACCTTTTTTGTTGATTTTGTGTAGGGAACTGTTTGTATGGTGTTGATCTCGCTAAGCAGTTGTGTGGTATGCTTCACAGTATCCTCTGGAGCTGTGGAAGCCTAAGAATATCTTTCTCGAGACGAACACCGAAGAAGGTAAAGTAGAAACTCTCTAGAGAATATCACATTCATGTGGAGCGGATATGGTCATCATCATAATCTCTCAATCTTTCTTCAGGTCAAAGAGATTATAACTAGAGAACTGAGGTCTAACCCGAAGGTTTACATTTGGGATGGAAAAGGTGATTTTTTTTTTGTTATTCTCTATCAGTGTCAAAGTCTGATGCTTTCTCTCTTCCATTTCTCAGACCCTAATCCGCATTTGGGGCATCTAGCTTTAGCCGATGCTTTTATCATAACTGCTGACTCTATAAGTATGTTGAGCGAAGCGTGTACTACCGGGTATCCTTAAGAGAACAACTACCTTTTTCCTTTTTCTCATTCTTTAGATGATACTGAAACTTCGTTCTCGACGCAGGAAGCCAGTGTATGTTGTGGGTGCTGAACGGTGTACATGGAAGTTCTCTGAGTTCCAGAAGACCCTTCGTGAAAGAGGAGCTGTTCGACCTTTAACCGGCAAAGAAAATGAATCGAGGATGTGTTTGAAGTAGCTGATGCGAAGCAGATTGCTGCCATGGCTCGTCGTACGTTTGACAAATGCGACCAACACAGCCACCGGAACTAGCCGTCACCGGTACCCCCGAGAATCTTCAGAGAGGTGTTTCCGGCGTAGTGAAACGAGCTATCGTGATCGGAAACGGCTGCGCCGGCGCGGAGAATCAGTGCTTCGGCCTTGTTCGATCCTTGGGCCTTTACGATCGCCACCTCTACTATGTAACCTCCGTCCAAAAAAAAAAACACTATTATTTCATCGGAGAATCTTAAAGTTTGGCTCTTTATTCCATTTTTGAATGTTTATGAGTTTTTTTCTACTGTATAGAGTGTAGCTAGGCCAAGAGGAGGAATCAGCAGGTGGCTTCATTGGCTTCCTATCTCTTTTTACAAAAAGCTTAACCATTTCATCAGCTGTGTATGTTCTGGATTCTCCATTAAAGCAAATGAATCGGGTTTCAGAAGATCTG

>TCONS_00013898

TCCGACAAAGTTTCCTCCTCCTTGCTCCGACGTTCGGAGACACCGGTGTTTCGTCGGTGTGCGTTAGCTCCGGGCGTCGGAGGTGCTTCTTCTCCTACATCTCGTTTTTATTTTGCTTCATCCCCTTGTCTCTCTTCTCTTTCGACATGTCAACTCTCTGGTACGACTTTGAAATCGGAACCCTAGATCCTCCCGGGGGGTTATAGATCCGGTGGCTAAGGCCCGATCTGGAGCTGAGACGAGGCGGAGAGGGTTGCAAGATGGCTGGAGTCGGCTTTTGCGGTGGAGTCTCGCCAGATCTACTTTTTCCTTTTTAGATCTGGTCGTAGCCTTCGGCGGTGGTGGCGCGTGGGTAGTGATATTGACTCTTCCGGCCCGGATCTTTCTGATTCCAGCTTGGTGAGGTGTAGCAGTCGAAGGTTTCTCTCCCTAGCCTTGGTGTCTCGGCGTGGGCGCACCTTCTCTCTGCTCTCAAGGTGGGCTTGGCGCTTCTTCTCCAAGACGTGCGGTGGTGTATTTTCTGGTGGCGGTGTCACTTGACAAATCCCTCTGTCGTGTGCGAGGATATGGTCTGTTCCGGCGTTCGGGGAATGGTTCTCAGGCGTTGGATTTCCATCTCTTCTCCCGATCTTTGTCTTGCCTCTCCTGCGGTCCTGTCTAATGGTGTTCCTCCTCCGGGTTTTGCGCATCACCCGGTGTCTTGGTTCGAGTATTGGTCTTCAAGTGGTGTTTGGCTCCGTTTCCTTCCGACGGAGTCTTCTTCTCTCTGCTTCAGTAAAGAACTTACTCTAGTTCTTAGGGTAAGAATACGTGTGGAGTCTGGTCTTGCTTCGCGAGTCGTCGGAGCCGTCAGTTCCTCGGTTGTGGCGTATTTTGGTGTTCATCCATCCCAGTGGTGTAGCGGTCTTATGGCTGCTTTGCCAGGATGAAACCTTTTCTTTTAGTGCAGGTACTCTCCCATCTTGGCATGACAGCTTCCTCAGCAAACCCACTTTTAGCGGGATTTGGCTCTTTGGTATGACCCTAATCCCTTCCTCCATTGCGAACTTCACAGTGACAGCATTACAGCAAGAAGGCGAGGTTGGTTGCGAAAGCACCAAACCTGGATTAACAAGCTAAGTGGTGAAGTTGATGTCTGGCCCGGGGTTGCTAGCCACAACTCGACTGTGTCGTCTTTCGGATTCGTAACTACAAAGCATGGTTG

>TCONS_00082025

GTTGACCATCTTGGATATATGCCGTCTGTGAGGTAGTAAGGCAAACCATATTGGTGCCCGTTTACCAGGTATTGTACCCTTGGAGCTCGACCTTGTAAAATGTCATCAAAAACAGGAGATCGATCGAGGACGTTAATATCGTTTAAGGTACCTGGAGGACCAAAAAAAGCGTGCCAAATCCAAAGATCTTGGGAAGCTACAGCTTCCAAGACAATGGTTGGCTTTCCTGATCCACGTGTGTACTGTCCTTTCCAAGCGGTTGGGCAATTTTTCCACTCCCAATGCATACAATCGATGCTTCCTATCATTCCCGGAAAGCCGCGAATCTCTCCAATATCGAGCAGTCGTTGAAGATCCTCTGGAGTGGGTCTTCGCATATACTCTTCTCCAAATAAATTAATTACCCCTTCTGTGAAATTCGTTAAACATGAAAGTGCCGTGCTTTCACCAAGTCTGAGGTATTCGTCAACGGCGTCAGCCGCACAGCCATAAGCAAGCATACGAAGAGCTGCCGTACACTTTTGTAGTGGAGATAGACCTAATCTTCCGACAGCATCTCTTCTTTGTTGGAAGAAAGGATAATTTTCTGATAGACGATCGACAATACGCATGAAAACTTCCTTGTTCATGCGGAAACGGCGTCTGAATAAATGAGGCGGAAATGTTGGATTTTCACTGAAGTAGTCATTCCATAGACGGGAATGGCCCTCTTCGCGGTTTCTTTCGACATATGCACGTTTTCTCTCTTTCTTTTTTTGCTTCTTTACGATGTCGTTGTATGTATCTTCCAAATATTCATCGACAGCTTCATCTAAAGCCGCATTTAATCTGCGATCCACTTCATCTCCCATATTTGGCTATATTTCGTTTTAAAAAAATGATCATGATTCGTAAACTATAGTTTGAAATAAAAAGAATAAAATATAGTTTCAACCTAAAGAATAATATTTACTTTTCTTTGTAAACGTAAACTATAGTTTGAAATAAAAAGAATCAAATATAGTTATAAATTTACTTATCTTTGCTTTAACAAAATGTTCCTTTAGAAATCATATTCAGAGG

>TCONS_00012563

CTCATTCTTTCGGATGCATCTACGTTTCTCTCTGGTCCTATTATTTTCCTCGACCGTCTAACTACTTGATATCATTCGATGGATATTACAATGAAGCATCTAACGAGCCTTCTACGTCTACTGGAAGTTTAGCGAGAAGATTGTTCCATGAGTATCGCCAATCCGTCATTCCTCTCCAAAATGGACATATACCAAGCATGGCGTTCATGAATAATCTCCCATACCTAGAGATTCAGACACAAGATATTCAAAGACTTGCTTCTAACGACGCACAGCGTCTCTTCTATCAGGTCATTATCAATTCTAACCAATAATGCTAATACCGTTTCTTGTCAATGATATGAAAATTCATTGCTAATCATTTTCCTACTCGTTTCATCAAATTTCACAGGAAGCAAGGATTCAGGTACATTTTTTATTTTTTGTATAACTTCCAACCAATGTAATACAGATATTATATTCAATTTATGTATATATAACGCTGTATTGAATATGTATAATGATCAGACGGTGATATTCATGGGTTGTCGGAGCGGGGAGATCGAACTCGGATTGACGTATGATGCTGCAAATGTATGTATTTATACTTATAAATAATTCAATAAAATCATTATTATTATTTTTTGTCTCCCTTTCTCTATATAAACATATATTAATGCTGTATGTACATGTACATTGACCCAAACGACAGATGAAAGTAGAAGCAAGTCTTCGAGATTGGTTCCCTGAAGATTTCAGTAGAAAAACTTCTCCGGTCAACTCAGACTATCTCCGGCCACAGCCTCCTCCGTCTTCATCCTCTTCTTCTCTTAGATCACTAGACAGTCCCCAAAACGCCTCCGAATATTCCTCTCTCTTATTCCCACTCATCCCTAAACCTTCAACGACGACTGACGCCGTTAACGTTCCGTTGCATACGCTGCTAGCTCCGGTCACCACAGCAGAAACAACGACCAACATGATCCATCAACAACAACAAGAGCCTTTGTTTCGCAACCGTGAACGTGAGGAGGAAGTAATGACGCAAGCTATCTTAGCGGTTTTATCGATGTCTTCAAGTCCTTCGTCGCCGCAGCGAAAAGGAAAGGCCACCGCTTTTAAGAGATACTACTGCGTGGCTAGCGGCGGCGGTGGGAGCGGTAGAGCACCGCAACCGCCGAGTGTACGGAGGCAAAGTATGATGAAAAGAGCTATTTCGTTCTACAATAGGCTTAACATTAACTGGAGAGAGCGTTTTCCCGCTACTGGCGGCGGCAGTGATGGAATCGGTGGAAGCGGTGGCGGGTGGGAGCGGTAGAGCACCGCACCCGCCGAGTGTACGGAGGCAAAGTATGATGAAAAGAGCTATTTCGTTCTACAATAGGCTTAACATTAACTGGAGAGAGCGTTTTCCCGCTACTGGCGGCGGCAGTGATGGAATCGGTGGAAGCGGTGGCGGGCGTGGGCCAACCGCAACGCAGTTGCATCATATGATATCGGAGAGGAAACGGCGAGAAAAGCTTAATGAGAGCTTTCAAGCATTAAGATCTCTCCTTCCTCCTGGAACTAAGAAAGATAAAGCATCGGTCCTCACCATTGCAAGGGATCATCTAACTTCTTTGCAAGGTGACATTTCGAAACTACTAGAGAGAAATCGAGAGCTGGAGGCTAAGCTAGCGGGGGAAAGAGAGATGGAAATTTTTTTACAAGCCGATGAGAGGTTTAACGTTCGTATAATACATATACCCGAATCCACATCCAGAGAAAGGGTTTTGGATCTAAGAATTGCTCACAGAGGAGACAACATTGGGGCTGATGATTTGATCATAAGGCTTCTAGAGTTCTTAAAGCATATCAACAATGTGAGTTTAGTATCAATCGACGGTAAAACCCGAGCTAGAGAAGATGGAGTTACTTCGGTCATTCTCGTGAGCTTAAGGCTCAAGATTGAGGTAAAATAACAATCTTCACTCTATTATCTTATTTGAGATTTGTTAATTACCTAAGGTTTGCAATTAACTTATTAAAAATGACTTACTTTGTATATTTTTTCTTTCAGGTAAAATATTTTTTTTAATAATACCTTCTTTCATTCATTTTCAATTTTGTATAAGTTAATTACTCATACTCATTGTTAGTCAGGTTATACAGAAGGATTAAGTATTTTGTAACGCTAATAGTTGCATATAGTTTTTTGGTCTACTTTTTATAAATTAATATCAAAAGAAAGTTCACATTTTCTAAGGTGTTATTTAGAATTTGGTGGGATTTATTCAAGAAATTGCATTGATTGGTTGGATTATTTGTTAGGTGGAAATTTATGTGATATATACATCCAATTTTTCTTAAAAAGGTCAAAATTTTCTCTTCCACAAATTTATTTGATGGTGTCTATATAATTATGTATAATTGATAAGATCATTTGATAACCACTAGTTCGATCTTGACTAAAAGATTTGATAGTCGTTGTGAAGAAAAGAAATAAACGTCAAAGTTTGATTGTTTTGTAGGGTGAATGCGACGAATCAGCCTTCCAAGAAGCAATCAGAAGAGTTGTTGCTGACTTGGCTCACTGACAATAAGTTTGATTATTTCCTAAATCCTAACCGTATGTTTAATTCTTATAATCATAGTTTCGTTTCTCTTCTACG

>TCONS_00057758

TGACAAGAAGCATCCTAGAGGGATTGGAAAGAATTAACTATAAGGGAAAACGAAGCAGAAGTACAATTCGAGGAGGGAGGGTGAAGCTGCAGTCCTAGACGGAAGAGAGGCTGCAGTCCGAGACGGAAGAGAGAGCAATCTATCCCGCATACTACGATCCACAACTTTGAAGAACTACGATCCACAACCTTGAAGAACGCCTCTTGAGTCATAGAAACATCTCTGAAGATACGGGCATTTCGTTCACGCCAAAGGTTGTAGATAATAACTTGATTCAAAAGCTTCAGCACCGCAACAGCTCGAGACGCATGGGGCCCTTGCAGCTGTCGGCATAGATCAGTCACCGCCGCTACAGATGTAGGTGGAACCGCCAAGTACCTGCCACAAAATCGATTCCAAATGACAGTAGCATAGGAACACTCAAAGAACAGATGGGAATGAGATTCCACAGCATTAGGACAAAGAACACAACCTGGTGGAAGAGTTATGCCCCAAG

>TCONS_00017827

GGATGAGCCAACGGAATGAAGTGTGCCGCCTTACTCAAACGGTCAACAACTACAAAAATCACCGTTTTCCCATATGACTTAGGCAAGCCGTCTATAAAGTCCATTGAAATGTCGATCCAAACAGCCTCAGGAATCAGTAACGGTTGCAAAAGACCCGGAGATGCCGCCGTATCATATTTATATCTTTGACAAACAGAACAGCTGCACAGATAAAACTGAATGTCCTTAATCATTCCCTTCCAATAGAATAACCCTTTCACTCTCTGCATTGTAACATCTCGTCCAGAGTGACCTCCTTGACCTGAACAGTGCAGCCATTCCAACACCTTATCCTTCAGACTCACCATTGCAGGTACCACAATCTTACTCTTACGCCTCAAAACCCC

>TCONS_00026939

ATTGTAGCGTATCTTCTCATATCAGCTGCGTCCTCTGCGATTCCGTTAACTAATAGGTTCAGAGAAGGTCAAGATAACATTTTCACTGATTCTGCTGCTTCAGCTATCACCATGGCTGTACTCGCCTTTGTGTCTTTAGCTCTCTCTGCTCTCTTCTCTGGTTACAAACTCTCTACTACTCATTCTTTTGTCTGAATACGTGGTGGCCATCGCGATTCTCGCAACGTTATACACTGCGTGGCAAACGTTTGTACATCTCTCGAAAAGAGACTTCTTTGATCGCCGGACATCAATATTTGTCGATTTCTCCGGCGACCAGCGATTCCAATCAATCACAGCTTCGAGATCAAACCGGCATGTCGACGAATCCCGATAACCAGAAACCGGTCGAACCTTCCGACGTCGAGGCTGCTGCGCCAACGACGTTTTCCGCGATTACTCAGAGATGGAAAAGAGAGGACACGATAAAGAAAGCATCTCCGATCACGAGAGGACTAGCTCTTATCTTCTCCATCCTCGCCTTCGTAATCATGGTCTCTAACAAACACGGACCCGGTCGAAACTTCGACGAGTACGAAGAGTACAG

>TCONS_00033779

CATTTTATCATATAATGAAATAGGCTGAAACTGGTCACTGAGAACTTCTTGACCACAAGGCCTTCTGAATGGTTGACCACATTTTATTCTCCTGATGATAAGCTTCTTCTCATTCTTTCTGCTCCTTTTTATAGTCTCATTTCCTAATCACAGGACCTCTGTAAGGAACTTTCCATATCACAAAGTTATCTGGAGGCTGATACTCTTCAAGCCATGTCACATTTGATATCACTTCTCCATTCATCACTTCAATCTCACCT

>TCONS_00032217

GGATTTGTAATTATCATCATTGGTCGTTTTGTTTCGAGAGGTTTCTTCTCCTTTGGTCAAGTCTTCAAGCTAATCCAATCTTGATGGTGAGTAGTTGCGAGCTTTGCTGCGAGATCGTGATCGCGATCCTTCTTCCTCCCGTCGGAGTTTGTCTCAGGCATGGCTGTTGCACTGTTGAGTTCTTCATTTGTTTGGTTCTCACTTGTTTGGGATACCTCCCTGGAATAATCTACGCTATCTACGCCATCTTGTTCTTGAACCGAGATGAGTACTTCGATGAATACAGACGCCCAATCTACTACGTTGCTTGATCATTGTTCTTGCATGGAAACATGTAACATATTCGTTACAATGTGATCTCTGTATCTTTTAATTACCATTGGACGTTGTGTTTTGCTATAAATATAATCATTTAAGACAGAGAACCAGGAACATGTTTCTTGAAGGCGTTTTCTTATGACCCATCTTATCAAATGTTCTGGT

>TCONS_00013865

CTCTACATAACTTCATTATCATTGCAGCAAAACTGTACAGACGCGTAGAAGCATTACGAAAACGAGATGACATATAAATACAAAAAGAGAAGATACACGTAATCACATACACGCAAACAAACTCAATACACTGATTGATACACACATAAACAAAAGAGAACCTCTATGTCCTACCAAAGGAAGAAACACGAACTCTGCATGGAAGGACCACTTGATGTGTCAAAACGAAAGCGTCTCGGGGGCTACTCTGTTCTTTCTTCTTCTCTCTCTCTCTCTTTAGAGATTTGGGGTTAGGAGATTAGAGATCACACGCCGTAAGTAGGAGGAGATTTCCTTGGTCCATTCTCTGATCCTGCTGCCTTATCGAACGCGTCCGATGCCGACCTTCTCCTAAACCGAAAGGGTTCCTTTCCTCCTGCGTTTGCTCCG

>TCONS_00030516

TTCCACAAGCATCGAGTTAATATCTTTAGCTCTCGGGTGAGAATCATCTTCCACACAAGAAACTCATGAAGCACACCATCAATGTCGATCCAGCTACACCCTGGGATCTTTTCAGCCTCATCTCCGAAACCTCTGACCAGTTTCCTTGGGAATCTTTTCAAACTCTAAAACTAACAACAGTGAAGTAATCCTAGTAGAGCACAAGTTTTTATGCTAGCCCTGAATAAAAAGAGGACAACTCTAGTTAAAATTAGATCAAAAGAAACGATTTTGTGAAGTAGTTGAAGCCGACACTAGAAGTCAGAACCCAACAAGAATATGTTTTAAGGTCTCACCTCTCCAGCACCCAAGAACAAAAAGGTATGGTCAGCTAGGCTTTTACCAAGCACTTTCTGAGCCGCAACAAGCTCAAACACCACTGCTGCAGTACCCTGAAATGTTACACAGACAACAACTTTTTACAGAACTCACAAAAAGTTGAGATTATGTTGAAGACGAGATAGAACTTAGAGAACCTTATCAAAAAATGGTAACAAATCTGAGATGTGTCAACCAATACAACAGGAAGCAAAACAAAATCAGAATCAAATGACAAACAAACCGAGTCTTACTTGTTTCAAGCTGTAAGTGCCGAATCCAGAATATGAAGACGACATGAGTAGTTGAACAAGTGATCATTTCTCTTTAACCAGAGAAACTGCAAGAGGCTCACAATCACCTACTCAAATTCAATTCAGTTCTACTTAACAAGAGAGCTAGTTTAAGAGCTTTGAAGATTGCAATCATACTGTACCAAAACAAAGTTCAAATCTTTAGACCTTTCCCAGACATAAGGACCAACTCGTAAAGCAAAACCATAAAGATTCGTTCTTTTTTGATCTTCTTACCTTAGGAAGACCTTGGGTTGCTTTAGAAATGCTTTCTCAGTCTGCAAGAGAGAGAAGAGGATGAGAATATTCGCAAGAGACAAAGAGAGAAGAAATAGAACCTGTTCAGCCATGGCTACTGGTTGCTGCTCCTGCATCCGTCGATCTCCTTTATCGAATCGGAGGAGACGCACGAAGGAGCGAAATTGAGGGGTTTGCGGAGATGATTCTGGGGCGTAATCGTAGTTCCATGAGAGATTGGTGGAGACGAGGATGATGGATTTGCAGACTGCAAGGCTAATCCATATGTTGTTTCTTAGATTGGAATTAATGGATAAGAAGAAAAGAAAACTCAAACAGAAAAGAAGATGGAGGATGAAGAGATATCAATTCAACTAAACAACCAACAAGAAGTAGATAAACAAAAGGAAAAATTGGCAAATTGCGCAAATTGCAAGTTTGGCAAATTGCAAGTTTGATATTAGGGAGTTGGG

>TCONS_00032405

CTGGACTATTGTCTTAGCTACCATCTGTCTAAACACAGTATGATCATAATTGCGAGATACCACTTTCCCTTCGATATTGAGTTGTTGCTGCCTTCCTCGATTCCTTGGATACATTTTACATCTCTCCCGATGTCTTCTCAACCCACTGGTTCCATGCGAATGAGATTGCCATGCATACTCACGTTTGCAATGATTGCATGCAGCTTTCAACTTTCCATTCGTTTTTTCGACCACCTTGAAATCTCTCCAGACATCTGATTTTTGCCTCTCTTCCCTTTCCTCTTCACCGATTTCATCCTCATCAAACTCTTCTTCATCCAGTTCATCACCAAGCTCGGGTATGTCCTCTGCGACCTTCTCCTTCTTCTTTTGCGACTGAGTAGGCGAAACCTTCTTCTTTTTGGACTTAGTAGGCGACTTTCTTCTCCCTCCACGAATCACAGACTTCATAGGAACAGACTTCCCCTTACTACACGCCTGTGCAGACGAGCCAACGCCACCATCGATAGTTGATTGCAACATGGGTTTCCCCGTGGCTGTTCTGAAACTGGTCTGTCTGCGAGGCTTGGTTTTTTGGGTTGGTTGACTGATTTCAACATCAGAATCATCAGTATCATCAACAAGACTGATAAGCCTTTTTCTGCTTCCTTGATGCTTCCCATCAGCACGAGTTACACCAGCTTCCTGAGAAGCGATCTGATCACCAGCCTCAGCTGCTGCAGCAAGAGTAGCTAAGACTTCCTGAGTGGCAAAGTCCACAGTATTTGGCGGGGTGTAATTCGGATCAAACGTCGACTGATCTTCG

>TCONS_00017456

ACCAATCTTTCATTGCTTTCCATGATAACGAGTGAGGAGTTCATGTCCATGATGACAAGTACGTGATGATATATCTTTTACTGATTATTTTTGCTTAGTCTGGGTGATGCTATTTTGTGTATTGTAAACGCTCTTATTTGTCAAACTGTTTGCATGAAAATTTAGTTATTAAGCCAGCCTCTCTGGTGTTCTTCCGGAACTATCCTAGAAATACATTCTTTTCAAAAGACAATCGTTCAGATAACTATGATCAATGCGTTTTTTTTGTAACATGATCTTCTTATGAGCCAAATTTCAAAGTTCTTAATCTTTGTTTAAACAGAGAAATTTTCATGGAATTTGATTCAATTCCTACATCTGAACTAACAATCAAAAAGAGAGAACATCCCCTGAGTTGCTACCACTACATTACTATCCATGATCCAAGGCTTAATGGTTTGCTTTTTTTTTTTACTTTTTTTTTTTGTTACAGTAATTCTCTGGTTACTACGAACTTTGTCACTACTTTTTGTATATTTGATCAGAGTAGTATTCGTGCTTGCCGACATAAGCGAGATACAATTCATGAGTTATCACCTGTTGTAAAACGTATGATCAGGAAGGCTATAGATCATGTAAGCTTAAGCATATTAACCAGAGGTGGAGATGGTCGGGTCATTGTGGACGCTTGCTGTGTTCCCCCCAATGCCCTGGCTCCTTACTGTGCTGCTACTCAACTCTCTGGATCCAGAATCTGGATCTTTTCTATCTGTTTTACTCACCATTACTCAGAGTACGTCTCACAACGTTAAGGATACTGCAATAAAAGATTCGGCTGCAGAATGCCTGATTAATATAATTGAGACTAAATTATACATATGAAAATGCGAAGAGATACAAACTTATTTTAACGCTATAGACATGAAGGGCTTGACATTCTTGCTACTGGTTCTCACAATCTAACTCCATTACATAATCCAGTGCATCCAATCGTCGTGATTAAACCTCTCATTAATAGCATATTCCCACTCTTCATACGATTTCACATTCAATCCATCTACTCATAACTTCTACGGTGTGAATCTATAATCTATAAATATGTGAAATTTTTCTCTTTTTCTTAATCTTCCTGAAACTTTCGTAAATCTTCTGAGTCGTCACCGAAGAGTAACGCGTTTCTTCGAGCGGGAAGAGATGAAAAGGCCGGTGATTGCTTCTCTGGTGGTCGTATAAGATGCGCTTGGATCACTCCGAAATCTG

>TCONS_00039234

AAAACAAAACCTGCAATCACCTTTGCCCGTATGAAAAGCTACATGTCATTGCCATTTTGTAGTAAGTTTTGGAAAACTTCCTTGTAAACTACATTCATTGTTGACTCTTGTGGCTTTCCCTCCTTGTCGGTAATAAGAATCTTTAATCCTTTCCTTGATTTAACCCTAGACACAGCAACATACAACTGTCCATGTGAAAAAACAGGTCTACGTAGAAACAAACCCACTTTCGCTAGACTTTGACCCTGACTTTTATTGATGGTCATTGCAAATGCAACTTTTAAAGGGAATTGCCGGCGTCTCATTTTGAAAGGAAGCTTTGTATCTGATGGTGTTATCAGCATACGGTGAAGAAAGACCTTCTCTCCAACTCGAGCACCTGTTATAAATTTTGCTCCAATAAGATGATCAGCCATTTGTGTTATCTGAAGTCTTGTTCCATTACGCAACCCTTCTGTAGGATCTAGATTTCTGAGTAGCATAACTGGCGTACCAATTCTTAGCCTTATAGAATGATTAGGTAAACCAGAGGTCTTTATACTATTTAGAAATTCTGGACAGAAAACCGAGTCATCCTTAGAGTTTGTATCAGCTGGATCAATACTATCAGCACTAAGATAAGTTCTTTCTTCGCC

>TCONS_00072755

CTGCATATTCTCTCTTTCGTTTTGTTCTTTAGATATTCTTTCAACATATCCATGTTTGTTGCTTGTCTAGACCGTGTGTTCGGTCGGTCTTGTTGGAAAACAGATCTGGATATTGTGATGTTTTTAAAATCTTCTATCCAACATTAGATGTTTATTTTGGTCGATACATAATACTTCTTAAGGTTCCATTCAAATAGTAGGTTGGTTAAATAGTTTTCATGTATTCTTCATTGAAGATAGTAAGAGATAGATATCATCTACAACTTTTTCATATTTTATGTAACATGTTACTTGAAATTATTTTGGATATTGTGATGTGCAAAAATCTTCCATCCGCATGTCATGTCGTTTTCGTAGGCAGAGCGGCCATCTTTTGCACAAATCAACTTTTTAATGCCTTTCTTCATGTGTTGACCCTTCGGTGTGTAAAAGATCGCAGTTTCAATATTTATTTTCAGTAATATTGAAATCGATTTTTTACCTTATGGCATACAATTTTACTCTCGATATGTATCTAAACGATTTGTAGTATATTTTCTCTATGTTGATACCTTATTGGCTTTATGGAAATCCTACCTTTAGGACAGTTAATTATTTACTCTTTAAAAAAAAATGTTTGGTAAAAATTTTTACTCCTGAACAGAGATTTACCCATCTAGTTTTGTAGTTTTATGTGTTCTGAGACTTGTG

>TCONS_00072533

TGAAAGTTGGGATTCCTGAGTTCTTGAGTGGGATTGGGGGAGGAGTTGAGACTCATATAGCTAAACTTGAGACAGAGATTGGTGATCTTTCGAAACTGCTTGTGACTCGTACCCTCAGGCTCAAGAAGTGTGGCGTCCCTTGCAAACATGTAAGTACTATCAAACCTTCTGTTCTGCTTTCATAGTTTACACAGAAAGGGTCCGAGGAACCAAGAGGATACTACTAATATGTTAGGATTGAAACAGCCTGGTACTTTGTATTTGTTCCATTATGTAATCTAGTGAAGTAACTATACAAGCATAGGAGAAAAGAAGAAGAAAAAAAGTTGAGTTCTTCAACTATTCAGGTACAAAGCGATTGATGGAGATGTATGTATAGTGCTAGTAGGGAGTCTTATTGTTATCACCCAAACAAAAACTTGAAGTGTAGGCTTGTTCACGCTTCCTGTCACTCACTAAGACATCTATCCTTTCATGTAAATGTTGATAGGTTTTGGTGAACAACACACCATCAATAATAACTAGAAGTGTCTCTATTTTTTTTACTCGTCCATGATATTTTTTGTTGACAAATCTGAATTGTGTTTTTTTTTTTGCAGAGGAAACTGATACTGAAATACAGCCAGAAGTACAGGCTAGGGCTATGGAAACCCAGAGCTGACGCTATAAAGGCCTGATTGATTCGTTTCCTCTTTTTGAGTCTCTGTCATGCTTGGCTTTTGCTACTGAAATGAGTTGTTCTCAGAATGGGTCAAGGCATAGCAATGTTTACTTTTCTGCATTCATGTACTTGTTGTTATCTCCTTGGGTCTAGAAACATTAAAGCCAGGCTTGAAATCCGACAACAGTAGTAGTTTTTTGAAGTATTGTATTGGGGCTCTCCCAGTATCAAGACCGTCTTTTTTTTTGTCGTGTCAAATATGCATGGCGTGGATGCAAATGATACAGAGCGCGAGATCTCTTCTAAGGACAACTCAACCTTCTTCGACTCCATGCCTCTCCAGATTCTACTCCAAACCTGCTCCTTATGCCG

>TCONS_00038361

GGCCAATCACGGGCCGCCATGTATCGCGGGGCCCGCGCTATAGTGATGATCCGGGTTCAGTGCAGTGAACTCCCTATGGAGTTCACTGATGCAGATGCTCTTAACTGCTTCTCTTAGTATTAAATAGATTAAAAATACTCAAATCACAGCCAAAATATGAAGACACGCAGCTTAATTACGGACGCAAAAGGGCGCCTCTTGACACACGTGTCTGAAGTTAATTGGGGAGAGGAAAAATGGTGCCGCGTTGTTTCGTCTCGGTTCTTCATTTCTCTATCTCTCTCTTTCTCTCTTCCTTCTCTCCTCTCCAGACGACCGAGAAGGCGATATCTCTTCTTCGCCGTGGCTGTTCTTCTTCTCGGTGGCTCTCTAGGCGGGTCGTGTTGATGAAGGAAAACGGAATCCCCTCTATCGGTTGGTCTCATCGGCGAAGGATAACAGCGAGATCTCTCTTCTTCGCCGTGGCTCTTCTTCTTCTCGGTGGCTCGAAAGAGGCATGATCGAAGCTCAAGCTTGGGCTTTGTCTGACAAACCCCCACGATTGTTCATAAG

>TCONS_00043796

GTCTCAAGCCTGTCCAGTACGTGGCAGAAGCTGAACCAACAACCAGCTTAGACTCCACCGATGGTAACCTGATCACATGGTTACGCAAAGGGACATCGATCAGTGAAATATTCAGACAAGATCCGTTCTTGGTTCTATCTCTGCTGTTTATCTGTTTACAAATGGCAATCCTCGTCTTCCCCATAGCAGAATCTCGCATGAAAGCGTTGTGGGCTTCTTACGTTTCCAATCTAAACCTGGAAAGGTTTGGAGAGATTAGTCAACTGTTCAGCCGCGCTCTTCACATGGTCGATGTGAGGAGGCTGTGGTTGAAACTAAGACTCGTCAAAACAAGGAACTTTCATGAGAGGGCAAAGAACGCACAAGCCTGGGCTTCATCGCTTGCATCTGTCTCTCTAGGCCAAGCTTCATCAGACCAGTCCTGATCCCTTGAAGAGATCCTTCTAGTATTTAAGCGTGTTCTTGAAACCTGAGAGTTTCCCTTTACATGGAATGTTATATTGACTTTCTGAGTTAATTTGTCCATCAAAAGCATGTAAATCCAGGAACAGCCTTGTTTCTGTCTGTGAATATCTTTTTTTTTCTCTTTTTTGTTTTTTGAATCTGAGTCTGAGATATATGTTTTGACTGTCTTTTCAAGGTATGGTATCCACAGCTTACCTTCAATCCTGATTGTTAATCGAACTTCAAAGGCACGTTACCATGGTCAAAAGGATCTTACATCCCTCATTGAGTTTTATGAGGAATCTACAGGTAGATGGAGAGACACTTGACAGGTTAATGGGTTTGAACTATGATGCGAATGGTTATATGTCTGTGCTCTTTTATGCTTCCTGGTGCCCTTTCTCACGCGCTGTGCGTCCTAAGTTTGATATGTTGAGTTCCATGTTCCCTCTGATACAGCACTTAGCTGTCGAGCATTCTCAAGCACTCCCATCCGAATAGGCATTTGTAATAAAAAAAACAACAGAGTTCGGACATTTTTTCGATCTCTTGTTTTCCTGTCCAAAAGGTAGCAAATTTGTGGGTTTCTCGGATCTGGGATTCTCCCCAATTTGATGTTTCTGATTCTTCTTCTTCAGACATTAAATTAGGCTCTGTTCTTCGCCTTTTTTTGCTTATAATAATCATTCGGTTAGAATTTTCCGACAAAATATTCCAATTTTGCGACAAAATATTCCAACCTTGAAGTTTCCTTATGGATTTGCGGGTTCCGATTCTCTTCTTCTTATTGAGTACAATATACTTTCCTTCTGTCTTGGCTTCATCGTCCCCTGTGGACTTTTCCGTCTGCAATCACGAATTCGAGCTCTTCCGCTTCGATTTAGACTCCAAATGTCCTCCTTCTCTCCATCCAGCTCCTCCTCTCCAG

>TCONS_00072366

GTGTCAGCATCGAAGGTACTACAAGAGACATGCAACTACATAAGAAAGCTGCATAGAGAAGTTGACAATCTCAGTGACCGTTTGTCGCAGCTCCTCGACTCAGTTGATGAAGATAGCCAAGAAGCTGCCGTCATTAGAAGCTTACTTATGTAACCTTCCAATATATATGTAATATGTTGATCGTCCTTAATATACTATCAAGCGACGTGTTTTTAATGAGCTTCCGAATTTCGGTCCAGCAGTTACATTTGATTATAGTCTGATTATGTTCTGTGCTTACAAAATATCGATCATTGTGGTGTCTGAACTCGATTGTGTCAGTCAAAAGATAGAAGAAAATGCAGAATGAATAAATAATGAATAATCCATTGTACTATTTTTATTCCTAATGGATAAATTGATCTCTTATCAAAATACTTGAAAGTTTTAAAATTCATTTCTAACAAATACTCCACATGTTCTTAAAAGATCCATATTCTACATTTTTCACACATTTTAATAAAACGCACTTAATTTGTATATTTTTTTGTGTTTATCTTTTTTACAATTTTAAGTCAATAAAAGATCAATTAGTGCAATTAAAATTTTTAAAGTTTGCAATTAGTTAATAAAACATGCATTGAAAATGTAAAAAATGAATTTTTTAGAAACAAATTTTTTTTCTAGAATATCTATCTTTAAGGAACGGATGGAGTATTTATACCTTATCCCTTTAATATAAAATATTTACTAATATTTATTATTTATTGGGTATGCACCGGCTCATAATTTATTGCATTCTATAAATGCACATGACGTTGACGAAGTAATGAGGGGTATCTACATTTTAACGTCCGACAAATCTATTTGGAAAAACTTTGGTTTTGTGGGTATGAGAAATGTCCCATGACGAAATATCAGTATGCCGAGAGTAATATTACTTTGGTTTCAAAAAAAAATTAACAATCAAGTAAATTTCATCCCCGCACTTTTCACACAATAACATTGTCATCGTTAAACTTTGGTTACTTTATTTTAATGCATCAAATTTAGTAAAAAAACATCGTCATCGTCATGTCTCTTTGCTGCCACCGGTAGGTTGTTCTGTTAATGTATGAGGCTCAACTAGTCTATAAAGTTTTGCCTTTAGATTGATTGTCATAGCAATACTTCACATTGTGCAGTTTGTAGCTTTTCAGAGACAATAGAAGAATAAAAATCGATTAAAAGTGAAAAGGGATAAGAATAGAGGCTGCGATGGACTATAGTCCTGTTCGTTTGGTTGCCGCAGGTTTCGGCGGCAGCGGCGGCGGCAGCGTCAATGAAAACAGTTGTTGTTCGTTTCGCAGACGCTGCCGCTGCCGCTGACGGTGCCGCCTACTATATCGCGACCGCAGGTTTTATCGGCGTCAGCCGCAGGACGCGGCGTTCAGACGCGGCGTCAGACGCAGCTTCCTGCGTCAACGAAACGAACAAGAGTTGACGCAGAATGTTGACGCTGCCGCTGCCGCCGGTACCAGCGGCAACCAAACGAACAGCCCTTATATATGAATCTATGATACTACATTAATTCAAAGATTTGATGAGAAAGGGGTGGAGGAAAAACCTTTAAAAGAATAAAAACAAATATACTTGTTTAAGCGATTTCCAGATAAGGTTTACAATATCACAATGTTTCTCTCATATTACATCATTGTCTAGGCTTATTTCTCTCTTCTCTCTCAATCCCTCCCCTGTGGTCCTCGACCTATTTAAGTCCCTCTTATCACTTTCTCAGTTTCTTTATATCTCTAAGTGTCTTTACACAATACTTCCTTACATTTCAGTTTTCTTCTCTTTCTGCTTCCACACCTTCAACAAAATATAAAACTCGTGGAATAAACATAAACCCTCTATATCTACTGGCTATATAGACTCTTATTTTTCTTCATAAACTTAACTCCCTTATTAAGTATTATAACCTTTTTATCTCTTGCTACTCCTCCCCATCTCTTCTCTATTGTCCAATCCGGAGAAAGAGATAAAGTACGCAGCTTAAGTTTCACTTTCTTGCGACATAGTAGTGTTACAACTAAGCAACAAGTAAAATCCTACAAAGAATGTCTAACAGAAGATCAAGACAATCTTCAAGTGCTCTGAGGATCTCCGATGACCAGATGATCGACCTCGTAAGTAAGCTCCGTGTTCTTTTGCCTGAGATTCGTGAACGACGCCGTTCTGACAAG

>TCONS_00062050

CATGCATAGAGCTTGATTGTCTCTTGGGGTAGCATGGAAGAATTTGCTATCTAGAAAGTATTTTCTTGCTGCACATATGAGTCAGTTGACTTGGAATCAAAGGTGCATTTTGATGCTCATTATATTACTTGCAACAACTCACTCACCAACCTATTATATGTGAATTCGGCAGTCTACAAAACGTGATGATGTGCCCTATATGAAGTTTACTACTTACAAAGATTAATTGACTGTGCGTGAAACAGCGAAGGGAGGTGTTTTGTGGTTGAATTGTTTACTTAGTTAAAATGGGTAAGGGTCTTGATGTCCTTTTGTTTGTTTTAATGTTAAAAAAATCATTGTTTAAGTGGCGTTTCTAATTGAGTGGCCCTGAAATGCGAGGGAAGTCATTAATGTTCCGTGATCCATTGCTGAAACTCATTAATTAATCGCAGTCATGCAAAGGTATGCATGCTGCCGTCTCAAACTGTTTTTTAAGTTTGCCTTTAAACATTTTGCATTAAAAGTCTTAGAGCAATT

>TCONS_00069897

AGACCATTGATAGCTCTGTGGCCGTTTCAACGTTTCATTAGAGCTTCAGGTATAAATACCTCCTCATCCAGTCATGGTCATTACAGCTACGACAAGAATCAGAAAGTCACTTTTTTCAAGTTACGGTTATTACAATGGCGAATTCTCGAGTACTTCTTGCCGATTTGAAAGCAGGTCGCTGTTCAAACGTTGCGGAGGTCCGGCTGCTCAGGTTCTGGGAGGCCAGAAATGTTAGGAAAGGGGGAG

>TCONS_00035773

GTTGCATGTGATCGAGATCGATCGGATTCATCTCGAATCGGACTCTCAGTCTCACTGTGCGGTCTGCAAAGAGAGTTTCGAGCTGAAATCGTCGGCGAAGGAGATGCCGTGTAACCATATATATCATCCTGACTGTATCCTCCCTTTACAGTAGCTGCTGGTGATGATGATTCTGCGGCGGGATTGACGATTTGGAGGATTCGCGGTGGGGAGGATTCCGGGAGGGGATAGAGTGATGCCGGTGGTGTACACGGAGGTAGACGGCGGGAGATTGGGGGAGGAGAGGCTTACGAGGAGAGGAGGAGGGTTTGCGGGTCGGATCATGAGACTGTTTGGATGTTTTAGCGGGTCCGGGTCGAGATCGAAGGTAACTAGTAGGGTACAGCTGCGTCTTCCTCGAGGAGACGAAATTGGATAAACTAAGCCTCTCATCAA

>TCONS_00051702

ATGATCATGTAAGCGATCGAAGCATCGAACGACGCGACCGGTTGCAACAAGACGGCGATCGCGAAGCACAGCGAGTCAACTCAAACCTCTCTCCCCCCGTCTCACATGACGCTCCTCAGCTCCCACCTCAACCAGATGAAACAATCCGGCCAGATCACGATGGTGAAGAACAACTACATGAAACCAGATCCGCAAGCTCCGCCCAAGCGCGGCCGCGGCCGTCCTCCGAAGGCGAAGCCTCAGGGAGAGTCAAGCCACGTGGCGGTCCCGGCTCCTGCTCCTTCCGTTTCCTCGCCGAGGCCTAGAGGTCGTCCTCCTAAGGCGAAGGATCCTTCCTCGGAGGTGGAGACGAAGGTTGCGGCACCGAGTGGCTCCGGGAGGCCACGTGGACGACCGCCGAAGAAGCAGAAGACGGAATCCGAGGCGGTTAAAGCCGACGTTGAACCTGCGGAGGCTCCGGCTGGGGAGCGGAGAGGGCGTGGAAGGCCACCGAAAGCGAAGCCTGCGATGGTGCCTGTTGGTTGCTAAAGTTGACGCTTTAGCAAATCGAATAAGCTTTGATTAGAAGCTTAAGCAACAAAATCCTAAAAGAGAGTTCTTTTAAAAATAATTAGTATTTTTCTAAGTCGAGTTTAATGGTTAATTGTCTGTTATTTTAAATTTTAGGGGTTGAGTGGAAAAAAAAATGTCGTCTGAGAATGAGAGAATGTAATTCGGGTTTGCATGTGGTGGTGTGATGTATAAATTTCGTGTATTTCTTTACCCTTTTTCTAAAGTAACGCTTTGGTGAAAAAATGAGTATTTTATTATGAGTGATTCACGAAAGCTTGCTTGCTCAAAAAGGAGGTACAATTGAATGTTGTGACAAATTAAAGGAAAACAAGATTCAAAATTTTCGTAATTTTGTTCTACTCGGTTTAAGGCGGGAAGGAGCTAGAGTTTTTCAAAAGATTTGGGTTTATGTGAGAGTGTAATGAACAGTTGTTCGCGGTTTGATCAATGATTTTAGTCTTCCCTTTGAATAAAAGAAACAAAAACTTTGAAGAGGGGGCCAAAAATATATCTAATGTGACTTATACTGGGCCTAAAGTAATACATGTTTGAAAAATATTAATTTGGGTAACATGCTTGCACTTAATAGTTGATACTTTCTTTCAATCATCCGAAACTTCCCAATTTTTTTTAAAAAAATTTAAAAGTTAATGGAGGCGAATGCACTTTCCTCAGCTTCTTTGCTTCAGCGCCCATTCAAGCAACAGTACTCTCTCTCCTACCTCACTCCTCTCTCTCTCTCTCTCTCCCTGCAAAATCATGGCGACGGAGACCGAGCTTGAACCTCAACAATCACCAATGGCGGAGGAGCAGCAGCCGAGTCCCTTCTCTCTCCCTCCGTATCCTCAG

>TCONS_00009457

AGGTGCAAGCACTTTGAGATTGGTGGAGACAAGAAGGGAAAAGGAACATCTCTCTTTTAAGTTATTTCCTCTTATCTACTTTCTTTTTCTTGTTACTGTCTTGCTTCGATACTTGAATACATTTTGAATCTTCCAGAGCCAAGACAACCAAGAAGATTGTCTTGAGGCTCCAGTGCCAAACATGCAAGCATTTCTCTCAACACTCGATTAAGATGGTGAGGAGACTCTCTTCTCTTCTTCCCTGTTGAGTTTCTCATAGTTTAGGAGGATACTTGGTGTTTTGTAGAGCTCTTGTTTGATCCCTGAGACACTTAGAGAAAGAGTTGAAACCATTGTTAATAGATCTGTTGTTTGATTTTTTTTTCTTTTCCTTGGTCGGTTTGTTTCTGATTCTGCCTCTACATTCGTGAATTAGAGTTTATTCCGTAGTTTAGAAAATGGTTAAATCACCAAAAAATTGTAAGAGGAGACTATCAGTCTTGACGAATTGAGTAGTCTAATCGTATATTAGCAACCATTTCTTTATGATATTTTGTTATTGATTGCTATTTAAAGTATGTTCTGATGATTATTGTGACTAGCAAGACATAATTAACAAGCATACACTTTGGTTTTTAATATTTGAATAGGGATCAGTCTTGAATTTTTGAATAGTGATCTTATGATTCCTATCAATATGTTGAAAACCGTTTTCTTTACGGAACTTAACTCAAAGTTGCTGTTGTTGTATGGTGAATTGGTAGGTGAACATTCCCAAGACAAAGAAGACTTACTGCAAGAACAAGGAATGCAAGAAGCATACATTGCACAAGGTCACCCAGTACAAGAAGGGTAAAGACAGTCTTGCTGCCCAAGGTAAACGTCGTTATGACCGCAAACAATCCGGTTACGGTGGTCAGACCAAGCCCGTCTTCCACAAGAAG

>TCONS_00038001

TTGGAACAATGATTGGGATTGCCTTATAGCTTAAACTTCTCTTGGAAAACTTGTATGCTTTGTGCCTTGAAAGCTCCGCTTGAAACTTGTTGTGCTGTGTGAAATTTATATAGCCTTGAAACCAGCTCCAAATGAACCTGAACTTAATGAATCTAATCTCTCTTGGATATGGACATTTGCATACTTAGTCATGGATCTCATACACATTTGAGTTATCTTATTCCATTCATTACTTTTTTGTTAACCCAAATGGCACTCCACACCCTTAAACCCTTACCATTCTTTGAGACCAACATTGATTTGCTTGAGTGAGGCCTCTTCTTGATAGCTTGTCATGTGCAAAATCTTGAGAGTATTGGGAGCGACATAGGTTTGTTCTCATCTTTGGCTAGCAATAGGAACCTCATTTGAGCTAGCATCTAGGATGGTGGGTGAGTTTGTGTACTCTAAGATCTTTTATCTTGGGTTTGGGAAGTGAAAAAAAAA

>TCONS_00036621

GTTCCTTGCTGTGTTGCAAGTGTAATACGACCATCTGGTAGCTTAACAGGTAATGGGACAGTGTCCCGTATATCAGATAAGTAGGTAAGTGAACCGGTCATGTGGTTCGTCGCACCTGAATCAACAATCCAAGAGGAGATAGTGGACTTACCAGAAAGTGTATTGGTTGCTGGTTTGTGATCATTGAGCATTGTAACCAGCGTATTCCACTGGGTTTCAGTCAGGCCTGTAAGTCCTACTCGATCAGCGGGCGTAAGAGCATGCGCAGAGTGCTGTGGCTGAGAAGAATGTGTCACAGCATGCGCACGTGCAGTTTCTGAAGTCGTGGAGCGATTTGCAGTAGTGGTGCTTCGTCCACCATTGTTTCCAGAAGGAAGTCGTCCACGAGGCCTATCTCCCCACCATTCAGGATAACCAAGAGAGCGGAAGCAATTGTCGGCAAGATGTCCTTTACGTCCACAGCTTTTACAATGGAGTTTCATTCTCTCTTCACGAGACAGAGAAGTTCCAGAAGAGGTCGCAGGAACAGAGGTTGTTCTAACCGCGTGAGACATATGGTCGGTTTGTTCATCTAAGACCCGAGTGGTGTGTTTAGAATCTTCATCTTGTAAGAGAGCACTGTATGCGACATCCAGAGAAGGTAATGGATCTCGTGACAAGAGATTAGACTTGACTGATCCATGAAGAGATTCATCAAGACCTTTGAGAAACTCGTGAAGACGGTCTTCCTCTCTTTCTTTCTCCAAGTTCTCACCAATAGGGCAGCCACAGTTTTTAGTGATGCGGTGCTCTGCGAGAGAGGTCCAAAGTTGCATAAGTTTGCCATAGTACTGTTCGATCGATAATCCATGCTGTCTGCAAGTGGCGAGCTCAGCTTTGATACGTTGAACTCGTTGGCCATTGTTAACGGAGTACCTCCGACGAATATGTTCCCAATAATCACTAGCAATCTCAAGATGAGAGAGATTAGAGCGAACAGACTCGTTAATTGTAAGCTTAATCCAAGATAGGATAAGAGCGTTGTTGGTCCACCAATCATCAAAGTCGTGAGAATCTTG

>TCONS_00074955

AACTAGGTTAATCCGTGAGGGAGAGAGTGAGATTGACAAAGAGAATAATAGAGAACTCTTTTCCCTTGAATTCATTTGTTTATGGGAACAACCCATTTATAGAACTCTTACAGCAAGATGTCGACATTAAGGAAACAATAAACAAAGAGGTTAAGACAAAGGAGAGATGGGCAGAGTGGTCCTGGATAGTGACAGGTCCTAACGGGTGGGAAGACTTTGAATCCTTAACCAGACCCGTGACCTGCACTCTCAACGGTTCCCCACTTCCCTTAGGCGATTCCAAACATTCTTCTTGCCTTCTTCTCTTCTACATCAGTCACTCTTATGCCTTGCACTCCAAGTTAGGGTTGTCTTGTACGGACAGACTTGTCTAGCAAGGTAAGTTGAGTGATTGCTTGATCCGGATGTAATTGTTCCCCTCTTGCTTCTTACTTCATTCTCCTCTCTTTATTGCTTCATGTCAACACTCCCCCTCAAGCTTGACAGTGGTGATTGTCAAGCTTGGAAACCATGAAGCATTCCCTCATTAAAACCTTTACTTGGAAAAACCACTTGGGATAAAAAACCAAGCAAAGGAAAAAGAGTAGAATACTCCATGGTGAGGGGATTATTGACATGGGAGGTTTATGAGTCCAAGTCTTGGAAGAATGGACTCACAAACTTTGCTACTGGCCGCCTTGGTGAAGATGTCAGCCAGCTGATCCTCACTTCTTGTATAACATGGGAGAATCACTTGCTGCTCTACTGCTTGTCTTACCTTGTGGCAGTCCACTTCTATGTGTTTTGTCCTCTCATGGAAGACTGAGTTGGATGCAATGTGTATTGCGGCTTGGTTGTCACAATGCATGGTCATGGGAGTTTTGATCTCAATTCCCAAGTCGCCTAGAAGCCCTTTTATCCAGATAAGCTCACTGGTTAGCTTCCTCATTGATCTGTACTCTGCTTCTGCACTAGAGCAAGACACTACCTTCTGTTTCTTGCTCTTCCAAGTGACCAGATTTCCTCCAATGAATGTACAATAGCCGGTGGTTGATCTTCTGTCTATTCTGTCCCCAGCCCAATCTGCATCACAGTATCCTATCACCTCAGTGTTCTTGTTGCAAGCCATCCATACACCCTGACCTGGTGCCTCTCTCAAGTATCTTAGTATCCTCTCCACCATGTTCCAGTGATGAACCTTGGGAGTTTGCATATGTTGGCTTACTTGATTAACTGCAAAGCACACATCTGGCCGAGTGATGGTTAGATAGATCAGCTTCCCAACCATCCGTCTATATTGTTTGACATCTTCAAATGGAGTATCTTCATACTCCCCCTCTCGCAATACCTTGTAACCTTCTTCAAGTGGTGTCTTGGCTTGTCTTCCTCCAAGATCTCCTGCCTCCTTTAAGATGTCCAGGGTATACTTTCTTTGGGACATGAACAATCCTTCCTTTGATCTGCACATCTCAATGCCGAGGAAGTACTTCAGTTCTCCTAGGTCTTTGATATCAAATGCGGCTTTAAGAAAGGTCTTGGTTGAACTGATCCCCTCCTTGTCACAACCAGTGATGATGATATCATCCACATAGACTAGAATCACAACAATCCCTTGCTTGCTGGTGAGAGTGAAGAGTGTGTGATCCGCTTGAGACTTCACAAAGCCTCTCCCATTCAGTGTAGTGCTCAGCTTGTGATACCAAGCTCTTGGTGATTGTTTCAAACCATATATGGCTTTTCTGAGTCTGAGTACATTCCCTGGTTTCACCATCTCTTCCATTCCCGGCGGTGGTCTCATATATACCTCATCTTCTAGTTCACCTTGCAAGAAGGCATTCTTCACATCCATTTGCCAAAGATCCCACTCTTGATTTACTGCCAATGAAAGGATAATCCGGATTGTGTGAAGCTTGGCCACTGGTGCAAAGGTGTCTAGATAGTCTTCTCCATAAACTTGTGTGTATCCTCTTGCTACCAATCTGGTCTTTTTTCTTTCTGGCTTTCCATTTGCAAGATACTTGATAGTGTGTATGAGTCTGCTGGTCACGGCTTTCTTTCCTTTTGGAAGCTCTGTCTCATACCAGGTGTTGTTCCTCTCCATGGCTCCCATTTCATCATTCACTGATTCCCTCCATTCATCATCTTCCATAGCTTCTGCATATGTTCTTGGTATCCATTCCTGATCCAATGAGCCCACAAAGGCTTGATGTTCTTCTGGATAGTGAGCTAGGGAGCACACGCCACTGGGTGGGTGAGCTACTGCCTCTGCATTGAAGTAGACTCTGGTGTTGTTCCAGTTTGAAGCCGGCTTCTTGATCCTAGTACTTCTCCTAAGTACTGGCTGATCATTTTCATTCTGAGTGCCTTCTCCACCTTCCAAGTTCTGATTCTGATCCTCCACTTCCTCCTCTTGGTCATGGTGCTCTTCTTGATCATGAACTTCCTCAGATACAGGTGGTTCACCCTGATTTCCCCCCTCAGGATCAAGGGGAGCAGCTTCTTGGATCTCAACTTCATCTGTAGTTGTCTCGGTTTGCTCTTCAGACCGGCTTGATCCTCCCACATTATCTGATCCTGCTTGTCCCGGCTCTTCCATAGGTAAGCTGACCCCAAGTCTCCTCATGACTTGTCTGAGATTGGCTGCTCTATCTGATGCGGATTGAGACAAGTTCTCCAGCTCATTCCAATCCTTGTTGTTGTAGTACCCTCTGGTTTCTACAAACTTCACATCTCTAGATACCAAGACTCTCCTAGTCTCTGGTTCATAGCACTTATAGCCCTTCTGGTTTGGTGAATACCCAATGAACATAGCTCTTGTGCTCTTAGCCTCCAGTTTGTTCCTCAGTTCTCCTGGTTTCAAAACAAAACAAAGGCACCCAAACACACGCATATGATCAATAGATGGTTTAGCTTTGTTCAATACCTCAAATGGAGAGCAGTCTTGTAGAACTCTGGTAGGAATGCGGTTGATCAGGTAGCATGCAGTTAGGACCGCATCACCCCAAAAGCTCTTTGGGACACTAGTATGGAACATCATGCTCCTAGTGACCTCCATGAGATGCCTGTTCTTCCTCTCGGCCACACCATTTTGTTGGGGAGTGTATGGACAGCTGGTTTGATGAATCACACCTTGTTTGGCTAGATGAGCTTTGAATGCATTGCTGGTGTACTCTCCTCCATTATCTGATCTCAAAATCTTAACTTTGGCATTATAATGGTTAGAGACATATGTTTCAAAGTTTATAAATGCATCTAAGACCCTATCCTTTGATTGTAAGAGAGTGATCCATGTGTACTTGGATTTCTCATCAATAAAGGTCACAAAATACTTGTGATGTTCTCTAGAAAGACATGGAGCTGTCCATACATCCGAGTGGATTAAATCAAAGCAGTGTTCATAGATTGTACTAGACTTGGGAAATACAGTTTTGCAATGTTTTCCTAAGATACAAGCTTCACAATCTTGCTGGAATGAAATACTAGGCAACATGATGTTCAAGGCACGAGAATGAGGATGTCCTAATCTAGCATGCCACATTACATCTTTAGGGAAATTAAAACTTGAATTGAAAGCAACAGATAGATCGGATGCAAGCTTAGTGTCTTCTAGCAAGTACAGGTTTCCCTTGCTCACACCTTTGCCAAGCAACCTACATGAGTCAATATCCTGAAAGTAGACATCATCAGGCGTAAATATAACATTGCAATTCAAGTCATTGGTCACCCTTTTCACAGATAATAGATTAGAAGTGAAACTAGGCATGTAGAAAGCTTTGGATTCTTTCTCAAATAATTTAAGTTCCCCTATGCCTTTAATTGGAACCTTGTTACCATCAGCTATCATAACATTTCCTACTGCAGGAGTGATATTTTTAATCAAATTTAAATCACTAATCATGTGATGAGAGGCTCCTGAGTCTATGACTAAGGGTTTAACTCTATCAGATTCATGAGCAACTTGAAACGCCGTGAATAAACTCCTAGGCAATTGATATGATGCACTAAGGGAGGAACCAAGAGTTTTACCAGACTCCTTGAGAGCTTTGATGAGTGCCTCAATGTCAGATTTCTTGATGGACTCATCCTGGTTTCCCTTGCCCTCATGAGTGCGGTAGGCCGTGGAAGCTTCTCCTACATCACCTGAGTAGTTTGCCCGACCATCAGTCTTGAACTTAGCCGGTTTGAGGTGTGGGTGAAGTATCCAGCACTTGTCCTTGCCGTGACCCTTCCTCTTGCAATGATCACACACCCACACTTTCTTTTCCTCCGGCTTGTAGTACCCTTTGTTGGCTACTCCGGCAGGTTTGCTCTCGGCTCCTTCAGCTTGATTGGCCATCACTAGCTCTCCCTTTCTCTTAAAGAGACCCACTGAGCCGTGCTCCTTCTGAATCTCAGCACACACTTCATCTAGAGATGGAAGCTCCTTGTTCCTCAGTATGTGCCTAATAAGGTCATCATACCCTGGGTTGAGAGTGAGCAGCAAGGCAAAGACCTTATCTTGCTCCCTTCTTTCATGTAGAACATCCGGATCAACTGTTGCGGGTCTGAGCATCTCTAGCTCAGCCCATAGAGACCGGAACTTCCCAAAATGAGCATCAAAGTCTGTGTCCTCTTGACTTAGAGAATTGATTGCTCCTTTTACTTCATACACCTTGCTGATGTTGGATTTGTTGGCATACACCTTCTTGAGAGTATCCCACAGCTCCTTGGAAGTTTCACAGAATGCATAAGCCTCTTGGAGAGAAGGGGCGAGGCTATTCTGGATGATAGACAACACCATGAGGTCCTCCTGACCTTTCTTCTTATCCCCAGCATCCGCAACCACCACTTCTTTCTGAGATTGGCTGCTCTATCTGGTTTGATTGGAACGACGATGAAGCTGTGCTATTCCCAAACTGGGAAACTTGAATCACCTGATTAGAAAGTGGGATAACTTTTTCATCCCAACTCCTATGAGATTTATTCAATTTCCTGGTGTTTCTCCACCATTTTATGTATCCAAATCAAGCTTCTTACATAGTGATTCATCCTGGTGTTATTGGAACGACGAAGAAGCTGTCCTATTCCTAAACTGGGAAACTGGAATCACCTGATTTGAAAGTGGGATAACTTCTTCATCCCAACTCTTATGAGATTTATTCAACTTCCTGGTGATTCTCCAACACTTTATGTATCCAAGTCAAGCTTCTTACAAAGTGATTCATCCTGGTTCGATTGGAACGACGGAGAAGTTGTCCTATTCCCAAACTGGGAAACTTGAATCACCTGATTAGAAAGTGGGATAACTTCTTCATTCCAACTCCTATGAGATTTATTCAACTTCCTGGTGATTCTCCAACACTTTATGTATCCAAGTCAAGCTTCTTACAAAGTGATTCATCCTGGTGTTATTGGAACGACGAAGAAGCTGTCCTATTCCCAAACTGGGAAACTGGAATCACCTGATTAGAAAGTGGGATAACTTCTTCTTTGAGAGTTTTATAGGATTTATGTGAGAGTTTAAGGAAGATTTGTGAAGAGATTAGAGAAGAACTAGGTTGAACCGTGAGAGAGAGAGAGAGATTGACAAAGAGAATAATAGAGAACTCTTTTCCCTTGAATGATTTGTTTATGGGAACAACCCATTTATAGAATTCATACAGCAAGATGTCGACATTAAGGAAACAATAAACAAAGAGGTTAAGACAAAGGAGAGATGGGCAGAGTGGTCCTGGATAGTGACAGGTCCTAACGGGTGGGAAGACTTTGAATCCTTAACCAGACCCGTGACCTGCACTCTCAACGGTTCCCCACTTCCCTTAGGCGATTCCAAACATTCTTCTTGCCTTCTTCTCTTCTACATCAGTCACTCTTATGCCTTGCACTCCAAGTTAGGGTTGTCTTGTACGGACAGACTTGTCTTGCAAGGTAAGTTGAGTGATTGCTTGATCCGGATGTAATTGTTCCCCTCTTGCTTCTTACTTCATTCTCCTCTCTTTATTGCTTC

>TCONS_00055220

AGAAAAGCCAAAAGCTTTCCTCTCGCTAAAACCCTACCGCCGCGACCACCGACACCGAGAGACTCCGCCGCCGTTAAACCCACCTCCGTTAACCGTTCGACTTGAATGACGAGGAGAGAACCTGATGATAATCAATGAGATGGACTTATCAGACGCGGTGATTGTGAAATCTAGTAAACTTAAATCAGCTGTGTGGAACGATTTCGACAAGGTGAGAAAAGGAGAGATACACGTGGCAATCTGCAGACACTGCAAGAAACGTCTTAGCGGTTCGAGCGCGAGTGGGACTTCTCATTTGAGGAACCATTTGATTAGATGTAAGAGGAGAGCTAGCTGTAACGGTGTTGGTGCTGCGAAGAGAGTAGAAACAACACCACTTGAGTTAGCGATTGTCGACCAGACAAAACATGAACATAGTGATGCTTCTGTAATAAACTCCGGTTTGGATCAGAGGAGGAGCCGGTTTGATCTAGCCAGGATGATAATCTTACACGGTTATCCGTTAACCATGGTTGAAGACGTTGGGTTCAGAGTGTTCCTTAGGAATTTACAGCCTTTGTTTGAGCCCGTTGTGTTTGAGAGAGTTGAGTCTGATTGTATGGAGATATACGCTAAGGAGA

>TCONS_00038136

AACAAAGATAGCTATTGTTGTTCATCGTTCAGAAGCCCGTTCGTAAAAAGTTTCCTTTAATTTTTATTCATTCTCTCTTACTTTACTTTTATTTTATATTTTATTTTAGCTTATTCAACTTCTGTATTAAATATGATTAATTTAAATATGATTAATATTAATAAAGTAGGAAAAAAGGATAATATGATAAAAAAAATGAAACCCCAGTCTTACGTTTCCACATCAAAGTGAAATAGAGAACTTCATTCTCTTTTTTTTTCATTTCATGCCTATTGGCGTTCCAAAAGTACCTTTTCGAAGTCCTGGAGAAGGAGATACATCTTGGGTTGACTTT

>TCONS_00043960

GTACGGAATTTTAGGAAAGAAGTTTAAAGATTCGTTCGGACATGTAGGGAGTTCCATCTTGGGAGGTCTCGTGGGTATGAAGAAACCTCAAAATCATGGAGTCCCATATTCTATAACCGAAGATTTCACCAGCGTCTATCGAATGCACTCGCTCTTACCTGATCAGCTCGAACTACGTGACATCGACGTTGTACCAGGAACTAATAAATCACTACCATTGATTGAAGAGTATATAACTTACTTTAGAGCTCTCTATTTTTGAAACTTCCAAATCAAATTTTTTTGGAAGTTAAGGTTTTTCTTTTAATTTCTCTCTTGTTTTTTTTTTTCTTATATAACTTGATTATATGGATTTTAGGGTTTCTTTCGGAAAATTGCTTGGTCCTAAGGGAGAACAAACCATGTCTCATATTGGATTCACTAAGCTAATGGTCTCAATGGGTCATCAAGCAAGTGGGGCTCTTGAACTGATGAATTATCCATTGTGGCTTAGGGATCTTGTTCCCCATGACCCCAATGGCTACGATCGTCCGGACCACATCGACTTGGCTGCTTTAGAAAGTAAGTCAATCCGATCCAAGCCGTTCGTTTTGTGTTTGTATTTATATATGTACAGTATTGTGGTTTGACTTGATGAGTGATGTGTTCTTCGTCATTTGATATAGTCTATAGGGACAGGGAGAGGAATGTTGCACGGTACAATGAATTTAGGAGATCTATGTTTATGATTCCGATAAAGAAGTGGGAAGATCTAACGGACGATAAGGAAGCAATTGAAGCACTAGAAGACGTGTACGGTGGTAATGTGGACGAGCTCGATCTTCTGGTGGGACTTATGGCCGAGAAGAAAATCAAAGGATTCGCTATTAGCGAGACTGCCTTTAACATTTTCGTCCTCATGGCTACAAGGTATGTATATTTACTTTAGCATTTTGTTTGTATAATTTTATTTAAATTTCTCAGGATAAAAAAATGGACAGCTGAATTTAAATTTCATGGATGGACAATATTAATATGTTATATATCAAATTAAATATATTTTCAGGAGATTAGAAGCGGATAGATTCTTCACGAGTGATTTCAACGAAATGACTTATACAAAGAAAGGACTTGAATGGGTGAATACTACAGAGAATCTCAAAGAGGTTTTCGATCGCCATTATCCTGAAATGACCGAGAGATGGATGAACTCTGAAAGTGCATTTTCAGTATGGGATTCACCACCGGTTGCCAAAAACCCAATCCCTCTGTATCTCCGAGTACCACCATCTTAAATCAGATCGATTATGTCAGATGTGACGCACTTTGAATGATTTGAATTTTATTTTATTGGTATTGTTGGTTACTGGGAGCTGCGAATGTGTGTTGCTGGGAGCTCTCAGCATTATTCATCTACTACAATTATGATGAAAGTAATAAGTTCTTTAATCTCTTCTGTTTTTCTCAAATTCATCCACAAAGACTTCCACGAGGTTTACTCAAGAATGCCCGTCTTGGATCGTATCATACTTCTTGTGAGTAATTAATTTTTCTTTTTGTAAACTAAAATCAAAAATTATTTCACAAAGGAAAGTATTTCTTTCATAAACGGATTATGTTACCATTAACTAAGTGTCAGAAAACTCTATGCAAAATTCTTTGTAATAAATAATATTCTCTTTCCGGAGGTTTTATGTACCATATTTTCACCATTAAGCTTGGATGGTCTATAATGTTTCTTATATACACATTCATTCATTCGAAAAATAGTTATGATGATTGGATATTACATGGTCATGCAGATCGTGCATGCAGTGGATAAGATGGTTCCGTGGCACAAGCTTCCGGTGTTCTTGGGTATGGCCTACCTAGGGTTGCGCAGACATCTTCACCAAGAATACAATCTCATCAATGTTGGTCAAACTCCGGTTGGGACCCGGTTTAATCCTGCTGACTATCCGTACCGGACTGCTGACGGAAAATTTAATGATCCGTTCAACGAAGGCGTCGGCAGCCAATATAGCTTCATCGGAAGAAATTGTCCTCCTGTTGATCAGAAGACCAAGGTAAGATTGCCTACATGCATATTTGTAGGTATAAAAAAGTCTTTAACACGGCTTCAGATTTGATCTTGGTTCGAATTTTGGGTATGAAGATTTATTTGTCAGTTTGTAAACGTATATAATAGACTAATTTGAATAACTTTTGCCCGACTTTTTTATATGGAGATCGATTGAGGATACAAATAATTTCGCTTTTTTCTTCAGTTGAATCGAACTTCCGAATAAGGAATGACTTGCTAATATTACTGATCTGAAACGTTATTATAAAAGTGACATCTTCTTGAGAATTTTCTATATTGTAGTATGTTTTCCAGTTTTCCAAGTCTTGAATATTACGTCTTCTTTTTTTTTAATTTAGAATATTACTTGAAAACTATAAAGCGATGTGTTTTTTTTTTCAAGTTGCTGAAGCCAGACCCCATGGTAGTGGCGACAAAACTATTAGCAAGAAGAAAGTTGATCGACACGGGAAAACAGTTTAATATGATTGCAGCTTCTTGGATACAATTCATGATTCATGACTGGGTTGATCATCTTGAAGAAACTAATCAGGTTCGTTAATTAATCTTCCATATCCCACATTTATACGATGATCAGTCATGGTTTAGGTGATGATTGTTTTCCTAATTTTTAGTTTTTGATTTTGGATTTTAGTGTTTGATTTTTGGTTTTTAATTTTTCAGTATTTGATTTTGGATTTTAATGTTTGATTTTTGGTTTTTAATTTTTCAGTAGATTTTTGTTTTTGAACAACATAAATGGTTTGTTTTAGATTTTGGTTTTTAGTTTTCGTTTTTTGCTTTTAAAATTAATAAAATGAAAATTATTTTTAATGGTAAAATGTTTATCTGAAAATTAAAAAATATATATATTGTCATCAAAACACACATAAATTTATTTAATAAAATTATTTTTAAACAAAAACCGTAAATACAAAATAAAAAATTAATATCATACATTTTATGAAAAATATTGCCAAGATAATTTTTACAAATTTATTCTATGCTTATTTCTTTTCTGTATAGATAATTTCTATAACAATGAATTAAAATAGTAACTTTTAAATAAAATTTCTTTTTGAAATATTTTTTGAACTTCTTTTAGTTTACAAGCACAAGAACTTTGTTCTTATATATAAATTTTCATTTTGTTAAATGCTGATTAAGGGAGTAACAATATCTACTTAACAAAGAATAAAAAAATATTGAAAAATTAACGTGATGTTTGTGAAGAAGCTGCTTGACCAAAGTAAAGTGGTGGTTGGCTTTTTTTAGAAAGTTGCCGTTTATTTAAAAAAAAAAACAGTTTTCCTAGATTATAAGGAATTTATTTGTCCAAAAACTTGATTGATTAAAAAGTGGTTTTTTGAAAACAAAAACTAAAAACCACTGCAAAATATGAAAGCAATCATCCTCTTAATCTTGTTATTAATGGGTAAACTTAGATTGAGCTCGTGGCTCCAAAAGAAGTAGCGAACGAGTGTCCCTTAAGCTCCTTCAGATTCTTCAAGACAAAGGAAGTTCCTACTGGTTTCTTCGAGATCAAGACCGGTTCGCTTAATTCCCGTACACCCTGGTGGTAAGTTATGCTATATTCTATAACTTGTAAAGTAAAACACCCTTATTAGTACTGGTAGTTAATTCTTAGCCTAGCTTGTATTGAGATCGTACATACTAGCACCTTAGTGTCAAGTAATTGATTCTCAATCTAACATATACATTACATTACCTATATTCTATTCCCAATTAAGTACACCTTAGTGCTAGTTAAGTAAGTCTAACATGTACATAGATCACAAGTGAATTATTGGGTCTACACTAAGCCACCAAAGAATCAAATGTCAGAATAAACATGAATACGTGTGGGTATTATGAAATTAGGGATTCGAGCGCCATCTATGGAAGCAACTCAAAAGCAATGGACAGAGTGAGAACTTACAAAGACGGGAAACTAAAGTTATCGGAGGAGACGGGTCTCTTACTCCAAGACCAAGACGGTTTAGCCATTTCTGGCGACGTACGTAACAGTTGGGTCGGTGTCTCCGCCTTGCAAGCTCTCTTCATCAAAGAGCACAACGCCGTATGCGACGCCCTCAAGGTAAATATATGTTTTCATTTTTTGTGTGTAAATTCGGACCTTTTTTCATACGCAAATAGAGTGAATGATTGTATATAAACTAGTTCAGTATTATATTTTTATGCATCATATAGAAGGAGTACGATGATTTGGAAGACGAGGATTTGTACCGCCATGCTAGGCTAGTGACGTCAGCAGTGATTGCCAAGATTCACACCATAGATTGGACCGTTGAGCTTCTTAAAACCGACACTTTACTTGCCGGGATGCGAACAAATTG

>TCONS_00059858

ATGATGGGTAAAGTAAACCAAGAGAGGCTAGCTGAGTACCTCGGGGAGTTGAAACAGAAGCAGGATACCAACGACAGATACGTGGCTGCTCTAAGGGGGGAGACTCTTACCAGGAAGCCTTACCAAAGAATTCAACCAGTCCCAAAGCCAAATGACACGGTCACAACCAAACCTCAGTAATGAATTATCACTTCTTAGAAAGAGCTACTGTAATGGATGCCAAGATAGGCATGTCTATTGGCCGTTCATGTTTTATTTTATTTTTTTGTTTGAATCTGGCGTGCTTTGTGGTTATCAAACTGGTGCAAGATTCAGTCACTTCAAACATTTTTTGATAGACAGAGAATAAAAATGTAAAACACATTACACATGTTTTTGTACATTGCTTCGTCAATCTTGTTCGTTCGCAAGCTAACATATATAGTACATAGGAAATTAATAGGTACAATCTGATTATAGAACAATGTCAGAGACACTTGGTAAACCTGAGACAGTATGTGCAAGGGTTTTGTTATGTGCAAAGGCACAGAGAGATCACGTGTAAAGCCTTAGCCGGCCGGTGAGTAGAAACGGTGACTCGGAGGGAGAGAACGATGTCGTTTTTGTATGAGAAGAGCGATACATGGAGATGGCTAGTGAGGAAGACGAGAGATTCGAGATCCTTCTTCTTCACGTTCGCTACCGTATGCGGTGTTATCCCTGGGATAATCGGCTATGGGGTGATGCAGGCCACCAATTCCACTAACCCGGAGCTCGAGGCCCGGCTCCGACAATCCGCCCGACCCGAAACCGTT

>TCONS_00074401

CACTGATGAAAAGGACGTAAAGATGAAAAGGATGTGCATTGTAGGGTCTTTAGTTCATGTCGGGTAGTATGGGATTCTGTGTAATAGATCTTATTAGCTCATGACTTGTGATTGTTTGCTCTTCCTGAGTTGAGCTCGTGGGTTCCTAGAACCTACCCTCACTGAGTATTATGTACTCACCCCTCATTTTTACAGGTATGGCCGAGAGGGAGCGGGAGTAGATGTCCGTATGGTGCAAGTGTTTTC

>TCONS_00026660

AAATTTTTGGCTGGTAGTTCATCCTCTGTTGCGACATTTGGTGGTTTGGTTTAAGCAACAGAAACTGCAATGTTCATGTTGAACGGATACGAGAGCAAATGTTTTATGGACAAAACTGTGTTTCCAGTTGCTGTTCCTTCATTGCAAGGATACTAATCTGAATTTTACGGTGACAATTGTTTACTTGTAAACATATCTTCAGCTATACAATTCTGGTCGAAATTGAAAAGCAAACCAGTTTGTTAAGTGATCATAAACCACAGATTCTAAATCATAAACTTTACAGCTTGAGGAAAGACCTAAAGAGGGTATATTTGTTTTATACATCAATATGGCTGGAGTTATGCAGAAGTTCCTTGTTGCATCAATGTTCATGTGGATGCTTCCTATTGCCATCTTATATGCTTTCAACCACGATTTTCTTCCTGGTATTTTGACTTTGGAACCTAGTTATAATTACTTACTTCGGTTTGATTTTTATACAAATCCTTTGATGGGTTTCTTAGATTAGGACCAGTTGTTGTCTTAAAGCAAAAAGTTTCCGCCTTTCCGGGTTTTGATTGATGTATGAGGGAATTTCTCTGTGTGTCGTTTCGTGCTTTACTTAAAGCTTGGAGCTTGGAGGTTTTTGAGCTGAGGAAAAGTAATTTAGAACTTGGAGCTCTTGATCTGAATCCTGTGAGAAGCCATTATTAGATTAGAAACTGTAGCTATGTGTTCAGATTTAGATTGTGGTACGTTTGCATTATTGATTGTGAGTTATCTCTGGATCTTTGTTTGACTAAATGGCTTTGATTAAGGGTATACTTTTGAGGAGTATGATGATTTTCTAGAAACTAGAGGGCATTGTTTTGCTAATTACTTATCCACCTTTGTATTGTCTTTACATTTGCTATTTAAGTCTGTCGTACAAATATGATCTAAAGATCTATATAATTCCTGATCATAAGTTTAGTCTTGTTGATTCATGAGTTATTGGTTTTTGGCTTGTGGTAGGAGGTTTAACATTTTAAGCATTTCAAACTGAGTTTAACCCGGAGAACCACTATCTGAAGAATTTAGGAAAGTAGGAACTACTCTTGTTCTCTTCATTATTTTCTTTGCAAAAGTATCTTTAGCCTGAAAACACTCTTTTAGTGAAACCTGATTTAGGAAAATATCTGATTGTTGGGATCTGACGAGATTACAATATCTGTTCTGTTAAAAAAAAATCATTTAGTAACTGCTAAATTTTATTAGAAGTTAAGTTTTAGGTATCGTTGAAAAGAGTTGGAGCTGGAAACATGAGATTGAAATGCTACAAGAGGCTATGTGCTATGGTTGTAACCTTAGTTCTTGTAATAAAGGCATTTTAGTCTTTGACGCTTAGTTCATGTAATGTACTTACGGTGTAATGTCTCAATCTCTCTCACTGTCTCACGAAGTCGTCTCTATTATTGTCGCAGCTTTAGTTTTACAATAACATAGGTGTCTCAGAGTTATTACCGCCTCTTACAGTTGCGCTGTTTAAATCTTGGACAGGTTCAACAACATTGTCTCCACATTCTCTTACACTACTGAGTGGATTTCTTGCGGCGGTATCAGTCAATGTAGTGATTGTGTTCTACATCTGTCTGGCCCTGAAAGAACCTACGGATAAACACAAGCCAGACGCTTCGTTTGTCGCAGAGGCCAAGGATAGCGTGAAGAAACTGACATCAGGAGTCCCAAGCACTGACCCGGCACTCAAGAAACAAGACTTTTGAAGAAAGCAGCATGAAACGACACAAGTCTGATTCCTCTTTCTCTTCCAAGGTCTTCAAAAATCAGAATTCATTTGTGGCCATGTAAGCTTCCTAACATTCTTGGTGCCGCTTACTAATGATCTTTGGAATTTTGTGGTTTAACAGGAGAGGAAGGACAAGGTAGGAGAACGTGTTTCAGCTCTTCAACAGCTAGTTTCCCCTTATGGAAAGGTACTACTATTAGATGGAAACTTCTTTTTCAAGCAACTGACATGATCTCTCTTACAGACCGACACTGCATCAGTTCTTCTAGAGACGATGCAATACATTCAGTTTCTTCAAGAACAAGTCAAGGTATGCTTCTTGTTTTCGAATCAAAATATGTCTCTTTAAATCCAGCATTGACTTTGCTTAACTGTTTGTATAGGTTCTAAGCGCTCCATATCTTCAAACATCGCCCACTATTACGCAGGTGAGCAGAATCTTGACGCTAACTTCAACTATCTGTGATGTGGACTCAAACATATCCTGCTTGTTTCTTGTAACCAGGAAGAGATGGTGGATCACAGTTTGAGAAGCAGAGGGTTATGTCTTGTCCCAATGGAGTATACGTTAGGGGTTGCTCAAAGCAACGGTGCTGATATATGGGCTCCTGTGAAGACTCCAACATCTCCTGCTTTTCAATGACTTGACTCCACCAGCTGCCAAAAATCGGTGTGCCTCACGAGTCAAGACTTAATAAAAAATAACCCAAAACGTTAATTAGTCTGAGGATGTCTATGTAACACAGACAGTGATGGCTGTTTCTGTTATTTTTTATGGTTAATCTCCAAACTACGTGATGGGCTTGATAAAGCCCATTACTTTGATTTGTATAATTTGGTCTAATACAATTTCGTAATAATTGATTGATTCGACGCAAAGTTTTAACTAAAGGGCGACTGGTTTTCCGCTACCATCCGCAAACGCAGTTTTTGCGGTCGCTAGCGGTTATCGGCGGTTTGCAACAATCGCTTAAATCGTTCTAAACTGCTTCAAACCGCTCTGAATCTCTTAAATTTAAACGCTGGTTTCAGCTAGCGTTTGCGGTTGCGGGCGGTTGCAGGAAGGTAAAATTTTTTTCCTTTTTTTAAAAACAATATATATACAAAAGAAAAAATATTTAATAAAAAAATTAAAATTGAAATTATGAAAATATTAAAATATATCTATTATATTTTAATTAATATTATAAAATTCTGTTACCGCATTTGGTAGTTAACCAGTCATAAGTCACCCGCAAACGCACCAATTTTTAACCGCAGTACCAGTCGTACAAATCTCTTAAAACTGCTAGAAACCGCAACCGTCCACATCCACAAACTCCCACAACCACAACCGCAACCGCTGCATTTGAACCAGTCAGCCCTAACTTCCTGTGAAATGAGCTTGTGCTTGGGTGGGACTAAACCGGTTTATTTCGGTTTAGAGTATTGGTCACTCTGGTTAACAATGGCTTCGTTGGGATTTGTATTGACAACAACAAAAAAAATCTAGACTTTGTTAAAAATTGCAATCAGAAACCTTTGAATCCAAGACCTGAGTTAACTCGCCAGATTCAAC

>TCONS_00030322

GCCCAAACAGAAGTATATTAACCACAGAAACTCCATACAAAGGCCCAAGCCAAAGAAGAGCCCAAACCAAAAAACCAAACAGAAGACAAAACACGTGTTTGAACCTCAGCCATCAAGTAAACACGCGTCGTCATGCTCCATCCCTATCACCGCTTCGGGAATCAACACCGGAGAAACATCCGATGATTCACCGGAACCACCGTAACCCACCGGAGTCAACTCGAAAACACGAGTCTCCACTGGTTTTTTCGACTTCAAGCTTCATCACCGAAACAAACACCATGCAAATTCACCATCAGAACCACCGGGAATTCAACCATAACCACCGAAAGAGCCACCTCCAACGACTGTATTGAAAGGTATAGGCTTCATCGGCTACAACACATACCTTGAGCTTTGGAGAGCATCAATCGATAAGAATCGAGATCTCATCCAAACTCATACTCTCTGTTTCGAATCAACCATCCTTCACTCAAGATCAAATCTCCAATCGAACTTCGACTGTCTGTCCGGCGAGGAGATCAATCGATGAAGGGATAGACAAAACGCCAATCTTGCGGAGATCCATCAAACCGCCAAGAAGTACGCCGAAGCTGGATGATTCCGTCTTGAAGATCACGAACCACCAGGGTCAAAACCAGCTACACCTCGTCAAGGAGACAACGATGTACCAGAGACGATGTTACAAGCCAAGAAGAGGAAGAAAAG

>TCONS_00016965

GAAAGATAGAACACTAAGAATCTCATGGCTCTCAATCCCAGATAGATTCTACTTGTACCATATCCTTTAGAACAGAGTGATTCCATTCACTATATAAACCCAAATCTAGTGAAGCAGACTAAAGACCCTGTTCGTATAAGGAAAAAAGTAGCGACCCAGAACTATAATTAAATTCATTTGAACCAACATCAAATCTAACAATCATAATTAGCTTCGTCGGACAGTCAGGCCGGCTCCCACCCACCCCCTCCTTCGGCGTCTGAGCTTACTGACGAAGTAGTTGGCATCCGGTGACGTTTCAGTGGTGCTTCTCTTCTCCTTTTTCCGATACCGTTTTTCAATCCATGGCGCGGCGGATCCCTAGGAGTGCCTCATGGGTCCTGGCGGCTCGTTGGTCGAAGGTGTTGCTCTGCTAGAGGACGGTGGAGCCCCGGCGAATAATGAAAATCAGTCATTGGAATTTCTAGGGTTTGTATCGATCTGTTGGGAGGGGCTCGTTTGACGGCGGATCTACCACGGAGAAGGCGGTTCTCCAGGTTCGTTTACTTTCCGACATAGTTACTTCTCGAACGGTGAGTGTAGGAGGAGGATCATGTGATTGTTCGGACCTATGAGTGGTGGAGCCTCTTCGCAGTCAATATGGAGAGATAGGGAGTCGGTGTTCTTTGCGAATCCGACGTTATGTGGCCGCAGGACCCCCTCCGAGACGGCGGTGCTTTGAAAGTACACCGGCGACAGTTCTATCGGCTGGCATGGACACCCCGTCGTAGTGATGACCAGATAACTGAATCTCCATGCCGTCGGTGTTCATCGGAGGTCGAGGCGTAGCCTCCTTCGTGTTCTTCCCCATGTTCCAAATCTTTTTGCTTGAAATCTGTTGTTTGTTGTGTTTCATCTACTTCCTTGTAACCTAGCAGTCTCTGTTTTAC

>TCONS_00018265

CACCTCCACAAGCTTTCCGTAGTTCTTGCTGTCTCAAAGCCCGCCGATAGCTCTTAGAGTGCTTAGTAATCCACACTGAGTCCTCCTCTTTAGACTCAAAGTCACCTTCCTGACTGTTCTTCGAACTGTTATTTTTGACCTCTTCTTCAACGTCTTCAATCTCCCCCTCCTCTAAAGAGTTCTCTCCCGCAATTACTGGTTTCTCCACTATAACTGACTCTAATTCCGAATTCAGAACAATCATCGCTGACTTCTTACTCGCTTCCAAAGACCCTATCACCTCCTCCACCACCAGAGTTTCTCTATCCACCACTCTAGCTCCTTCAGTAGGAGAGCTAAGAGCCCTTGCTCTAGCTCTCGCTCTCGACTGAGACCTCCTACGCGACCTTGAACGCGCTCTCAAACTCACTCCAATACACTCCTCCTCTTTCCTTTTTTGATCGTCTCCCTGATACGCTTCTCCCAAAGAAACTTCTTCCCCAGACTTCACCACACTGACGACCTTCTCTTTCTTCTGCACCTGAGCTTTCTTCACCAAGGGTTTATGGCATCGATTCATCAAATGACCATACTTCCCACAGTTTATACACTTCGGCGGGAGATTAGGGTACTCAACATTGATCCTAACCGAATTACCTTGAGTATCTCTAACCTCTACTACCTCAGGAGGAGTTTGTGACAGATCAATTTCCACCTTAACTTTCGTATCACCGAAGTGGTAGGGATCTAGCCG

>TCONS_00029442

CTTTTCTCTCTATCTCACTTCATCTAAAAAAAAATACATCTTTCTTTTCTCTCTGCACATTGGTTCATCCATGGCGTAACGTCCACCATCTCTCCCATTCGCCACCGTCTTCTCTCACCACCACCAGTCACCTGCAAATCACATTCGTGACTTCACCGAACCGGGGCGTCCAAGACCAGCCAAGCTCTGTCAAGAGAATCCTCAGAGATTTCGCCACCACCGGATAGTCCCGCGGCCTCAAATCACCGGAAATCGAAAAGATGTCGGTTCCCGAAACCCTAACCTCTGTATCTCGCTTTGCAGAGTCGCCGCTCAAAGTCCGAGGAAGAACACGCCTTCCACAGCTCCGGTCCATGAGAAGAATCAACGCCGTCGGAGACAAAGCCTAGAACCCCATCATCCTCGAACGGAGGCAACCACTATCGACCGACGAGACACCAAGACGCTGGATCTTTCGTAGGGAACCATCGCTCAAGGCCCACCCGACGCACGGAGACTGTTAGTTTTCACCGAATCTTGTCTGCTCCGTCAGGTTGACCGGATTCCGAGAAGCAAGGGGGGGGGGGCATCTACGCAGTGGTGGTCATTGGGTTTGCGGCTATGAGGTGATGGAGAAGAGATAAGAGGTGGTGGTCATGGGGCTTGTGGCGAGGAGGCAGGCCCAAGAAGATTAGGTCTTGTATTAGGTCTAATTAAGATTAGG

>TCONS_00069443

GAGAACAAGAAGAAGGCCAATCATGGAGATGGCTGGCATGATTGTGTCCTTCCTGTACATAGCCAAGTAGTGAAGATCAAGAAAGAGTTTGAGAAGATTCATCAGCCGCAGATGCTGAGGGTGCTTCGCTATATCACAGGTCCACAGCGTTCCCGTTCTCCTCTCGGGTTAGGGCAGAGAGACCGCAGACCTATCACCGTTGGAAAATTGATAACAAAGAAAGAGCAATGGAGATAGGTTACTATCTGTGTATTTTAGGCTCTCTTTTGTCTTTCAATGTAAATATCTTTAGTTAATGTCATGAGTCTCCTATGTCAACAAATCTTTTCCTTCTCTGGGATAAACCACAACTAATTCATAGAGAAGGGCTCAGATAAGCTTAAACAGAGGTTTTGAAACAAGCAAAAAGACTAGATAACTTATCATTATCACGAAGCATTAGGTGTCTCCTGAGCTGATAGTAACTTAACCCTTCTTCTGAGGTCTTCGATGACACCAGAGAACACTGATTTTTTC

>TCONS_00061181

CAGATTTTACGTTTTTGACAGAATTACAAAGGATAACAAGAATCAGAGTAAACGAAATTAAAAACTAAACACAACTCTATCTAGCATCAACAGGGGCAGGCACAACAAACCATGGCCGCTATCCAGAGGCTCGACCAAATCCACACCACGTGCTTGCCGCAAAAAGATAAGACAAGTTCAACCGAGAGCCGGAACCCGGTAATTTTGGCTTTCCCGATGATCCGCTCTTAAAGATAATGACCCATCTGAGAACAGCTTGCCGAGGGCTTCATAGCACCTGCCTACACGAACAGACAACAACAGTAGCGGCCAAGCACACCCGCCTCAACTAAATCGAGCATTTATTAGACCGGAGACGAGCAGAGAACACATAGTACAATGTACAGCGCCCCTAAACACCAAACGGCCAAAGAACGGGTTGAAATTGTCACTGAACTCTGACCAAACAGTCTCTGCACCTGCACAAACTAGCTAATCACACTACCAAAGCTGAGCAAATGAACAGCTTAGGCACTGGGATGAGTGATATCGCCAAGCATTGACTCCGAGACTAAGGACGGGTCCTCAGCCTCACAATACTTGCTGCCACACGAGAACCGTACGACAGTAGCATGAGACAAGAGAAGCTAATCTCCAGACAAATGGAGAGGAGCAAACATGACAAACCAAGAGACACCTCTGAGAAGCCAGACTGCCGTCGACCCCGGAGGTTGTGGAGAGAGACAAAGCAGGGATATTCGAAGTGATGTGGGCAGCGAACAGCCGCGGAGAAGCCATAGACGACAACCCTTTAAGAAGCTCACGAACCCACACGAGCCAGGCCACTGAGC

>TCONS_00074234

CTCAAGTCATAAGCTTGGGTAATATTTGCTTGCTGGATGGATCTTGGACAACATCTGCCAACTTCAGTGGACTTGGATGGGTTTGGATGGACAATACGGGAAATACTCAGCTCATGGGGATGAAGAATCTTCCTCGACGTGAATCAGCCTTGCATTCGGAAGTAGAAGCGCTACGGTGGGCAATGGAGAATATGCTACAGCACTCGACATGCCAGCGCTTTGGGACGGATTGTAAGGAGCTGATCGCAATGTTAGATGATCCTCATGCGTGGCCTAGCTTTGCGACGGAATTGGAGAGGATAGAGACGCTACGGATATGCTTCCCGGAGTTTAGCATCACTCATGTTCCACGAGCAAG

>TCONS_00063540

GTTCCGTGATAAGGATTAACTGCAGGTTTTTGCAACATAACACGATAGAAAGAAAAAATGATTAACGACCCTTGAAGATTATATATCTCTAGGAGCTAATTGCTCCTGTTTCTTCTGGGACATAAAGGCTGATCATAGATCATGCAATGTGACGAAGTATCCAACCCCGGATACACAAGGAAGAGACAGAAGCATATTCTCTGAAATGATCAAAGAAGTAGTCAAGAACATGACGGTTCCGGTGTCGGTTTTGGATGTAACTTCGATGTCAGCGTTTAGAAGTGATGGTCATGTGGGTTTATGGAGTGATAACCCGTTGGTATCTGACTGTAGTCATTGGTGTCTACCTGGAGTTCCTGATATCTGGAATGAAATCTTGCTCTTCTTCCTCTTTAGACAACAATGGTTGTTACTTTCAGGTTGGGAACTCACTGAGGCTCGGGATGTCTATTCCTTCAGCGTATAAAGTAGCGTTAGAGACATGGGCGTCATGGATAGAGACTCAGATTGATCCTAACAAAACTCGTGTGTTGTTTCGTACCTTTGAGCCATCGCATTGGAGCGAAAAAACATCAAAGTCTCTGTTCTTACCTACCGTTCTCAAATTCGCAATCTTTCGCCAACTTCTTTTCACGGTATCGCTTCTTCTCTTTCGATTCGGCTGGATTTGATCGATACCCATCTCGTGATTCAATCTGGGTTTGCGTCAAAATGTTGAATTTCCCTGGAAAATTACATCAACTCTAAGAGATTGGATCATCTCTCTGTTCTTGCATGATGGGTTTAAATAGAAAGTTAAGGAGCTAAGAGATAGATAGATAGATAGAAGGATCAAATGATTGAGGAACAATGAGCAGTTTCACAAAGAGCACATCGTTCAATAGAAGAGCATTGAGTTCTTTATCTGTAGAAAGCCCTAGGAGCACCTCTTCAACCGCCTTCAACAGCCCCATTGGCTCAGCTTTCGCCAGCCCGAGAACATTCGGAGGTAGTCCACGTCCGTACACAAACAGGTTGAAAGAGATCTCGTACTTGTTTCAAGTCCTCATCGTCGCAGGAACACTTGTCTCGTTTCTTGTGATCATAGCTGGTGGTTACCTCTACGTTGTTCCTAGCCTCGGTTATAATGGTGCATTGCAGTTTAATGACACCTCTGTTATAATTAATAGTAAGGAGTGTGACATATTCGATGGGAGTTGGGTGGTTGATGCTAGCTACCCTTTGTACAACGCGTCTGAATGTCCGTTTGTAGAGAGAGGGTTTAACTGTTTAGGCAACGGGCGTGGGCACGATGAGTATCTAAAGTGGAGGTGGAAGCCTAAGCATTGTAGCGTCCCGAGGTTTCAAGTGCGTGATGTGTTGGAGAGGTTGAGAGGTAAAAGGATAGTCTTTGTGGGAGATTCGATGAGTAGGACGCAGTGGGAGTCTTTGATATGTATGTTGATGACGGGTTTGGATGATAAGAGGAGTGTTTATGAAGTCAACGGGAACAATATAACGAAGAGGATAAGGTTCTTGGGTGTGAGGTTTAGTTCTTTTAACTTCACGGTTGAGTTCTATAGATCGGTTTTCTTGGTTCAGCCTGGGAAGTTGTTGCGTTGGCACGCGCCGAAACGTGTAAAGTCCACGTTGAAGCTGGATGTTTTGGATGTGATTAACAATGAGTGGAGCTCTGCGGATTATCTTGTTTTCAATACTGGTCAGTGGTGGGTGCCTGGGAAGCTTTTCGAAAC

>TCONS_00052238

GTGGGGAATACTTATGGTATGTACTCGGTATGATCCGAATGAGTGCTTCTGCATGCAAAATAGCATGTCCCCATACAGAGGTTGGAAGTTTTGATCTCATGATCAATGGTCTTGCAATCAATTGCAGACGCTTAATTAAAGATTCAGCCAAACCATTTTGCGTATGAACATGAGCAACCGAATGTTCAACTTCAATTCCCGTTACCATACAATAGTCATTGAATGCTTGGGATGTGAATTCACCAGCGTTGTCTAGTCTAACTCTTTTAATAGTATAATCAGGAAACTGTGCTCGCAGTTTGATTATCTGAGTTAGAAATCTCGCAAATGCCACATTTCGAGATGATAATAGACAAACGTGTGACCATCTACTGGATGCGTCAATTAATACCATAAAATAGTGGAATGGTCCACAAGGTGGGTGTATAGGTCCACATATATCGCCTTGAATTCTTTCAAGGAACTTTGGTGATTCTTTATCGATTTTGGTTGGCGATGGCCTTACGATCAATTTTTCTAGAGAACATGCAACACATGTCATTTTATTCCCTTGAGAAATCTCCTGGATTTTCAGTTAATGACCATGTGAACTTTCTATGATTTTA

>TCONS_00055672

CTTAAAAGTACCAAAGAACCAATGCCAATTATGCCATTACCATTAAACCAGTAAGTAATCGTTTGTATAAATCCTGTTTTTATTATTCAACTCACTCTTCCAGATTACATATTATTATGGTTTGTGAATAATCAATCATCTACCAACCTCACAGTTTAGTTGACGGTGAGCTTTCCGACCATACCAGCTCCCTGGTGCGGCACACAGTAGAAACTGTAAGAGCCTGGCTCTGTCAACGTAACCTCGTACGTCTCTCCCGCACCATTGAGAAGCGCTTG

>TCONS_00060702

CAGACAAGTGTTAGATTGGATTTAGTCCAATGGATGATAGATGTTATTCCGCTGTGCATAAGTTGAAATGATCTAAGCTAGGGAATGACTAAGGTAGTCTTGAGCTAAGATCTGAGTTAGCCCTCGCCTATGGGCGATGTTTTTAAATAAAGGGCAAAATTTTTAGAGGTTCAGTCAGTGTATAGACCGAGCGACGTGAGGCATCGAC

>TCONS_00010234

AACGCGGTTGAAGATGTGCGGCAAGCGACGGAGATTCTTTCCGGGTTCTCGGGTCGGGTTGTGGTGATGATGGTGGTGGATCAATGTGTTGTTGTTAAGAATTGGATGGACTCTCATGGCTTTCCGGGTCGGGTTTCTAGATAAAAGTCAGAATGAGATTACAGATCGATTTCAAAGGAACCAAAACCGTAAAAGAACAAAAAGATTGCGTCTTCCTCTTCTTCTTTTCTTT

>TCONS_00009276

TAAAATGGAGATAGATAAGGTAGAAGACTCAGCAGCATTGGGTGACTCCAGAGGAGACTTCGACTGCAACATATGTTTGGATCAAGTACGTGACCCGGTCGTGACATTATGTGGCCACTTGTTTTGTTGGCCCTGCATATACAAGTGGACTTATTCCACCAACAATACAAGAAGACGTGTCGATCAGTACGATAAAAAGGAGTCCCCAAAATGTCCGGTCTGCAAATCTGATGTCTCGGACGCTACGCTTGTCCCTATCTACGGCCGGGGGCAGAAAATACCCCAGTCCGGTTCAAACGTACCGAACAGACCATCCGGTCCGCTTTATGACTCAAGAGGAGTTGGTCAACGTTTAGGAGAAGGTGAGAGTCAACGGTACATGTATAGAATGCCTGATCCAGTGATGGGTGTGGTATGCGAAATGGTATATCGGAGACTATTTGGAGAGTCTTCTACGAACATAACACCGTACCGTAGCGACCATGACACAAGTCTACGGTCAATGCGGCGGACAATGCAGGTGGATGAGTCGCTAAGCAGAGTCTACTTATTCCTGCTTTGCTTCATGGTTATGTGTCTACTTCTCTTCTAGTTAGTTTCGAAAGGCTTAATTAGCACTCAAGTCAGAAGAGATCTATATATCCTATAACCTCTTCCAAGGAAAAAGGAAGAATGGCGTTTCGTTACTTTATCTACTTATTTTTAATTAG

>TCONS_00055600

AATTTGAAGGCACACAGAGAGAAAAAAACAAACACATGAAGAAAGATTATCATACTACACAAATGTATTAGCTATTAAACTCTATTAAGTTTAGAGATTTGCGTTGGAGTGGAGTTAGAGAGAGAGACAGAGAAGGCCAAGAAGCTTGAGGCAAGTTTAAGTGAGACTACTAGATGAGTCTTCTTCTGAAGTTTCTGGTGGTTGATTGATAGGTTCGCACCAGAAGGCCAACACCAATGCCGAGACCTACACAGACGCCAAGACCAATTCCAACGCCAACCCTCACACCAGC

>TCONS_00060038

TTCTCATCTGTAGGTCGATGGCGTGTTCCACCGCTGCAAGCATTGCTAGTCCTTCTGCCATGATGGACGAGATCACAAATGCTGATGATTGACTGTGAGAACTGATTCTCTCACTTTGATTCCGGGAGAAACTCCACGCCATTCCAGCCGTGTGAGTTTCCTTCTTCCAAGCTGCGTCCGATCGACAAGTAATTTCCACAGCTAGCGTGTCTTGGGGATGAGGTTTCCTAAGAATTAAAGACCAAAT

>TCONS_00042190

GCATAGAGTTCAAGCATTGCTTCTGACCTAGCCGATGGTAATCTCCAATTCCCCTCTCGATTCAGTGAAGCGATGGTTGCATTCAGCGGAATGCCTAGACGGCCACCTGGGAAGACCTGCGACATGGTTATGGAGCTTGTGGGTGCCCAGTTATCTAGCCAAAAGCGGGCCGATCTTCCATTCTCAACCCTCATCTTGATTAGTACTGTTGTTTCTATGCACATGATTCATTGTACTCAACACAGTACTCTGATTAATTGTACTCTGAACAAACTTGTGAGATGGATAAGAGAGTGGTATATTCTTATTTTTTCCAAAATGTACTCACGTTAAAAATTGTTCACCTACTCGCACCATTTGAGTCTTTGGCCTGAGATTTCTACCCCGTAACTGCATCATTTGATCAAGTAAAGATCTTTACTAATCCGAGACGATATTTCATTAAATCCCACGAGACTCAAATAAAGAACTTTCATGTTTGCAAGGCCCACCAGCCCAAGAAAGTGAATCGGTAGACAGTTTAGGAAGTTGGAGAAAGTTGCGTCGATGCTTACGAAGGAGTTGATCGAGTTGGAGTTGTTTCGAACCAACGCTGCATCATCTGTGAGGAGAGGGTAGGATTTGATTCCCTAAAGCTTTGGATCTTGTTTCTAATCTGACGGTCAAGCAGAGAGAAGATGGAATCAACCGACCTGAAGGTGTTGTTATGGTGACGGTCATTGCGCTCCTTCCATAACCAATACACAGTTGCTTGCCATGAGAGGAGTGTAAGAAGACGCTCAGCTTTCCGCAGAGGTAACGTCTCCATTTGGGCGATCGTTCGACTCCAAGATCTCCTGGGGGTGATTCGACATCGCGTTGTTACCATTGTCCAGAGTGCGAAGCCGTAGGAGCAGTCAAAGTATAGGTGATCTCTTGATTCGGGAGCCAAATTGCAGAGGAGGCAGGTAGGATCTACTTGAATTCCCCATTGGATCATTCTATCTCTAGTTGGACACCGATCAAGAGTCACCAACCACAAGTGGAAAGAGTGTCTCGGGATAGCTCGAGGAAACCAAATTGCTTTAGTCCAAGATGGTTCTGAAATTGTACCTCGCAA

>TCONS_00047173

CAACCGTGTCATAGGCTGAGCCAAGCTTGCGAATCCTTTCACAAACTTTCTATAATAACCTGCCAGCCCTAGGAAGCTTCTGACTTCCGTGGCACTGCGTGGTCGAGGCCAATCCTTGATTGCCCTGATCTTCTCTGGATCCACCGAGACGCCCTCACCGGACACAATGTGGCCGAGAAACCCAATGCTCTTCTGCCAAAAACTGCATTTACTGAGTTTTGCATAGAGCTTGTGTTCCCGTAATCGTTCCAGCACTGCTCTCAGGTGTTTCCGATGAGATTCCTCATCCTTGGAGTAAATCAGGATATCATCAATGAAGATGATCAC

>TCONS_00081665

CTATATAATTACAACTCCAATAATTAACCATTTCTTAAGTGGTGTTGAAGAAACCGGTCAATACATTGGTTTATGTTCTCTGAATATAACATATCAATCTATGGAGGATGCTCAAATACGTAAATACTTCAAAATGAGTCCTGAAATGACAGGAGTACTTATAAACGAAGTGTATCCGCTGTCTGGCGCTCATGGGATTCTGAAGAAAGAAGATGCCATTCTTGCCATTGATGGTGTTTCTATAGGAAATAATGGGACAGGTAATACTCCTAAAAAAGTATATATGGGTAGTACTATACTCCCAATGATGAGTGTTTGTTGAAATGGATTTATATTTGTTGCATGCATGCAATTCCTTTTCGCGAAGAAGAACCCGTGGATTTCAATTATTTGTTTCCTCTAAAGAAACCAGGCGAAACAGTCTTAGTTAAAGTCTTAAGAAAGGGAAGACAACATGAGTTCAATATCAATTTAGAACTTGTAAGTCATATATCTACGTAAACCATTTTGGCTCCTTGAGATCTAATGATTAAATCATTGGCTTACATTTGTTTACGGTTATCTATGATACGGAGGAGAAACTGCTGGTTCCGGACCAATATCTTCTAAGTTATTACATACTTTCAGGTTTTGTCTTTGTGCCTCTTTCCAAACCATTCATTGATGATTCGGCTGATATGTGTGAATGTCCAACAAACGAAAAGGCTAGAATGTCTGGTGAACAGATTGTTATCATATCAAAGGTTTGTGTTTTTCGCTACCCAATTCTTTTTAAACGTGAAACCGGGCATGGTCCCTCTTTGTATTGAAAGCCTGTACAAGAGGTTGATATGTATCTTTTGGTGTAGTTCTTAATGAATGACACAACCAAAGGATACAACCATTTAAAATTATTGCGGGTAAGTTATTTATTTATTCTTCTCAAGGGAAAGTAGTCTGCAGCACCATGACTATATAGTTTTACTAAATCCTACAGGTGATGAAAGTGAACGGAGTCGAAGTGTTGAATCTCAGACACCTAAGACAGCTTGTGGAGGAGTGTTGTGCTGAACAGTTGAGTTTTGACTTAGAAAATGGAAACGTTATTGCCGAGAACTGCAAATCTGCAAAAGAAGAGACTCCGTTAATGTTGGAACATCATGGAATACGGTCGGCCCTGTCGAAGGACCTTATGAAAGAAGCCAGTGAACTAGCTTCCTCTGCAGTAGCTTGAAAAATTAGGAACATGTTTTTTTAAATAAAATTTCAAATTTATTAGATCAATAAAAATAACATTTACAAAAGAATAGATCTATAGAATGATCCTTTATGTGAAATAGAAACGAATGATGTCGAATGAAAGCTTCAAATCGAGTACTGAAAGTCCGAATGTTGTTCCGGTCTGTTCGTATTGTAGCCAGGTTCTACAACTCCTCGAACACCACGATTCCTCGATTCCTCTATTATCCTTTGATATCTCGTCGCTATTCTTCTGTGTGCCTTGACTCGCCAAGTGATTCAGCACAAGAAAACGAGAGTCGTTCGGCTATTGACTCGGTGGTGAAGATCTTCTCCTTCTATAGGTTGCCAAATGTTGTCTAACCTTGGCAGACAACTGAGGAAGAATACTCAGGCTCTGGTATTCTTATTCATCTCTCTCGTGTGATATTATATCTTCAAATTCAGTTATGTTCTTTTTGTTATCTCCAAAATACTATATGGTATTGTTCTAACTTACTTTGGAACATTAATCTAGGATTTGCAATCTCTGGAAGGAGGATTCTTACAAGTGCTCATGTTGCGAATCACTCATACGTACAAGTGAGAAAGCATGGTTCACCCACCAAGCATAAGGCAAAAGTTGAAGCGTTTGGGTATGAATGTGACTTGGCCATTTTAGTTGTTGATAGTGAAATATTTTTGGAAGGATATGAAGCCTTTGGAGCTTGGAGACATACCCTTTAAACAAGAAACTGTCTTCGCTTTAGGTTATCCTGTTGGTACGTCTGGTTTGAACAATGCATATTAAATTTATGCCAAGAAAACAAAATATTTGAACGTGAAATTGAGAAAGGAATAACAATCATGCATTTGAAGATAAGTAATTTTGGGAATTAATATGTGATGGAACGAAGAATCAAAGGAATGATTAATATTTTATGATATGCGTTCAAATGCAGGTGGTGACACCATTTCGGTTACAAAAGGTGTTGTATCAAGAATTGAATCACGAAAATATTCTAACAGCTCCATTGAACTACTAGTGATACAAACAGGGTCGGACGCAAGAAGGCGTTGGGTGTGGTCTGTGCCCCACGCAAAATGTTGTTTTTCCTTTAATAGTTAACATAAAATTTATATAGACACCCTTCATCTAAGCATTTTTTATCCCATACTAAATGAGTGGCTGGCTCCACCCCTGGATACAAAGCGATGCGGTTATTACTAAAAAAAATATTTTTGATTGGAATCCTTAATTAATAACAATTATTTTTTTATTCCTAAGAATTCCATCTTCGGGAACACCATTGGACACACAGTTTTGATTGGAATCTTTAACTATTCAAAAAAAAAATATTACTAAAATATTCCTAAGGATTCTAAGGTGGAGTACACTATTGCATATGCTCTTAAGACCATCTTTATCGGTGAGTCCTTAGTGAGTTTTTAAGTATAATAAAGTATTTAATTAAAATCAAAACAAAAACAAAATTAGATAATCGATCCTTAATAGAGGGTTTTGGCAACCGATAGTAAAGAGAGTTCTTAATACACGTGTAAGCAAACTGTGCATTATGAATTTTTTTTCTTTTTTTCTTCTCTCCGGTCCTCTAGCGTTCTCCGTTGTCCAGACGATCGATCCTTCGAATTCTAAGTACTTGATGTCCGATTTCAGAAAATTCCTTAAGAGATTCGTACTCGTTGCGCAACGCCGCCGTGAGGCCCGTGACTACGAGGAGGAATCAGCGGCGGAGGCCACAAATACTGATTTTGGCGAGGGGGAGATCGCGAGCGTTCACAGCGCGGGGCTTACGAACATGCTACACAATGTTGGGACTTTAGAGAGTTTTACTTGTCTGAATGGAGACCATTTTAACTAGTGTGTGGGAGTTTGAACAGTTGGGGATCTGATTTTCAGTGTTTTTATTGATCTGCTGCAGAGATCATACGTTCACAAGAAGAGGAACAGAAGAATGAGGGAGAAGCAAGAGTTATTGCGTGGTGTGAATGCTTTCGCAGCAAACATGTTTACGGTATTACCTCATCTGTGTGTTCTGTTTTTTTTATCTTGCCTCATCTGTTGTTTGTTTCTGTATGCAGTTGTTGCCCCTCCTCTTTCATTATTTGTAGAAAAATTGGGTTGCTTTACGGATTGAGCTTCTTGCAAAAAGTTACATGCAAATTTAGTGTTTCCTGCTTTGCTTCTCCCATAAGCTGAAACTTTGTCCTATAGGCCACTGCTGGTTCTTCTTATGCTCAATTGCTCATAGTATTAGCTTCTTCAGTTACTAGAATGTTCTTTGTGAAGAAATTAGAAGGTAATATTTGTTAGTTGTTCCTCTTTATGAATAAGATTGCTAGAAGCTTTTGAGTCTCTGTTAGGTTTGAACTTATGTATAAGATAGCAAATTGTTATGTAGTTAAATTTTTTTTTTTTTAAGAAACTCAAATCAAGAAACTACCATTGTAGGTTAAAAATTAGGTGATTCTTAACTAAAATCCTTAGCTAACATTTATTAGTTAAATAATCATTAAAATGTACTTAATAAACTTTATGGGTTTCTAGGGATAATCATGCTCTTAGTTTTCCCTTCCAACTTTTTTTGATCGCACTAATAATTGGAAGGTGGTTGTTTTAAAAAAAAAATAAAAAAAAATTGGAAGGTGGTTTTAAGTAATAAATGAATATTATTTTCTCAGGGGAGATAATGGTGGTCCGGTGGTCATGGGGAAAAAGGTCGTTG

>TCONS_00044063

CAAAAAAAGACTCCAAAAAGAAATTACCTACTAGCTATCAAACAAGCGACGCCAAGTAGAGAGAAAAGTAACAGTGGAAGGGAGGTTCCTCTGCTCTCTGGAGAGTATGTCCATCTTTATTCGAAGAAGCTGCTTGATCTCCAAAATCAGTGAGGCTGAAGGACGGGAAACCTGAGAGTGTAACCTTGAGTTCCTTTCTTTCCAGACCAAATAAACACAAGCTTGAAAAGCAAGTTTCAGAATAAGATTGACATTGTTGTCGCGAGTAGGGCCTTTGATCCATCTTAGTACATCCATGATCAAGGTAGGAGGAGAAAGACGGAGGGTAGAGTAGAAGAAGGACCAAACTTCCTTGCTGTACCCACAGTCAAAGAAGATATGCTGGCGAGACTCATCCGCTGTGCCACATAGAGGACATGTAGGAGAGACCAATACACCCCATTGTCTCATCCTGTCCCTAGTTGATAATCTGTCCAGAGCTACTAACCATGAAATAAAAGCATGCTTTGGAATCCTTCCCTTGAACCAGATAGATTTATGCCAGTTCACTTCAGGTTCCTGTTCATACAAATGATTCCACATGAGGGACGATGAGAAACCTTTGACAGGGGCGTTGTCGCCAACCTTCCATAGAAAGCGGTCATCTGCTTCTGATGAAACTACTTCCTGATGGTCGGGGAGGCAGTTCTTCAGTAAGCATATTGTCGGGTTTCTTGATCTAGATGAGCTTATCCACCAACGGTTACCTACCAGCGCATCTCGAACTACTGCCATAGCTGGTAGGCCTGTCAATCTCGGACCTGACTCGCCTGTTAGCTGAATCAGAGGACCCAAGGAAGTCCAGTTATCGTGCCAAAAATTAGCGGAATTGCCAGAGCCAATCTCACATATCACAAAGGGACGAGCCAGTTGCCTAAGCTTACAAAGTGACTTCCAGATCCAGCTGCCTGAGCGAGTAGCATCAATGTTCCAGAAGTTCTCTGCCCCTATAAGATGTTCCCGAACCCATGAAACCCATAAGGAACCAGAAGAGGCAAAAAGAAGCCATATTAGTTTGAGTCCCATGATCTTATTCCAGAGACCCAACCGCCTGAGCCCTAAGCCTCCTGATTTCGTTGCAGAACAAACGATATCCCAAGCAACTTTTGCTCCTCTAGCTCCTGTAGGAACCCCTTTCCATAAGAAGCTGTTGCACATTTGCTCCAGTGCCTTGAGGCACCTATTAGGAAGGAGAAAGATGGAGGCCCAAAAAGATATAGTAGCATAGATCACTGACTTTAGTAATTGCAATCTTCCTGCAAAGGAGAGGTGCTTTACTGTCCAAGAAGAGAACCGAGAATGGATTTTATCCAGGAGGGGTTGATAATCCTGCCTTCTCAGTTTCTTAGTAGTCAGCGGCACCCCTAGGTACCTAATGGGGAAGGAACCTTGCGAGATGCCATGAGCAGCTGCAGAAGTTCTGTTCCTAGAAGCATCGCCTCCATCAAAGAACACTGCAGTCTTGCTTCTATTAATTCCCAGCCCGGAGCACTTGTTAAAGTCTTCAAGAATAGTCAATAAGCCTTGCAGAGATTGATCAGTACCATCAAAGAAAAGAAGAACATCATCCGCGAAGCTTATGTGGGTAATGAGAGGTGCATCGCCAAGCGGGTGAAGGCCAAAGACATGATTCATAGCGCCTTTGTCTAACATCTTGGAGAGAATGTCCATGGCTAAAACAAACAGAAGGGAAGAGATAGGATCCCCTTGCCGCAAGCCCTTTCTACCAGGAAAACAATCCAGAAGCTCACCATTAAACGCTATGCTGAAGGAAGTTGTAGTGATGCATTCTTTGATCCAGTTGATGAATAACTCTGGAAGATCCAAAGCTGTAAGAATGTTGAGCATGAAGTCCCAATTGAGGTTGTCATAAGCCTTAACCAGATCAATTTGAAGACAGCCACGAGTAGTAGGTCCTCTCTTATGAAACCCTGTAACTAGCTCAGAAGCTAGTAGAACATTCTCACATAGTAATCGGCCTTTAATAAAGCCAACCTGATTTAACTGCACTGCTTCTGGAACAAATAACTGAAGACGTTGCTTTAAGAGTCTAGCCACCACCTTGTAGATTGTAGAGCAACATGAGACTGGCCTGAACTTGGACAACTCGTCCGCGCCTGTGATCTTTGGTAACAACGCTACTGTGGTTGCATTGAACTTTTTTAGCAGTCTCCCTGAAGTGAAGAATTCCTTTACAGCTTGGATAGAGTCCTCACCAACCACCTCCCATGCCTCTAGAAAGAATTCCGCTGGGAAGCCGTCCGGACCAGGAGCCTTACACTTAGGCATTTTGAAGAATGCAGCCGTAATTTCATCGTCTGTGGGGATCCGCGTGAGATCAGATGCCAAATTCGAGGAGCACCTGAAGGGGTGGAGTTCCCGGATCTGGTGGACTGTCATAGGCTCAATACCCCTACTCTCCGAGCCCAGCAAATTCTTGAAATAAGCCACTGCCATTCCTTTAATCTGCTGCTGATTATAAATTCTCAGACCAGAAGCATCCCTGATGTAACGAATGGCGTTCCAAGACTGATTGGCCATAACAACGCGATGAAAGAATTTTGTGTTTGAGTCTCCTTCTCTCAACCACCTAATTCTTGATTTGAGCTTAAAGAAGTTCTCCTGAGCAGAAGCAAAGAAGGTCCAGACATCCCTTGCTGCTGACTCTTCAGCCACTAAAGCCTCGGTTGGAGCAGTTAGCAGAGCAGCTTGTACTCTTTCCAGATCCTCTAAAGCTGTTTTAGTTCTATGTTGAATATTTCCAAACCCATCCCTGTTAATCTTCTTGCAACAGTCCTTTGCTTTCTTTAACCTCTGACCGAGAGTGAAAAGCTTTGAGCCTATTCCCACTGATTCTCCCCAAGCTGCCCGGAGACGAGGCAGAAAGTCGGAGTGTGTGGAGACAAAGGAGAAATACTTGAAGCTCTTCTTTCCAGAGTGGAGAACCGGAGAGAGAGAGATGAGACATGGAGAATGGTCAGAGTCACCTGGTGGATCAAAGGTAGCCAAAGACTCAGGGAACTGGGAATTCCATTCTGGATTTACTACTGCCCTATCAAGCTTTCGCAAAATGGGGTCTTCTGGTCTACAGTTGGTCCACGTATAGTAGGTGCCTCTGCTGCTTAGGTCTGTGAGGTCATTAGTTTGCAAAAATGAACGCAGATCATCCATGCCTCTGATAGGAGTATCGTAAGCCTGAATGGAGTAATGCTCACCAGCATTGAGGATTTGGTTAAAGTCTCCTATTACCAGCCAAGCAGAGCTGGCGAGAGGAGTAGATGAGTTAAGGCCAGATAGCTCTCTCCACAGGTCCTTCCTCTGACCCTCTGTATTGAATGCATAAACAAAGGCCACCGAAACAGATGTACCAGTCGCTGGATCAAACACACCGCACAAAATAAACTGAGCTGATTTCTTATAAACTACCACCGAAAGAGCAGGATCCCATATCACCCAAATTCTGCCCAAATCCGCAGAAGAGTAATTATTTTCCCACTTCCAACCTGGAAAGGTGGATTCCTTTATTCTTAAAGAGTTCTCCTCCAAGACCCTCGTTTCCAATATACAGCCGATGAGAGGTTTATTATTATTGATCCAAGATTTAACAAAGAGTTGCCGCGCAGAATGATTAAAACCTCTAACATTCCATGAAAAAATCTTCATATATTCAACAAAGGACAGGATACGAGCTAGCAGATGTGAGATTCCCTCCCCAAGGATTTACCTCGGATCGAAGAGAACATATGAGATTCCCTCCCCAAAGAG

>TCONS_00039788

CAGTGGCCGTATTAGTATCGTGGAAAGCAAAGCTTAGCCGATCAGATCTCTCCTGTTATCTTCCTTACACAGAAATGGCCAAGTCGAAGAATCATACCGCGCATAACCAGTCGGCTAAGGCCCACAAGAACGGAATCAAGAAGCCCAGGAGGCACCGTCACACTCCCACCAGAGGGATGGACCCAAAGTTCTTGAGGAACCAGAGGTACGCGAGAAAGCACAACATCAAGAGCGGCGAGAATGCGACCACTGAAGACTAAGCTTTTGTATTCTCCCACCTCTTTTAGTAGACAAAGAGAACCAGTTTACTTTTTATCTTTTCGTTTGATTATGTTTCCGCAAATGGTTTGTGCACTCTTTCAATCCAGGCCTTTAATTTAATATCAGAGATTTTATAATTCGTCTTCTCTTACAAGTTCCATTTCTTGATTTGATTTAGCTCGTTCAACTGCTATGTTGTCTGGGATAGTTCGATCTGGTGGTTTTTATTCATAAATGCGTTCTTTTCATAGTGTCTGTGTTTATGATAGTTCTTAAGTGTGAACTTCGAAACTCTAAGAGGATCACAATCTACATGTATCTCTTACCAATTTCTATTTTGGTTTTGTCTAACTTAAGCTGGTATGATTAGTGGGCCATAGTTGAACAATTTTGTTGTCCGTCTTGTGAATGTATCCGTGCGTGTTACAATATTAACAAATGGTCTCAAATTTTTATTTTGCTTAATCAGGGAAAAGAGTTATATAATAATTTGAGCAACTATCTGTGCTAATGAAAACTACAAAACATGGCGAGTATAAATTAGTAAAGCGGTTAGGGTTCTTTCGTTCTAATTAATATAATC

>TCONS_00028292

CACATTATCTCAATAAAGCTTATAGAGTTGAGGATTCATTGTCAATACAAGAACACATCATATGCTAATATCTTTAACATACAAAACTAAAAAAAAAAGAGTGCAGTCCTGCAGAGGTGAGTAATCAACAAAAGGATCTAATCACAAGTAAAGATAACAGGAATCTACTCGCCAGGAACTTGAACTCCTTGCTTCAGCTTCTCTTTCTTCATCTTCTTCTTTGAAACGGGCTTCTTCTTCCTCGGAGGCAGATTCTTCTGCGCGTAGGTGTAAAGGACGAAGTTCCCTACGAGGAATGCCACTAGCAACCCTCCAATCACAAGAAGGACGATCAGCCCCGGGTTAAGCCCTTTGCTTTCAATCTTTCCCTGTTTCATTGGTTCTTCAACAACTTAGAAAATGAAATCAAGATGCAAGGAGTACAATATGAGAAGTGACACAAGTGCTCAACAATAACAGAACTCAAACAAGCATGAAACTCTCCAAAGGCTAAAACTTTACCGATCCAATTCCACAAGATCACAGAACCAAGCTAAAGTTTCATGTAATCATGTCAAAATGGACTCAAAATGCTCGCAATTTT

>TCONS_00006713

GAGTGTTGCATCCCAAATCTGCCTCAAGCCATAGTATACCTCGAACGGTTTAGTCCTTCTTCCGTATCTTGTGGTCCCACTACTTCTTTCCATCATAAGCTTTAAGATGTCTTGTTGCCAACAGAAAGGTGCATGTGTGTCCATCGTCTTGTACTACAGTTGAACCATCTCTTCCTCCAACGCTAAGCTCCAAACAAATGTTGAACAATTCAGGCTTGTACGGCTCTGTTTCTTGTACGTCTCAGCATAAGCTGGAGACTTGAGAAGCTGGAAGAATAAGTGCATCTAGGTGATCATGTGCAGTCGCATCGCTCTGAATCGGACCACAAACAACGCCCACCTTGACATCGATAACACACGAGTCCGGAGATGTATTCGATGTATCAATTATGTCCCATATCTCTCTCTAAAGAATTGGCAACCCCCACTAGAATTCAAAAGAACGAAAGGAAGGCATGACATTACTGACTCTTAACAGGGCAGTAGTGAACCCACCATGAGGGTAGCAAACTCGAGTCTGGTGATACTAACATCGGTAAGTACGGTCCACCCGTTCAAGCTTGTCTCGGTGCTTCTGCTATGAAATATCACTGTTAGGCAACTACCCATGACTATCTATCCTAAAATGAAGGAACAAGCCAGCATATCCAAGTTATCCTTGCGACTCATTTTGACTCGAAAAACATTTGAAACAAGTCCCATTCTAGCATCGTATAACCATGCTCTGATACCAC

>TCONS_00027586

GCGAACAACTCTTGGAGATGGAAATCTTGGTAGAGAATCTATCTTTCGGTACTGTGTTATATGAGCTATAGATCGTTGGAAGAATGTTATACTTTATACTTAGTTATGATATTACGAGCTGTAAGATAAGCAGACAAGATCATGCATAAACTACCCAACCATGGATCCATCTTCCTTAATAAGACCAGGAACAGGGACAAGAGATGAAATGAAATAAAGATGCTTCTTGCAACAATTTTGAGTATTTAATGCATCAAAATTCTAGTGCTTCACAGGGCGGTGTTCTGCTGACCTGACTCATGCTATACCTAACATACATAATAAAGGAAACTAGAACTTCTTCTTGTAGATGGACATTTTCAACATGTTTTGCTTGTGCTAGTTCCTGCAGCAGCTGTGTTAACCTGTTTCGAGAGATAAGCTTCATAGATTTCAACGACAAGACGAGGCTCCTTTTCCACAAGCTCCATGTATTCCTCTCTTTTGGTAACATTCTCCATGTTTTCAATGATCTGTAACAACGCAGCATCACGCACGTGTTTTGCGTTGTGCTGGTGGGCAAAGGCATAGGTCATGAGAGAGTTGTCCGGATTGAGTTTGGTTACCAAAAACTTCTCACAGTAACTCTTGAGATGTTTGACTTGGTATTTCTCTGACATGACCAAAAGATCACATGCCATTTGCTCATCAAGACATGCTTCAGCTGTGTAGAGATAATAGACGAACGTTCGAAGAGTGTCGTAAGATATGTCGCTTATCTTGATTGTGCCGCTACGGCTCTCTTCCATCTCGTTCTCAAGCATTGCTCTGAAGACTGGGGAACGACTCACCT

>TCONS_00055595

CGCAGTTTCTTTTTTCCTACACTTATTGATTAGTTTGGTTTCTTTGTGTGTTCATGAAACATCGGTATGTTTATAAGATTATGTTGTTTGAAGTTTTTCCCTAGTTTAGTTGATGTAAATGCATTTGTTTACCCACGTTTCTACCCATGATGAGTAAAATCACAAAGAATGGTAGAGATGTTAAGAACGGGTGCAGAGATTGAGATGGTTATCTTACCAACCCATTGCATATTTTTCTCTTGCTTTGGCGTCAAATGAATCGACCGAAATTTCTCTTTTAGCTTAGGGATTTTAGATTTTATGGCAGATGATGAATTGATTACTTGTGCTTAAATCACAGAATAATAGTGTTGACAAACAATCTCCGCTTATTATCTGATGCCATGATTTTTTTTAATCGAATTTGAATAGTTTATTTTCTTGTGGTTTTGTATTCGGATCATACAGTGATGAGATAAGCAGACGGGCGGTCTGAATTGATGCCATGTAGACGTGTCTGCCTTCGCTACAACTTTGTAGCTTCTCTGAGTATTGTCTCTTTGTTTGGTTTATGTGTTTAATTGATATTTGCCATGATGAATTTGCTTCTATTATGTCTGGACATATGAGATTATTCAATCTTTGATCTGACTGGTGCAAAAAATTCTGAGCTACCTATGTTTTCACTTTTGTTCTTGTCATTGATGTAATCCTAATCTGCTGTAACTAGGGTCGTGTATAAATTGCTACAGTAAAGGTATTGTTATATCCGGAAAGTCTAGATAGACCATTGCCGCGTGAGCTTTTTATTTCTTGTTAGTCAACTGATTAAGTTGCAATTTGATCGGAGAAGATAGTCCAATGGATATGATCATGATAACTGAGTTGATAAACTACCATTGTTTTACTATCATCTTTTACTTTTTGTCTCATCTATTTTAGGGAAAGGACCTTTAACTAAAGTAACGAACTGGAAATATATATACTGCAACAAAAAGTTACACAAGGCGATAGAAATGACATAAGATGGCTAGAAGTAATTGCCCAATATTAACCAATATGAACCAAAGCAATTTATTCAGTTAGACACACTACCTTTCAATAAGGCAAAAGCACGTTAAGAGTAACATCTTAAAAGGACTATAGAGGTTTTGTAGTGTTCACTATGTCGAGGTTTGATCTTGGCAGATTCATGTGATGCAAATTCAGAACTCTCGAAGTTATGTAATCCAGATTCATTCTCTCGAAGTTATCCTGTAGATTCTAGAGAGTATCAATTTATGAAGTCAAAATACATATTCAAAATGGATTGATCGGATCTTCATTATCTCATAAGCTGGTTCAAAGTAAACCTTCAGCTCAGTGTGT

>TCONS_00072185

GTGTACAGACATGGATATACAGCAATTCCAAAACCAACCAAGATGATCGAAGAAGAAGAAGTCACGATCAGGTAGTTATATTCTTTTTATAAAAGATACCAAAATAAATTAAGTTAAATAAACTGAAAATTGGGATTACGAATAATTGACTCATTGACGTGGTTCCCTGATTCTTTCCTTGTTTACGCGTACAGAAGATAGCTTCCAAATAGTTTATTACTCATATTCCACAAATAATGAATAATACTAATAAAAATGATAATTTTTGTTAATGAAACAGAGAAGGTGGAGGAGGAAGAAGAAGATTAGTGTCTATAACCGAACCAGACTTTACTCAGTCTAACTCTGTTGCTACTTTCTTCAGATCATTAACTTTCACTGTAAGTTACCGACCTCTCTTTTTTCTCTAACTAGTTTAATTTCTGGAAGTATTTTTATTTTAGTTATTTACCAGAAGTTTGGGCAGATAGGGAGTGTAACAAGAGGGATAATCCATATTAGTAAATAAATTACTTAAAAACAAATTATGACGAGGGAAAAGGTCGTTATTAGTTTAGGGAGCTCTCTGAAATAGATATGGCAACCACTACTTACTATAGTCATAGCTTTTTTTTTGGCCAAATCTATTTCGTAGCTATGTGGCAAAGCCTATAAGTGCTATTATAATGTTTCTTTTCCCACCATGAATAAAGCGAATTCTTCAATTTATTTAAAACCAAAAGTAAAATGTTACCGTTATAAATTTTGTCACCATTTATCTGACGTATTATGAAAAGAAGAATTTGGTGAAGGATTTTTTTTAAGTTTGTTTTGGGGGTTAGAAAAATTATATTTAATGGTTGGAATGTGTAAGGTTGGGTAAGTTTGGTGGTTGGCCAAGAATTTTGAGTTTTTAGACTCAACAATTTTAAAACATTGCCTCACCTAAAATATCACAACTCCTAACAATATATTTTTTCTTACCCTTTCTGATAGTTTTTATATCCAGAGTCAGTTCTGAAATTTTGAAGATATAATTATTTTGTATAAACTTCAGCAATATATATTTTAAAACAGTTAAACGATGCTTTTAATATAATTTGGGGACACAAAACTTTTGTATATTGATTCTTAAAATTTCAGAGAGTAAAACCAATGTTTCATTTTGATATGTCGACCATGTGTTGTTGTATGTTATAGTGTTTTTTTTTTCTTCTTTTTAGTTTGGTGGTAAGTAGATCATAGTTTCTTTTTTACCTGTTGTTGAAGCCTAATGTTATGATTTTGGACCCATTTAGTTAAAGTTAAACCAATGTTTTTTCAGTGTGTATGAAAACATTTGGTTAAAAACTAACGTACACACATTATTATTGCAGCTAGCAGTGTTGTTGTTGATGAAACATACATTCGATGTGATTTATGGAACGGAGGAGTATCCATTCACTGTATTCACGGTAAAAAACAATGCTCTTTTTATTGTCGACACGAAATCATTTACTAATATTAAATCATAAATAAGAATTGGGTGAATGGATATATAAATGTAGGTACTAACATTAAAAGCCATTGGAATTTTATTGCCAATGTTCATTATCATTCAAACAATCTCAGCTATTCAGAAAAATATCCGTCGTCGACAGCAATATCCTGTAAGTTCACCAATTTACATTTAATCTATCAATTTCCAAAACTCATAGTTATAATGAAAAATGCATATGCATGTATGTTTGACTATATATTTGGAAATAATTTGCATGTGAGATGTGAACATTATTAGTATTTACGCATGCTAGTACGGATTAATTATAGGTTTGTATAGGGTAACCACGTTTTGAAAAGATTTGTGATCATGGGAACAATCTTTTAAAAACCATATTTGACTTTGAATATTCATTCATTCATTCATCATTTAATGCCAAATGTGAAGTTTCAACTTTACTCTCTGAATATGCTGACTTTTTGGGGGGGATGTTTGACTTTTGAATATTAGGATTCTGAGGAAGATACGTTGAGCTCCTCTGACGACGACGAAGATGAGTTGGACGATGATGAAGAGCAACAACAACATTTGGCATAAACACGCATATTAACCGAGATTAACTATTCTTTTTTTTTATTTCTTCTAATTCATTTTCTTTTCTTTCTTTTTTAAACGAAAAGCATCTATTCGATGCTCATTGTACAATCAGTACAAATAAAAAATAAAAAGAGAGTAAATTTACATATAAGAAAATCAACTTTATACGGTTAAAAAGAACCACAAACAGGAAAGGCGTAGAGCATCTAAAGTCTAAACCAAGTAGTTACCGGAGAAATGTGTTGGTCATGTAGTGTATGTAGCTCAGACAACGAGTCCTATGAAGTTGAGTAATCTGATGCAACACTACTAGTGATCTGTTCACACGCGTAAGGGTAAAACAGGCAATTCATATATATTATTTTTGCTTGAAAAGCTGTATAAAGTTGCAGTCTCTCTGACATAAAGCTCTGTCTTCAAAGGGATAAAAAAAACAGAGCAACGTCTTCATAGGAAAAAAAAAACAGAGCAACGTCTTCGTAAAAAGTTAAATTCAAAAAAAAAAAACAGAGAAGGATCCAGAGAGAGATGGGAGAAGTGGTGATAATCATTGATGAGACGAAGCGTTCAAAACAGAGAATATCGCGTTGCAGAATCTGTCACGAAGAAGAAGCAGAAAGTTTCTTCGAAGTCCCGTGTGCTTGCTCTGGAACCGTTAAGTTCGCGCATAGAGATTGCATACAACGTTGGTGTAATGAGAAAGGAAACACCACTTGTGAAATCTGTCTTCAG

>TCONS_00041723

GCCCATCGCAGCGCTTCTACCTCCGAATGCAGCGCTGATTCACATCGAGTGAAGTTTCGTGTTCCCATAAGTTGTATGTCCTCCCTACTATCCATCCAGACCCATCCACATCCACTAAAGCGGTCAGAAGCTGTCCAAGATCCATCTAGTAGACAGATATTACCCAAGCTTAAGACTTGGTTTGCATCATTATTGGTAGCCTGGACGTGTGGCGGTATCGTCTCATTCGCGTTAAACCAGGCTTGACATTCACTCTCTGCGTATCGAACGAGTTCCAAAGGGTCTCTGTCTATACCCCTGAAAAGCTTATCATTGCGAGCCTTCCAAATATACTATATTAGCCAGGGATAAGGATCCCTGCCTTGGTCTGGTGCAATGATATTATTCTTCCTCCAGAAAAGATAGTCCATGTTTGTGTAGAAGCTTGCCACTGGGAATAAACCTGGACTTGTAGGAGTTGCCGATAAAGACCATACTTGCAGGGCTGGAGGGCATTCAAATATTGCATGAGTTACAGATTCCTCTATTTCCCCACACCTTGGGCAATAATTATCGCACCTCATATTACGTCTTACTAGATTCCTCGTTACTGCTACCTGTCCCGTTATCAATTGCCATATAAGA

>TCONS_00058258

GCGCTTTACCCACTATCAAGAAGTGAATCAGAGACTCTAAATTGTTGCCTTTAATGAGCAAACAAAAACCAAACACTACATATAATTTGAAACAAAAAGGTCAGAGGAATCATAGGTATGTAAGGCCTTTTGAATGCACATGAACTATTTAAACTAGATCCCGTACCAGAATATCAGCCACATTCAGTTTCGGCAACAAACAGCGCTTAATACATTATTGATAATAAAAAGTGCACCAGCGACTCTAAAATGTTGCCTTTCGGTTCAAAACACAACGACAAAACACTTTCAAAACAAAACAAGACAAAACAGAGCAGATCTCATCAAAAGCAAAACTAGGCTCAGGTTATAAAAAAAAAAGAAGTATGCATCAACGAGCTGTGAATCTCTCAAAGTTTGATGGTAATCTTTTCTCATCATCCTCGCCTAACATTAAAAGAACAAAACAATAGAATGTAACGTATATAATAAACAGATCCAAAGCCTCAGAACTTAGAGGATACAACTCATCAAGGAGAAATCTCAGATGGTACGGACAATCTCACACATCACAGGCT

>TCONS_00081573

AAAAAAAAAATCCAAGGGATTGTGTCATCTGATAATGCCCCCTCACTTGATAAAAAACCAGAGACCTTCCCCTCAAGGGGTTGAAGAAAAAGAAACATATGACATGAAAACAAATGTAGTAGAGTATTATGATAGTTCGATGAACCTTTTGTCCCCAAAACCACCTGAAAACGACTCCTCCTACTCACCTCCGGCCAAAGTCTCACCGGCGTCTCTTTCCAGTTTCATTATCTACCCC

>TCONS_00063944

CAACAAAGCTCTCATCATGCCTATCTTTAAGGACTTGAATCTTTGACATTGAGTTGAGACACTTTGTTTCCCCTTATATTTTTCATCTGCTTAGCCAATTACTCGAGGGTAATTTTCCCAAATCGAAGTGCTACTCCCTGTCGTGCCCGCTCCTTTTTCCCAGCCTCCATGTCGCCTTTGGTTCCGACCTTGCTGATGTTGGAAGAGTCCGTGCTAGCATTTCCTCCTTGCTCAGTACTTGGTGCCTTTTTCCATTTTATATATATATATAGGAAACGAGCTTGGCTGTAATATCAGAACGATGCATCGCTTTTTCCTGTAGAGATGTCTCTTCCGTGAGGCGGCGCATGCCTCGACCTTGTGTCTGATCCTCATCCCCTAACCTTCCTGGCCGGCGTGGGGTGGTGATGTCGGCGAGACGTATCATCTCAAGCTAGTATGTCCGATCTTCCGCTCCTCCTTAGTCTGTCGTGCTAGGCAACTGATCCCTTTGTGCACTTGTCTTAGAACTCCTTGTTAATCCCTTCCTGGGCCTCTAAAATTCCTCCACACTACGGTTCAAGACCTTTTGTTGTTCTGATCTCGGCCGAAGACTCTGAGCTGTTCGAATTCTCTTTCAACACTGACTACAACGGGTCCAACAGAACCATAAGGAAAGAATGAACGATGGCGGAAATACTGATCCCTTAAGTTAATATTAAGGGACCTTAACCTGGCTCTGATACCAATTGAAACACCCGTCCCTCCCCATTTCCAACCGGGGTTCCAGGGCGCGGGGTTTAGGACACACATGTCTACTTTCCCGACATCTCCGTGTGTCCCGTTTCTGGTCCCATGAGTTTCAGGTGCGTTTCCTTTCTTACCGTCGACATTTCCACTTATAGTTCTGCAAAATGAGAGGGAAAAGGAGGGGTGAGTACGAATACTCAGTGAAGCGATTCTAGACCAGTCTCC

>TCONS_00052974

CGAACGGTCCATTCTCTTGTGGTGTGGTCTCTGGAGGGTTAGTCTCTTGCGATCCGCTCTCTCTATATGTGATTGTGGTTAAATGGGTGTAGACGGTCACGGAAGGTGGGGCTGGAGGCGGCATTCTCCATGTTTCCTTTCCGAACGACGGCATCGTCGCTGGTGGTTCTCTCTCGTGCCGTCGGAGGTTATTGCTGTCGAGTGTTATGGGTTTGTTCGTGCTCAGATTCTCTGTCCGGATCAGGTTTGGGTAGGTAAGTTGGAAGCTGCATGAGCAGTTGTTTCAAGGTTTGAGGGCGCCTTTCTCTTGGTAGCTCGTGGTGCCCGACTCCCGACCTCAACCAGCTCCGATTGCTTTGGTCTGTGCTCTTTTTTGTTGCTTAGCTGGTTCTAGGTTTGGTTCTACTTCTGTTGCGGGTTGAATCTTTTGTCGCATTAGGTTTTGATGTTTAGTTTAAATTCCGTAATCCGCTACTAGTTTCTTTGTAATTTCTGTCACAAACTTGAAAACTTGGTATAATATTTAACATTTTACCAAAAAAA

>TCONS_00052040

CCTGAATCTCCTTGATCTCTCCTAGGAGCGTCTCCTTACGTTGCGCAACATTCCCAAAAATCTCCTTGTTCCACTTCCTTAGCTTCTCTCTCAAGATCTCCAAAGCAGCCGGCGTGCTTAACTCCCCATTCCAAGATGCAGAAAGTAGCTCTTTGAACCCTTCGTGCTTAAGCCACGCAGCCTCAAATCTAAAGGGCCTTCTTCTCGGGTCTCCCCTTTTCTCTGGTTCCAGCTGCACATAGATGGGTGTATGATCCGATGCTAGGAACGGAAGATGCGACACCACCGCTTCCTGCCATCTTACACGCGCCTGAGCGTTGCATAGAATCCTATCTAACCGCTTGGCCACAAAATTCTGCGACTCCTTTCCTCTTCTCCATGTGAATGTGTTCCCTTTAAAACCCATATCCACCAAAGCTAACTCATTTATCCAATTCCCAAAAGCCAGTGAGTCTGGTGAGAGTCTACCATTCCCTCCTGTTCTCTCATCTAACCTCAAAATCGTATTAAAGTCTCCACCAATCAACACTGGCTCATCGATATTTTCCAACACCCGTTTTAACTGTCCCCCCAGGCCACTCCTGCGACTGACTGTAGGTGCAGCATAAACCGCAACAATATGCATAACCTCCATCCCAATTACTACCTTAGCATGCACGAACTGGTCTGACGCTTCCACCACCGTAATCACACCAGCTTGCTCCCTCCACAAGATCCAAATACCACCACTCTGCCCAATAGCATCAACACGATATGAGTGATCAAACCCCAAATTCTGACAAATTCTCATCGCTCTATTCCCTCCAGCATGGGTCTCAAATAGCGCAAGAAAGTCCGTATCAAATTTCTTTAAAATGTAACGAATAGATCTTCTGAAGTTGGGTTTATTCGCCCCCCGGCAATTCCAAAATAAACAATTCATCATTCTTGTAAGCTCAAGGTAACCACGAATTACCGGGGCAATCTGTCATGCAAGAGACAGAATCCTACCATCTCCTTGCAAGTCCGTCTGTACATCAGCCTCTCCCTGGTCCGTCGCACTGATATTACTTTCCATCTGGTCCTCCACCTCCTCATCTCTTAACCTCAACGGTTTTTCTATAACTCTGGCAACCTCCATATTGTCTCGGAACTCACCATTCGATCTTCCCGCAACCGAGCTTTCCACTCGCAACCTCTTCCCCGACTCTGAGAGGCTAATCTCCCCCTTCGTTGGACCAAAAACCAAACCTCTAACGGGCCTATTAGGTAACTGCTTAGGCCTTCCTCTTACTCCTTCGCTCAACTTCTTATTATGACCCCCCCATCTCTCCTTCCTCACCCCTGTCGAGATGGTTAGTTCACTCTTTTTTCCTCCAAAGATTAATGTTTCCTTCTCCAGCAAGGTTCCCTTACCCTGCTTCTGCTGAATATTTGTATTTTGATTCTCCTTATTCCCCTCACCAGCCACTTCCTCATTAATTGATTCCTCCATATTCGTAACTATCTCCAGGCTCCCATATTTATTCGACACCACAATATTCTCTGCAGCCTTCAGTTTTGGGGTCTCTCTAGGACTTCTATGAGCTCCTCCGTTAATCTCACCAACTCCAGCGTTTACTTCCCGAGATGTTTGTGTTCCTCTCCTCCCACCCCGTACTTGTGTAAACCCATCTTCTTGCCTTTGTTCAATCTGTTTAGTGGCCATTCTCTCCGTCGTCAATGCTAAGTTCGCCACTCTTTCAGATATTGTCTTCGGGCATCCATGAACCAAGTGTCCGTAGATACCACACTTAGAACAGATTTCGGAGAGGCCTTCATACGCGACAAAATACCTTTCACCATTGATTAGTACTGTCCCCTTCAGAGGTTTTGCAAGATTCACTTCCACACAAATCCTTGCAAACCGAGCTCTCTCAAAATTCAGTGTCGTAAGATCCACACGTATCGGCGTACCCAACCCCTTCGCAATCCCCATGAGTATCGATCGGTGGTAAAAGTTCACTGGTATATTTGATAGTCTGATCCAAACGGGAGTTGTAACAATTTCATCTCTTAGAGGGTCAAAAACTGGCGACCAGGCTCTCACCATGAGATAACTCCCAAATATCCTCCATGGTCCTCCAGACAACGCCGCCAAATACTCCTCCTCCCTCTCGAAACGAACCATAAAGAATTGACGAGGTAAATCCATTACGTACATTGCTCCCTTCGGACTCCACAATTCACGTAACTTCTTGCTCAAGGCAGCAATCGCAACATCCCTCCCCAGCACCTTGACAATCATGCACTGCTTCCACATCCCATTCATTGCTTCCAACACCTCCGGCTCGATCGTAATGGATGGCTCTCCCTCATCTCCATTCGGGAACTCCACATGAAGCCTCTCCGTCACGAACGCATCATCAAGCAAACTCTCCGGTATCGGCATACCTCCTCCGTTCATGCCCACCACCTTAGACGCATACGACGCCCCCGCATCTGGCGGTTCTCCTGGTGGTCTCACCCTCTCCCCCACCTCGGTCATG

>TCONS_00016368

AAATGGAAGATTCAGTGCTTCAGAAATGAGGATTCTGCTCCCGAGAATCCAGAGCCGGAGGAAGTAGTTAAGAAGCCAGATCAAGATGTTCAGCAGCCATGTACTGATCAAAAACCTTGGAACACAACTCTCCAAAAGGTAAGCATCGCCTCAAGTTACTGAACAAGAGAATAGGGTTGCAGCAAAATCTCATCTTTCGCACTAAACTCTTATGTTTCATCATCATCCACTCGATCTTTGTTTACTAGTCTTTCTCAACAAAGGAGTTTGAGATTTCTTATCATTTGGAAACTCTTACGCCTTGGTTTTATTGAAATTTATGGGATAATGCAAATTAAGGCGACTCTGAAAAAGGTCTTGTGTTCATAACATTATTATTATTGGGTTTGAAGTAAACTTTTGTTTCGTTGGGGTTTCAGGCTGCAGATGCAGTTTTGAAGGGCATTGGTACTCGTTGGAAGGTACCATGGACTGCCGAGACCATTGTTCAGGTACCTTAATGCCTCTTTTTTGTTGCCAATTTTGGGTTCACACTTTTACACTAGAGCATTCCTACTAGAACCTTTACTTGATGGAGTTGTATATGAATGAGTGATATTGAGTCTGCGTATGACTTAGAAGTAGCACCTTTTAACGCCGGCTACTATACCTGACTAGAAACTCTGGATAGGACTACATCTTGCCTCTGAGCCTGTATTAGTTGGGTTTAGTTTTTGAAAGTAAACTTTTGAGTTTGAGCTAACAGTACTATAGTTGCCGTGGTGCATGTAGGAGATGAAAAACAATTAGTGATGACAAATGAGTTAAATACCAAAGCTTTCAGTTTCACTCAGATTAATTCCTTAGTTAAACTTGCTATTAACTCTCATGTTCCCCAACTTAAACTTGTTCAGGTAATGCTACTATGGGTAGCCGCCTTCTGGTTCATAGGATCATGGATGATCCCATTCATGGCTCATATATCAGGTTTCCACAAGGACTCTCTCACGTTTAGAGGCCAAGCTTTGTTCAGTCTTATAACCGATGTAACAGAAGGACTAGCCGGGATTGCCATCCTCCATCGTTGCCTCTCCATGTTCCGTCCACTTGCAAGTGATTGGTTCCGCTTTACCCTCAAAGGAAACTGGCAGCTAGACGTCATCATAGGCTGTTTCATGTTCCCTTTCGTTAACCGTCTCTCCCAATTAAACCTGAACCTCTTGCCACTCCCACCAACCTCGAGTCCTGTCTCGCTCTCTAGTGTGGAGCAGTCGATAATGGCAAGAGACCCTGTCGCGATGGCGCTTTATGCGGTTGTGGTATCCGTTTGTGCACCGGTTTGGGAAGAGATAGTGTTCAGAGGGTTCTTGCTGCCGTCTCTGACTAGGTACATGCCGGTTTGGTGTGCGATTCTGGTGAGTTCTGTTGCTTTTGCGTTGGCGCATTTCAATGTGCAGAGGATGTTGCCGTTGGTGTTCCTTGGAGTGGTTTTGGGATTGATATTCGCGAGGTCAAGGAACTTGTTACCTTCGATGCTGTTGCATAGCTTGTGGAATGGCTTTGTCTTCATGGAATTGATGCGGTGATGAGGAGGATGACTATATCTCTTTATGTAGGGTGTCTGTGTCTTTTACCCCTTATTCTTGTAATCGATTCATGTTATGATTCTTAGATATTTCATTAAATGGACAGTTCAACTGTAAAAAAAAAGAAAAAAATTGTTAAAAGGAGATTCTCCCATGTTGACCTCACCATGCTCTTCTTCGTCTCTCCTCTGCCACCGCCCAGCTCTCTCCATCTCGCGCTCCAAGTTTAGGCTTCCTCGTCGAGTTCTAGTCTCCCCTGCTCTCACCCACGTTTCTCCTCTCACTGCTTCCCCTTCCAAGTCCACCAAGCAC

>TCONS_00010243

GCAGAGGATTATACATATTATACGAGGACGTGAAGTCATGTCCATACGAAGATGTTCATGTTCTGTGGTCTATACTCGTGGAGTCTCACCCACACACTTACCCAAACAATAAAAACTCCATTCATTATTGATACGTAGCTTTCGTCTTGGTTCAGCGAGTTTATATATGGAATGTTCATGCTCAAGGACGAAACAAATGAGAAACTTGTTAGCGACATGATTCATATATATGTTTTCTCTCACTTGTACATAATAATATAAATACACAGAGATTCTCTTTCCCATAAGGCCTCAGGCACTGTATGAGAAATGAGGTGGCTCAGTTCCTTCTTCTCTCCCTTGAAGAAACTATGGGTTCGCGTCAATTCTGCTCAAAAGAAGA

>TCONS_00069227

CCAGGCTTGACATTCACTTTCTGCGTATCGAACTAGTTCCAATGGATCTCTGTCTATTCCCCTGAAAAGTTTATCATTACGAGCCTTCCAAATATACCATATTATCCAGGGATAAGGATCCCTGTCTTGGTCTGGTTCTAAGATTTCATTCTTCCTCCAGAATAGATAGTCCATGTTCGTATAGACGCTTGCCACTGGAAATATACCTTGGCTTGTAGGAGTTGCTGATAGGGACCATACTTGGAGTGCTGGAGGGCATTCAAATATTGCATGTGTTACAGATTTTTCTAATTCTCCACACCTTGGGCAATAATTATCGCACCTCATATTCCGCCTTACTAGGTTCCTCGTTACTGCCACCTGACCCGTTATCAGTTGCCATATA

>TCONS_00050531

GGAATATGGGCTGGTATGATCTTTGGAGGTACCGCAGTTCAAACAATAATCTTAAGTTTTATTACAATGAGATGTGACTGGGAAAAAGAGGTAAATTGAGTGCCTGCATTTGGGGCTGGAGTTGGACTGGTGGTTCACTTTCTAGTGTGTTGGCTGTTCGTGGACGGGCTAAAACTAGGTGCCGTCGGAACTATGGCTACGGTGAGTATCTCTTGGTGGGTCAACATTCTTATTCTATTAGCATACTCCGTTTGCGGCGGCTGTCCACTCACTTGGACCGGCTTCTCCTCCGAAGCTTTCACCGGACTTTGGGAGTTTGTCAAACTCTCCGCTTCTTCAGGCGTCATGCTTTGGTACCAATTTACTATACTATGTCTATGTGATACTATTGACCAATGTTGTACCACACAACTACCTAACAGTAGATATTTGATCTTTTTGGGGCAGTTTGGAAAACTGGTATTATCGTATATTAATTATAATGACTGGGAATCTTCAGAATGCTCGAATAGCTGTTGACTCTTTGTCCATATGGTAAGTCCGTTTCTTTTACATTTTTCAGCCGGAAAAGTCATCAAACATAATCCGTATATGACCTTATTGCATCATTTTAGGTTGTTGCGAAAATTTGTATAATTTAATAATCATTGTTATACATACTAATTTTTTTTGGCAGCATGACGATAAATGGCTGGGAGAATATGATTCCTCTTGCTTTCTTCGCCGCAACCGGGTAAAATCCAATTTTCTCGATTGCTAATTTCTAAACCGGTTTGTGATCTAACTTGATCCGGTTCCGTTGAGTCCACTTTAATTCAACTAATTGTAGGTTGGTTGTTAACAGGACCATCAAATTTAAAGTTATAATGAATCGCGAACTGTTTTCCCATCCTCCAATTTTGGTGGTTCGGAATAATCCTGAAAAAAAACTCAAAATAGTAAGAGTACGTACTAGGTTATATAGTGCTTAACACAAAAATAAAGGAAATTAATTCTTAATTAGAAGTTTAATTACACCTTAAAATATAGGAACAACTGAGTCATATGATTATAATAACTATTATGTTTCAACAACAAGCTGAAATTTCTCTCTTTTTAGGAAGCACAATCACTAAAGTCTGAACCTCGAAAATGTGATTTTAATACTATGAACAAACAAAAGAAGGTCATATTAATGGTATCTGGTCCTTCCATCACCAAAAGATCGACTGAATTAAAAAGGTGAACTAGTTGTTAGACAAGACAGCTAAAGAAGTCTATGAAATCATTCACAACCTAACAAATACTTAATTGATGTATAATTCAACATGACATTTTTATTTGTTTATGTTAAATGTTGCAGTGTTCGTGTGGCAAATGAATTAGGAGCAGGTAATGGAAAAGGAGCAAGACTTGCGACGATTGTATCAGTGACACAATCATTAATCATTGGATTATCTTTTTGGGTGATAATAATGCTTTTCCATAACCAAATCGCATTGATATTTTCTTCAAGTGAAGCGGTCTTAATGGCTGTCAATAAACTCTCCATTCTACTAGCTTTCACGATCCTTCTTAACAGTGTCCAACCAGTTCTTTCTGGTAATATTTTCATTTTAAAAAATTTGAGAAAAATCTTTATATAGTGTATCAACGTTTTGTGTTAATTTTCGGACTCATCGTAGGTGTTGCGGTTGGATCAGGATGGCAATCCTACGTAGCATATATAAATTTGGGATGTTACTATTGTATTGGGATCCCACTTGGATTTTTAATGGGTTGGGTTTTCAATTTTGGCGTCATGATGAGAGGAAGAGGACGAGAAGAAGATGAATCGGAGTCGAGGGTTCCACTGTTGGAAAGTCCTCGCACGATGGAGGAAAACGGTGGAGGATTGAGGAAGAAACTTTGGGTTGAAACAAAGAAGCTATGGCAAATCGTTGCTCCGGCAATGTTCACTCGTATAACGACATATTCGACGCTTGTAATAACTCAAGCATTCGCTGGTCATCTCGGAGATCTCGAGCTAGCTGCAATCTCCATCGTTTATAACGTCGTTGTTGGCTTCAACTTCGGCCTCTTTGTATGATACTTATTTAATTGTAATACTAATCATTATTATATTTTCTGATATGACAATACCTAACATGAACGCGGTGCAGCTTGGAATGGCGAGTGCGTTGGAAACATTGTGTGGACAAGCGTTTGGAGCAAAGAAGTATCACATGTTGGGAGTTTATATGCAGCGTTCTTGGATTGTTCTCTTCGTTTGTTGTATGTTGTTGTTACCGACTTATCTTTTCACAACTCCGGTTCTCAAATTGTTGGGGCAACCGGACGATATAGCCGAGCTCTCCGGTGTCGTGTCCGTTTGGGTCATCCCTCTTCATTTCGCCTTCTGTTTGTCTTTTCCGCTCCAGCGTTTCCTCCAGTGCCAGCTCAAAAGTCAC

>TCONS_00073036

GATACATCCTGTTGAAACTATATAAGGAATGGCCAATACACAATTAAATCTGGATATTGGGTGGCTCAAAATTTATTAACGACAGCGGAAGAAAATGAAGTTTTTGAACCAAGCATCACTAAGCTCGAAGCCTTTGCTTGAAAGCTAAAGACACCTATGAAGATATGTCATCTTATATGGCAATTGTTAACTGGTCATGTGACAGTAACGAGGAACTTAACAAGACGCAATATGAGGTGTGACAACTACTGCCCAAGATGTGAAGAACTAGAAGAAACTGTAACCCATGCCATATTCGAATGTCCGTCAGCTCTCCAAGTTTGATCATTATTATCAACTCCAAGAACCCCATGTATATTTCCAGTACCAAGCATCTACACAAATATGGATTACCTATTATGGAGGAAAAACAACATTATCGAGTCAGAACAAGATATAAATCCTTATTCCTGGATAATATGGTATATTTGAAATGTTAGAAATGACAAAGTTTTTAGGGGGTAGATAGAGATCCTTTGGAGCTAGTTCGACATGCATGAAGTGAATGCCAAGCCTGGTTTGATGCCAACGAAGTGGTACAACCAGTGATACAAGATAACAATATTGACGAATCCCAAGTCATAAGCTTGGGTAATATTTGCTTGTTAGATGGATCTTGGACATCCTCTGCTCACTTTAGTGAATGTGGATGGCTCTAGATGGACAGTTATGGGAACATTCAACTTATGGGGACAAGAAATTTCACTCGACGCAAATCAGGTTTTGCATTCGGAAGTAGAAGCACTGCGATGGGCGATGGAGAATATATGCTTCAACATTCAACATGCCAGAGCTTCGGGACAGACTCTAAGGAACTGATTATAATGATAAAGGAACCTCAGGCTTGGCCAAGCTTTGCGACAGAATTGGAGAGGATAGAGACGCTACAAATATGCTTCCCGGATTTCAACATCATTCATATTCCACGAGCGCGCAATCAGATTTCAGACTTTTTAGCTAAGACTGCTAGATCCTTCCATAGGGAGTAACATTTTATTGGTTGTTTTATTCCGATTTGGTTATCCAGACCACCTCAAACTTGAGTAATAGAATAGTCTTTCGACGTAAAAAAAAAAAAATCTTGTAATTGTACATCGAACGGCCTTCTTTATTTTGTATAGATACGAAAATAAATTTATCCTAGTCACTCGCATGAGTGTCTTTAACCATCTCTCGTGTATATGACAAGGCGCTATAACCCTTCGTAGAACCAATATAAATACCATATATTGTCTTCATACTGTCCATTACATGTCTATACTAGATTCATTTAATATCATTACCACCATTAAGTTCTTCAATATTTAATCTTGTATTTCATCAATAAAGTGGCTGTATTTCTTCATTTCATTTGTTAAAATTTGACTCAATTTTTACTTAGATTTTTCAATGGATTTTTCAAATATTTTTCGGTTGGGTGTTCCAATTCTGCCCCACTAAATTGGCTTCATCTCGATCTGTTTGTGAAGTTTTCATTTTGTGTTATCCACCAAAGAAGTCAATTTTTT

>TCONS_00075701

TTCCGACAAACAAAAACGACTCCGATGTTTTATTCCATTACAGATCCGATATATAATCTGAAAGCTTCACGAAAATTCAAAACACAGAAGAAAATTCAACACAAAGAGAACAAAAGTCTTAAGACAAAACGGAATCAAAGAGCTTTAAGCATATCCTCAGCACTAGAGTTGGTGAAAGCACCTCCTTCCTCAAGATTCAGCACCTTCACAACACCATCCTCCGCCAGAATCGCGTACCTCCTCGACCTAACTCCGAGC

>TCONS_00033727

TACGGAGTGAAGCGAAAGGCAGTTGGTATCTGGGGGTGCAAGGATTGTGGCAAGGTCAAGGCTGGTGGTGCTTACACCATGAACACCGCCAGTGCTGTCACCGTCAGAAGCACAATCAGGAGGTTGAGGGAGCAGATCGAGGGTTAATATCATCATCCTATGCTGAACTCGATATGGAGCGAGTATCAGGAAGCAGATTAAGAAGATGGAGGTCAGCCAGCACAGCAAGTACTTCTGTGAGTTCTGCGGCAAGGGCGAAGAGAACGAAGAAGGTTGGAATCGTCGGCAAGTACGTG

>TCONS_00032636

ACTCGGATACAACAGCTCATTTCCATTTATATATTTATTCGAAACAGCCCATGAAAGTCCAATTAATAAAATTTGCCACCGCTTCCATTATCTTCGTCATCTTGCCACGCGTTGGAAGATGAAGAAAAGAAGGACACGCGTCGTCTAAATCTTCTGCACGTTGCCAAGCCATCTCACGCGCTTCAGGAACGCGCCGTCGTGCAGAAAGAAGAACTCGCGTCCTGGTATTTTCCGGCTGATCGATAGTTCTTTATCTGCATATGTGAGCCCGTTGATCTTTGTTTGAAAGGGCCTTCCACCATTAACCGCGGCTTCTTGAGCAATATATCATGTTTCGCCTGAGATTTAAGAACAGGGGAAGTTGTAGCCAATATGAAGAAAAACCATTTGCCGGACCGTCATAAACACCGACTCTTCTTGATCAACTCCATAAATGACTGCCAAATCCAACTAACCCTCTCGACTCCCAAATCAGAGATGCTTTCTTCAAGCCAGCCGGTTATATTAATTAAGAAAGTCTGATTCTCCTAGGAAAGTGATAAGGAGGTCAACGATTCAGGAATACAATTTTGAACTGGGTGAAAAGCTAT

>TCONS_00067062

CACAACTTTAAAAAGCTTTACTCTTTATTACCTTTTTTCCACACCCCATTTCCTGTGAGAATGAAACTTAGCATGCGGGAATATAACTATACGGCTTTGCTTCTGAAAAAACGACACAAAACTTATCTTTATATGACTCACACACATTTATACAAGTCTTTGAATCTGTGTACGTATATCTATCTATTTTACAGTATATATATGTTGGTTGACATCAGTTTGAAAATATAATAAGCAATATGTTTCTTGCTTCAAATCTTCCTCAAAAGCTTGTGTTCTTGATCTTGGTTTGTCTCGGTGAGAGTGAAGATGATGACGCTGGAAGCTCTTGTCCTGGTTAAGTCACGTTGAGGTCATCTACTCCATTGATGAAGCTAAGTGGGGAATAAGCATTCCTGGTCCGAATTGACTTCACTTTTAAATCTTATTACATATGTCTTTTATAATAACTCAATGTTGTTAGTTAAAGTTGGTATGTTTTAATCACTTACGTGTGTGTTTAACTCACTGAATGTACGGTAGCATTTTTATCATAAAGTAATCAAAGGCTTATGCTTCGGAATATAAGATTTCACATGAATTTAAATACGTCTAGCTAGAGATTACATTATAAACAACAAGCTTCC

>TCONS_00009394

CACCTAAGACACTTTATGACTTATCAATTACTTTTGGAAACACAATTAACTTTGTACACACATTTTACATGGATAAAGACACAAAAACCTTTCACCTTAACCAAACTAAACTTTTTCAACTTTCTTCGTTGTATAAACAAGACCAATCGAGACGTGCACACTCCCTAACACGTGTAAAAACCTTTGACTAATGTAAATCTAATCCCACACAAAGTTCAATCGTCTTCATTTTTGCTCACCCCTAGATTCTCGATGCTTCCTTCAAATCCAGCAAAACCTTTGTCAGATCTAGAAGCGAGATTGTGAGATGAATCTAGAAACGACACGATAAGGATCTCAGTTAACGACGCTGCAATTTCAATGGCAAGTGTGTTGGGAGAGGGATGAAAAAGTTGGAGGACAAGAAGAGACGAAGACGACGAGCATGTTGGAAGCGGTGATTGCGGAAGCGGTTGAGGATGTGAAGAACTGGAAGCAATGATGAAAGATGCGGAGGAGAGCTTAGAACTGAGGTTGGGGCGATTGCGGATGCGGCGGTGGAGAGGTTAATTAAGAGAGTGACAAAGAGTGGGTTTCAGGCTAAGAGGAGAGCTTCTGGGATGGGAGGGTCTGAGGGGCTTCCTCCTTCCCCAAAAGATATGTATGTTGGTTTTGGATCAAGATAGGCTCTGGTTGTTACTCTGTCCACAGTCCGCTCGTCCACGCCTTTGTCAAAAGCATCAGGTACACACATCCCATGTACACAACTTGTCTTTATCCAACTCTCCTATTCACACCTCCCATGTCCACACACCTAGACTAAGAACAACTTCATGTCTATATTTTGTTGTTATGGTAAATGAAAAACCAGATTCATAAGTCATCCGTCCACAAATTGTCTATCCATAACTCACATGTCCACATAATTAGAACTCATGTCCAAATATTGTTTATCCA

>TCONS_00009579

TTTCCAGGTGATGGCCTTATTATTAATTTCCCTTGTGAGCATGGAATACATGTAATGCTCATAGGGATAGTTATCCTATCTTTCAAGGAATGGCCAGTTGAGCTCGAGATTATTTTGCGCATCATGGATCTGCCAGGATGGCCAAGCCTTTCATGCCATTGTTTAAAGGCTTCTCTGAACTCATTAGGAATTGAAGTGTTAGCCTCACTCGATTTTATATTGGCACAATAGAGGCCTATTGATATAGCAGCA

>TCONS_00034595

GTATTTTTAACGGCAAAAGTGGAATCCTCATCTTGAAATTCCTCTAGAGTATGTGAATCTCGTTCATCTTCGACGATCATATTATGGAGAATGACACATGCTCTCATAATATTTCCTATTTTACTTTTATCCCATAAATTAGATGGATTTTTAACAACGGCAAACCTTGCTTGGAGGACCCCAAACGCACGCTCAACATCTTTTCGGACGGCTTCTTGGTATTTAGCAAATAACGAATTTTTTGGACCCTGTGGCAGTCGGATAGATTGAATAAAAGTCGCCCAATTTGGATAAATACCATCCGTCAGATAGTAAGCCAACTGGTACTCCCTTCCGTTCACATAGAAGTTAACTTCCGGAGCTATTCCGTTAATAATGTCATCAAAAACAGGTGATCGATCAAGAATATTAAGATCGTTCATAGTACCTGGAGCTCCAAAAAAAGCATGCCATATCCAAAGATCATACGAAGCAACCGCCTCCAACACAATTGTTGGTTTTCCGGTTCCTCGTGAATACATTCCTTTCCAAGCGGTGGGGCAATTCTTCCACTCCCAGTGCATACAGTCGATGCTGCCCACCATCCCGGGGAATCCACGTTGTTCTCCCACATAGAGTAGTCTTTGAAGATCCTCTTGTGTGGGACGTCTGAGGTATTCATTCCCAAACAAGTGGATTATTCCGGCGGTAAATTGGTGTAAACATTTTCGTGCAGTTGTTTCACCAAGTCGGACATATTCGTCAATGCCATCAGCCCCACCCCCATACGCCAATTGACGAATTGCTGCGGTACATTTTTGGAGCGGTGATAGACCAGACCTTCCGGCTGCATCTTCTGTTGGTTGAAAATACTCTACTTCTGTAGAGAGACGATGCACAATACGCAAGAACAAGGGCTTGTTCATTCGGAACCGTCGCCGGAATAAATTGTGCGGGTATGTCGGAGTGTCGCTAAAATAATCATTCCAGAGCTTGTTGTGGCCTTCTTCCCGGTTTCTCTCAATAAAAATACGTTTTTTTCGCTCTTTTGGTTCAGGAATAAAATCGAGATTTTCAAAGATATCCTCAAAAATAGAATCAAATTCATCATCATCACCGTTGTGGTAATGATAATGTGAAGAAGAAGCCATTTAAATGTTGCAAATGAGAGAAAATGTTATGAGCGTGAGGAGAAGTGCAAATTTAAGTGGGAGAAGAAGGATGTTACTTATAGGGAGACAGTGAAGAAAAAAACAAAGCATGTGACTTTGGAGTTGCTATTCTCAACCCCC

>TCONS_00019674

GAAAGATGTAAGGTCTAGAGTCTTGCCTTAATTCCCTAATAAAACATATAAGGTAGCTAGATTTATCGGTTAAATCGCTTGTTTTTGACAAGCTAATGTTTCATGACTCAAGTCACATGCGTATAATATATCAAAGGTAAGAGGAGGTGACAGAAGAGAGTGAGCACACATGGTGGCTTTCTTGCATGCGTTTTTAATTAGGGTTTTATGCTTGAAGCTATGTGTGCTTACTCTCTCTCTGTCACCCCTTCTTTCTCTCTCTATCTCTCTCTCACACATGATAAACCATAGCTGATAAAAGATCTCGATTTCGTTTGATTATCTTTAAGGTTTTGTACTTATACTATCATCAACTGGGTAAGACTAATTTGAAGCTCGAAATTTCTCATTAACAAAAAATAATAGTTTGCAACTCTGGTTTTAATTGCTAAGCTATTTTTCTCATTTATGTGTCAATTATTTTGTTTCATGAGGATGCAGTTGGACTTATACAAAGGCCTATAACTAGGTGCGGCCAATCAACTAGGGCATACACTCAAATATTACACAAAGAGAGACAGGACTACAGAAAGTTTGAGAACTTGTATGCCGAAACAAACGATCCGGACTGAGTTGCAAAAACGACATCAAAAGTTGTACTTCATTTCTCATAAATATAGAAAATTTGAAACATAAGAATACAGAAAATTTGGATTGATTAGCTAAGTGATTAAAGATGTGAGATATAGTTTATAGCCTGT

>TCONS_00053434

GCCGAAGGGTCACTTTGACCAGTCCGCAAATTGAAGAGTCTTTCTTCTTTGTCTTTCAAAATTCTAGTCAAATGTCTTTAATTTTATTTGTCTTTCAAACTTCTAGTCAAGTAGCCCCTTTGTCTCCTACTTGTACTATAAATAAGTGTTTCATTTCATTAATAAAATCAGATCATTTTGGATTAAGTTTCTGAGTTTAAACTCTCTGTTCTCTTTAGAACTTGTTTTCGATTGTCTTGGTGAGTCATATCCGAGCAATTCACTTCTCAACTAGTTGGTCTTGTGAGTCATATCAAGCAACCAGTTCGAGATCTTCCTTGGCGGACTTGTGAGTCATATCAAGCGCCATTGAAGTCGGGAATATCAAGGGAACATCCGCAACCCTTGTGCGACCCAACGTTCCATCAGTTCTCCTTTCCGGAGTTCATATTCAAACGAATCCAGTCGAGGGCAATCCGATCTTAGGG

>TCONS_00038592

CTTTCCACAGACTCAGACTTCTTAAAACACCATCTCAAAAGCATTGCCCGAGAGAGAGAAGAATCAAAAACGTAGAATCAAATCAATTATTAAGCAATGGCATCGATGAGCATGACAGCCACATTCGTCCCGGCCGTTGCAAAGGTCCCGTCAACCACCGGCGGACGAAGGCTCTCAGTGATCAGAGCTTAAACGAGCGACAACACAACCAGCTTACAAGTCAAGGAGACACAGAACAGCACCACGATGAGGAGGGATCTCATGTTCACAGCTGCAGCTGCGGCAGTTTTTTCCTTGGCCAAGGCAGCCATGGCAGACGAGGAGGAGCCCAAACGAGGGACAGATGCAGCGAAGAAGAAGTACGCTCAAGTTTGTGTCACAATGCCGACAGCCAAGATCTGTCGCTACTGATTCAACAATCCATCTTTCATGTCTCTCTCCCTCTATCTTTCTAATATTTCGGTAAAATTTTCTCAAACTTTGTTTGTAATCATATACTAAATCAATTTCATATTTTGCTACTAGATTTTCGAATATAAACTAAACTTGACAAAAGCATTGCATAGTGTCTGTCTACAATAAAGTTGCTGAAAACTAGAATAAAGCCTTGTACCATACTTATTCAAAGTGAACACAAGCTAAGAGGAGATATTGGGTTTAACCACCTGCATACGTATAGACAAACCTCTCTCATCCCACGTAGTATCCCCAAACACAGCTCCCGTCTTATCACGAACAACGCATTCACCTCTCTCTCCCGGTTTGAACGAACAGCCTCTCGATGTCATCAGAGGAACACTCAACACGTTTCTGTTGATCCCACGTGGAACAAAAAGCACATCCCTGATCGTCGTCTTCTTCTTCCCTCCCTTCATCACAATCTTCACATCTCCTCTCCCTTCCGCCACGATAACCGTCCCGTCCACCAAACCAACCTTAGCCTTCTGCGTTCTGTCTAGACTGGTGAAAAGCTTCTCATGCGGAGTCATGTTGCTCGTGGCGTGGCCGTATATCATCCACGTATCCTCATCGTATGTGAAGGCACCCAACTCCAACTTCGCTAGCAATATGTAGTCAACTACCAGTTCTGTCTGATTCTGAGCTTCAGTTCCCGCCCGTTTGAAGCAGTGTCTTCCATAAATCCCTTGTTTAACACCCCAGTCCCCTTCCTTAGGGATGGAATGACACTCTTCAAGGTTATGATCTTCCTTGTAGCACACGTCACACCACTTGCCAACACCAAACATAGCTTTAAGCCTGTTTCGCTTAATCTCTCGATGAACATATTCATCTGGCATCGCATCAAATCTACGAAAAAACTCAGCGAGGCTCTCGAGGTTCATCGTCTTCAGATCCATTAGCTCTTCAATCACAGGAGCAGCTTCCTTGTACGACCCCGAGAGCGATCCCAAAACCTTCTTCACAACCTCGTAATCAGATTTCTGCAACTCTTCGACGATCCGCGAGACCCTAGCGAAGTAGGAAACGATCGATTCCTTTTCACACATGCTAAGTTCCTCGAATTGGTGCTCTAGCCTGCGGAGTTTCGCTTCTTCGTTACCTTTCGCGAGCGAATCCCAAAGCTCCTTGGCGGAAGCAGCCGCGATGGTCTTCCTGAAAGACGAATCCGGGAGAGAGGCTTGCAGTATCTTCAGCGCTTTGATGTCTCTCATCACGCGGTTTCTCCAGTGAGCGAGATCCGCGACTTTGATCGTCGCCGCTAACTCTGGAATCTTCGTAGGATCGGGAGAGAGGCCGTTTTCAACAACCTCCCAGAGACCTTTCTCCACGAGTGTTGTTTTCGCGGTGGGAGCCCACTTTTCGTAGATTAGGTCACCGGCAACACCGTCTTCTGCATTCGCCGCCGCCATAGCTGTCGTAACACTCAGGGTTTCCTAAAACTTTGTTTGTATATTCTCTTGGAGAGAGCGAATCGCAAATCGCAATGCACGCGTCGCTTACTATTTATACTTACAATAATGACATTAAACCAAATCGTTAACCCTCTTTCGGTGTACGCATTTATTCAAGATAAGCTTGGGATATTGATTCGGTTTAGCGTAACTGGAGGATATTTTCGGTTTAGTTTCTCCTCTAGCCCTGAGCGGGGTTCTACTTGGAATTCAAAATTTAAAATTTCTGGATTTTCAATATCATGCACACAGGTTTTATTTGGTTTTTCCAAATTTTCAGTTCAGTTTCGCATTATACTGTATCTAATAAAATCTTTGAAACTTTTAAAAACTGAAAAGTATCTATGTTAATAAAATTGTCAAACTATAACACCAA

>TCONS_00071406

GATGAGGAGAGACACCGTTGTTTGCCCTAAACCACGCCGTGTCGGTGTACTCAACAACCATCACCTTGCTCGATCTCTTCGATGGCAAGTCAGTCAGCAGATGGATCTACGCGAATCGAATTCACGAGGCAAGATATTGGATTTCATCCTCGCTAATGGTAATAGTTATGAACAAGATCCGAAGATGACGTCATCGCCGCCGCTGTTCTTTACAGGGTCGCCGCCGACTAGAGTTTCTAACCCTTTAACAAAAGACTTACTCTTTCGGGACGAGCTTCTCGTGGTGGCTCCTCCTCCGTCCACTCCACGAGCAACCAAGCTACCACCGCCGTCGTGTCCTACGGTTCGAGTGAGTACTAACTTCGGGAACAATCCTACGGTTCGAGTCGTTGGATTCAACTGCCTTGACAGGGACAGACGCAGCAGCGTTCCGACTCTTGCATAAGAGTCTCCCAAAAACGCCGAAACGAGAGATAAATGCAAATATATAACACAGAAGGGGTAAAAAGAAATTTAAAAAGGTTTAGGAAATGATTAATGATGTGATCATGTTGGGTGTCTATTTTGGTGGAAAATATGTAAATGTCTTGAAAGAAGAGGGTAATTTTTCAAGGGGCTGTTATATAATTTTTGGTTTTGAGTGTCCAATTTGGGAATTTTTGTAAAGTAAAAAGGGAGAGAGAGAAGGGTTTGTGAGTGTTTGGTGTCTTTAGTGAAAATAAGAGCATCACTCTATAACAAAAGGAAAGAGTTTTGAAATGTGAATATATCATTACTCCATTGTATCTTATGTACATACGTAGTTATCAAATGAATTGTAAAGTTTTAAATTCTTATTTAGTCCCTTTGTTACAAAATTTGGTGATTAGTGGTGTACAG

>TCONS_00027686

GGAATATGACAGTTTCTTTGTCATTCTAATCAAACCAGAATGAATCGCGATGTAAGAAGCTTGTTTTTGATACATAAAGTAATGGAGAATGACTAGGAAGTTGAATAAATCTCATAGGAGTTAGGATGAAGAAGCTGTCCCACTTTCAAATCAGGTGATCCCAGTTTTCTTGTTTGGGAATATGACAGCTTCTTTGTCATTCTAATCAAACCAGGATGAATCTTGATGTAAGAAGCTTGATTTTGATACATAAAGTAATGGTGTATCACTAGGAAGTTGAATAAATCTCGTAGGAGTTAGGATGAAGAAGCTATCCTACTTTCAAATCAGGTGATCCAGTTTTCCTGTTTGGGAATATGATAGCTTCTTTGTCATTCTAATCAAACCAGGATGAATCTTGATGTAAGAAGCTTGATTTTGATACATAAAGTAATGGTGTATCACTAGGAAGTTGAATAAATCTCGTAGGAGTTAGGATGAAGAAGCTATCCTACTTTCAAATCAGGTGATCCAGTTTTCCTGTTTGGGAATATGATAGCTTCTTTGTCATTCTAATCAAACCAGGATGAATCTTGATGTAAGAAGCTTGATTTTGATACATAAAGTAATGGAGAATGACTAGGAAGTTGAATAAATCTCATAGGAGTTAGGATGAAGAAGCTATGCCACTTTCAAATCAGGTGATCCCAGTTTTCCTGTTTGGGAATATGACAGCTTCTTT

>TCONS_00027868

CATAATCGGATCTGTGGTGGTGGTGATCGGTTTATACGTATTTTTATGGAGCAAGAGCAAACAAATAGATGATTGTGAGATCAATAAGTTACCCACAAATACCGTGGAAGCAGGAAAGGAAGAGGAAGATCATACAAATGTTAACAAATTAGGCAATCTTTTGGTTATCCCCATGACTCCTTGATCAAATCCTTACGTTCAACTAGAAGGAGCGACATGCCATCTTGAAAAATCACTGTTCGAAAAGCTAAATATGAAAACAAAAGTGGGATATTGCATCGTTCTTGGAACTATGATAAGCTTAGGAGGAGCTTTAGTTCTTACGATGTACCAAGGCATTCCGTTAACAAACTCTCAAGAACAAGCATCGACTTTAAACCATCTCACAAAACATGCGCATTGGACCAAAGGTTGTATATTTTTATTCATATCAGTTATGTTTTTCAGTTCTTGGATGCTTATACAAGCCAAAGTCAACGTGAATTACCCTTGTCCATACTCGAGCACCGTTATTTTATCTGCTTTCGGTACGCTTCAATGCGCCCTTCTTAGCTTGATCAAGACTAGAAATGTGGAAGAATGGATCCTCAAAGACAGACTCACCATCATCACTATCATTATAGCGGTACATTCATGTATATACAAATTGTCATCGATATGTGTGAATATATAATTTGGTGATTCGACCTAGTTTCGGGTTGAAAATTTTCACTACTATTTTAATTACGGTTGGCCACATCTTTAATAACAAGAATATCCGGTGCTTCATATGCTCGAGGTTTAGTCTAGATTTCAGTTTTGGATTCAAAATCATTTTAACATTAATAAACTGGGTGTACGTATATTTATAGGGAGTGGTTGCACAAGGAATGTGTACGGTGGGAATATCATGGTGTATTAAACAACGAGGACCTGTCTTTACCTCAGCATTTACTCCTGTCATGCTTATGTCTGCAACCTTGTTCGATTTCTTGATATTTCATCGTATGATTTATTTGGGAAGGGCGAGCTTGGTACTATATTTCTACTTACTCGGACTCTCCTATACTTCTGCCACATTGGGATCTGCCTTCACGGCGATAATGCCTGCCCTTACTTTCATTATGGCTTTGATATTTAGCTGGTCTCTCTGCTTCACTACGGGAACAAGTTATCAAAGAAGATATAGAACATACATTATTGAGAAGAGAGAGAGAGGGGCAGCTAGAGAAGGGGCGCTTGATTAAGTAATAACTTACTGATCATGGGTTATATCGATGGAAAATGGGCACCGCTGATCATGATGACTGTGATCCACATGATTAATGGGATGGTTAACGCTTTGATTAAGAAAGTTCTTGATGGAGGCATTAATCACATGGTTATTGCTACCTATCGTTTGGGTATTTCCACCTTTTTCCTTCTTCCGATCGCTTATTTTTGGGAACGGTAAGTCTAATTTTTGTTTTCAATTATGTTTTTCTTTTTTTTTTGTCAACAATTATGTTTCTTATTTTTTTCATTATTTACATTGCGTTAGCTGTTAGAAAACTCAGGAAAGTTTTTAAAATTCATCTTTATTTTTAAAGTTAATTTCAATGCTCCATCAGGAAAACAAGGCCAAAACTTACCATAAGCATTTCGTGCCAGCTTTTCATCAGTGCTCTTTTTGG

>TCONS_00042206

TCTCTCTCTCTTTCTCTCTCTCTCTCTCTCTCTCGATCTGATCTGGTGAAGTGTATGGCGCACCGGCATCTGATGAAGCTGCCAGCATGATCTAATTATCTTTCTTTCTCCGTTGACGATGGAAAAGACATGAGTGTTGATTAGATCATGTTCGCAGTTTCACCCGTTGACTGTCTCGCCCTTAAACCCTAATTATTAATCTTCAATTCTATTTAAAATTTCACAGTAGGCTCTCTCTTCTTCAGTGCTTGATTTTCAAAAGACCTGTGTTTCTATTTTTTCTGATTTCTGAGCTTAATTCTCTTTTCTGTGGCTATATTTCTGTTCATCTAAATTTTTTATTTACTACTCCTGTCATTTCATTACCTGTGTGTTTGTGATGGGTTCAATGTTTTTGTTGTATTTTAATAGGGCTATAGTGTTAATTACATGATTTTTTAAGTTCTATTTCGTCCAACTCATAACAGATTGGGATGATAGTATCTATTTATTTTTTGGGGATCTTAAGAGAGACTTCCAAATTCTTCTTTTCCACTATACTGTTCTATTTATTTATTTTGTTATAATGGTTTAGGGTTTCTGCGAAGTTGAAACTTTATATATCTCCCAAAAACTATATAAGTAAAGATTTATGGACTTAGTCACTTCTTGCAACTCAAACTAGTTTTGGATTCTAAGTTTTAGATTTCAAGACCAATGAACTTTCATCGGTTTATATTCTATAGCAGAAATGGTGTCTAACCTATAAATTACTGCTGAGCAAATCTAAGCTTATGCTTTAGAGTCTTTCAGCTTTGGCCTCAGAAAATGAAGTATGTTTTATGGGATTTGAATTCTTTCATGGATCCAAGATTTGTGTTTATTGAACTGGAACACATGGTGAGTAAAATGAGAGAAACTAAAGAGCGGTTCAGATTAAACTTCAAATAAAACATTGGACTATAAATTCTACTCTAAAGAAACGTATGTCAATTTACTGTTTCATTGCTGGAAATAGGTGGCAAATGGTCCCCTTCCATGTATTAAACATACCCATAATACTAGTTAATATTTGTCTCAAG

>TCONS_00027585

AAAAAATAAGGTTGAGAGAACAAACGAGAACGGCTTTATGAGCGGGAACAGGAGGTGAAGTGGCGGAGTCTTCGGAGGCGATGAGAACAACATCAGTGAAGGACCTTGAAGAAGAGCTGTTACCGTGGTCGAGAGAGGATGATAAGCGGAGGAAAGTAACTTTGGCTTTAAGGTCTTCGATCTCGCGTTTGAGCTCTTCCTCAGTCTCTCCCGCCTCTACGTAGCATTCCTTGCAGGTGCCTGCGTCGCGAGGGAGGTACTCTTCCCGGCACGATATGCAGCGCATCGACGTGTCCGAATCGGATTCGGATCCGCTCTCGCTGCTCTCGGATTGGACGCATCGGTGGGTTCTCCTCATCTTACACGACGGCGATTTTGCTCTCTCTCTT

>TCONS_00048168

GTGGTGGTCGTGACTCGTGAGCAAGAGCTGGTGTTGTGCAGAGGAGATGTCTGGAACTCGTCTCCGTCTAGACTCACACGAATGTTGCAGTCTCTCACTGGAATTGCCAAGTGCAGCTTTTGAAGGTATTGTGACAGTAAAGACTGAGACTTTCTTCTTCTCAGAGTTTTGCTTATGTCTGCTGATTCTTTTTTTTTCTGTAAAACTTCATTCTTAATTATAAATAAAAGACCCAAAATATTTTACAAATCTCTTAGGCTATAGTAATAAGACCCATAGTTAAAAGCCCAAAAGAATCACATATAGAGCCCAGTTGATATTGGTTGCTAGTTCTGGCCCATCCCGATTTTCTTGTTGCTTAAAATGGCTCGCCATCCTAATTTTGGTCGAAAAACATAGAATCTTTCGGTCAAACCTCTTGGTCTCTCTGATACGAAAGTTTTGTTCTTTTTGGGTTTCTGAGTCCACAAAGGAGATGATGAAACTGTCTCTGTTTGTCTCTCATTTTTTTTCCCTTTATGTTTAGTTGTGTTGATCCATGTTTGAATGGTTTGTACTTTCTTTTTTATAAGGTGGACTATGCCATAGCGTTAGAAATTGATATAAGACGGCCAGATTAGCTACTCACTCAAAGTATTGTTAAAATCTGCCCAAGATGTGCGTTTCACTAATATTCTAATATTGGTGCAGGACACTCCTAAATGTTGCAGCTGAGCAGCCAGATATGGAGAGGCACATTTTACTTGACGTCAACACGCTCTACAAACAATCAGACACTCTCCTTAAGTTGTCCCATATTCAACAGACAGGTCATGGGTTCAGCGAATCACACTCAAGTTCGTTTATGCTTTATATGGTTTGAGTTGAGTTAAAGTTCGGTTCTTTAATTATGTGTGTTGCACAAGTCCAAGAGGGATGACTCTTCCAAAACTATGTCCCAACGTTTGCAAG

>TCONS_00038729

TTAAAAGAAAATTTTCCTATCTTTAAAAAACTATTCTTAAACAAAGATGAAAAAATCCGTCCCCCTTCCCGACACATGAACCCTAAATCCCTATATTCCTCTATTCTCGAAGCTCTTACGTTTTGACTCAATCCGCCGAGAATCGATCTTGTTCTTATACTTCTCCGCCGAGAATTTGAATCCCAGAAATCTGACGAACCCCTAATCCCCTTTCTGAAATTCTGGGGAACACTAAATCACAAGATGTTGTCCCAGGTGGAAGTCCAGAATGGCTAAGAACCGTGATACTTGCTGTTGTTATTGCTACTTTCATAAGTCTGACTATCGTATACCTCCGGTAATGCTCTATCTCAAAGCTTTGTTTTGGAAACTTATCACAATAATTAGTAAACATTACTTGAAAAATTTATGGTCCCCAGGTTCGAAAAGGTCGATTGTTACTTGCACTATGCATTATCACTGACCTCTCTGTTGCTCTTGTATCTTCTGGTTTTCTTCTTGCATTTACTGAGTATACTGCAAGAGCAGTAAATGTAATAAGTAGTCCCTCTCTGTTGTCTCTCGTTTCATTGGAAGTTGTTTGTTTTTGGGGTGGCAATTGTTTAATTCTTTTAATGTTGCTAATGTGGTTGTTCACATGATATGAAGTAGAAGACTGGTCTCTCTTGGAAAGTCCCACGGAATTGCAGTCTTTATTCACTTCGGAGATATGATAAATCTATGTATGAAGAAAATAGCGAGCCACCTGGAGAATCCCAAGAGTGAAGTGTGTATGTGTTTACAAAAGTATATAGAGATTTGTTTTTGTTACATGACCAAGAAGAAGAAAGCTGCAAGCCAAATTTTGTAAAAAATCATCCTGATCCATTGTTCTAAAAAAAGAAACACAACACAAATGTCTTTTTTTTGTTGCGCCAAAAGAGCCCATTTTTCTTCTGCAGCACTCTTTGTCTCGTGTGATCAATACCTCAACAACACTCTTATA

>TCONS_00080683

CGAAAAGATAAATCAGATCTTTTTCTGCTTGTTTAGTTAGCGATTATAATTATTGACTACTCAATCAAATTTCTTTTCCTTCTGTTAATTTGTATAGTAATATATACTCGATCTCTTTAGTGTGTGTGTAAATCTGATATAAGTTCTCGACGGATCCGTGGAAGAGAGCTCATCAGTCGTTTTCTGTAGGTGCAGCACCATTAAGATTCACATGGAAATTGAAAAAAACCCTAATTTATATTAATTAGGGTTCTGATATGTATATGAGAATCTTGATGATGCTGCATCAACAACCAACGGCTACCAAACACCTAACGCTTCAGAAAGAAATGATGTCTGGATCTTCGGTAATGCGATGATCAAGAACAACACGACATTGACCACAAATCGAATACTTATGTCATTTTAATGTTTT

>TCONS_00018414

ACGAAATTGGGGGATAAACATGGAGAAATTGAGCTGAAAGACAAAAAGATAACAAGAAAATATTATGAATGTTGGGGATAAAAAAGAAGAGGTGATGATAAAAATCGATTTAAATTATAATGGGTCCCGTAATACAGCTGGTGTGGAGAAGTACAGAAGTGTTCTTTAAGGAACGGAAAATTATAATAGTTGAGTGGGGGATGATCAGACACCAAGTACGATTTTATATCCAAAAACAAGATTGAGATCTGGTCTTTGGATGGAGTGTAATAATGCCATAATAGGGACGTGAAATTTTTTTGTTGAACTTTTAGATATTTCAAGCGGAAGCAAAATGATATTTAAATAAGGTGAGTTAACGTCGTATTATTTTTGGGATTAGGAGCTGAGATTAGGATGTATTACACAAAATTAGGCATCACGCTTGGAAGTATTGGCAACTAAGAGCTGGAGTTGTGCATGCATTATATGCCAGTAACCTTCACCGATGCCGATGACTTCTCCGCCATCGACATTACTCTCCAGCACCATCATAAGGAATCAATCTGTACTTCTTCAGGCATTTCTTCTTCAGATTCACCAGCTTCTTTAGTCTAGATAAACCACTTAAGCCTCAACAACTCCGAAACAAAATATTTGTTAGAAAGTTTTCAAACAAGAAATCCTTAAGTCTACTTACCCTGTCCCAAAAGAGGTAATTTTATACATTCTTCTAATCAAAAGGCGTAATCAGATTTTTTCGGTTATGGACAAACCTACTAGAACATTCCTCTAGTCTTCTTCTTCTTCAAATCTCTCTCTCTCTCTAGATATGGTGACGAACTCCACAATCAAATTTCTATGTAGCTATGGCGGGAAGATTCTACCTCGTTATCCCGATGGCAAACTCCGTTACAACGGCGGCCACACCCGCGTCCTCGCCGTCCCCCGCTCCGTCTCATTTTCCG

>TCONS_00008002

GTACAATGCCAAGCGCAGGCACTGGCGCAGAACCAAGCTCGGATTCTAAGTGAAAGCTCGAGACTGTTTGTCTTCTGTTCGATTTAGCCGTCCCACAAGTCGTTTATGATAAAGAAGAAGCTGGCCAAGAAGATGAGGCAAAACAGGCCTATTCCCCACTGGATTCGTCTTCGTACCGACAACACTATCAGGAGGGATTTCTCAACTGCTTCTTTGTTTTATTTCATATGGTTATCATTGAGTTTTTGTTTGCTTGATATTCATTTGTGGCCTTAGCTGGATCCCAGTGACGATGAATAAATAAAAAATGTTAAAAGTAGTGACTGATTAAAACCATGACGAATAATCAAATTCTGAGGGATCCAATCCAGCTATTGTTTTCATCTTTTTATGATAGCAAGAATGTTGTTCGGTAGGTGTAGTTATTAAGATAAGCTTTATGTGCTGATGTTCATAGTGATGAGTTATGATTATTTGTTTCCTCGAGTTTCATAATGATTTACTGATTGCTGTTTCTTTAATCAGTTGTTTGTCATCTATTTCAGTTTCCATTTGTTTTCAATCTGTTGACCGCATTGTTATTATTAATTTTTTCCAACATTAAGTATTTTCTCTGTTCTGGCCTGCGACAAATGAAAATCTGGACTCCACTGGTGTGTATTTTTAAGCTTCAGTTGTGTCAACACAGTCTGTTATGGAAGACCAGACCAAGGACTCGGTTGCATGGCTTGATCAGAGGAAAGGGTCTGTGCAGAGTCGAGTCCTTATTGTAGTCACTCGCCCAATAAAAATTCTGTTTAAATACATTCTTAGTCGTATGGTTGTAGTATTGATCCGAGTGTCGTATTTAAACGATGGTTTCCTGTAATATTAAGCACTGTAACTACGAGAAAGTAAATGTACCTTGAACTCGAGATAAACATGAAACCATTCCTAAATAATTGCCTTGAATAATTGCAACGAAACTATATGACAGGAGACATCTTACCCATGAACCAAATTTTTTAAACAATGTATAAACAATAGTACTATTTTATCTGCAGCAGTTCTAAAGAAATGTGACTATCTGAATTATATAGGGTAGAAATTCTACTTTCACAGACATGCCTACAGTCCTGCACATTGATGGTATATATATATATATATATATCAAAATCACTATTATCATGATAACAGCTAAATAATAGAATGGGCACAAAGAAGAGACTAACTCACAGTGATGAATTTCTGTCTGCATATTAGAAGAGGATGCGATGACGATGATGACAACATGGATTTTCGTTGTGAAGTATTGAAGTGTGGTTTGTTCAAAAAAAGTATTGAAGTGTGATAGTTTCTAGATATATTATGCCACAAACTAAAAAGTAAACTACATATGAAACGGGCCTTTAGGGCTTCGTTTTTGCTCGACTACATAATGGGCCTTGATCCGAAAGAAAGTTCGTTTTGAAAACTTTCATATGGTAAGCCCAGCCTTTTTTACTTGACTAGGGTTTCCTCTTACTATAAGTCAAGAAAAGGTTGGTCGCCACTTCTTCTCGTTTAGCTTCGCTCCTCCCCAGCTGCATAACTCACGACTTCAAGATG

>TCONS_00046283

TATTGCTTGACCGCCCTCGTGACCGAGTCTTCCGTATCCTCTTGTATAAATCAAACCCTAATCACCTCTTCTCTTTCACAAATCGAAAAACAAATCATTCTCAAGTCTACCTCTCTCTCTCTCTCTCTCTCTCTCTCTCCCGAATTACGATCAAAGATGCAGATCTTCGTTAAAACTCTCACCGGAAAGCTCCGACACCATCGACAATGTTAAGGCCAAGATCCAGGACAAGGAGGGAATCCCACCCGATCAGCAGAGGCTTATCTTCGCCGGAAAGCAGCTCGAGGACGGTCGCTGACTACAACATCCAGAAGGAGTCGACCCTCCACTTGGTTCTCAGGCTACGTGGTGGTATGCAGATCTTCGTCAAAACCTTAACCGGAAAGACAATCACCCTTGAAGTGGAGAGGGAATCCCACCGGACCAGCAGAGGTTGATCTTCGCCGGCAAGCAGCTTGAAGACGGCAGAACATTGGCCGACTACAACATTCAAAAGGAGTCTACACTTCACACGTCAAGGCCAAGATCCAAGACAAGGAAGGCATCCCTCCAGACCAGCAGCGTCTCATCTTCGCTGGCAAGCAGCTTGAGGATGGTCGCACCTTAGCCGACTACAACATCCAGAAAGAGTCCACTCTTCACTTGGTGTTGCGTCTCCGTGGTGGCTTTTAAGACTCTCGTCTTGTGTCGTATCATACAAAACAACAATGTCTCTTTTTCTTTTAAAAACTTCTCATGGTTTGTGCGTGTCTGAGGCCTTACAAAGTGCCTTATAAATAATCAATGAACACTGGTTTGATTCCTTTAGTTTCTCTTGGAATTTCTCGATATTGGAATTTATCCTCATTGCTATCGTTTGTATCTGCATTTATATTATAAAAGTGCTTTGTTGTTTGAAATGTTATGCCTTACAGTGAACAAGAGAAGATGCGGCGGGACATACTCAATGGGTTGTGTAGCTTTTTACTTTTGAAATCTTACGTCTCTTGAATTTGTTCGGAGATACATGAGAAGCTTTATTGCATTGTCTACTCAGTCGTCGTCTCTTCATTGTGGTTAATTGAGAGATGTTGTGCTGATCATTTAAGTGGGTTGCTTACATTGATGAGCTTGGAAGGGTAGCATTTTGGTTTACTCGACTCATTGCTGACTGAAGTCAAGAGCTTCGGAGTGAGCTCAAACAAGTCCGAGATGAACCGTGAACGTGATCGCCAGGTTGCACAGTCACAGAAGCTGGCTGATGAGATACTCAAGTATTAAGAGAGTGTTGGAAAGTCATCTAATGAACTAGACATTTTGATTGTAAAATCAGTATCATTGGAGGAAACATGTTCCTCGCAAAAAGAGCGTATAAAAATGTGGGAGCAGGAATTAACTTTTGCTAATGGTGTGATCCGATTGGTTCGTTACATTGACTTTTAATGTGCTTTCTACCGATGGTAGATGCGTCTATGTCTCTTACTATGACGGAGTTTGAGGATAAAATAAAACATATGCATGAGCTGCAAGACCGCCTTGCAGACACGGAGCGTCAACTTTTCGAAGGAGAGGTGTTGAGGAAGAAGCTTCACAACACCATTCTTGTAAGTATATATAATTTAGCTACATGTTAAGTTCCAGCAGCTTTCTCTATGTGCGCTAAACAAAGCTTTTATTTCAGGAACTGAAAGGAAACATACGTGTGTTCTGCCGACTGCGCCCCTTGCTACCGGATGATGGAGGACACCAAGAGGCCAGCGTCATTGCTTACCCTAGATCATCTGAATCTCTTGGGAGAGGCATTGATGTGGTCCAAAGTGGTAAATTCAGTGACTCACCTAATTTACTTTGCTCAAGTAGGATATTTCGTTTTTCATGCCTTGTGCTCTATTTGGGTTCTCAGGGTAACAAGCATCCTTTCACACTTGACAAGGTTTTCGACCATGGAGCTTCTCAGGAGGAAGTATTCTTTATCGCAACTTGTTCAAAGCGCATTGGATGGCTACAAGGTTTGTTATCTTCTTATTCTGACTATCTTGTAGATCCTTTGGTAAATGCAACTATTTGTATCTGACAACACTTTGACTGATCTTTCTTTTGTCATTTGCCTGCCTTAAATCTTTGTGGACATAAGTTTGTATTTTTGCATACGGCCAAATAGGTTCTGGCAAAACCTATACCATGATGGGCAGGCCTGAAACGCCAGAGCAGAAGGGACTGATCCCTCGTTCGCTTGAACAAATGTTCAAAACGAGCCAGTCTCTTAGTGCACAAGGCTGGAAATACAAGAGGCGTGTATCTATTTTTCCCAGAATGGTGAAACATTTGTTTAATGCTTTGATTGCTAGCGCTGTTTCTTATTGGATTTTTAACATAAAACCCCCTCAATTAAGTTTATTTCGGGTATAAACCCCTAAATTAAAGATTCAACGGAAAAACCCTCAACCTAAGAAATGGTAACGAATTGGCCCCTCTGTTACTTAGCCACGTTAAAATTTTGAAAACCTCCGTTAGTTGAAACACAT

>TCONS_00017954

ATGTTGTCTCACAACTCGAACTAGGTCATAGTGGAAATCTGGATCTATCTGTGTTCTTTCATCCTGCAAAGACATTTCTATTGCACGTTCTTTCCAATAATCCTCATCGTATTCCTCTGTATGTTCCTCGTTAGGTGAAGTAATTACAGTGTCAACTGCAAAACTTTCATGGAAACCACTGTCTGCCCAACTGCCTATGGAATAATCATCATGTCCTCTCCTGGTTGGAGGTTGGAAGGCAAAATGTTGGTAACAATGATTAGGTGAAGCGAAATGGTCAACTGGGTGCATTACGTCGAGTGACGTGTTGTTGTGGTCATCGGTCACCATTGACACATTGGAATCGATCGATGGAAAGGTTGGGGTGTTGATCGATTCCGAGTACTCTGTTTCGTACTCCGATTCGTACTCTGCTCCACAATGGCATGCTGCAATGTAGCCAAGATCATTTCCAACATCCACGAGGTTGAC

>TCONS_00068667

CCACAGGGTACTTCACTGAACGATCAGCCAATACCAGAGAAAGTCTACACTTCTTGTACTGAGTGAAACCCAGCTTCTTAGCAACAGATAGGGGCATCAAGCTGACACTAGCTCCCAAATCGCAGAGACATCTATCAAATACCATAGGCCCAAGAGCGCAAGGTAATGTGAAGCATCCAGGATCTTCTAGCTTCTTTGGAATGACCAGCCTCTGAATGATAGCACTGCACTCATGAGTGAGAATCACCATGCCCTCCATCTCTTTCTTCTTTGCAGCTACAGCATCTTTGAGGAACTTGCTGTATTGAGGGATCAGCATGAAAGCATCTATAATGGGCATTGTGACTTGGACTTCACTCATTTGCTTGTCAAACAAAGCTTTGTACTTCTCTAGCAGCTGCCTCTTGAATCGACCTGGGAATGGAAGCTTG

>TCONS_00045021

CTTCTCCCCTCAATTTGAACCGAAGAGAGAAGACAGCGGGAAACTATATATATATGTCTCGAGAGATCACTTCATCTCTTGCCGCCATATCTTCACAAGCTCTAACACTCACTCAACTAAACCAATTCCATGGTCAGTTCGTCATCTCCAACTCCCTCCATCGACAAAGCTACTGGGCCTCGCGTCTCATCGCCTCCTGCACGCGCCGCCGAGCGCCGTCTCACTACGCGCGGCTCGTTTTACGTTCAGTGGCGTTCCCCAACGTCGTAGTCTTCAATTCGGTGTTTAGATACTTTTAGCTGGTGGGTATGGCGAACGATGATCTTCGTCTCTACCATCAAAGGTCTGGATGATGGATATGTATGGGAAGTATGAGTCAGTGGGAAGTGCACGCAAGGTGTTCGATGAATTTACTCAGAGAAAGGTTTCTTATTGGAATGTGATGGTTTCTGGGTATTGGAGGTGTGGGAATAAGGAGGAAGCTTGTAAGCTTTTTGATATGATGCCTGAGAGTTAAACAGATGTGGTTTCTTGGACGGTGATGATTACTGGTTTCGCAAAGCTCAAGGATTTGGAAAGAGCAAGGTAGTGTTTTGATCGGATGGGTGAGAAGAGCGTTGTGAGTTGGATTGAAGGCGATGCTTTCAGGTTATGCACATGATGGGTTTACAGAGGAAGCTCTGAGGCTGTTTAACGATATGTTGAGACTTGGAGTTAGGCCAAATGAGACCACATGGGTCACTGTTATATCCGCGTGTTCTTTCCCTTCCTCATTCACTGGTGAAACTGATTGGTGATAAGAGAGTCTGTTTGAATTGCTATGTGAAGAGGGCGTTGCTTGATATGGCATGCTAAGTGCAAAGATATTCTATCTGCTAGGAGAACTTTCAACGAGCTAGGCGCTCAGAGGAATCTTGTCACTTGGAACGCTATGATTTCGGGACATACCAGAATGGGAGATTTGAGTTCGGCTATGCAGCTTTTTGATGCAATGCCTGAGCGGAGTGTTGTTTCTTGGAACTCGATGATTGCTGGTTTTGCTCATAACGGACAGCCTGCACTGTGCAGAGTTTTTCAAAGAGATGATTGACTCTGGAGATTCCAAACTGGATGAAGTGACAAGGTTACTGCTCTCTCGGCTTGTGGACATGTGGGGGATATGAAATTAGGTGATTGGATTGTGGATTACACTGCTAAGAACCAGATCAAGCTAAGCATTTCGGGATACAGGTCTTTGATCTTCATGCACGCAAGGTGTGGAAACTTGAGGGAAGCAGAACGGGTCTTGGAAGAAATGAATGAATGAGATATTGTCTCTTATAATACGTTAATCTCAGCCTTTTCAGCTAATGGAGAAAGAGCTGAAACGCTGAATTTATTCTCAAAGATGAAAGAAGAAGGCATAGAGCCAGACCGTGTGACTTAGACTGATGTCTTGACTGCTTGCAGCCGTTCAGGATTACTTGGGTCAGAAAATATTCAAATTAATAAGAAATCCATCGGCGGATTTACTGGGGTGAGTTGGCAAGCATAGTGAGACAAACAGAGTAATTGAGAGAATCCCCATGAATGGGACTTTCAGGAGTATTTTCATAGGCCTAATAAGAAGGCAGCGGCATCACATCCCAGGCTGAGCATTTGCCACTTTGCTGAAACAAAGAAACACTTTGTGAAGGCAAAGATAACCTCCTTTTTTT

>TCONS_00051125

CAGGATGAACCGAGAATCTGGACTTATGAGCCTCCCTCATAATCTCTTCCTTTAGACTTCAATCATTAGGAACACTGATCCGACCGTTTACTAGGATGGTACCATCCTTTGCAGTCTGGTACTCCGTCCTATCATTCTGAGCAACCTTATTCAGGTTCTCGTCCTGACCTTGAGCCAACCGGATTCGAGTAAGCAGGTCGGCTTGGTTAACCGCCTCCAACCAAAAAGCTTCGGTTGC

>TCONS_00018232

AGTTTTTCCGCTCAATCGATATCTTGAAAAGAAAAGTTTTACCAACCAAATTGTTGATAGCACCGGGCAAAAGATATGGTTCTTGGATCTCCATAAGAAAAGTAGAAAAGGCCATTCTTACATCAGTCGCTCAAAACTCAAATGAATTATTTAAAAAGTTATAAAAGAGATTTATAAGCAAACCTCGTCGGATATGAGTCCGGTGAGCTCAAGGCAAGGCTGATGTAAGAGCTGATTTGCTAGATTATCAAAAAGTAGGAACTTTGGTGTCACTTGTATGATCGAGCACAACCACATGTAACTTATACCTACAATATATGAATAGCCATTACTTGTGTACATGTCGTCAAAAGCAAAATTATAAATAGAATTATAATCAGATTATAAATAATAACCTTGGAATAAGTTGGGGGTCATGCAAATTGCATTTCACGCAAAAGTAACTGTGCCTGAACACATTCACATCGAGTCCATCATAAACTTAGAGAGCAGGAATAAATTTAGTGAGCAG

>TCONS_00004633

GAAACTCTTAACCTTATCTCAGTTTCCCTTTACCCATTAAAAAACGGGGGAAAACCGGCGGCTGCTACTCCTAGCAAACACAAAAGCCAATTCCAAGACGGTTCATCATCGACGGCTCCGACTTCTCTCTTTTCTCAACGGCGTTCTTACTGGACGTGAATATGAAGAAACCCTAAACATTGTGACAGGAGGCCTCCTAATTGGCCTTGAGATGCTGTTGAAGCAGAAGCGCAGAAGAGAGAAGTTGCTGCTGGTGACTTATCTCCCAGGTACCGTTTTCACCTCAATTACGTTCAGCTTAACTATGACGTTTGAATCAATAACTTTGTGAAAAAAAAGAAACGAGTCTGTGTAGTGAAATTTCCGCGATGAGTAGTTTGATTGATGAAATCAGCTTGGTCTGATCTGTAAATACAAATTATTTGGTCGTCTGTCAATTATTACAAATCTCTTAACAAAACAAAGTTCCAATTCGTTGTGGGGTTGGATGATTAAGAAAAAGTAGTCGCTGGTGATGCACTTAAGGTGTTAGTCCTGTATTAAAAAGCTGTAACTTTTGTGTGCTGATGTAATCTTTTAGTTGCAGGTTGTTAATTTTAGAACAGAATTGTAAAAAACCCTTTTATTTGGTTTCTGTATAGTGATGGAAAATATTTGATGCCGGTTACTTTGTGTCTCAGTAAGCAAGCTACTCGATAGTTTCTATCATAGTTTTCTGCAAGATCATGACCATAATTACGGCCAGGGCCAGCGAAGCACAAGAACATGTTAGAGCTAGAGTTCTTTGAAAAGCTATTTGTTCATTACCTTACAAAAATTCCTGATTGGTGGCTGCGGTTTACTATCTTCTATGTTTCTGTTTACAGTTAATCACATGTAAGACACAAAGTAAAGGTCCGCAGAAAAATACGATATCCTGAACTTCAGAAATAGTACGAAAAGGTTCACAACTACTCATCAGAAA

>TCONS_00078608

CGTAGCTTCACTCCCATCCTGAACCACCGTGATATTACTCTAATCGGCCATCATTGCTGATGGGACAACACCCTTCGCGTCTTCTCCATATGAAAATTCCCCATCTTATAATTCGTCTCTTAAGCGAAGGTGAGGGCTTTTTTCCATAATGATTTGTTATCTTTCCATTCGACAGAGTTTAATGTTAAACCGAGATACCTACGAGACGAGCTATATAAGTTGATTTTTATTTGAATGTGGTTTATGACTATACTTAATATATATATACAAAGACATGTATGCTTGTCACACTGTTTTACTCGAATGAAGTTTAAATCGTATAAGGATGATCTTTAGGGTCCAATAGTGCCCATTTCATGATCTTTCCTCGCAATGTTGCGAATCTGACTGAACTTTGGTTTAATTATAAGTTTATAACTAGATTTTGATCTGCACGACCTATGATTTTTTTCTAATAATCAAATATTTTATGTTATACAATAAAATATATAAATAGATTAATATAATTTGGTGTTATATATTATCTAATTTTATAATAATATTTTTGTGGCGTATAATATTATTACTATAAAATTTCTAATTTTGTATTTTTGTAAACAAAGTTTATTTTGAAAACATTATTAGTTAAAAATACTGATT

>TCONS_00014375

CCTCAGTCGAATACAGTCTCTTATAATATTTTGTAGCTAGCTTTTCCAGTTCCTCGGACTGATCCACCCAGCGGCCATCCTCATCCTTTAGCATCTCTATCTTATTTCTCTTCCGCCGTACTATTGTAGTTGTGTGATAGTACTTTGTGTTCCTGTCGCCCAGGACTATCCATTTTTCACGCGATTTTTGATACCACAAAACCTCCTCCTGCTCGAGGACCACATCAAACTCTTTCTGCAGTACCGCCTCCTTCAAGAGTAAATCATCACATGGATTTCTCTCCAACTGCTCCTGAATCTCTTTAATATCTCCTAAAAGCTTTTCTTTTCGTTGAATAACATCTCCAAATATCTCCTTATTCCATTTTTTAAGTTTGGATTTTAGAGATACCAATGCCTCCGGAGTACTCAGTTCACCATTCCAAGATGTCGAGAGTAGCTCTTTAAAGCCTTCATGCTTGAGCCACGCGGCTTCAAACCTAAACGGTCGCCTCTTTGGGTTCCCCCTCTGCTCAGGTTCTAGTTGCAGGTAGAGTGGTGCGTGGTCTGATGCCAGGAACGGGAGATGTGAAACCGCTGCCTCCTGCCACCTTACTCGCGCATGGGCGTTACATAAGACCCTATCCAAACGCTTCGCCACAAAGTTCTGCGTCTCCTTTCCTCTTTTCCATGTGAATTTATTCCCCTTAAATCCCATGTCCACCAATGCAAGCTCATTAATCCATTCCCCAAATGCCAGAGAATCTGGTGAAAGCCTACCATTACCTCCAGTTCTTTCATCCAATCTCAAGATAGTGTTAAAGTCTCCACCAACCAACACCGGTTCATCAATATTCTCCAATACTCTTTTAAGCTGCCCCCAAAGCCCACTACGACGACTTACTGTAGGAGCAGCATAAACAGCGAGGAGATGAATGCTCTCCGTCCCAATCACCACCCTAGCGTGAACAAACTGATCCGACGATTCCAATACCGTAAGAGCTCCCGCCTGATCTCTCCACAATAGCCAAATTCCACCACTTTGACCAGTGGCATCAACCCGAAAAGAATTATCAAATCCCAAATTCTGACATATCTTTCTCGCTTTTTCTCCACCCGCATGAGTTTCGAATAGCGCCAGGACGTCAGTATTAAACTTCTTCAAAATGTACCGAATCGAGCGTCTGAAATTGGGTTTATTTGCCCCCCGGCAATTCCAGAATAAGCAATTCATCATCGTCAATATGTCAAGGTATATACGAATTACCGGAGCCCCCTGTTATGCAAGAGAAAAGATCCCTCCATCTCCCTGCAGGTTCGTCTGTGTATCAGCCATTTTTTGTTCCGCCACGCTGATAGTACTCTCCATGGGATTATCCACCTCTTCATCACGCAGTAGCAGAGTTTTAGATCTAGTCTGACCGTTCTCCACGCTATTTTTGAACACACCCCCAGCTCTCCCTGTCGTAGTATTTTCCACTCTCAGTCTCTTTCCAGATTCTGATAGGCTAATTTCTCCCTTTGTCGGCCCAAACACCAGCCCTCGAACAGGCCTATTGTTCACTTTCTTGGGCTTTCCTCTACTACCCTCAATCAATTTTCTGTTAGCGGCCCACTTCTCCTTATGCGCTCCTTGTGAACTAGTTGAAGTACTAGCTTTCCCGCTAAAGATCAAACCTTCTTTCACTTGCGAGAAACCTTTACCTTTCCTGTTCTGAATATTCATGTCCTGATTCTCCTTATTCCCTTCAC

>TCONS_00017894

GTCCAATTTCCATTGATTTCTCCCAAAAGGGGGTTTTACAACAGAACATTACAACTCAAAATTGAGTAAAATTGGTACAACCATGCTTTGTAGTTACGAATCCGAAAGACGACACAGTCGAGTTGTGGCTAGCAACCCCGGGCCAGACATCAACTTCACCACTTAGCTTGTTAATCCAGGTTTGGTGCTTTCGCAACCAACCTCGCCTTCTTGCTGTAATGCTGTCACTGTGAAGTTCGCAATGGAGGAAGGGATTAGGGTCATACCAAAGAGCCAAATCCCGCTAAAAGTGGGTTTGCTGAGGAAGCTGTCATGCCAAGATGGGAGAGTACCTGCACTAAAAGAAAAGGTTTCATCCTGGCAAAGCAGCCATAAGACCGCTACACCACTGGGATGGATGAACACCAAAATACGCCACAACCGAGGAACTGACGGCTCCGACGACTCGCGAAGCAAGACCAGACTCCACACGTATTCTTACCCTAAGAACTAGAGTAAGTTCTTTACTGAAGCAGAGAGAAGAAGACTCCGTCGGAAGGAAACGGAGCCAAACACCACTTGAAGACCAATACTCGAACCAAGACACCGGGTGATGCGCAAAACCCGGAGGAGGAACACCATTAGACAGGACCGCAGGAGAGGCAAGACAAAGATCGGGAGAAGAGATGGAAATCCAACGCCTGAGAACCATTCCCCGAACGCCGGAACAGACCATATCCTCGCACACGACAGAGGGATTTGTCAAGTGACACCGCCACCAGAAAATACACCACCGCACGTCTTGGAGAAGAAGCGCCAAGCCCACCTTGAGAGCAGAGAGAAGGTGCGCCCACGCCGAGACACCAAGGCTAGGGAGAGAAACCTTCGACTGCTACACCTCACCAAGCTGGAATCAGAAAGATCCGGGCCGGAAGAGTCAATATCACTACCCACGCGCCACCACCGCCGAAGGCTACGACCAGATCTAAAAAGGAAAAAGTAGATCTGGCGAGACTCCACCGCAAAAGCCGACTCCAGCCATCTTGCAACCCTCTCCGCCTCGTCTCAGCTCCAGATCGGGCCTTAGCCACCGGATCTATAACCCCCCGGGAGGATCTAGGGTTCCGATTTCAAAGTCGTACCAGAGAGTTGACATGTCGAAAGAGAAGAGAGACAAGGGGATGAAGCAAAATAAAAACGAGATGTAGGAGAAGAAGCACCTCCGACGCCCGGAGCTAACGCACACCGACGAAACACCGGTGTCTCCGAACGTCGGAGCAAGGAGGAGGAAACTTTGTC

>TCONS_00009674

GTGAGAAGCAGCTAAGAACTGGTGTACAACTAAAAGAAGCAACCCTACGATAAGAAGATATTCCCATCCTCTGAGGCCGTCGATGCTTATCCACCGTAACTGAAAAGGATGGAAACAATTAGCGGTGGTCGGAACGTCGGGTCAAGTAGTGGTGGCACAGGCAGTAAGGAAATCAGGACCAACACCGGCGAAGGTACAGAGTTTGAGCTCTAAGTTTCTGTATTTGTTTTTACAATTGTGTGCTTCCTCTATTTGGCAAATGACCCTAACGGGTGCTCATGCTTGGACCGTAGGTCTGTGTAATTCTTGCATTGTGGTTATGGCTGGGAGGTGGATAATGTTTGACAATGGAGCTTGGGATTTCAAGATAGACAATGATAGAATGGGTAGAGCCGTCGATTCGTCCAGAATTAGAGGTGCTGAGGAGTTAAAAACGTGTATTCTAGCTGCGTATGGGCTTTTGGGAAGGGAAG

>TCONS_00037051

ATGCGGAGAGCTGACCAAGTGGTCTTTGTACAGAGCCGTCATAGCCGAGTTCGTAGCCACTCTCCTCTTCTTGTATGTCACTGTTTTGACTGTCATCGGCTACAAGATTTCGTCCGACACTAAGGCCGGAGGCGACGAGTGCGGAGGCGTCGGAATCCTCGGCATCTCATGGGCCTTCGGTGGCATGATCTTCATCCTTGTCTACTGCACCGCCGGTATCTCAGGTATTAATATAACGAACCAGTCCGGTTAATATCGGTTCTGATTTTTTAAAATAAACGTGCTCTTTGGTTTCTTGTTTATTTGGTATTGACCCCAAAACTTTATGAAGGTGGTCACATAAACCCTGCGGTGACGTTCGGCTTGTTCTTGGCAAGGAAGGTATCGTTGGTTAGAGCAGTGCTATACATGGTGGCTCAGTGTTTGGGTGCGATTTGTGGAGTTGGTTTCGTCAAAGCCTTCCAAAGCGCTTACTACGTACGTTACGGTGGAGGAGCAAACTCTCTAGCCGATGGATACAGCACAGGGACTGGACTCGCTGCAGAGATCATTGGAACATTCGTCCTGGTCTACACTGTTTTCTCCGCCACTGACCCCAAAAGAAACGCACGAGACTCCCACGTTCCCGTAAGTATTAATCATCAGTTACTTCCAGTTACAAGATATAGTTAAGTGAACATTTAACTAATTAATTTGTGGTTATGATTTATGAAATAAAAGGTGTTGGCGCCACTCCCAATTGGATTTGCCGTGTTCATGGTACACTTGGCCACTATTCCAATCACTGGAACCGGTATTAACCCGGCCAGGAGTTTCGGAGCTGCAGTTATCTACAACGAGTCCAAGCCGTGGGATGACCACTGGATTTTCTGGGTGGGACCATTCATCGGAGCTGCGATAGCTGCATTTTATCACCAATTCGTCCTAAGGGCCTCTGGTTCCAAGTCCCTCGGGTCCTTCAGAAGTGCGGCCAACGTTTGA

>TCONS_00026253

GAGATTTCAAGAAAAAAATCCAGAAGATAAAAAGATTCATCGTAAGGTCTAGAAAATGAGTCTTTCCAATAAACTGCATCTGTGACTTGTTGAATGAAAAAAACGAGAAATTAAAAGAAAAACACCAAAACTTGTCTAAGTGACAGACTAGTCCTCTCCTCTCAATCCTCCTCATCGATGACCTTAGTGCCGAGTCCACTAAGTCCTT

>TCONS_00038341

TGTCATTCTAATAGAACCAGGATGAATCGCGATGTAAGAAGCTTCATTTTGAAACATAAAGTAGTGGAGAATCACCAGGAAGCTGAATAAATCTCATAGGAGTTAGGATGAAGAAGCTATCCCACTTTCAAATCAGGTGATCCCAATTTTCCTGTTTGGGAATATGACAGCGTCTTTGTCATTCTAATCAAACCAGGATGAATCACGATGTAAGAAGCTTGATTTTGAAACATAAAGTAATGGAGAATCACCAAGAAGCTGAATAAATCTCATAAGAGTTAGGATGAAGAAGCTATCCCACATTCAAATCAGGTGATCCCAGTTTTCCAGTTTGGGAATATAACAGCTTCCTTGTCATTCTAATAGAACCAGGATGAATCGCGATATAAGAAGCTTGATTTTGACACATAAAGTAATGGAGAATCACTAGGAAGTTGAAGAAATCTCATAGGAGTTAGGATGAAGAAGCTATCCAACTTTCAAATCAGGTGATCCCAGTTTTCATGTTTGGGAATATAACAGCTTCCTTGTCATTCTAATAGAACCAGGATGAATCGCGATATAAGAAGCTTGATTTTGATACATAAAGTAATAGAGAATCACTAGGAAGTTGAATAAATCTCATAAGAGTTGAATAAATCTCATAGGAGTTAGGACGAAGAAGCTATCCCACTTTCAAGTCAGGTGATCCCAGTTTTCCTGTTTGGGAATATAACAGCTTCCTTGTCATTCTAATAGAACCAGGATGAATCGCGATATAAGAAGCTTGATTTTGATACATAAAGTAATGAAGAATCACTAGGAAGTTGAATAAATCTCATAAGAGTTGAATAAATCTCATAGGAGTTAGGATGAAGAAGCTATCCAACTTTCAAATCAGGTGATCCCAGTTTTCCAGTTTGGGAATATAACAGCTTCCTTGTCATTCTAATAGAACCAGGATGAATCGCGATATAAGAAGCTTGATTTTGATACATAAAGTAATGGAGAATCACTAGGAAGGTTAATAAATCTCATAGGAGTTAGGATGAAGAAGCTA

>TCONS_00014356

GTGGAGAAGCCGCTATACATCACCGGGGCTGGGCAGAGCCAGCAATCCACTATCAACGAAGATCGAGCTCTCTCTCAAATCAGAGAACACTCGAAAAAGAAGAGATACAGTGTTTGTTACGCTCTGTACAACTTGTACCAAAGCTCTCGGTTTAATCCTTTTATATGAATAAGGACTTAACCGGAAATTACAATCAACTTATATCAAACCAATATATACCAACATAACCAGTGGCTTGTGATACGTGACTTCTTTCTTCTTTTTGTTGACCAGAGCTGAAACACATTTCAACATTCTCCACCTTGTTTTAGCTCTCAACAACTTCATCTCTTGTGTTAAAACATCTTCGTGATCTCCTGTGGATTTCTCTGGAATCTACACCTCCAGCAACAGAGCCTTAAGCTCCTTCAAGCTTCTACACCCTTGAGCAAGTCTGACACGTCACTGAGCTTGCTAACCGGTAGACCTTTAGAAGCGCCATCAATGAATAGAACGTGCGAGACCTTCTCTGTGAACGTCAACTCCGCACTGATTTTCTGTGTCGCTAACACTCCAATCCTTTATTGAGTTATACACCAGCTTTAACACTGACTTGTATTTTAGCAAAACTCTCCATTGATTTCTTCATGCTGCTGTAGCTTGAATACTTGCGAGAACCACTCAGCAACTCCCGTTAACAAGTTACGTTTGTTTGATCGAACTTGTTATTCACAACAGAATTCTCCATAAGCTCCACCTACTCATGAACTCTCCCTCCAATCACACAGTATCCTCTGTAGTTTCTGTCAATACCACAGACCTGTTGCAGTGACTGAAACTCACTTATCCCTTTGACATCACCTTGCTAGCTTCTAGGTTTCTTCCTCATGCGCATGTAGACCAAAACCCTTTAAATCCACTAGAACCAGATGCAAAGAACACCAAATGTTGAAGTTTCTATCTTGAAACTCTTCCATTTACCCTGTAACCAATCTCACCAACAGCTGACACCACTGCAGAAACCTGAGATCCACCTTTACAACAACCTGTCTAACCAGATAACCTTGAACACTTTGATTTCTCAATCATCCTGTTAAAAACAGAACCCACTAGCAGCTTTGGAACCCACTACTAGCACTCATATACTCTGTCTCATATCCTGAGACAACTCCTTCCAGCCGCTTCTACATCACATCTAGAATCCATGATGACCTTGAGCTGTAACAGTATCCTGCAATACATTCTCCCATATGCAGTCTCCACCATAAAACTTGCTTCATGTATCTTTACCAGATACTACTTGACTTGAATCTCTTTATAACCAATCAGCTTCACTTGTATTGTGCCTCTGATACTTCTGAATTCATCACTTGACTGTTATGTAAGTGATCCCTGAGATGATCCCTAAGCCTTCTTATGATGCCGACACTAGAACACGTCTAGTCGTTATCTTCAAATGCAATTTGATCTCTCATGCATTTTTCGTCTTCTTCTGAGATTTTTCTTTTAGTTTCATCTCCAGCCTTGAGATGGCATATTCACTATGATGAATACATAACAGCTTCCAGCTACTTTGAAGTTTCTGACCTGCATTCGCTCATGCTAACGTGGAATTTATTCCCTCAATATCTCTTCAGAGATCCCACTGAACACTTCAAATTATCCTCATCTCAAGCTCATACTCTAGTATCTGCTCTGAAACTTTAGAGAACTTGCAACTTGTATCTCCATACTTCTATCAAAATCCACAAGTTCCTTCTCGAACTCAACAAGACATACCTTTTGGTCATCGAATGAGACCAGTCCATGCTTGAATATCTTACACTAACACGATCTTGATTACCTTTTGTCTTCTCAAGACACTGAACACCCACGAGCCTCAACTTTCACAGTAAACAACTTCCATCCAACTCCTAGTCTTGAACAAACCAGATCACATAACCAGACCAACTGATTTATCTGAAGCAACCCAGACATTTTCTAAACAAGAATGTGACACTCCACCAGGAACCAAGAACCCATATTCTGAGGATGCATCACTCATTACATAATATTGATAGCTCCACCTGAACAAACAATCATTTAGAAGTTCCATGTCTCTGCAAACTTAATTGAGCTGAACATCCATTCACTGTCACTTTGACCACTATAAACCCTGGTGTAACAGGCTGAATGAATAGCTTCCATGTTGTACTGGATATCAGTCCCAAACACACCATTCTTCATCTTGCAGCCACTTCAGTTTATCTTGTACCAGAAGATAAAGAACACTCCTTTGCTTATTTTTAAATCAACCTTAGCTTCACTCACTTAGATTTAATTCTCCAAACTCTGCACCCACATCAAGTTGTTTGATGTACTTGAAGAAACATCACGAGCCACCTTATCCTTCATTAAGCATTTAGCTTGGAGCCTTTCACACATTCTGATTGTAGACAACCTTAAGA

>TCONS_00018831

CCAAGATTGTATCTCTGATATGGCGATCAATGAGCTTATACAACATTTGAGGTGTAGACGAGATTCCGTCATGCAGACGTTTGTTCCTTTCCCCCCAGATACTATAGATAGTTGCGTGAGCAACAATTCTTTTAAGTATCAGTGGAGTAGTGAAATCCGTGAGAGATAACCACTCAATAAAAGATGTCCAAGTGATGAAGCATGAGTGAGTATACCCCAATCTTCTCAGAATCAAAACCCAAACCTGTTTACTGATATCACAACGCAGGAGCAGATGGTCACGGGTCTCCGGCGCTGAGTCACAGAGGCAACAGTTTGATGAAACCCCAAGGCCCCAGCTTGCGAGCCTCACTCTAGTTGGCATCCTATCGTTCTGTGCGATCCACATGAGGAAAGCATGTCGAGGAGTTGCCATTTTGAACCATACACTGTTTGACCAAGTGACTGCAGGAGCTCTGTTTCTAATCGCCTCCCATGTTCGAGAGGCAGAGAAGTCATCTAAGTTCTCCCCATCAATGAGCCAGTAAAATACATCCTCAGTTTGTGAGGATGACGGCAGATGTATACTCGTGAGATGAATATGGAGGGTCTCTGCAGCAGGCGAGCGCGCACCTCTCAGTCTCCAACCATTTTCATCACAGCTATCAGCAACTGAAGCAAATAAAGGAATTGAGAGCTCACGGGGACCTGTGTCGCCGAATATATTGATTAAGCGACCTAAAGGGGTCCAAATATCCCACCAAAAGCTTGTGGTTTCACCATTTCCAACCTCTGCTCTCAAGAAGTTTGTAGCTAAAGATCGCAGGTTTAAGAGAGATCTCCATGTCCCTGAACCCGCATTTTTAGCTTCGAGACTCCATAAACTCTCATCTCCAATTCTGTACTTCTTAATCCAAGAAGCCCATAAAGATGGGTTAGGGATATATAACCTCCAGACCAGCTTCAGACAGAGGGTCTTGTTCCAAATTTCTAGATTTCTAAAGCCAAGCCCCCCTTCTTTTTTGGGTAAACAAACTGTACTCCACGCAATCTTTGCAACTCCACGATCAGTAACTTTTCCAGTCCATAGGAAGCGGGAGCACAAGCTCTGAATTTGCTGCAAGCATCCTTTAGGAAGAACAAAGGCAGAGGTCCAGAAATTTATATTTCCATAGATAACCGATTTAATCAACTCAGCTCGACCAGCATAAGAAAGCTTTTTAGAAGACCAAGCAGTGAAGTGGCCCGAGATTTTCACCAAGAGTGGCCTGTATTCAGAAATGCGCAGCTTGCGGTGCATCAGAGGAAGACCCAAGTAGCGGACTGGCATTGAACCCAAAGTGAAACCTAGACTTGTCAAATCAGTAGTTTCAGCTTGGTTCAAACCACCTACAAAGAGTTCTGTCTTTTCTTTGTTCATCCTCAAACCAGACCAGGTAGCAAATAACTCCATAGTATCAACAATATTTGCAAGGGAGTTCTTCTCACCATCAAAGAATATCATAAGATCATCAGCAAAAGCAAGATGGGTTACCTCAAGGTCCGCTGTGTTCGGGTGATAACCAATATCTCCCGCTCTGAACTTCACATTTAGCATTTGAGAGAACACTTCCAGCGCAATGACAAAAAGATAGGGAGAGAGTGGGTCGCCCTGGCGTAAACCCTTTGTTCCCTTAAAATAGCCACACAATTCGCCGTTGACAGATACAGAGAATCTGGTAGTCGTGATGCACTGGGAGACTAACTTCCTGAAGTGGCTTGGAAACTCGAGAGCTTCAAGAGTGTTCAAAACGAAGTCCCAATTGATTGAATCGAAAGCTTTCTGTAGGTCCACTTTCAGCATACAACGCTTTGAGATGTTTTTCCATTTGTATCCAGCCACAAGCTCTGTTGCTAGAAGAACATTTTCAACAAGAAGACGTCCCGGGACAAACGCTGATTGGGCAGAGATTTATTTCC

>TCONS_00005405

GGCGTGCGGTACGTCCCAAACGGGCCAGGAAAGATAGGCTAAGTCCGGAATCTGATTCTTCTCGAGGATCATGGGTTCTGGCTTGACTGTTGGCTCTAGAATGGCGAATGGGTCGGGGAAGAACGCCTGTGGTTTGGCTGATTTGGTCATCAGGTCGTGGATCCATCCTTAGGTCACGTCCGGGTTCACTCGGGTCGATCCGGTACACGGCTCTGTCCGTTGATCTGGTACGGCTGAATGGCTGAACCTCAGTTGGAGTGATCTGATCGTTCTGGTCACCATGCTGAGTCTGCTCCACGTCCTGGGTTCTATCATTCCTGAAGAACCCTAGTGAAGGGAGGATCACTTTAGCTGGGTGTTGGATATGAACCGAGAAGCAAATGGCGCTTCTGGAACTGAGATGGATTGAAAGGATCGTCAAGAGATGCGGAATGGCTCTTGATATACCCACGATCTAAGGCTAACCACCTAAGAACGAATGTAGTGTGTTGGCTAACCACCAAACGACACACAAAGATGATTGGCTGACCACCAAATCAACAAGCCGGTTCAAACCGATGAACTAGCTAAGAACTCTCTCTCTCTCACTCAGAGAAGAAACGAAATAAACAACCAAAATGAACTGATTTTATTCATCAAAGGGACTACATATTTATAGTGTTTTGTAGTCAAAGACAATAAAAGAAAATGTGGGAAAACAATGCATAGGAAATATAAATTTGACTAGATCTAGTCAAGGCACGGAAAATAGAGAAGACTAGATCTGGTCAAGGCACGGAAAACGGAGAAGACCAGTCAAGATGTCTAGATTCATCCGAACCAGATGGATGCACGGGGTAAAGATAAGGACGCTTGCGGGACTGTCCGGATGGTCCGCCGGATGAGAATGCATGTCCGTCTTTGGATTTTTCACCACCCAAGCTAAGTCTGGCTCGACCCAAGTCAAGTATGGCACGGTGAAAAACATGAACCTCGGTTGAAATGTTCAGAACGTCCTGAACTCCATGCTGAGCTGGTTCCATGTAATGATTCGTGGTCTGCG

>TCONS_00064588

ACCTGTTCTTCTCTCTTTCTCTTTGGATACCCTTTTTTTCTTCTAAATTTTGATCTGTAGTCGCTGCGGCAGCGTTCGTTTAAGAGGATTCATCGTCACATCCTCATCATCTCTTCTTCTTCTTCAATGTAGATTTGGGGTTTGAATCGGTTCAGTCTCTGAATAACAGAAAGTAACCTTTGGTTTCCATGAAGCAGTGAATCTTCTTATCAGTACATCTCCCCATATTAACTCAGGCGATCATTAATGCTTCGGAATCGTTACACTTTAGCTATTTCTTCATCATGTTAAACACTGAAACCCTTCATCTCAGATACAGCATCTCAGATACAACTCCCAACTCCGAACAAGAAGCTAATGCTATTCGCTCTCCAACTCGCGATCCCCACCGGAATCAGAACCCTAGGATTTATCTGAGCCACGGTGGTGCTTCTCCGCGGGTTTGCAATCACTCTCAACGGTTCCGATTTCTAGGACATCACCGTCAAAGGCGCCAGAATCTTCAACATAAGCCATGAGTTCGAGTGGCAGCACCAAGCCACGATGGACCATTGCCTGCGTCGGGATCAGCAGCTTCCGCGCTCTCGGATCAAACTCAGCCTCGCTTCTCAGATGTCCACGAATTCGATCGACGGTGACAAGAGACTGCAAAGCGGCC

>TCONS_00026435

GTGGGATTTAATAGCAGGAAGAGTGCCAGGAAGACAACCAGAAGAAATAGAGAGATATTGGATAATGAGAAATAGTGATAGCTTTGCTGAGAAACGACGCCAACTTCATCACTCCTCTCACAAAAATACCAAACCTTACCGTCCACGTTTTTCAGTTTATCCTTCTTAGTTTTTAGTCATTATATTTATTCTGTTTTCTGCCCACAGCCACAAAAAAAAAATTTGATAGATGAGTTGTAGTATTATTAAGGGTAACGTTTAAAAATATTAGCGTAACGAAATTATTATGTTTGTATGTCTTTTTGTTGTGCTTTATGTTTTAATAATATAGGTTCCAATTCGTATTTAGAAAAGGTCCCATTTAGCAATCAACTATTGATCAGTGTGCTTAATAAGGTGGCAGTGGTTTTGAATTTTTGATGGAACTTTGATGATTTTTGGTGAATTTTGGTCTGTTGTTATGAATATTTTTAATAGAAATCTATCAAAGTACCACTAAAAACAAGTGAGCAGTATCGAATGGGAGTTTATCAACATGACAGAACAAGAAGAAGATCTCATCTGTCGAATGTATAGACTTGTAGGTGACAGAACAAGACCACACAACATTTCTTACTATTAGTTTGCAAAGGCTGTTCGTTGGGGATTTCCTCGTCTTCATCTCTCTCTCCTCTCTTCTTTTTCTCTATATAATTCTCATCTCTCACATTTTTCTTTTATTGCATTCTCCAACTAAACCTCGAATTCCATTTACCTTACCTCTATTTTCTTCTTCTTCTTACTATCTTCAATAGCAATGGATAACACCGACCGTCGTCGCCGTCGTAAGCAACAGAAAGTCACTCTCCATGACTCTGAAG

>TCONS_00042088

TTGGGGTCAAAATCGGTCACGACGGAATCAATGTCTGAAAGTCCGTAAAAATCAGCATAAACGTTTTTACGAAAAGTAATCTTCGTAAAGATATCTTTACGAAGAGCCTTGCAGTAAAATATCGTCCAAATCTCAATCGAACCACTAAATACCGATTGTCCGAAGGCAACGGACATGTATCCAAATCGGCCGCGGACAAGCTCGAGTATGGAAATCAGACCGCG

>TCONS_00058485

GTTATCACCTCACGAGCATATCGTCGCCGCCAAATTGGAGAATATGGGATGTTCCAGATTCCTCGAGTTCAACATTCTCCGAAAGCGAATGTTAAGATCTGCTTACGCTGGGGAGGTTGCAGCGACGGTGGTCTTTCCGCCAAATTTCTATCTATGCCGATGAAATGGAAACCTCCTCGACGATGTGTGGTTTCAAGCTCTCCGACGCATATGGCTATGAGCTCTTCTGTTTCTAGCGATTAAGATTTGTTTATGAGTGGAATCAGTCTGATTGATTTTCTTACTGGATTAATCATCATCATTTTCTTCTCAATATCATGTATATGGAGTTTCTCATTCTGTTGAACCATAGAAGCTTGAAGATTATCAACCACCATGCCATCTTCAAATGGAGTTACAAAATAGAGACATATTCTCAGGTATTGATTGCTTAAACTTTTTCCTTCTTCATTTGAAGATGGCATGGTGGTTGAGGAAAATATTTAGGAAAATAGTTATCATTTAAGGGTAAATTTGAAAAATAATATCAATTTGGTGGTAAAATTGAAGTTCTCTAAAAAACTATGACACTTTATAGGGGTATAAATGTTTTATAATAATCACATAGCTTTCTGAAAAATAGAACATACCATCATAGGAGGAGTTAGGATATACGGTTTCAAAACATCCTTCGAATTTAACCAAAGTTCGAACAATACAAACTCTGTGCGACTTGAGCATTTGCACAATGACAAAAACGCATTTTCACAAAGAGCATGTTTAGCCATGGCTTTTCATAATACTCTGTAGACATAATCCGATTCCGAATTGTTTTATCAATGAGATGAGCTAAGTGGTTCACAGGGAGAGAGGTCCGGTTGTGTCTTCGTTCATTCATTTCTCTCCAAATGTAATAGATAAAAACTTGAAAAGTTAGCCTTATCAGAATAGATGTGAGTCATGTGATCCTGCATGAAGCTGATTCACATTTTGAGCATGCCTGAAGAACCTTTATCTTTTTTGTCCATGTCCTCTCGAAATTTAGGTTCTTCGTAATCTTCGATCTCTGACTTCAGCAATAGAGTTTTGAAAGATTGATATGCTCAAATCAACGTGAAAAATCTCTATGTAGTGTCTCAATCGCATTGTTTGATCTTCTTTACATATCAATAGAAATGTTTCGAGATATAGATTCACCGCCTTTATAGCTTTCTTAACTCCATCAACCACTGTCTAGTCTTCAATGCAAGTTTATCTTCTGTAAGTCTACGACTATAAAAAG

>TCONS_00057043

TGTTTGCTATCTGAGTGTTTTTTTACGCTCAAGCCAACCATCTTAACCAGAAGTTATTGGCCTTGGATGTGGAGCCTGAGGTGATGCCGCTACCAGTAATAACCTCAAGTTCATGGGAGGTAACCTTTCATTGATCTCCTACAACTTGCAGGCTGGATTTTCCTAAATGCAATTTCTGGCACACATATATACTTACTATGACAAAGAAACAAGTGACAGTGACACCTTCATAGCCAGGTAGTTTGCTCTAGCTTTAACAATTTGTTCTTGATTTTTTTGCTTGAAAACCCACAATGTAGACAAATACGACTGTTTTTTGTAGCTTTGGTAATGGTGACTTAGCCTCTTCCCAGCGCCGGTCAATATGTTTCATGGGCCCTACTCAAAATTTGGACTTTAGCTGTGTAAAAAAATTGATGAAACAGTACCTTAAAGGAAAAACGAGAATTATGTATGGGCCCGTAAGCGGCAGCTTATACTGCTTCCGCAGCGGGCTATGCCTGCCTCTTCCACAAGTTCGATGAAATATGGTGGTGCTGATAAAATAGAAACAGTAACAGTGTCAGAGCTCAACACCTATGTTCTCAACTCTACACCTCAGATCCATTGCCTATATCTTTCCTAAGCTACCATGATGCAAGCCACCATCAATGCTAGCGGGCTTCCGAGATTTCGGTCTAGGCTCGCCGCCGGGACGATGTTCTCCGTCTCCGGATTTGATGTGTCTGGTGTGCCCAGAATTTCCTCCTTTCTTCACTATCACCACGAGTAGAAAGATGACCATCATGAAGGCTTGAGTCATCTGTAATTTTTTCCTCAAGATCTAAAAAACAAGATTGAAGAATCTAAGTTATAAAGAAGCTTTGCAATTTGATGAAAGTCGTGCGTCCTGAAGCCGCCACGGGTCCATCCACACAAAACGCGCCGTTTCATTTATTCTTCAGCATAGTGGGCTTTCTTTGGGTGTTCTGTATCTAGGCTTTGGACTGTCGAGTTTAGTTCCGTATCCGCCCCGAGAGAACAGAAGACGCCACTAACCCTAATCAGTAGGCATGGGCGTTCGGGTACCCGTTCGGGTTTCTTGGATTTCGGTTCCATTTTGTAACACCTCCTAGGTCCCATTCTAGTAAATTTACAGGTACAGATCGGATTCGGATATAACACTTCGGTTTTGGATCGGTTCGGATATATCCGAAGTAACCACATATCATTCGGATTCGGCTTATATCGGATCGGTTCGGATATATACAAAGTAAAATCTAAAATTTAAAAGTATAACATAAGAAATATATTTTTCTTTGCATATAATAGAGTATTTAAGGTATTTATTTAATTTTTCAATACTTATTGTTATATATCATATCCAAATAAATATGAAATTAAATATTTGAAGTACATATTTATATCAAATATTTATATTATGTATTAATTTGAACATTCGGATTGGTTTCCTCGGATATTTTTTCGGGTTTTCGTGCTTTTCAGATTTTCGGGTTGCCCGTTCGGGTTCGGTTAATAACACTCCGGGTTCGGATATGTTTAGTAACACCCTATAAGATCCATTGAAGTATTTTTTACATTTCGGATCGGGTACGGATAGGTTTTTTCGGTTCGGATTCGGATTGGATTTCGGGTTTCGGATTTTATGCCCAGCCCTACTAATCAGCTTCAGACAAGAAACGAAGGTTGTATCTCTGCATCCTTCGACGGCGACTCGATGTCGTGTCCTCTTTCTATCACCATCTGAAACAGTCCGAGTTAAGAGTTCGTGTTTAATCCCATCGATTCGCTCTCCCACGATGTGTATTCAAAGCATTCTTCGAGCTGAGAGGCGGTGTGGTTTTTGTATAAATATATGTGAGATTTCTCCCTTTCAACTCATCTTCTCTTTGAATCTCTTATATTCATCCTGATAGATTTTGATATGGCTAACTCCAGGGTTTTTTCTCTGATCTGAAGTCCGGCAAGTGTACCTCCGTCATCGAGGCTAGGCTCCTGCGGTTCTGGGAGGCCAGGAACATTAAATGCGGTGGGGAACTCATGTGGGTTGATATGCTCCTGGTGGATGTTAAC

>TCONS_00036228

TTTTCATTAGCATAATTAAACAGAGACTTATACACGTACATCAACATACACCAGTTGATTAAGTAGAGTGAAACACGAAAGATGCTAAACAATAGAACAGAAGATACATATGATAAAATATCAGAAGAAGAACTCATCCGAATTTCTATCTAGTAGCTAGTATGCGTCGAGCTCACACTCTAGCTATAAGAATTGTTATTGTCACAAGCGAGCTAATATTAAGATTAATTATATGAAGGATCTCGATCGAGTTGGTTCAATTGGCCTGGTAAAGGAGGGACTGATAGGGCTTGATCAAGGGGACGAGAGAACCACTAAGGTGAAGTGACATGACTTCATTGGTGGAACCAACGAGCTTGCCACC

>TCONS_00005812

GTTGTCTCCTTCCTTATTTATTCCCGATTGAGCAAACTCTAGTCTCTACTTCAACTTTTCCGTGTCTAGAAATGGTTTCCCCAAACAAAAACGATAAGATTCACATGCTTCCGATTTCAGACGCATCGTCATCTTCATCTCAGACTAGGGTTTTCACTTCCAGGACTCGTAGCGTTCCTCTCTCAAACCCTACTGAAGAAACTGGCAACTCGAAAGCTGCGACTTTAGGCTACGCAGGTTCTCTTCCGAGCCAACGTCCTCCTTTATTCCCAATGACTGGCCCTCTTTCCTCTTCTACTCGAAGATCTTCTGGCTATTTTGGTGACCTGGAAGAGGTAAACTCCTCAGACAACGATGAGTTATTGAAACACGCACATCGCCTGAGATCTGGAAAGCTGGGGATGTGTAACGATCCTTACTGTACCACTTGCCCTTCTAACTATAATCCCAAAGCATCCCGGCTTCCCAATCCAACTGTTTCTGCTTCCACGGTATGTTTGATTTTACCCTAACAGCACTAAATACGCTTGAGACTCGAGAAAATTTTGCTGACATGGAGTTTTTTTTTTCTTTTTCATATTAGTTCCATAATGCCTTGTATGATGATGCTAGAAGTTGGGCTAGGCGATTTGCTTCTTCTGTTAATAGATGCTTACCTGGAATCATGAATCCTCATTCCAAATTCGTTCAAATATGGACTAAGTTCTTTGCCGTCTCAAGCTTGTTAGCCATTTTTATAGACCCCCTCTTCTTCTTCATCATATTAGTCCAAAAGGTATATATATGTTTCTTCTTGGATCTCTTTCTTCCGTTTGAATATCTTTTTGACCATCTTTCTGACTGCAGAACAACAAATGCATAGTGATTGATTGGCCGATTGCTACCGCATTTGTTATTGTTAGAACTCTGACGGATGTTATATTCTTTGCTAACATGCTGCTTCAGGTGTGTTTCCTTTTACTCCATTTATTGTAAGCAAAGTCGGTACTGGACATAGCGAGATAAAACATTCGCATCAGCCCTAAATTTAAGTGGCTGATATGTTTAGATTATTCGTCTTTCAAACTGATTAAGAAAAATATATGTATTAATCATTTACAGGAGCCTGTCCCGGTACACATCATTTTACAACCACTAGAAGATAATAATTTTGTACTGTTGTTATGTCTGTAAAATCTTAATGTAATTTGGCGCATAGTGATTGATTGGCCGATAGCTACCGTAATTGTTTCAAACCTCATAATCTTAGGCCTGATTCTTCTTACAAATAAATATTGGTTTACATTTAATACAATTTTTACTTAATATTTTGTTGCAGTTCCGACTGGCATATGTAGCTCGTGAGTCTACGGTAGTTGGTGCTGGCCAGTTGGTTGATCGTCCAAAAAAATTGCTCTCCATTACTTTCGAGGATATTTTATCGTAGACATGATCATAGTGATGCCATTTCCACAGGTTTTGTAATGTTAAATACTCAATACTACCAGTGGCGGATCTAATCCGGGGAAAAACAAAAAAAAAATACTACTATATATTTACAAAATTACACTAACTAGAAAATTTCATGGGCAACTGCCCCACCCACTCACATAGATCCGCCCCCGAATACTACTATTCGTGTTTTGAGTATGGTATTTGGAAGATGTTCATCTACCACAAATCTTACCTTTCCATTTTTTTTTTGGCCTTACAGGTATTGGTACTATCGGTAATACCAGCACAGTTAGCCATATCCGGGACAAACTATGCGAAAAACCTTTTACGTACTGGAATTCTTGTCCAATACATTCCAAAGCTATATAGACTTCTACCACTGCTTGCTGGACAAACACCAACAGGCCTCATATTTGAGTCAGCTTGGGCTAACTTTGTTATTAATCTTCTCACCTTCATGCTTGCTGGACATGTTGTCGGCTCTTGCTGGTATCTTTTTGGTCTGCAGGTACATTCAATTTAATGCATGTCTTGTCCAAATGCAATAGAAGACTTTTGTATATATTTCAAGTGATTTATTGATTCTTCTCATTGCCTTCTACCTCTTCTCTTGTTGTGTTTGGTCGTTTTAGAGAGTTAATCAATGCCTTCGAAATGCTTGTGGTCATTCTGGGCGTGAATGTAGAGGTCTTATTGATTGTGGCCATGGAAATAGTAATATATCAGCATCCCTACGAGCTATCTGGAGAAATAGTGCGAGTGCAAATGCTTGTTTCCAAGAAGATGGGTTTTCTTACGGAATATATTTGAAAATGGTCAATCTTACAACTCATACTAGTCTCTTGACAAGATATAGTTACTCTTTGCTATGGGGATTTCAGGTAATGGTTTACTCAAACAATGTTTGGTAACTGTCCTAGCTACAGAATTGCTATGTTGCTATCCATTGCGTACTTAATTTTGGCTATGAGTTGTGTCCCATAGCTACAAACTATGTTAGTTAATTCTTGTAGTGGTGGAACTCTTAAACTGACGTCATGTTCTTATAACTTTCAAAGCAAATTAGCACGCTTGCTGGAAACCAAGTCCCAAGCTACTTTTTCCCTGAGGTCGTTTTTACTATGGGTATCATCGGACTGGGACTTTTGCTTTTCGCGCTTCTTATTGGTAATATGCAAAACTTCCTTCAGTCTCTCGGTAAAAGGTTAGAAAATAAACCCATCATTACATACATCATCAATGTATATAATAAGATATTTTGAGACATAATGTATGGTTTATAAATCAACTTGTGCTCTGGTGATTGTGCTTTGTGTATCGTAGGAGCTTGGAAATGACGCTAAGACGGCGTGATGTAGAGCAGTGGATGAGCCATAGACGGTTGCCAGAAGGGATAAGAAAGTATGTGTACTATATCTATGCCTAATCATTTAAACTTTGTCTAGTCTTAGTAAAAAACTTTAACTCACACTCAAAAACTAACTTCACTTATAAATTGCACGAATTTTATTTTTTCTCAGCCATGTGAGACTTTAATAAATCATCTTGAAATGTGGGTGGGAAATAATTTAAGGGAAACGGGTCAAATCCAACATCTCGGTCGGATATACCGCAACAAGATGTGCTAGTTATACATACTACATAAATAAAAGAAAAATTGTTTATGTCCAAAAGTAATTTTTGGCTTTATCATTAGCTTTGATACAATATTAGTAATATACCTAACTTACATCCAAAAACTAATTCAAGGTCTATGATTGTTACATCACGTATACATATATTGCCCAAATACCTGTTGTCTATGGGATGTGTGACTTTAGAGCATCTCCAATATGACACTCTATTTTGAAGTTTCCACAACTCTATATTTGAAGTTTAAAGGTGTTATTCTCCAAAAAGAAAATTTCAAATTTAACATCAAAACTATTTGTATTTTATATTATGATCTTTTTATTTGTCATAATTAATTTATATTCATAAGATTTTTGTAAATAACTGGCACACGTATAAAAATATTACAACAATATTAATTAATAAAATGTTACATTAAAATATCAGATTTTAAATAGAAATACATAGTTGATATTAAACTCAAGAAAAATACCACATTATTCCAAAAAATTATATCTGTAATGGATATATGATCAATGGGTGCATTTCGAAGTTAAAAATGACTTTCTTTATTTTTAATTTGTAACAAGCTAAAAATTATTGAAATCAGGTGATATTAATACTTGTAAATACATTAGATTTTAACAAGAAAGTAAAAGAGGAAAATAAAATATTGCTAAAAGATTTAACTTCTATCGATGATATTAATACTTGTGAATACATTCGATCTGAACAAGAAAGAATCGTACAAAAAAATATCAAAATAACAACAATGATCTTCGGTTACACAAAATTTGTTTGGAAAATATTTTGGAGGTTTCAGATTAATTTTTGCTGAATATTAGTGTTATTGTAATATTTAAATTTGTATAATAACTATGCATATTTTTATTTTTTAAAGCTTTTTTTTGTTAAGCTTCGTTTGTATATTGTTGTTGTCTAAATCTACTATTAAAATATTTAAATCTTATTTTAATTTTTTATTTAATTTTATGTGTAAAACTTAAATTTATACAAAATTGAAATATTTATGATATTTAAAAATTTTAAGGATTAAAAAAATAAACGAGAAATTATTTAAGAATCATAAATGTGATGTGTAATTGAGGGGACCAAGATATGAAACTTCAAATTTAAAGTTTTGATTAGTGAAACTTCAAATTTAAAGTTTCACTCTTCAAAACTTCTTATTTCTTTTGGAGATGCTTTTAATACTTTGCTCCTTTGTTTCCTTGAGAGTTCCATTTTCTCAGGCTATGTATTTTCATAACCAAAATGTTAAAACGTTTAGGAGGGTGCGAGAGGCAGAGCGTTTCAATTGGGCTGCTACCAGAGGAGTTAACGAAGAGTTGCTCTTTGAGAATATGCCTGATGACCTTCAAGGAGATATAAGAAGACACCTCTTCATATTTCTCAAGAAAGTAAACATCAGAATCCATTTCTCTCTGTTGAAGTTATATGTACTTAACTTAGACCCCAATTCCTTAGTGCTTTTGCATAGTGACTATCAGTGAAACTGAAGTCTGAAGTAATATACGGGGATCACAGTATAAGCATTCCTGAAACTATGGTGAAGGATATGGACTAAAGAAGTTGATGGGTTTACTTTACAGGTAAGAATATTTTCGTTGATGGATGAATCAATTTTAGACTCAATACGTGAGAGGCTGAGACAGAGGACATACATAAGTGGTAGCACAGTCTTGCACCGCTGGGGTCCGGTGGAGAAAATAGTTTTCGTAGTGAGAGGTGAGATGGAGAGCACTGGAGAAGACGGTTCTGTTCTTCCTCTATCAGAAGGAGACGTTTGTGGTGAAGAACTTCTCACTTGGTGCCTTGAACGCTCTGCTGTAAACCTCGATGGGAGGATGCTTTCCAAGGGATTGCTTAGCAGCAGAAATGTTAAGTGTGTGACAAACGTGGAGGCGTTTTCGCTGAGTGCAGCAGACCTTGAGGATGTAACGAGCTTGTTCTCGAGATTCTTAAGGAGCCATCGAGTCCAAGGAGCCATAAGATACGAGTCTCCTTATTGTAGGCTACGAGCAGCTACGCAGATTCAAGTGGCTTGGAGATACAGAAAGAGACGGCTCCAGAAGTTAAGCACTGCTCAAAAGAAACAATATTCGTCAGAGTTAATCCAAAAAGACATGGCGAAAACATAG

>TCONS_00029001

GAGAGACGAACTATCCTGACAAAGTGACAGTCCAAAGCCAACGCCGCTATTTTATCGGTCCTCCGTCAATTCCGTGCCAACTGTACCAGCGGTGCAAGCGTACTCCACCCACTCCAACCCCTCCGTGCCACATCTACAACCGATGCAAGGGTTCTCCAGGCAAATACGGATCTGGTCACTGATCTTACTATAGAATCAAATGCGTTATATACATATCGACGTTTCTACGATATAGTTTGCTTTCCAGTTTCTGTGGTTCATCGTCTTGTGTTGTCATGTTTTTTTATAGATTTATCAATTACACATATCAGTAAGTTCACTCATCTACCATGTGTTCTACTCTCTCTTATTTGCTCCTCTGTGTTCTATATAAATGGGTCACATGAGTCACACTGATGAGTAAACTAAAACGAAATACTATAAGATGATGACATCTTCCATAGTCTCCTGCACGTTTTTCTTTTTCCTTCTTCTGATTGTGTTTCCGCATATGGATAGAGCCCTTGGCGAACAAACGGAGCTCCACAAACTCAGCA

>TCONS_00030257

GGGTTTGGATCGTTGTTTTTGCTACTTGCTAGTGGTGTTTATGTCTGATCCATCTTGCTCAATACTATCCCCAGCTAGTTTCAATGAACAAAAAAAACAGGACTTTTATGTCCAACTTAAATCAGAGTTTTGAATGTTAAGACTTCAACTATCTTTCTATGAAGCTACATCATCGAGGAAAAACCGTAGCCTCGGGAAGGAGCTCGCGACTGCAGCAGTAGCATCTGTCTTCTTGGCGGCGACACCGATTGCGAGTCCTATTCCGGTGGCGATGTACCCGACTCTGTCGGTGGTCACTCTAGCGATTGGCCTCGTGATCACAGCTTTCTTCTTCATGCTGCTGCTCATTGTTCCTTCCTCAGATCGAAAAGCGAAGAAGATAGCGACGACAATG

>TCONS_00013899

CCTGAAGGTAAGGTTAAGGGCATCAGGGAGCTAAGGCATAGCTAATCCAAGAAGGAGACAAACTCAAAGGAGCTGGACAAGCGAGCTGGTAGCTGGACAAGATCCAGGTGAAGCTCGATGAAGTGAGTGATCAGAATGGATCATGGGAAAACTAAGTCTAGGTCTGGAAATTGACCAAGGGACTTAGAAGGATTGAAGAAGATAAAGGAAGCAAGCTGGACACAGTGTATAACAGCTGGGCGATGCAGTAAGCAAGCTCGACCAGCTAGGTGAAGTGTAGTGCAGCTCGGTGTAGCTCACTGAAGTGTAGGTCAGCTCCCTGAGCTGGATAGTCTAGCTCACTCAGCTGTATCAGCTGGGGATCAGCTCAACTCAGCTGGACTGAGTGTTCAAGTCATGGGCAGTTGGGCCGGGTCTGGACAGTGGCCGGGCCATGTGGGTGACCCGTGTGTGCCGATGGGCTGGTGGGCTCTTGGGATTGAGCCAGGGGCATGGGCAGTCCGTGTGGGATAGTTTTGGACATGTCCAGGAGT

>TCONS_00063858

TGGCCAAGTACTCGGTGGCGCAGATTGGTACCTTCTAACTCCTACTCTTATCTGGAAAGTCTCAAAACCGAGCCTGGTGAGACAGATTCATCTCAGCGTTTGGTTTCTGAGCGCGGGAGCTAGCCAGAACAATCGGTCTCGCAAGATAAAAGAACTCGACAAGAAGGTGAGACACAAGTTGTGAGATACAAATTATGTTTTGTTGACATGTGATCAAGCCAGTGATTGATGAATCCTTGACACATATCACTAACATTATCTATAGGCTAAAAGCTCAACGAAGAAGAAGAATAAAAAGCTTGGAATAGAATGAGAAGCTTTTCATATATTCATGTTAGAGCTCGAGTAACTTAGCATCTCACATATTTGCACAACTCTTTTTTTTAATCTTAATCAGTTGATTAATTGCTAATTCACTACTAATTTTCAGATTATAAGAGAGAAGGGAGAAGATTAGTGCATGGATGAAGCTGCTACAGGAACTGGTCCCAGACTGTGATAAAGTTTGTCTCTCAGATAATGACTTCTGATCAATATGCTAATTAATCTTTATGTCTTCGTTTGTGCTTAACGACATAATATTTTCTGAAAAGACACCCTAAAAGTAAGAATATTGGTTTGATGATATAGTTTCTTTTTGTTAATTAATCAATTTTTTTTCTAGATTTCTTAATTGCTTTTTATGAAGAAGAGTGGTTTCTCGTGTGCCAAAGAGGCCTTAAAGATCTTAACTATATTGTTTCTGTGTTAGTGTGTTGGGAGAAGTATCCTCACAGGAAAACTTCAGGATTGACCGTTTCTTTCTTCTCCGTGTTTACTCCCCCCTTAATTCAATCACCACGATATAGCCTTGAATGCGTGCTTTGATTAGGAGTCGGTGAATATGAGAGTATAAATAGGTGAGCATTCTTTCTCTTACGATCATCTTATCGGCGGCGGAGAAGGCTATGTCTGTCTGCGAGCTCAAAGAACATCACACCGACGCTACAGAGACGGTTAACAACCTCTGTAATAAGCTTAGACAGAGACACCTCCAGCTCCTTAATACAGATG

>TCONS_00023924

CGTTTCTGATCATCTTTGGAGTTGATCCTCCTAGCAACATAAGATAATCTGGTGGGATTTCTTTGAAGCTTTCTTCCTTGCAACCTGAAAAATTCTCAACATTCTGGACAAAAAGCAAGTGCATTATATCTGTCATGGAAATATTAAGAACATGCTGATCTAAAATGAATCTTACCATAGGTTTTGGAACTTGAACAATAGATTGTTCAGATTTCGGTTTCCACTTGGGAGTGAATTCATGGCTGAGCTTATGGTCTGGTTCTTTCTTTGGGACTTCAATAAGCTTATAGCTTTCATTTATTCTCCCTGCACCAAGAACACACACGTCAGTACTAGTATCCAAAGTTGGTGTTAGACACTTACCTTGGTGGGATACTTTTAGGATCGGTTTTGCTTCTTTAAACAACATGGACTGCATTGTGTCTTGAACCATCTTATGGTCCATCACAGGTGTGGCGCCTGGTGGCTCCTCTCCTTTGAATTCATGCTCCTTAGTTCCTGTTACATCACCCTTTGACAAAGACAAGTGCATCATACAAGAATTTTGAGCTAATAAATCAGATTGAATAAAACTATCACTTTTCATAGATAAATCTGTTTTCTCTTCTAGTGATGTTTCTATCAATGCTTGCTTAGTAGGACAAACTACAGCATAGTGTCCTTTCTTATGACATCTA

>TCONS_00033749

TTCTTTACATCAGTACTGTGAACATACCTTTAGCTGACGGAGCTTAGACTCTTCCTCATGCATTCATCATCATCACCAGCCATGGCTATCTTATAGATGTTACCTGCTTTGACCTCTTTCTAAAACAACTCTTGCACTTGACGATTGACATGTATCAAAGATGTTTGTTTTTCTTGAATGAATTTTTGTTATAAACATGGTAATAGCAGACGTCACTTAACGCAAACTAATTTAATTACTTGCGACTTGTACAAAATAAGCAAGATAAGCATTCTTGCTTGTCTTGACGGGTATATGAACATAGCAATGGAGCAGACAGAGGAGTATGTTAACGGGCAGCTCAAGAACAAATACGGTGATGCCTTCATCCGTGGAAACAATGTTACGTTAAAGGATTGTGCTTTGAAGAAGAACAGATAAGATGAGTGGAAGTGGAGATAATAAAGTTTCTGGAACCACGAAGACACCTGCTGATTTCCTCAAATCCATCCGTGGGAGACCTGTTGTTGTGAAGCTCAACTCTGGTGTTGATTATCGTGCGATTAGCTTCAATTGAGTCGAAAGTTAGGAGCTTTAGAGTACTAAGAACTGAGAGGCTGTTGAGGAATAAC

>TCONS_00021953

GGGTAATACCGTAAATAAACTGAAAGCGAGAGATGCGGGCACGTGAGACGAATTTCTGACTCTCCAATCGACCTTATCCACCTTGCGAGACATCAGATCCAAGCGCCACGTCAGACTAAGATCAACGGACGAGATCTTCCTGGTCGCCTTTAAAGTCATTACTCTCTGCAACGTTTGTGAGAAGAAAGAAACATTACTTGCGACCAAAAATTACAGATTCTTGAAACTATTAGTGTTGATACTTGATAGATACCATGGGCTTACTAGATCATCTCTGGGATGATACCGTCGCTGGTCCTAGGCCAGAGAACGGCCTTGGCAAGCTTCGGAGACACCATACCTTCAGTTTCCGGCCTAGCTCCGATCAATCGGACGGTGGAAGCGTGAGATCGTACGGTGAAGAATCACCGGAGGAGGCCGTGAAAGTGACACGTAGCATCATGATAATAAAACCACCGGGATACCAAGGCGGTTCAGCTCCGGTTTCACCAGCCGGTTCAACTCCGCCAGTATCTCCTTTCTCTGGTGAGTTTTATTATATTAGTTACCATTTTGTTTTGTTCAAAAAAAAAAAGTTACCATTTTGTTTCCAGCAGTAACTATTTGGTTAGGTGAAATAGAAAGTGTACTGTACACTCACTGATTACGTTAAGGGTAATTTCGTCTATACGTTAAAAAAAACTTTGACAGGATTAACATGTTGACACTGTGACTTATGTGATGCATGCAGCATGATCCTTGATAAGATGAGATGTGACACAGTGAACTCCATCTATGATATCACTGATTATTATTGCCCCTTGATTGATATATTTGTTAGGTGAGCGTCTAGTCAAATGTTGTGATATGCTTAAAAGACTTGGTCATAGATTAGGACCGTATTCAAAACATGTTATGGTTTACTAACGAACTTGGAACCTTTGGGATGGTTAACGGAGCAAACGCAGGAGGAAAGGAACCCTTTCGGTTTAGGAGAAGGTCGACGTCGGACGCGTTCGATAAGGGAGCAGGATCAGAGAATGGACCAAGGAACTCTCCTCCTACTTACGGCCTGTGATCTATAACTCAAATCTCAATAGGGAGAGAGAAAAGAAGAAGAGTAGCCCCCAAAACGCTTTCGATCAAGTGGTCCTTGCATGCAGAGTTCGTGTTGCTTCCGTTTGTAGGCCAAAGAGGTTCTCTCTTTTGTGTGCGTGTATGAGTTTGTTTTGTGTGTTTGTGGTTACGTCTATGTTTTTGTTTTTGTATGTCATCTCGTTTTGGGAATGCTTCTACGTGTCTGTACATATTTTGCTGCAATGATAATAAAAATGTGTAGATGCTTAACATCACTTCTTTTTGGACACCACAGTCTCTTTGGAGGCCATATTGAGAGTGACTGCAAAGACACTTTACTCTTTTCTTGAATTGATAAATTATCTCCAATCCAAATTGTCAAAGCCTAGCTTGAAAAAAAGAGGAAATTGACAAATGGCCAATTGTGTTTAGTTGAGGAAGCTTGCAGGGATTATGATGTGGA

>TCONS_00019796

AAGAGACTGTTGTATATGAAGAGGTTTGGACCGCTCTTCTTCCTCTTGAGCCAGTCTACTACAGCCATTGCTGGCGTTAGTCTCTTGTCCTTGCCGAAGCCTCTTATCATAGCGCAGTAGACTTGAAGAGGCAACTCTCCTTTCTCCTTGAGAACAACGTCTACATCATCAGCAGTCTTGGCAGCTCGTAGACTATACGCTAGCTCTCTTACGTTAACTCTTGAATTGGTTTTGTCCCTGGAGGCTTCTTCCTCTTCTCGTTGCTCCGTGGCCCATCCTACTCCAACGGATGATCTCAACAAACTTCTCTTTGGCTCACACAGAAACAGAACCTTTGAGGTATGGCTGATACTACTACCAAAGTTACAATGTCTCTTTCTAATCTTACGGCTAACTACGAAACAAGAACAATCCAACTCAGATTCCAATCTTGATCCAACCAAAAGACCAAACTTTAATGGCCAAACGCTTAAAGCTTGCATCTTTCACCAGCTAAATCAACCAATAATAAC

>TCONS_00074054

GTAGAGTCCCTCATTGACTCTACCTCGCGTACTTGGAATTCAGACGCAATTCGGGCTTTGGTGGACCCTCAGGATGCAAAACTTATAGAAAGTATTCCCCTAAGCAGGACTCAGCGGGTTGATAGAGATGAATGGCACTTTACAAAAAAATAGAAAATATACGGTCAAATCAGGATATCAGGTAGAGCGGATTTATCCAGATAGAGAAAGAGTACCAATACTGATTGGTCCCACAGTGGATGTATTGAAGGCTTACTGCTGGAAAATACGGTGCTCGCCAAAGCTAAAACATTTTCTATGGCAACTAGTGACAGGGTGTATAGCAGTGCGGAAGAATTTACAAGCAAGGGGGATTCAAGGGGATATTTGTTGTGCAAGATGTGGAGCTCCTGAGGAATCAATAAATCATGTGTTTTTTGAATGTCCTCCAGCTCTCCAAGTTTGGGCTCTTTCGAAGATACCATCAAATCCAACTATTTTCCCAACAAGTTCTCTCTTCACAAACATGGATCATCTATTCTGAAGAGTTTTACCACAGATGGAGGATCACCAGTTTGTATGGATACTATGGTACATCTGGAAAGCTAGGAATAATAAAGTTTTCAGCAATTTGGATATGGACCCTATGGATACGCTTAAATTGGCGGAAACAGAATCGACATTGTGGGCTGAGGCACAAGTTGTGAATGACCAGAGGATGCCACCACCAATAATAGATATGATATTGCCGTCGATCCCAGGAAGATGGTGTTTCACAGATGGCTCGTGGAAAGAGGGGGTTACTTTTTCAGGGCAAGGTTGGCTTAGTACTTTGGAAGGTTTTAATGGATTGTTGGGGGCGAGGAATGTTCGGGCTAGTCTTTCGCCTCTTCATGCGGAGATGGAAGCGCTACTATGGGCAGTGGAATGTATGAGGAATTTACGTCAATTTCAGGTCACGTTTGCAACAGATTGTTCTCAATTGGTGAAGATGGTTTCGGAACCAGAAGAATGACCAGCTTTTGCAAATTATTTGGATGATATCAAGATCCTGAAAGAGAGTTTCACCCGATCAGAGCTTATCTATGTACCACGAACGCACAATTCAAAGGCGGATAGTCTCGCACGCAGTGCTAGAAAGC

>TCONS_00017694

CTGAACTATAAATTAGAATCAGATTGAAAATTAGCTATTTTTCAATTTCAAATAAAATTCTACTGCCGTTTTATTCCGTCTAGTTCATTTTTCCAACTAAAATAAACCAACCAAGACACATAAAACAATAACTCAAACAGTTTTCATGAGATTGAGGCGTACCTTCTATTTTTAGTCGAAGCAGACCTTCTGTAGGACACGTACTTTGACATTGAGGTCGTGAACCTGTTCCTGGAAAATGGAAAACACTCACAAATGTCAAAATTATCATCCAATGTCAAAATAAAACACTCACAAACACTCACAAATGTCAAAAAAATAGAACTACTGTATTATCTAAATATAGGAGGATTCTCACCGCCAGTGTTTCAATCTGTTTATTGAGTTTTGCCACCAAAAGAGTCACCTCCTCGGACTCCTTCACACGTTTTGTCAGACTCTCGATCTCCTCCTGGACACCAAACACCCAAGGATGACGAAAATGCAACCCATCATCCTTGGTTACAACAACAAATACATATCAGATTAAACGGAGGCAAATAACTTATACAAACTGAAAAGGGATACACAATGCGGAATCGTTTACCTCGTAGTTCTTGCACGTGAAAAAACGCTTCCCAGGAAGGCTGTCGTACTCCTCCTTTCCACGAACCTCGTTAATGATGCGCGAACCACACAGACACCTCGTCGGAATCCCGTATTGCGCATCAGCCGCGCATTGAAGCATCATGTAGTGCTCCTTTTGCCTCTTCATCTCTCTTCTCTCTTCTGCTGGGTCCATCTGTACCACAAGTCACACCATTTAGAACCCTAATTAGAACCCTAAAACGAGCCTCTCAATTAGATTAAGTACCCTAACCAAACCGCATGACGAAATATAACCATTTATCAAAAACCCCCTTTTCGATTTAGAACCCTAGAACCCTAACTCTTAACAATGGAAATCGAATTCGCTTTGGACCACGTAATCGATGGAATGAAACCGTTAATCTAGTCGATTCTCACCTGAGCTTGTAGAGGAAAGCTCCGGCGTCGTCGTCGTGGAGAAAAGCTCCGGGTAGAACCGTCGTACGATAAATCACCAAGGAGGCGGAGTGAAATGAGAGAGAAGAGCAAATGAGAAACATTTCGAGAAACCCTTGTTTCGCTTCCCTCTACAAAAAAAAACGCCACCAATGCCCTCTTGCCACGTCGCCTGAACCCGTCTCATACGGTATCATTCGGACGAACCGGTTCACCAAATATCGGCCTTTTAAAAATTTTAAAAACGCAAAAGGCCATAAGATCCCGAGCCCATGAACCCC

>TCONS_00061758

TACATACATCTATCTTCCTTCTTTAACCCTACAAAGAAATCAGATTCCCATTTTCAAAACCCCCTTTTTCAATCCTGTACTATCAATATTCATACATATTCTCTGAATGGCTTCCAACTCCATGAGTTCTAACGCGTCTTGGACACGTAAGGAGAACAAACTATTTGAAAGGGCTTTGGCTATATATGACCAGGACACTCCTGACCGTTGGCATAACGTTGCTAGAGCAGTTGGTGGGAAATCAGCTGAAGAAGTAAGGCGACACTACGAGCTACTCATTAGGGATGTCAATGACATCGAGTCAGGGCGTTATCCACAGCCTACTTACCGTTCGAATGGAAACTGAAAGCATTTAAGCTACTCAAAAAGGTTCCTCTGAAACCAACATGGTTACAAACCAGAAACAAACAGAGAGCATCTAAACAAATTAAGAGATATCACTGTATTTACTTCTATTTGTGTGATTGTGATATATATACGACTATGTAAGAATCATCTATAAAT

>TCONS_00037477

TGACATGATAGGTGGTGGTTCTTGATGATTGTGACACTCTCAAGATGATCTGCTTGGAAGTTTTAAGGAAGCTCTTCAAGGCTCGATAATTATAATTTTAGTTGTATTTCAGTTTGTGTGATTAGCGATCAAGCATCCAAATTCAAATTTTCTTCATAAACTTGAATCAATGTAGATATATAAGCTAAATGGAGCACATACATAGACGTGTATCCATGCACCACTTACACAAAAATGGAGTACATAGACATTTGAAGTTACATGCATACACCAAATATACAAAAGCAAGAAAATTTTCAGCCACGAACACAAAAGGGAACGTCATCATCTTTTAGTTGAGCCGTCAAATACTGCCCTCAAAGCTCTGAAAGCTTCTGCTGTCAAACTACAACACTGTTGAAGTAAGCACCTATTACTGAGCCCTGGAAGAAAACTACGAAAGAAAACTCATTATATAGGTTTAATATTTAGCTTGTACATGTTTAAGTATCTTAGGTCGTACCGGTCCATGTCTTTGTTCAACGCAATCACGCAAAGCCAATAAAGGAGATTGTACTTTCTCTCGTGTGGAGAAGTTGGAAGCGGTGGTGACGTTAACGGTGATCATGCCTTTGTCTCGCGTAATAGAAACGGGGTCACTAAGAGCTACGGGTTTGCTGCGACTTTGTTTTTTTTACCTGAAGAATCTTCTCAAGCGTGGAGATATCCATGATCATCAACAATAAGGACCGTAATCAAAAGCCAATAAATATATTAAAGAGACTCGAAGTATGTAAGATCATAACAGGCATCAACTTCAGAATATGAGATGTGGAAATCAATTAAAAGACTGCATTTAATTTACCATACTATTAGGACAGAAGCCTATATTTTAGGAGACTTGAGCAGTTGAAACATACCGCCTCCGCCTACTGAGCTTTGTGTGAACCTCCCTCTAGTCGTTTATTGCTAATTTGACGTTCACCCACCTATTCTATCTAGCTGTCCTAGTAAACTTGTTTTATTTGTTTTAGAATTGGTTGCTCAAGCCACTTTTTTTCTCAAGACACTTTCTAAAGCTAATCCTG

>TCONS_00068466

GTCGAGGCATAATTAAGAATAAAAGGGTTTAATGATTTGTCCCACATCGCTAAACTATGAATCGTTCACTCTCAAAGCTTATGTATATAAAGGAAGCTCGATGGACCAAACAATTCATACCTTTCTCGGCCTTTTGGCTAAGATCAAGTGTAGTATCTGTTCTTATCAGTTTAATATCTGATATGTGGTCCATCGGATCACACGATATTAACTCTATTTTTTAAGGGAGAAAGTCTATTTAAGATAGCTTGCTATCTGGGCTTTCAAGAGTCGCCTTTGTCTTGCACTACTGCATGGGCTGGCTCAACCTACCAACAATTATGTTCAACATTTAGCTAAAACCAATCTGTCTCAGTTTCGGTTGATTACTGAATAAGCATTGCATATTTGGTTCCCA

>TCONS_00070286

AAGTATACTAGAGCCGGTGTCCGCGCTTCGCGCGGATTACATATGTAATTAAAGTAATGTTTTATTAAAATTTGGAGTTTTAGTTTTTGAAAGTGTGAGTTAAGTTTTGTTTTTTTGGATGTGAAGACGTAGAAACAAAATAGTTTGACAGTAGAAATAAGTAATTGTCCAAAGGGAATAGAATCCAGTAGATAACGAAAAAGAATTAAAATAAGATAAGACGTAGACGATGTCTGTGAAGTATATTATGAATGATCTTGTAGAGCGGCCACTTTCTATTGCGGGCGGACCTATAGATAAAAACAATTTTGTTAGTACATCTTAATTATTGAGTTTGAAGAGTTTCGGGTGGGCAGACTTACTGGTAGTTTGCATCTTATGGTATAGAAAGTTGTTTAAGATTCCGGGAAGTCTGCTCTGGGTGAATAAGTAGAATAAGTAAATGAAATTATTTTGTTGGAGCAAGTAGTCGAGACATGTCTCTATTATATTTGGGCTTTTAATAATTGAAAAATATAGAACATTGCTGGATTAAGTGATTTAATTTTGTGTATACCTTTCACTTTATTCCGTACTCCTAGTGGACGTCCGCGTTTACGTTTCGTTGTGT

>TCONS_00060532

ACAGGATTTCTTTTCTCCTGAAGGAGCAGGAAGGCCTAATAATACAGGCTGTCACCCACGGTCGCCGGGGAGACTAGAATAGAAGAGTCCGCCAACTTCCTATATCGCTAAAATGGAAAATAAGGAGTCCGTCGACCCAAAGTCTTGTATGCTTGCTCTATGTCTTTGGTTTATGTACTATGTTCTTAGTTTCCTTTTTAATGGTGTGTGGCTGGTCTACGGTGTGTGTAGGCTTCCTATTTTATGTATGCTTGTAATGCTTCTGCTTTGTCAAATATTCCTTTGAATAGCCGGTCTTCATCTTCCTTAGATTAGATGCTACTTCCTTCGTCTAACGCACTGCCCCACAGATAATCAAAGTCTGTAATAGCGGACAATTCTATCAAACCCTAAAGTAGAAGAAAAAGTAGAAGCTACGAAACCATAAAGCTTCCCTCCTGTTTGAAACAGAAAGCATTGATATCATTGGACACTAAAGGGTCAAACTTTGCTTTTT

>TCONS_00075505

GTGATCAGAGAAAGGAGTCCTCAGTACTGCATCCTTTTTCTTCCTCATTCTTTGTATTCCCGCGAACCTTTTCACCAAAGTAGCCTAGGAATTGCCGTGATCCCTTTTATACTTGTAAGAAGTAATGGATGCAGCGTTCTGCCATTGCAGGGTTCGTTAGGATTGCACTGGCTTTGTTAGATCAATTTTTTTTAACATTCTGTGAAAGTCAATGTCATTCTCGAAGCTCTGTAATCATTCATAATGAAAAATATGTTTGCTGTCAATTCCATATTTGCTCAAATGGTAGGTAATCCCTTAAAAAATGTTTTCGCAGAAGCAGTCAAATGAAGTTGTACTAAAGAAACATAGAGACCCTCATATATTGGGATCAGTGGCTTGCTTTTCTTTCTAACCTATGCAATTAACTCATGAACCCCAGACTCTGACTAAATATCAACAGTAATTGTTGCTTCATCCCTAAACCACACGGCCCACACCATAGGATCTTTAAAAATTGCTATGGCTACCACTCTCTACTATTTTCTGCCTGATAACTGAAGTCCTCTTTCACCCACCATTGTATCATAGGCCTGCAACAGGGAATACGTTTTTCAATTTAGTTTAAATCCGGAAATTAGGGCTGCATAGTTGATCAAGTGCAGTTAAAAATACCTGTTGCAGTCCACTAATGAAGCCATGAGC

>TCONS_00052134

TCTTCCAGAAAATCCCATCTGATACGGTCGTATGCTTTCTCAAGATCCAGCTTTAGGAGCATCCATCCTCTTCGTCCTTTTTTCCTCCTCATAGAATGTACAGCTTCCTGGACAATAACAATGTTATCCGAGCTCAGCCGGCCAGGTATAAAGCTAGCTTGTGATGGGCCAATGAGCTTTGACATGAGCTTCTTCAGTCTCAACACCATTGTCTTTGTTATGATCTTGAACAAAACATTGCACAAGCTAATCGGTCTGAACTGCATAATCCTTTCGGGTTTAAGGACTTTCGGTATCAGCACCACCAGCGCATCATTCATGCCTTCCGTAAGAACCCCAGACTCAAAGAACTCCAGCCCAAAGCGAGTTACTGACGCCCCTACTACCTCCCATGAGTCCTGATAGAACACAGGTTGAAATCCATCTGGCCCTGGAGCCTTAAACTTTCCCATGGATTTAACTGAAGCTTCAATCTCCACCTCCGAGAAAGGTTCATCCAAGATCTGCACTTCTTCTCTAGTAAAACCAGTAAAGCCTTGTTGGGGAAGCTTCCTCGTGTCAAGATTCAGATCCTCCGTTGAGTACAGCCTCTTATAATAACTTATGGCCAGCTTCTCTAGTTCCTCCGATTGGTCTATCCAGCGACCATCCTCATCTCTCAGCATCTCTATTCTGTTCCTCTTGCGCCTGACAATTGTACTCGTATGATAGTACTTAGTGTTCCGATCTCCCAGGACTACCCATTTCTCCCGCGACTTCTGATACCATAACACCTCCTCTTGCTCCAAGGCAACATCAAGTTCTTTTTGAAGAACAGACTCCCTCAACAGAAGATCATCTCTTGGATTCTGTTCCAGTTTCTCCTGTATATCCTTGATCTCC

>TCONS_00072957

TAACCATTCGTCAGGGTAAGAGTGGGCCGTGACGAAATCAATGCCAGGGATGCGGTTATTGGCGATGAAATCTGTTCCAAACTGGCCTGGTGGGTTCAGAGTCTTGCTTTGAGGTGAGGACTGGCCGTAGAAACCTTCGAGGCCAGCTTCAAGCAGGTGGTTGGTATCTAGTGATTTCACATGAGCAGCCATTTCAGTAATCCAAGCCTGTTTTAAAATTGAAAAATAAAACAAAACCAACAAGTTAGATCAATAAAAAAAAAGACTATACAAGCTAAGATAATATGTTAAGGTGAAAACCTGAA

>TCONS_00060344

CCTGGGCTTAATATACTGAAGCCGAAGTCCATAGTATAACCCGCACCGAAGTATCGGTTTTGGATCGGTAGCGGACCCAAAGAAAATACCGATTGGACTTTAAAGCCCACAAATTTCGGCTATGGTCCGGTCCGGTTTCAAACCGAGATCAAGACAAGGTAACCGAAACATACCGACCATTTATATATTTCTATTACATATACATACATTCATTTATACCGATACAGAACTCATCGGTCTCTTCTCTTTCACGCGTGAGGCGAACAGCTGAGGCGTGATTCACGAACCCTAGTTCCAAAAAAAAAACTCTTCGGTTATTGATCATCGTCATTAAGAATCTCAAACTGGCCTATATAAATGTCTTACCGTCTCCATCTTCTTCTTCTTCACACGAGCAATCGAGAATCCAATCAGAGATGGACTCAACATCATCATTTGGCCGAGACAAAGACGATGCTGGAAACATTCAAGCTCCCAATAGTACAGAGACAAGGAAAGAGTCAGAGAGAGCTAGTGGAGCGACAAGCAAAGTGAAGCGTATTCTCCCTCCAAGATCCGACGTTTGGCAGCACTACACAAGGACTACAGAAGACCGAAACAAGTGTGTTTCCAACTACTGTCAGAACACATTTTCGTGTCTCACAACCTCAGGGACGTCCAATCTAAGGAGCCATCTTGAGAAATGTAAGAGCCATCTTGCTTGGTCGGCTGGACAAAAC

>TCONS_00064880

AGCTAATGGCTCTGGTGATAGAAGACCTTGGAGGTATCGCCATCTTCCTTAATAATAGATGCGATGTTAGCAGTGGATAAATCGAGCGCACGAGTGTGTTCATGTTGAAGCAATGAGGTATCTTAAATCAAGTAACTTGTCTGACTTTTTGTATGTAAAATTAGATACAGTGTTTCTTTGTTGAAAAAGAGTATTTCATTGTTGGCAAGTTAAAGGAACCATCGAATATTTAGCGAAAATCTTACGTTGTATTTTGCATAAGGACTTGTGCATAATAATTTGTATTGTTATTTTGATGTAGGCAGTTAAAAAAGAAAAAGTTGTGCATTGGCCTTTAATTTCACTTGCAGCGTTAGTTCTAATGAAATTATACAATTATGTCGAAGTGATGGTCTGATCCTTGTGTCGCACTATTGTGGATATGCTTTTTTCATTAGGATCCTTCCAGTACAGAGAGCTTCATGTTAACTTGGCCAGGTTCGTGTTAATAGATCTGTAAGCTCAAAATGGTAGAGCTCTCGTTATGAGAGGTTGGTGGTTCGAATCCACCCAGATCTGTCTACTTCCTAATCTTTTCACCTCTTTTTCGTGAACGTAATTTAGAATCCACTTTAGTCATGATCTATTAGTGTTTTTGCACTTATTAATAGGGTATGCTAAAACGGAATTGCTGACAAGAAAAATATTTACTTTTGATTTGGCAATTGACATCTGATCTCGCATCATGATCTCGGCAAGCTAGGTTCGTCGGATCCGTGATATGCTGAGGTGAGGATGAGTCCGGGAGGAGCAATTGAAGAAGAGGGGAGAGGATTGAGATATGTGAATTGATAATGAGTATGAAGGATGTATAAGATGTTTGTTAAGGACGATATAGCCTGATGGATTAGGCTTGAGTTCTGATTAGCATCTTTGTCATCCGACCTTTGCCATGACAGGTGCGCTTGCATGGCAGGTCACACACTAAATAATAATAAAAATAATTGTTTCGTGTTGGTTAGGGGAGTTCGCACGGGGCACTTTTTTTTAGCCCGGGTTCTCCCCTCACCTTTGTGTCTTGTTCTCTCTCTCTCTCTCTCTGATCCCTTTGATCTCAATTCACCAAAATATCTCTCGACACAGACATATTTTGAATCGATTCCTATCATTACGATTGATCGGATCTGGAGTCTAACGCTGCCATATCTAAATCCAACATACTCCACAGAGGCAAAGCCGCTTTTTTTATCGTTATCTCTTTAAGCTTTTCGTTCATCGCTTTTCTCTAGATCTCTCCGTTACGTCGATTTGATGACGTATTGGAAGACTCTCCGATTCAGTTCTGCCGTCGATCTTCGAATCGGCGTCGTATACACAATGGATCTGGTAGCTCCGGTTTGATTCATGATCCAGAAGACGGATCTTAGCTGCTGAGATCTCCGATGATCGGTTGAAGAGCTG

>TCONS_00009147

GATCCAGGATCCATGTTGAATCCGAGGTTTCCAAATCTTGAGCGAGTTCCGGAGATAACTCCACGAGAAAATCGGCGAGGACTTGAGCCTTAGCAGCGGTTCTGCACTTATAGGTGATGTCGAGCTCTCCGAGTTCGATCGCCCATTTGGTAAGACGGCCGGCTCTGTTCGTGTTTTGCAGGACAGTTCTGAGCGGCTGGTCGGTGAGAACTTCTACCGAATGTGACTGGAAATATGGGCGGAGTTTCCTCGCTGATTCGACGACGGCCAGGGCCATCTTTTCAAGTGTCGGGTACCGGGTTTCTGGTCCGGTCATCCTCCTGCTCATGTAGAAAACAGGTCGTTGTTCTCCTCGGTCCTCCTTGATTAGCACGCTGCTGACTGCGGCGGATGAGACCGCAATGTAGAGAGATAAAACGTCGCCTATGTCCGGCTTTGCCAGGATAGGGGGCGTCGTCAGGTAATGTTTGAGCTGACTAAAAGCCTCCTCACATTTATCGTCCCAAATGAATTTCTTGTTTCCTCGGAGGAGATCGTAGAATGGGAGGCATTTATCGGTGGATCGGGAGATGAAACGATTAAGCGCTGCGATTCGGCCTGTTAGTCGTTGGACTTCTCGGCAATTTCTCGGACTGGGGAGATCAAGGACAGCTGATATTTTCTTCGGGTTTGCTTCGATTCCCCGCTGCGTCACTATGTAGCCAAGGAATTCTCCGGACGATACCCCGAAAGTGCATTTGGCCGGGTTTAGCTTCATCTTGTACGCGTTTAGGATGTCGAAACACTCCCTTAAGTGTGCCAGGTGGTCGTTTGCTCGGACTGATTTGACGAGCATGTCATCGATGTAGACTTCCATCGTTGTACCGAGCTGTTTAGCGAACATGCGGTTGACAAGTCGCTGGTACGTCGCACCGGCATTCTTCAGCCCGAAGGGCATTACTTTGTAGCAGTAAGTTCCTCGATCCGTTATGAACGACGTCTTCTCTCGATCGTCGGGATGCATCATGATCTGGTTGTATCCCGAGAAAGCGTCCATAAACGTGAGCATTTCATTGCCAGCAGTTGACTCGACCAGTCTGTCGATGTTCGGAAGTGGGTAGCTGTCTT

>TCONS_00013069

GATTCACATTGTCTCGCTCGCCTAACACTTACCCCACTCACACACTCTCATCTTCATTTCTCTCTACCCTTTATTAATTTCTTCCTCTCCTCTCCTCTCCTCTCTACAATTATCAAATCCATGTTCTAGCGCTGCTGTACAGTTAACCTTAAGGTATTCAAAATTATCATTTAATTGTTGGGAAGAAACGATGAATCAGTGGGCGATTCAGCCAAAGGCTTTCGCTGCGGGAGGAGAACAGAGTGTTGTAGTTTGCCCAAAACCACGTCGTATTGGTCTCCGTAACCATCCCTCCTCCCGCTCTCTGCGATGTTACTTCAGCCAGCAAGTTGAATCCAAGGCAGAGACTGATGATATCTTAGATATCATACTCACCACGGTATAATCTATTTTTTTGGTAGTTCAAATTTGATTATCAAACCGGTTACGTCTTGGTTCATCTTAACCAACAAATCAGTTGAAAGTCGGTTTCTCTCTTTCTCTATATTAATTATTATTATAATATTTCAGAAACGGAAAAATATATATAGAGAGTAATCGTGTACGGATCTTGATTTAAAGAACACACATGTTGCGCGTTGAGCCTTGTCGAAAGTTTCTTTTCTTTTCATAATAAAATAAGACGTCTCGTGTTAAATCCTTACAGCACATACGTGGGCCTGTTTCTGACCTTTTTTATAAATATATATAATATGCGTCTTTTCTTGAAACCATTTAGCCACATGATGATGCTTTTAGTTTTAGTTAATCAATCACTCTCTCTCTCACGCGTCATCAAAGCTGTTTAAACTGATGATGTCTTTGTTGTGTGGTGGTTACAGGAACAAGTTAATCATTCGCAGGTATTAGACTCGCCGTCCCCGTTTTTATGTGGGTCGCCGCCGAGCAGAGTCGCTAACCCATTAACACAGGATGCTCGTTTCAGAGATGAAATCAAGCTGGTCTCTTCTTCCCCGATCACGACTCCGCTGGGCCATCCTCCGTCGTCCCCTTCTTCCTCTGGGAGGAAAGGAGGATGTGTTAGAGGCAATTTTGGTAACAGCCCAGCGGTTAGGATCGAAGGGTTTGATTGCCTTGACAGGGACAGTAGAAACTGCAGCATCCCTGCCTTGGCTTAGAAACCAAACCAACCTCGAAAACGCATCTACTCTGTTGAATTATCTCATGAAAACCAATTTAGTGCGTCCGTACATATATGAGTTTAAGGATATGGAGATGTGTGGGATATAATCTTTGAGGGAAGTGAGATCAATCTTTTGGAGTTTGGCTGTTTTGTTTTTAAAGAGTGTAAATAGGTGACGGAGTAAAACCAGTTTTCTGGGTTAAGTTTTTAGTATTCGTGGTCATTTTGTAAGTATAAGTATCAAGGCTAAGGATGAATCGTTGAGGAGAAGGAAGAATAATATTTGAAGGTCAGGTTTTCTTGTGTAAAGAGAAAAGCGAGATAGAAAAGTGCATTTTGGGTATTTTCTACTTTTGTTTTTGTTTCTTTTATGTGTTGG

>TCONS_00018097

GTAGCAGGAGCTTCTAGAAGCTTTGAGAAGGGAGTGAAGCATACTCTAACGGAATTAGAGAGTTCAAACAACAGTTCAGGAGTAGAGGATAGCATTCCAAGTCTCCAGAGTTTAATCACATCAACACCACAACGTCCTACTTCTACTTCTTTGAGAAAAAGACCAAGAAAGGGTATCCCTCACCGATCTCTACTGCAATGAGTTTAGATCGCAAAGAAGCAGGAGGTTGTGTTACATTCCAAGGCTTCTTATTCTATATTAGACATCCTGAAAAGAGATAGTAGCTCAATACAAGACACAACATAAATGGTTTTAACATTGGTCTTACTCCAATGGCGATCGAAAACCACGAGAACCCAAATCGCAGAAGAAGCATGGTACGTTCAATATCATTACATTCTTGACTCTGTTTTCCATTGTCCACTCTGTTTTTCAATGTCTACTCGCAATGCATTATCGATTCTAAGATTTGGGTTGTTGCTATTGAACTGAAATTTCCACTGGAAACAAGAAAAGTTTTAGTCTTTCCAACATTACTTTCCAGATTTTCAAATTCCAGTTTCTACTGAAAATGCTCTGTTTTGTGTATAAAAGAACGTCAATGCTCTGTTTTGATATGTGGAAGGTGTTTCTGTTAGATTAACGAGTGATCTTGTTTATCTCAGGGAGGTGGTGGACCAGAGGAAGAAGAGAACCAGTGGCCTCCATGGCTGAAACCATTACTGAAAGAAAACTTCTTTGCTCAATGCAACTTTCATGGACACTCACCTAAAAACGAATGCAACATGTATTGTTTGGACTGCACTAATGGCGGCTCCCTTTGCCCTCTCTGTCTTGAACACCACAAAGATCATCGTACCATCCAGGTGCGTCGCGTTTCAAGGAGGATCCAGACGTTCTCATATATAGATTGCATTTGGAAGAAACTTGATTTTTGAATGTTACTGCTCTTGATTTGGGCAGATAAGGAGATTGTCCTATCACGATGTGATAAGGGTCAACGAGATTCAGATGCATTTAGATATCTTGAGCGTTCAGACGTATGTGAACAACAGCGCAAAGGTTGTGTTTTTGAATGAAAGGCCTCAGCTTGAGCGCGTTAGAGGAGTTAGAGTTACTAATGCCTGCGATGTCTGTTCTCGTGGCCTTGCTGACGATTGCTTTTGCTTCTGCTCTCTTGGCTGCAAG

>TCONS_00039924

GGAGTAAATAAATAAGTGTTAAGAGAGAGGACTTGCTAACCGAACCCTTCAACCGTGTCCATACATCTTCTATAACCACTGATGGGATAAACTACGTCGAGTTTCAGGTTCCGGCCGAACTCCGGCGATAAACCGAGTACAAAGCTCGTGACTCCTCCGAAGTTCGCACCGATGGCGAGCACAGAGGACGAGCCTATACCAAGAAGTAGCCGCCGGTTGAACTTCTCAGCAAGGGGAGAGAACTCCTCGGCCGGGGAGGTTTTCCGGTCACCGGGGGATTGTTGAGCGATCAAAATGCTGGAGGGTTTGGCATTTTTGAGTGATGATGAGTGAAGAGAGAAGTAAAGTTTTAGAGACATTTCTTGTCCGTTCTTTTAAGGATGTCATCGAGTGTTGCAGACTTCTTATCACTTACGGAGCTAGTTGTTAAACGACAGCGTTTTACATTCGACAGTGATTTA

>TCONS_00017966

ACCGTTACTCAGTTAGACCCGGCCTCCTGCCATGATCCAACTTCCAAGTAACGGGACCCTGCATAAAAACAACTCAGCAATCAATTGATTATAACTCACAGTTTTTGGTTGACCGTGGCCTTGACCCCAAACCCGACTTCTGTGATCCGAACCCTTTCCCGACCTTCACAAGTAGACCCACGTCTGGACTGATCTTCACTTGAACTGGTCTCCACTAGAACTGATCTCTCTTCACTTGAACAGAATCTTCTCCAAGTGCTGACTCAAAATCGGCAGAGTAACTGTTCTCGAAAACTGCCTAAATCGCTCGAAAACTATCGAAACTCGATCTCTCATTCTTT

>TCONS_00082279

CAGATGTGAAGCCATGAAACGAAAAACTCTATGGGAATTGTACATCTACTTTTTCTTTCCACTGTTGTAGGGTGGATGGTGGCATAGTCCGCATATAACCACAGCCTCGTAGCTCTAGATTCTGTGGTCCCATAGTGGTTTTAACACAAATTATTTAGAAGCAGAGATGTCTTAAGTTGTGGGGAAAGCAATATTCTGCAGGGTACCTTTGAATACTCTACTGTGACTTGGTGGACCACGGGAGCATCTCATACTTTCCCGGGAACATTTTGCAGGTCTGATGGGTGGTCTCTTTTCCAAGGTATGGGCTTCTTAGCCATATGGTAGGAGTCAAAGTGTTGCTGCAGGGAAGGTTATGAATTTTGTTCTGGGAGGATATATTTATCAACCGGTTGGAAAACAGGTTATTATGAATCTATATATGAGTAGGAATATTTTCGATTTGTTCATGTTATTATTTTGTAATGTTTTCTCTTATTATTTGCCGGGCCCTACTTCTAATCAGTGGCGGATGTACGTTGAGTGGAATGGGGGCAGCTGCCCCCAGTAAAACAAAAATAATCAGTGTATGTTATTAAGGAAAATAGTTCTTGCCCCCGTACAAAATCATATATTTGCCCCCATTAATTTATTTTGTTTGTTCAGTCATTTTATATGATAATGCTTTTTAATTTGTAGAGTTTTGTGTCCATCAATATATAATAATCAAGTAGATTCACTTACTTTATTTACTAAGATGATTAGTATTACTTCATTATTTTTTATATTTTCGTACATCCCATATGATATTAGTACCGTAT

>TCONS_00046134

ATCAAACCGTTATCCAGATAGTCCAGCCTCCTGCTATGAAACTATCCTTAAAGATAACGGGAACCTGCACTCAACCATATCAACACGCAAGAATAGAAAACATGATGCATCCTAGCCCTAGTCCTAGTCAGGTTATTAACTCAGTCTTAATTCCATTCATCTCAGCTTAGCCCTTGCTAAACTGAGTCCGGTTCCATAAATAAAATAACCCTAAGGACTCTACCATTCAATAACGTGATCATTCTACTCTATATGCGACTCGATCTACCCTAAATTCCAAGTGGAATTCAAACAAACCAACTCACAGTGTTCTAGGCGAGAAGCAGCTGGTTGATCGGAGAGGACTTGGCCAAAACCCAAGGGACGCCTTGACTGGACTGGACTGGACTGATCTTGACTTGTACAGAACCTCCCCTGATACTGGTCTGGACTGAACTCAACTCGAACAGCACCTTCCTTAGCTTCTGAACAATTCTTCCGAAATCTGAACAGAGTATCGAACCATAACCTCAACTGAACTGAAACCATGGAACTGAACTGATCTTGGACAGCAC

>TCONS_00075465

GCAAGACCAACCGCATCAACACCAGACTGGTGAAATTTCTCCAATAGGGCTTCTGGAATTCGAAAAGCTCAGGATTTGCATCTTGCAAACGATCCACAGTTCAATGCAAATCGTTACAGAAGAGGGGACTTCGACCTGAGGAATCCAGCGTCCACTCAAGACATCAAATTAAACTCCCGCTTTAGGGCTCCATAAAATTTGAGTGAGGTACATGGCCCATGAGCAACAACACCGGGGTGCATTGTCCCGAGTGAAGGCTCATCCAAAGGAGGTCACTGCCTCTGAACTTAGGCACAAAAGGGAACTGTGGATCCTAAAACTCGGGTTTTGGCGGCAGCCATATCATCACTTCATCCGAGTCCACATTAGACAGGAGTGAAGAGCTCAACCACCGGTTAGCACGAGGGCCAGACCAGCGCTGGGATATTGAAGCTCCACCATATGGTTTATCAAAGCTCCACACCTCCAGCTGACAACTTCTGAGATGTGAGAGCCTGCCATATGTCATCGCCGAGAACCGGCCAACAAACTCAAGTCTTTCACCAACACCTTAAGGTGAATACCGGCTTGTAATCGCACTGGAAAAGAGAACTTTCTCCTCTCGGTTTGCCACACTGTGGTTTTACAACGAGGGGTGAAGTGTTGACACCGTGACTGGAGGAACCAGAGAGAGAGAGACCATTCTTCTGAACAGAACTGAGCCATAGACGACTCAGATTCCAGCTTCAGTCGGATGAACTACACGCGCCGTCTTCAGGGGATCTCCATCAGCACACGCCAATCAAGCTCTCCTGCACGCGTCTCCGGAGGCATGACCACCAGAATCCTTCCCGAACAAGGTCTAGAGAGGCGTTCACACCATATCGGAGCATAACGCCTCTGACCTAAGTTCCACTCCTTTGAATACCTCACCGCTACAGAAAACGGAAGGTCCAAGATGGAGGAAGTTCCTGAACCAAACCACGCGCCACTGCCGCGAAAGCACCGCCATAGATCTGAAGGGGAAAATAGATCCGACGAGCCGTCAACCCTAAACGCCGACTCTCTCAACACCCCCTGCTCCTTCCACCTCGCCGCTGCTCCATGTCCGTCAGTAACTGAAGGCCTTCAATCCTCGCGAGCAGATCTCCGGCGTTAGGTTCCCGGCGAAAACAGAGCAGAGAGCATATCGGATGGGGATTAGGAAGTGTAGAAGAGAGCAAAAGGAAAAAAAGAAAAG

>TCONS_00010576

TAAAAAAATCCTCAAATCGACCCTTAGAACCCTAAGCCCCAATTCAATTTCACTCTTGTTCTCTTCCTCTGCAACCCGATTTTGTATTTTTTTGTCTTCTCTGCGACTTGGTTTCATCTCTTTTTATTACAGGCTAAAATCATGTGAAAGAGAAAAGTTTGATTCTGTTAGAAAAACGAGATTTTGGAAAGAGAGATTTGGCATCGAAGTGTGTGTTGTGGGTAGAGTAATCATTGCTTGAAGAGATTGAAGATTCACTTCCAAAACTTGAAAACATTCAACTGAGTTTTGTAATATAAAAGGCTAGGTTGATTTCTTGAAGAAGGTGACTGATGACTTAATAGAGGATGTTATTTCGACAAAGTTCGAGATGAGGAAGTGGAAACTCATCACTGAGACATCTTGTGGACTCTTTTGTTTTATGTTGGTTTTAATCACAGTTGCCTATCTCATCTCTTAAGCTCTTGTATCTTTGTCTCAAGACTGTATTTGTTGTTGAATGATGCCTATTTGTTCTTAAACCACAAAGCATTAAACGTGGG

>TCONS_00009682

GTTGGTCTTCCAGCTCAGAACTTCCCTGAACTCGGCCTCTCGGGTTAAGTGATGTAACAGTGACCCATGGATCATCTCTGTTCCTAACCCGGGGGTACTTGATATAACAAACCTGTAACATTTAAAAAATTATTTAATTTGTAAATAAGTGTTATTAATACACATGATGATGAATCATTATGAATAATTAATATATGTATATTTAATTACATGATCGGCCTGAGAAACAAGAATGAAAGGATCATCATATTGCAACTTTCGCCTCGAATGTACTAATGTAACACCAAATGCATCTGTTCTCACACCTCGATCTGGAGTGTTGTCGTACCAATCACAATAGAAAACAATACAGCGCAATCCAACCATGCCCGGATACTTGATTTCTATAATCTCCTGTATGTTTCCGTAGTATACATCATCTCCAGATGCAGAACAAACGCCAGCATCATAA

>TCONS_00019084

GTCTTGCTCCAGGAGGAGGTGCTGTAGCTGCTGGACCCGACTTAAGTTTTCTAGCTCCAAACCCATCTCCAGGGAACCACGGATCTTCCTTGCTTCCCTTCTCCTTCACCACCATACTCAGCACTATGTTCTTCGTATTGTTCTTGTCTCGTGTCTAGATAAAAGCCTTATTCACCAGCCCGATTTGGTTGTATGAAATAGCTTTGGGTTTAATTTGGGTTTTCATGACTTTAATAGTTTTGCGTGTCTTTCGAGTATTCTGTTTCAGACCATGTCGGGTTTATGTTCGTAGTTTTACTCTTTAGTTATGTTTCACATTGGTGTCCGCCGCTCCTACTATGGCACCAGATACAAAACGTCATCGTTAATGTGCAACAGCAACACCCAAAGCAACCGACCTACAAATACCCCCTTGTCCTTTCTCCTCCTCCTCCTCCTCCTCTTTACTCTTCAAAGTCTCCGCCACACACTTCTCTCCATTGAAACTCCTCTCATCTTTCACATAAACACCTACAATGGCCACTTCATTCTCTACCTTGACACCTTTTCTTTTCATCCTCCTTCTCTCCATCTCCTCCGTTCTTGAAGCAGCTCATCACCACACGGCGGCTCCAGCTCCGGCTGTAGACTGTTCGATGCTCATACTCAACATGGCTGATTGTTTGTCCTTTGTTTCCGCTGGAGGCACGGAGGCTAAGCCGGCTAGTTCTTGTTGTAACGGGCTTAAGACGGTGCTTAAGACCGACGCAGAGTGTCTATGTGAGGGGTTTAAAAGTAGTGCTTCTCTAGGCGTTACTTTGAACATGACTAAAGCAGCAACACTCCCCGCTGCATGCAAGCTTCACGCTCCTTCTATGGCTGCTTGTGGAT

>TCONS_00060837

GGCAGTTGCTCCTCCTATTCTGAAGAAGCGACCCCAAATCTTACGCTGACACTCCCTTCGCGGATAAGATAGCGCTAGTAGAGATGCCAAGAAGTTCTCCTTTCCCAACATGAGGATGTACGATGGCACCAAGGACCTAGATAACCATGTGGCCCAGTACAAGCAACAAATTATCACTTTAGCAATCCAAAAAGAGCTAAGGGAAGCCATTATGTGCAAGGACCTCATATCAACCTTGATAGGATCTGCCTTACAATGGTCCAACGGGTAGATACACTCCTTCAAAACCGTGCCAAACAAGTTTGTAGAGCAATTCACAAGGAGCATAAGTCCCGAGAAGACGACTGACAACCTCTATGAGATCCTTCATCATCGAGCCGAGCCCCTGCGCAGCTTTATAGCATGTTTTAACCAATAAAAGGTAGCCATCCCAAGATGAAATGCCTCCACCGCAATCTCAACCTTCAAAAGAGGCTATTACGTGATGGAGACCTATATAAGGAACTCAACAAATATCAGTGCAAAACTATGGAAGACATATTATCCTGTGCATGGGCCCATGTAAAATGGGAAGACGATGTCGCGACTCGATCCAAGTTCCCTCTGAAGAAGAACCAGAAACGACAAGAAGAACCGAGATGAGAAATCCTATTAAAAGCCTGGCAAATAGGCAAGAAGCAAGAACTGGTGTAGGTATCAAAACCGACCCCTGAAGATAACCGAAGGGATAACATTGTCCATGTGGC

>TCONS_00046989

GTGAATTTGAACAGTCCAGAATCTCGAGAACTCAGACTCATCTGCTCATCCTCATCCACCTCCATATAAGTCACCAAACCATTCATCTTCCCAACCTCCACCTTCATGGTCTCTCTTCTTCACACGCTCTCTCAATCTCTCACTCAATTCAGCATCCAGCCGCAGCTCCAACACAACCACCAGAACCTTATCAACTACCATCTCATCCGACCCCACTCTCTCTTACCTCGATCCTACTACGGTGGTGTCAAAACAGTCACCGTCTCCTCCCTAATTGTCGGGAAGAAAACCATCCTCTTCGCTGTCCCATGATCTTTCACTCCAACCTGCTTAACACGTCCATGGATTCGTTTCCAAAGCCGGAGAGCTCAAATCCAAATCAAAGAAGGCGAGAAACAACGGTATGTGAAGCATGAAATGATGAATTACACGGATACAAACTCAACTATTTATAGTCGTAAATTCACCGACTTGAAAAAGAAGATATTATTGAATGAGAAACGGTAGAATTAATGGGGAGAATGAATGAAGAAGAATGTTAATGACTACGGTTGAATGTAGTCCGTAACGCTTCTGGTTTCTCATTACCGATGAGAAGAACTCCAGCCGACCATCGGAGATTGGCCGTTATTAATCGTCTCTCGAGAAAATATAAAAACCCTAAAAATTTGGAGTCACTATTCTTGAAGAAAACAATAGTGAGAAGACAAAGGTAAATTGGGCTTAACTTCATTTTACACTTTTAGTCCCAACCAAATTTCAACTCGTTTAAAAGCCCATTAGAAACCAAAACGAAATCAGAGTTTTTGTTATATACACACGTGTCACTCT

>TCONS_00049015

CTCTGTGTCTCTCTCCCTCTCGTCGACTATTCTCCAAAATCTCTCAGACAATACTCGATGGCGACGATTCCTCCCCAATTCTTCAGAGCAAGTCAAATATATTTCTTATGTGTGTTAGATTATGGACGGAGGAGGTGGCGACGGTGGTGGTGCCGCGTACAACTCACACACGGTGGGGGAGGTTTTAGGGATTTTAAGGGTCGTAGGAATGGCATGATTAAGGCTCTAACCACTGGTAATATTTGGTCACTCGCTCTTCGTTTTGATTCTTCTTCTTCCTCCATTGTCGTTAAAAATCTCAACTTTTTTATTGTGTGTGTGTGTTTGGCTCTGTTTTGCTGAGTGTTGAATGATGCATTAGGGGTTTTGATAGTTTCTCTGAATTATGAGCAATAAAGGAGTAAAGTTTTGTTTTTTTGTTTGTGGGATTGATTTCCTCCGATGAAAGTCTTTGCTTTTTTTGTTTGAGTTGTCTTCCTTGTGCTCTGCTTCTCTGAGAGTAAAGTTTAGATTTTTGAATATTGATTTTCAAAATAATGTCGTTCTGTGTGAAATCTAGTTTCGTAGTGTCACAAGTTCTTGATGAGCAGAAGCTGTGTCAGATACGAAGAAAGCTATTGAGTTGGATCCTGCATTGGCCAAAGCTTACTTAAGAAAAAGGTATGTCTTTGTTGTTTCATCTTGATGTATTGATTGAGGTTGGTGAAGGAGAAGATTGAGTTTGCTGTGAATCAGATGAATTGGCCACTAGAGGTTGTGGCTTTGTTTCCTCAGCTACTTGGATACAGCATGGAGAAGGGATTGTGCCTAGGTGTAACGTAATCAAAGCTCTCATCGCCAAAGGGTTGCTTGGAAGTGAACCCCTTTCGATTCCATGTGTCTTGACATATACCAATGAAGCCTTCTTAAAAAGGTATGTGATGAAGCACGATGATAAGCAGCTTGTGACTTAGTTATTAGCCATCTTCATGACAGATAGTAATAAAATAGATTAGAAAAGATATATGTTAGTGTTAT

>TCONS_00017861

GAAGAAACAGCCGGGTGAGGAACCTGCGACGAGGGTCTTAGCGGCAAAGCTGTTGAAGTTGGCGTGTATACTGGTTCCGTGTCGGTGTCTGTAGTCAGAGATCGCGGCGACTGCGTATCAGAATCAGAGAACGGGAAAGTGAATTCATCAAATTGGACATGGCGAGAGCAGTACATCCGATTATTGGCGACATTATAACACAAATAGGCGCTCTGGGTTGGTGAGTACCCAAAGAACACACATCTTGCAGATCTATCTTCGAGCTTATGACGAGTAGACGGCTTAAGCCATGGGTAACAGGCACATCCGAAAACCCTCAGCTTGTTATAGTTTGGTGATTTCCCGAAGAGCTTGCTAAATGGAGATTGGAGCTCAAGGAGAGGCGTTGGAAGACGGTTTATAAGGTAAACCGCAACGACGAACGCAAACTGCCAGTATTTCTTCGGGATGGAAGCTTTGGAGAGAAGTGTAAGTCCTGTTTCGACCACATGGCGATGTTTCCTCTCAGCAATGCCGTTGTGTTCTGGAGTGTGAGGTGGAGACGTCAAGTGGGAGATGCCGTTCATCTGAAAATACTGTCTGAGCGCAATGAACTCTCCCCCATTGTCTGAGTAAAAGGTACCAATCTTGGCCTGAAGAAAGTTCTCAATTAGATTTTTGAAAACGATAACTGTTTCTTTGACCTGAGATTTTTGTTTGAGGGGAAAGAGCCATGTGTATCTGGTGAAATGGTCAATAATGACTAGGTAGTATTTGTGATTTTCAGAGGATAGAATAGGAGAGCTCCACACATCAGAGTATAGATATTGCAAGGGTCGTATAGAGGTGATTGTAGATTGATGAAAAGGTAGTTTATGGGTTTTATTAATGAAGCAATCATTGCATGATAAGAGTTTTTGAGATGGTAAAGAAACAGGAAGGGAAAAACTGGAAACAACAGTGTTTAGAATTGAAGTAGAAGGATGGCCGAGACGCGAGTGCCACGAAGAAAGTGTGGCCTTAGCACTTGGAAGAGAGGCCGTGTTCACTTGTGGTACTGCTCGAGGCCACTGATAGAGTTCATTCTGAGCTTTGCCACATAGTAACTGGACCCCCGTTGACAGATCCTTCACCTGAAAAGATTGTGGGAAAAATTCCACTGATACTCCATTAGAATTGCATAGCTTATAGATAGAAATAAGATTCTTATCTATATTGGGTACATAAAGCACATGTGGTAGTTGTAAAGGACGAGATAAGGTTGAGAGAGTTGTGAAACCAGATTGTTGAATTGGCACAGTGGATCCATCAGCAAGCATCACTTCACTGGTGCCCTGGTACGGATGATGCAAGGCCAAATTGTTGAGGTCTGATGTAATGTGATGAGTGGCACCACTGTCCAGTAGCC

>TCONS_00054815

AAACATGGTACGCATCGCCCACCACCAACTCCCTAATCATTTAAAGGTCTGTACTGATGCGGCGTGGAGCGAAACACAAAAACAACGGTAGGGGTGGGTCTTCTCAGATCATCAGAGGCCATCTTTCTACCAAGGAAGCAACACAGAGGAATTTGTCATGTCGCCGTTAATAGCCGAAGCTCTAGCAATCCGACAAGCTCTCGAGGAAGCCAGGAACATGGGCGTCACCAACTTTCTCGTCAACTCCGACTCTCAAATCTTAATGGAATTATCCAAAAGAAAGACTTTATCAAGGAACTTATAGGCATCCTGCAGGACTTATATGAGCTCTCTTGTTGTTTTTCATCTATCTTTTTTAGCTTTATCCCTTGAGGGAAAAACAGTCTAACTGATTCTGTCGCGAAACATGCTTTGTA

>TCONS_00060776

GCGTCATAAGGGCGACGTCTCTATCTCTCTCGTCTCTCTTTCTCTCCCTTGGTGGCTCCGGTCGAGCTCTTCCGATGGTGCTTCCCCTTCCCGCCCTTTGAGGGACATGGTCATCACCACCATCATCATCGGTCCATAGCAGGGACATGGTCATCACCACCATCATCGATTCCACAAGAGAGGAAAGAAGAGAATGGTGGAGGCGGTGTGGTTTACTACGGCCCAATCACATCAACATTGGGGAAATCAGCTTTGAGTGGTTGCAGTTTATTCTCCAGAAATGCCTCCATTGATGTTATTAGTTTCTCAGGGGTCTTGCTTTGAACCGGAGTTGGGATGTGAGAAATTACCTTATCTCTAATCATTGAGATTAGTTCAATATATGGATTTGTTGACAGCACTGGAGTTGACTCTTAGGAAAGCTCGTGCTCATGGTGGTGTTGTTCGTGGTCTCCACGAAAGCGCTAAGCTTATTGAGAACCGTGTTGCTCAGCTCTGTGTATTGGCTGAAGACTGTTAGCAGCCTGATTACGTCAAGCTTGTTAAGGCTCTATGTGCTGATCACAGCATCAACTTGCTTACCGTTCCAAGTGCCAAGACCCTCGGCGAATGTGCTGGTGTAAGTCCCTTCTACTGTGACTCTCCGTATATACGTAGCTTACTAAAAGCGTCTTTTTTTCTCGAATGAATGTTTGGTTGAAAAATTGAACCTTTGATGTTTCAGCTCTGTAAGATTGACTCCGAGGGTAATGCTAGAAAGGTTGTTGGATGCTCAAAATTCTCTAGAAGGGTATGAAGGTAGTGTGTCTGCATTGCTTATCTTTCTAATGTCTAGAGTTTTGCTGGTGTTTACAGTGTTTTTAAAAACCCCAAAAATAAGAGACTTGCATTGGAGGTGCTAATTGTAGGAGTCTCTTAGTTATGTCTCTTAATCTCACTTTAACTACTTAAAAGTATTAAAAAAATATTAAGAGACCCAAGAAGGGGCTTTAGGATAATCATGCTCTAATGTTCATCTAATCAATGTCAGTACCTCCTAAATTATATGATAAAACGATCTAAATAGCCTATCAATTAGCTAATTGGTGTTGTTAGGTTTCATTCATTCATGATCTGATTACAACTTACATTACACGACTTAAACTTAACCTAATGTTGTTCAGCATCGCAAGATCCTCCTTCTCCTCTTTCGCCTAATCTAATTAACTCATAACCACACTCTCTTAACCGGAACGAGAACTTATTTGCCGTTAGTTTGGTTTGGTTTGATATAATTTTGTTCGGTTACTTTCGAGCCGATCCAAATGAAATAAGATTAGGGCTAAGTAATTTTACCTGGAATCTGCAGATGTAATTTTTTTTTTTTTTTTGAATTATACAGAGGTATTCTGGTTCCACAGAAGTGATCCAGACTAGTTACGTGTTGCCACGTGTCGGTTCTTTGTCCCTGACAGTGCCGAAATGTTAATTCCCGAGTGGCTGAAATTCGAACCCAGGTGGCGGTACTCACAATTGTAAACCCTCGAACCCCGGTGGTGATGTAATAGTCATCACCAAAGACACGTCTAAACGTCGGCATCGGGACACAATCTTTGTTCCTCGGAGAGAAGGCAACGCTCATGTTGACCAGAGCCTTGACTTTCTCCGGTCGGTACATACACAACTGCCACGCAATCAACGCTCCCCAGTCATGTCCACCACGAAAACCGCCTCATCTCCACCGGCCACGGCGTCGATGAGTGCCTCCAAGTCTCCGACCACGTTTGAGTAAGTGTACTCCTCCACTTTCTCCGTCGCATCGGTGTCTCCATACCCTCGAAGCAGTGCTGTGCGAGGAATTTTGGGGGCTCAAGGCAAATTTTAAAAATATTTTTTTTTACAATGGTTATTAGAAAATATAAATTTTAGTTGTGATAGAAACAATATAAATTTGTAATTTAAAAGTATAGGTTAAAATTATAAGTAGAAAACCGCTATTTTTAGAGAAAGTTAAAATAATAATTTGGTATTTTAGAAAGAGAAAAAATAATCATTAGAATACAAATGATAAAATAATAAAAATAAAAGTGACACTAACAAAATATTGAATAAAATAATGGTAGATAAAGTATACAACATTTTGTCGTTTAAAAAATGATGATAGATAAAAATATATATGTTAAAACGTATCATTTATTTTTTCTATGAACTCTTTCATCAAACTATCATACTGTATAAATAAAAATACAAACAACTAATTTTTGATAAGAAAAAAATTGGATTATAGCAAATAACAAAAAAAAAATGAAAATGAGAACTGTGAGTTAGATCTTGTCAGTTCTTCAATTATTTTTTTTGCATTTTTAACACATATTGAAATTGATTAGAATAAAAGAATTCATCAAAAACAAAAGTCAAGTTCAGAATCAGGTGAAAATCTACCTTCTTTTTTGTTTGGTCAAATGGTGAAAATCTACCTAAATCTATATATTTAGCTCTACAGAATAATTTTATGAAATTAGGGGTCCTAAAAATTATACATTTTTTGGGGGCCTAAGGCAGAAACCTTTTTCAAACAATGGTAAGCACGCCTCTGCCTCGAAGATCCGGGGATATCGTCCTGTATCCCAAGGACAAAAGCGCAGTCATCTGGTGCTGCCAGGTGTACCCGAGCTCTGGGAATCCGTGGATGAAGAGGATGATCTGATCTCTTCCAGATCCGGATGCTGGAATCTCAGCCACGTGCATGCTGGAATCCGGATGCTGGAATCCGGTTTTGTGTTCTACGGCGTCTGA

>TCONS_00068211

GTTCTACATGTTTTGGGAGTTAAGTGAAGCACAGTTCACACCAAGATAAGCTCGGGGACAGCAAGACTTTGTTACTTGCGAGCATGCCGCAACCGGCGCAATCTTCACAAGAACAAAAACGAGATGGAAATGGTGAGACATTGCCGACAAGGAAGCTCGTGCCCAGTAATGGAAGAGGCTTTGATGGAGGATTCCGAGATGGCTTCTATCTACGTCCAGTACTCGGGACGGTCATCACTTGCTTGGCACGAGCGATAGAATAGGAGGAGAGTTCCTCCATGACGAGGAGAAGCATCAATTGGTCATGATCATGCC

>TCONS_00050015

GGATCTTCAAGTGGTGGGACTCTGTGTGACCAGGAGTGGCCCGTGGTTTATCACGGTTTGAAGTAGCATTGCTTGTATTTTTATTGTTGTGCTCCTCGCTTCGAAGTTTGGTGGGGAACTTTTTCTAGTTCCAGTATCTTTGTTGTTTCAGCTCTTTTCAGAGAGTTGGGTTCGGTGGTGCGTCTGGCGCCAGATCTGAGATCGGGGATCTCTTGACGGTGCGGTGCAGCCGCTTGGTGCGGGGCAGGCGCTTTCGCTGACGACATTACTGTACTTTCTTCAATTCTAGTTTTGAGTCTGTTCTTCTTTGTGCAAGATTTGAGTTGTGTTTGGGTTCTTCGTCAAGAGACCAGGAGTTCTCCTTTTCCCATCTCCAATTCGTCTCCACCTTGCTTTCGTCGTCGTTTTGGCTTCCATGGTGTAGTCCTGCTGCCTTGATACTGTCTGGATTATGGGTTTGGACTTTAAGGCCATTATGCAGCAACGGGTGAAGATAATTGTTTGGATTTTCTTGTTGGTTGATTCCTTATAATTGTGGAGTTTGCTAGGTGGGCAAATGTCTTCGTGGGAAAAGGTTTATCCTAGTGTGATTTTATCTTCTCAGAACATGGTTTCTATACGGGCTCTTCTATGGTGTTTTGAAGCCGTTCACTTATATCTGGCGGTTAATCTTGCTTGTTTCAGCTTTGATATTAGTTTGGTTATTAGTTGCCTTGATTGGGCTGTTGTTTCCAGGGATATATAGTTGTTCCGCTGGCTTAGTTTCCGGGGATGTACGGTTGTTCCGCCGGCTGTGGTTCCCTTTCATGGGTTCTTGTAAAACGATGTTTTACGACTAATATAATTTTAGACGGAAAAAAAAAATGTCCACTGCAGGTGGACCAAATATATCTCCATCTCGCATTTTTATGCGTTTCAACCAAGGCAAGTTGCCGCAGCTGTTCCATCTCGAAATCATTGCTTTCACTCATTTTTAACACATCCC

>TCONS_00078462

CTCTCTACTGCTTGTTTACGAGTTGTTGAGTGTTCTTGTTGGTGTGTAATATCCAACAGCCCAAGAGTGTCTATCTCGGTGTGTCATATCCGATCTAGTCACTTAGGAGTATCAAGGAGCCTTTCCGCAGCCTCTTGTGTCACCAATCGATCCACAACCTTGTAGAGCTTGAGTCTTTGATTCGTTCTAGCAAGGATCTATCCCATACTATCCAGAGAAGCAATCTAGGGACGTATTAATAAATCACATAGGAGTTGGCGTGAAGAAGTTATCCCACTTTCAAATCAGGTGATGCCAGTTTCCCAGTTTGGGAATAGCACAGCTTCTTCGTCGTTCCAATCAAAACAGGATGAATCACTTTGTAAGAAGCTTGATTTGGATACATAAAATGGTGGAGAATCACCAGGAAGTTGAATAAATCACATAGGAGTTGGCGAAAAGAAGTTATCCCACTTTCAAATCAGGTGATTCCAGTTTCCCAATTTGGGAATAGGACAGCTTGTTCGTCGTTCCAATCAAACCAGGATGAATCACTTTCTAAGAAGCTTGATTTGGATACATAAAATGGTGGAGAAACACCAGGAAGTTGAATAAATCTCATAAGAGTTGGGATGAAGAAGTTATCCCAC

>TCONS_00046217

GTCTATACACACATCTATCATTTCCAAAGGTAAACCAAGGCTATGAACCAATTTCTGAATCTCATAATAAGATTCAGCACACTGTTTATCTTCATGCAAATACTCTTTAAACAACTCAGCCCATGCATCCATACAAACTTCAGGTAAGTTATGATCAGTTTTGATATTCATCATCCTAGCTGCCAATGATAATTTAGAAAGACCTTCTCTACAACCTTCATAAATTGGCTCATTAGCAGCTGCTAGCATTTCATAAAAACTTTTTGCATCCAAATTAGGCCCCTCTACATTTTCTACCTCCTCTATCACTGATCTTGTTTCACGAAATGCATCTGTAACCATATCATGCATCCTATCATGATCTACCATATGATCCTCTTGAAGGTTACAACTTTCAGAAGGTAAATGTGGTT

>TCONS_00070167

ATTTGCCAATGACCACAATTACGCTGATTAAGTGATTATACAACCAAGGCAAGACAGCTTTTTAAAAACCACATTAGGTAGATTGGAAGTTAATTTATATGCTTTTTACAAAAAAAAATCAATTAGGTGAACATAAGATTGTAGACCCTATGGTTTTGGAACATGTTTGGAGCGGAGATTATTGAAAGACAATTTCCAATGGTGATAACAAACATGTTACCAGTTTGGCAGTTTCATTAACCGTGGTGGTTTTTTGAACTTTGGATTACACAACTTAATTCAAAAATATTGGGCTTTAGAGAGTCAAATATCAGGACTTACAAATGAAATGGGATTACCCAACATGACAACGTTACGTGAATTATGTATGTTTCGTGGTGATTAGTTTTTGGGTCTAAAATTTTTGGAATTACACCATAGTTATAAAAGGAAAAAAACATGACAACGTCAAAATCAGACAAAAGAAGATGGCTGAAACATATTCAGAAAACTAAACTAGTAAACGAAGAACATACTTAAAGGGCTTAATTTATCTATCTCTTCCCTCATGCATTTTGTTGGCTAAGATTTGGATTACGCTTATGTTGTTGTTGTGAACTTTGAATCGATGTAGAACGGAACTGCTGAGGAGGTGAAGAAGATTGTCAACACTCTTAACGAAGCTCAAGTACCTTCCCAGGATGTCGTTGAGGTTGTGGTTAGCCCTCCATATGTGTTTCTTCCTCTTGTTTAGAGCACATTAAGGTCTGACTTCTTTGTCGCGGCTCAAAACTGCTGGGTTAAGAAAGGAGGTGCCTGCTGGGTTAAGAAAGGAGGTGCCTTCACTGGTGAAGTCAGGTGAGATTCCCTTGCAGCTGAGTTCTGTTTGATTTATTTTGTGTTAGAAACAAATTAGTTTCGTTTTGTGTTCTCTTCTTTGTCCTGTTTCATATTATATCCTGCCTCTTATGCCTTACGTAATTTGTGTGTGCAGTGCGTAGATGCTTGTGAACTTGGACATCCCATGGGTTATCCTTGGTCACTCTGAAAGGAGGGCACTCCTCAATGAATCAAACGAGGTTGGTGACCTTTTAATATTGAAAAAATTGCTTTCACTATACGGATTTGAGATTCATGGTTTTGATATTTTACCTCAATCCTACGTTTGTAGTTTGTTGGAGACAAGGTTGCCTATGCACTTTCTCAAGGTTTGAAAGTGATCGCTTGTGTTGTGGTGAGACTCTTGAGCAGCAAGAGGCTGGGTCAACCATGGATGTTGTGGCTGCCCAGACTAAAGCTATAGCTGGTATATGTATATCCATTATATTGTCAAACACTTGTCATTGTTTATCTCCAACCTGCATAGAAATGTTTTGCTGGCCGTTCTTCTAACAAAGAACTTTTTTGTTGGCAATGGCAGATCGCGTGTCGAACTGGTCAAATGTTGTCATAGCCTATGAACCAGTGTGGGCCATTGGAACCGGAAAGGTCGCTAGCCCAGCCCAAACTCAGGAAGTAAGTTGTTTTCTATGCGTGTGAGAGCTAAGCATTGTTGCTTATCGCCATGACTATATATAACATGAACACTTATATTCCTTCTCATCTATGAACACAGGTATGATGAGCTTAGGAAATGGCTTGCAAAGAACGTGAGTGATGATGTGGCTGCTACAACCCACATCATATACCGAGAGGTAAACAATAAATAACGTCATATGACTATATTTTGATTTAAATCAAATTCCAACACAAAGTTTCTTTGATTCTCAGCTTGATTATGTATATAAATTATCAGGATCCGCTCAATGGTGGTAACTTCAAGGAGTTAGGTGGTCAGGCCGATGTTGATGGTTTCTTGGTCGGTGGTGCTTCTCTAAAGTTACATTTATATTGTGTTTTGTCTTTTGTTTGTTTCAGTCAAACCCATTAA

>TCONS_00074322

TTTTTTTCTGGCGGCGGTGCTCTTACCTGTGCTGTGAGAAGTCTTCTATGAGTCCTCTTTACTTTTAATCGACTTCTGTAGTTTTAAGGTCTTCGGATGTACTGGAAGGTGATGGAACTTCTCTCAATGGTGATATGGTGTGGTTGAGCGACGGTTGGTTTCTGCCTTGTTACCAGTGAGGTCGAGTTCGTCGTCAGGGCGTGATCTATCCGCCTGTGGACGATGGTGAATCTAACTGTCCGGTGGGGTTTGAGTCCGGCTCGGTGGTTCCACGTCCAGCGGGGCCTCTGTTTTCATCACTTAGTTGAATTTGTCTTTGATTGGCATATCCTGCATCAGTGTTGAAGATCTGGGTGCAGGGACCAGTCGCTTGTTGCAGTTCTAGTCCTTTCTCAGATTGGGTTTCTTAGTTTTATGTGTTCTGCAAATTTAGTATATTTGCATTAACAGTTTCAGTTATTTTAGTCATGATAGTAACTCACTTTGATCAAAAAAAAGATGGTAACTCACTTATTCATTATGTGTTCTACAATTCTAATGTAGAAACAGTTTTCCTTAATCGACTTATAAGGTACACTAATTGTGGTTCTTGTGCTAATATCTTGTTCTTTCTCAGTTCACTTCTGGCTTTTCATATTTTTGGTTTCTTTTAGAAAAAGAATGTTGTCGGCATTGCTAAGTTAGATGTCGTCTCCTAAAATGTGGAAGAAAAGTGAGAATCAGTTCAGGGTGCAAAGACAGAAGAACATACCGCAAGATCTGAACCTCGGAGACTGTGGGTTTATACACTTTGAAGTATATTGACTGTTTAACGGTTTGAACGACGATATTATTCCAGTTGTACATCTGAAGATGGCTGTAGAAATCTATGAGGAGGTTCCTTAAGTTTGCTAAGTAGTATGCATGTTGTTTTAAAAACTGCTTATGTAGTTTTTTTGTTGTTTTTTTTGTATTGGATGTACAAGTATTCCAGTACGCGAGCTGATGTTCTTTAGTCGGATTATTGTATATGCCAACCGGGAGTGTTGTTACTCCCGCTCTTATAATTTCATCAAGAATCTCACACAGATTTTTTTATTTACTTTGACCCTAGTAACACTATTTATAGAAGCATATTTGTAGTGACAAAATAATCGTGGACTAAGTTAAATCCAGACCTTATGCTACAGGTGATGAAAGAAACTTGGACAATGATACGTGAGAC

>TCONS_00043199

CGAGAATCTTTTATTTTATATAATGGCCCCGTTGTTTTTTATTTTTTCAATTTTGAAGTTGAGAAGTTTTGTAACTTTGGGATTTTAGTGACGGATATACATTGAATGTTTAAGAAATGGGATTGGAGAAACGGAAGAGAAAAGAACATTAGTTATGTTCAAGTCCTAATGAAATAAGACTATTTACAAATCCATTGGACGTGTGTTCAAGAGCAGGCGCATGACAATCCATCGATCTTCAACGGGATAAAAGTTCGCGAGCTCTTCTTTTTGTCTACTCCCGGATGAACTTATGTAGTTCGAGTTGGTCCTATTTAATGTGCTTGTTGAAATGTTACTTTTCTTAATTAAAAAAGGATATGAGAGTAGGTTTAGATCATTTATCAAGCCTCTAGAAAATTGTTACTTGCACTCACAATCTCCAAAATAAACAGATCCAAGAAAAATAAGATAAATCTCTGTCTGTGTAGATAAATAAAAAGGGAGTTGATCCAAAACATGTTCTACAAACGATTGTTCCAACTTAAACGTAAAAAAACACAAAACATAAAAGGTTCAGAGAGTTATGGATAGGCATGGAGACACAGAGACTACTTGGATCGCTGTCTCATCTTCCTGCGCTTCCTCTTCAGTCTCCTCATCCGCTTCTTCTTCCACTACAAACAAAAGATACGATCAAACCACAACAATGACATCACTCTAACCAGTTAATAACAAGAATCAATAACTGTAAGCCAAATGCAAAATCAATTAGCAATTTAACTCATAAAACGAACGATACTCAGCATCCATTAAAGTTCTGCTAAATGTAAGAGAATCAATCTACAATACGTAATACTAACAAGGAAACCTATAGAGAGCCTTGTCGGAGGTTACAGAGGTAATTTTCGTACCTTCGCCCTCATCGTCAAGTCGCCGAACTCAGATTCTCCTCCGCTGTGTTGTTAGTCGAGCGCACCACTCCATATTATATAGCTAGGGTTTGGGCGATAAGGGCCATGTTTTTTTAAACGGGCCGAACATTTTCGAAGGCCCATCTAAGGATTTGAAATTAATTATAAGAAGAACAAGAAAGTACCTGTACAAATGTACATAGAGAGAGATATGAGAAGAAGACAAAGCAAAGTACTGTTTCTTCTTCTTATACATAAGTTCCAAATTTGACTTTAACAGTAATAAACTCATATCCATCCTCATTCCCATTGCAATCACATACACATTTTTGTCACTTCTTTTTATTATCCCTCTTTTTTTTTTCTGCTTCTCACTGTACAAATAATTAAACAATAAATATTTATTATTGAATTTGGCATCTGGTTCACTGCGGCAAACAGCTAATAACC

>TCONS_00005677

TCCGAGTGCATAAATCCCTAAAAAATGCTCCAACTCCTACAAATTCATCATCAAACGAAAATATTATTTCAATTAACAAAGACAATAAATTTATATATAATTTTAAAAGTCACTTTTATTACCGGCTATTGCTTCATGAACATGTTTCGGTAATAACTCCGTCATTGCGAACGGTAGGAGCCGCTGCATGAAAACATGACAGTCGTGACTCTTCATACCAGAAAATTTTTTTCCTTGCTCGATGCATCTTGAGAATTTTGACACATATCCATCTGGAAACTTTACATCAGAATTTACCCAGTCAAACAACTTTTGCTTCGCCACCCCAGACAATCTAAAATTCGGAACTGGTAATTTTCCTTCTCTTGTCACATGCAGCTCTGGTCTTGCACATAACTCTGGCAAATCCAACCTCGATTTCAAATTATCTTTACTCTTTCCCTTCACATCCAAAAGAGTGTTCATGATATTCTCAAAAACATTCTTCTCTATGTGCATCACATCAAGGTTGTGCCTTAAGAGATGATCCTTCCAATATGGAAGTTCCCAAAATATGCTCTTCTTATGCCAATTATGAGATGCCGTATACCCGTCTGGCATGTTAGGTGGAGTGTGCCAATTTCCTCCAACTTTACATGTCTCCTTAGCACCGTAATAATCAATCTGCTCTAACAAAGTTGCTCCTGGTTGCATTACTGGGGGAGGAACATGCACTACCTTGTTTTTCCTAAACAATTTCTTGTTCTTACGATATGTATGATGCGGCGGAAGAAATCTACGGTGACAATCAAACCAACATGACTTCCTCCCATTTTTCAGCTGAAAAGCATCTGTCCTATCCATACAATATGGACATGATAATCTTCCATGTGTGGTCCAACCCGATAACATTCCATATGCGGGGAAGTCACTAATGGTCCACATAAGCACTGCACGCATATTAAAGTTTTGTTGCTGCGAGTAATCCCATGTCTGCACGCCATGATGCCACAACATCTTCAGCTCATGGATCAAAGGTTGCAAAAAAACATCAAGTGCTCGCTTTGGATGTTTTGGACCAGGCACTAGAATACTCAAGAACAAAAATTCTCTTTGCATGCACATCTCCGGAGGGAGGTTGTATGGCGTCAAGATGACAGGCCATAAAGAGTATTGCCTTCCAGACATACCAAATGGACTAAATCCATCCGTGCATAAGCCAAGATAAACGTTCCTAAACTCATTCGCAAAGTCGGAATATACGGTTTGAAAGTGTTTCCATGCCTTTGCATCTGAGGGATGTGTAATCTCGCCATCTTTCACCGAATGTTGGGCATGCCATCTCATCGACGCGGCTGTCCTTTCTGAGTGGTACAAACGCTTCAATCTATCTGTGATCGGTAAGTACCACATACGTTTGTACGGCACAGGATTCCTTCCTGTGGTTTCTTGATATCTTGGTTTTTGACAAAATCGACATTCCGTCAGTTTTTCATCATCTTTCCAGTAAATCATGCAGTTGTCAATGCATACATCAATCATTTCAGATGGTAGACCAAGACCGGCAACTAGCTTCTGAATTTCATAGAAATTATCAGCAGCAAGATTACCTTCTGGTAAATACTCATTAATGAGTTGAGCCCACGAATCCATGCACTTCTCATTTAGATTATGATCACTTTTAATAGTCATCATCCTAGATGCTAGTGATAATCTAGAAAGCCCTTCTCTACATCCTTCGTAGATGGGTTCATTGGCAGCGTCTAACATATTATAAAATCGTTGTGCGTCTATGTTAGGTTCTTCCCTACTACTATCAGGAATAGCAGCTTCATGCAATGCATCTGTAACCATATCATGATACCTATTTCCCTGTTGCTCATACCCATCCCCCGCATTGTTTCCATACATATTTTGCTCATACCCATCCCCCGCATTATTTCCAAACATATTTTGCTCATACCCATCACCCGCATTATTTCCAAACATATTTGGCTCACGAGGATCATAATAATCTGCATTATAATTTGGCAGTTCAAATCCCTGACCCGTACTACTACTTGATGCACCTTCCCCATGCAAAAACCATATGTAGTAATTTGGTCTAAATCCTCTATTATATAAATGCCTCTTCACAGTATCTATTTCTAAATATGGCTCGTTCTTGCATTTTCTACAGGGACAAAAGATTTTACCATGCTCCCTTGCGAATGGTTGATTAGTTGCCTGTTGTAAAAATCCTCGTAGTCCAGCACGATACTCTTTCGTTACTTCACCAGTCTCCGGATCCTTATGATTGTACATCCATGCCCGTAACGCGTATATTTCATGTCCGGACGCCATTTTCTCTCAACTTTTTATTTTTTTCTTACTCACAACTCACGGCAAA

>TCONS_00047236

GATTTATACAATATATAACAATAAAATGTAAATTATATCTTTGAAAGTTTTGCGATGGTATCTGTTATATATTTATAAAATGAAAATCTACTCGCACGGATGTGCGGGTGAAAAATCTAGTTAATAATTGTAATAAGAGTTCCATGAAATAAATAATTCAATCCCGTGAAAAAATATTATGGCAATTTCGGCTTTCTCTCTCCTCTCTGTTCTCTCCTTCATACTTTTCACAGAGACGGATGATTGAGAAAGCGAAGATGAATGGTCTCTCTCTCTTTTTTCCTTTATCCTTCAGAAAAAGAGAGGCTCCGTCAAGCTGGTCGGATTTGTTACCGGCGCTTGACTCCCGATGGTCGGGCTATGACTCTGGCAAGTGGTGGCTCTTAAAGCACTATCTTGGCCGGCTTCCGTCTCCGGCAAGCGGTGGTTCCCATTAACACCGTATTTTCCGGCTTTTATTTTGAGATTCATCTCTCATCTCCGATGGATTTCAAATCTGTTTTATACAGTTTTCTTCTTGTGTTTTTTTAATCTTTCTTTGTTTCTGTTCATCATTTCTTAGTTTCCTTCGATTCGATTTTGCTAAGGGTGGTTTTTGATTACTGTGATCTTTTCCCATTCATTTGGGTGGCCCCATCTTCAAGCTAAACTTTATCTTTAAAGATGGTTTTTAAAAATTGTTAGATCTTCAGTTTTCTTTGTTCTAAATGGACCTTAGTTAGATCGATTTGATTTCTTTGAAACGGTGTAGTGGTTTATGGCTACTGATTTCGAGCTTCTCAGATGCTTAGCTGTTCTATTCCTGAGCTTCCTGTTCCGGTACGTTCCGGTGGATCTCCGGTTAGAAATTCCGGCTTCGACTTGAAAATCGGTGAAGAAAGGGTTGTCTCTGCTCTTCCGCCACGCGTCCATCTACCGCACGCGTCTGATGCGTTGGCTGTGAGCTGTCTCGTTGACTGCTGAGCCTCTTTCTTTCTATGGGCCTTTATGTTAGTTGGGCTTGCGGTTTGCCCTAGTTGTTAGTTAGTTAGTTTGGCCTTTCTGTTTGGGCCTTTATCTTTTATATTCTTGTACTTTGTTGGCCTTTTGGCTTTTAATAAAACGAGATGGCAAAAAAA

>TCONS_00019131

AGGCTAAGATTACAAGCATACAAACCTAAGATTAATACATTCGGTCCATTACGAAGCCCGAACACAGAAGATTAGGATTCCCGCATGTTCGACACGTTGTGCTTGCGTGGCCTCGCCTTGCGCCGCCGGAGCTCATCACCGGACGAGGAACCATCAAACTCCCTTCTCGCCTCGTTTGTAGTGCTTGCACGCCGCTCCTTCTTCTAATCGCCATTGATTCACTAAGAATTCTTCATCACTTACTAGAGATTTATCCAAGAAAAGCTTCAGAGAGAATCGAACACAACACCTTGCCGGAACACTTTCCTAAACGGGAAGCTCCGCCTCATCTGGAACTGCTTCAAAACGTCCGACCTTATACACATCTTTGGTCTTGATTTTTTGGGAAACTCCACTTGAGCTGAGGCGAAGATTGATTCCTATCCAGAATCAAGAACAAAGCTTTCTAACAAAGTGAGATAAAATCTTCAGAGATCTTACGGAATACAAATGAAATCAAAGAAGGAAAAGCTTTAGAACACATCAAAAGTCGCCTAACGAACCAAATTGATCGCCAAACGAAAACTATTCTG

>TCONS_00043514

GTCGTTGATGGCAATATCTAAGCTGCACAGAGAAGGTTACAAAAGTTTAGGATGGTTAACCGGAGGGTTTAACAGAGCAACCGAAGGAGATTTTCCAGAGATTGAAGGACCAGAAGAGCTCCGGTTTGCAACTATAGGTGGCGCATCATACTACTTCCTCAAACTACTTGTATTGTTGCCGAATTTTGGCAAAGAGAGCCGTTGAATACAATTGGCAAAATATCATAAATGTAAATCTTCCTCAGCAAGAAAAATATCCAAAAACATAAAAGTTAATTAAATTTCATTTAGAAAAGAAAAGTTAGAGATGACGACGACACTTCTTCTTCCTCAACCAAACCGTATCTTCAAGCCACTACTAGTACCAAACCAACGCCTCCGAAAACCGTACAGATTACCAGTGATCTCCGCCGTATCCGGTCAGCAGTTGGTAACTTCCGGAGAGGTCCGGGCGGTGGAGCCGAGAGAAGCAAAAACGGTGTTATCGTCGGAGGGATACGTGCTATTAGACGTGAGACCGTCGTGGGAAAGAGAAAAGGCACATGTGAAAGGATCCTTACACGTGCCACTGTTCGTGGAGGATCCAGACAACGGGCCGATCACGCTGCTGAAGAAATGGATCCATTTGGGATACATTGGGCTTTGGACGGGCCAGAGATTCACCATGTTTAATGACGAGTTTACCCTCCAAGTTGTAGAGGCTTTCCCGGACAAGAAGAGTAAAGTGCTCGTCGCGTGCGGTGAAGGACTCAG

>TCONS_00072414

AACCACTGTGTTCAGTCACTCTCAAACAGTTGTAGTGTGTGGAAACTGTCAGACGGTTCTGTGCCAGCCCACCGGTGGTAAAGCTAGGCTCACTGAGGGTTGCTCTTTCAGGAAAAAGTGAGAGGATGAGAGATTTCACACAAAAGTTGTAGTCTTTTTCACAAATGTTGATGTGAGAGATGAGAGATCAGATCTTTTATTCAGTTTGGTTTTTTCTTGCTTAGTAGTTTTTCATGAACTTATGTTTTGCTTATGAGTAGATTGTTAAGGTATCATCAAACTACTAGTTGAGTTATTCATCCTCTGCGTTGTTCTTTGTCTTCAGTTTAAACAGCCGGACGTCAAGTGCCAGGGATGCTTCAACATGTTCTCCAAAACGACATCGATCTGCTGAACCCTCCCGCTGAGCTCGAGAAGAGAAAGCACAAGCTCAAGCGTCTCGTTCAATCTCCCAACTCCTTCTTCATGTGGAGAGTGAATTAGGCAGTTGCATCCTCCCTCTAGGTTTTCACCAATTCGTTTTCGTTGCTGCATTCCCTCAAACAAAACAATCATCATG

>TCONS_00059078

TTGGATAACTCGCCGCCGAAATCTGAAGGGTGGAACAGTTGATGCAGACGCTGAGGGAAGAGACTAGCATGGATATTGTTGTTGCGTGTACAGACGCAATCCCATAAACGGCATGCTGTGTGTGTCATGCAAAAGTGGTCAAAGTATTAGCTCAAATTGAGATATGGTGTTGACCAAAAGGTTAAAGATTGGTCCTCGGTTCCATTAGATAAGGGTGTTAGATATAAAATATATTGATATATTATTATTGTGGGTGCTAATCTTTCATTTTATTGTACATAATATACTTCTTGCAAACGAATCACTTGCTTAAGTTCCAACAAGAAGTAGTAATAACATAGTATCAGCAAATGGAGTTTTTCCATAACGCTAAAGCCGTTAGGATGCGTAACGTCCACGAGAAGTATCTTATGGCGGACGAGGACGAGGAGACGGTGGCTCAAGAAAGGAATGGTTCCGACAAGAGAGCTCGATGGATCGTCGAACCAGTTCGTGGTTCCTTTGAAGTGATCCGTTTGAGGAGTTGCTACGGTAGCTATCTCACCGCATCGAAAGAGCGGTTCTTGCTCGGTGCCACGGGGCGTAAAGTGGTTTTGTCGAAACCGAGTGGACTTGACTCGTCCGTAGAGTGGGAGCCGGTGAAAGAAGGGTCAAAAGTAAAGCTCAAGACTAGACACGGTCGCTTCCTCCGAGGCAATGGTGGGCTTCCTCCGTGGCGTAACTCAGTCACTCATGGCTCTCATCATAGTTCGGACTCGTTCTTGTGGGATGTTGATGTCGTGGAGATCTTGGTTGAAATGACGTCTCCGGCTCCAGCTCCGATATTACTACTCCGCAACATATGAGGCTGTCGAAGACCTCCGATGTCTAAGACCACTTCAGGAAAATCGGAGGAAGGG

>TCONS_00038334

CACGAGTCACGACATCGACAGAAAACTCAGGTGGAGCAATCTCCGATCAAGAGTTACCGTCGTCCTATCTCACTCCATCAACATCTCCTCTGTCACAGCCGCCGTCGATCTCGACATTTCCTCCTCAAGTAGCCATCGTGTCTCTTTCAAGTATGGCTTCTTCCTCGGATATGAGAAAAGATTCTCTTCGTCTGTATGAGGTTGGAAAAACCCAATTCAGAATATGAGCATGAACCATAGTTTTTTTCTGTCCAACATTCAAATGGTGGACGAAGCTGTTGGAGAAGATGTTTTATCTGAGTTGAGGGAATCAGCTCTACTATAGGGCATCCATAGTCTTTTCTCGTACTCTGTTCGCAGTTTCTACATTGTATGCTTCATCTGAAGGCTTTTTGTGCTTCTAATCTTCAGATATGATTGTACAAATGGCTTATCATTGGTTTTTATCTTTTCACAATGCTCTGATTCTGAGCATCTGTCACTGAGAAAAGTGAGTTCCACGAAGCGAAAAGTTTAAGAGATATTTCACAAATTATATTTAGGACGTGTCCTGGAACAAAGTCTAGTGAAACACTTC

>TCONS_00009877

TTGGAGGAAAGTGATGCAGCCCTTAATCAATCACTATGGGGAGCTTCTTCTTCCGCAGAACATCATCAACAACAGCAACAACAAGGGATGAGCTCTTATCAAGCAAACATTCCGAGTTAAGAAGCAGGTTTCTTCAGGCCTTTACATGGGACGTTTGTTTGTTTTTATGGAAAGATCATTACAATCATTTGTATGACAAATGCAAGCAACTCTGCAACATCACATAATATTATTAACGGGTTCTTCCGTGGATGGATGGTCTGAAAAAATTCATAGCAATCTATACGTATGTGTTCTATAGATAACATATCATGGTTGTGCCATTTGGTTTTTCCAAGAGAACAATAGTTCATGACTACAAAACACTGATATATAACTAAGGCTGTAATATCATGGTTGTGCCATTTGGTTTTTCCAAGAGAACAGTAGTTCACGATTACAAAACGCTGATATATAACTAAGGCTGTAATATCACACTCCAGATTCTTTCTTTTAGCTCCTAAAATATATAAATTTTCTGAACTTTTTAAAATAAAAATTTTTGTGGGAGAATTCCTTTAATTATTATAAAATATTCAGAACGGTACTGATTCATTACACATCTTAAAGTAAATGCTTACTCCAAATCTCAAGCTACTTAATTCTTCTGTCTTTAGGCATTTGCTAGGTGACCCGGTATGGGAGTGGATGAGCTTGAGCAGCTAGAACTTTAAGTAGATACATCACTAAGGCAAAAATGATCCACAAAGTTTTTTTTGGTTTCAGATTTACCACTAATCTTTCCATCTATTGATGATTCTCTTAAGTCTTCGCGAATCTCATAAAAACTTACCTTTCCCACAATCCTCTTTGGAAACATCTTCAGCTTCCATGATTGGAAGGGCTTGCCTTTGATGATTTCTGTTTTAAAGCCATGGTTGGAAGATCTACAGGTATTGCTTATTTCTTTCTCTTTGGGTTCTGTTTGATTTCAGACATAAATAACTTACTCCTGGATTTCCTTGAGATTGATGCGATTTTAGGTTTACCGGACAAGCCATGTGAAGAAGAGGATGGAATCAGATTTCTGAGTTCATGAAAATCTGACCGAAGATGCATTAGATTGAATCAATGATATTGTTTCCACCATTTGTGAAAGTCGCGACTCTTGCGTAAGAATACTTGTCTTCTCAAAAAATTATCCAAATTATGTTGTAGATCTCCAATAGTTCGACTTGGATCCAATGGTACTGTGCAGCATGTGAAACCAATTGAAGTCCTATCTCTGGTATTTTTCCATATATATAGATGATACTCATTGGTCTTATATGATTGGAGAGTTTGATGATAACACATGAGAAGTACTCTTTCTTGATTTTTTTTCCTGGCTTTGTAGCAGGTCTGACATCTTTTGCACGCATCATCGAAAGAGCCATGACGATTGTGGAAGAAAGTGGAGGCCGGTACCATGTTCTTCTCATAATCGCTGATGGACAGATAACAAAAGACGTGTTAGAACTGGAACTTGAGTTCAGTTATAAAT

>TCONS_00056558

ATTTATGCCTTCTTCAGACATTTTTCAGTATCATGTTCTTCTGGAACCTTTCCACTTATGCCTTCTTTCAGACATCTTATAATATCAGGTTCTTCTGGAACCTTACCATTTAAGTGTTCTTTCAGACATCTTTTAGCATCAGGTTCTCATTGAACCTTTCCACTTATGTCTTCTTTCAGACATTTTTTACTATCAAGTTCATCTGGAACTTTGCAAATTTTCTCAACCAATTTCTTTTGGGGTTTTTATCTCTAGAACCCGCTAGTCTCCCCCTCTTTAACTGAACCCTAGGTTCATTTTATTTCCAACAGTTTGTTCTTTAGGAACATCAACTTTTGATGGAACACTTTCAGCGGGTATATCATATCGTATCTGTGAACACATTTGGTAACTGGTTTGCCAAATTATGCAAATGCACAATTTTCTGAATTTCCAGCTCTCTTTGATCCGTAGGGGGACCAAGGTGTAACAACGACTATGTACACCATGTAATTTCTTTAGGAATTTCTCAAGTTCCTCCTCCTAACGCTGGAAATTCACCTTCATTAAAATGACAATTGACAAATCGTGCCGTAAACATATCACCAGTTACTGGTTCAAGATACCTTTTATACGGCTTGTTCTAACTCTTCCAGAGTTTTATACATTCCCAAG

>TCONS_00069089

CTGGCATCCAGTCGGCGCATGTGCGTATGTGCCGGCGCCGGTAAAATCCTCCTTCTCCATATTTCTTTTGCTTTGCTCATCTGTCGTCTTTCTCCAGCCTTCGTCTTGTTCTCGTCTCTGGCTTTGGCTTTCTCTCTGGTAAGGTCCTCTACTCGCGAAGATGATTCAATCCGGCCGCGATGTGCTTGTTCTAGAGTCTAGGCGCAGAGGTTGGTCGTGGTTTTGTCTGTGGAGATCAGCTTTAAGGGAGGTTTTCAATTTTTGGATCTGTCTGGTGGTCTTCTATTGCTTTCTTGGGGTTTGATGGACAGTCTTCTCGGATGGTTACCTCCGCCGGTTATGTAACTTCCTCGGAGGTGATAGTGGGTGGCGGTGGTTGGTTTCCCGGACCTCTACGGCGCCGTGGTGATGCGCTTCCTATTCCGCTTAAGTCAGGTGAGCTTCAATGGTCTTTTTGACACGGAGATAAGCTGTGGACCGTTGTATGCAGTCGAGTATGGTACACAGTTCTGTTTCTGGCTCGTCAAGGGTTATGGAGGTCGTTGTTGGGAGGTCGTCTAGAACTTTCTTGTAGCAGCTCAGTGGATCTTCTCATCTCAGATTCTCCAATATTGTCACTGCCTTGTTCTTTCGCTAGATGGCGACGGCTGTCTCTCCTGGAGGTTCGTTCCTTGGGTGTTATTGTTAGGTTGCTATCCTTGTAACACAGGTGGATTTCATTGCGTGTCATGGGTTCCTGATCTTTTGTTTCTCAAAATTGGCAAAAGGTGTTGCGATAGTAGTCTTGGTTGTGGCGGTGTCCCTTTTGAGTCCAGTGCACATGCCAGTGCTGGTTGTTGATCATTTGGTTTACGATTGGTAGTGTTTCTTGACAGCTTATGTGCAGGTCGTTAGAGTCTAAAGGAAGTGTTTGGTGGCGAATTCAGGTCCGTCTTCGTGAAGAAGGTAAGGATGTGCAATCGGGCTTTTGGATGCACGGTTTGTGTTCTTTTGCCTTTCTCTCCTTCTGTTCTCTTAGAGATTTATTGACAGGGAAGCTTACGAGTTTAAACAAATACATCGAGAGCCAATTACTCTGGAGGTGACTCCGGAAGTCTCTACATCCGAGGCAAGGAGGTGAATCACACCTTCCCATGTCTCTCTCGTCAAAAGTTGAGAACTGCTCTCGATTCTCATTGCAA

>TCONS_00045825

GGGAAAGATGAGTAATCGGAGGTAGAGAATGAGCTTTGGATCGGGTTTGGATTCTCAGCTCCGGCGAAAACTTCTGATGACGATGAATAAGTCACTGGTCGGTGATGGTGAAGAGGAAGGGAGGTAGACGACGATATCGAGTTTCTTGACCGAGCTCGAATCGCTCCCATGACGGAGTCGTAATCCGACGGCGAATTCTCAGCCACGGGAGGCAAAATCTCCGATCTTGCGACCCGGTTACCCGTACCGGGTTCTTTATCCATACGGGCACGAACCACACCTTCGACGTAGGCGTAATCAGGAGGCCGTGGATGCTCCACTTTCTTCCGATTACACCAGCTGAGAATTGTTGATTTCATGGCGAGGTTAGGGATTACCGTTGACAAATCGGGTTGACTTCCGTCGTGGAGCTTCGGCGCGAACCCGAGATTCCGGCAAACTTGGACGGAGATACGCTCAAATGTCTGCCCCGATGCTACGACGACGGGGTCGGACATTAGAAATCCGGTGATTGGACAGAGGAATTCAGTCGGAGTTTCGCCGGGATTATTTTGTGGCATAGACGTGGCGGAAGCTGAGCGGTGGTGGTGGAAGGAGAACCACCTGTGTCTTCCGTTCTCTCCCATGGCAGTAATTATGAACAACAAGAAGAAACACAAAAGACGAATCTGGGAGATTGTTGTAAGAGATTTTTGACTTCAACTCCTGGGGTTGTCAGAGGTGAGATGAAATCGAGTTGTTG

>TCONS_00054718

TTTATTATACAAAGTTCCTCACCAGTTGATGAGCGGAATGTTGTTGTATATCTACGAGGAGTCCTAAAAAAAAACAAGTGCTCTTTCTTTTGAATCAAAGCCTCACACAAGAAGAAGAATGAGCTTTGCGTGCCTTGTGTGTCACAGTGTAGAGAGCCCGTCCCACTCCTTTAGAAGCTACTCCGTGTCTAGCTCAGACAACGAAGGAAGATGCTCTGTGATCGCGAGCTGCCTCACTAGGAAGTCACTTATTCAAGCTGCAAGGTCCAACACTTTCCCTGCATCATCCTCAAAGGTAACCCCACAACCCAATTTCCAGGCAGGCGATCTGATGATTACACAAGGAGCCTCGCCACGGTTAGTGCGAAGCCGTGCAGGGAGAAAAGACTGGAACTTCAACGAAATCGAGGCAGAGCTTTAAAAAACCAAATCTGACTCTATTCTCTTATAGACTAGTGTTAGTTTGTTCATCCTTCGGGTGATCTTAGCAGTCTATCTTTTGAGTTCTGTTCACACGATCTTACATTCATCACTTGTCAAATTTTGTACTCTTCTCACTACTTTATAAACCAATAAATCTATAAAATTATCTTTCACGTTTCCGACCGTTTAGTTTGGTCTAAATCATACGAGCCTGAGCTGGACATGTTAACAACATGTCATTTGTCA

>TCONS_00076899

GTTAACAGGACCTTGAATCATTGTCGAAGGTGATAGGTCAAAAACAAAAGGAATTTAACTTTCCAGTCTGAATTAAAAAGCAGTTAAAGGAGGAACAAAGAGTTATATGAAATTTTTCTCAAAAAAAGAGTTAAATGACACCGAAATTACCTGAGGAGCATGTCGACACCCATGAGACACTTAAGCCATGAGTTTGCCGCATTTCTTAAAGTTTCAGACTTCCCAGAATCTAAACAATCGGACTCTGACGGTGTTGGAATGGCTTCCAGCTTTGAAGTTGGCGAGTAAAATCGTTGGGACAGCCATTCCTGGTGCGAGATTTTCTAAGGATTCCAATGATGCCAAAAGGAGAATCTGGTGAATCTAGCAGACCGAGAACCCTTATTTATAGGTCTGGGAGACGTGGGTCAGTGGTGTTAAAAGCTAGCCTAAATGAGTATTTTTGAATGCAACAAACGACGGTATCGGAGCGAGGGGATGGAACATCGATCGAGACAATGCAGAGTCAGATTTGTCGAGAAAAATACTTCTTGTTCTTCAGAAGGGACGGCTTCGGTTTAGTAGAATAGAAGAGTACAC

>TCONS_00022643

GAAAAACTCAAACTCTGAGGAGACAGTGAGGCTGTGATCTAGATCTCTGACAAGCTTCAGAACCAATGGACTGTTGTTGATGTAATCTGTAATTACATTGACAGCGTCAACTATGTATCCAAGTGAATCTAAGTCCCCAATCTTCAACAGCATAAGCTCAAAGCAGTGAGACACACTCACAGACCAGAACACTCCCTTCTTGTTACTAGCATAAGACTCGCCTAGTTCACCAACCCAACCAGAAGCTGAACATGCAACAATCTGAACCACGATTTGCACTCCCACTTCATCAACAAGCCCATCCACCAAAGATATCAAGGCCTTGCTGTCGTGTTTTATGTCAGAGACATCCAAAGATGTTAGATAGACAGGACCCGCTGGACAGTCTGCAATGAAAGTAACAAGATCACGGCCTTTACTGTCAACCCAAGCTTCCAACAAGATGCTGCAACCAGTGATGGCCCATGAGTCTTTGATCTTCTTCACATACTCTTCCACTTCTCCCAAAGCTTCTTGAAACATCCGTCCTTTTAAATCATTAGAATCAGGGATGGTACCATCACTTGCAGCTATCATCATCTCTCTAAAGCCTAAGGACTCCACAGCTGAAGGAGTATCACTTTCTCTGGATGAATCTTGGCTTCTCCCACGCTTATGAGCATTAACCAGTTGGACTTCCCCAACACTCCTACTCTTAGCAGATCTAAGATTATGTTTCTCCTTGGTAGCCACATCTCGAAACATCTCTCTAACAGTAGATGCAGCTTGTTCACATGGAGTCGCATCTGAACCAACACCACCCAAGTGACATTTCAGAAGTTGCAAGCCAGACATTTCTTCTCCACAGTGTTTGCATTTAACCCACTTCTTC

>TCONS_00042299

CGCCTGTGTCGTATAAATATGGTTCTTCTTCTAAAATAAATTGAAACAGCATAGCTGCTTGCTTCTATATATTTTCTTTCTGCTTAGCTTTACAATGAACAGTAGGAGTTCTTTTCATTGTTTTCTCTGGGGTTATATTGATTTTGTTTTTTGGATTTGTGGCGGGTAGGAAGAAGGGATATGGTGATGAAACGAATAATTTCGTGGACTAGAGTTTCCGATCTGGGCTCTTCACCCAGTAGTTGACGATACATCCCTTGGCTGTCTGAGTATCCCTCTTCTTTACACTCCCGCCTCCACCACAATCATATTTGCTTTTTTGACATTCTTTTTAGATTAGCCGATGAGGATAAAAACAATTATCTTAGCGGATTTCAGTGAGGTTTTAAATCAATGATCTGAATTGTTACACACACTGAGGCTGTTTTTATTTGATAAATAATTTGAATCGTTTCTCTGATTTATTGTTTTGATGCGATGATGATGAAACTGCACGGAGGTGCACGAAGGGCAGTGGACAAAACATAAACTGTTTTGCCGTGAGCTCTCTTATTTAAACGACTTCTCCTTCCACTGAGCATTAATTAGATATTTTCAATTTGTTCTTTATATTATGGTATATATGGTGGAAAGGAGGCGTCTTTTACCAATTCTGAAGGATAAAAACAGTTCCTTTAGGTATAATGGCATTGACGCAATATTGATTTGTGGTATATCCTGATCATTTGGCCTTTTCTATGTTTTTTGATCCTGGAAATGCCTCGCATATTATCTCTTGCACTTAAGTTGTATACATTGCCATTGGTTGATTTCTAGCATACCTCCTCATTAAAGTGATGTCAACTTAGTTTGTAATTTCCTCACTTGTTGATGAGTAAGTAACACTAAAAGCTTTATGAATGAGACTCAAAGTTACTGATTTGAACCTGGATCCGCATGACCTGATTCTTCACTTTCAATGTCATGACCTAACATTGACTTTCAACCTTTTAAAAGTTACGGAACTTGTCAAATTTAAAATATGTTGATCTGAATAAAATAGTACTAGA

>TCONS_00042043

ATTTTTTTCTCCTCTCTAACAAATGATATGTTGATTGTTACTCCCTGGTGAGTTCCACCTGTTCCCGGTTAAGATAATGTTTCTTTCGAGGTTTACTTGTCTGCTCTGGAACTGGTTGTAGATCTACTTCATATATCTCTGTTGCTGGTTCTGATAGGCATCAGTAGAGTTATTGAAAGATCTAAAGTCTAATCTCACTTCATTTTATCATGGTAGATTCGTGTGTATGTGGTCATTCTATGTTTGTGTTACATGATGAGTAAATACACCAGAATGATGAGTCAAGGAGGAAGCTCCTACCAAGCGAGAAGGCGACTTTATCTTGATGATGGCATCATTTGGATCCCAAAGTTTGATATGATTGCTATGGCCGAGTGAGTCAAACTCACCTTTATTGGAAGGGTTGTCCACCTTGGTGGCAGAAGCATTAAAGCTTTGATTCTCTGCTCCTGAGAGTTCGCATATGGAATGTTGAAGGTCGGGTGCATGGACTGAACCTAAGAAATGGTCAATTTCAATGTGATTTCGACAATAAAATACACATGATGGTGATACTGAATATGAGGCCTTGTTATTTTAACCAATAGAACTTTGTGCTTGAGTGATGGGAGAAAGCCTATTCACAGGCTGTATCAGATTATAAACGCACTTTACCCCAGATTTGGAGGTGATGAGAAATCCATAAACCATTTCTTATTCTAATGCATGTGTCCTCCAGACGTGGGAATTATCGGATATTCCATCAGTTCCAGAACAATTCCTGAGTGGTTCCGTCCATGGAAATTTTGACTTCCTCCTACTTACAAAATCAGCAGAAGGCGTACCGGCAAAACTATTATCAAAATTCTCAAGGATTTTGTCATACTTACGGAAAGCCAAAAATGATAAGCTTTTCATCAATAGACATCTCGCCTTTTCACATTACAGAGGGTTATTCAAGAGACCTAGGTTTGGAATGTTGCACTAACAGTTTTAAAGACAAGAGACATGAGGGAATTCCCAATCAACCAACCGGGAAGAGAAGAATATGTAACACAGAACAAGGTGTCAAATGGATGTATCTTGGACAAATGATCAGTCTACTTTGGATGGAGGTTTTGTTATAGAGCAGGAATCGGGAATCATGGTCTTTGGATCTCTAGGAAGGAGCCAAGTCCATCCACCCATTCACATGGAGTTCAACACACCACTATGAGCAATGAGTAACCCTCTTTGTATGGGTTTCCATCTATGCATTTGGCATTGGATTAGATGCAAATGGTGAAGCCGATCGATGACGAAGAAATACAATCGTCTCTCGCTAAAGAATAAGAATATATTGTTCACCTTCGTTCTATGTAATTCACCTTATTTTTACTATTTTTATTTCTCTCGATTTTAGTTTTCGAACTGACATCTTTGCAAAATGAGTTTGTGCAAATGTTTCTAAATTTTCTATATAAACTATATGGTTATGTCATAGTTAGTTTC

>TCONS_00072897

GTGATAATATACTCACCAGCTAGAACTGCGACTCAGCAAGGATCTGGCAAACTTGGCAAATGGAAGATCAACTTCCTCTCCACCTTAAAGTATCTTCCTTCAATCTATTTTTGTTTTGTTTTGTAAATGGCGCTTCGTGCTACTCAATCGACTGGCCGTATCGCGGCGGCGACGCTACGGCGTGTCGCTCGACCTTTCTCAACGGACGCGGTGGTGGAGACGGATTACAAGCGTGGGGAGATCGGTAAGGTCTCCGGAATCCCCGAGGAGCATCTTTCCCGCAAG

>TCONS_00005850

GCGGACTTTCCTAGGACGCCCAGGTGGGCGGCGTACTGCTGGTGGAAGTAATGGAGCTCTAGTTCCAGATCCGAGTTCTAATCCACCAACAGATGGGACCGGATAGATTTTTCCGATGAAGCCTTTGCGCCATGTTGGTACACGGTAAGCCGGTGCCACCAGCGCATCTGATGGAAATCCAACCCTGGTGGAACAAGCTAGCGCATGCATACAGGGTATCCCTATTAGTTGATACTCACCACATGTGCAAGTACCGGGACCCAATAAAACTGTGAAGCACTCCCCGGTGGGGTCCTGCACTTGGTATTCAAGATCGGCTATATCACGTACAGCCATAGCGGTGGAGAGATCAAAGTTTTCCTCCACGAGTTTCCTGACATTTGGGGTGATAGTTCCTTGCTCCCGGGTTGATTTCGCTCGACGCAATGCGAACCAAGACATAACTGTTGTACGTATGTACTCAAACATGGTAATGAGGGGATATTCACGTGCTTCCTTAAGCACCCCATTCCAGGATTCAGCTATGTTTGAATCCATGATGTTAAAC

>TCONS_00051964

ACCTTTTAATGTTTTGCATGATACTGGATCGTTCTGAGAACCAAGTACTTCCAGCTATCCACCTGCAAATGAAGACAACAACCTTGCGAACAATAGAAACATAACACGGTAAATGACTTATTTAGAGGTATCGACAAACTGCTCTTACTGGAGTAAGAGAGGAAGAGTAGCCAGAAGTGATCTATACAAAAAAGCAAGACGGAAGGGAGAAGTACAAAAATATGAGAATCAGTTCTACAAAAGTCAAAGTGAAGGGACAAAAAAAACATGGTAATTCAAATCGTCAAGGATAATCGATGATTGACTTTTTAGAAGTTCTGGCGAAGATGAATGACTTGATTGCAGAAGCTCTGTTTAAATGCTAGAACACCCAAGTATCGTGATCTGAGGAATCACAGAATCTACACTGCAAGATTCATCTTCCCCTCGGTGTCTCTTTTATTTTTTTGTGTCGGTGAGGCAGAGATGTATTTCTTCATCTCAATTCATCAACCTTAAACAATCTCCTAATCTCAATCGAAGACTCACCTAACGAAACGAAAAAGATTGTCCC

>TCONS_00022046

CAACTATGTGCCCATCAATAGTCATCGCACTTGAAGCTTCGAGCAATTGGACTTGCCTTCCGCCGACGGGAGTGTCTTTAAACATACTCTTTTGGATCATGTGGGCGTTGTGAAACACACGAAACCAACTCTTATTTGAGTCGCGCCCCCAATCATCCTCAGAAGTGGTCTCCAAGGCTTTGGCAGCAGCAAGGGAATGGCAGCTTGCTCAGAACCCAATCGACACCTCACGTAATACGCAAAAGGAGCCATCTATCACTCTGACGCCGACGATCCCCTCAGGGACAATCCTATGCAATAGCGACGCAGCGTGGGATAAGAACTCGGAATCGACGGGACTTGGATGGATCTTTACATCTCCAGGAACCCCAACTCTTCAAGGCACCCAATTTCAGACTCACGTGCAATCGGTCCTCTTGGCTGAAGCCCTAGCGGTACGATCTGCCCTCCAACACGCCATTCGTCTTGGCTACACACAAATCTGGCTTAGATCAGATTCTCTTGTGCTCATCAGAGCCATCACTTCGATCAACAAACCGAAGATCATCCACGGAGTTCTCTCGGATATCGGAGCTTTATCACTTTCCTTTGCTTTCTGTTTTTTCTCTTTTATTCCTCGGGAGTTTAATGGACCTGCGGACCACTTAGCAAAAAGCTCTCTTTGTA

>TCONS_00068290

ATAACGGGAACCTGCACTCAACCATATCAACACGCAAGAATAGAAAACATGATGCATCCTAGCCCTAGTCCTAGTCAGGTTATTAACTCAGTCTTAATTCCATTCATCTCAGCTTAGCCCTTGCTAAACTGAGTCCGGTTCCATAAATAAAATAACCCTAAGGACTCTACCATTCAATAACGTGATCATTCTACTCTATATGCGACTCGATCTACCCTAAATTCCAAGTGGAATTCAAACAAACCAACTCACAAAACTACCCTAAATTCCAAGTGGAATTCAAACAAACCAACTCACAAAACTACCCTTCATGATATCAACTTAATGAACATAACCTCAATTATTAACTACTCAAACCGTTACTCAGTTAGACCTGGCCTCCTGCCATGATCCAACTTCCAAGTAACGGGACCCTGCATAAAAACAACTCAGCAATCAATTGATTATAACTCACAGTTTTTGGTTGACCGTGGCCTTGACCCCGAATCCGTCTTCTGCGTTCCGAACCCTTTCTTGACCTTCACAAGTAGACCCACTTCTGAACTGATCTTGAGTCGAGCAGAACCTCTCTTAGATGTAGACTCGAATCGAGTAGAATAACTGTTCTCGATAACTGCCGAAAAGACTCGAAAACGATCGAAACTCGAACTTTCTTTCTCTAGCTTTCTCTCTCACGTTTTTCTCTGAGTTTCAGGTGTGCTGGATGGTTTGAAATGAGCTGGGGTCGTGGGGTTTATATAGGATGCAGCAACCAATCAGAACAAGCCGTGTGGCAGCCCGTGTGTCGCCTCGCATGGCTCCGGACGCATGCGTCGCGGCACCTCGTGCTCCACATGTCTGATGGCATGACCAGGACATCATGCAGAGTGACACCATGCCCTCCAGATGTCTGATCCACGGCCTGACCTCATCCAGATTGACACTCAGCGCACACATGTCGCTCAGCATGCTCCGATCGCAGGTTTCGCGGCACCTCGTGCTTCTGGGTGTCAATCAGCATGCTGTGCTCTCCTGGGCTTCAACACCTCCTGCTTCCCTTGCCACATACCATGCCAGGCAGGTTACATCACGTCCTTATCTCATAGATAAGGCCAGCTCGAGCTTCTCGGTCCATTCGACTGATTTCGACCCTTCCGGTGAATTTTCGTCCCGCGATCAATCCCGAATATTTTTCTGCGCCCGTTCTGATGAGATGAATATTTTTAATAAACTCCAAGTGAATCCTGACCTTGATGGAAAATATTTCCCGAGCCTCCGGCTTCCTCAAAGAATTTCGTAATACCGAAATTAGGGTTTTCGCCCAACTTCGGGTTTTCCCGTCGTGCTTCAATCCCGTCGTGCTTGCTTCCCGTCCTGCTTAATTCCCGTCCTGCTTCCGACTTATAATGTCTTCAGAATAATATTTTACTGATACGAAGATATTCCGAGAAAACTTCGTGATGAAGAAACGTCAGTCTTCAAAAACGCCGAGCTTCTAAACCGTCGTGCTTCCAAAAATGTTATGCTTCCAAAACATCCTTCTGATCAATCTAACACTTTTCTAACCATGGTCCAGCAGGTTATGGTTCACCCATGATCAATTCTTCGACCATCCATTGCCCGCGGTACGACCACTA

>TCONS_00010032

GATCCAACTCCAAATAAATTAAATATATAAATAAAATAAATTGAACTCTTACCCGGGGTTTCTTTTACAAATTTATTTATTTAATTTATTTGGAAAATAACAAATCATTAGTAATAAATGAGGATGTCTAAAAATGTCAAAGTGTTGGTCTCTCTAGTTCTTGTAGTGTTATTGGCTCTTGCAGCAACCAATATTGAGGCAAGATACATAAATTATCGCGATCTTCGTAGTGGAGATCACAGTTTGGCTTGCGATACGGCGAATTCTAGCACCTGCACGAAGCAAGAAGTCAATCGTTACCAAAGAGGATGCGAGTCGGCCGATCGGTGCCGTGAATCTCGTTAATTTATACAGATGATAACCGAAGAGCATATTCATGTATCAAATTAGTTTCCTAATTACTATAATAGATTGACAGGCTTCTAACTTGGAGAACAAATC

>TCONS_00020041

ATGTTAAATGGGCTGTGCGATTTTTTAAAAATTTGTCGTGGTAGAGTTGGACCATGAAGCCCATGTTATTGATAGAAGAAAACCCTGATTTCCTCTCTTGATTCGGGACACCCTTTTTGCTCTTTCTCTTCTTCATTCCGTTAAACGATTTGATCGTCGTCGGAGAAGGTGAGACGGCTCCGGTGGGAGCCACGATCGGTTTGCTGGCGGAGACTGAGGCTGAGATCGATCGAAGAACCTCAGAGCAAAGCCGCCTCGAAATCTTCCTCTGCCTCTCCCGTTGTTGTTCCGTCTCCTCCTCCTCCGACTGCTTCGTCTCCTGCGCCGGCGATCGCTCAGCATGCTCCGGTGGCGACGGTGTCTGACGGTCCGAGGAAGACTGTAGCGACGCCGTATGCTAAGAAGCTTGCAAAGCAGCATAAAGTGGATATCAGATCCGTTGCGGGAACTGGACCATTCGGTAGGATCACTGCTTCTGACGTGGAGGCTGCGGCTGGGATCGCTCCCTCCGTGGCGGCACCACCGCCTCCTGTGGTGAACGCAAGCTGCAAAGGTTTGCTCTAAGATTGATTTTTCATCAAATTAACAAGATATCGTTGATCACTGAGATCAGTCCTTTTGTTGACTTTCTTACATTGTCTCTTTTATCACCTGAAGACTGTAAAACCAGTTCTATGTTAATATGGTCTCTCCTTCTTTTGTTCAAACTTTTTGTGAAAATTAGAAAAAATAAATGTTTGAGTAAATACAATCAATGGGTACACTCATGTTTCTGATTTTTAGGAGTTATCGGACCTGTAACGAAACAGTGATTTGCTATAAAAGGTGAGTGTTTTCATTCAAAAATAAAAATAAAAGGTGTGTTGCTTTTTGTAATGTTTCCTTTGGATTTATATTTATTGACACAATGTTGTTTTATCAGTTGATCTGATTTGCTTTAATATTGCTCTGTCTATACTGTGATAGTTTAAGATTCTTCCTGTGTGGGAGAGCAGGCCAGGTGAAGCAACATAAAAAGCGTAGTATTCACTACACCAAAGGAAACAGAACTTTGCCAGATTGATGTTCCGTTGAAATTTACAGCGTTCATGTATACCAGAGTGCATTGGTTCAATGTTCTCTTTAATGGAAGCACAAAGATGGTCCACGACTGCTACTGGTCACCAACAAGGCAACAACACTTTGGTAACAAATAAGATGTATTCTCTCCAGCCTATTCATGTCATGGCAGGGCAAGAAATCAGTGGTAAACTTCATTTGGTTGTCCACAGGAAAAGTGTGGGGCCCAGCGCCGATAAGGGTGGAATCCTTCACACATCATCGTGCAAATTCAACTTGAAGGAACCCTATTATAGAATGTCTCAGCCCTCTTGCACAAGAACCACCAGCACAACCGTAAGTAAGGTTGGTTATTTTTTTGGTTAGACAAGTAGGCTATGATCCCTGTTGTGTAATCATTTGGAGCTTTATTATCTCTATGAGTGGAATACTATTGAAAGAATATGTCATGAATGCAAAAGCAGAAGTAGATCTCAGCATTACAACATGTGTTTACATTTCTCGGTGTAGGACATACATATACTGAGTGATGACTTGAAAGAAGTAGAGTTGATTACCTATCTTTTTTCCATCCAGTTGATTACCTATCTTAGCTTATATTTCTCCATTATAAATTTATCACTTTTAAATTTGGTATTGGAAATAAAACTTGGCAGAGGTTAAGTTAGCGTCTACGCTCGATCACATTGGTAATGTGGCTCAGCTTTCTCCTCTTATTGGCTTTGTACACAAGCATGTTGTGATTGTTGATACTGAGCCCGAGCTAGAGAAATCGGAATTTTTGGTAATGTCTTTGGAGAAATGTGTTAAACAGCTCGAGGAAGAAAGAGCAACAGAAGAGACTTATCATCATCAGCAGCGGAAGTGTAAACTCTGAAAGATGCAATGCATCTTGCGAGAGGCAAGAGATGGGCCAATTTAAATATGGGGTTGAATTAGCAGAGACAAGCTATTGAATATATAGTATTTAGTTTTCTTATTTTTGTGTATACTGGATTTCATTTATTTCTCC

>TCONS_00009417

GTTTGTTACTATCTAACCACCCAAGACAACAAACTCAACACATTCTTCATCTCTCTTCTTTCATCATCTCTTTCTACTACGCACCATGGGATTTTCTCGAAAGGAAAATCTCAACACAAACACTTCCACTTCAAACCCTAATTTCTCTTGTTCTGCCAATACCCATAATAGTTCATATGTCTACCACGGCCCACAAGTCCATACCGTTAGAGAGTCCATAACATCTTCTACAAACGCAACTTTTTTGGTCTATCAATATCCAAAGAACTCTGTCCTTGTAAAACAAGCAGTTTTGCCCGCAACTATAGAGTCAGCTCAACGTTTCCGTTACTCGTCTCTTGCTTAAACCTGCCCGCAACTATAGAGTCAGCTCAACGTTTCAGTTACTCGTCTCTTGCTTAAACAATAACCAAAACTAAAGGCGATAAATAAGATCACTGTTCCAGGTTTCGTTGTTAAGGGATGAAGTTAGAATTACGGGAAAAGTTGTCGGAGTTGGGTTGCAATCTTTGTTTATTAAGTATGGTATGAGTGTGTGAGTGCAGACCGGCTCAAACTTACTCTCTTTGAGAGTTAGGTTGTAATATAATGTTACATTTGTGTGCGTGTCTGTTTGTAGCATGTTGAAAGTACATATATGTAACCAGGT

>TCONS_00034556

TGATGTGCCTCTCACAATGAGTTTCTTCGACAGACAAGCTGTAAAGTTTCACAAGCAACTTGAAGCCATACGTGGTGGTGACCCAAGGGTGGTTGTTGCAACCGGCATTAATCCAAAATTCGAGGGAGGTATTTAGTTATTCTCTGTAATTTGATATTCTAACTTCTTAGGTAACACAAAAGTAGGCATGTTAGGATATGACCAGTTTCAGCCTATGTTTGTGGTAAAATCTCAATATTCATCAATGAATCTCAATTCCAAGAATATCAAATCTAAGGGGGCATGTGACGTATATAGCCAAGAAAATAATTTTTCCCGTGATATGATTTTGAATTGTTTTATATTCAATTGATTTAATAGTTTGTTTCATTTTCATTAGCCTTCTTATTTTGTTTCATACATAACTAGATAATCTAAAATCTATAATATTCGGGATGTGTACATGTAAATTTTCCAGCGAGTCGGTATAGCTGAAACTGATAGTTTTCTAGATCCCTTGGCAATTCCCTCGAAGTATTATGAAACAATATTTACATCTAATCTAATTTTAGTATCACCTAAAAAGCTTTTGGTTCTTCCTTGCAGATAGTGTTCTTGATCTCACGATCAATAGTGTGAAAATAGTTGTAGTGGTAAGTGAGGTGTTGTTGTGAAAACATTGTTATGCTGCTTCCAAATATGAAACACGACAGCCTGAGTTACCATTTTCAAAAGGAGCCGCTGCTTCCATTCCATAGTAGCAGCAGATACAGTACTGGCGTTGGAGTTGTTGGTTTGATTGCTTCTGATCCATGCTAGCAGTTCTGACCCATTAAGAAACTTTTGCAATGGTCCGCTTATACATGAGAACCATACTCCATATATTTGAGTTGTAGTCACAGCTGAGAAGAAGATGATCTCTGGTTTCTGGATCCCGAGAGCGGAGGCAGCTTGATGTTTGGCTTAGTATGCTTGATGTGAAAATGTTATACGTTCTATGTAGACATACACAATTAGTACTATAAATTGTTTTGTTTTGTATTGATACATTTTTATTAGTTAATACAAAGAAGGGTTCATTTCTGTTGACCAAACATGTGAAGAGCAAAGATAAACAAATCAATTAAATACAAAAAGCTGAAATTAGTTACTGATTTCTTAATAAAAATATATACAACCCATTTGTATATATTTTATATGTGGATTTATATAAGAGTTATTTTCATATATAGACACTTTATGACTTTTTATTACAAGAAAGAGCACTTGTTGCTACCAGGACACTTTTTGTTGGTTAAAGTCATCTTTGTCCTCAAAAAAGGCAAACTAGACGTGGGCATATCTGTAATATAACATATTCTTCTGGTTTCTCTGGTTTGGGAGTTTAAAATTTAAAAATAAGAGAAGCTGATTGGTGGATTCCATAGACGTAGTGTGTTTTGCTTAATTATCTGAGATTGGCCCCGTGATAAAGTAAATAACTCTATATGTTTCAACTTTTCCCGAGTCTGATAACCTAATTTTACATTTCCCAGACCGTTTTTCCGACGACGACCAACAGGATTGGAGAACTGAGAAGCCGACGAATGAAGAACGCGTGTAAGAACGGAGAAGCAGACGAACAAGATTTTCGGACGGAGAAGACGACGTCGTCTCGAAAGCGGAGAGCTTCAAAGGACAAGGCTAAAGGTTATATACTTTCCTTCCTCGCAGTTTCATCGATTTGGATTTCGGATTGGGGATTAGAGTTTTGATTTCTGGGTTTCAATTTTTCGAGTTTGGTAAGAGCCTGGCGTGAGGTGTTAGGGTTTCGATTTGGGGGCGCGGTTATAGATTTATGAAAAACTTCAATTAGGGTTTTGATTTGTAGATTAGAGTTTCGATTTAGGGGTGCGATTCTAGATTTCCAAGTTCTCTAAGTTTTCTTTCTGGTTTCTGTTTGTTTTACAGAGATGGCGCAAACTGAAGAGGGTGCGGTGAATGGAGGAAAAGGTCGAAGCTTGTTGCTAGTTTTGGTGTTTTGACTGGTCGTCCAAGTGCGGTTGCTCTTCTACGAAACCAAGAAGACGTGGAGGGGAACTGATGTCTCTTGCTATGCTCTTATTGGATTCGAAGCCCCTTCACGCCGCCATCAGGGTTTCCGTTCTTCTTCCAAAATCTGAGACCGATGTAAATAATTATGCGTTCTCACACAGTTTCAATGGTGTTTTGATTGTTTGGATTTCTGAGATCGAGATGAAGAATCTCTTCGTTTGTTTCCACCGCGTCGTTTAACTTCTCCTCTACTGAAACGAAACGGCCGAGTTACACATTCAACGCCATCAGCGTCATCATCGCCTTCTTAACTCTATAGACGGACGTGTAGCACGATCTTGACATTGATTGTGAGTTATCCCTCTCCAAGGCGGTCTATTCTTCACCTATAAAAAG

>TCONS_00033963
[truncated: 2,955,589 more chars]
