# Supplementary material for: Integrative analysis of genome‐wide lncRNA and mRNA expression in newly synthesized Brassica hexaploids
Source: Ecol Evol. 2018 May 15;8(12):6034–52. doi: 10.1002/ece3.4152 (PMC6024132; doi:10.1002/ece3.4152)
Supplement: Supplementary file 2 [file ECE3-8-6034-s002.docx]

**Integrative analysis of genome-wide lncRNA and mRNA expression in newly synthesized *Brassica* hexaploids**

Ecology and Evolution

Ruihua Wang^1^, Jun Zou^2^, Jinling Meng^2^, Jianbo Wang^1^

Corresponding author: Dr. Jianbo Wang

College of Life Sciences, Wuhan University, Wuhan 430072, China

E-mail: [jbwang@whu.edu.cn](mailto:jbwang@whu.edu.cn)

**Figure S1 Quality scores across all bases in reads for three cDNA libraries.**

**
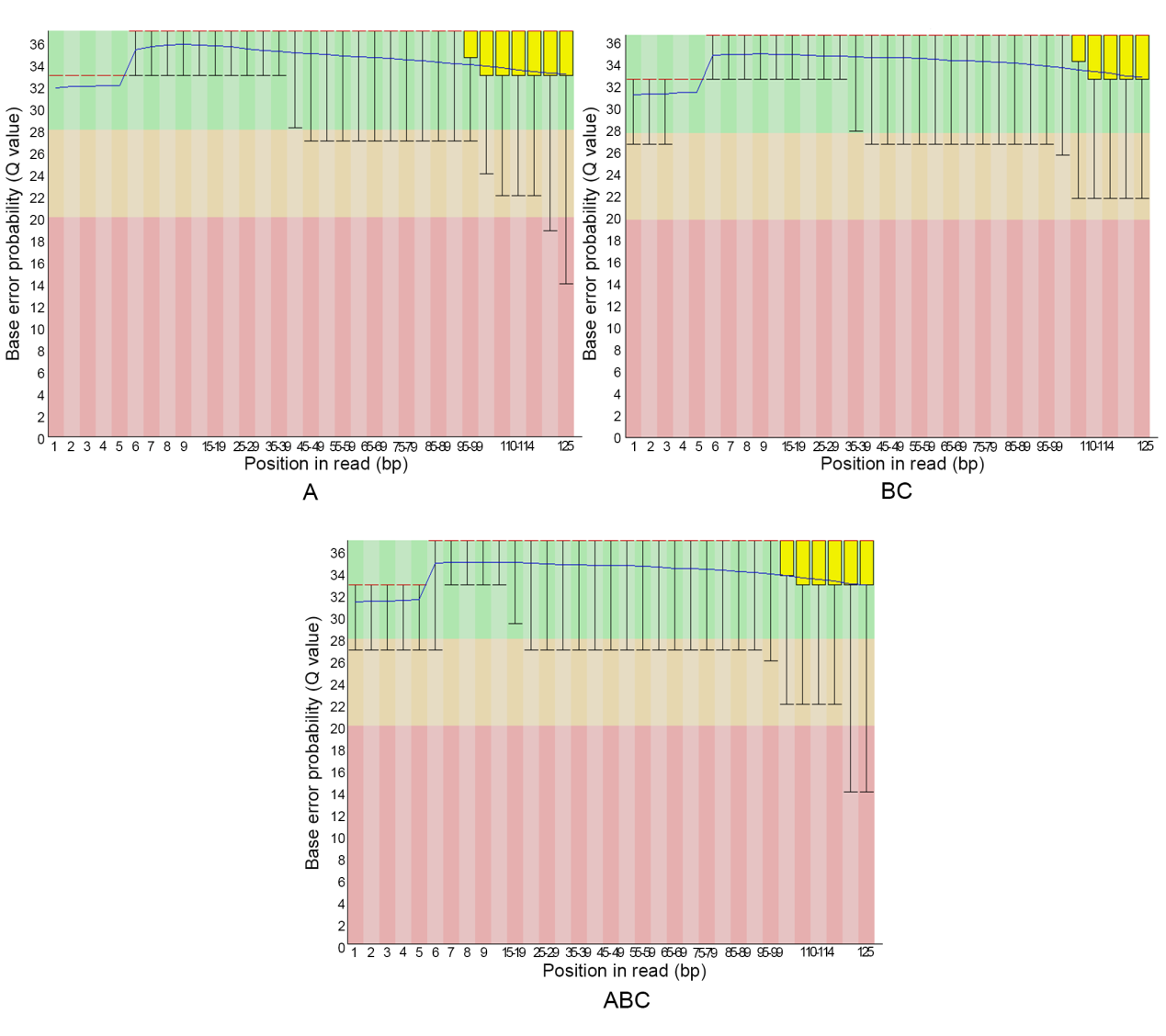
**

A, *B. rapa*; BC, *B. carinata*; ABC, *Brassica* hexaploid. Q20 shows that the base error probability is 1%. Q30 shows that the base error probability is 0.1%.
