# Supplementary material for: Integrative analysis of genome‐wide lncRNA and mRNA expression in newly synthesized Brassica hexaploids
Source: Ecol Evol. 2018 May 15;8(12):6034–52. doi: 10.1002/ece3.4152 (PMC6024132; doi:10.1002/ece3.4152)
Supplement: Supplementary file 3 [file ECE3-8-6034-s003.docx]

**Integrative analysis of genome-wide lncRNA and mRNA expression in newly synthesized *Brassica* hexaploids**

Ecology and Evolution

Ruihua Wang^1^, Jun Zou^2^, Jinling Meng^2^, Jianbo Wang^1^

Corresponding author: Dr. Jianbo Wang

College of Life Sciences, Wuhan University, Wuhan 430072, China

E-mail: [jbwang@whu.edu.cn](mailto:jbwang@whu.edu.cn)

**Figure S2 Sequence content across all bases in reads for three cDNA libraries**.


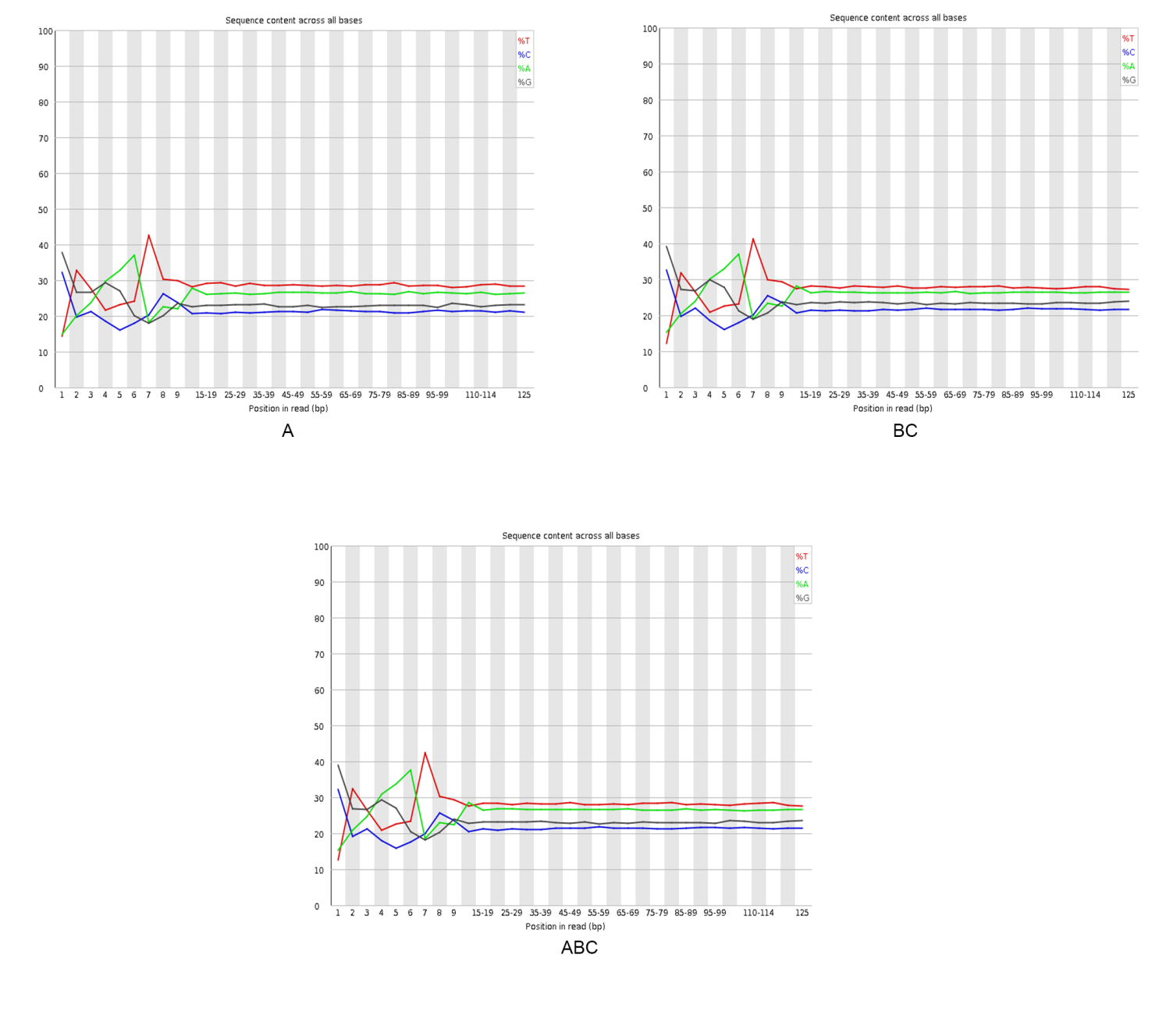


A, *B. rapa*; BC, *B. carinata*; ABC, *Brassica* hexaploid. Different color represents different base types.
