# Supplementary material for: Integrative analysis of genome‐wide lncRNA and mRNA expression in newly synthesized Brassica hexaploids
Source: Ecol Evol. 2018 May 15;8(12):6034–52. doi: 10.1002/ece3.4152 (PMC6024132; doi:10.1002/ece3.4152)
Supplement: Supplementary file 4 [file ECE3-8-6034-s004.docx]

Integrative analysis of genome-wide lncRNA and mRNA expression in newly synthesized *Brassica* hexaploids

Ecology and Evolution

Ruihua Wang^1^, Jun Zou^2^, Jinling Meng^2^, Jianbo Wang^1^

Corresponding author: Dr. Jianbo Wang

College of Life Sciences, Wuhan University, Wuhan 430072, China

E-mail: [jbwang@whu.edu.cn](mailto:jbwang@whu.edu.cn)

Figure S3 Conservative motifs of lncRNAs associated with GO functional groups.

| GO: hydrolase activity | GO: nucleotide binding |
| --- | --- |
| 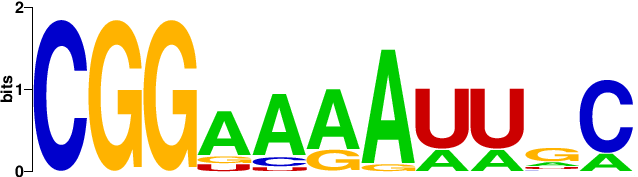  *E*-value = 6.0e-005  Width = 11  Sites = 15 | 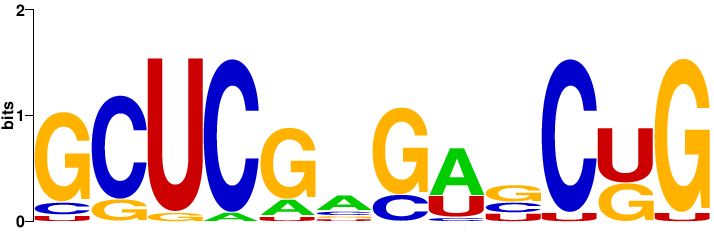  *E*-value = 3.0e-005  Width = 12  Sites =17 |
| GO: protein binding | GO: intracellular |
| 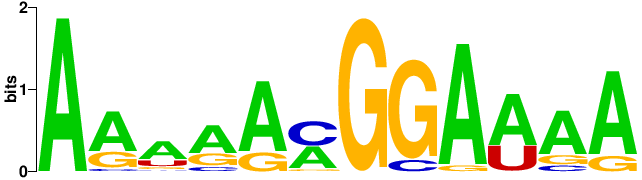  *E*-value = 2.1e-007  Width =12  Sites =18 | 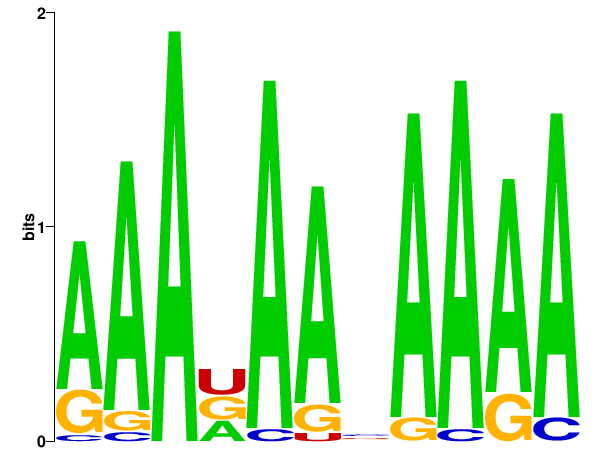  *E*-value =1.2e-003  Width =11  Sites =27 |
| GO: intracellular organelle | GO: intracellular part |
| *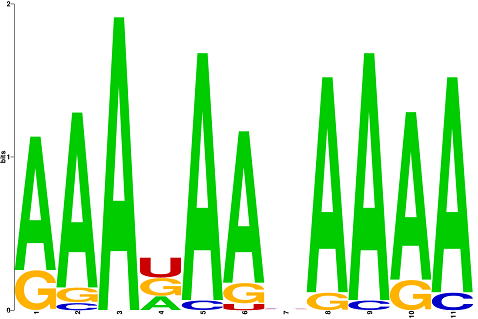*  *E*-value =2.4e-004  Width =11  Sites =24 | *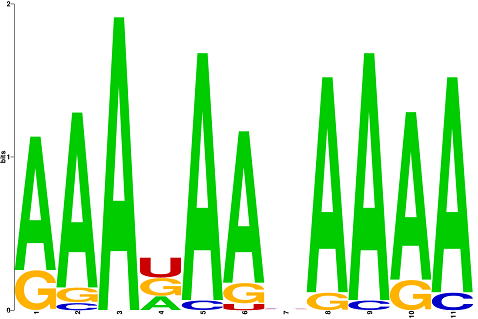*  *E*-value =2.4e-004  Width =11  Sites =24 |
| GO:non-membrane-bounded organelle | GO: organelle part |
| 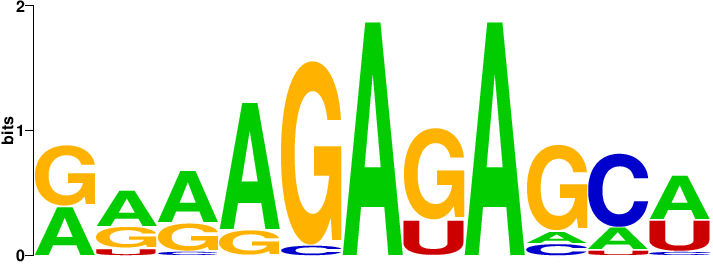  *E*-value =6.5e-003  Width =11  Sites =18 | *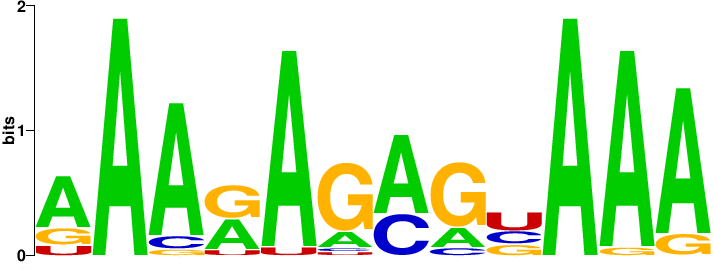*  *E*-value =6.7e-007  Width =12  Sites =23 |
| GO: biosynthetic process | GO: cellular metabolic process |
| *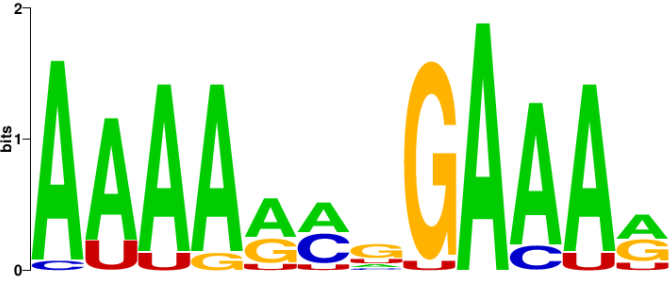*  *E*-value =4.0e-oo7  Width =12  Sites =20 | 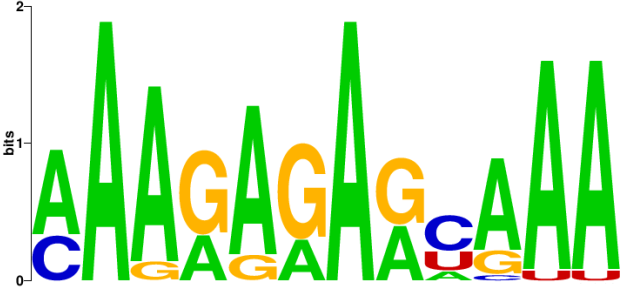  *E*-value =7.9e-007  Width =12  Sites =20 |
| GO: macromolecule metabolic process | GO: microtubule-based process |
| 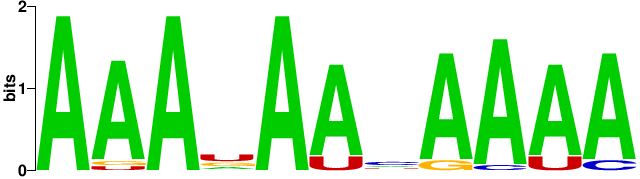  *E*-value =4.2e-007  Width =11  Sites =21 | 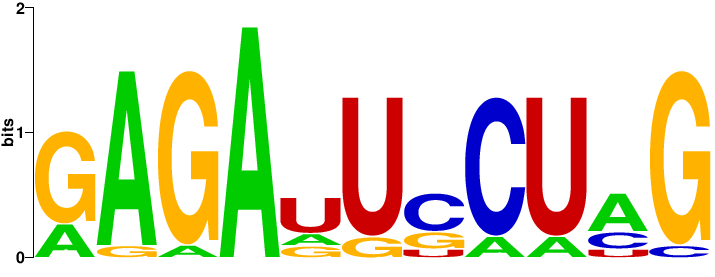  *E*-value =2.0e-oo4  Width =11  Sites =15 |
| GO: multicellular organismal development | GO: nitrogen compound metabolic process |
| 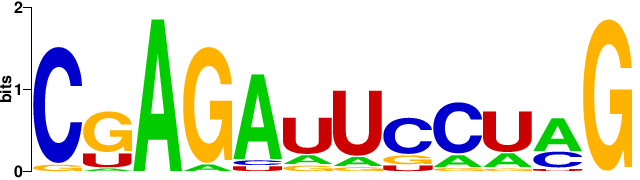  *E*-value =5.6e-005  Width =12  Sites =16 | 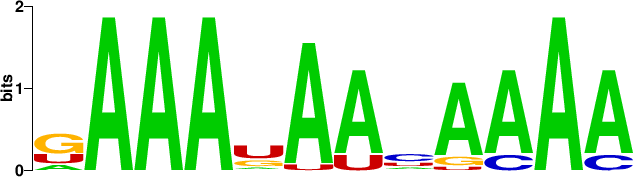  *E*-value =2.2e-006  Width =12  Sites =18 |
| GO: organ growth | GO: organelle organization |
| 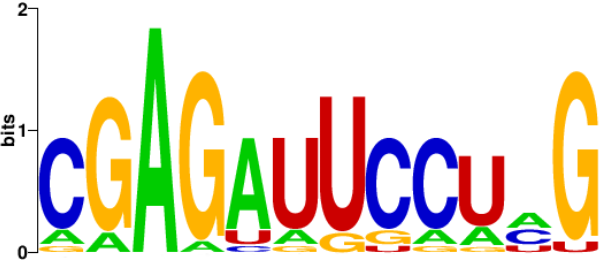  *E*-value =5.7e-004  Width =12  Sites =15 | 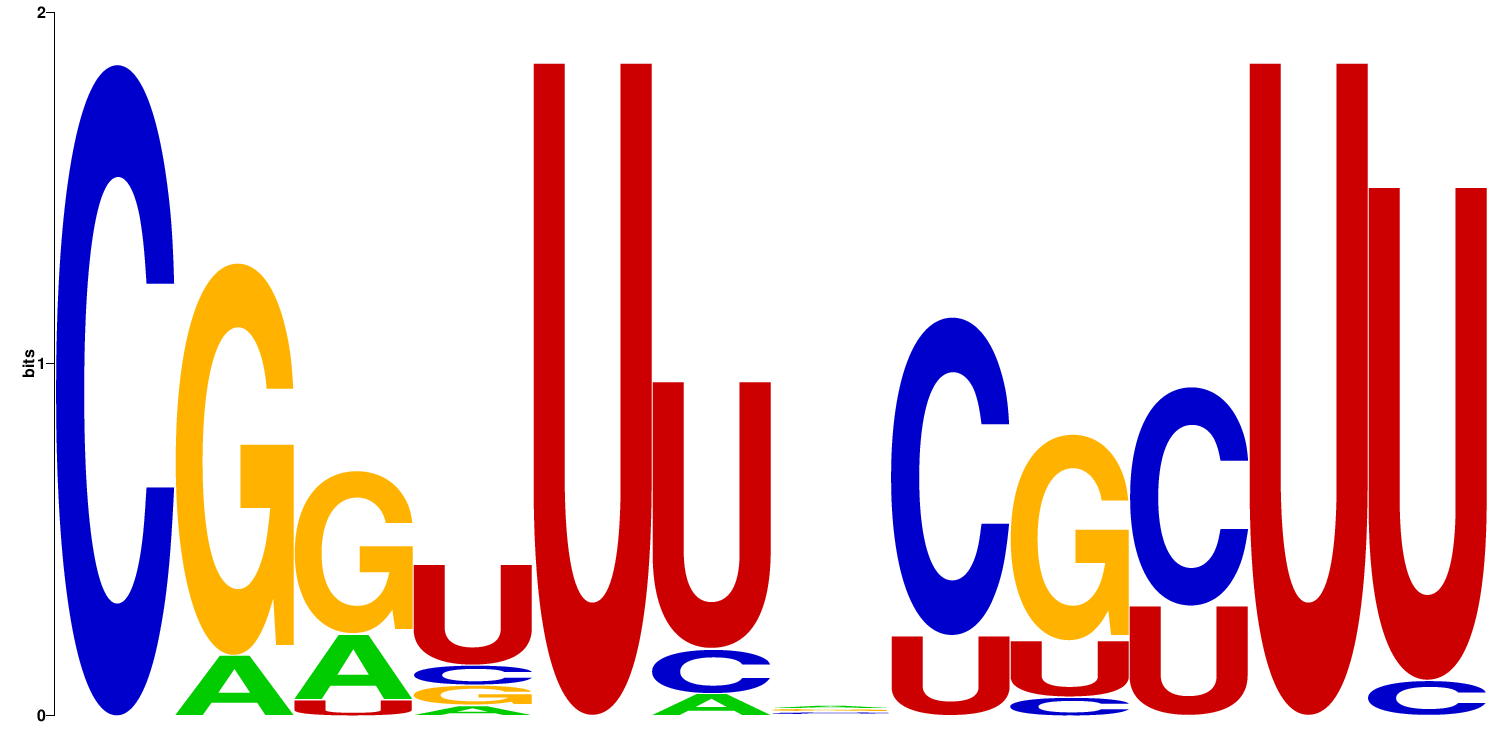  *E*-value =1.3e-002  Width =12  Sites =15 |
| GO: primary metabolic process | GO: regulation of biological process |
| 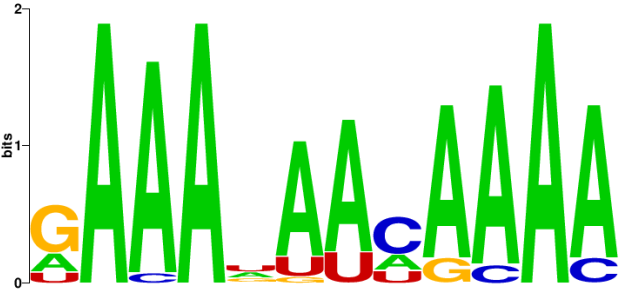  *E*-value =2.2e-007  Width =12  Sites =21 | 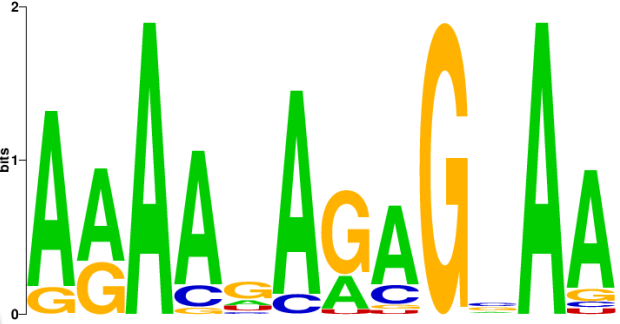  *E*-value =2.6e-006  Width =12  Sites =22 |
