# Supplementary material for: Integrative analysis of genome‐wide lncRNA and mRNA expression in newly synthesized Brassica hexaploids
Source: Ecol Evol. 2018 May 15;8(12):6034–52. doi: 10.1002/ece3.4152 (PMC6024132; doi:10.1002/ece3.4152)
Supplement: Supplementary file 5 [file ECE3-8-6034-s005.docx]

**Integrative analysis of genome-wide lncRNA and mRNA expression in newly synthesized *Brassica* hexaploids**

Ecology and Evolution

Ruihua Wang^1^, Jun Zou^2^, Jinling Meng^2^, Jianbo Wang^1^

Corresponding author: Dr. Jianbo Wang

College of Life Sciences, Wuhan University, Wuhan 430072, China

E-mail: [jbwang@whu.edu.cn](mailto:jbwang@whu.edu.cn)

**Figure S4 The predicted secondary structures of lncRNAs and miRNAs sequences.**

| TCONS_00023069 | bna-miR167c |
| --- | --- |
| 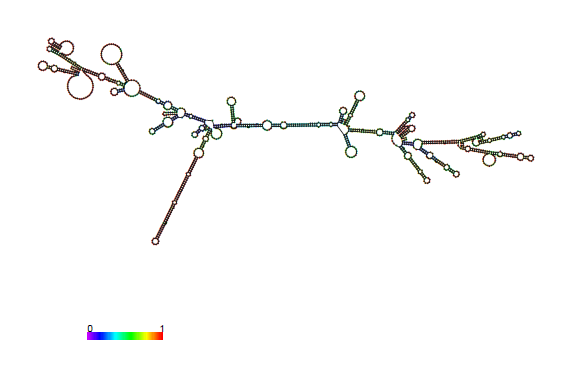 | 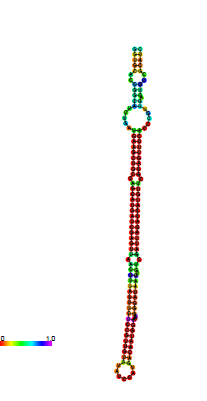 |
| TCONS_00025056 | bna-miR172a |
| 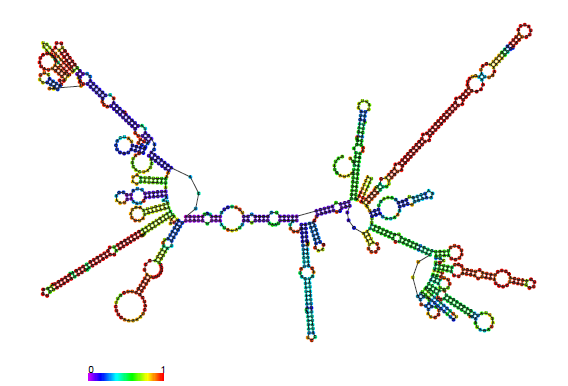 | 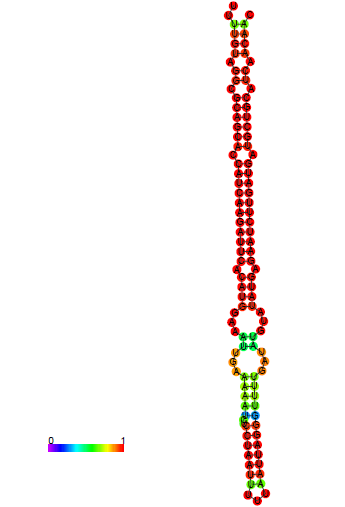 |
| TCONS_00033505 | bna-miR166a |
| 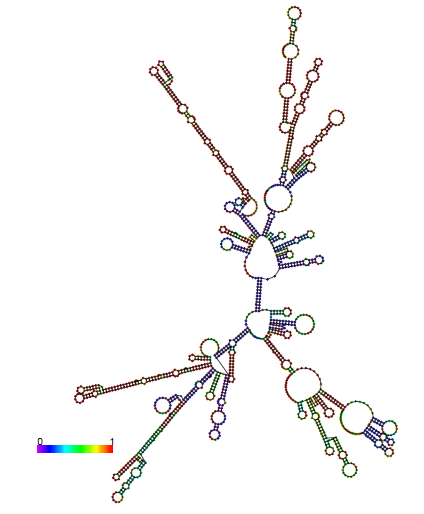 | 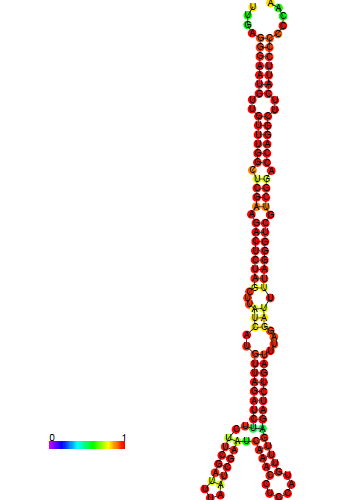 |
| TCONS_00040258 | bna-MIR403-5p |
| 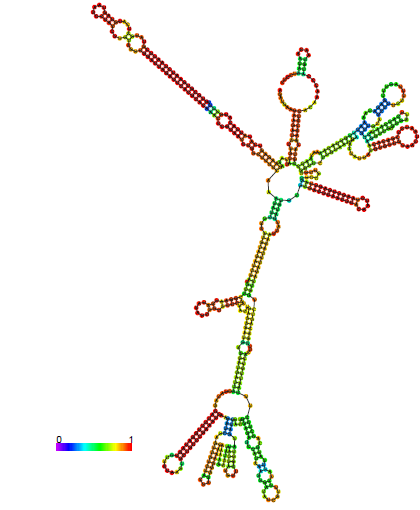 | 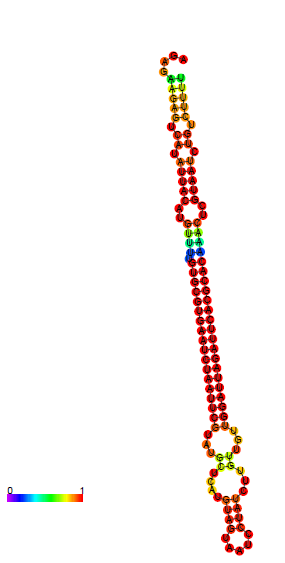 |
| TCONS_00042206 | bna-miR167a_R-1 |
| 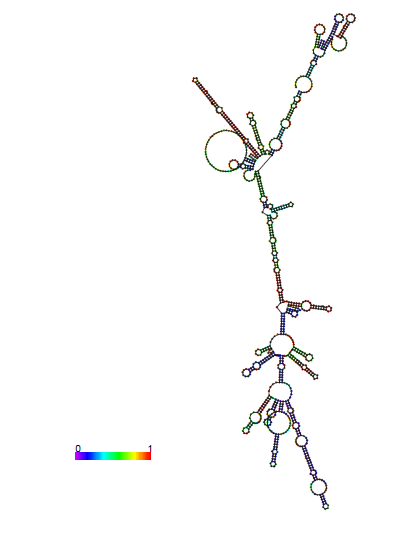 | 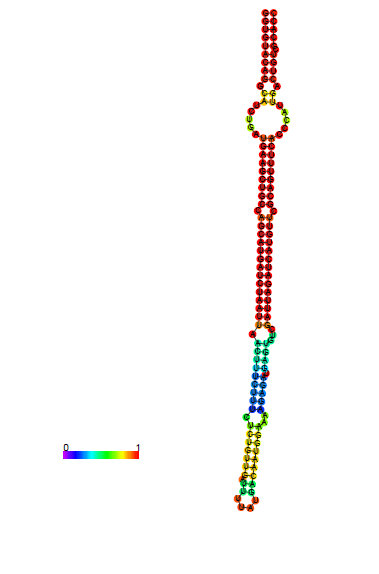 |
| TCONS_00047503 | bna-miR169g |
| 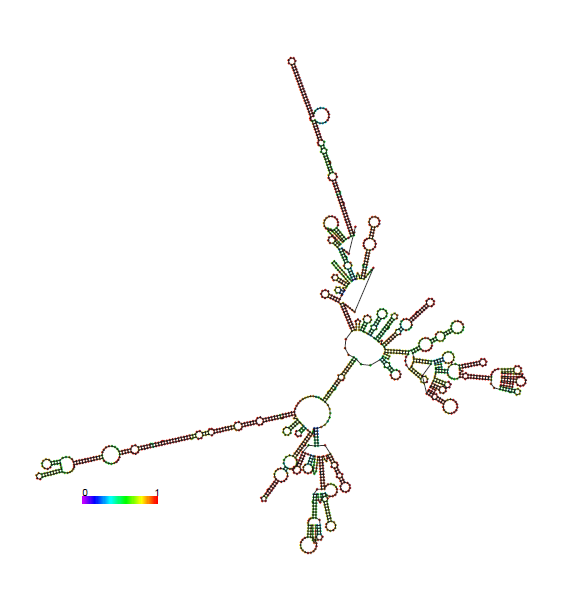 | 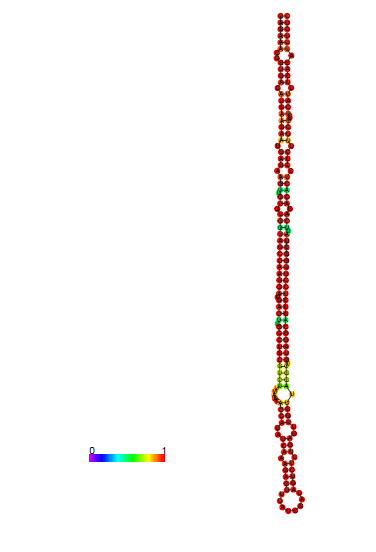 |
| TCONS_00059368 | bna-miR159 |
| 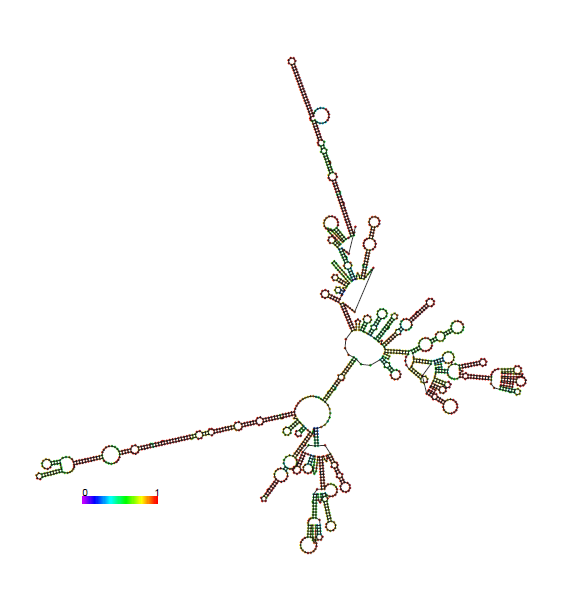 | 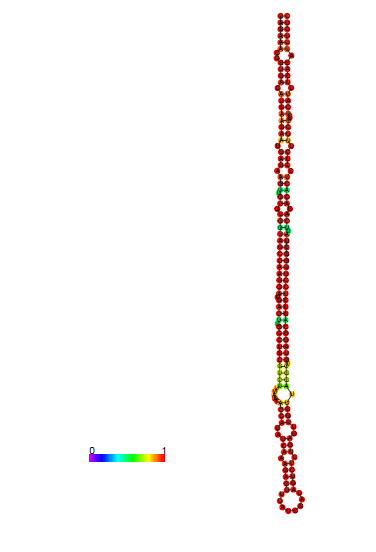 |
| TCONS_00004526 | bra-miR168b-5p |
| 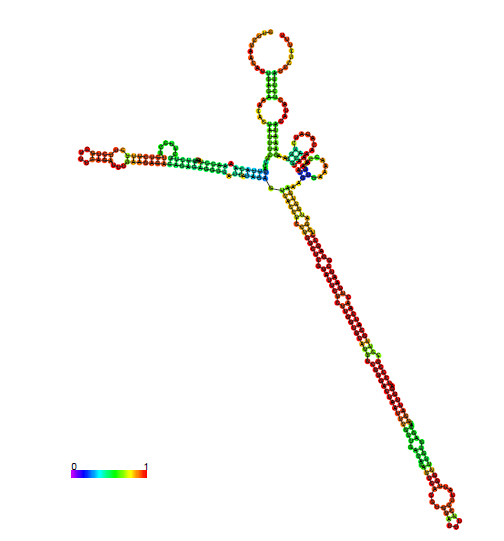 | 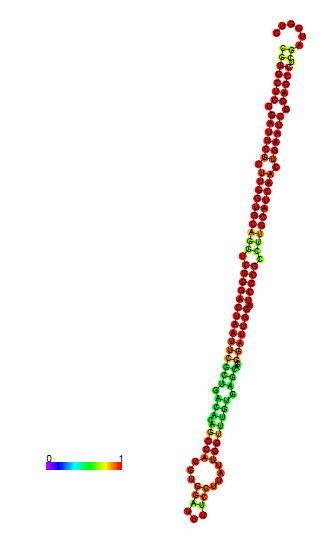 |
| TCONS_00019674 | bra-miR156a-5p_R+1 |
| 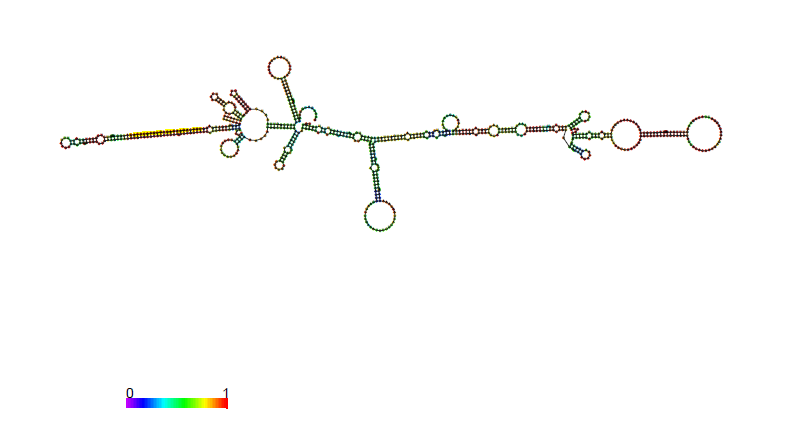 | 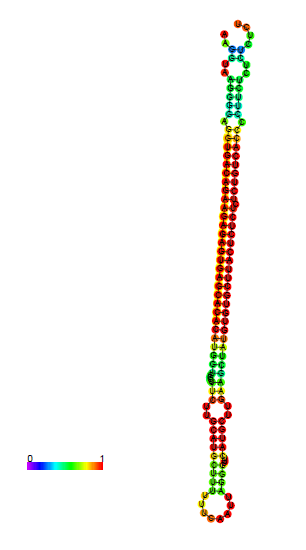 |
| TCONS_00019806 | bra-miR398-5p |
| 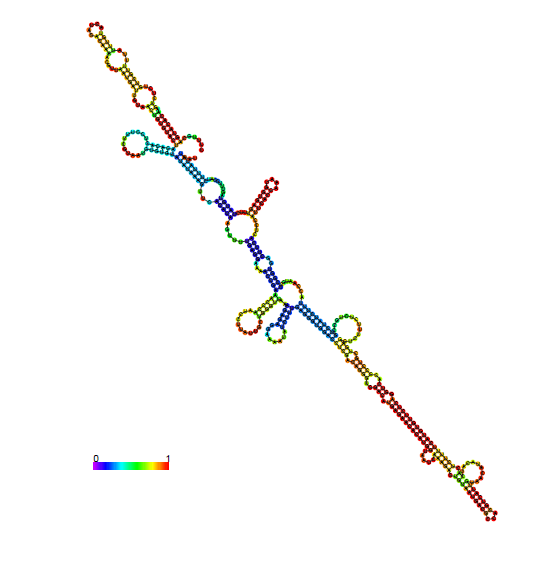 | 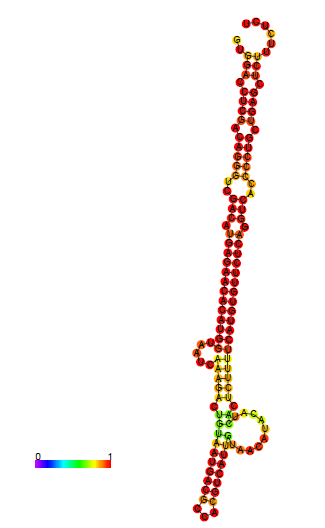 |
| TCONS_00023382 | bra-miR162-5p |
| 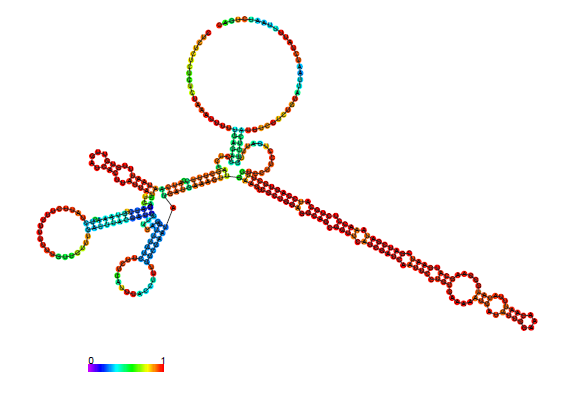 | 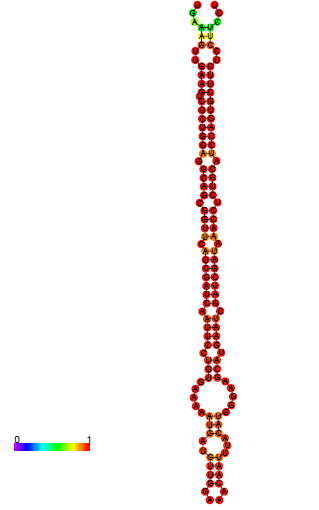 |
| TCONS_00027855 | bra-miR9561-5p |
| 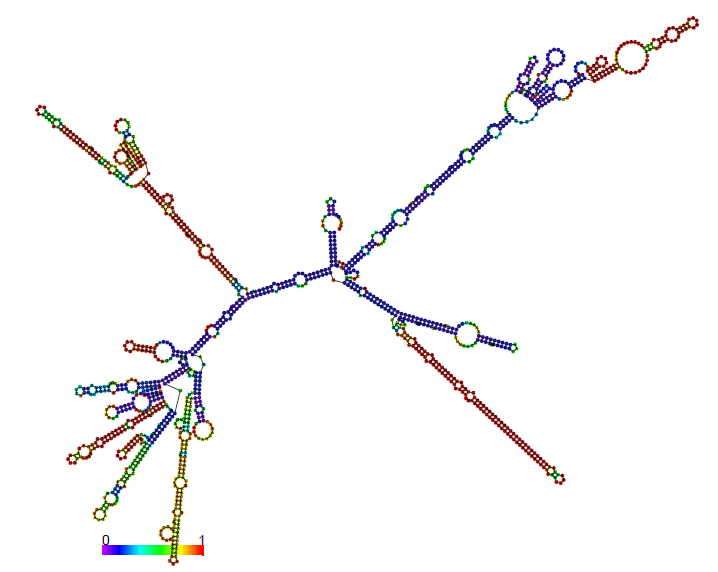 | 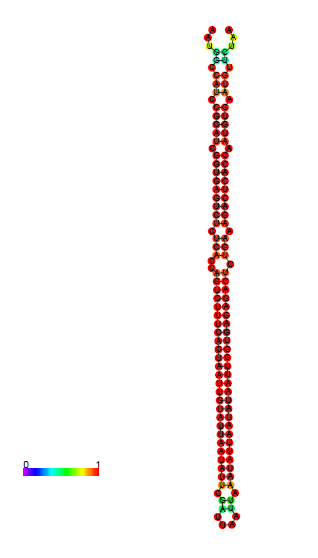 |
| TCONS_00060408 | bra-miR158-5p |
| 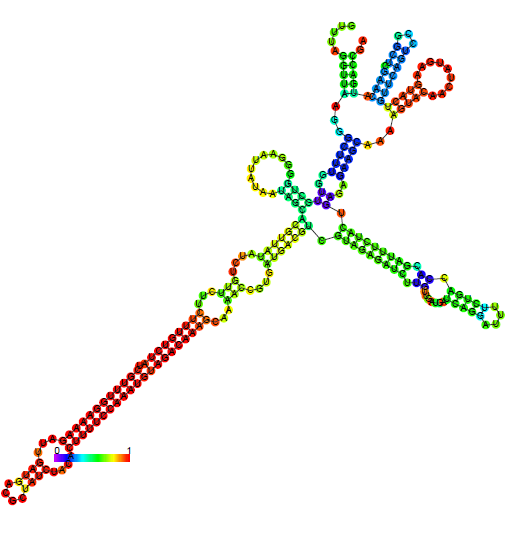 | 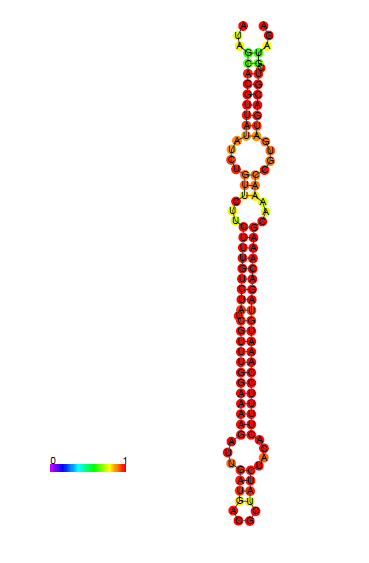 |
| TCONS_00074716 | bra-miR400-5p |
| 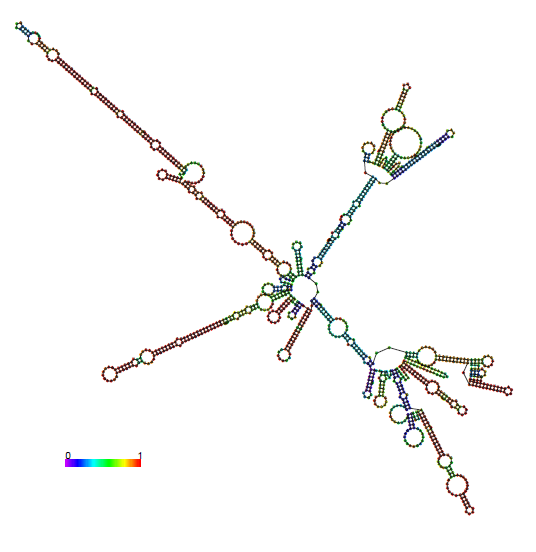 | 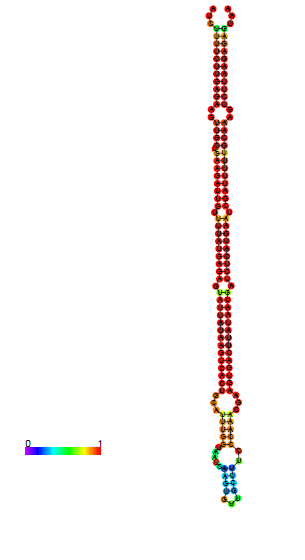 |
| TCONS_00080683 | bra-miR172b-5p |
| 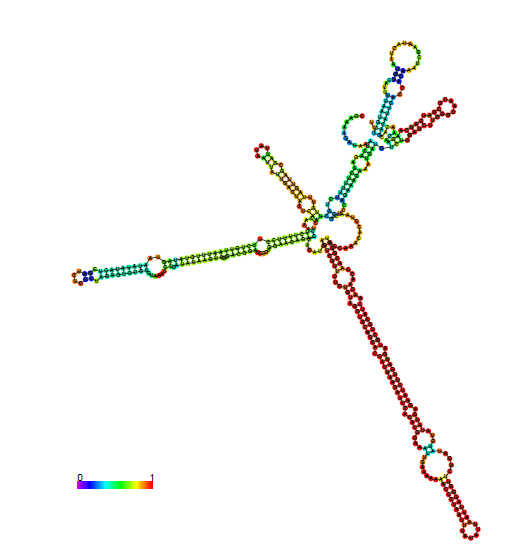 | 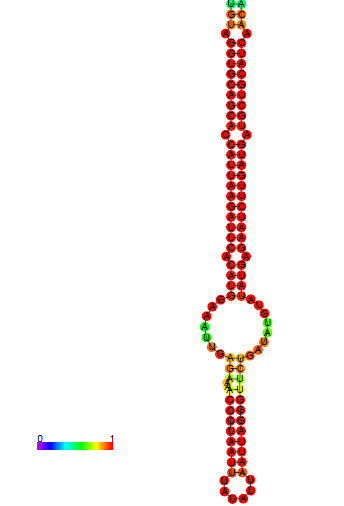 |
